# Synthesis and Evaluation of Small Molecule Inhibitors of the Androgen Receptor N-Terminal Domain

**DOI:** 10.1021/acsmedchemlett.3c00426

**Published:** 2023-11-17

**Authors:** Martyn C. Henry, Christopher M. Riley, Irene Hunter, Jessica M. L. Elwood, J. Daniel Lopez-Fernandez, Laura Minty, Diane M. Coe, Iain J. McEwan, Craig Jamieson

**Affiliations:** † Department of Pure and Applied Chemistry, University of Strathclyde, 295 Cathedral Street, Glasgow G1 1XL, U.K.; ‡ Institute of Medical Sciences, University of Aberdeen, Foresterhill, Aberdeen AB25 2ZD, U.K.; § Medicine Design, GlaxoSmithKline R&D Ltd, Gunnels Wood Road, Stevenage, Hertfordshire SG1 2NY, U.K.

**Keywords:** Prostate cancer, Androgen receptor, Intrinsically disordered protein

## Abstract

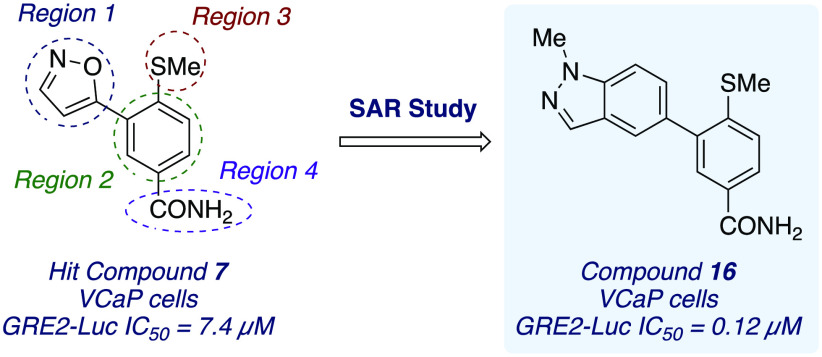

The androgen receptor (AR) is central to prostate cancer pathogenesis and has been extensively validated as a drug target. However, small-molecule anti-androgen therapies remain limited due to resistance and will eventually fail to suppress tumor growth, resulting in progression to castration-resistant prostate cancer (CRPC). The intrinsically disordered N-terminal domain (NTD) is crucial for AR transactivation and has been investigated as a suitable target in the presence of ligand binding domain mutations. A screening campaign identified biaryl isoxazole compound **7** as a weak inhibitor of the AR NTD. A library of biaryl analogues were synthesized, and their biological activities were assessed in a VCaP cell-based luciferase reporter gene assay. A structure–activity relationship (SAR) study revealed that indazole analogue **16** exhibited increased potency and favorable physicochemical properties with a benchmarked pharmacokinetic profile, providing a suitable starting point for further optimization of **16** as a CRPC therapeutic in the presence of AR mutations.

Prostate cancer remains the second most common cancer, with one in eight men receiving a diagnosis in their lifetime and almost 400,000 deaths worldwide in 2020 alone.^1^ Despite significant advances in localized prostate cancer therapies, around 20–30% of patients will subsequently present with advanced and metastatic forms of the disease and will require systemic treatment.^2−4^ While effective at inducing remission and offering a relief from symptoms, treatments such as androgen deprivation therapy (ADT) ultimately only slow the course of the disease before almost inevitable progression to castration-resistant prostate cancer (CRPC), which is associated with a poor prognosis.^5,6^ Central to prostate cancer pathogenesis is the nuclear androgen receptor (AR), a 110 kDa transcription factor which is primarily responsible for androgen-mediated regulation of gene expression.^7^ In common with all nuclear receptors, the AR protein consists of the ligand-binding domain (LBD), in which the ligand binding pocket (LBP) is located; the DNA-binding domain (DBD); the N-terminal domain (NTD); and a hinge region which connects the LBD with the DBD. Due to the critical role of the AR protein in the progression of prostate cancer, AR antagonists that compete with androgens for binding in the LBP are used clinically and have traditionally been the primary focus of drug discovery campaigns.^8−10^ In this regard, the second-generation nonsteroidal anti-androgens (NSAAs) enzalutamide (**1**), apalutamide (**2**), and darolutamide (**3**) have all been approved within the past decade for the treatment of CRPC (Figure 1).^11−13^ However, anti-androgen therapies will eventually fail to suppress tumor growth due to a complex array of resistance mechanisms, which inevitably leads to reactivation of the AR signaling axis.^14−19^ For this reason, alternative small-molecule CRPC therapies have been sought that do not rely on binding to the canonical LBD but instead target other domains of the AR.^20−22^ It has been shown that the NTD is critical for the transactivation and function of AR, in which the modular activation function (AF-1) is of particular importance for gene expression and in facilitating protein–protein interactions.^23,24^ However, due to the intrinsically disordered nature of this domain, it is not amenable to structure-based drug design and has therefore garnered less attention as a potential target for therapeutic intervention in the past.^25^ Despite this, in the past decade, several small molecules have been developed which act on the AR via the disordered NTD.^26−35^ For example, the EPI family of compounds,^36−39^ including racemic EPI-001 (**4**), have been reported to block AR transcription via interaction with the AF-1 region of the AR-NTD, while other EPI analogues (EPI-002 (**5**) and EPI-7170 (**6**)) have been shown to induce conformation changes within the NTD and disrupt interactions between the AF1 and essential protein coactivators.^40^ Furthermore, EPI-7386 (structure not disclosed) is currently in phase-1 clinical trials and has effectively demonstrated that AR NTD inhibitors could be potential therapeutics in the presence of LBD-driven resistance in the context of CPRC.^41,42^

**Figure 1:**
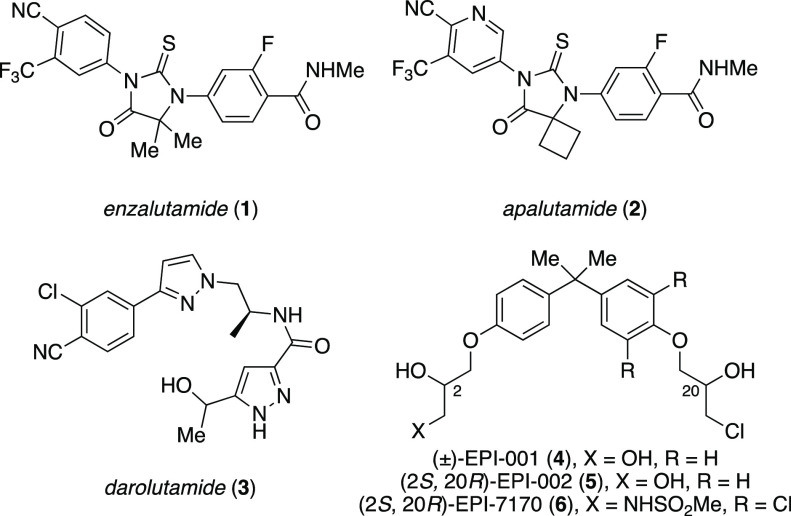
Clinically relevant nonsteroidal AR antagonists and recent AR-NTD AF1 binders.

Herein we report our work toward the design, synthesis, and biological evaluation of a novel series of biaryl small-molecule antagonists of the AR receptor NTD. Previous work within our laboratories identified compound **7** as a weak binder of AR splice variants (AR-vs) lacking the LBD using a high-throughput screen utilizing a functional cell-based assay developed in our group, with hits from this assay counterscreened for cytoxicity and inhibition of luciferase activity to confirm specific activity.^43^ In an effort to develop understanding of the structure–activity relationship (SAR) and identify more potent AR-NTD inhibitor analogues, we synthesized analogues of **7** focusing largely on changes to the LHS heterocyclic region, alternative substituents on the RHS aromatic ring, and replacement of the potentially metabolically labile thioether functional group (Figure 2a).

**Figure 2:**
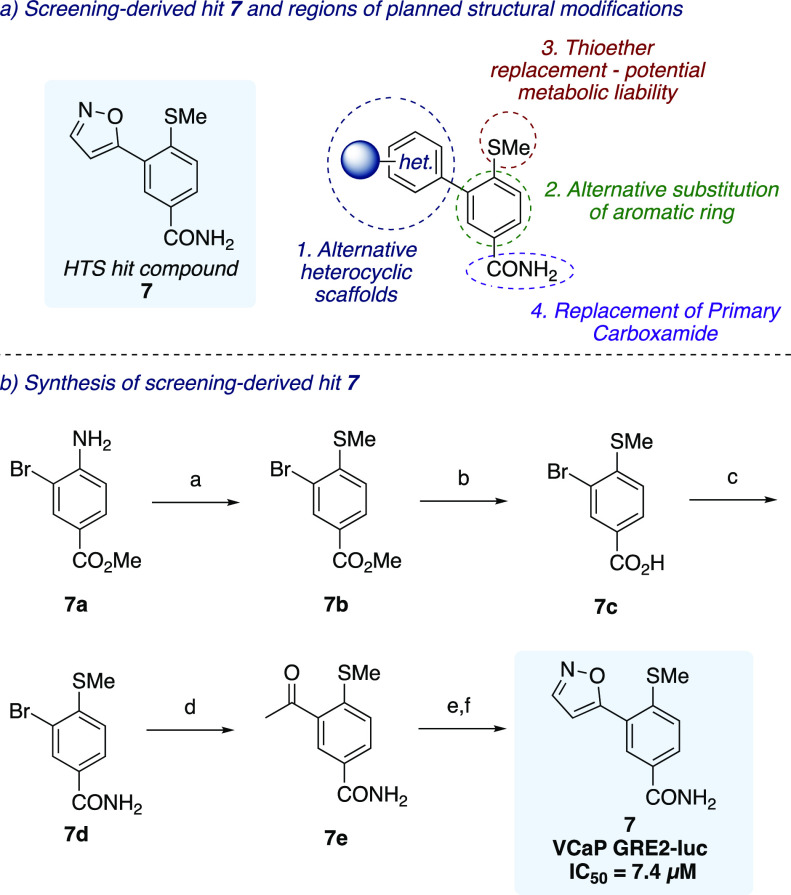
Proposed structural modifications and synthesis of initial HTS hit compound **7**. Reagents and conditions: (a) NaNO_2_, 1 M HCl, rt, 1 h, then NaSMe, rt, 2 h, 86%; (b) 4 M NaOH, THF/EtOH (1:1), rt, 16 h, quant.; (c) (COCl)_2_, DMF, THF, rt, 1 h, then NH_4_OH, 0 °C, 2 h, 74%; (d) ethyl vinyl ether, Pd(dppf)Cl_2_·CH_2_Cl_2_, K_2_CO_3_, DMF/H_2_O (9:1), 90 °C, 64 h, then 2 M HCl, rt, 0.5 h, 43%; (e) NaH, EtOCHO, rt, 24 h; (f) H_2_NOH·HCl, EtOH, 85 °C, 1 h, 25% over two steps.

Our current work began with the validation of our screening-derived hit compound **7** in a cell-based reporter gene assay. This required the synthesis of compound **7** from readily available starting materials, which is shown in Figure 2b. Aniline **7a** was converted to the corresponding diazonium salt by treatment with sodium nitrite, which was followed by a Sandmeyer-type reaction with sodium methanethiolate that provided thioether **7b** in 86% yield. Hydrolysis of the ester group followed by amidation via *in situ* formation of the Vilsmier reagent afforded primary carboxamide **7d**. Palladium-catalyzed Heck reaction with ethyl vinyl ether gave the intermediate enol ether, which was hydrolyzed under acidic conditions to afford ketone **7e** in 43% yield. Finally, formylation of the α-position of the carbonyl in the presence of sodium hydride followed by ring closure with hydroxylamine afforded isoxazole **7** in 25% yield over two steps. Compound **7** was then evaluated in our VCaP cell-based GRE2-luciferase reporter gene assay and was found to have an IC_50_ of 7.4 μM.

For reference, the benchmark AR antagonist enzalutamide (**1**) and EPI-001 (**4**) were also examined for their effect against GRE2-luciferase activity in our hands (Figure 1). In this assay, enzalutamide was very potent, with an IC_50_ of 0.34 μM, while EPI-001 had an IC_50_ of 37.4 μM.

As shown in Figure 2a, the four main regions of the template were targeted for further SAR study. In our initial investigations into modifications of the LHS region, the biaryl C–C bond was used as a linchpin disconnection, with alternative heterocyclic groups being introduced via Suzuki–Miyaura cross-coupling with aryl bromide **7d** and a range of boronic acids under thermal or microwave conditions (Table 1; see the Supporting Information for further details). Compound **8** with an unfunctionalized phenyl ring was inactive in our assay.

**Table 1:**
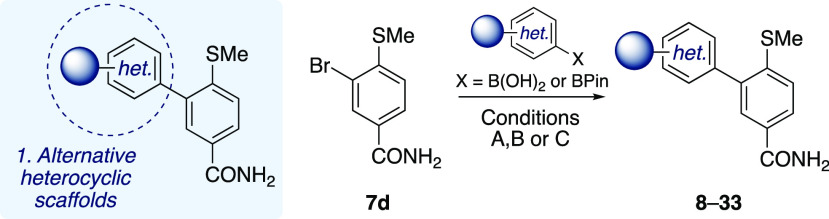

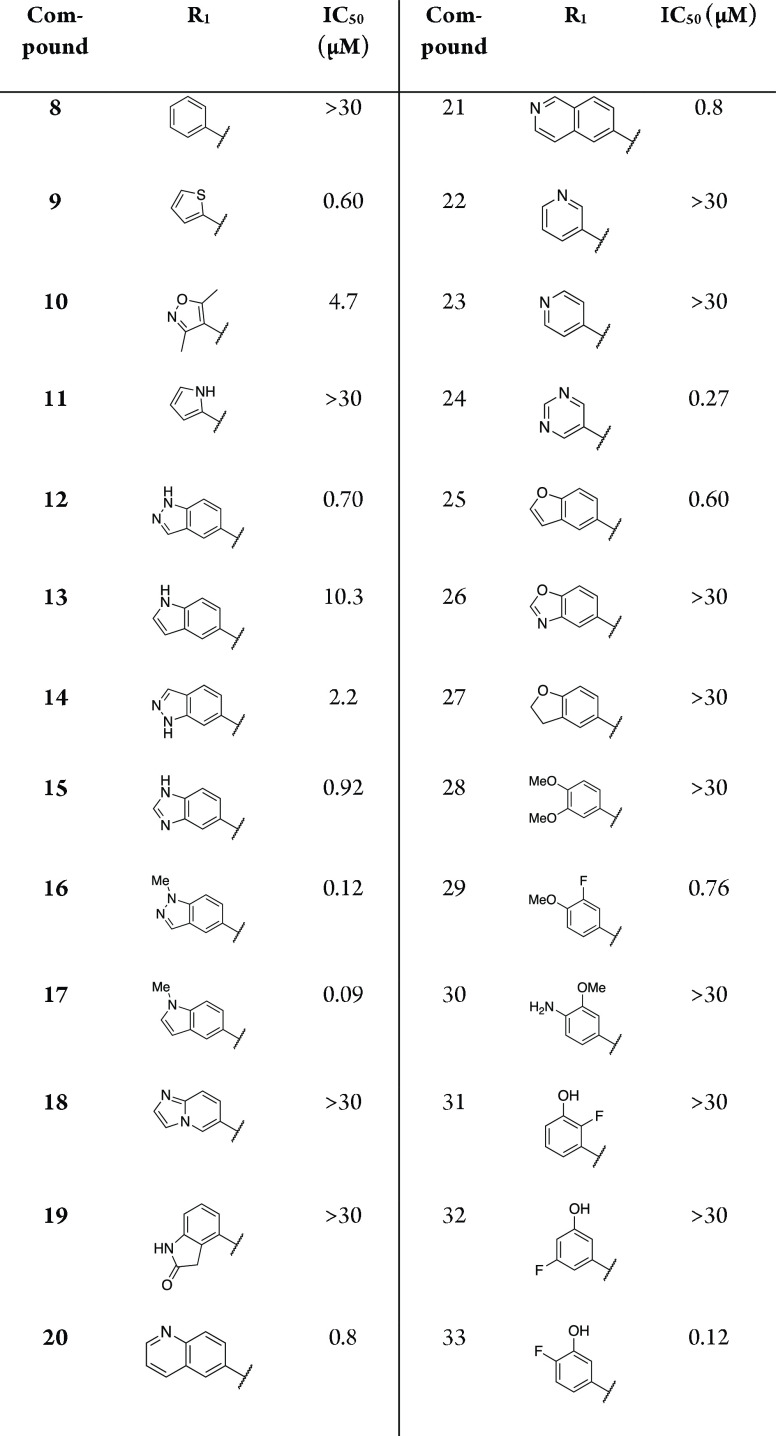
Region 1 Investigations into Alternative RHS Heterocyclic Scaffolds^a^

a Conditions: (A) XPhos-Pd-G2 (5 mol %), K_3_PO_4_, toluene/water (9:1), 90 °C, 16 h; (B) Pd(PPh_3_)_4_ (10 mol %), K_3_PO_4_, DMF/water (9:1), 90 °C, 16 h; (C) Pd(PPh_3_)_4_ (7.5 mol %), Cs_2_CO_3_, 1,4-dioxane/water (9:1), 120 °C, 20 min (see the Supporting Information).

Following this, the LHS region was explored via the introduction of smaller, five-membered heterocyclic motifs. Thiophene **9** showed robust activity (IC_50_ = 0.60 μM), while dimethylisooxazole **10** was weakly active (IC_50_ = 4.7 μM) and pyrrole **11** retained no activity. Next, benzannulated heterocycles bearing hydrogen-bond donors (HBDs) were examined. Indazole **12** showed good activity, with an IC_50_ of 0.70 μM, while indole **13** displayed a 10-fold decrease in activity (IC_50_ = 10.3 μM). Interestingly, indazole **14** featuring different connectivity compared with analogue **12**, such that the HBD is oriented in a different direction, was less potent (IC_50_ = 2.2 μM). Activity was recovered with imidazole **15**, which displayed an IC_50_ of 0.92 μM. Removing the HBD from the RHS heterocycle by blocking the free NH group of indazole **12** with a methyl substituent gave compound **16**, which exhibited excellent potency (IC_50_ = 0.12 μM). This represents a 60-fold increase in potency over our initial hit compound **7** and compares favorably with enzalutamide (IC_50_ = 0.34 μM). Deletion of a nitrogen atom resulted in approximately similar potency in *N*-methylindole **17** (IC_50_ = 0.09 μM). Imidazopyridine **18** and oxoindole **19** were tested; however, both displayed no activity. Quinoline **20** and isoquinoline **21** were active, both with an IC_50_ of 0.80 μM, demonstrating that the position of the nitrogen atom has no effect on the activity. However, deleting the aryl ring resulted in a loss of activity of pyridine analogues **22** and **23**. The addition of a nitrogen atom in pyrimidine analogue **24** resulted in recovery of activity with an IC_50_ of 0.27 μM. Our attention next focused on more lipophilic benzannulated heterocycles such as benzo[*b*]furan **25**, benzoxazole **26**, and 2,3-dihydrobenzofuran **27**. Of these compounds, only unsaturated benzo[*b*]furan **25** displayed activity (IC_50_ = 0.60 μM). Activity was abolished in electron-rich acyclic ether analogue **28** but recovered to an extent with the incorporation of a fluorine atom in analogue **29** (IC_50_ = 0.76 μM). Increasing the electron density of the ring and increasing the number of HBDs via the replacement of a methoxy group with an aryl amine resulted in loss of activity in compound **30**. We next examined fluorinated phenol isomers **31**, **32**, and **33**. Interestingly, the position of the fluorine atom on the aromatic ring significantly influenced the bioactivity, as *ortho* and *meta* analogues **31** and **32** were inactive while *para* derivative **33** was potent (IC_50_ = 0.12 μM).

Subsequently, in an effort to improve solubility and other physicochemical properties, a π-deficient pyridine ring was introduced into our scaffold in analogues **34**–**37** (Table 2). It is clear from matched molecular pairs **34** and **16** that the pyridine ring is not tolerated on the RHS, resulting in a loss of activity, perhaps due to unfavorable conformation changes or a decrease in electron density. Substitution of a hydrogen atom for a fluorine atom at the *ortho* position with respect to the thioether in compound **38** gave activity (IC_50_ = 0.24 μM) similar to that of compound **16**. Moving the thioether group around the ring to furnish *meta* substitution relative to the biaryl C–C bond resulted in an inactive compound (**39**).

**Table 2:**
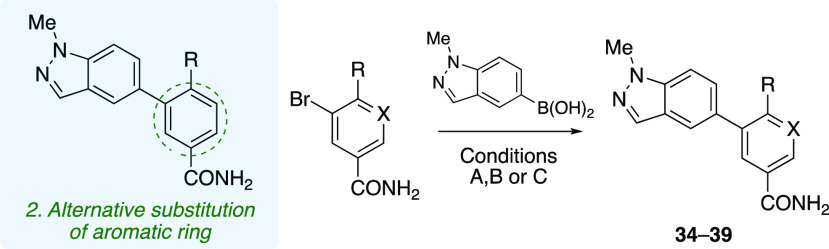
Region 2 Investigations into Alternative Substitutions of the LHS Aromatic Ring

compound	substitution	IC_50_ (μM)
**34**	R = SMe, X = N	>30
**35**	R = SBn, X = N	>30
**36**	R = H, X = N	>30
**37**	R = OMe, X = N	>30
**38**	R = SMe, X = CF	0.24
**39**	R = H, X = C–SMe	>30

Replacement of the methyl thioether functionality was next investigated, as it was reasoned that this could be a potential metabolic liability (Table 3). This required *de novo* synthesis from prefunctionalized starting materials (see the Supporting Information for experimental details). For example, ether starting materials were prepared by O-alkylation, while nitrogen functionality was introduced via nucleophilic aromatic substitution with the desired amine. Compounds **40**–**57** were then delivered by subjecting these starting materials to a palladium-catalyzed Suzuki–Miyaura cross-coupling with methylindazole boronic acid, as detailed previously.

**Table 3:**
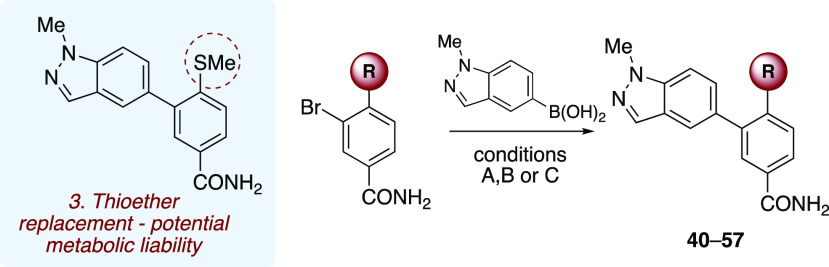

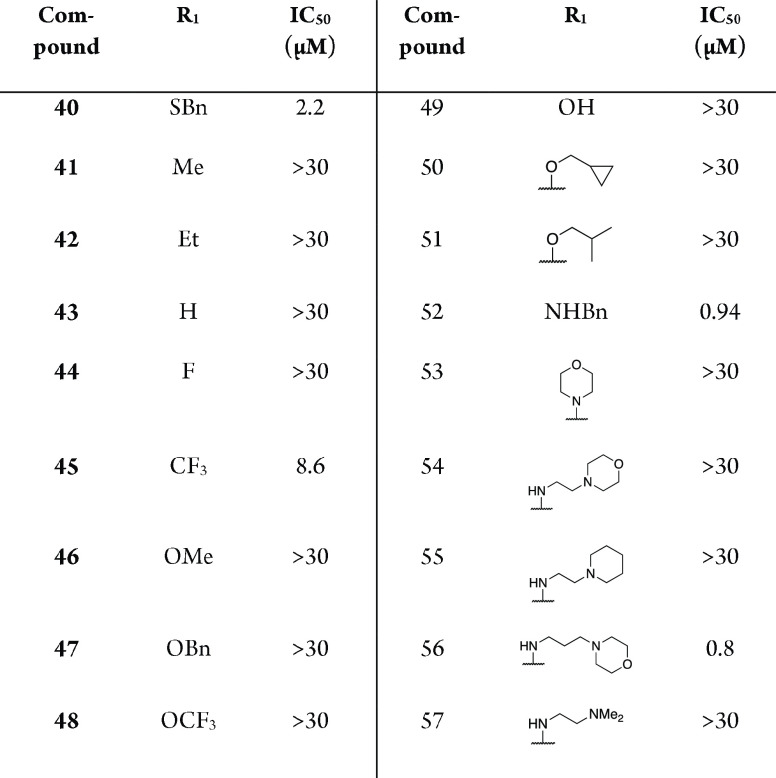
Region 3 Investigations into Potential Replacement of the Thioether Functional Group

First, replacement of the methyl group with a bulkier, more lipophilic benzyl group resulted in moderately potent compound **40** (IC_50_ = 2.2 μM), while substitution of the thioether with alkyl functionality (methyl **41** and ethyl **42**) resulted in loss of activity. Similarly, no bioactivity was observed for compound **43** after removal of the thioether and replacement with a hydrogen atom. While fluorinated analogue **44** was inactive, compound **45** bearing a trifluoromethyl group was weakly active, with an IC_50_ of 8.6 μM. Attempts to introduce ether functionality resulted in complete loss of activity, with methyl ether **46**, benzyl ether **47**, and trifluoromethyl ether **48** all displaying IC_50_ > 30 μM. Furthermore, a similar lack of activity was observed with free phenol derivative **49**, while bulkier ether analogues **50** and **51** were all inactive in our assay. Changing the C–O linkage to a C–N bond in compound **52** resulted in modest activity, with an IC_50_ of 0.94 μM. Alternative N-linked analogues were synthesized, including cyclic morpholine compound **53** and acyclic derivatives **54**–**57**. Only analogue **56** featuring a morpholine ring attached to the LHS aryl ring via a linear propyl amine chain was active in the assay, with an IC_50_ of 0.80 μM.

For reasons of synthetic tractability, molecular matched pairs of pyrimidine analogue **24** were investigated where the primary carboxamide was replaced with tertiary amides via coupling of carboxylic acid **58** with cyclic secondary amines (Table 4). In the VCaP GRE2-luciferase assay, carboxylic acid **58** was active, with an IC_50_ value of 0.12 μM, while tertiary amides **59** and **60** exhibited IC_50_ values of 0.13 and 0.26 μM, respectively. Compounds **59** and **60** displayed comparable potency to matched pair **24**, which suggests that the HBD capabilities of the primary amide are not crucial for binding to the AR.

**Table 4:**
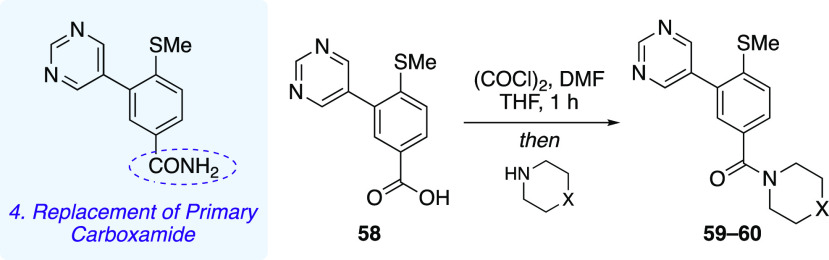
Region 4 Investigations into Potential Replacement of the Primary Carboxamide

compound	substitution	IC_50_ (μM)
**58**	CO_2_H	0.12
**59**	X = CH_2_	0.13
**60**	X = O	0.26

During the SAR investigation of the core scaffold, control of lipophilicity was maintained with the aim of keeping this parameter in the desired Lipinski-type space. A ligand lipophilicity (LLE) plot showing the effects of changes by region revealed no significant correlation between cellular potency and cLogP (see the Supporting Information for further information). The *in vitro* activity (IC_50_ = 0.12 μM) and favorable physicochemical properties placed methylindazole **16** within lead-like chemical space (Table 5). The lipophilicity (cLogP = 2.3), topological polar surface area (TPSA = 86), and numbers of HBDs (2) and hydrogen bond acceptors (HBAs) (4) were all within the desired property space associated with lead assets.^44^ The intrinsic property forecast index (iPFI), a metric proposed by Young and co-workers,^45^ is the sum of cLogP and the aromatic ring count, which is a predictor of important considerations such as solubility and off-target effects and should be kept <7. The iPFI of compound **16** is in the acceptable range, with a value of 5.3. Moreover, compound **16** has a ligand efficiency of 0.33 and a ligand-lipophilic efficiency of 4.7.

**Table 5:**
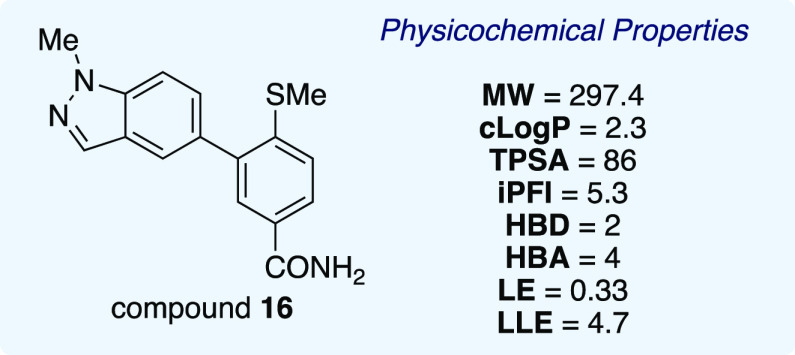
Physicochemical Properties and *In Vitro* Pharmacokinetic Profile of Compound **16**

VCaP GRE2-luciferase IC_50_	0.12 μM
solubility	2.6 μM
Caco2 permeability P_app_ A2B (nm/s); P_app_ B2A (nm/s)	331; 394
Caco2 efflux ratio	1.2
HLM CL_int_ (μL min^–1^ mg^–1^)	25.6
HLM *t*_1/2_ (min)	54
plasma fraction unbound (mouse)	6.9%
CYP IC_50_ (3A4; 2D6)	>25 μM
hERG IC_50_	>10 μM

Having demonstrated robust *in vitro* and physicochemical properties, we next sought to further probe the pharmacology of emerging lead compound **16**. To this end, AR-v splice variant activity was determined through transfection of PC3 cells with an AR_NTD–DBD_ expression plasmid and a GRE2-luciferase reporter gene (Figure 3).^43^ This construct retains the constitutively active NTD/DBD core; however, it is insensitive to LBD antagonists such as enzalutamide. Compound **16** shows 35% inhibition of AR-v activity at 10 μM, whereas enzalutamide is not active in this assay. In addition to this, we evaluated hormone-dependent expression of prostate-specific antigen (PSA) in VCaP cells (see the Supporting Information). This showed a similar level of inhibition (34%), confirming pathway-relevant pharmacological effects.

**Figure 3:**
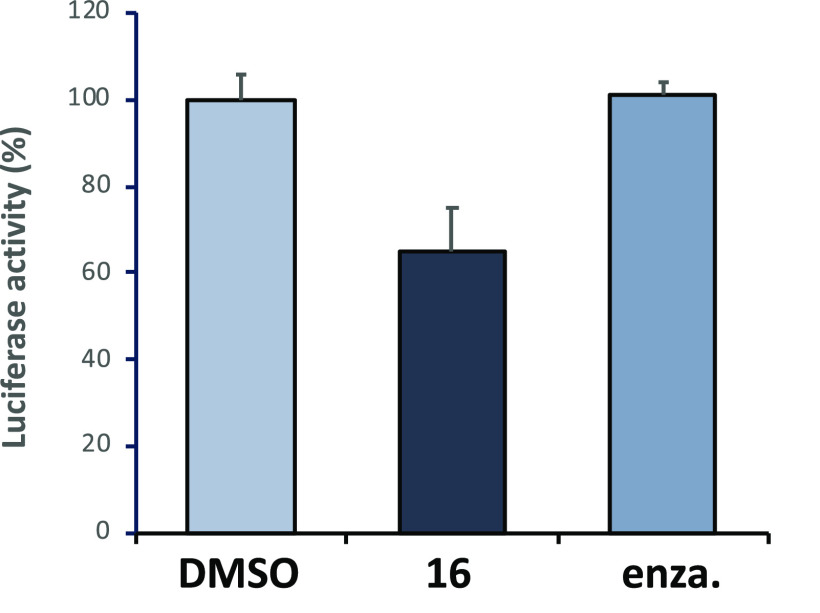
Small-molecule inhibition of androgen receptor transcriptional activity of compound **16** and enzalutamide (enza.).

We next explored the potential developability of this compound as a potent AR inhibitor with an investigation of the *in vitro* pharmacokinetic properties of **16** (Table 5). Compound **16** exhibited a moderate thermodynamic aqueous solubility of 2.6 μM and good Caco2 cell permeability (apical to basolateral of 33.1 nm/s and basolateral to apical of 39.4 nm/s), with an efflux ratio of 1.2. Furthermore, compound **16** displayed promising intrinsic clearance (CL_int_) in human liver microsomes and a half-life of 54 min. The plasma protein binding of **16** was determined and showed a free fraction of 6.9%. The potential for drug–drug interactions was next investigated, and no inhibition of CYP450 was observed (with both 34A and 2D6 isoforms), while no hERG channel inhibition was observed via a Q-patch clamp assay.

Encouraged by the promising *in vitro* profile, compound **16** was advanced, and a pharmacokinetic (PK) profile was obtained in male CD-1 mice (Table 6). When compound **16** was administered intravenously (iv) at 1 mg/kg, a short half-life (*t*_1/2_) of 0.29 h was observed along with a high clearance of 126 mL min^–1^ kg^–1^. The maximum plasma concentration (*C*_max_) was 432 ng/mL, while the area under the curve (AUC) was 131 h·ng/mL (as can be seen in the *C*_max_ versus time profile in Figure 4). Following an oral dose (p.o) of compound **16** at 10 mg/kg, a mean half-life of 4 h was observed along with adequate plasma exposure (*C*_max_ = 195 ng/mL) and measurable exposure (AUC = 173 h·ng/mL) with an oral bioavailability (*F*) of 16%, suggesting that target engagement may be difficult to achieve when considering the potency and exposure data.

**Table 6:** *In Vivo* PK Profile of Compound **16** in Mouse (*n* = 3)

PK parameters	iv (1 mg/kg)	po (10 mg/kg)
*t*_1/2_ (h)	0.29	4
*T*_max_	n/a	0.25
CL (mL min^–1^ kg^–1^)	126	n/a
*C*_max_ (ng/mL)	432	195
AUC_0–*t*_ (h·ng/mL)	131	173
AUC_0–∞_ (h·ng/mL)	132	208
*V*_ss_obs_ (L/kg)	1.38	n/a
*F* (%)	n/a	16

**Figure 4:**
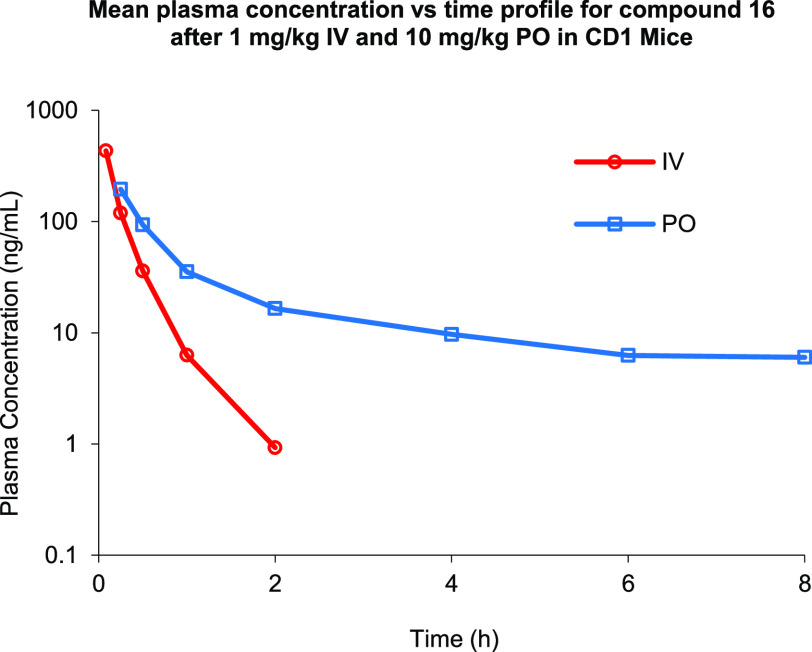
*C*_max_ versus time profile for compound **16**.

In summary, after identification and validation of initial hit **7** as a weak binder of the AR NTD, 50 analogues were synthesized to explore the SAR and optimize potency. Investigations into region 1 revealed that several benzannulated N-heterocycles were tolerated and had elevated activity compared to the initial hit. In particular, the *N*-methylindazole analogue **16** showed a 60-fold increase in potency over the progenitor compound **7**. Furthermore, compound **16** compared favorably in terms of potency to the clinically deployed AR antagonist enzalutamide (IC_50_ = 0.34 μM) and was more potent than known AR-NTD AF1 binder EPI-001 (IC_50_ = 37.4 μM) under the assay conditions. Together, the excellent potency, favorable physicochemical properties, and benchmarked pharmacokinetic profile offer a starting point for the further optimization of compound **16** as a tool compound to investigate the pharmacology and engagement of the AR NTD. Further optimization of this emerging chemical equity is underway in our laboratories, focusing on tuning the metabolic profile to attain exposure consistent with target engagement as well as biophysical studies of the interaction with the AR-NTD, and will be reported in due course.

## Supporting Information Available

Preparative details of all compounds and requisite intermediates, associated spectral data, and bioassay procedures (PDF)

## Abbreviations

ADT: androgen depravation therapy
AF-1: activation function-1
AR: androgen receptor
AUC: area under the curve
Cl_int_: intrinsic clearance
CRPC: castration-resistant prostate cancer
CYP: cytochrome P
DBD: DNA binding domain
HBA: hydrogen-bond acceptor
HBD: hydrogen-bond donor
LBD: ligand binding domain
LBP: ligand binding pocket
NSAA: nonsteroidal anti-androgen
NTD: N-terminal domain
iPFI: intrinsic property forecast index
SAR: structure–activity relationship
TPSA: topological polar surface area.
