# Supplementary material for: Synthesis and Evaluation of Small Molecule Inhibitors of the Androgen Receptor N-Terminal Domain
Source: ACS Med Chem Lett. 2023 Nov 17;14(12):1800–6. doi: 10.1021/acsmedchemlett.3c00426 (PMC10726465; doi:10.1021/acsmedchemlett.3c00426)

Supporting Information for:

Synthesis and Evaluation of Small Molecule Inhibitors of the Androgen Receptor *N*-Terminal Domain

Martyn C. Henry[1‡], Christopher M. Riley[1‡], Irene Hunter[2], Jessica. M. L. Elwood[1], J. Daniel Lopez-Fernandez[1], Laura Minty[1], Diane M. Coe[3], Iain J. McEwan[2]* and Craig Jamieson[1]*

1.Department of Pure and Applied Chemistry, University of Strathclyde, Glasgow G1 1XL, United Kingdom.
2. *Institute of Medical Sciences, University of Aberdeen, Foresterhill, Aberdeen, AB25 2ZD United Kingdom*
3. Medicine Design, GlaxoSmithKline R&D Ltd, Gunnels Wood Road, Stevenage, Herts, SG1 2NY United Kingdom

Table of Contents

1. Biological Materials and Methods **S2**
2. Overview of Analogues and Physicochemical Properties **S4**
3. Chemistry General Experimental **S10**
4. Synthesis of Initial Hit Compound **S12**
5. Synthesis of Starting Materials: Experimental Procedures and Spectroscopic Data **S16**
6. Synthesis of Final Compounds: Experimental Procedures and Spectroscopic Data **S31**
7. Copies of HPLC data for Final Compounds **S70**
8. Copies of ^{1}H and ^{13}C NMR spectra for Final Compounds **S96**

1. Biological Materials and Methods

Cell culture/plasmid

VCaP cells, obtained from the American Type Culture Collection, were maintained in Dulbecco's Modified Eagles Medium (DMEM) supplemented with 10% foetal bovine serum. Cells were maintained at 37°C without antibiotics in a humidified atmosphere containing 95% air and 5% CO_2.

The luciferase reporter plasmid, GRE2-TATA-luc has been described previously (Hay and McEwan, 2012).[1]

Transient transfection and Luciferase Activity Assay:

Cells at 70–80% confluence in 24 well plates, were transferred to DMEM supplemented with 5% charcoal stripped serum (CSS) for 24hr and transfected with 300ng/well of the firefly reporter plasmid, GRE2-TATA-luc using JetPEI (PolyPLUS Transfection) according to the manufacturers' protocol. After 24hr, the medium was replaced with fresh DMEM +5% CSS containing either DMSO (vehicle control) or 0–30 μM small molecules together with 1nM DHT. Twenty four hours later, medium was aspirated, cells were washed with PBS and extracted with Passive Lysis Buffer (Promega) for 15min at RT, with shaking. Cell debris was removed by centrifugation at 13,000 xg for 5min and supernatants collected. Luciferase activity was measured in triplicate, using an in-house luciferase assay buffer (13 mM $MgSO_4$ $7H_2O$, 30 mM GlyGly pH 7.8, 1.7 mM Na_2ATP and 11 μM luciferin (Invitrogen)). The protein concentrations of the samples were determined by the Bradford Assay (Bradford, M, 1976).[2] Luciferase activity, determined as relative light units, was normalized to the amount of total cellular protein.

Inhibition of hormone-dependent expression of prostate specific antigen (PSA):

VCaP prostate cells were treated with 1 nM DHT in the absence or presence of inhibitor. After 24 hours cells were harvested and PSA detected by Western blotting and normalised against β-actin. Under similiar conditions EPI-001 (50 μM) or enzalutamide (10μM) inhibited PSA expression by 19% and 82% respectively (not shown).

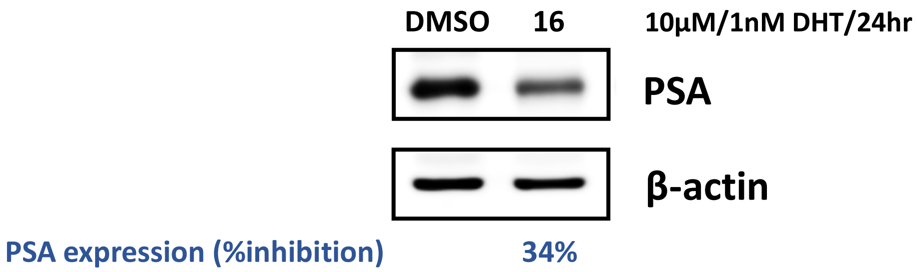

IC_{50} determination:

Luciferase activity in cells treated with small molecules was determined as a percentage of the untreated control, which was set at 100%. Plots of activity against small molecule concentration (log) were analysed in GraphPad PRISM 5 and IC_{50} values for each small molecule determined.

DMPK and physicochemical data were generated by Cyprotex Ltd, Alderley Park, Macclesfield, UK and Pharmaron Inc, Ningbo, China.

2. Overview of Analogues and Physicochemical Properties

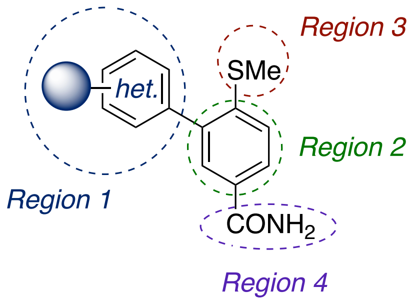

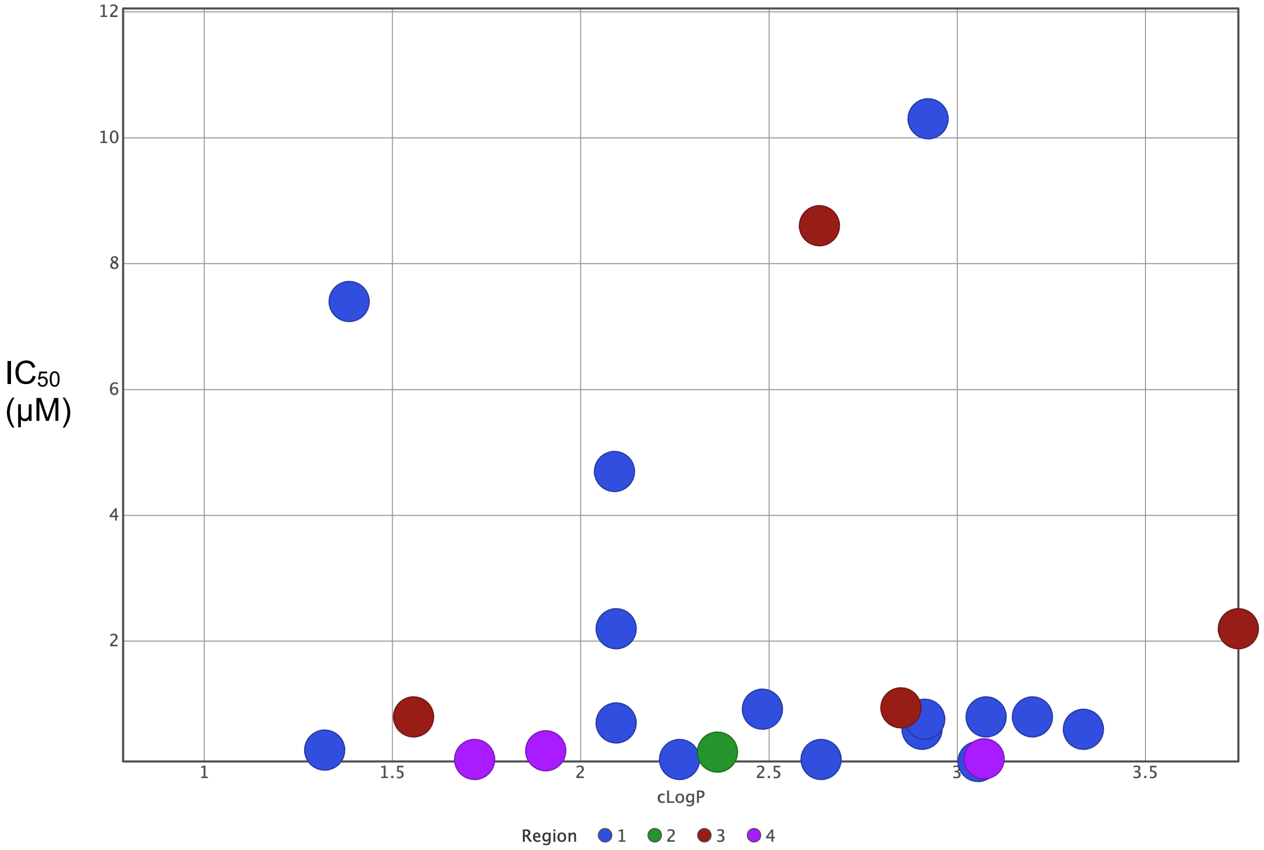

LLE plot: IC_{50} vs cLogP (Calculated using Datawarrior).[3]

	Structure	*in vitro* IC_{50} VCaP GRE2-luciferase (μM)	MW	cLogP	TPSA	Ligand Efficiency (LE)	Lipophilic Ligand Efficiency (LLE)
**7**	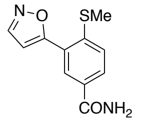	7.4	234.28	1.4	94.4	0.70	6.7
**8**	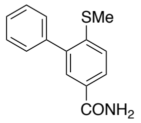	>30	243.33	2.9	68.4	0.61	4.6
**9**	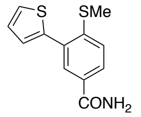	0.60	249.36	2.9	96.6	0.79	6.3
**10**	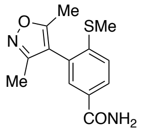	4.7	262.33	2.1	94.4	0.63	6.2
**11**	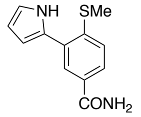	>30	232.31	1.7	84.2	0.65	5.8
**12**	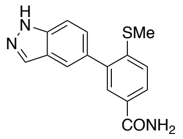	0.70	283.35	2.1	97.1	0.59	6.6
**13**	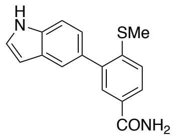	10.3	282.37	2.9	84.2	0.54	5.1
**14**	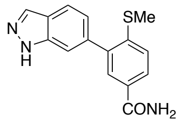	2.2	283.35	2.1	97.1	0.63	7.1
**15**	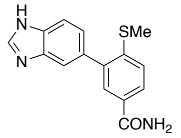	0.92	283.35	2.5	97.1	0.62	6.6
**16**	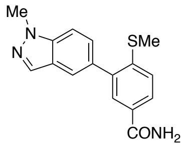	0.12	297.38	2.3	86.2	0.65	7.7
**17**	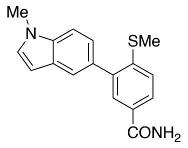	0.09	296.39	3.1	73.3	0.66	7.0

**18**	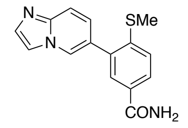	>30	283.35	1.8	85.7	0.52	5.7
**19**	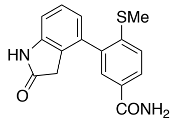	>30	298.37	2.0	97.5	0.49	5.5
**20**	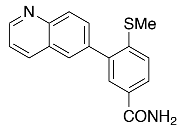	0.80	294.38	3.2	81.3	0.59	5.9
**21**	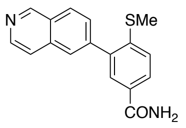	0.90	294.38	3.1	81.3	0.59	6.0
**22**	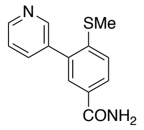	>30	244.32	1.9	81.3	0.61	5.6
**23**	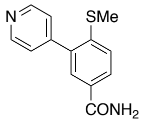	>30	244.32	1.9	81.3	0.61	5.6
**24**	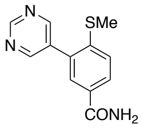	0.27	245.31	1.3	94.2	0.77	8.2
**25**	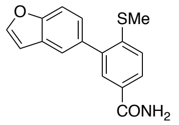	0.60	283.35	3.3	81.5	0.63	5.9
**26**	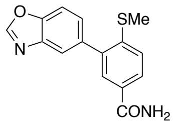	>30	284.34	2.9	94.4	0.52	4.6
**27**	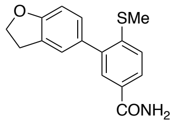	>30	285.37	3.1	77.6	0.52	4.4
**28**	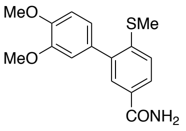	>30	303.38	2.7	86.9	0.49	4.8
**29**	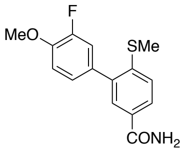	0.76	291.35	2.9	77.6	0.63	6.2

**30**	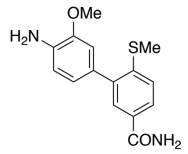	>30	288.37	2.1	103.6	0.52	5.4
**31**	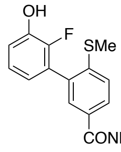	>30	277.32	2.64	88.6	0.54	4.9
**32**	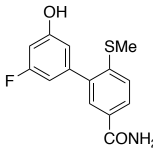	>30	277.32	2.6	88.6	0.54	4.9
**33**	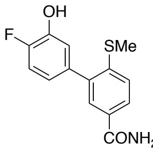	0.12	277.32	2.6	88.6	0.72	7.3
**34**	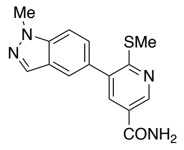	>30	298.37	1.4	99.1	0.49	6.2
**35**	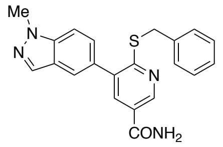	>30	374.47	2.8	99.1	0.38	4.7
**36**	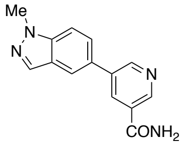	>30	252.28	0.8	73.8	0.54	6.7
**37**	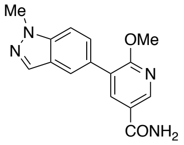	>30	282.30	1.1	83.0	0.49	6.5
**38**	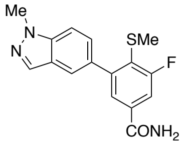	0.24	315.37	2.6	86.2	0.60	7.3
**39**	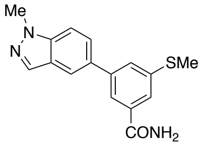	>30	297.2	2.3	86.2	0.49	5.3
**40**	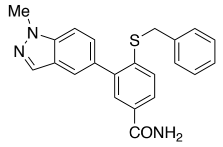	2.2	373.48	3.8	86.2	0.44	4.9

**41**	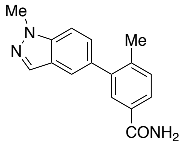	>30	265.32	2.1	60.9	0.52	5.4
**42**	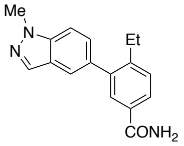	>30	279.34	2.5	60.9	0.49	5.0
**43**	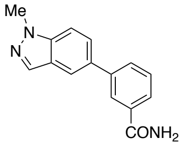	>30	251.29	1.8	60.9	0.54	5.7
**44**	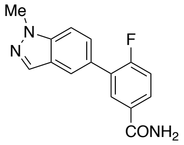	>30	269.28	1.9	60.9	0.52	5.6
**45**	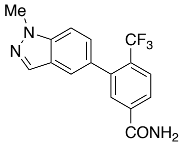	8.6	319.29	2.6	60.9	0.48	5.4
**46**	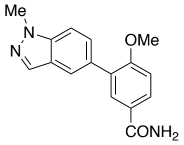	>30	281.31	1.7	70.1	0.49	5.8
**47**	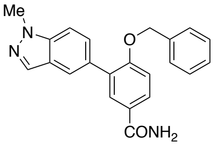	>30	357.41	3.1	70.1	0.38	4.4
**48**	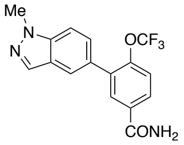	>30	335.28	2.9	70.1	0.43	4.6
**49**	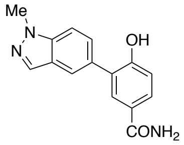	>30	267.29	1.4	81.1	0.52	6.1
**50**	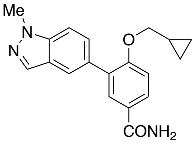	>30	321.38	2.4	70.1	0.43	5.1
**51**	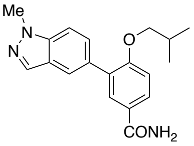	>30	323.40	2.8	70.1	0.43	4.7

**52**	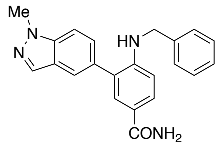	0.94	356.43	2.9	72.9	0.46	6.2
**53**	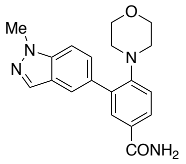	>30	336.39	1.5	73.4	0.41	6.1
**54**	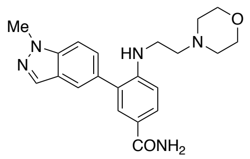	>30	379.46	1.1	85.4	0.37	6.4
**55**	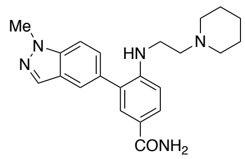	>30	377.49	2.3	76.2	0.37	5.3
**56**	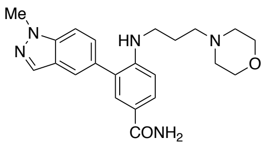	0.80	393.49	1.6	85.4	0.43	7.5
**57**	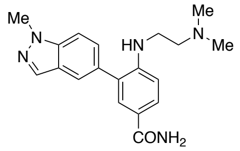	>30	337.42	1.1	76.2	0.41	6.4
**58**	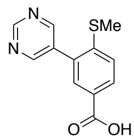	0.12	246.3	1.7	88.4	0.80	8.2
**59**	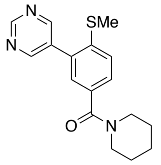	0.13	313.4	3.1	71.4	0.62	6.8
**60**	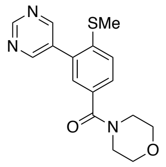	0.26	315.4	1.9	80.6	0.60	7.7

3. Chemistry General Experimental

All reagents and starting materials were obtained from commercial sources and used as received without further purification, unless otherwise stated. Acetone, dichloromethane, 1,4-dioxane, diethyl ether, *N*,*N*-dimethylformamide, ethanol, ethyl acetate, methanol and petroleum ether 40–60 °C were used as obtained from suppliers without further purification. All dry solvents were purified using a PureSolv SPS-400-5 Solvent Purification System.

All reactions were performed using round-bottom flasks or microwave vials of appropriate volume. Reactions were carried out at elevated temperatures using a temperature regulated hotplate/stirrer and DrySyn block with a contact thermometer. Room temperature generally refers to ~ 20 °C. Reactions under microwave irradiation were carried out using a Biotage® Initiator+ reactor. Reactions requiring a reduced temperature were performed using an ice bath (0 °C) with a temperature probe unless otherwise stated. Brine refers to a saturated aqueous solution of sodium chloride.

Reactions were monitored by thin layer chromatography (TLC) using Merck silica gel 60 covered aluminium backed plated F254. TLC plates were visualized under UV light and staining using potassium permanganate solution, vanillin or ninhydrin. Flash column chromatography was performed with Fluorochem silica gel 60 (40−63 μm). Reverse-phase HPLC purification was conducted using a Gilson preparative HPLC system of 322 pumps coupled to a 151 UV/Vis 163 spectrometer, 234 Autoinjector and a GX-271 liquid handler using a Waters XBridge Prep OBD C18 column (19 x 50 mm, 5 μm packing diameter) at room temperature. Purifications were performed using gradient methods ranging from 5–95 % acetonitrile in water over 30 minutes at a flow rate of 15 mL/min, with a 0.1% TFA modifier and UV monitoring at 254 nm. Analysis was conducted using Gilson Trilution v2.0 software.

Reverse phase HPLC data were obtained on an Agilent 1200 series HPLC using a Machery-Nagel Nucleodur C18 column using a gradient method 5–95 % acetonitrile (containing 0.1% TFA) in water (containing 0.1% TFA) over 18 minutes at a flow rate of 2 mL/min and UV monitoring at 250 nm.

Infrared spectra were recorded on a FTIR spectrometer; wavenumbers are indicated in cm^{-1}. ^{1}H, ^{19}F and ^{13}C NMR spectra were recorded on a Bruker DRX 500 NMR spectrometer at 500, 471 and 126 MHz, respectively or on a Bruker AV3 400 NMR spectrometer at 400, 376 or 101 MHz using the deuterated solvent as the internal deuterium lock. Chemical shifts (δ) are reported in ppm relative to the residual protic solvent where δ ($CDCl_3$) = 7.26 ppm (^{1}H) and δ ($CDCl_3$) = 77.16 ppm (^{13}C); δ (DMSO-d_6) = 2.50 ppm (^{1}H) and δ (DMSO-d_6) = 39.5 ppm (^{13}C); δ (acetone-d_6) = 2.05 ppm (^{1}H) and δ (acetone-d_6) = 29.9 ppm (^{13}C); δ (CD_3OD) = 3.31 ppm (^{1}H) and δ (CD_3OD) = 49.0 ppm (^{13}C). ^{1}H signals are described as singlets (s), doublets (d), triplets (t), quartets (q), multiplets (m), broad (br), app (apparent) or a combination of these and coupling constants are measured in Hz.

Low-resolution mass spectra were obtained using an Agilent Technologies 1200 series instrument with a 6130 single quadrupole LC/MS using a poroshell EC-C18 column. Analysis was performed using a gradient method, eluting with 5–95% acetonitrile (containing 5nM ammonium acetate)/water (containing 5nM ammonium acetate) over 18 minutes at a flow rate of 1 mL/min, with UV monitoring at 254 or 214 nm. High-resolution mass spectra were recorded using a Thermoscientific Exactive Plus equipped with a Vanquish LC.

All compounds are >95% pure by HPLC analysis.

Safety Statement: No unexpected or unusually high safety standards were encountered.

4. Synthesis of Initial Hit Compound

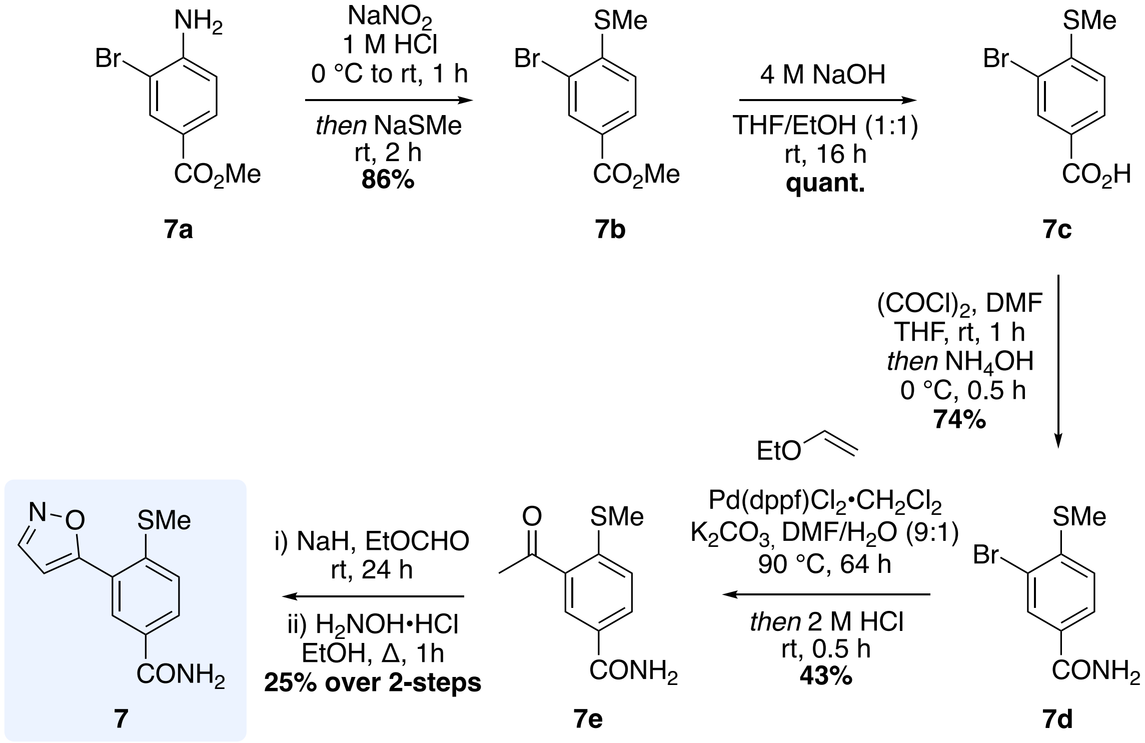

Methyl 3-bromo-4-(methylthio)benzoate (7b)

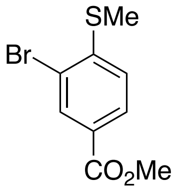

To a stirred suspension of methyl 3-bromo-4-aminobenzoate (**7a**) (3.00 g, 13.0 mmol) in 1 M aqueous hydrochloric acid (80 mL) was added sodium nitrite (0.897 g, 13.0 mmol) at 0 °C. The reaction mixture was warmed to room temperature and stirred for 1 hour. The reaction mixture was treated with sodium methanethiol (1.82 g, 26.0 mmol) and stirred for 2 hours. The mixture was diluted with ethyl acetate (50 mL), the phases were separated, and the aqueous phase was extracted with ethyl acetate (3 × 50 mL). The combined organic extracts were washed with water (100 mL), dried ($MgSO_4$), filtered and concentrated *in vacuo*. Purification by flash column chromatography, eluting with 5% ethyl acetate in petroleum ether, gave methyl 3-bromo-4-(methylthio)benzoate (**7b**) (2.94 g, 86%) as a yellow solid. FT-IR (neat) ν_{max} 3066, 3005, 2949, 2920, 2842, 1713, 1585, 1546, 1427, 1374, 1275, 1249, 1234, 1189, 1114, 1027 cm^{-1}; ^{1}H NMR (400 MHz, DMSO-d_6) δ 8.04 (d, J = 1.8 Hz, 1H), 7.92 (dd,

J = 8.4, 1.8 Hz, 1H), 7.39 (d, *J* = 8.4 Hz, 1H), 3.85 (s, 3H), 2.55 (s, 3H); ^{13}C NMR (101 MHz, DMSO-d_6) δ 164.9, 146.4, 132.3, 128.6, 126.6, 125.1, 119.7, 52.3, 14.7; HRMS (ESI) m/z: $[M + H]^+$ Calcd. for $C_9H_{10}{}^{79}BrO_2S$ 260.9579; Found 260.9580.

3-Bromo-4-(methylthio)benzamide (7d)

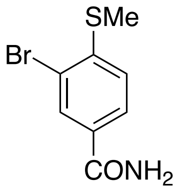

To a stirred solution of methyl 3-bromo-4-(methylthio)benzoate (**7b**) (1.72 g, 6.59 mmol) in methanol (20 mL) and tetrahydrofuran (20 mL) was added 4 M aqueous sodium hydroxide (5 mL) and the mixture was stirred at room temperature for 16 hours. The mixture was concentrated *in vacuo* and the residue was dissolved in water (20 mL). Concentrated hydrochloric acid was added dropwise and the resulting white solid was collected, washed with water and dried under high vacuum which gave 3-bromo-4-(methylthio)benzoic acid (**7c**) (1.76 g, 100%). 3-Bromo-4-(methylthio)benzoic acid (**7c**) (1.76 g, 7.12 mmol) was dissolved in anhydrous tetrahydrofuran (30 mL) and oxalyl chloride (2.0 mL) and *N*,*N*-dimethylformamide (2.5 mL) were added simultaneously. The resulting mixture was stirred at room temperature for 1 hour. The reaction mixture was cooled to 0 °C and 35% aqueous ammonia (12 mL) was added dropwise. The mixture was stirred for 1 h and then concentrated *in vacuo*. Water was added and the resulting precipitate was collected, washed with water and dried under high vacuum. This afforded 3-bromo-4-(methylthio)benzamide (**7d**) (1.30 g, 74%) as a white solid. FT-IR (neat) v_{max} 3374, 3184, 1622, 1605, 1586, 1537, 1249, 1112, 1024 cm^{-1}; ^{1}H NMR (400 MHz, DMSO-d_6) δ 8.08 (d, *J* = 1.8 Hz, 1H), 8.02 (br s, 1H), 7.89 (dd, *J* = 8.3, 1.8 Hz, 1H), 7.41 (br s, 1H), 7.32 (d, *J* = 8.3 Hz, 1H), 2.53 (s, 3H); ^{13}C NMR (101 MHz, DMSO-d_6) δ 166.0, 143.5, 131.4, 131.1, 127.2, 124.8, 119.6, 14.7; HRMS (ESI) m/z: $[M + H]^+$ Calcd. for $C_8H_9{}^{79}BrNOS$ 245.9583; Found 245.9577.

3-Acetyl-4-(methylthio)benzamide (**7e**)

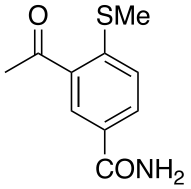

To a microwave vial was added 3-bromo-4-(methylthio)benzamide (**7d**) (49.2 mg, 0.200 mmol), [1,1′- bis(diphenylphosphino)ferrocene]dichloropalladium(II), dichloromethane complex (16.3 mg, 20.0 μmol), potassium carbonate (41.5 mg, 0.300 mmol), and ethyl vinyl ether (200 μL, 1.09 mmol), followed by 9:1 dimethylformamide:water (2 mL). The vial was capped and stirred at 90 °C for 64 hours. The mixture was cooled to room temperature and 2 M aqueous hydrochloric acid was added dropwise (2 mL). The mixture was stirred at room temperature for 30 minutes, then filtered over celite, diluted with ethyl acetate and saturated aqueous sodium carbonate, and the organic phase separated. The aqueous phase was extracted with ethyl acetate and the combined organic phases were washed with 5% w/v aqueous lithium chloride (× 5), dried (Na_2SO_4), filtered, and concentrated *in vacuo*. Flash column chromatography, eluting with petroleum ether/ethyl acetate 1:2 to 0:1, afforded 3-acetyl-4-(methylthio)benzamide (**7e**) (17.8 mg, 0.0851 mmol, 43%) as an off-white solid. FT-IR (neat) ν_{max} 3435, 3351, 3293, 3167, 1677, 1655, 1603, 1545, 1417, 1381, 1359, 1307, 1269, 1243, 1059 cm^{-1}; ^{1}H NMR (500 MHz, DMSO-d_6) δ 8.45 (d, *J* = 1.9 Hz, 1H), 8.14 (br s, 1H), 8.03 (dd, *J* = 8.4, 1.9 Hz, 1H), 7.50–7.44 (m, 2H), 2.63 (s, 3H), 2.42 (s, 3H); ^{13}C NMR (126 MHz, DMSO-d_6) δ 198.4, 166.7, 145.8, 133.0, 131.1, 130.3, 129.2, 124.7, 28.2, 15.1; HRMS (ESI) m/z: $[M + H]^+$ Calcd. for $C_{10}H_{12}NO_2S$ 210.0583; Found 210.0580.

3-(Isoxazol-5-yl)-4-(methylthio)benzamide (**7**)

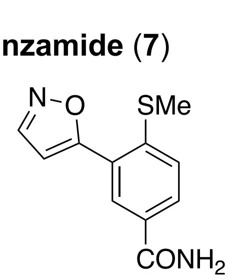

To a suspension of 3-acetyl-4-(methylthio)benzamide (**7e**) (36.0 mg, 0.172 mmol) in ethyl formate (3 mL) was added sodium hydride (75.0 mg, 1.88 mmol; 60% in mineral oil). The mixture was stirred at room temperature for 24 hours, quenched with methanol (3 mL) and concentrated *in vacuo*. The residue was diluted with ethyl acetate

and 1 M aqueous hydrochloric acid, and the organic phase was separated. The aqueous phase was extracted with ethyl acetate, and the combined organic extracts were dried (Na_2SO_4), filtered, and concentrated *in vacuo*. The resulting residue was dissolved in ethanol (3 mL) and hydroxylamine hydrochloride (59.8 mg, 0.861 mmol) in water (0.5 mL) was added. The mixture was stirred at 85 °C for 1 hour and concentrated *in vacuo*. The residue was diluted with ethyl acetate and saturated aqueous sodium bicarbonate, and the organic phase was separated. The aqueous phase was extracted with ethyl acetate and the combined organic extracts were dried (Na_2SO_4), filtered, and concentrated *in vacuo*. Purification by flash column chromatography, eluting with petroleum ether/ethyl acetate 1:2, afforded 3-(isoxazol-5-yl)-4-(methylthio)benzamide (**7**) (9.90 mg, 0.0423 mmol, 25%) as a yellow solid. FT-IR (neat) ν_{max} 3416, 3170, 2925, 1653, 1623, 1603, 1522, 1471, 1385, 1206, 1165, 1122, 1089, 1039 cm^{-1}; ^{1}H NMR (500 MHz, acetone-*d*$_6$) δ 8.55 (d, *J* = 1.8 Hz, 1H), 8.29 (d, *J* = 2.0 Hz, 1H), 8.04 (dd, *J* = 8.4, 2.0 Hz, 1H), 7.65 (br s, 1H), 7.55 (d, *J* = 8.4 Hz, 1H), 6.92 (d, *J* = 1.8 Hz, 1H), 6.70 (br s, 1H), 2.62 (s, 3H); ^{13}C NMR (101 MHz, DMSO-*d*$_6$) δ 166.6, 166.2, 151.4, 141.7, 130.4, 129.4, 128.0, 125.0, 124.1, 103.7, 14.7; HRMS (ESI) m/z: $[M + H]^+$ Calcd. for $C_{11}H_{11}N_2O_2S$ 235.0536; Found 235.0532.

5. Synthesis of Starting Materials: Experimental Procedures and Spectroscopic Data

General Procedure 1: Ester Hydrolysis

To a suspension of ester in 1:1 ethanol:tetrahydrofuran (0.250 mM) was added excess 4 M aqueous sodium hydroxide. The mixture was stirred at either room temperature or heated until completion as monitored by TLC. The mixture was concentrated *in vacuo*, diluted with water, acidified with concentrated hydrochloric acid, and the precipitate collected by vacuum filtration. The resulting solid was of sufficient purity and was used immediately in the next step.

General Procedure 2: Amide Bond Formation

To a suspension of carboxylic acid (1.00 equiv.) in anhydrous tetrahydrofuran (50.0 mM) was added oxalyl chloride (3.00 equiv.) and a catalytic amount of *N*,*N*-dimethylformamide, and the mixture was stirred at room temperature for 1 hour. The mixture was cooled to 0 °C, and ammonia (33% in water, excess) was added. The mixture was stirred at room temperature for 1 hour then concentrated *in vacuo*. If the residue was a solid it was suspended in water, sonicated, and collected *via* vacuum filtration. If the residue was an oil, it was diluted with ethyl acetate and water and the organic phase separated. The aqueous phase was extracted with ethyl acetate, the combined organic phase was dried (sodium sulfate), filtered, and concentrated *in vacuo*. The resulting residue was purified by flash column chromatography.

General Procedure 3: Nucleophilic Aromatic Substitution (Pyridine Derivatives)

To a stirred suspension of 5-bromo-6-chloronicotinamide (**S1**) (1 equiv.) and potassium carbonate (2.5 equiv.) in anhydrous tetrahydrofuran (40 mM) was added the appropriate nucleophile (5 equiv.). The reaction mixture was stirred under reflux for 16 h. After cooling to room temperature, the mixture was concentrated in vacuo and the resulting crude residue was purified by flash column chromatography to afford the desired S_NAr adduct.

General Procedure 4: *O*-Alkylation

To a stirred solution of alcohol (1 equiv.) in *N*,*N*-dimethylformamide (800 mM) was added potassium carbonate (3 equiv.). The resulting solution was stirred at room temperature for 1 hour, then alkyl bromide (3 equiv.) was subsequently added. The mixture was stirred at 60 °C for 3 hours, then cooled to room temperature and diluted with ethyl acetate (50 mL) and water (50 mL). The phases were separated, and the aqueous phase was extracted with ethyl acetate (2 × 50 mL). The combined organic extracts were washed with water (50 mL), dried ($MgSO_4$), filtered and concentrated *in vacuo*. The product was used as crude without further purification.

General Procedure 5: Nucleophilic Aromatic Substitution with *N*-Nucleophiles

To a stirred suspension of 3-bromo-4-fluorobenzamide (**S12**) (1 equiv.) and potassium carbonate (2.50 equiv.) in *N*,*N*-dimethylformamide (500 mM) was added the appropriate amine (1.2 equiv.). The reaction mixture was stirred at 120 °C for 8h, an additional portion of amine (1.2 equiv.) was added, and the mixture was stirred 120 °C for a further 24 h. After cooling to room temperature, the reaction mixture was quenched by the addition of saturated aqueous ammonium chloride (2 mL) and extracted with ethyl acetate (3 × 20 mL). The combined organic extracts were washed with brine (2 × 30 mL), dried ($MgSO_4$), filtered, concentrated *in vacuo*, and the resulting residue was purified by flash column chromatography.

5-Bromo-6-chloronicotinamide (S1)

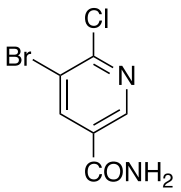

The reaction was performed as described in general procedure 1 using methyl 5-bromo-6-chloronicotinate (1.00 g, 4.00 mmol) which afforded 5-bromo-6-chloronicotinic acid (917 mg, 97%) as a beige solid which was used immediately in the next step. Following general procedure 2 using 5-bromo-6-chloronicotinic acid (914 mg, 3.87 mmol) afforded 5-bromo-6-chloronicotinamide (**S1**) (0.525 g, 58%) as a beige solid. ^{1}H NMR (500 MHz, DMSO-d_6) δ 8.83 (d, J = 1.8 Hz, 1H), 8.60 (d, J =

1.9 Hz, 1H), 8.25 (s, 1H), 7.79 (s, 1H); ^{13}C NMR (126 MHz, DMSO-d_6) δ 164.0, 151.6, 147.6, 141.6, 130.4, 119.2; LCMS (ESI) m/z: [M − H]$^+$ Calcd. for $C_6H_3{}^{79}Br^{35}ClN_2O$ 232.9; Found 233.1 at 6.24 mins.

5-Bromo-6-(methylthio)nicotinamide (S2)

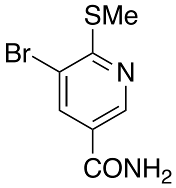

The reaction was performed as described in general procedure 2 using 5-bromo-6-chloronicotinamide (**S1**) (100 mg, 0.425 mmol), potassium carbonate (147 mg, 1.06 mmol) and sodium methane thiolate (149 mg, 2.12 mmol). Purification by flash column chromatography (petroleum ether/ethyl acetate, 1:4) afforded 5-bromo-6-(methylthio)nicotinamide (**S2**) (84.2 mg, 80%) as a white solid. ^{1}H NMR (500 MHz, $CDCl_3$) δ 8.77 (d, *J* = 2.0 Hz, 1H), 8.16 (d, *J* = 2.0 Hz, 1H), 5.82 (s, 2H), 2.59 (s, 3H); ^{13}C NMR (126 MHz, $CDCl_3$) δ 166.0, 164.2, 145.8, 137.9, 125.2, 118.9, 49.4; LCMS (ESI) m/z: [M + H]$^+$ Calcd. for $C_7H_8{}^{79}BrN_2OS$ 246.9; Found 247.1 at 6.78 mins.

6-(Benzylthio)-5-bromonicotinamide (S3)

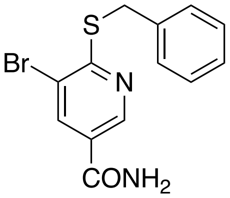

The reaction was performed as described in general procedure 2 using 5-bromo-6-chloronicotinamide (**S1**) (100 mg, 0.425 mmol), potassium carbonate (147 mg, 1.06 mmol) and benzyl mercaptan (0.750 mL, 6.37 mmol). An additional portion of benzyl mercaptan (0.250 mL, 2.13 mmol) was added and the reaction mixture was stirred under reflux for a further 2 h. Purification by flash column chromatography (petroleum ether/ethyl acetate, 3:2 to 0:1) afforded 6-(benzylthio)-5-bromonicotinamide (**S3**) (131 mg, 96%) as a colorless solid. ^{1}H NMR (500 MHz, $CDCl_3$) δ 8.78 (d, *J* = 2.0 Hz, 1H), 8.16 (d, *J* = 2.0 Hz, 1H), 7.42 (d, *J* = 7.3 Hz, 2H), 7.31 (t, *J* = 7.3 Hz, 2H), 7.27 (s, 1H), 5.81 (s, 2H), 4.47 (s, 2H); ^{13}C NMR (126 MHz, $CDCl_3$) δ 165.9, 163.4, 145.8, 138.2,

136.9, 129.2 (2 × CH), 128.6 (2 × CH), 127.4, 125.5, 118.5, 35.8; LCMS (ESI) m/z: [M + H]$^+$ Calcd. for $C_{13}H_{12}{}^{79}BrN_2OS$ 323.0; Found 323.0 at 8.18 mins.

5-Bromo-6-methoxynicotinamide (S4)

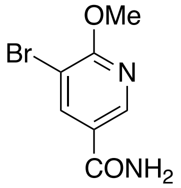

The reaction was performed as described in general procedure 2 using 5-bromo-6-chloronicotinamide (**S1**) (113 mg, 0.480 mmol) and sodium methoxide (51.9 mg, 0.961 mmol). A further portion of sodium methoxide (60.0 mg, 1.11 mmol) was added after 16 h and the reaction was stirred under reflux for a further 4 h. Purification by flash column chromatography (ethyl acetate) afforded 5-bromo-6-methoxynicotinamide (**S4**) as a colorless solid. ^{1}H NMR (500 MHz, $CDCl_3$) δ 8.53 (d, *J* = 2.2 Hz, 1H), 8.29 (d, *J* = 2.2 Hz, 1H), 5.84 (s, 2H), 4.07 (s, 3H); ^{13}C NMR (126 MHz, $CDCl_3$) δ 166.1, 162.4, 145.2, 141.2, 124.0, 107.4, 55.3; LCMS (ESI) m/z: [M + H]$^+$ Calcd. for $C_7H_8{}^{79}BrN_2O_2$ 231.0; Found 231.1 at 6.19 mins.

Methyl 3-bromo-5-fluoro-4-(methylthio)benzoate (**S5**)

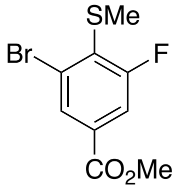

A solution of methyl 4-amino-3-bromo-5-fluorobenzoate (543 mg, 2.19 mmol) in dimethyl disulfide (5 mL) was heated to 70 °C under an inert atmosphere. *tert*-Butyl nitrite (700 µL, 5.89 mmol) was added and the mixture was stirred at 95 °C for 16 hours under an inert atmosphere and then concentrated *in vacuo*. Purification by flash column chromatography (petroleum ether/ethyl acetate, 99:1 to 49:1) followed by trituration in hexane afforded methyl 3-bromo-5-fluoro-4-(methylthio)benzoate (**S5**) (470 mg, 1.68 mmol, 77%) as a white solid. FT-IR (neat) ν_{max} 3081, 2962, 2932, 1731, 15551, 1426, 1396, 1294, 1217, 1173, 1122 cm^{-1}; ^{1}H NMR (400 MHz, acetone-d_6) δ 8.01–7.99 (m, 1H, H-e), 7.69 (dd, *J* = 10.2, 1.7 Hz, 1H, H-g), 3.91 (s, 3H, H-i), 2.60 (d, *J* = 2.5 Hz, 3H, H-c); ^{13}C NMR (101 MHz, acetone-d_6) δ 164.8 (d, J = 1.9 Hz), 163.0 (d, *J* = 249.2 Hz), 133.0 (d, *J* = 19.6 Hz), 132.1 (d, *J* = 9.0 Hz), 130.1 (d, *J* = 3.3 Hz), 127.7, 116.7 (d, *J* = 26.5 Hz), 53.1, 18.0 (d, *J* = 9.4 Hz); ^{19}F NMR (376 MHz, acetone-

d_6) δ −103.04–−103.11 (m); HRMS (ESI) Calcd. for $C_9H_9{}^{79}BrFO_2S$ [M + H]$^+$, 278.9485, found 278.9491.

3-Bromo-5-fluoro-4-(methylthio)benzamide (S6)

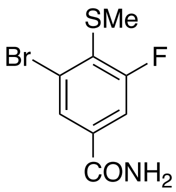

The reaction was performed as described in general procedure 1 using methyl 3-bromo-5-fluoro-4-(methylthio)benzoate (**S5**) (251 mg, 0.900 mmol) which afforded 3-bromo-5-fluoro-4-(methylthio)benzoic acid (217 mg, 91%) as a colorless solid which was used immediately in the next step. Following general procedure 2 using 3-bromo-5-fluoro-4-(methylthio)benzoic acid (196 mg, 0.740 mmol) afforded 3-bromo-5-fluoro-4-(methylthio)benzamide (**S6**) (177 mg, 91%) as a colorless solid. FT-IR (neat) ν_{max} 3381, 3191, 1653, 1621, 1534, 1454, 1396, 1240, 1180, 1128 cm^{-1}; ^{1}H NMR (400 MHz, DMSO-d_6) δ 8.13 (br s, 1H), 8.03–8.00 (m, 1H), 7.73 (dd, *J* = 10.5, 1.7 Hz, 1H), 7.64 (br s, 1H), 2.53 (d, *J* = 2.1 Hz, 3H); ^{13}C NMR (101 MHz, DMSO-d6) δ 164.7, 161.7 (d, *J* = 248.1 Hz), 135.7 (d, *J* = 7.9 Hz), 128.8 (d, *J* = 19.7 Hz), 127.6 (d, *J* = 2.8 Hz), 127.0, 114.5 (d, *J* = 25.9 Hz), 17.6 (d, *J* = 7.9 Hz); ^{19}F NMR (376 MHz, DMSO-d_6) δ −101.90–−101.96 (m); HRMS (ESI) Calcd. for $C_8H_8{}^{79}BrFNOS$ [M + H]$^+$, 263.9489., found 263.9487.

Methyl 3-bromo-5-(methylthio)benzoate (**S7**)

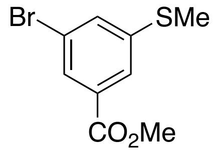

To a suspension of methyl 3-amino-5-bromobenzoate (1.15 g, 5.00 mmol) in 1 M aqueous hydrochloric acid (20 mL) was added sodium nitrite (345 mg, 5.00 mmol) and the mixture was stirred for 1 hour. Sodium thiomethoxide (701 mg, 10.0 mmol) was added and the mixture was stirred for 10 minutes. The mixture was diluted with ethyl acetate and water and the organic phase separated. The aqueous phase was extracted with ethyl acetate and the combined organic phase was dried (sodium sulfate), filtered, and concentrated *in vacuo*. Purification by flash column chromatography (petroleum ether/ethyl acetate, 19:1) afforded methyl 3-bromo-5-

(methylthio)benzoate (**S7**) (814 mg, 62%) as a yellow oil. FT-IR (neat) ν_{max} 3074, 2993, 2952, 2923, 2839, 1723, 1558, 1437, 1265, 1192, 1133, 1102 cm^{-1}; ^{1}H NMR (400 MHz, acetone-d_6) δ 7.82 (app. t, *J* = 1.6 Hz, 1H), 7.79 (app. t, *J* = 1.6 Hz, 1H), 7.63 (app. t, *J* = 1.6 Hz, 1H), 3.90 (s, 3H), 2.58 (s, 3H); ^{13}C NMR (101 MHz, acetone-d_6) δ 165.6, 143.5, 133.4, 132.8, 128.9, 126.1, 123.4, 52.9, 15.2; HRMS (ESI) Calcd. for $C_9H_{10}{}^{79}BrO_2S$ [M + H]$^+$, 260.9579., found 260.9575.

3-Bromo-5-(methylthio)benzamide (S8)

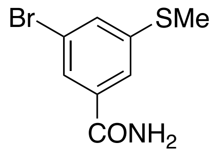

The reaction was performed as described in general procedure 1 using methyl 3-bromo-5-(methylthio)benzoate (**S7**) (755 mg, 2.89 mmol) which afforded 3-bromo-5-(methylthio)benzoic acid (676 mg, 95%) as a yellow solid which was used immediately without further purification. 3-Bromo-5-(methylthio)benzamide (**S8**) was then synthesised according to general procedure 2 using 3-bromo-5-(methylthio)benzoic (650 mg, 2.63 mmol). Purification by flash column chromatography (petroleum ether/ethyl acetate, 2:1 to 1:1) afforded 3-bromo-5-(methylthio)benzamide (**S8**) (601 mg, 93%) as a pale yellow solid. FT-IR (neat) ν_{max} 3382, 3126, 3038, 2804, 1655, 1609, 1580, 1554, 1442, 1380, 1255, 1160, 1095 cm^{-1}; ^{1}H NMR (400 MHz, DMSO-d_6) δ 7.69 (br s, 1H), 7.33 (app. t, *J* = 1.6 Hz, 1H), 7.26 (app. t, *J* = 1.6 Hz, 1H), 7.12 (app. t, *J* = 1.6 Hz, 1H), 7.09 (br s, 1H), 2.09 (s, 3H); ^{13}C NMR (101 MHz, DMSO-d_6) δ 165.9, 141.5, 136.6, 130.0, 126.5, 123.6, 122.2, 14.5; HRMS (ESI) Calcd. for $C_8H_9{}^{79}BrNOS$ [M + H]$^+$, 245.9583., found 245.9584.

4-(Benzylthio)-3-bromobenzamide (S9)

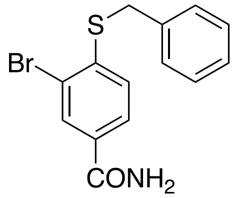

Oxidation of Thioether: To a stirred solution of 3-bromo-4-(methylthio)benzamide (**7d**) (500 mg, 2.04 mmol) in anhydrous dichloromethane (30 mL) was added *m*-CPBA (2.50 g, 10.2 mmol). The resulting solution was stirred at room temperature for 18

hours. The reaction was quenched by the addition of 2 M aq. solution of sodium hydroxide. The phases were separated, and the aqueous phase was extracted with dichloromethane (2 × 100 mL). The combined organic extracts were washed with water, dried ($MgSO_4$), filtered and concentrated *in vacuo* which afforded 3-bromo-4-(methylsulfonyl)benzamide which was used immediately in the next step without purification.

Nucleophilic Aromatic Substitution with Benzyl Mercaptan: To a stirred solution of 3-bromo-4-(methylsulfonyl)benzamide (60.0 mg, 220 µL) in toluene/isopropanol (10 mL, 1:1) was added benzyl mercaptan (380 µL, 3.25 mmol). The resulting solution was stirred under reflux for 3 hours. The mixture cooled to room temperature and concentrated *in vacuo*. Purification by flash column chromatography (petroleum ether/ ethyl acetate, 1:1) afforded 4-(benzylthio)-3-bromobenzamide (**S9**) (21.2 mg, 30%) as a colorless solid. ^{1}H NMR (400 MHz, DMSO-d_6) δ 8.08 (d, J = 1.9 Hz, 1H), 7.84 (dd, J = 8.3, 1.9 Hz, 1H), 7.53–7.42 (m, 4H), 7.39–7.25 (m, 4H), 4.37 (s, 2H); ^{13}C NMR (101 MHz, DMSO-d_6) δ 165.9, 142.0, 135.9, 131.9, 131.3, 129.0, 128.6, 127.4, 127.1, 126.1, 120.0, 35.6.

3-Bromo-4-methylbenzamide (S10)[4]

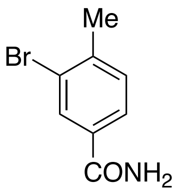

The reaction was performed as described in general procedure 2 using 3-bromo-4-methylbenzoic acid (1.00 g, 4.65 mmol) which afforded 3-bromo-4-methylbenzamide (**S10**) (960 mg, 97%) as a colorless solid. Spectroscopic data were consistent with the literature.[4] ^{1}H NMR (500 MHz, $CDCl_3$) δ 7.99 (d, J = 1.8 Hz, 1H), 7.64 (dd, J = 7.8, 1.9 Hz, 1H), 7.31 (d, J = 7.9 Hz, 1H), 5.89 (br s, 2H), 2.45 (s, 3H); LCMS (ESI) m/z: [M + H]$^+$ Calcd. for $C_8H_9{}^{79}BrNO$ 213.9; Found 214.1 at 5.62 mins.

3-Bromo-4-ethylbenzamide (S11)

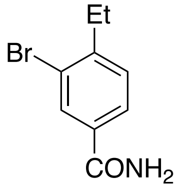

The reaction was performed as described in general procedure 2 using 3-bromo-4-ethylbenzoic acid (500 mg, 2.18 mmol) which afforded 3-bromo-4-ethylbenzamide (**S11**) (417 mg, 84%) as a colorless solid. ^{1}H NMR (400 MHz, $CDCl_3$) δ 7.99 (d, *J* = 1.9 Hz, 1H), 7.68 (dd, *J* = 7.9, 1.9 Hz, 1H), 7.31 (d, *J* = 7.9 Hz, 1H), 5.86 (s, 2H), 2.80 (q, *J* = 7.6 Hz, 2H), 1.25 (t, *J* = 7.5 Hz, 3H); ^{13}C NMR (101 MHz, $CDCl_3$) δ 167.9, 147.8, 132.7, 131.9, 129.7, 126.6, 124.7, 29.6, 14.0; LCMS (ESI) m/z: $[M + H]^+$ Calcd. for $C_9H_{11}{}^{79}BrNO$ 228.0; Found 228.1 at 2.94 mins.

3-Bromo-4-fluorobenzamide (S12)

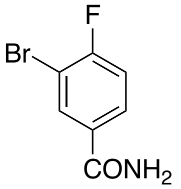

The reaction was performed as described in general procedure 2 using 3-bromo-4-fluorobenzoic acid (440 mg, 2.02 mmol) which afforded 3-bromo-4-fluorobenzamide (**S12**) (440 mg, quant.) as a colorless solid. ^{1}H NMR (400 MHz, $CDCl_3$) δ 8.06 (dd, *J* = 6.5, 2.2 Hz, 1H), 7.75 (ddd, *J* = 8.5, 4.6, 2.2 Hz, 1H), 7.23–7.14 (m, 1H), 5.88 (br s, 2H); ^{13}C NMR (101 MHz, $CDCl_3$) δ 167.0, 161.5 (d, $J_{C\text{-}F}$ = 253.7 Hz), 133.4, 131.0 (d, $J_{C\text{-}F}$ = 3.7 Hz), 128.5 (d, $J_{C\text{-}F}$ = 8.1 Hz), 116.8 (d, $J_{C\text{-}F}$ = 22.9 Hz), 109.8 (d, $J_{C\text{-}F}$ = 21.5 Hz); ^{19}F NMR (376 MHz, $CDCl_3$) δ −101.4 (ddd, *J* = 8.1, 6.5, 4.5 Hz); LCMS (ESI) m/z: $[M – H]^+$ Calcd. for $C_7H_4{}^{79}BrFNO$ 216.0; Found 216.1 at 6.19 mins.

3-Bromo-4-(trifluoromethyl)benzamide (S13)

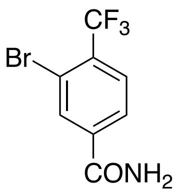

The reaction was performed as described in general procedure 2 using 3-bromo-4-(trifluoromethyl)benzoic acid (200 mg, 0.740 mmol) which afforded 3-bromo-4-(trifluoromethyl)benzamide (**S13**) (198 mg, quant.) as a colorless solid. ^{1}H NMR (400

MHz, $CDCl_3$) δ 8.15 (dt, *J* = 1.5, 0.7 Hz, 1H), 7.87–7.71 (m, 2H), 5.95 (br s, 2H); ^{13}C NMR (101 MHz, $CDCl_3$) δ 165.8, 137.3, 133.4, 132.7 (d, $J_{C\text{-}F}$ = 31.6 Hz), 127.7 (q, $J_{C\text{-}F}$ = 5.1 Hz), 125.5, 123.3, 120.4 (d, $J_{C\text{-}F}$ = 45.5 Hz); ^{19}F NMR (376 MHz, $CDCl_3$) δ −63.0 (s); LCMS (ESI) m/z: $[M – H]^+$ Calcd. for $C_8H_4{}^{79}BrF_3NO$ 265.9; Found 266.1 at 1.17 mins.

3-Bromo-4-((2-(trimethylsilyl)ethoxy)methoxy)benzamide (S14)

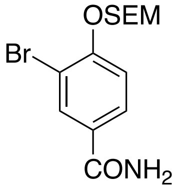

SEM Protection of Phenol: To a stirred solution of methyl 3-bromo-4-hydroxybenzoate (500 mg, 2.16 mmol) in anhydrous dichloromethane (10 mL) was added *N*,*N*-diisopropylethylmine (750 µL, 4.32 mmol). The resulting solution was stirred at room temperature for 30 mins, then 2-(trimethylsilyl)ethoxymethyl chloride (460 µL, 2.60 mmol) was subsequently added and stirred for a further 5 hours. The reaction mixture was quenched with 1 M hydrochloric acid and the aqueous phase was extracted with dichloromethane (3 × 30 mL). The combined organic extracts were washed with water (50 mL), dried ($MgSO_4$), filtered and concentrated *in vacuo* to afford methyl 3-bromo-4-((2-(trimethylsilyl)ethoxy)methoxy)benzoate which was used immediately in the next step.

Ester Hydrolysis and Amidation: The reaction was performed as described in general procedure **1** using methyl 3-bromo-4-((2-(trimethylsilyl)ethoxy)methoxy)benzoate (920 mg, 2.56 mmol) which afforded 3-bromo-4-((2-(trimethylsilyl)ethoxy)methoxy)benzoic acid (498 mg, 56%) as a colorless solid which was used immediately in the next step. Following general procedure 2 using 3-bromo-4-((2-(trimethylsilyl)ethoxy)methoxy)benzoic acid (490 mg, 1.42 mmol) afforded 3-bromo-4-((2-(trimethylsilyl)ethoxy)methoxy)benzamide (**S14**) (410 mg, 1.42 mmol, quant.) as a colorless solid. ^1H NMR (400 MHz, $CDCl_3$) δ 8.11–7.97 (m, 1H), 7.72 (dd, *J* = 8.6, 2.3 Hz, 1H), 7.21 (d, *J* = 8.6 Hz, 1H), 5.86 (s, 2H), 3.85–3.70 (m, 2H), 2.17 (s, 2H), 0.98–0.88 (m, 2H), -0.00 (s, 9H); ^{13}C NMR (101 MHz, $CDCl_3$) δ 167.7, 157.1, 133.0, 128.1, 127.8, 115.2, 112.9, 93.5, 67.2, 18.2, -1.3; LCMS (EI) m/z: $[M + H]^+$ Calcd. for $C_{13}H_{21}{}^{79}BrNO_3Si$ 346.0; Found 346.1 at 8.40 mins.

3-Bromo-4-methoxybenzamide (S15)

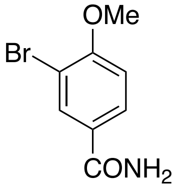

The reaction was performed as described in general procedure 4 using methyl 3-bromo-4-hydroxybenzoate (1.00 g, 4.33 mmol) and methyl iodide (380 µL, 6.06 mmol) which afforded methyl 3-bromo-4-methoxybenzoate which was used as crude without further purification. The hydrolysis reaction was performed according to general procedure 1 using methyl 3-bromo-4-methoxybenzoate (500 mg, 2.04 mmol) which afforded 3-bromo-4-methoxybenzoic acid (420 mg, 89%) which was used immediately in the next step. The amidation reaction was performed as described in general procedure 2 using 3-bromo-4-methoxybenzoic acid 400 mg, 1.73 mmol) which afforded 3-bromo-4-methoxybenzamide (**S15**) (373 mg, 94%) as a colorless solid. ^{1}H NMR (400 MHz, $CDCl_3$) δ 8.02 (d, *J* = 2.2 Hz, 1H), 7.78 (dd, *J* = 8.6, 2.2 Hz, 1H), 6.94 (d, *J* = 8.6 Hz, 1H), 5.74 (s, 2H), 3.96 (s, 3H); ^{13}C NMR (101 MHz, $CDCl_3$) δ 167.5, 159.0, 132.9, 128.5, 111.9, 111.5, 56.6; LCMS (ESI) m/z: $[M + H]^+$ Calcd. for $C_8H_9BrNO_2$ 229.9; Found 230.1 at 6.14 mins.

4-(Benzyloxy)-3-bromobenzamide (S16)

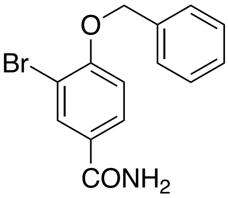

The reaction was performed as described in general procedure 4 using methyl 3-bromo-4-hydroxybenzoate (1.00 g, 4.33 mmol) and benzyl bromide (620 µL, 5.20 mmol) which afforded methyl 4-(benzyloxy)-3-bromobenzoate which was used as crude without further purification. The hydrolysis reaction was performed as described in general procedure 1 using methyl 4-(benzyloxy)-3-bromobenzoate (1.24 g, 3.86 mmol) which afforded 4-(benzyloxy)-3-bromobenzoic acid (1.08 g, 3.51 mmol, 91%) as a colorless solid which was used immediately in the next step. The amidation reaction was performed according to general procedure 2 using 4-(benzyloxy)-3-bromobenzoic acid (522 mg, 1.70 mmol) which afforded 4-(benzyloxy)-3-bromobenzamide (**S16**) (520 mg, quant.) as a colorless solid. ^{1}H NMR (400 MHz,

DMSO-d_6) δ 8.12 (d, J = 2.2 Hz, 1H), 7.94 (s, 1H), 7.88 (dd, J = 8.6, 2.2 Hz, 1H), 7.52–7.46 (m, 2H), 7.45–7.39 (m, 2H), 7.37–7.30 (m, 2H), 7.27 (d, J = 8.7 Hz, 1H), 5.28 (s, 2H); ^{13}C NMR (101 MHz, DMSO-d_6) δ 166.1, 156.6, 136.3, 132.3, 128.6, 128.5, 128.0, 128.0, 127.4, 113.3, 110.8, 70.2; LCMS (ESI) m/z: $[M + H]^+$ Calcd. for $C_{14}H_{13}{}^{79}BrNO_2$ 306.0; Found 306.0 at 5.12 mins.

Methyl 3-bromo-4-(cyclopropylmethoxy)benzoate (S17)

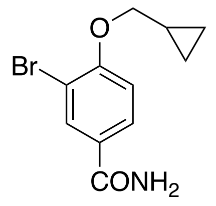

The reaction was performed as described in general procedure 4 using methyl 3-bromo-4-hydroxybenzoate (500 mg, 2.16 mmol) and (bromomethyl)cyclopropane (600 µL, 6.49 mmol) which afforded methyl 3-bromo-4-(cyclopropylmethoxy)benzoate which was used as crude without further purification. The hydrolysis reaction was performed according to general procedure 1 using methyl 3-bromo-4-(cyclopropylmethoxy)benzoate (513 mg, 1.81 mmol) which afforded 3-bromo-4-(cyclopropylmethoxy)benzoic acid (478 mg, 97%) as a colorless solid which was used immediately in the next step. The amidation reaction was performed according to general procedure 2 using 3-bromo-4-(cyclopropylmethoxy)benzoic acid (450 mg, 1.67 mmol) which afforded 3-bromo-4-(cyclopropylmethoxy)benzamide (**S17**) (451 mg, quant.) as a colorless solid. ^{1}H NMR (400 MHz, $CDCl_3$) δ 8.01 (d, J = 2.2 Hz, 1H), 7.74 (dd, J = 8.6, 2.2 Hz, 1H), 6.89 (d, J = 8.6 Hz, 1H), 5.73 (s, 2H), 3.96 (d, J = 6.7 Hz, 2H), 1.41–1.27 (m, 1H), 0.74–0.58 (m, 2H), 0.49–0.36 (m, 2H); ^{13}C NMR (101 MHz, $CDCl_3$) δ 167.6, 158.6, 132.9, 128.4, 126.7, 112.8, 112.5, 74.0, 10.1, 3.4, 3.4; LCMS (ESI) m/z: $[M – H]^+$ Calcd. for $C_{11}H_{11}{}^{79}BrNO_2$ 268.0; Found 268.1 at 0.998 mins.

3-Bromo-4-isobutoxybenzamide (S18)

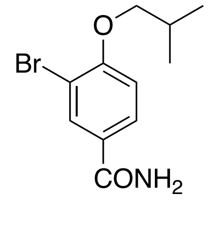

The reaction was performed as described in general procedure 4 using methyl 3-bromo-4-hydroxybenzoate (250 mg, 1.08 mmol) and 1-bromo-2-methylpropane (350

µL, 3.25 mmol) which afford methyl 3-bromo-4-isobutoxybenzoate which was used as crude without further purification. The hydrolysis reaction was performed according to general procedure 1 using methyl 3-bromo-4-isobutoxybenzoate (309 mg, 1.08 mmol) which afforded 3-bromo-4-isobutoxybenzoic acid (283 mg, 96%) as a colorless solid which was used immediately in the next step without further purification. The amidation reaction was performed according to general procedure 2 using 3-bromo-4-isobutoxybenzoic acid (283 mg, 1.04 mmol) which afforded 3-bromo-4-isobutoxybenzamide (**S18**) (271 mg, 96%) as a colorless solid. ^{1}H NMR (400 MHz, $CDCl_3$) δ 8.01 (d, *J* = 2.2 Hz, 1H), 7.75 (dd, *J* = 8.6, 2.2 Hz, 1H), 6.89 (d, *J* = 8.6 Hz, 1H), 5.71 (s, 2H), 3.84 (d, *J* = 6.4 Hz, 2H), 2.31–1.95 (m, 1H), 1.08 (d, *J* = 6.7 Hz, 6H); ^{13}C NMR (101 MHz, $CDCl_3$) δ 167.6, 158.6, 132.8, 128.4, 126.5, 112.4, 112.3, 75.7, 28.4, 19.3.

4-(Benzylamino)-3-bromobenzamide (S19)

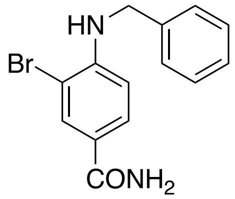

The reaction was performed as described in general procedure **5** using 3-bromo-4-fluorobenzamide (**S12**) (100 mg, 460 µmol) and benzylamine (250 µL, 2.30 mmol). Purification by flash column chromatography (petroleum ether/ethyl acetate 4:1) afforded 4-(benzylamino)-3-bromobenzamide (**S19**) (67.4 mg, 48%) as a colorless solid. ^{1}H NMR (400 MHz, $CDCl_3$) δ 7.98 (d, *J* = 2.1 Hz, 1H), 7.58 (ddd, *J* = 8.5, 2.1, 0.6 Hz, 1H), 7.40–7.28 (m, 5H), 6.59 (d, *J* = 8.6 Hz, 1H), 5.87–5.36 (m, 2H), 5.16 (br s, 1H), 4.46 (d, *J* = 5.6 Hz, 2H); ^{13}C NMR (101 MHz, $CDCl_3$) δ 168.0, 147.7, 137.8, 132.4, 129.0, 128.3, 127.8, 127.3, 122.5, 110.5, 109.2, 47.9; LCMS (ESI) m/z: [M + H]$^+$ Calcd. for $C_{14}H_{14}{}^{79}BrN_2O$ 305.1; Found 305.2 at 9.07 mins.

3-Bromo-4-morpholinobenzamide (S20)

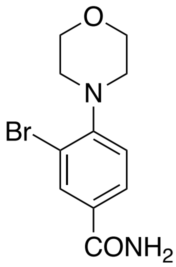

The reaction was performed as described in general procedure 5 using 3-bromo-4-fluorobenzamide (**S12**) (100 mg, 0.459 mmol) and morpholine (48.0 µL, 0.550 mmol). Purification by flash column chromatography (petroleum ether/ethyl acetate, 1:1 to 3:7) afforded 3-bromo-4-morpholinobenzamide (**S20**) (0.0501 g, 38%) as a colorless solid. ^{1}H NMR (400 MHz, $CDCl_3$) δ 8.04 (d, *J* = 2.1 Hz, 1H), 7.73 (dd, *J* = 8.3, 2.1 Hz, 1H), 7.05 (d, *J* = 8.4 Hz, 1H), 5.88 (s, 2H), 3.98–3.79 (m, 4H), 3.12 (dd, *J* = 5.4, 3.8 Hz, 4H); ^{13}C NMR (101 MHz, $CDCl_3$) δ 167.7, 153.7, 133.6, 129.1, 127.8, 120.4, 119.1, 67.1 (2 × CH_2), 51.8 (2 × CH_2); LCMS (ESI) m/z: $[M + H]^+$ Calcd. for $C_{11}H_{14}{}^{79}BrN_2O_2$ 285.1; Found 285.1 at 6.18 mins.

3-Bromo-4-((2-morpholinoethyl)amino)benzamide (S21)

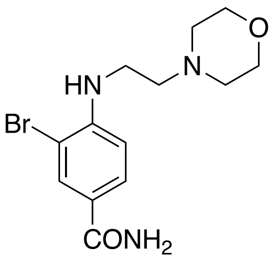

The reaction was performed as described in general procedure 5 using 3-bromo-4-fluorobenzamide (**S12**) (100 mg, 0.459 mmol) and 4-(2-aminoethyl)morpholine (72.0 µL, 0.550 mmol). Purification by flash column chromatography (ethyl acetate/methanol, 9:1) afforded 3-bromo-4-((2-morpholinoethyl)amino)benzamide (**S21**) (80.4 mg, 53%) as a colorless solid. ^{1}H NMR (500 MHz, $CDCl_3$) δ 7.94 (d, *J* = 2.0 Hz, 1H), 7.65 (dd, *J* = 8.5, 2.0 Hz, 1H), 6.58 (d, *J* = 8.5 Hz, 1H), 5.57–5.40 (m, 3H), 3.89–3.61 (m, 4H), 3.25 (q, *J* = 5.5 Hz, 2H), 2.71 (t, *J* = 6.0 Hz, 2H), 2.51 (s, 4H); ^{13}C NMR (126 MHz, $CDCl_3$) δ 168.1, 148.2, 132.3, 128.5, 121.9, 110.3, 109.2, 67.3 (2 × CH_2), 56.2, 53.2 (2 × CH_2), 39.7; LCMS (ESI) m/z: $[M + H]^+$ Calcd. for $C_{13}H_{19}{}^{79}BrN_3O_2$ 328.1; Found 328.2 at 5.77 mins.

3-Bromo-4-((2-(piperidin-1-yl)ethyl)amino)benzamide (S22)

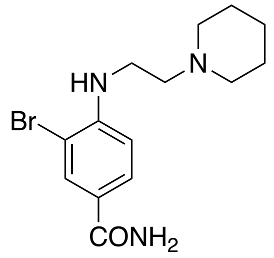

The reaction was performed as described in general procedure 5 using 3-bromo-4-fluorobenzamide (**S12**) (100 mg, 0.459 mmol) and 1-(2-aminoethyl)piperidine (78.0 µL, 0.550 mmol). Purification by flash column chromatography (ethyl acetate/triethylamine, 99:1) afforded 3-bromo-4-((2-(piperidin-1-yl)ethyl)amino)benzamide (**S22**) (78.9 mg, 53%) as an off-white solid. ^{1}H NMR (400 MHz, $CDCl_3$) δ 7.93 (d, *J* = 2.1 Hz, 1H), 7.64 (dd, *J* = 8.5, 2.1 Hz, 1H), 6.57 (d, *J* = 8.5 Hz, 1H), 5.80–5.40 (m, 3H), 3.28–3.18 (m, 2H), 2.65 (t, *J* = 6.1 Hz, 2H), 2.43 (br s, 2H), 1.66–1.42 (m, 8H); ^{13}C NMR (101 MHz, $CDCl_3$) δ 168.1, 148.4, 139.6, 132.2, 128.4, 110.2, 109.2, 56.4 (2 × CH_2), 54.3 (2 × CH_2), 40.1, 26.3, 24.6; LCMS (ESI) m/z: $[M + H]^+$ Calcd. for $C_{14}H_{21}{}^{79}BrN_3O$ 326.1; Found 326.2 at 5.08 mins.

3-Bromo-4-((3′-morpholinopropyl)amino)benzamide (S23)

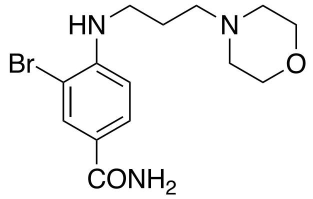

The reaction was performed as described in general procedure 5 using 3-bromo-4-fluorobenzamide (**S12**) (100 mg, 0.459 mmol) and 3-morpholinopropylamine (80.0 µL, 0.550 mmol). Purification by flash column chromatography (ethyl acetate/methanol 1:0 to 9:1), afforded 3-bromo-4-((3-morpholinopropyl)amino)benzamide (**S23**) (104 mg, 66%) as an off-white solid. ^{1}H NMR (400 MHz, $CDCl_3$) δ 7.94 (d, *J* = 2.1 Hz, 1H), 7.64 (dd, *J* = 8.6, 2.1 Hz, 1H), 6.60 (d, *J* = 8.6 Hz, 1H), 5.85–5.40 (m, 3H), 3.96–3.64 (m, 4H), 3.42–3.19 (m, 2H), 2.57–2.42 (m, 6H), 1.87 (p, *J* = 6.2 Hz, 2H); ^{13}C NMR (101 MHz, $CDCl_3$) δ 168.0, 148.3, 132.2, 128.3, 121.5, 109.7, 108.8, 66.8, 57.8, 54.0, 43.4, 24.6; LCMS (ESI) m/z: $[M + H]^+$ Calcd. for $C_{14}H_{21}{}^{79}BrN_3O_2$ 342.1; Found 342.1 at 5.47 mins.

3-Bromo-4-((2-(dimethylamino)ethyl)amino)benzamide(S24)

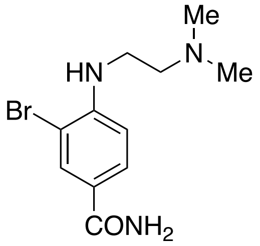

The reaction was performed as described in general procedure 5 using 3-bromo-4-fluorobenzamide (**S12**) (200 mg, 0.920 mmol) and *N*,*N*-methylethylenediamine (150 µL, 1.38 mmol). Purification by flash column chromatography (dichloromethane/methanol, 1:0 to 20:1) afforded 3-bromo-4-((2-(dimethylamino)ethyl)amino)benzamide (**S24**) (70.2 mg, 27%) as a colorless solid. ^{1}H NMR (400 MHz, $CDCl_3$) δ 7.93 (d, *J* = 2.1 Hz, 1H), 7.64 (dd, *J* = 8.5, 2.1 Hz, 1H), 6.58 (d, *J* = 8.6 Hz, 1H), 5.63 (s, 2H), 5.39 (br s, 1H), 3.23 (td, *J* = 6.2, 4.8 Hz, 2H), 2.61 (t, *J* = 6.1 Hz, 2H), 2.29 (s, 6H); ^{13}C NMR (101 MHz, $CDCl_3$) δ 168.1, 148.2, 132.3, 128.4, 121.8, 110.1, 109.1, 57.5, 45.3, 41.0; LCMS (EI) m/z: $[M + H]^+$ Calcd. for $C_{11}H_{17}{}^{79}BrN_3O$ 286.1; Found 286.1 at 4.37 mins.

6. Synthesis of Final Compounds: Experimental Procedures and Spectroscopic Data

General Procedure 6: Suzuki Cross-Coupling under Thermal Conditions 1

To a microwave vial was added aryl bromide (1.00 equiv.), aryl boronic acid or boronate ester (1.50 equiv.), XPhos-Pd-G2 (5 mol%), and potassium phosphate (1.75 equiv.), followed by 9:1 toluene:water (75.0 mM). The vial was capped and the mixture was stirred at 90 °C for 16 hours. The mixture was filtered over celite and concentrated *in vacuo*. The residue was then purified by flash column chromatography which afforded the coupled product.

General Procedure 7: Suzuki Cross-Coupling under Thermal Conditions 2

A microwave vial was charged with aryl bromide (1.00 equiv.), boronic acid (1.50 equiv.), tetrakis(triphenylphosphine)palladium(0) (10 mol%) and potassium phosphate tribasic (2.50 equiv.) followed by 9:1 *N*,*N'*-dimethylformamide (1.8 mL) and water (0.2 mL). The reaction mixture was stirred at 90 °C in a sand bath for 16 h. The reaction mixture was diluted with ethyl acetate (10 mL) and washed with saturated aqueous sodium bicarbonate (10 mL). The layers were separated, and the aqueous layer was extracted with ethyl acetate (3 × 20 mL). The combined organic extracts were washed with water (5 × 50 mL), brine (2 × 50 mL), dried ($MgSO_4$), filtered and concentrated *in vacuo*. The resulting residue was then purified by flash column chromatography and triturated or recrystallized which afforded the coupled product.

General Procedure 8: Suzuki Cross-Coupling under Microwave Conditions

A microwave vial was charged with aryl bromide (1 equiv.), boronic acid (1.25 equiv.), tetrakis(triphenylphosphine)palladium(0) (7.5 mol%) and cesium carbonate (2.50 equiv.) followed by 9:1 1,4-dioxane (1.8 mL) and water (0.2 mL). The reaction mixture was heated under microwave irradiation at 120 °C for 20 minutes. The reaction mixture was diluted with ethyl acetate (10 mL) and washed with saturated aqueous sodium bicarbonate (10 mL). The layers were separated, and the aqueous layer was extracted with ethyl acetate (3 × 20 mL). The combined organic extracts were washed with brine

(100 mL), dried ($MgSO_4$), filtered and concentrated *in vacuo*. The resulting residue was then purified by flash column chromatography, eluting with 50% ethyl acetate in petroleum ether (40–60) 100% ethyl acetate, and triturated or recrystallized which afforded the coupled product.

6-(Methylthio)-[1,1'-biphenyl]-3-carboxamide (8)

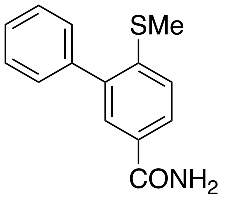

The reaction was performed as described in general procedure 6 using 3-bromo-4-(methylthio)benzamide (**7d**) (36.9 mg, 0.150 mmol), phenylboronic acid (27.4 mg, 0.225 mmol), XPhos-Pd-G2 (5.9 mg, 7.50 µmol), and potassium phosphate (55.8 mg, 0.263 mmol) in 9:1 toluene:water (2 mL). Purification by flash column chromatography (petroleum ether/ethyl acetate, 1:1) afforded 6-(methylthio)-[1,1'-biphenyl]-3-carboxamide (**8**) (30.4 mg, 83%) as a white solid. FT-IR (neat) ν_{max} 3369, 3292, 3185, 2920, 1655, 1611, 1551, 1404, 1383, 1269, 1162, 1103, 1076, 1037 cm^{-1}; ^{1}H NMR (500 MHz, DMSO-d_6) δ 7.97 (br s, 1H), 7.88 (dd, *J* = 8.3, 2.0 Hz, 1H), 7.70 (d, *J* = 2.0 Hz, 1H), 7.46 (app. t, *J* = 7.8 Hz, 2H), 7.43–7.36 (m, 4H), 7.30 (br s, 1H), 2.42 (s, 3H); ^{13}C NMR (126 MHz, DMSO-d_6) δ 167.3, 141.0, 139.5, 139.0, 130.2, 129.0, 128.5, 128.2, 127.7, 127.2, 124.0, 14.7; HRMS (ESI) m/z: $[M + H]^+$ Calcd. for $C_{14}H_{14}NOS$ 244.0791; Found 244.0791.

4-(Methylthio)-3-(thiophen-2-yl)benzamide (**9**)

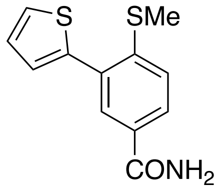

The reaction was performed as described in general procedure 6 using 3-bromo-4-(methylthio)benzamide (**7d**) (36.9 mg, 0.150 mmol), 2-thiopheneboronic acid pinacol ester (47.3 mg, 0.225 mmol), XPhos-Pd-G2 (5.9 mg, 7.50 µmol), and potassium phosphate tribasic (55.8 mg, 0.263 mmol) in 9:1 toluene/water (2 mL). Purification by flash column chromatography (petroleum ether/ethyl acetate, 1:1) afforded 4-(methylthio)-3-(thiophen-2-yl)benzamide (**9**) (34.8 mg, 93%) as an off-white solid. FT-

IR (neat) ν_{max} 3353, 3178, 2920, 1653, 1611, 1550, 1402, 1281, 1240, 1159, 1107, 1062 cm^{-1}; ^1H NMR (400 MHz, DMSO-d_6) δ 8.02 (br s, 1H), 7.90–7.84 (m, 2H), 7.66 (dd, *J* = 5.1, 1.2 Hz, 1H), 7.39 (d, *J* = 9.0 Hz, 1H), 7.35 (br s, 1H), 7.31 (dd, *J* = 3.5, 1.2 Hz, 1H), 7.17 (dd, *J* = 5.1, 3.5 Hz, 1H), 2.48 (s, 3H); ^{13}C NMR (101 MHz, DMSO-d_6) δ 167.0, 141.7, 139.9, 131.3, 130.2, 129.3, 127.9, 127.5, 127.4, 126.9, 124.4, 14.8; HRMS (ESI) m/z: $[M + H]^+$ Calcd. for $C_{12}H_{12}NOS_2$ 250.0355; Found 250.0348.

3-(3,5-dimethylisoxazol-4-yl)-4-(methylthio)benzamide (10)

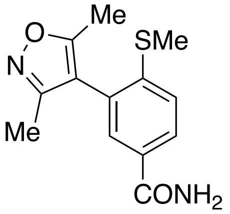

The reaction was performed as described in general procedure 6 using 3-bromo-4-(methylthio)benzamide (**7d**) (36.9 mg, 0.150 mmol), 3,5- dimethylisoxazole-4-boronic acid pinacol ester (50.2 mg, 0.225 mmol), XPhos-Pd-G2 (5.9 mg, 7.50 μmol), and potassium phosphate (55.8 mg, 0.263 mmol), in 9:1 toluene:water (2 mL). Purification by flash column chromatography (petroleum ether/ethyl acetate 2:1 to1:1) afforded 3-(3,5-dimethylisoxazol-4-yl)-4-(methylthio)benzamide (**10**) (13.6 mg, 17%) as a yellow soild. FT-IR (neat) ν_{max} 3392, 3178, 2928, 1683, 1629, 1595, 1556, 1379, 1301, 1286, 1264, 1232, 1160, 1085, 1029 cm^{-1}; ^1H NMR (400 MHz, DMSO-d_6) δ 7.97–7.90 (m, 2H), 7.66 (d, *J* = 2.0 Hz, 1H), 7.40 (d, *J* = 8.4 Hz, 1H), 7.35 (br s, 1H), 2.46 (s, 3H), 2.22 (s, 3H), 2.04 (s, 3H); ^{13}C NMR (101 MHz, DMSO-d_6) δ 167.0, 166.2, 158.7, 143.7, 130.2, 129.7, 128.3, 126.5, 123.8, 114.2, 14.2, 11.3, 10.1; HRMS (ESI) m/z: $[M + H]^+$ Calcd. for $C_{13}H_{15}N_2O_2S$ 263.0849; Found 263.0844.

***tert*-Butyl 2-(5-carbamoyl-2-(methylthio)phenyl)-1*H*-pyrrole-1-carboxylate (S25)**

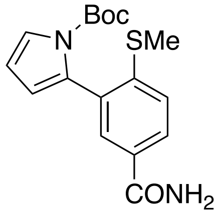

The reaction was performed as described in general procedure 6 using 3-bromo-4-(methylthio)benzamide (**7d**) (36.9 mg, 0.150 mmol), *N*-boc pyrrole- 2-boronic acid (47.5 mg, 0.225 mmol), XPhos-Pd-G2 (5.9 mg, 7.50 μmol), and potassium phosphate

tribasic (55.8 mg, 0.263 mmol) in 9:1 toluene/water (2 mL). The crude residue was resubjected to the above conditions. Purification by flash column chromatography (petroleum ether/ethyl acetate, 1:2) afforded *tert*-butyl 2-(5-carbamoyl-2-(methylthio)phenyl)-1*H*-pyrrole-1-carboxylate (**S25**) (35.1 mg, 70%) as a pale brown solid. FT-IR (neat) ν_{max} 3392, 3200, 2984, 1733, 1644, 1612, 1571, 1549, 1398, 1342, 1312, 1143, 1161, 1087, 1067 cm^{-1}; ^{1}H NMR (500 MHz, acetone-d_6) δ 7.95 (dd, J = 8.3, 2.0 Hz, 1H), 7.75 (d, J = 2.0 Hz, 1H), 7.48 (br s, 1H), 7.40 (dd, J = 3.3, 1.8 Hz, 1H), 7.35 (d, J = 8.3 Hz, 1H), 6.63 (br s, 1H,), 6.29 (app. t, J = 3.3 Hz, 1H,), 6.16 (dd, J = 3.3, 1.8 Hz, 1H), 2.44 (s, 3H), 1.22 (s, 9H); ^{13}C NMR (126 MHz, acetone-d_6) δ 168.3, 149.7, 145.7, 134.0, 132.1, 130.8, 130.0, 128.5, 124.3, 122.5, 115.4, 111.4, 83.8, 27.6, 14.8; HRMS (ESI) m/z: $[M + Na]^+$ Calcd. for $C_{17}H_{20}N_2O_3SNa$ 355.1087; Found 355.1078.

4-(Methylthio)-3-(1*H*-pyrrol-2-yl)benzamide (**11**)

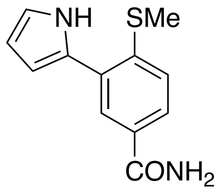

A solution of *tert*-butyl 2-(5-carbamoyl-2-(methylthio)phenyl)-1*H*-pyrrole-1-carboxylate (**S25**) (78.3 mg, 0.218 mmol) in freshly prepared sodium methoxide solution (5 mL, 50.0 mM) was stirred at room temperature for 16 hours. The mixture was diluted with ethyl acetate and water, and the organic phase separated. The aqueous phase was extracted with ethyl acetate and the combined organic phase was dried (Na_2SO_4) and concentrated *in vacuo*. Purification by flash column chromatography (petroleum ether/ethyl acetate, 2:1 to 1:2) afforded 4-(methylthio)-3-(1*H*-pyrrol-2-yl)benzamide (**11**) (24.8 mg, 45%) as a dark yellow solid. FT-IR (neat) ν_{max} 3252, 3180, 1677, 1618, 1549, 1396, 1290, 1081, 1024 cm^{-1}; ^{1}H NMR (400 MHz, DMSO-d_6) δ 11.10 (br s, 1H), 7.89 (br s, 1H), 7.87 (d, J = 2.0 Hz, 1H), 7.74 (dd, J = 8.3, 2.0 Hz, 1H), 7.31 (d, J = 8.3 Hz, 1H), 7.30 (br s, 1H), 6.88–6.85 (m, 1H), 6.47–6.45 (m, 1H), 6.17–6.12 (m, 1H), 2.46 (s, 3H); ^{13}C NMR (101 MHz, DMSO-d_6) δ 167.6, 140.1, 130.8, 130.0, 128.3, 127.3, 125.7, 124.0, 118.9, 109.5, 108.2, 14.8; HRMS (ESI) m/z: $[M + H]^+$ Calcd. for $C_{12}H_{13}N_2OS$ 233.0743; Found 233.0738.

3-(1*H*-Indazol-5-yl)-4-(methylthio)benzamide (12)

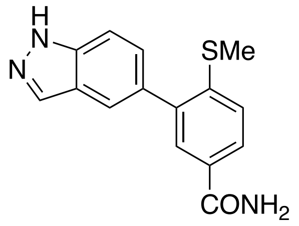

The reaction was performed as described in general procedure 8 using 3-bromo-4-(methylthio)benzamide (**7d**) (50.0 mg, 0.204 mmol), 1-*H*-indazole-5-boronic acid (62.0 mg, 0.295 mmol), tetrakis(triphenylphosphine)palladium(0) (17.0 mg, 0.0150 mmol) and cesium carbonate (108 mg, 0.570 mmol) in 9:1 dioxane/water (2 mL). The reaction mixture was stirred at 120 °C under microwave irradiation for 1.5 hours. Purification by flash column chromatography (ethyl acetate), followed by trituration from diethyl ether afforded 3-(1*H*-indazole-5-yl)-4-(methylthio)benzamide (**12**) (9.80 mg, 17%) as a white solid. FT-IR (neat) v_{max} 3283, 3171, 1676, 1595, 1419, 1379, 1254, 1072, 949 cm^{-1}; ^{1}H NMR (500 MHz, $CDCl_3$) δ 8.26 (s, 1H), 7.82 (dd, *J* = 8.3, 2.1 Hz, 1H), 7.68–7.64 (m, 2H), 7.46 (d, *J* = 8.3 Hz, 1H), 7.30 (d, *J* = 8.3 Hz, 1H), 7.24 (dd, *J* = 8.4, 1.7 Hz, 1H), 6.60 (t, *J* = 2.7 Hz, 1H), 6.01 (br s, 1H), 5.54 (br s, 1H), 2.42 (s, 3H); ^{13}C NMR (126 MHz, $CDCl_3$) δ 169.0, 143.7, 141.7, 135.6, 131.3, 129.0, 128.9, 128.0, 126.7, 125.1, 124.0, 123.6, 121.6, 110.9, 103.3, 15.7; HRMS (ESI) m/z: $[M + H]^+$ Calcd. for $C_{15}H_{14}N_3OS$ 284.0852, found 284.0853.

3-(1*H*-Indol-5-yl)-4-(methylthio)benzamide (13)

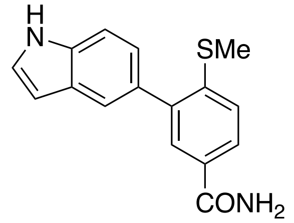

The reaction was performed as described in general procedure 8 using 3-bromo-4-(methylthio)benzamide (**7d**) (100 mg, 0.408 mmol), (1*H*-indol-5-yl)boronic acid (82.0 mg, 0.510 mmol), tetrakis(triphenylphosphine)palladium(0) (35.0 mg, 31.0 μmol, 7.5 mol%) and cesium carbonate (217 mg, 1.02 mmol). Purification by flash column chromatography (petroleum ether/ethyl acetate, 1:1 to 0:1) followed by trituration from diethyl ether afforded 3-(1*H*-indol-5-yl)-4-(methylthio)benzamide (**13**) (48.0 mg, 42%) as a white solid. v_{max}/cm^{-1} 3390, 3268 (NH_2), 1667 (C=O), 1599, 1560, 1552, 1412, 1282, 1073, 754 cm^{-1}; ^{1}H NMR (500 MHz, DMSO-d_6) δ 11.17 (s, 1H), 7.95 (br s, 1H),

7.84 (dd, *J* = 8.3, 1.9 Hz, 1H), 7.73 (d, *J* = 1.9 Hz, 1H), 7.54 (br s, 1H), 7.44 (d, *J* = 8.3 Hz, 1H), 7.39 (t, *J* = 2.6 Hz, 1H), 7.34 (d, *J* = 8.3 Hz, 1H), 7.25 (br s, 1H), 7.10 (dd, *J* = 8.3, 1.5 Hz, 1H), 6.46 (br s, 1H), 2.40 (s, 3H); ^{13}C NMR (126 MHz, DMSO-d_6) δ 167.5, 141.4, 140.5, 135.3, 130.2, 129.9, 128.9, 127.5, 126.4, 126.0, 123.6, 122.3, 120.5, 111.0, 101.3, 14.7; HRMS (ESI) m/z: [M + H]$^+$ Calcd. for $C_{16}H_{15}N_2OS$ 283.0900, found 283.0897.

3-(1*H*-Indazol-6-yl)-4-(methylthio)benzamide (14)

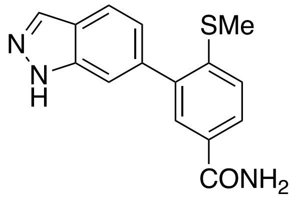

The reaction was performed as described in general procedure 8 using 3-bromo-4-(methylthio)benzamide (**7d**) (107 mg, 0.435 mmol), (1*H*-indol-6-yl)boronic acid (88.0 mg, 0.543 mmol), tetrakis(triphenylphosphine)palladium(0) (38.0 mg, 0.0329 mmol, 7.5 mol%) and cesium carbonate (354 mg, 1.09 mmol). Trituration from methanol afforded 3-(1*H*-indazol-6-yl)-4-(methylthio)benzamide (**14**) (12.2 mg, 10%) as a white solid. ^{1}H NMR (500 MHz, DMSO-d_6) δ 13.08 (s, 2H), 8.13 (d, *J* = 1.1 Hz, 1H), 7.94 (dd, *J* = 8.3, 2.0 Hz, 1H), 7.83 (d, *J* = 8.3 Hz, 1H), 7.73 (d, *J* = 1.9 Hz, 1H), 7.55–7.48 (m, 1H), 7.45 (d, *J* = 8.3 Hz, 1H), 7.11 (dd, *J* = 8.3, 1.4 Hz, 1H), 2.45 (s, 3H), one H resonance not observed; ^{13}C NMR (101 MHz, DMSO) δ 176.6, 153.1, 149.3, 149.0, 146.4, 142.9, 139.7, 138.3, 136.2, 133.6, 131.8, 131.3, 130.0, 119.8, 24.1; HRMS (ESI) m/z: [M + H]$^+$ Calcd. for $C_{15}H_{14}N_3OS$ 284.0852, found 284.0853.

3-(1*H*-Benzo[*d*]imidazol-5-yl)-4-(methylthio)benzamide (15)

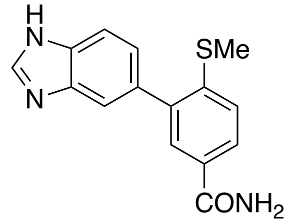

The reaction was performed as described in general procedure 8 using 3-bromo-4-(methylthio)benzamide (**7d**) (110 mg, 0.450 mmol), 1*H*-benzimidazole-6-boronic acid pinacol ester (136 mg, 0.560 mmol), tetrakis(triphenylphosphine)palladium(0) (39.0 mg, 34.0 μmol, 7.5 mol%) and cesium carbonate (364 mg, 1.11 mmol) in 9:1

dioxane/water (2 mL). Purification by preparative reverse phase HPLC (water (0.1% TFA)/acetonitrile (0.1% TFA); 5–95%) afforded 3-(1*H*-benzo[*d*]imidazol-5-yl)-4-(methylthio)benzamide (**15**) (12.0 mg, 9.4%) as a colorless solid. ^{1}H NMR (400 MHz, CD_3OD) δ 9.31 (s, 1H), 7.93 (dd, *J* = 8.5, 2.1 Hz, 1H), 7.89 (d, *J* = 8.5 Hz, 1H), 7.85 (s, 1H), 7.77 (d, *J* = 2.1 Hz, 1H), 7.64 (dd, *J* = 8.4, 1.4 Hz, 1H), 7.45 (d, *J* = 8.4 Hz, 1H), 2.46 (s, 3H); ^{13}C NMR (101 MHz, CD_3OD) δ 171.6, 144.3, 141.8, 140.1, 139.7, 132.7, 132.2, 131.0, 130.3, 129.3, 128.8, 125.4, 116.2, 115.3, 15.3; HRMS (ESI) m/z: $[M + H]^+$ Calcd. for $C_{15}H_{14}N_3OS$ 284.0852, found 284.0846.

3-(1-Methyl-1*H*-indazol-5-yl)-4-(methylthio)benzamide (16)

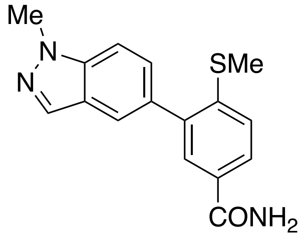

The reaction was performed as described in general procedure 8 using 3-bromo-4-(methylthio)benzamide (**7d**) (50.0 mg, 0.203 mmol), (1-methyl-1*H*-indazol-5-yl)boronic acid (45.0 mg, 0.254 mmol), tetrakis(triphenylphosphine)palladium(0) (18.0 mg, 15.2 μmol, 7.5 mol%) and cesium carbonate (165 mg, 0.508 mmol). The residue was triturated with acetone (×2) and methanol which afforded 3-(1-methyl-1*H*-indazol-5-yl)-4-(methylthio)benzamide (**16**) (44.2 mg, 73%) as a white solid. FT-IR (neat) v_{max} 3353, 3147, 2926, 2784, 1682, 1627, 1553, 1510, 1384, 1284, 1269, 1219, 1173, 1160, 1109, 1067, 1028 cm^{-1}; ^{1}H NMR (400 MHz, DMSO-d_6) δ 8.09 (d, *J* = 1.3 Hz, 1H), 7.98 (br s, 1H), 7.89 (dd, *J* = 8.3, 1.3 Hz, 1H), 7.77–7.74 (m, 2H), 7.70 (d, *J* = 8.7 Hz, 1H), 7.42 (dd, *J* = 8.7, 1.6 Hz, 1H), 7.39 (d, *J* = 8.3 Hz, 1H), 7.30 (br s, 1H), 4.09 (s, 3H), 2.42 (s, 3H); ^{13}C NMR (101 MHz, DMSO-d_6) δ 167.4, 141.4, 139.3, 139.0, 132.7, 131.7, 130.1, 128.9, 127.6, 127.0, 123.9, 123.4, 121.1, 109.4, 35.4, 14.7; HRMS (ESI) m/z: $[M - H]^+$ Calcd. for $C_{16}H_{14}N_3OS$ 296.0863, found 296.0862.

3-(1-Methyl-1*H*-indol-5-yl)-4-(methylthio)benzamide (17)

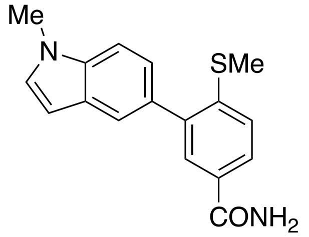

The reaction was performed as described in general procedure 6 using 3-bromo-4-(methylthio)benzamide (**7d**) (36.9 mg, 0.150 mmol), *N*-methylindole-5-boronic acid (39.4 mg, 0.225 mmol), XPhos-Pd-G2 (5.90 mg, 7.50 μmol), and potassium phosphate tribasic (55.8 mg, 0.263 mmol) in 9:1 toluene:water (2 mL). Purification by flash column chromatography (petroleum ether/ethyl acetate, 2:3) afforded 3-(1-methyl-1*H*-indol-5-yl)-4-(methylthio)benzamide (**17**) (41.0 mg, 92%) as a brown solid. FT-IR (neat) v_{max} 3412, 3338, 3280, 3129, 2920, 1666, 1612, 1550, 1511, 1380, 1331, 1267, 1244, 1147, 1101, 1067, 1028 cm^{-1}; ^{1}H NMR (400 MHz, DMSO-*d*$_6$) δ 8.00 (br s, 1H), 7.85 (dd, *J* = 8.4, 2.0 Hz, 1H), 7.74 (d, *J* = 2.0 Hz, 1H), 7.55 (d, *J* = 1.4 Hz, 1H,), 7.49 (d, *J* = 8.4 Hz, 1H), 7.38 (d, *J* = 3.0 Hz, 1H), 7.35 (d, *J* = 8.4 Hz, 1H), 7.32 (br s, 1H), 7.17 (dd, *J* = 8.4, 1.4 Hz, 1H), 6.46 (d, *J* = 3.0 Hz, 1H), 3.83 (s, 3H), 2.40 (s, 3H); ^{13}C NMR (101 MHz, DMSO-*d*$_6$) δ 167.6, 141.6, 140.4, 135.9, 130.4, 130.0, 129.0, 127.9, 126.6, 123.7, 122.5, 120.9, 109.5, 100.7, 32.6, 14.8, One quaternary C resonance not observed; HRMS (ESI) m/z: [M + H]$^+$ Calcd. for $C_{17}H_{17}N_2OS$ 297.1056, found 297.1053.

3-(Imidazo[1,2-*a*]pyridin-6-yl)-4-(methylthio)benzamide (18)

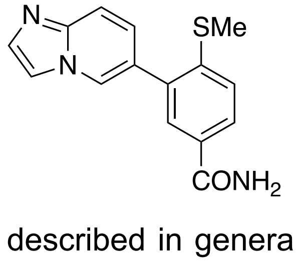

The reaction was performed as described in general procedure 6 using 3-bromo-4-(methylthio)benzamide (**7d**) (50.0 mg, 0.203 mmol), 6-imidazo[1,2-a]pyridine boronic acid pinacol ester (62.0 mg, 0.254 mmol), tetrakis(triphenylphosphine)palladium(0) (17.6 mg, 0.0150 mmol, 7.5 mol%) and cesium carbonate (0.165 g, 0.508 mmol). Purification by flash column chromatography (ethyl acetate/methanol, 4:1) afforded 3-(imidazo[1,2-*a*]pyridin-6-yl)-4-(methylthio)benzamide (**18**) (12.0 mg, 21%) as a light brown solid. FT-IR (neat) v_{max} 3337, 3171, 1672, 1624, 1377, 1312, 1128, 721 cm $^{-1}$;

^{1}H NMR (500 MHz, $CDCl_3$) δ 8.13 (s, 1H), 7.85 (dd, *J* = 8.3 Hz, 2.0 Hz, 1H), 7.72 (d, *J* = 2.0 Hz, 1H), 7.67 (d, *J* = 1.0 Hz, 1H), 7.62 (d, *J* = 9.3 Hz, 1H), 7.59 (s, 1H), 7.31 (d, *J* = 8.4 Hz, 1H), 7.20 (dd, *J* = 9.3, 1.7 Hz, 1H), 6.46 (br s, 1H), 5.70 (br s, 1H), 2.46 (s, 3H); ^{13}C NMR (126 MHz, $CDCl_3$) δ 168.5, 144.8, 143.9, 135.8, 134.3, 129.6, 129.3, 127.9, 126.8, 125.5, 124.8, 124.3, 117.3, 113.0, 15.5; HRMS (ESI) m/z: $[M + H]^+$ Calcd. for $C_{15}H_{14}N_3OS$ 284.0852, found 284.0840.

4-(Methylthio)-3-(2-oxoindolin-4-yl)benzamide (19)

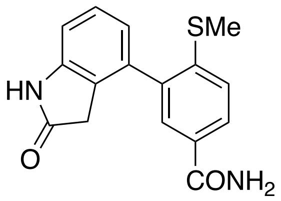

The reaction was performed as described in general procedure 6 using 3-bromo-4-(methylthio)benzamide (**7d**) (36.9 mg, 0.150 mmol), oxindole-4-boronic acid pinacol ester (24.8 mg, 0.188 mmol), tetrakis(triphenylphosphine)palladium(0) (13.1 mg, 11.3 μmol, 7.5 mol%) and cesium carbonate (122 mg, 0.375 mmol) in 10:1 dioxane/water (3 mL). Purification by flash column chromatography (petroleum ether/ethyl acetate, 0:1) followed by trituration in hexane afforded 4-(methylthio)-3-(2-oxoindolin-4-yl)benzamide (**19**) (6.50 mg, 15%) as a red solid. FT-IR (neat) v_{max} 3178, 1696, 1618, 1552, 1404, 1387, 1322, 1290, 1229, 1106, 1055 cm $^{-1}$; ^{1}H NMR (400 MHz, DMSO-*d*$_6$) δ 10.49 (br s, 1H), 7.94 (br s, 1H), 7.90 (dd, *J* = 8.4, 2.0 Hz, 1H), 7.68 (d, *J* = 2.0 Hz, 1H), 7.38 (d, *J* = 8.4 Hz, 1H), 7.30 (br s, 1H), 7.26 (app. t, *J* = 7.8 Hz, 1H), 6.86 (d, *J* = 7.8 Hz, 1H), 6.84 (d, *J* = 7.8 Hz, 1H), 2.43 (s, 3H), 2.08 (s, 2H); ^{13}C NMR (101 MHz, DMSO-*d*$_6$) δ 175.9, 167.2, 143.8, 141.1, 136.8, 135.9, 130.0, 127.8, 127.7, 127.6, 124.4, 123.9, 122.3, 108.7, 35.3, 14.3; HRMS (ESI) m/z: $[M-H]^+$ Calcd. for $C_{16}H_{13}N_2O_2S$ 297.0703, found 297.0702.

4-(Methylthio)-3-(quinolin-6-yl)benzamide (20)

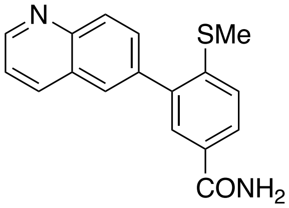

The reaction was performed as described in general procedure 6 using 3-bromo-4-(methylthio)benzamide (**7d**) (50.0 mg, 0.203 mmol), 6-quinolineboronic acid pinacol ester (102 mg, 0.400 mmol), XPhos-Pd-G2 (8.00 mg, 10.0 μmol), and potassium phosphate tribasic (72.0 mg, 0.340 mmol) in 9:1 toluene/water (2 mL). Purification by flash column chromatography (petroleum ether/ethyl acetate, 1:1 to 0:1 to ethyl acetate/methanol, 9:1) afforded 4-(methylthio)-3-(quinolin-6-yl)benzamide (**20**) (52.0 mg, 89%) as a colorless solid. FT-IR (neat) v_{max} 3344, 3191, 2925, 2858, 1659, 1621, 1599, 1554, 1502, 1415, 1379, 1076, 841 cm^{-1}; ^{1}H NMR (500 MHz, $CDCl_3$) δ 8.96 (dd, *J* = 4.3, 1.8 Hz, 1H), 8.23–8.14 (m, 2H), 7.87–7.82 (m, 2H), 7.80 (dd, *J* = 8.6, 2.0 Hz, 1H), 7.73 (d, *J* = 2.1 Hz, 1H), 7.45 (dd, *J* = 8.2, 4.3 Hz, 1H), 7.35 (d, *J* = 8.3 Hz, 1H), 6.07 (s, 1H), 5.61 (s, 1H), 2.45 (s, 3H); ^{13}C NMR (126 MHz, $CDCl_3$) δ 168.6, 150.9, 143.3, 139.7, 138.0, 136.5, 131.2, 129.5, 129.1, 128.4, 128.3, 127.4, 124.4, 121.7, 15.7; HRMS (ESI) m/z: $[M + H]^+$ Calcd. for $C_{17}H_{15}N_2OS$ 295.0900; Found 295.0902.

3-(Isoquinolin-6-yl)-4-(methylthio)benzamide (21)

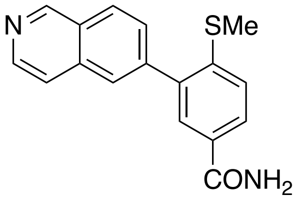

The reaction was performed as described in general procedure 6 using 3-bromo-4-(methylthio)benzamide (**7d**) (50.0 mg, 0.203 mmol), isoquinoline-6-boronic acid (69.0 mg, 0.400 mmol), XPhos-Pd-G2 (8.00 mg, 10.0 μmol), and potassium phosphate tribasic (72.0 mg, 0.340 mmol) in 9:1 toluene/water (2 mL). Purification by flash column chromatography (petroleum ether/ethyl acetate, 1:1 to 0:1 to ethyl acetate/methanol, 9:1) afforded 3-(isoquinolin-6-yl)-4-(methylthio)benzamide (**21**) (33.0 mg, 56%) as a colorless solid. FT-IR (neat) v_{max} 3347, 3170, 2927, 1670, 1631, 1599, 1556, 1385, 1275, 1074, 957, 895, 834 cm^{-1}; ^{1}H NMR (400 MHz, DMSO-d_6) δ 9.37 (s, 1H), 8.55 (d, *J* = 5.7 Hz, 1H), 8.20 (d, *J* = 8.4 Hz, 1H), 8.00 (d, *J* = 1.6 Hz, 2H), 7.95 (dd, *J* = 8.3,

2.0 Hz, 1H), 7.88 (d, *J* = 5.7 Hz, 1H), 7.83 (d, *J* = 2.0 Hz, 1H), 7.73 (dd, *J* = 8.4, 1.7 Hz, 1H), 7.46 (d, *J* = 8.4 Hz, 1H), 7.35 (s, 1H), 2.46 (s, 3H); ^{13}C NMR (101 MHz, DMSO-d_6) δ 167.1, 152.2, 143.3, 141.6, 141.1, 138.3, 135.1, 135.1, 130.3, 128.9, 128.7, 127.7, 127.5, 126.6, 124.3, 120.4, 14.7; HRMS (ESI) m/z: [M + H]$^+$ Calcd. for $C_{17}H_{15}N_2OS$ 295.0905; Found 295.0900.

4-(Methylthio)-3-(pyridin-3-yl)benzamide (22)

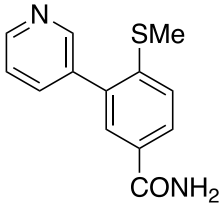

The reaction was performed as described in general procedure 6 using 3-bromo-4-(methylthio)benzamide (**7d**) (50.0 mg, 0.203 mmol), 3-pyridinylboronic acid (29.0 mg, 0.240 mmol), XPhos-Pd-G2 (8.00 mg, 10.0 μmol), and potassium phosphate tribasic (72.0 mg, 0.340 mmol) in 9:1 toluene/water (2 mL). Purification by flash column chromatography (petroleum ether/ethyl acetate, 1:1 to 0:1) afforded 4-(methylthio)-3-(pyridin-3-yl)benzamide (**22**) (18.3 mg, 37%) as a colorless solid. FT-IR (neat) v_{max} 3381, 3183, 1646, 1621, 1545, 1405, 1258, 1117, 1031 cm^{-1}; ^{1}H NMR (500 MHz, $CDCl_3$) δ 8.68–8.63 (m, 2H), 7.83 (dd, *J* = 8.3, 2.1 Hz, 1H), 7.76 (dt, *J* = 7.9, 2.0 Hz, 1H), 7.63 (d, *J* = 2.1 Hz, 1H), 7.41–7.35 (m, 1H), 7.33 (d, *J* = 8.4 Hz, 1H), 5.99 (s, 1H), 5.59 (s, 1H), 2.45 (s, 3H); ^{13}C NMR (126 MHz, $CDCl_3$) δ 168.4, 150.1, 149.4, 143.6, 136.9, 136.9, 135.3, 129.6, 129.0, 127.7, 124.6, 123.2, 15.6; HRMS (ESI) m/z: [M + H]$^+$ Calcd. for $C_{13}H_{13}N_2OS$ 245.0743; Found 245.0747.

4-(Methylthio)-3-(pyridin-4-yl)benzamide (23)

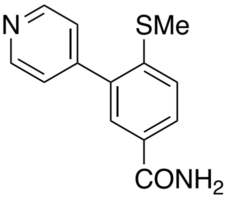

The reaction was performed as described in general procedure 6 using 3-bromo-4-(methylthio)benzamide (**7d**) (50.0 mg, 0.203 mmol), 4-pyridinylboronic acid (49.0 mg, 0.400 mmol), XPhos-Pd-G2 (8.00 mg, 10.0 μmol), and potassium phosphate tribasic (72.0 mg, 0.340 mmol) in 9:1 toluene/water (2 mL). Purification by flash column chromatography (petroleum ether/ethyl acetate, 1:1 to 0:1) afforded 4-(methylthio)-3-

(pyridin-4-yl)benzamide (**23**) (26.9 mg, 55%) as a colorless solid. FT-IR (neat) ν_{max} 3310, 3170, 2921, 2854, 1681, 1629, 1595, 1554, 1385, 1085, 1072, 828, 724 cm^{-1}; ^{1}H NMR (500 MHz, $CDCl_3$) δ 8.71 (s, 2H), 7.82 (dd, J = 8.3, 2.1 Hz, 1H), 7.64 (d, J = 2.1 Hz, 1H), 7.42 (s, 2H), 7.34 (d, J = 8.4 Hz, 1H), 5.99 (s, 1H), 5.56 (s, 1H), 2.46 (s, 3H); ^{13}C NMR (126 MHz, $CDCl_3$) δ 168.2, 149.8, 142.8, 137.7, 129.7, 128.6, 127.9, 124.7, 124.4, 29.9, 15.6; HRMS (ESI) m/z: $[M + H]^+$ Calcd. for $C_{13}H_{13}N_2OS$ 245.0743; Found 245.0745.

4-(Methylthio)-3-(pyrimidin-5-yl)benzamide (24)

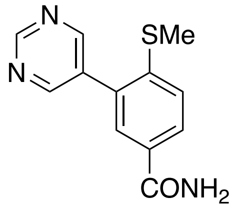

The reaction was performed as described in general procedure 6 using 3-bromo-4-(methylthio)benzamide (**7d**) (50.0 mg, 0.203 mmol), pyrimidine-5-boronic acid (49.6 mg, 0.400 mmol), XPhos-Pd-G2 (8.00 mg, 10.0 μmol), and potassium phosphate tribasic (72.0 mg, 0.340 mmol) in 9:1 toluene/water (2 mL). Purification by flash column chromatography using a gradient system (petroleum ether/ethyl acetate, 1:1 to 0:1 to ethyl acetate/methanol, 9:1) afforded 4-(methylthio)-3-(pyrimidin-5-yl)benzamide (**24**) (15.0 mg, 31%) as a colorless solid. FT-IR (neat) ν_{max} 3390, 3204, 3081, 3051, 2925, 1666, 1623, 1554, 1402, 1383, 1350, 1074, 1053, 908, 858, 832, 726 cm^{-1}; ^{1}H NMR (400 MHz, DMSO-d_6) δ 9.24 (s, 1H), 8.90 (s, 2H), 7.99 (s, 1H), 7.97 (dd, J = 8.3, 2.0 Hz, 1H), 7.81 (d, J = 2.0 Hz, 1H), 7.50 (d, J = 8.4 Hz, 1H), 7.39 (s, 1H), 2.49 (s, 3H); ^{13}C NMR (101 MHz, DMSO-d_6) δ 166.9, 157.6, 156.8, 141.5, 133.2, 132.3, 130.7, 129.0, 128.6, 125.0, 14.9; HRMS (ESI) m/z: $[M + H]^+$ Calcd. for $C_{12}H_{12}N_3OS$ 246.0696; Found 246.0700.

3-(Benzofuran-5′-yl)-4-(methylthio)benzamide (25)

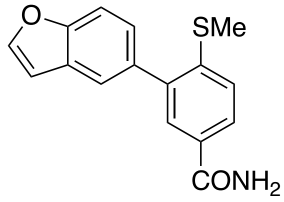

The reaction was performed as described in general procedure 8 using 3-bromo-4-(methylthio)benzamide (**7d**) (50.0 mg, 0.203 mmol), benzo[*b*]furan-5-boronic acid (41.3 mg, 0.255 mmol), tetrakis(triphenylphosphine)palladium(0) (18.0 mg, 0.0152 mmol, 7.5 mol%), and cesium carbonate (165 mg, 0.508 mmol) in 9:1 dioxane/water (2 mL). Purification by flash column chromatography (petroleum ether/ethyl acetate 4:1 to 0:1) followed by trituration in diethyl ether (×3) afforded 3-(benzofuran-5′-yl)-4-(methylthio)benzamide (**25**) (34.7 mg, 60%) as a beige solid. FT-IR (neat) ν_{max} 3150, 2918, 1665, 1611, 1381, 1098, 766, 739 cm $^{-1}$; ^{1}H NMR (500 MHz, $CDCl_3$) δ 7.81 (dd, *J* = 8.3, 2.1 Hz, 1H), 7.67 (d, *J* = 1.9 Hz, 1H), 7.65 (d, *J* = 2.1 Hz, 1H), 7.63–7.61 (m, 1H), 7.56 (d, *J* = 8.3 Hz, 1H), 7.33 (dd, *J* = 8.4, 1.9 Hz, 1H), 7.29 (d, *J* = 8.4 Hz, 1H), 6.80 (d, *J* = 2.1, 0.8 Hz, 1H), 6.05 (br s, 1H), 5.77 (br s, 1H), 2.42 (s, 3H); ^{13}C NMR (126 MHz, $CDCl_3$) δ 168.9, 154.8, 145.8, 143.5, 140.7, 134.3, 129.2, 129.0, 127.7, 127.0, 125.8, 124.0, 122.1, 111.3, 106.9, 15.6; HRMS (ESI) m/z: $[M + H]^+$ Calcd. for $C_{16}H_{14}NO_2S$ 284.0745, found 284.0727.

3-(Benzoxazole)-4-(methylthio)benzamide (26)

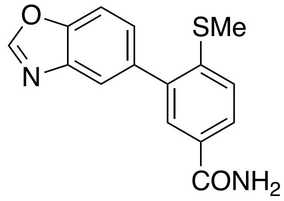

The reaction was performed as described in general procedure 8 using 3-bromo-4-(methylthio)benzamide (**7d**) (50.0 mg, 0.203 mmol), benzoxazole-5-boronic acid pinacol ester (62.2 mg, 0.254 mmol), tetrakis(triphenylphosphine)palladium(0) (18.0 mg, 0.0152 mmol, 7.5 mol%), and cesium carbonate (165 mg, 0.508 mmol) in 9:1 dioxane/water (2 mL). Purification by flash column chromatography (petroleum ether/ethyl acetate, 1:1 to 0:1) followed by trituration in diethyl ether (× 3) afforded 3-(benzoxazole)-4-(methylthio)benzamide (**26**) (45.5 mg, 79%) as a colorless solid. FT-IR (neat) ν_{max} 3183, 3107, 1692, 1622, 1518, 1402, 1385, 1061, 816, 667 cm $^{-1}$; ^{1}H NMR (500 MHz, $CDCl_3$) δ 8.15 (s, 1H), 7.85–7.81 (m, 2H), 7.66 (d, *J* = 2.1 Hz, 1H),

7.64 (d, J = 8.5 Hz, 1H), 7.43 (dd, J = 8.3, 1.9 Hz, 1H), 7.31 (d, J = 8.3 Hz, 1H), 6.03 (br s, 1H), 5.66 (br s, 1H), 2.43 (s, 3H); ^{13}C NMR (126 MHz, $CDCl_3$) δ 168.5, 153.2, 149.7, 143.3, 140.2, 139.8, 136.2, 129.2, 128.9, 127.1, 127.1, 124.1, 121.5, 110.8, 15.5; HRMS (ESI) m/z: $[M + H]^+$ Calcd. for $C_{15}H_{13}N_2O_2S$ 285.0697, found 285.0682.

3-(2,3-Dihydrobenzofuran-5-yl)-4-(methylthio)benzamide (27)

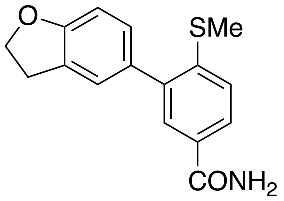

The reaction was performed as described in general procedure 8 using 3-bromo-4-(methylthio)benzamide (**7d**) (50.0 mg, 0.203 mmol), 2,3-dihydrobenzofuran-5-boronic acid (41.6 mg, 0.254 mmol), tetrakis(triphenylphosphine)palladium(0) (18.0 mg, 0.0152 mmol, 7.5 mol%), and cesium carbonate (165 mg, 0.508 mmol) in 9:1 dioxane/water (2 mL). Purification by flash column chromatography (petroleum ether/ethyl acetate, 1:1 to 0:1) followed by trituration in diethyl ether and then recrystallization from toluene afforded 3-(2,3-dihydrobenzofuran-5-yl)-4-(methylthio)benzamide (**27**) (34.9 mg, 60%) as a colorless solid. FT-IR (neat) ν_{max} 3408, 3179, 2974, 1649, 1605, 1551, 1493, 1398, 1377, 1227, 1109, 1065, 982, 912, 816, 770 cm^{-1}; ^{1}H NMR (500 MHz, $CDCl_3$) δ 7.77 (dd, J = 8.3, 2.1 Hz, 1H), 7.59 (d, J = 2.1 Hz, 1H), 7.26 (d, J = 8.3 Hz, 1H), 7.23 (d, J = 1.9 Hz, 1H), 7.14 (dd, J = 8.2, 1.9 Hz, 1H), 6.84 (d, J = 8.2 Hz, 1H), 5.93 (br s, 2H), 4.62 (t, J = 8.7 Hz, 2H), 3.26 (t, J = 8.7 Hz, 2H), 2.43 (s, 3H); ^{13}C NMR (126 MHz, $CDCl_3$) δ 169.0, 160.2, 143.4, 140.6, 131.8, 129.3, 129.1, 128.8, 127.3, 126.7, 126.0, 124.0, 109.2, 71.6, 29.8, 15.7; HRMS (ESI) m/z: $[M + H]^+$ Calcd. for $C_{16}H_{16}NO_2S$ 286.0896, found 286.0884.

3-(3′,4′-Dimethoxyphenyl)-4-(methylthio)benzamide (28)

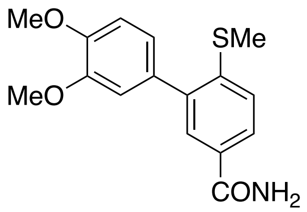

The reaction was performed as described in general procedure 8 using 3-bromo-4-(methylthio)benzamide (**7d**) (50.0 mg, 0.203 mmol), 3,4-dimethoxybenzene boronic acid (46.5 mg, 0.255 mmol), tetrakis(triphenylphosphine)palladium(0) (18.0 mg,

0.0152 mmol, 7.5 mol%), and cesium carbonate (165 mg, 0.508 mmol) in 9:1 dioxane/water (2 mL). Purification by flash column chromatography (petroleum ether/ethyl acetate 1:1 to 0:1) followed by trituration from diethyl ether (×3) and then chloroform (×3) afforded 3-(3′,4′-dimethoxyphenyl)-4-(methylthio)benzamide (**28**) (21.1 mg, 34%) as a colorless solid. FT-IR (neat) $ν_{max}$ 3437, 3173, 2974, 1672, 1616, 1518, 1460, 1381, 1260, 1238, 1211, 1146, 1018, 822, 766, 667 cm $^{-1}$; ^{1}H NMR (500 MHz, DMSO-d_6) δ 7.96 (br s, 1H), 7.85 (dd, J = 8.5, 2.0 Hz, 1H), 7.71 (d, J = 2.0 Hz, 1H), 7.36 (d, J = 8.5 Hz, 1H), 7.30 (br s, 1H), 7.04 (d, J = 8.2 Hz, 1H), 6.98 (d, J = 2.0 Hz, 1H), 6.96 (dd, J = 8.2, 2.0 Hz, 1H), 3.81 (s, 3H), 3.79 (s, 3H), 2.43 (s, 3H); ^{13}C NMR (126 MHz, DMSO-d_6) δ 167.9, 148.9, 148.9, 141.7, 139.4, 132.4, 130.5, 129.0, 127.3, 124.3, 121.9, 113.4, 112.0, 56.1 (2 × CH_3), 15.2; HRMS (ESI) m/z: Calcd. For $[M + H]^+$ $C_{16}H_{18}NO_3S$ 304.1002, found 304.0989.

3'-Fluoro-4'-methoxy-6-(methylthio)-[1,1'-biphenyl]-3-carboxamide (29)

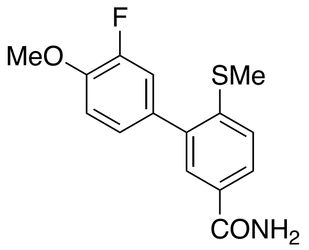

The reaction was performed as described in general procedure 6 using 3-bromo-4-(methylthio)benzamide (**7d**) (36.9 mg, 0.150 mmol), 3-fluoro-4-methoxyphenyl boronic acid (38.2 mg, 0.225 mmol), XPhos-Pd-G2 (5.90 mg, 7.50 µmol), and potassium phosphate tribasic (55.8 mg, 0.263 mmol) in 9:1 toluene/water (2 mL). Purification by flash column chromatography (petroleum ether/ethyl acetate, 1:1 to 1:2) followed by preparative reverse phase HPLC (water (0.1% TFA)/acetonitrile (0.1% TFA); 5–95%) afforded 3'-fluoro-4'-methoxy-6-(methylthio)-[1,1'-biphenyl]-3-carboxamide (**29**) (9.10 mg, 21%) as a white solid. FT-IR (neat) $ν_{max}$ 3440, 3170, 1683, 1618, 1517, 1435, 1394, 1305, 1269, 1234, 1134, 1076, 1020 cm $^{-1}$; ^{1}H NMR (400 MHz, DMSO-d_6) δ 7.97 (br s, 1H), 7.86 (dd, J = 8.3, 2.0 Hz, 1H), 7.70 (d, J = 2.0 Hz, 1H), 7.37 (d, J = 8.3 Hz, 1H), 7.30 (br s, 1H), 7.29–7.22 (m, 2H), 7.18 (dd, J = 8.4, 2.1 Hz, 1H), 3.89 (s, 3H), 2.43 (s, 3H); ^{13}C NMR (101 MHz, DMSO-d_6) δ 167.3, 151.0 (d, J = 244.0 Hz), 146.7 (d, J = 10.4 Hz), 141.1, 137.6, 132.1 (d, J = 7.0 Hz), 130.2, 128.6, 127.3, 125.6 (d, J = 2.7 Hz), 124.1, 116.7 (d, J = 18.5 Hz), 113.6, 56.1, 14.7; ^{19}F NMR (376 MHz,

DMSO-*d*6) δ −135.60 (dd, J = 12.4, 9.0 Hz); HRMS (ESI) m/z: $[M + H]^+$ Calcd. for $C_{15}H_{15}FNO_2S$ 292.0802, found 292.0794.

4'-Amino-3'-methoxy-6-(methylthio)-[1,1'-biphenyl]-3-carboxamide (**30**)

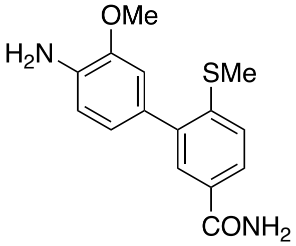

The reaction was performed as described in general procedure 8 using 3-bromo-4-(methylthio)benzamide (**7d**) (36.9 mg, 0.150 mmol), *tert*-butyl (2- methoxy-4-(4,4,5,5-tetramethyl-1,3,2-dioxaborolan-2-yl)phenyl)carbamate (65.5 mg, 0.188 mmol), tetrakis(triphenylphosphine)palladium(0) (13.1 mg, 11.3 µmol), and cesium carbonate (122 mg, 0.375 mmol) in 10:1 1,4-dioxane/water (3 mL). The residue was dissolved in 1:1 dichloromethane/trifluoroacetic acid (10 mL) and stirred at 40 °C for 16 hours then concentrated *in vacuo*. Purification by flash column chromatography (petroleum ether/ethyl acetate, 1:2 to 0:1] followed by preparative reverse phase HPLC (water (0.1% TFA)/acetonitrile (0.1% TFA); 5–95%) afforded 4'-amino-3'-methoxy-6-(methylthio)-[1,1'-biphenyl]-3-carboxamide (**30**) (21.3 mg, 49%) as an off-white solid. FT-IR (neat) v_{max} 3344, 3193, 2921, 2852, 1653, 1595, 1523, 1411, 1376, 1251, 1212, 1182, 1152, 1080, 1029 cm^{-1}; ^1H NMR (400 MHz, DMSO-d_6) δ 7.97 (br s, 1H), 7.85 (dd, J = 8.3, 2.0 Hz, 1H), 7.70 (d, J = 2.0 Hz, 1H), 7.36 (d, J = 8.3 Hz, 1H), 7.31 (br s, 1H), 7.08 (d, J = 8.0 Hz, 1H), 7.04 (d, J = 1.7 Hz, 1H), 6.92 (dd, J = 8.0, 1.7 Hz, 1H), 3.87 (s, 3H), 2.43 (s, 3H), aniline NH resonances not evident; ^{13}C NMR (101 MHz, DMSO-d_6) δ 167.4, 149.1, 141.1, 138.8, 134.5, 130.1, 128.5, 128.1, 126.9, 123.9, 121.7, 118.8, 112.5, 55.9, 14.8; HRMS (ESI) m/z: $[M + H]^+$ Calcd. for $C_{15}H_{17}N_2O_2S$ 289.1005, found 289.0997.

2'-Fluoro-3'-hydroxy-6-(methylthio)-[1,1'-biphenyl]-3-carboxamide (31)

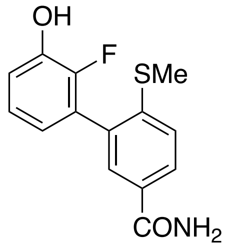

The reaction was performed as described in general procedure 8 using 3-bromo-4-(methylthio)benzamide (**7d**) (36.9 mg, 0.150 mmol), 2-fluoro-3- hydroxyphenylboronic acid (29.2 mg, 0.188 mmol), tetrakis(triphenylphosphine)palladium(0) (13.1 mg, 11.3 μmol), and cesium carbonate (122 mg, 0.375 mmol), in 10:1 1,4-dioxane/water (3 mL). Purification by flash column chromatography (petroleum ether/ethyl acetate, 1:2) followed by preparative reverse phase HPLC (water (0.1% TFA)/acetonitrile (0.1% TFA); 5–95%) afforded 2'-fluoro-3'-hydroxy-6-(methylthio)-[1,1'-biphenyl]-3-carboxamide (**32**) (16.7 mg, 40%) as a white solid. FT-IR (neat) ν_{max} 2923, 1653, 1597, 1552, 1472, 1418, 1374, 1286, 1202, 1115, 1053 cm^{-1}; ^{1}H NMR (400 MHz, DMSO-*d*6) δ 9.92 (s, 1H), 7.96 (br s, 1H), 7.92 (dd, *J* = 8.3, 2.0 Hz, 1H), 7.70 (d, *J* = 2.0 Hz, 1H), 7.39 (d, *J* = 8.3 Hz, 1H), 7.31 (br s, 1H), 7.07–6.97 (m, 2H), 6.69 (ddd, *J* = 7.5, 6.4, 1.8 Hz, 1H), 2.43 (s, 3H); ^{13}C NMR (101 MHz, DMSO-d_6) δ 167.1, 148.2 (d, *J* = 241.8 Hz), 145.2 (d, *J* = 12.5 Hz), 142.3, 133.2, 129.9, 128.8, 127.8, 127.7 (d, *J* = 16.6 Hz), 124.1 (d, *J* = 4.1 Hz), 124.0, 120.9, 117.6, 14.5; ^{19}F NMR (471 MHz, DMSO-d_6) δ −138.25 (app. t, *J* = 7.1 Hz); HRMS (ESI) m/z: $[M + H]^+$ Calcd. for $C_{14}H_{13}FNO_2S$ 278.0646, found 278.0636.

3'-Fluoro-5'-hydroxy-6-(methylthio)-[1,1'-biphenyl]-3-carboxamide (32)

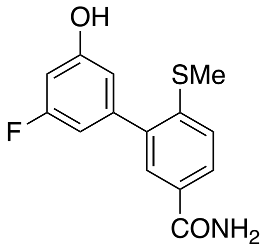

The reaction was performed as described in general procedure 8 using 3-bromo-4-(methylthio)benzamide (**7d**) (36.9 mg, 0.150 mmol), 5-fluoro-3- hydroxyphenylboronic acid (29.2 mg, 0.188 mmol), tetrakis(triphenylphosphine)palladium(0) (13.1 mg, 11.3 μmol), and cesium carbonate (122 mg, 0.375 mmol) in 10:1 1,4-dioxane/water (3 mL). Purification by flash column chromatography (petroleum ether/ethyl acetate, 1:2)

followed by trituration in diethyl ether afforded 3'-fluoro-5'-hydroxy-6-(methylthio)-[1,1'-biphenyl]-3-carboxamide (**32**) (17.1 mg, 41%) as a white solid. FT-IR (neat) ν_{max} 3403, 3219, 1677, 1595, 1551, 1426, 1385, 1338, 1266, 1215, 1132, 1053 cm^{-1}. ^{1}H NMR (400 MHz, acetone-d_6) δ 9.06 (s, 1H), 7.94 (dd, *J* = 8.3, 2.1 Hz, 1H), 7.75 (d, *J* = 2.1 Hz, 1H), 7.56 (br s, 1H), 7.40 (d, *J* = 8.3 Hz, 1H), 6.73 (t, *J* = 1.8 Hz, 1H), 6.68–6.61 (m, 3H), 2.46 (s, 3H); ^{13}C NMR (101 MHz, acetone-d_6) δ 168.5, 164.2 (d, *J* = 242.9 Hz), 159.7 (d, *J* = 12.1 Hz), 143.5 (d, *J* = 9.8 Hz), 142.6, 139.6, 131.2, 129.3, 128.5, 125.0, 113.5 (d, *J* = 2.1 Hz), 108.0 (d, *J* = 22.3 Hz), 102.8 (d, *J* = 24.2 Hz), 15.3; ^{19}F NMR (471 MHz, DMSO-d_6) δ −112.54 (app. t, *J* = 10.2 Hz); HRMS (ESI) m/z: $[M + H]^+$ Calcd. for $C_{14}H_{13}FNO_2S$ 278.0646, found 278.0637.

4'-Fluoro-3'-hydroxy-6-(methylthio)-[1,1'-biphenyl]-3-carboxamide (**33**)

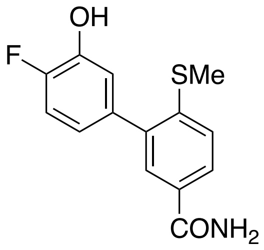

The reaction was performed as described in general procedure 8 using 3-bromo-4-(methylthio)benzamide (**7d**) (36.9 mg, 0.150 mmol), 4-fluoro-3- hydroxyphenylboronic acid (29.2 mg, 0.188 mmol), tetrakis(triphenylphosphine)palladium(0) (13.1 mg, 11.3 µmol), and cesium carbonate (122 mg, 0.375 mmol) in 10:1 dioxane/water (3 mL). Purification by flash column chromatography (petroleum ether/ethyl acetate, 1:2] followed by preparative reverse phase HPLC (water (0.1% TFA)/acetonitrile (0.1% TFA); 5–95%) afforded 4'-fluoro-3'-hydroxy-6-(methylthio)-[1,1'-biphenyl]-3-carboxamide (**33**) (19.4 mg, 47%) as a white solid. FT-IR (neat) ν_{max} 3185, 2921, 1655, 1595, 1552, 1510, 1420, 1385, 1310, 1264, 1242, 1191, 1113, 1076 cm^{-1}; ^{1}H NMR (400 MHz, DMSO-d_6) δ 9.99 (s, 1H), 7.97 (br s, 1H), 7.87 (dd, *J* = 8.3, 2.0 Hz, 1H), 7.68 (d, *J* = 2.0 Hz, 1H), 7.35 (d, *J* = 8.4 Hz, 1H), 7.30 (br s, 1H), 7.20 (dd, *J* = 11.4, 8.3 Hz, 1H), 6.97 (dd, *J* = 8.6, 2.2 Hz, 1H), 6.78 (ddd, *J* = 8.3, 4.3, 2.2 Hz, 1H), 2.43 (s, 3H); ^{13}C NMR (101 MHz, DMSO-d_6) δ 167.3, 150.7 (d, *J* = 241.8 Hz), 144.6 (d, *J* = 12.4 Hz), 141.1, 138.2, 136.0 (d, *J* = 3.8 Hz), 130.1, 128.4, 127.2, 123.9, 120.2 (d, *J* = 6.9 Hz), 118.5, 115.9 (d, *J* = 18.3 Hz), 14.7; ^{19}F NMR (471 MHz, acetone-d6) δ

−139.82– −139.89 (m); HRMS (ESI) m/z: $[M + H]^+$ Calcd. for $C_{14}H_{13}FNO_2S$ 278.0646, found 278.0637.

5-(1-Methyl-1*H*-indazol-5-yl)-6-(methylthio)nicotinamide (34)

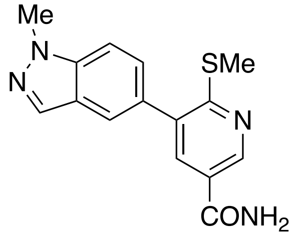

The reaction was performed as described in general procedure 7 using 5-bromo-6-(methylthio)nicotinamide (**S2**) (50.0 mg, 0.202 mmol), 1-methyl-1*H*-indazole-5-boronic acid (54.0 mg, 0.303 mmol), tetrakis(triphenylphosphine)palladium(0) (23.3 mg, 20.2 μmol), and potassium phosphate tribasic (107 mg, 0.505 mmol) in 9:1 *N*,*N*-dimethylformamide/water (2 mL). Purification by flash column chromatography (ethyl acetate to ethyl acetate/methanol, 9:1) followed by trituration in diethyl ether followed by toluene afforded 5-(1-methyl-1*H*-indazol-5-yl)-6-(methylthio)nicotinamide (**34**) (17.6 mg, 29%) as a colorless solid. FT-IR (neat) ν_{max} 3348, 3204, 1672, 1622, 1593, 1393, 1373, 1263, 1113, 1092, 826, 814, 752, 644 cm^{-1}; ^1H NMR (500 MHz, DMSO-d_6) δ 8.93 (d, *J* = 2.2 Hz, 1H), 8.12 (s, 1H), 8.10 (s, 1H), 7.99 (d, *J* = 2.2 Hz, 1H), 7.85 (d, *J* = 1.5 Hz, 1H), 7.75 (d, *J* = 8.6 Hz, 1H), 7.51 (s, 1H), 7.48 (dd, *J* = 8.6, 1.6 Hz, 1H), 4.10 (s, 3H), 2.49 (s, 3H); ^{13}C NMR (126 MHz, DMSO-d_6) *δ* 166.2, 160.6, 147.0, 139.2, 135.3, 134.9, 132.8, 129.2, 127.2, 125.3, 123.5, 121.4, 109.8, 35.5, 13.3; HRMS (ESI) m/z: $[M + H]^+$ Calcd. for $C_{15}H_{15}N_4OS$ 299.0961, found 299.0952.

6-(Benzylthio)-5-(1-methyl-1*H*-indazol-5-yl)nicotinamide (35)

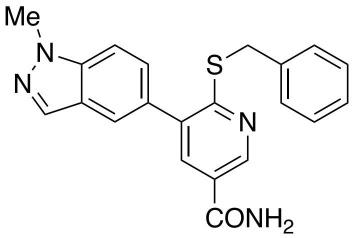

The reaction was performed as described in general procedure 8 using 6-(benzylthio)-5-bromonicotinamide (**S3**) (50.0 mg, 0.155 mmol), 1-methyl-1*H*-indazole-5-boronic acid (34.2 mg, 0.193 mmol), tetrakis(triphenylphosphine)palladium(0) (13.4 mg, 11.6 μmol, 7.5 mol%), and cesium carbonate (126 mg, 0.388 mmol) in 9:1 dioxane/water

(2 mL). Purification by flash column chromatography (petroleum ether/ethyl acetate, 1:1 to 0:1) followed by trituration in diethyl ether followed by recrystallization from toluene afforded 6-(benzylthio)-5-(1-methyl-1*H*-indazol-5-yl)nicotinamide (**35**) (40.6 mg, 70%) as a colorless solid. FT-IR (neat) ν_{max} 3410, 3348, 3210, 1659, 1624, 1369, 1090, 748, 708, 692 cm^{-1}; δ_H (500 MHz, $CDCl_3$) 4.09 (3H, s), 4.45 (2H, s), 5.83 (1H, br s, NH), 6.03 (1H, br s, NH), 7.18–7.23 (1H, dd, J = 8.5, 6.0 Hz), 7.24–7.29 (2H, m), 7.34–7.39 (2H, m), 7.41–7.47 (2H, m), 7.76 (1H, br s), 7.88 (1H, d, J = 2.2 Hz), 8.00 (1H, s), 8.87 (1H, d, J = 2.2 Hz); δ_C (126 MHz, $CDCl_3$) 35.3, 35.8, 109.3, 122.1, 124.2, 124.7, 127.3, 127.6, 128.6 (2 × CH), 129.4 (2 × CH), 129.5, 133.3, 135.72, 135.75, 137.6, 139.8, 146.4, 162.7, 167.5; HRMS (ESI) m/z: $[M + H]^+$ Calcd. for $C_{21}H_{19}N_4OS$ 375.1274, found 375.1262.

5-(1-Methyl-1*H*-indazol-5-yl)nicotinamide (36)

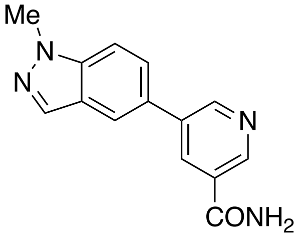

The reaction was performed as described in general procedure 8 using 5-bromonicotinamide (50.0 mg, 0.249 mmol), 1-methyl-1*H*-indazole-5-boronic acid (55.0 mg, 0.311 mmol), tetrakis(triphenylphosphine)palladium(0) (21.6 mg, 18.7 μmol, 7.5 mol%), and cesium carbonate (203 mg, 0.623 mmol) in 9:1 dioxane/water (2 mL). Purification by flash column chromatography (petroleum ether/ethyl acetate, 1:1 to 0:1) afforded 5-(1-methyl-1*H*-indazol-5-yl)nicotinamide (**36**) (47.9 mg, 76%) as an off-white solid. FT-IR (neat) ν_{max} 3358, 3167, 1651, 1524, 1439, 1396, 1221, 1111, 988, 893, 860; cm^{-1}; ^{1}H NMR (500 MHz, $CDCl_3$) δ 9.04 (d, J = 2.3 Hz, 1H), 8.95 (d, J = 2.1 Hz, 1H), 8.42 (t, J = 2.2 Hz, 1H), 8.07 (d, J = 0.9 Hz, 1H), 7.98 (t, J = 1.2 Hz, 1H), 7.66 (dd, J = 8.7, 1.7 Hz, 1H), 7.53 (d, J = 8.7 Hz, 1H), 6.15 (br s, 1H), 5.72 (br s, 1H), 4.13 (s, 3H); ^{13}C NMR (126 MHz, $CDCl_3$) δ 167.3, 151.5, 146.2, 134.2, 133.5, 129.5, 126.0, 124.9, 120.1, 110.1, 35.9; HRMS (ESI) m/z: $[M + H]^+$ Calcd. for $C_{14}H_{13}N_4O$ 253.1084, found 253.1075.

6-Methoxy-5-(1-methyl-1*H*-indazol-5-yl)nicotinamide (37)

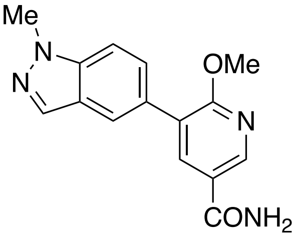

The reaction was performed as described in general procedure 8 using 5-bromo-6-methoxynicotinamide (**S4**) (50.0 mg, 0.216 mmol), 1-methyl-1*H*-indazole-5-boronic acid (47.6 mg, 0.271 mmol), tetrakis(triphenylphosphine)palladium(0) (18.7 mg, 16.2 μmol, 7.5 mol%), and cesium carbonate (176 mg, 0.540 mmol) in 9:1 dioxane/water (2 mL). Purification by flash column chromatography (petroleum ether/ethyl acetate, 1:1 to 0:1) followed by trituration in diethyl ether followed by recrystallization from toluene afforded 6-methoxy-5-(1-methyl-1*H*-indazol-5-yl)nicotinamide (**37**) (43.6 mg, 71%) as a colorless solid. FT-IR (neat) ν_{max} 3343, 3210, 2945, 1672, 1622, 1601, 1439, 1402, 1379, 1258, 1217, 1171, 1015, 781, 677 cm^{-1}; ^{1}H NMR δ (500 MHz, $CDCl_3$) δ 8.59 (d, J = 2.0 Hz, 1H), 8.13 (d, J = 2.0 Hz, 1H), 8.02 (br s, 1H), 7.91 (s, 1H), 7.60 (d, J = 8.6 Hz, 1H), 7.45 (d, J = 8.6 Hz, 1H), 5.89 (br s, 2H), 4.11 (s, 3H), 4.04 (s, 3H); ^{13}C NMR (126 MHz, $CDCl_3$) δ 167.5, 163.4, 145.1, 139.6, 138.3, 133.4, 128.2, 127.9, 125.1, 124.3, 123.1, 122.0, 108.9, 54.5, 35.8; HRMS (ESI) m/z: [M + H]$^+$ Calcd. for $C_{15}H_{15}N_4O_2$ 283.1190, found 283.1179.

3-Fluoro-5-(1-methyl-1*H*-indazol-5-yl)-4-(methylthio)benzamide (38)

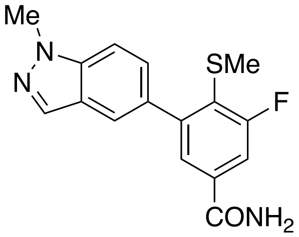

The reaction was performed as described in general procedure 8 using using 3-bromo-5-fluoro-4-(methylthio)benzamide (**S6**) (39.6 mg, 0.150 mmol), *N*-methylindazole-5-boronic acid (33.0 mg, 0.188 mmol), tetrakis(triphenylphosphine)palladium(0) (13.1 mg, 11.3 μmol), and cesium carbonate (122 mg, 0.375 mmol) in 10:1 dioxane/water (3 mL). Purification by flash column chromatography (petroleum ether/ethyl acetate/acetone, 1:2:0 to 0:0:1) followed by trituration in acetone afforded 3-fluoro-5-(1-methyl-1*H*-indazol-5-yl)-4-(methylthio)benzamide (**38**) (16.4 mg, 35%) as a white

solid. FT-IR (neat) v_{max} 3355, 3185, 3057, 2921, 2852, 1675, 1618, 1560, 1513, 1392, 1355, 1327, 1221, 1128, 1098, 1061 cm^{-1}; ^1H NMR (400 MHz, DMSO-d_6) δ 8.13 (br s, 1H), 8.11 (d, J = 0.9 Hz, 1H), 7.79–7.77 (m, 1H), 7.75–7.69 (m, 3H), 7.55 (br s, 1H), 7.46 (dd, J = 8.7, 1.6 Hz, 1H), 4.09 (s, 3H), 2.25 (d, J = 1.4 Hz, 3H); ^{13}C NMR (101 MHz, DMSO-d_6) δ 166.0, 162.0 (d, J = 243.9 Hz), 146.2, 139.0, 134.6 (d, J = 8.4 Hz), 132.8, 131.7, 127.9, 126.2 (d, J = 17.4 Hz), 125.3, 123.3, 121.4, 113.5 (d, J = 25.9 Hz), 109.1, 35.5, 17.8 (d, J = 7.0 Hz); ^{19}F NMR (376 MHz, DMSO-d_6) δ −105.50– −105.57 (m); HRMS (ESI) m/z: $[M + H]^+$ Calcd. for $C_{16}H_{15}FN_3OS$ 316.0914, found 316.0903.

3-(1-Methyl-1*H*-indazol-5-yl)-5-(methylthio)benzamide (**39**)

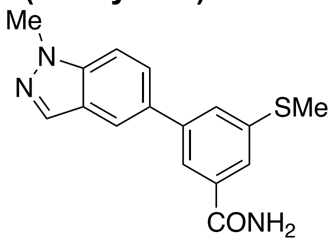

The reaction was performed as described in general procedure 8 using 3-bromo-5-(methylthio)benzamide (**S8**) (36.9 mg, 0.150 mmol), *N*-methylindazole-5-boronic acid (39.6 mg, 0.225 mmol), XPhos-Pd-G2 (5.9 mg, 7.50 µmol), and potassium phosphate (55.8 mg, 0.263 mmol) in 9:1 toluene/water (2 mL). Purification by flash column chromatography (petroleum ether/ethyl acetate, 1:1) followed by trituration in acetone 3-(1-methyl-1*H*-indazol-5-yl)-5-(methylthio)benzamide (**39**) (5.00 mg, 11%) as a white solid. FT-IR (neat) v_{max} 3341, 3158, 1652, 1618, 1572, 1507, 1436, 1389, 1348, 1324, 1310, 1283, 1213, 1108, 1063 cm^{-1}; ^1H NMR (400 MHz, DMSO-d_6) δ 8.15 (br s, 1H), 8.13–8.12 (m, 1H), 8.11 (d, J = 0.7 Hz, 1H), 7.97 (app. t, J = 1.6 Hz, 1H), 7.80 (dd, J = 8.8, 1.7 Hz, 1H), 7.74 (d, J = 8.8 Hz, 1H), 7.70 (app. t, J = 1.6 Hz, 1H), 7.69 (app. t, J = 1.6 Hz, 1H), 7.47 (br s, 1H), 4.08 (s, 3H), 2.59 (s, 3H); ^{13}C NMR (101 MHz, DMSO-d_6) δ 167.4, 141.3, 139.3, 135.5, 132.9, 131.7, 126.5, 125.7, 124.1, 123.1, 122.5, 119.0, 110.2, 35.5, 14.6; HRMS (ESI) m/z: $[M - H]^+$ Calcd. for $C_{16}H_{14}N_3OS$ 296.0863, found 296.0862.

4-(Benzylthio)-3-(1-methyl-1*H*-indazol-5-yl)benzamide (40)

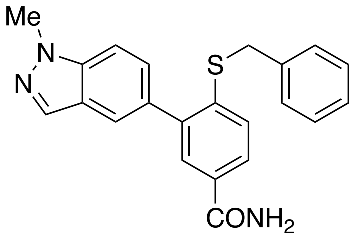

The reaction was performed as described in general procedure 8 using 4-(benzylthio)-3-bromobenzamide (**S9**) (50.0 mg, 0.160 mmol), 1-methyl-1*H*-indazole-5-boronic acid (57.0 mg, 0.270 mmol), tetrakis(triphenylphosphine)palladium(0) (18.0 mg, 16.0 μmol), and potassium phosphate tribasic (57.0 mg, 0.270 mmol) in 9:1 *N*,*N*-dimethylformamide/water (2 mL). Purification by flash column chromatography (petroleum ether/ethyl acetate 9:1 to 1:1 to 0:1) afforded 4-(benzylthio)-3-(1-methyl-1*H*-indazol-5-yl)benzamide (**40**) (37.3 mg, 63%) as a colorless solid. FT-IR (neat) ν_{max} 3366, 3152, 2923, 2526, 2355, 1742, 1687, 1657, 1599, 1556, 1456, 1387, 1242, 1225, 1070, 893, 813, 759, 702, 690 cm^{-1}; ^{1}H NMR (500 MHz, CD_3OD) δ 8.06 (d, *J* = 1.0 Hz, 1H), 7.82 (dd, *J* = 8.2, 2.1 Hz, 1H), 7.79 (d, *J* = 2.0 Hz, 1H), 7.76–7.73 (m, 1H), 7.62–7.54 (m, 2H), 7.47 (dd, *J* = 8.7, 1.6 Hz, 1H), 7.31–7.19 (m, 6H), 4.13 (s, 3H), 4.12 (s, 2H); ^{13}C NMR (126 MHz, CD_3OD) δ 143.0, 142.5, 140.8, 138.1, 134.1, 134.0, 131.8, 130.6, 129.9, 129.8, 129.5, 128.6, 128.3, 127.9, 125.1, 122.8, 110.0, 38.3, 35.6; HRMS (ESI) m/z: $[M + H]^+$ Calcd. for $C_{22}H_{20}N_3OS$ 374.1322; Found 374.1326.

4-Methyl-3-(1-methyl-1*H*-indazol-5-yl)benzamide (41)

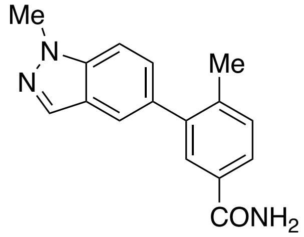

The reaction was performed as described in general procedure 7 using 3-bromo-4-methylbenzamide (**S10**) (100 mg, 0.480 mmol), 1-methyl-1*H*-indazole-5-boronic acid (123 mg, 0.700 mmol), tetrakis(triphenylphosphine)palladium(0) (55.0 mg, 48.0 μmol), and potassium phosphate tribasic (174 mg, 0.820 mmol) in 9:1 *N*,*N*-dimethylformamide/water (2 mL). Purification by flash column chromatography (petroleum ether/ethyl acetate 1:1 to 0:1) afforded 4-methyl-3-(1-methyl-1H-indazol-5-yl)benzamide (**41**) (65.0 mg, 51%) as a colorless solid. FT-IR (neat) ν_{max} 3435, 3064,

2999, 1660, 1489, 1441, 1316, 1186, 1124, 1029, 1000, 851, 810, 7222, 698 cm^{-1}; ^{1}H NMR (400 MHz, CD_3OD) δ 8.07 (d, *J* = 1.0 Hz, 1H), 7.80 (d, *J* = 7.3 Hz, 2H), 7.73 (dd, *J* = 1.6, 0.9 Hz, 1H), 7.64 (dt, *J* = 8.6, 0.9 Hz, 1H), 7.43 (ddd, *J* = 9.7, 8.5, 1.3 Hz, 2H), 4.13 (s, 3H), 2.33 (s, 3H); ^{13}C NMR (101 MHz, CD_3OD) δ 172.3, 143.6, 141.3, 140.6, 135.2, 134.0, 132.5, 131.6, 130.4, 129.6, 127.4, 125.3, 122.2, 110.1, 35.6, 20.8; HRMS (ESI) m/z: $[M + H]^+$ Calcd. for $C_{16}H_{16}N_3O$ 266.1288; Found 266.1288.

4-Ethyl-3-(1-methyl-1*H*-indazol-5-yl)benzamide (42)

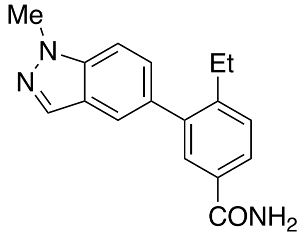

The reaction was performed as described in general procedure 7 using 3-bromo-4-ethylbenzamide (**S11**) (100 mg, 0.440 mmol), 1-methyl-1*H*-indazole-5-boronic acid (154 mg, 0.880 mmol), tetrakis(triphenylphosphine)palladium(0) (46.0 mg, 40.0 μmol), and potassium phosphate tribasic (159 mg, 0.750 mmol) in 9:1 *N*,*N*-dimethylformamide/water (2 mL). Purification by flash column chromatography (petroleum ether/ethyl acetate 1:1 to 0:1) afforded 4-ethyl-3-(1-methyl-1H-indazol-5-yl)benzamide (**42**) (46.7 mg, 38%) as a colorless solid. FT-IR (neat) v_{max} 3439, 3321, 2971, 1655, 1616, 1569, 1497, 1381, 1229, 1061, 994, 890, 858, 830, 774, 747, 687 cm^{-1}; ^{1}H NMR (500 MHz, $CDCl_3$) δ 8.00 (d, *J* = 1.0 Hz, 1H), 7.77 (dd, *J* = 8.0, 2.0 Hz, 1H), 7.69 (d, *J* = 2.0 Hz, 1H), 7.63 (t, *J* = 1.2 Hz, 1H), 7.42 (dd, *J* = 17.2, 8.3 Hz, 2H), 7.33 (dd, *J* = 8.5, 1.6 Hz, 1H), 5.96 (br s, 2H), 4.12 (s, 3H), 2.64 (q, J = 7.6 Hz, 2H), 1.10 (t, J = 7.5 Hz, 3H); ^{13}C NMR (126 MHz, $CDCl_3$) δ 169.3, 146.7, 142.2, 139.3, 133.5, 133.0, 130.8, 129.5, 129.1, 128.1, 126.6, 124.2, 121.3, 108.7, 35.8, 26.5, 15.4; HRMS (ESI) m/z: $[M + H]^+$ Calcd. for $C_{17}H_{18}N_3O$ 280.1444; Found 280.1445.

3-(1-Methyl-1*H*-indazol-5-yl)benzamide (43)

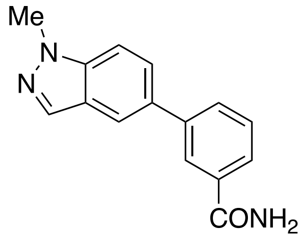

The reaction was performed as described in general procedure 7 using 3-bromobenzamide (100 mg, 0.500 mmol), 1-methyl-1*H*-indazole-5-boronic acid (176 mg, 1.00 mmol), tetrakis(triphenylphosphine)palladium(0) (58.0 mg, 50.0 µmol), and potassium phosphate tribasic (180 mg, 0.850 mmol) in 9:1 *N*,*N*-dimethylformamide/water (2 mL). Purification by flash column chromatography (petroleum ether/ethyl acetate 1:1 to 0:1) afforded 3-(1-methyl-1*H*-indazol-5-yl)benzamide (**43**) (43.4 mg, 35%) as a colourless solid. FT-IR (neat) $ν_{max}$ 3329, 3141, 1670, 1629, 1582, 1439, 1402, 1227, 1176, 1122, 992, 882, 798, 759 cm^{-1}; ^{1}H NMR (500 MHz, DMSO-d_6) δ 8.21 (t, *J* = 1.8 Hz, 1H), 8.12 (s, 1H), 8.10 (d, *J* = 1.7 Hz, 2H), 7.89–7.72 (m, 4H), 7.54 (t, *J* = 7.7 Hz, 1H), 7.41 (s, 1H), 4.08 (s, 3H); ^{13}C NMR (101 MHz, DMSO-d_6) δ 167.8, 140.5, 139.2, 134.9, 132.9, 132.2, 129.5, 128.9, 126.0, 125.8, 125.6, 124.1, 118.7, 110.2, 35.4; HRMS (ESI) m/z: $[M + H]^+$ Calcd. for $C_{15}H_{14}N_3O$ 252.1131; Found 252.1134.

4-Fluoro-3-(1-methyl-1*H*-indazol-5-yl)benzamide (44)

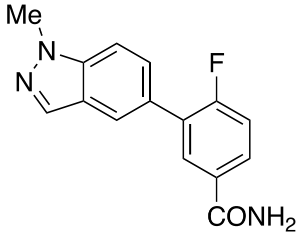

The reaction was performed as described in general procedure 7 using 3-bromo-4-fluorobenzamide (**S12**) (100 mg, 0.460 mmol), 1-methyl-1*H*-indazole-5-boronic acid (161 mg, 0.920 mmol), tetrakis(triphenylphosphine)palladium(0) (46.0 mg, 40.0 µmol), and potassium phosphate tribasic (165 mg, 0.780 mmol) in 9:1 *N*,*N*-dimethylformamide/water (2 mL). Purification by flash column chromatography (petroleum ether/ethyl acetate 1:1 to 0:1) afforded 4-fluoro-3-(1-methyl-1H-indazol-5-yl)benzamide (**44**) (25.6 mg, 21%) as a colorless solid. FT-IR (neat) $ν_{max}$ 3379, 3329, 3163, 3077, 1714, 1638, 1595, 1493, 1400, 1251, 1214, 1176, 1160, 1132, 996, 880,

808, 791, 768, 754 cm^{-1}; ^{1}H NMR (400 MHz, DMSO-d_6) δ 8.14 (d, J = 0.9 Hz, 1H), 8.11 (dd, J = 7.8, 2.4 Hz, 2H), 7.99 (q, J = 1.3 Hz, 1H), 7.91 (ddd, J = 8.5, 4.8, 2.3 Hz, 1H), 7.78–7.74 (m, 1H), 7.63 (dt, J = 8.7, 1.7 Hz, 1H), 7.44–7.37 (m, 2H), 4.09 (s, 3H); ^{13}C NMR (101 MHz, DMSO-d_6) δ 166.8, 160.8 (d, $J_{C\text{-}F}$ = 250.0 Hz), 131.0 (d, $J_{C\text{-}F}$ = 3.5 Hz), 130.5 (d, $J_{C\text{-}F}$ = 4.2 Hz), 128.6 (d, $J_{C\text{-}F}$ = 9.3 Hz), 128.3 (d, $J_{C\text{-}F}$ = 13.7 Hz), 127.2 (d, $J_{C\text{-}F}$ = 3.1 Hz), 126.7, 123.7, 121.2 (d, $J_{C\text{-}F}$ = 3.2 Hz), 116.1 (d, $J_{C\text{-}F}$ = 23.5 Hz), 35.5; ^{19}F NMR (376 MHz, DMSO-d_6) δ −114.9; HRMS (ESI) m/z: [M + H]$^+$ Calcd. for $C_{15}H_{13}FN_3O$ 270.1037; Found 270.1040.

3-(1-methyl-1*H*-indazol-5-yl)-4-(trifluoromethyl)benzamide (45)

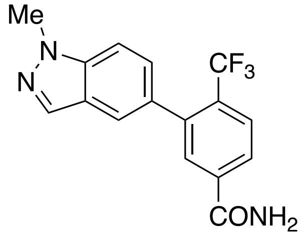

The reaction was performed as described in general procedure 7 using 3-bromo-4-(trifluoromethyl)benzamide (**S13**) (100 mg, 0.370 mmol), 1-methyl-1*H*-indazole-5-boronic acid (132 mg, 0.750 mmol), tetrakis(triphenylphosphine)palladium(0) (34.0 mg, 30.0 µmol), and potassium phosphate tribasic (133 mg, 0.630 mmol) in 9:1 *N*,*N*-dimethylformamide/water (2 mL). Purification by flash column chromatography (petroleum ether/ethyl acetate 1:1 to 0:1) afforded 3-(1-methyl-1*H*-indazol-5-yl)-4-(trifluoromethyl)benzamide (**45**) (49.6 mg, 42%) as a colorless solid. FT-IR (neat) $ν_{max}$ 3435, 3308, 3172, 1681, 1608, 1498, 1394, 1312, 1173,1132, 1111, 1031, 908, 862, 785 cm^{-1}; ^{1}H NMR (400 MHz, $CDCl_3$) δ 8.02 (d, J = 0.9 Hz, 1H), 7.91 (ddd, J = 8.3, 1.8, 0.9 Hz, 1H), 7.86 (d, J = 8.3 Hz, 1H), 7.81 (dt, J = 1.7, 0.7 Hz, 1H), 7.68 (dt, J = 1.5, 0.7 Hz, 1H), 7.44 (dt, J = 8.6, 0.9 Hz, 1H), 7.38–7.34 (m, 1H), 6.12 (s, 1H), 5.71 (s, 1H), 4.13 (s, 3H); ^{13}C NMR (101 MHz, $CDCl_3$) δ 167.9, 142.4, 139.6, 136.1, 133.3 (d, $J_{C\text{-}F}$ = 7.4 Hz), 131.7 (d, $J_{C\text{-}F}$ = 7.1 Hz), 127.7, 126.9 (q, $J_{C\text{-}F}$ = 5.0 Hz), 126.3 (d, $J_{C\text{-}F}$ = 7.2 Hz), 123.8, 121.5, 108.6 (d, $J_{C\text{-}F}$ = 7.6 Hz); ^{19}F NMR (376 MHz, $CDCl_3$) δ −57.3; HRMS (ESI) m/z: [M + H]$^+$ Calcd. for $C_{16}H_{13}F_3N_3O$ 320.1005; Found 320.1003.

4-Methoxy-3-(1-methyl-1*H*-indazol-5-yl)benzamide (46)

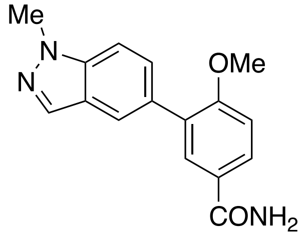

The reaction was performed as described in general procedure 7 using 3-bromo-4-methoxybenzamide (**S15**) (100 mg, 0.430 mmol), 1-methyl-1*H*-indazole-5-boronic acid (151 mg, 0.860 mmol), tetrakis(triphenylphosphine)palladium(0) (49.0 mg, 43.0 μmol), and potassium phosphate tribasic (155 mg, 0.730 mmol) in 9:1 *N*,*N*-dimethylformamide/water (2 mL). Purification by flash column chromatography (petroleum ether/ethyl acetate 1:1 to 0:1) afforded 4-methoxy-3-(1-methyl-1*H*-indazol-5-yl)benzamide (**46**) (60.9 mg, 50%) as a colorless solid. FT-IR (neat) ν_{max} 3360, 3156, 1687, 1631, 1508, 1452, 1389, 1258, 1240, 1178, 1130, 1022, 1037, 996, 910, 893, 728, 659 cm^{-1}; ^1H NMR (500 MHz, DMSO-d_6) δ 8.07 (s, 1H), 7.94–7.86 (m, 3H), 7.85 (d, J = 1.5 Hz, 1H), 7.66 (d, J = 8.7 Hz, 1H), 7.54 (dd, J = 8.7, 1.6 Hz, 1H), 7.18 (dd, J = 8.4, 4.9 Hz, 2H), 4.07 (s, 3H), 3.83 (s, 3H); ^{13}C NMR (101 MHz, DMSO-d_6) δ 167.4, 158.5, 138.8, 132.6, 130.1, 129.9, 129.6, 128.3, 128.1, 126.6, 123.5, 121.0, 111.1, 109.0, 55.8, 35.4; HRMS (ESI) m/z: $[M + H]^+$ Calcd. for $C_{16}H_{16}N_3O_2$ 282.1237; Found 282.1237.

4-(Benzyloxy)-3-(1-methyl-1*H*-indazol-5-yl)benzamide (47)

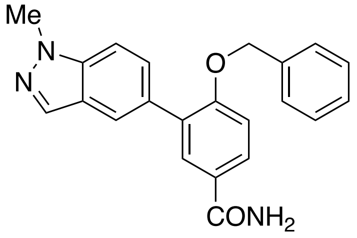

The reaction was performed as described in general procedure 7 using 4-(benzyloxy)-3-bromobenzamide (**S16**) (100 mg, 0.330 mmol), 1-methyl-1*H*-indazole-5-boronic acid (116 mg, 660 μmol), tetrakis(triphenylphosphine)palladium(0) (34.0 mg, 30.0 μmol), and potassium phosphate tribasic (118 mg, 0.560 mmol) in 9:1 *N*,*N*-dimethylformamide/water (2 mL). Purification by flash column chromatography (petroleum ether/ethyl acetate 1:1 to 0:1) afforded 4-(benzyloxy)-3-(1-methyl-1H-indazol-5-yl)benzamide (**47**) (105 mg, 89%) as a colorless solid. FT-IR (neat) ν_{max}

3368, 3144, 3038, 1677, 1625, 1605, 1498, 1389, 1353, 1262, 1238, 1223, 1128, 1027, 992, 895, 813, 757, 737, 687 cm^{-1}; ^{1}H NMR (500 MHz, $CDCl_3$) δ 7.99 (d, *J* = 1.0 Hz, 1H), 7.89 (d, *J* = 1.6 Hz, 1H), 7.86 (d, *J* = 2.4 Hz, 1H), 7.78 (dd, *J* = 8.6, 2.4 Hz, 1H), 7.63 (dd, *J* = 8.7, 1.6 Hz, 1H), 7.41 (d, *J* = 8.7 Hz, 1H), 7.35–7.27 (m, 5H), 7.07 (d, *J* = 8.6 Hz, 1H), 5.83 (d, *J* = 168.2 Hz, 2H), 5.17 (s, 2H), 4.11 (s, 3H); ^{13}C NMR (101 MHz, DMSO-d_6) δ 168.9, 158.7, 139.4, 136.6, 133.2, 131.6, 130.8, 130.2, 128.7, 128.7, 128.2, 128.0, 126.9, 126.2, 124.3, 121.9, 112.8, 108.5, 70.6, 35.8; HRMS (ESI) m/z: [M + H]$^+$ Calcd. for $C_{22}H_{20}N_3O_2$ 358.1550; Found 358.1552.

3-(1-Methyl-1*H*-indazol-5-yl)-4-(trifluoromethoxy)benzamide (48)

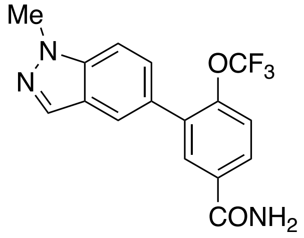

The reaction was performed as described in general procedure 7 using 3-bromo-4-(trifluoromethoxy)benzamide (100 mg, 0.350 mmol), 1-methyl-1*H*-indazole-5-boronic acid (124 mg, 0.710 mmol), tetrakis(triphenylphosphine)palladium(0) (34.0 mg, 30.0 μmol), and potassium phosphate tribasic (127 mg, 0.600 mmol) in 9:1 *N*,*N*-dimethylformamide/water (2 mL). Purification by flash column chromatography (petroleum ether/ethyl acetate 1:1 to 0:1) afforded 3-(1-methyl-1H-indazol-5-yl)-4-(trifluoromethoxy)benzamide (**48**) (82.3 mg, 70%) as a colorless solid. FT-IR (neat) ν_{max} 3357, 3165, 1703, 1685, 1629, 1396, 1266, 1251, 1225, 1173, 1161, 1148, 1109, 994, 892, 800, 787 cm^{-1}; ^{1}H NMR (400 MHz, $CDCl_3$) δ 8.04 (d, *J* = 0.9 Hz, 1H), 7.95 (d, *J* = 2.3 Hz, 1H), 7.84–7.81 (m, 2H), 7.54–7.42 (m, 3H), 6.06 (s, 1H), 5.67 (s, 1H), 4.13 (s, 3H); ^{13}C NMR (101 MHz, $CDCl_3$) δ 167.9, 149.1, 139.6, 135.9, 133.4, 132.2, 131.4, 128.4, 127.9, 127.6, 124.3, 122.0, 121.3, 109.1, 35.8; ^{19}F NMR (376 MHz, $CDCl_3$) δ −57.0; HRMS (ESI) m/z: [M + H]$^+$ Calcd for $C_{16}H_{13}F_3N_3O_2$ 336.0954; Found 336.0955.

4-Hydroxy-3-(1-methyl-1*H*-indazol-5-yl)benzamide (49)

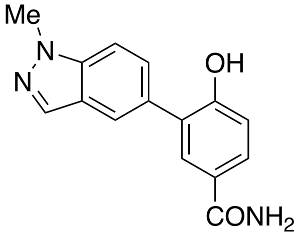

The reaction was performed as described in general procedure 7 using 3-bromo-4-((2-(trimethylsilyl)ethoxy)methoxy)benzamide (**S14**) (150 mg, 0.430 mmol), 1-methyl-1*H*-indazole-5-boronic acid (153 mg, 0.870 mmol), tetrakis(triphenylphosphine)palladium(0) (46.0 mg, 40.0 μmol), and potassium phosphate tribasic (155 mg, 0.730 mmol) in 9:1 *N*,*N*-dimethylformamide/water (2 mL). Purification by flash column chromatography (petroleum ether/ethyl acetate 1:1 to 0:1) afforded 3-(1-methyl-1*H*-indazol-5-yl)-4-((2-(trimethylsilyl)ethoxy)methoxy)benzamide (254 mg) as a colorless oil which was used directly in the next step without further purification.

To a stirred solution of 3-(1-methyl-1*H*-indazol-5-yl)-4-((2-(trimethylsilyl)ethoxy)methoxy)benzamide (100 mg, 0.250 mmol) in anhydrous tetrahydrofuran (5 mL) was added *tetra*-*n*-butylammonium fluoride (1.26 mL, 1.26 mmol). The resulting solution was stirred under reflux for 48 hours. The reaction mixture was cooled to room temperature and concentrated *in vacuo*. The residue was dissolved in ethyl acetate (20 mL) and washed with water (10 mL). The organic phase was dried ($MgSO_4$), filtered and concentrated *in vacuo*. Purification by flash column chromatography (petroleum ether/ethyl acetate 1:1 to 0:1) afforded 4-hydroxy-3-(1-methyl-1*H*-indazol-5-yl)benzamide (**49**) (29.4 mg, 44%) as a colorless solid. FT-IR (neat) ν_{max} 3349, 3156, 1677, 1616, 1605, 1573, 1508, 1409, 1284, 1221, 1128, 1104, 996, 982, 791 cm^{-1}; ^{1}H NMR (400 MHz, DMSO-d_6) δ 10.07 (s, 1H), 8.07 (d, *J* = 0.8 Hz, 1H), 7.93–7.85 (m, 2H), 7.82 (s, 1H), 7.71 (dd, *J* = 8.4, 2.3 Hz, 1H), 7.67–7.59 (m, 2H), 7.09 (s, 1H), 6.97 (d, *J* = 8.4 Hz, 1H), 4.07 (s, 3H); ^{13}C NMR (101 MHz, DMSO-d_6) δ 167.7, 157.1, 138.7, 132.6, 130.4, 130.3, 128.0, 127.5, 125.5, 123.6, 120.8, 115.5, 108.9, 35.4; HRMS (ESI) m/z: $[M + H]^+$ Calcd. for $C_{15}H_{14}N_3O_2$ 268.1081; Found 268.1085.

4-(Cyclopropylmethoxy)-3-(1-methyl-1*H*-indazol-5-yl)benzamide (50)

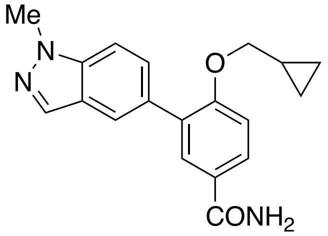

The reaction was performed as described in general procedure 7 using 3-bromo-4-(cyclopropylmethoxy)benzamide (**S17**) (100 mg, 0.370 mmol), 1-methyl-1*H*-indazole-5-boronic acid (130 mg, 0.740 mmol), tetrakis(triphenylphosphine)palladium(0) (34.0 mg, 30.0 µmol), and potassium phosphate tribasic (133 mg, 0.630 mmol) in 9:1 *N*,*N*-dimethylformamide/water (2 mL). Purification by flash column chromatography using a gradient system (petroleum ether/ethyl acetate 1:1 to 0:1) followed by trituration in diethyl ether afforded 4-(cyclopropylmethoxy)-3-(1-methyl-1*H*-indazol-5-yl)benzamide (**50**) (41.8 mg, 35%) as a colorless solid. FT-IR (neat) ν_{max} 3349, 3405, 3087, 2927, 2861, 1660, 1605, 1495, 1400, 1379, 1268, 1253, 1223, 1126, 1005, 898, 881, 789, 770, 677 cm^{-1}; ^{1}H NMR (400 MHz, DMSO-d_6) δ 8.08 (d, *J* = 0.8 Hz, 1H), 7.90 (t, *J* = 2.2 Hz, 3H), 7.84 (dd, *J* = 8.6, 2.4 Hz, 1H), 7.71–7.58 (m, 2H), 7.18 (s, 1H), 7.14 (d, *J* = 8.6 Hz, 1H), 4.08 (s, 3H), 3.94 (d, *J* = 6.7 Hz, 2H), 1.16 (tdd, *J* = 9.9, 5.4, 2.9 Hz, 1H), 0.57–0.44 (m, 2H), 0.35–0.27 (m, 2H); ^{13}C NMR (101 MHz, DMSO-d_6) δ 167.4, 157.9, 138.8, 132.6, 130.2, 130.1, 129.7, 128.2, 128.1, 126.6, 123.6, 121.0, 112.3, 108.8, 72.4, 35.4, 10.0, 2.9; HRMS (ESI) m/z: [M + H]$^+$ Calcd. for $C_{19}H_{20}N_3O_2$ 322.1550; Found 322.1548.

4-Isobutoxy-3-(1-methyl-1*H*-indazol-5-yl)benzamide (51)

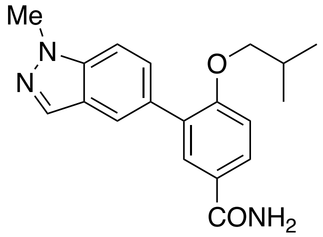

The reaction was performed as described in general procedure 7 using 3-bromo-4-isobutoxybenzamide (**S18**) (100 mg, 0.370 mmol), 1-methyl-1*H*-indazole-5-boronic acid (130 mg, 0.740 mmol), tetrakis(triphenylphosphine)palladium(0) (34.0 mg, 30.0 µmol), and potassium phosphate tribasic (134 mg, 0.630 mmol) in 9:1 *N*,*N*-dimethylformamide/water (2 mL). Purification by flash column chromatography

(petroleum ether/ethyl acetate 1:1 to 0:1) afforded 4-isobutoxy-3-(1-methyl-1*H*-indazol-5-yl)benzamide (**51**) (102 mg, 85%) as a colorless solid. FT-IR (neat) ν_{max} 3316, 3331, 3303, 3141, 2958, 2932, 2923, 2874, 1670, 1603, 1577, 1495, 1374, 1268, 1255, 1238, 1221, 1161, 1026, 931, 817, 793, 774, 733, 677 cm^{-1}; ^{1}H NMR (400 MHz, DMSO-d_6) δ 8.07 (d, *J* = 1.5 Hz, 1H), 7.92 (d, *J* = 2.4 Hz, 2H), 7.89–7.84 (m, 2H), 7.80–7.48 (m, 2H), 7.20 (s, 1H), 7.14 (dd, *J* = 8.7, 1.8 Hz, 1H), 4.07 (s, 3H), 3.83 (dd, *J* = 6.4, 2.1 Hz, 2H), 1.93 (dtd, *J* = 13.1, 6.6, 1.9 Hz, 1H), 0.90 (dd, *J* = 6.8, 2.0 Hz, 6H); ^{13}C NMR (101 MHz, DMSO-d_6) δ 167.5, 157.9, 138.7, 132.5, 130.2, 130.0, 129.6, 128.3, 128.2, 126.5, 123.5, 121.0, 111.8, 108.7, 74.2, 35.4, 27.7, 19.0; HRMS (ESI) m/z: $[M + H]^+$ Calcd. for $C_{19}H_{22}N_3O_2$ 324.1707; Found 324.1705.

4-(Benzylamino)-3-(1-methyl-1*H*-indazol-5-yl)benzamide (52)

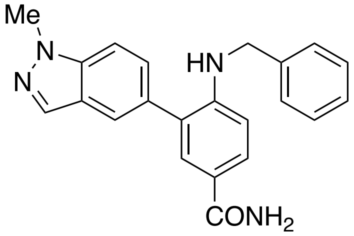

The reaction was performed as described in general procedure 7 using 4-(benzylamino)-3-bromobenzamide (**S19**) (50.0 mg, 0.160 mmol), 1-methyl-1*H*-indazole-5-boronic acid (56.0 mg, 0.320 mmol), tetrakis(triphenylphosphine)palladium(0) (18.0 mg, 16.0 μmol), and potassium phosphate tribasic (57.0 mg, 0.270 mmol) in 9:1 *N*,*N*-dimethylformamide/water (2 mL). Purification by flash column chromatography (petroleum ether/ethyl acetate 1:1 to 0:1 to ethyl acetate/methanol 9:1) afforded 4-(benzylamino)-3-(1-methyl-1*H*-indazol-5-yl)benzamide (**52**) (19.0 mg, 33%) as a colorless solid. FT-IR (neat) ν_{max} 3325, 3185, 2927, 1655, 1599, 1523, 1428, 1374, 1363, 1331, 1223, 996, 906, 895, 821, 728, 698 cm^{-1}; ^{1}H NMR (400 MHz, $CDCl_3$) δ 8.00 (d, *J* = 0.8 Hz, 1H), 7.80–7.75 (m, 1H), 7.71–7.62 (m, 3H), 7.50–7.43 (m, 3H), 7.35–7.26 (m, 4H), 6.66 (d, *J* = 8.5 Hz, 1H), 5.74 (br s, 2H), 4.38 (d, *J* = 5.6 Hz, 2H), 4.11 (s, 3H); ^{13}C NMR (101 MHz, $CDCl_3$) δ 169.3, 148.5, 139.5, 138.6, 133.0, 130.6, 130.4, 128.9, 128.6, 128.0, 127.5, 127.4, 127.1, 124.7, 121.9, 121.5, 109.9, 109.9, 47.8, 35.8; HRMS (ESI) m/z: $[M + H]^+$ Calcd. for $C_{22}H_{21}N_4O$ 357.1710; Found 357.1710.

3-(1-Methyl-1*H*-indazol-5-yl)-4-morpholinobenzamide (53)

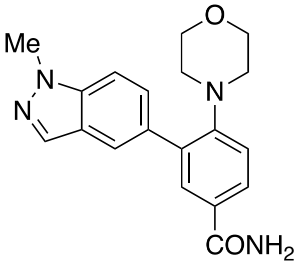

The reaction was performed as described in general procedure 7 using 3-bromo-4-morpholinobenzamide (**S20**) (38.5 mg, 0.135 mmol), (1-methyl-1*H*-indazol-5-yl)boronic acid (35.9 mg, 0.203 mmol), tetrakis(triphenylphosphine)palladium(0) (15.6 mg, 13.5 μmol), and potassium phosphate tribasic (71.6 mg, 0.338 mmol) in 9:1 *N*,*N*-dimethylformamide/water (2 mL). Purification by flash column chromatography (petroleum ether/ethyl acetate, 1:1 to 0:1 to ethyl acetate/methanol, 19:1) followed by trituration in toluene afforded 3-(1-methyl-1*H*-indazol-5-yl)-4-morpholinobenzamide (**53**) (26.6 mg, 59%) as a white solid. IR (neat) 3391, 3350, 3208, 2957, 1665, 1620, 1599, 1422, 1366, 1350, 1227, 1207, 1111, 922, 779 cm^{-1}; ^{1}H NMR (500 MHz, $CDCl_3$) δ 8.00 (br s, 1H), 7.90 (br s, 1H), 7.79–7.71 (m, 3H), 7.43 (d, *J* = 8.7 Hz, 1H), 7.05 (d, *J* = 8.2 Hz, 1H), 5.81 (br s, 2H), 4.12 (s, 3H), 3.61–3.54 (m, 4H), 2.92–2.84 (m, 4 H); ^{13}C NMR (126 MHz, $CDCl_3$) δ 168.9, 153.6, 139.3, 134.6, 133.1, 133.1, 131.7, 127.8, 127.7, 127.2, 124.6, 120.7, 117.8, 109.0, 66.9, 51.0, 35.8; HRMS (ESI) m/z: $[M + H]^+$ Calcd. for $C_{19}H_{21}N_4O_2$ 337.1659; Found 337.1648.

3-(1-Methyl-1*H*-indazol-5-yl)-4-((2-morpholinoethyl)amino)benzamide (54)

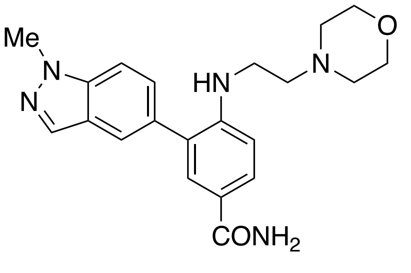

The reaction was performed as described in general procedure 7 using 3-bromo-4-((2-morpholinoethyl)amino)benzamide (**S21**) (40.0 mg, 0.122 mmol), (1-methyl-1*H*-indazol-5-yl)boronic acid (33.0 mg, 0.183 mmol), tetrakis(triphenylphosphine)palladium(0) (14.0 mg, 12.2 μmol), and potassium phosphate tribasic (65.0 mg, 0.306 mmol) in 9:1 *N*,*N*-dimethylformamide/water (2 mL). The reaction mixture was stirred at 90 °C for 16 h. After cooling to room temperature,

the reaction mixture was diluted with ethyl acetate (10 mL) and washed with 2 M aqueous hydrochloric acid (3 × 20 mL). The combined aqueous washings were basified by the addition of 4 M aqueous sodium hydroxide (20 mL) and extracted with ethyl acetate (3 × 50 mL). The combined organic extracts were washed with brine (100 mL), dried ($MgSO_4$), filtered and concentrated *in vacuo*. Purification by flash column chromatography (ethyl acetate to ethyl acetate/methanol, 9:1) afforded 3-(1-methyl-1*H*-indazol-5-yl)-4-((2-morpholinoethyl)amino)benzamide (**54**) (32.8 mg, 71%) as a brown solid. FT-IR (neat) ν_{max} 3383, 3289, 3163, 2924, 1663, 1599, 1518, 1431, 1377, 1333, 1287, 1219, 1177, 1107, 746 cm^{-1}; ^1H NMR (500 MHz, DMSO-*d*$_6$) δ 8.07 (s, 1H), 7.82–7.71 (m, 3H), 7.66 (br s, 1H), 7.63 (d, *J* = 2.2 Hz, 1H), 7.41 (dd, *J* = 8.5, 1.7 Hz, 1H), 6.90 (br s, 1H), 6.69 (d, *J* = 8.5 Hz, 1H), 5.27 (t, *J* = 5.0 Hz, 1H), 4.10 (s, 3H), 3.41–3.35 (m, 4H), 3.17 (q, *J* = 6.0 Hz, 2H), 2.46 (t, *J* = 6.0 Hz, 2H), 2.31–2.25 (m, 4H); ^{13}C NMR (126 MHz, DMSO-*d*$_6$) δ 167.9, 147.9, 138.9, 132.4, 130.7, 129.8, 128.5, 127.6, 126.2, 124.0, 121.6, 120.9, 110.2, 109.4, 66.2, 55.6, 52.6, 35.4; HRMS (ESI) m/z: $[M + H]^+$ Calcd. for $C_{21}H_{26}N_5O_2$ 380.2081; Found 380.2067.

3-(1-Methyl-1*H*-indazol-5-yl)-4-((2-(piperidin-1-yl)ethyl)amino)benzamide (55)

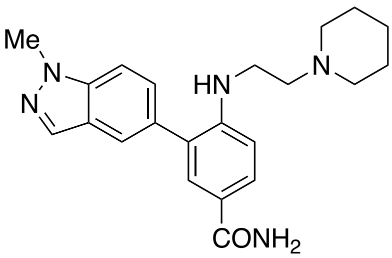

The reaction was performed as described in general procedure 7 using 3-bromo-4-((2-(piperidin-1-yl)ethyl)amino)benzamide (**S22**) (40.0 mg, 0.123 mmol), (1-methyl-1*H*-indazol-5-yl)boronic acid (33.0 mg, 0.184 mmol), tetrakis(triphenylphosphine)palladium(0) (14.2 mg, 12.3 μmol), and potassium phosphate tribasic (65.0 mg, 0.306 mmol) in 9:1 *N*,*N*-dimethylformamide/water (2 mL). The reaction mixture was stirred at 90 °C for 16 h. After cooling to room temperature, the reaction mixture was diluted with ethyl acetate (10 mL) and washed with 2 M aqueous hydrochloric acid (3 × 20 mL). The combined aqueous washings were basified by the addition of 4 M aqueous sodium hydroxide (20 mL) and extracted with ethyl acetate (3 × 50 mL). The combined organic extracts were washed with brine (100 mL), dried ($MgSO_4$), filtered and concentrated *in vacuo*. Purification by flash column chromatography (ethyl acetate/methanol, 4:1 with 1% triethylamine) afforded 3-(1-

methyl-1*H*-indazol-5-yl)-4-((2-(piperidin-1-yl)ethyl)amino)benzamide (**55**) (35.1 mg, 76%) as a yellow solid. FT-IR (neat) ν_{max} 3347, 3208, 2936, 2812, 1661, 1599, 1516, 1368, 1221, 1111, 922, 756, 671 cm^{-1}; ^{1}H NMR (500 MHz, DMSO-d_6) δ 8.03 (s, 1H), 7.76 (d, *J* = 8.4 Hz, 2H), 7.69 (d, *J* = 8.6 Hz, 1H), 7.64 (d, *J* = 2.1 Hz, 1H), 7.40 (dd, *J* = 8.6, 1.6 Hz, 1H), 6.91 (s, 2H), 6.70 (d, *J* = 8.5 Hz, 1H), 5.09 (s, 1H), 4.08 (s, 3H), 3.19 (s, 2H), 2.96 (s, 2H), 2.32 (s, 4H), 1.33 (s, 6H); ^{13}C NMR (126 MHz, DMSO-d_6) δ 167.5, 147.6, 138.7, 131.9, 130.3, 129.3, 127.9, 127.1, 126.1, 123.7, 121.6, 120.4, 109.4, 109.0, 55.6, 53.0 (× 2), 34.8 (× 2), 24.7; HRMS (ESI) m/z: [M + H]$^+$ Calcd. for $C_{22}H_{28}N_5O$ 378.2288; Found 378.2274.

3-(1-Methyl-1*H*-indazol-5-yl)-4-((3-morpholinopropyl)amino)benzamide (56)

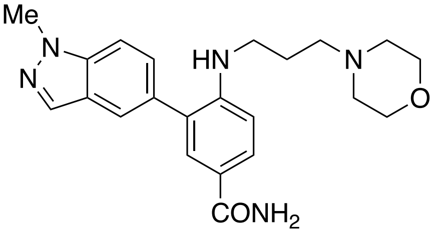

The reaction was performed as described in general procedure 7 using 3-bromo-4-((3-morpholinopropyl)amino)benzamide (**S23**) (40.0 mg, 0.117 mmol), (1-methyl-1*H*-indazol-5-yl)boronic acid (31.0 mg, 0.175 mmol), tetrakis(triphenylphosphine)palladium(0) (13.5 mg, 11.7 µmol), and potassium phosphate tribasic (62.0 mg, 0.293 mmol) in 9:1 *N*,*N*-dimethylformamide/water (2 mL). The reaction mixture was stirred at 90 °C for 16 h. After cooling to room temperature, the reaction mixture was diluted with ethyl acetate (10 mL) and washed with 2 M aqueous hydrochloric acid (3 × 20 mL). The combined aqueous washings were basified by the addition of 4 M aqueous sodium hydroxide (20 mL) and extracted with ethyl acetate (3 × 50 mL). The combined organic extracts were washed with brine (100 mL), dried ($MgSO_4$), filtered and concentrated *in vacuo*. Purification by flash column chromatography (ethyl acetate/methanol, 17:3) followed by trituration from diethyl ether then toluene/water (9:1) afforded 3-(1-methyl-1*H*-indazol-5-yl)-4-((3-morpholinopropyl)amino)benzamide (**56**) (32.3 mg, 70%) as a pink solid. FT-IR (neat) ν_{max} 3374, 3154, 2945, 1668, 1599, 1427, 1377, 1113, 827, 756, 675 cm^{-1}; ^{1}H NMR (500 MHz, $CDCl_3$) δ 8.00 (d, *J* = 0.9 Hz, 1H), 7.77–7.71 (m, 2H), 7.58 (d, *J* = 2.2 Hz, 1H), 7.48 (d, *J* = 8.6 Hz, 1H), 7.41 (dd, *J* = 8.6, 1.5 Hz, 1H), 6.71 (d, *J* = 8.6 Hz, 1H), 5.68 (br s, 2H), 4.60 (br s, 1H), 4.12 (s, 3H), 3.56–3.35 (m, 4H), 3.28–3.20 (m, 2H), 2.42–2.24 (m, 6H), 1.80–1.68 (m, 2H); ^{13}C NMR (126 MHz, $CDCl_3$) δ 169.3, 149.0,

139.5, 133.0, 130.8, 130.4, 128.8, 128.1, 127.2, 124.7, 121.8, 120.9, 109.8, 109.4, 66.7, 56.9, 53.9 (2 × CH_2), 42.4, 35.8 (2 × CH_2), 25.6; HRMS (ESI) m/z: $[M + H]^+$ Calcd. for $C_{22}H_{28}N_5O_2$ 394.2238; Found 394.2220.

4-((2-(Methylamino)ethyl)amino)-3-(1-methyl-1*H*-indazol-5-yl)benzamide (57)

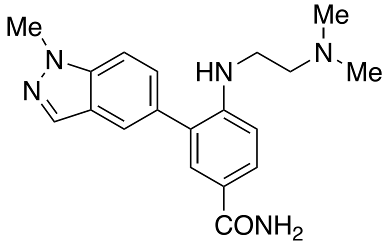

The reaction was performed as described in general procedure 7 using 3-bromo-4-((2-(dimethylamino)ethyl)amino)benzamide (**S24**) (50.0 mg, 180 μmol), 1-methyl-1*H*-indazole-5-boronic acid (63.0 mg, 360 μmol), tetrakis(triphenylphosphine)palladium(0) (12.0 mg, 10.0 μmol), and potassium phosphate tribasic (66.0 mg, 0.310 mmol) in 9:1 *N*,*N*-dimethylformamide/water (2 mL). Purification by flash column chromatography (dichloromethane/methanol 9:1) afforded 4-((2-(methylamino)ethyl)amino)-3-(1-methyl-1*H*-indazol-5-yl)benzamide (**57**) (17.6 mg, 29%) as a colorless solid. FT-IR (neat) ν_{max} 3368, 3358, 3349, 3200, 2977, 2943, 2863, 1659, 1605, 1526, 1379, 1296, 1275 cm^{-1}; ^1H NMR (400 MHz, $CDCl_3$) δ 8.00 (d, *J* = 0.9 Hz, 1H), 7.77–7.70 (m, 2H), 7.61 (d, *J* = 2.3 Hz, 1H), 7.50–7.38 (m, 2H), 6.68 (d, *J* = 8.5 Hz, 1H), 5.68 (s, 2H), 4.94 (t, *J* = 4.9 Hz, 1H), 4.12 (s, 3H), 3.21 (td, *J* = 6.2, 4.9 Hz, 2H), 2.47 (t, *J* = 6.2 Hz, 2H), 2.15 (s, 6H); ^{13}C NMR (101 MHz, $CDCl_3$) δ 169.4, 149.0, 133.0, 130.8, 130.4, 128.7, 128.1, 127.2, 124.7, 121.7, 120.9, 109.6, 109.6, 57.6, 45.2, 41.2, 35.8; HRMS (ESI) m/z: $[M + H]^+$ Calcd. for $C_{19}H_{24}N_5O$ 338.1975; Found 338.1978.

Methyl 4-(methylthio)-3-(pyrimidin-5-yl)benzoate (S26)

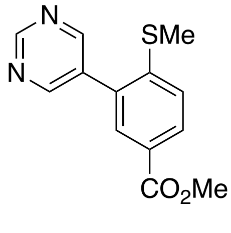

The reaction was performed as described in general procedure **7** methyl 3-bromo-4-(methylthio)benzoate **7b** (874 mg, 3.35 mmol), pyrimidin-5-ylboronic acid (830 mg,

6.70 mmol), tetrakis(triphenylphosphine)palladium(0) (393 mg, 0.335 mmol), and potassium phosphate tribasic (1.21 g, 5.70 mmol) in 9:1 *N*,*N*-dimethylformamide/water (20 mL). Purification by flash column chromatography (pet. ether/EtOAc 1:1) followed by trituration with diethyl ether afforded methyl 4-(methylthio)-3-(pyrimidin-5-yl)benzoate (**S26**) (483 mg, 55%) as a colorless solid. FT-IR (neat) ν_{max} 2962, 2925, 2858, 1729, 1599, 1554, 1418, 1316, 1266, 1195, 1122, 763, 735 cm^{-1}; ^{1}H NMR (500 MHz, $CDCl_3$) δ 9.25 (s, 1H), 8.82 (s, 2H), 8.08 (dd, *J* = 8.3, 1.8 Hz, 1H), 7.85 (d, *J* = 1.8 Hz, 1H), 7.34 (d, *J* = 8.3 Hz, 1H), 3.92 (s, 3H), 2.48 (s, 3H); ^{13}C NMR (126 MHz, $CDCl_3$) δ 166.5, 158.2, 157.1, 144.8, 133.5, 133.0, 131.0, 130.6, 127.0, 124.6, 52.4, 15.6; HRMS (ESI) m/z: $[M + H]^+$ Calcd. for $C_{13}H_{13}O_2N_2S$ 261.0692; found 261.0696

4-(Methylthio)-3-(pyrimidin-5-yl)benzoic acid (58)

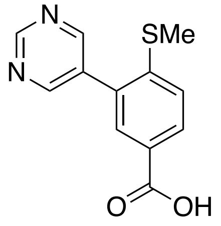

To a stirred solution of methyl 4-(methylthio)-3-(pyrimidin-5-yl)benzoate (**S26**) (433 mg, 1.67 mmol) in methanol/tetrahydrofuran (1:1, 25 mL) was added 4 M NaOH (267 mg, 6.68 mmol) and stirred at room temperature for 18 hours. The reaction mixture was concentrated under reduced pressure and acidified with 12 M HCl. The resulting precipitate was filtered and dried under vacuum to afford 4-(methylthio)-3-(pyrimidin-5-yl)benzoic acid (**58**) (366 mg, 1.48 mmol, 89%) as an off-white solid. The acid was of sufficient purity to be used in the subsequent steps without purification. FT-IR (neat) ν_{max} 3061, 2928, 2858, 1689, 1599, 1556, 1415, 1390, 1275, 1253, 1193, 767, 737 cm^{-1}; ^{1}H NMR (500 MHz, DMSO-d_6) δ 13.03 (s, 1H), 9.24 (s, 1H), 8.88 (s, 2H), 8.01 (d, *J* = 8.2 Hz, 1H), 7.78 (s, 1H), 7.52 (d, *J* = 8.2 Hz, 1H), 2.50 (s, 3H); ^{13}C NMR (126 MHz, DMSO-d_6) δ 166.7, 157.8, 156.7, 143.9, 132.9, 132.5, 130.5, 130.2, 127.2, 124.8, 14.7; HRMS (ESI) m/z: [M + H]+ Calcd. for $C_{12}H_{11}O_2N_2S$ 247.0536; found 247.0536.

(4-(Methylthio)-3-(pyrimidin-5-yl)phenyl)(piperidin-1-yl)methanone (59)

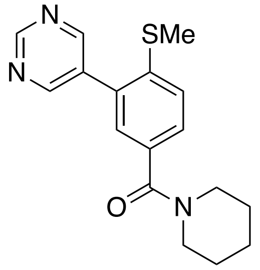

The reaction was performed as described in general procedure **2** using 4-(methylthio)-3-(pyrimidin-5-yl)benzoic acid **58** (50.0 mg, 0.203 mmol), oxalyl chloride (40.1 μL, 0.467 mmol), *N*,*N*-dimethylformamide (80.5 μL, 1.04 mmol), and piperidine (101 μL, 1.02 mmol) in tetrahydrofuran (5 mL). Purification by flash column chromatography (petroleum ether/ethyl acetate, 1:1 to 0:1) followed by trituration with diethyl ether afforded (4-(methylthio)-3-(pyrimidin-5-yl)phenyl)(piperidin-1- yl)methanone (**59**) (13.2 mg, 21%) as an off-white solid. FT-IR (neat) ν_{max} 2936, 2858, 1629, 1544, 1443, 1415, 1262, 1119, 1005, 759, 731 cm^{-1};^{1}H NMR (400 MHz, $CDCl_3$) δ 9.23 (s, 1H), 8.82 (s, 2H), 7.46 (dd, *J* = 8.2, 1.9 Hz, 1H), 7.35 (d, *J* = 8.2 Hz, 1H), 7.25 (d, *J* = 1.9 Hz, 1H), 3.71–3.43 (m, 4H), 2.43 (s, 3H), 1.67 (br s, 4H), 1.61 (br s, 2H); ^{13}C NMR (101 MHz, $CDCl_3$) δ 169.3, 158.1, 157.1, 140.0, 138.1, 133.7, 128.9, 128.3, 126.0, 125.8, 66.0, 24.7, 16.1, 15.4; HRMS (ESI) m/z: $[M + Na]^+$ Calcd. for $C_{17}H_{19}N_3NaOS$ 336.1141, found 336.1139.

(4-(Methylthio)-3-(pyrimidin-5-yl)phenyl)(morpholino)methanone (60)

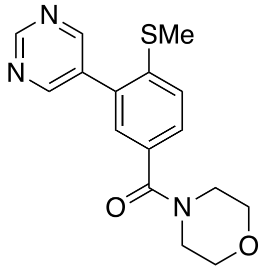

The reaction was performed as described in general procedure **2** using 4-(methylthio)-3-(pyrimidin-5-yl)benzoic acid **58** (50.0 mg, 0.203 mmol), oxalyl chloride (40.1 μL, 0.467 mmol), *N*,*N*-dimethylformamide (80.5 μL, 1.04 mmol) and morpholine (88.0 μL, 1.02 mmol) in tetrahydrofuran (5 mL). Purification by flash column chromatography (petroleum ether/ethyl acetate 3:7) afforded (4-(methylthio)-3-(pyrimidin-5-yl)phenyl)(morpholino)methanone (**60**) (20.9 mg, 33%) as a colorless solid. FT-IR (neat) ν_{max} 3008, 2927, 1714, 1616, 1554, 1439, 1363, 1223, 1117, 1014, 905, 847,

761, 731 cm^{-1}; ^{1}H NMR (400 MHz, $CDCl_3$) δ 9.24 (s, 1H), 8.81 (s, 2H), 7.47 (dd, J = 8.2, 1.8 Hz, 1H), 7.35 (d, J = 8.2 Hz, 1H), 7.27 (d, J = 1.8 Hz, 1H), 3.71 (br s, 8H), 2.44 (s, 3H); ^{13}C NMR (101 MHz, $CDCl_3$) δ 169.4, 158.2, 157.0, 140.9, 133.7, 133.5, 132.3, 129.2, 128.5, 125.6, 67.0, 16.0; HRMS (ESI) m/z: $[M + H]^+$ Calcd. for $C_{16}H_{18}N_3O_2S$ 316.1114, found 316.1114.

References

1. Hay, C.W.; McEwan, I.J. The Impact of Point Mutations in the Human Androgen Receptor: Classification of Mutations on the Basis of Transcriptional Activity, *PLoS ONE* **2012**, *7*, e32514.

2. Bradford, M. M., A rapid and sensitive method for the quantitation of microgram quantities of protein utilizing the principle of protein-dye binding, *Anal. Biochem.*, **1976**, *7*, 248–254.

3. Sander, T.; Freyss, J.; von Korff, M.; Rufener, C. DataWarrior: An Open-Source Program for Chemistry Aware Data Visualization and Analysis, *J. Chem. Inf. Model.,* **2015**, *55*, 460–473.

4. K. Mallareddy, J. Miura, H. Wang, X. Wang, J. D. Lawson, J. Tyhonas, *2-Oxo-2,3-Dihydro-1H-Imidazo[4,5-b]Pyridin-6-Yl)-4-Methylbenzamide Derivatives and Similar Compounds as RIPK2 Inhibitors for Treating e.g. Autoimmune Diseases*, *Int. Pat. Appl.,* PCT/US2020/023984, March 20th 2020.

7. HPLC data for Final Compounds

HPLC Trace of 3-(isoxazol-5-yl)-4-(methylthio)benzamide (7)

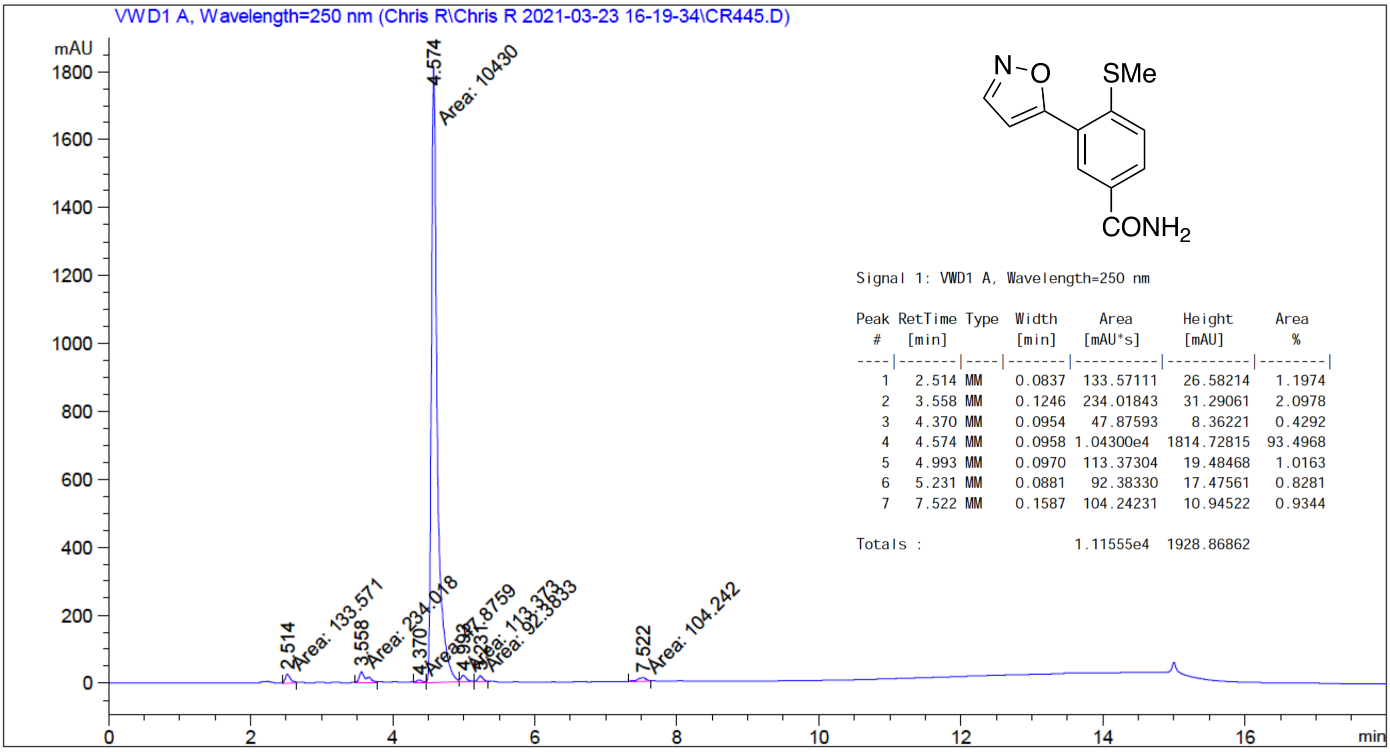

Signal 1: VWD1 A, Wavelength=250 nm

Peak #	RetTime [min]	Type	Width [min]	Area [mAU*s]	Height [mAU]	Area %
1	2.514	MM	0.0837	133.57111	26.58214	1.1974
2	3.558	MM	0.1246	234.01843	31.29061	2.0978
3	4.370	MM	0.0954	47.87593	8.36221	0.4292
4	4.574	MM	0.0958	1.04300e4	1814.72815	93.4968
5	4.993	MM	0.0970	113.37304	19.48468	1.0163
6	5.231	MM	0.0881	92.38330	17.47561	0.8281
7	7.522	MM	0.1587	104.24231	10.94522	0.9344
Totals :				1.11555e4	1928.86862	

HPLC Trace of 6-(methylthio)-[1,1'-biphenyl]-3-carboxamide (8)

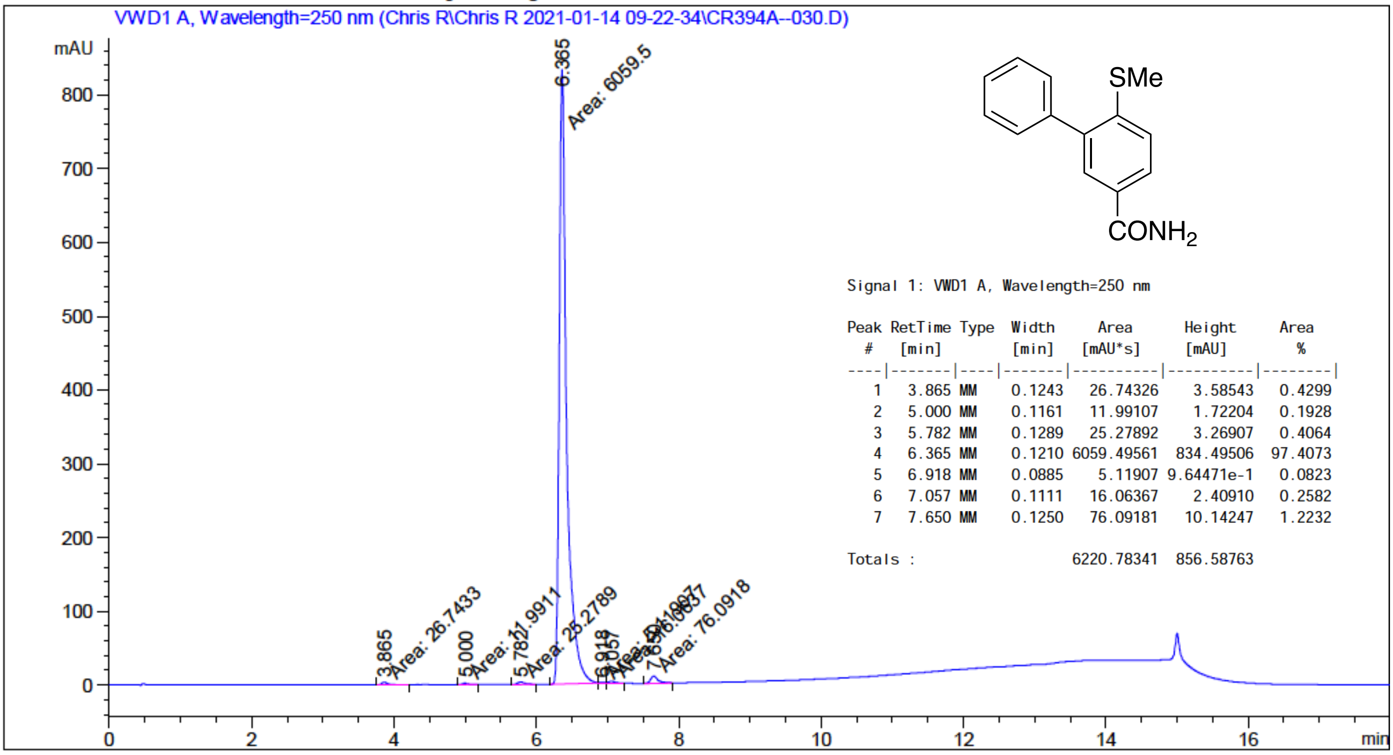

Signal 1: VWD1 A, Wavelength=250 nm

Peak #	RetTime [min]	Type	Width [min]	Area [mAU*s]	Height [mAU]	Area %
1	3.865	MM	0.1243	26.74326	3.58543	0.4299
2	5.000	MM	0.1161	11.99107	1.72204	0.1928
3	5.782	MM	0.1289	25.27892	3.26907	0.4064
4	6.365	MM	0.1210	6059.49561	834.49506	97.4073
5	6.918	MM	0.0885	5.11907	9.64471e-1	0.0823
6	7.057	MM	0.1111	16.06367	2.40910	0.2582
7	7.650	MM	0.1250	76.09181	10.14247	1.2232
Totals :				6220.78341	856.58763	

HPLC Trace of 4-(methylthio)-3-(thiophen-2-yl)benzamide (9)

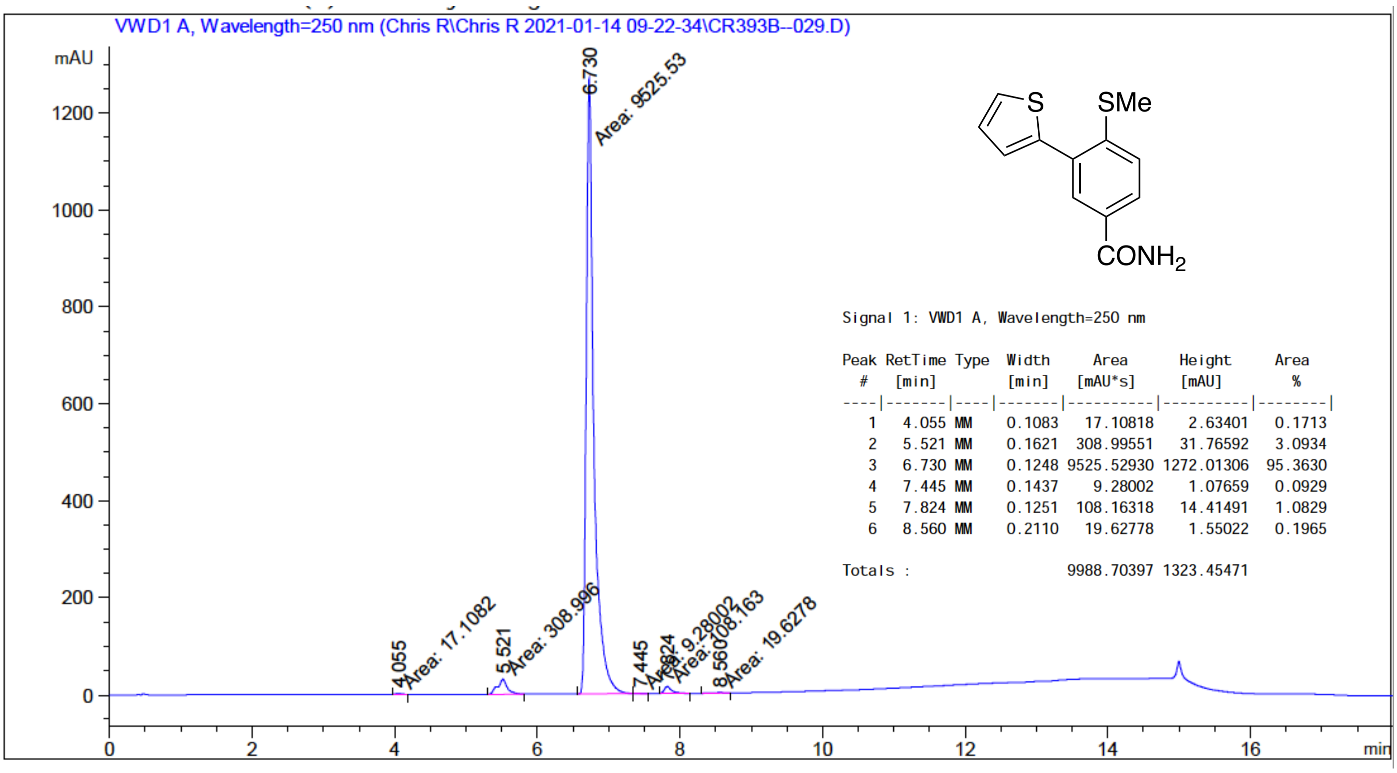

Signal 1: VWD1 A, Wavelength=250 nm

Peak #	RetTime [min]	Type	Width [min]	Area [mAU*s]	Height [mAU]	Area %
1	4.055	MM	0.1083	17.10818	2.63401	0.1713
2	5.521	MM	0.1621	308.99551	31.76592	3.0934
3	6.730	MM	0.1248	9525.52930	1272.01306	95.3630
4	7.445	MM	0.1437	9.28002	1.07659	0.0929
5	7.824	MM	0.1251	108.16318	14.41491	1.0829
6	8.560	MM	0.2110	19.62778	1.55022	0.1965
Totals :				9988.70397	1323.45471	

HPLC Trace of 4-(methylthio)-3-(1*H*-pyrrol-2-yl)benzamide (11)

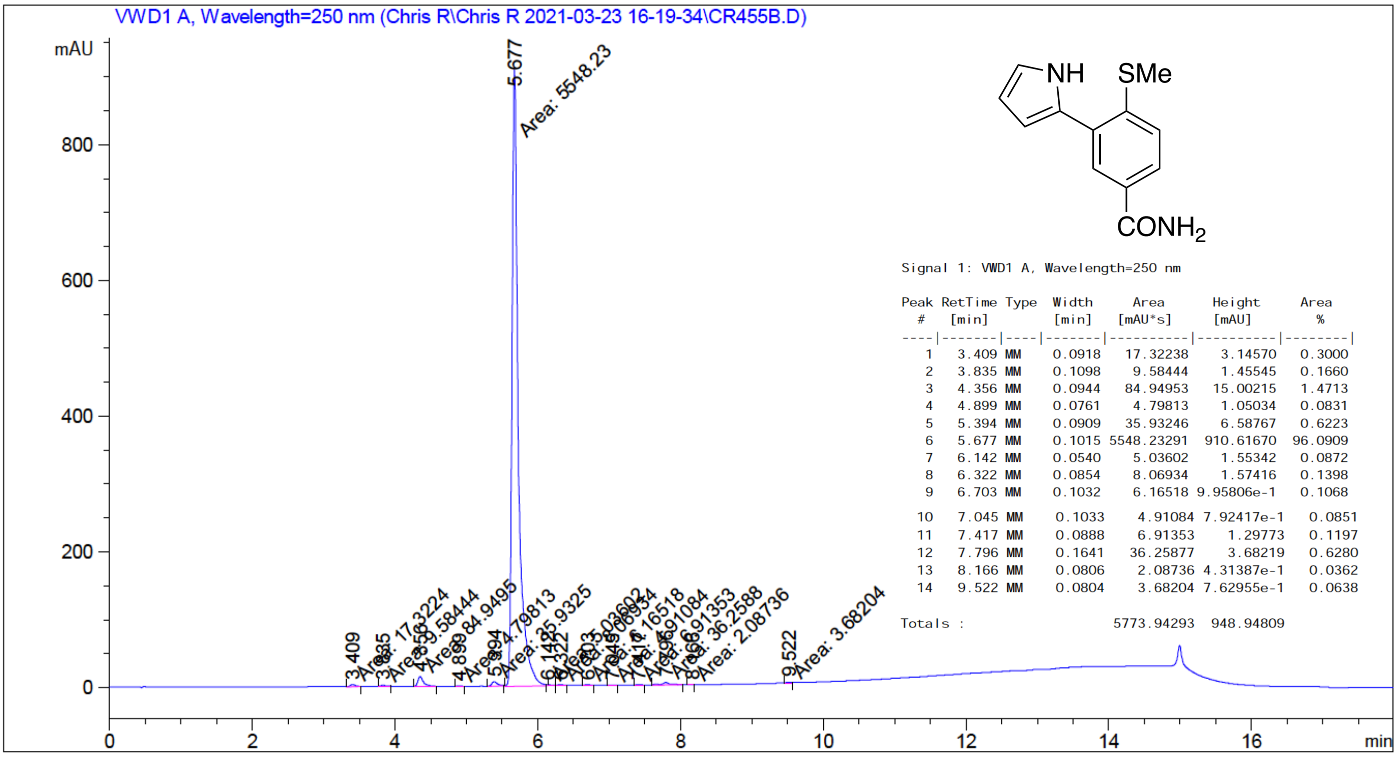

Signal 1: VWD1 A, Wavelength=250 nm

Peak #	RetTime [min]	Type	Width [min]	Area [mAU*s]	Height [mAU]	Area %
1	3.409	MM	0.0918	17.32238	3.14570	0.3000
2	3.835	MM	0.1098	9.58444	1.45545	0.1660
3	4.356	MM	0.0944	84.94953	15.00215	1.4713
4	4.899	MM	0.0761	4.79813	1.05034	0.0831
5	5.394	MM	0.0909	35.93246	6.58767	0.6223
6	5.677	MM	0.1015	5548.23291	910.61670	96.0909
7	6.142	MM	0.0540	5.03602	1.55342	0.0872
8	6.322	MM	0.0854	8.06934	1.57416	0.1398
9	6.703	MM	0.1032	6.16518	9.95806e-1	0.1068
10	7.045	MM	0.1033	4.91084	7.92417e-1	0.0851
11	7.417	MM	0.0888	6.91353	1.29773	0.1197
12	7.796	MM	0.1641	36.25877	3.68219	0.6280
13	8.166	MM	0.0806	2.08736	4.31387e-1	0.0362
14	9.522	MM	0.0804	3.68204	7.62955e-1	0.0638
Totals :				5773.94293	948.94809	

HPLC Trace of 3-(1*H*-indazol-5-yl)-4-(methylthio)benzamide (12)

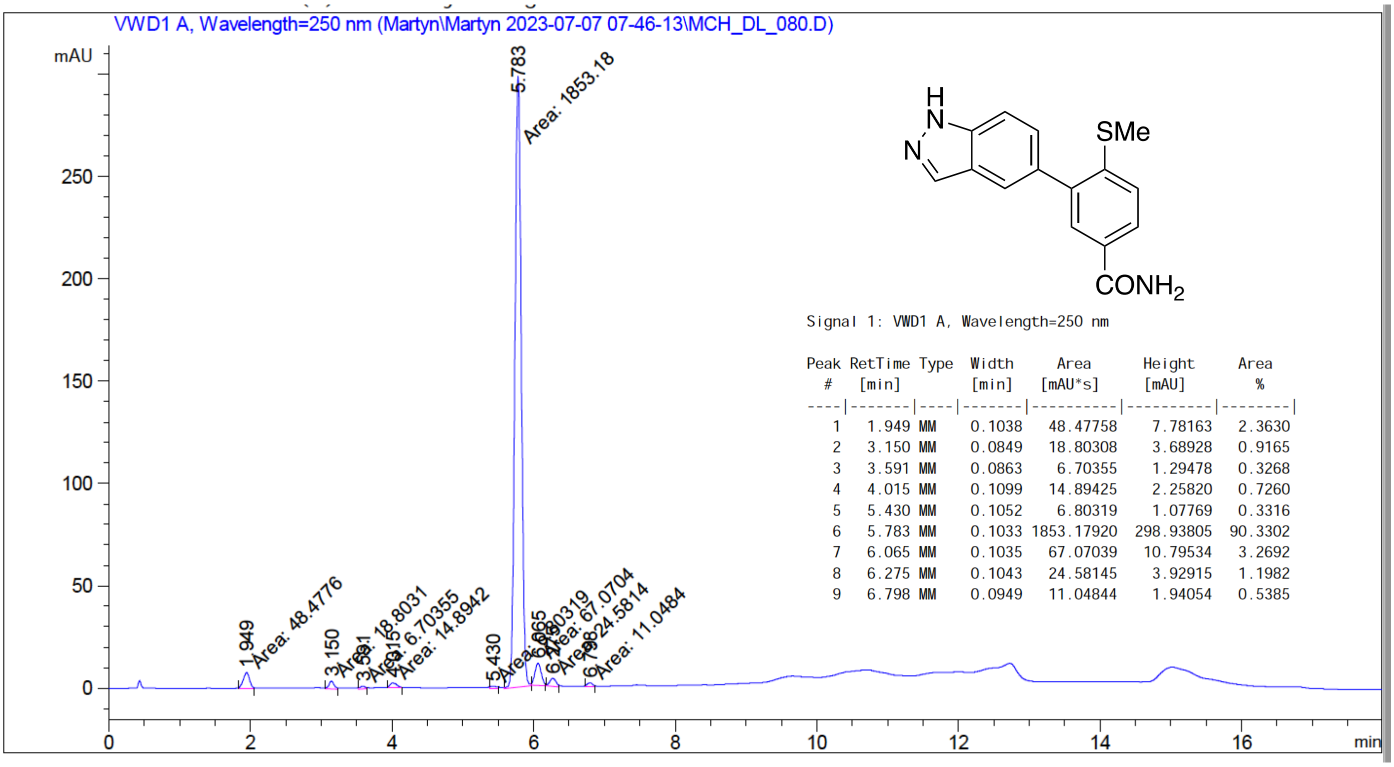

Signal 1: VWD1 A, Wavelength=250 nm

Peak #	RetTime [min]	Type	Width [min]	Area [mAU*s]	Height [mAU]	Area %
1	1.949	MM	0.1038	48.47758	7.78163	2.3630
2	3.150	MM	0.0849	18.80308	3.68928	0.9165
3	3.591	MM	0.0863	6.70355	1.29478	0.3268
4	4.015	MM	0.1099	14.89425	2.25820	0.7260
5	5.430	MM	0.1052	6.80319	1.07769	0.3316
6	5.783	MM	0.1033	1853.17920	298.93805	90.3302
7	6.065	MM	0.1035	67.07039	10.79534	3.2692
8	6.275	MM	0.1043	24.58145	3.92915	1.1982
9	6.798	MM	0.0949	11.04844	1.94054	0.5385

HPLC Trace of 3-(1*H*-indol-5-yl)-4-(methylthio)benzamide (13)

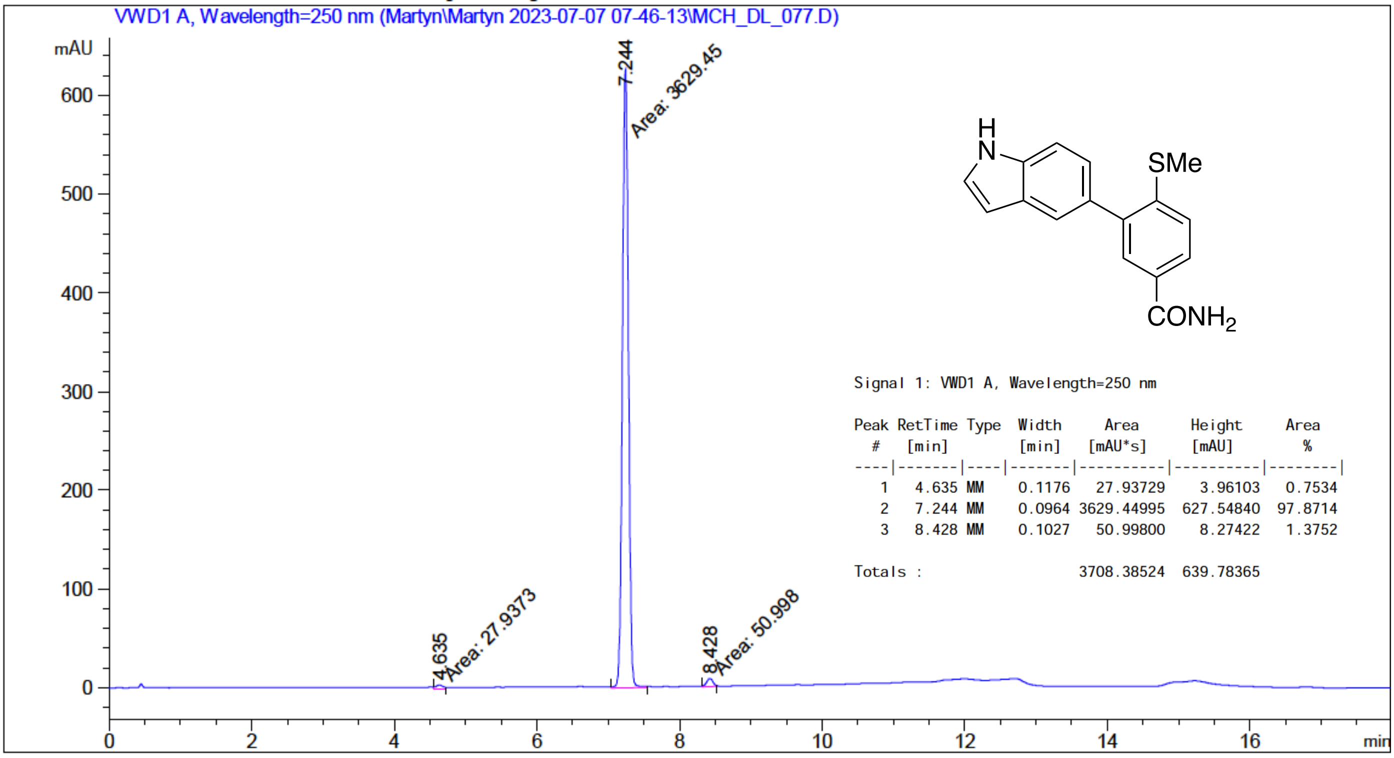

Signal 1: VWD1 A, Wavelength=250 nm

Peak #	RetTime [min]	Type	Width [min]	Area [mAU*s]	Height [mAU]	Area %
1	4.635	MM	0.1176	27.93729	3.96103	0.7534
2	7.244	MM	0.0964	3629.44995	627.54840	97.8714
3	8.428	MM	0.1027	50.99800	8.27422	1.3752
Totals :				3708.38524	639.78365	

HPLC Trace of 3-(1*H*-indazol-6-yl)-4-(methylthio)benzamide (14)

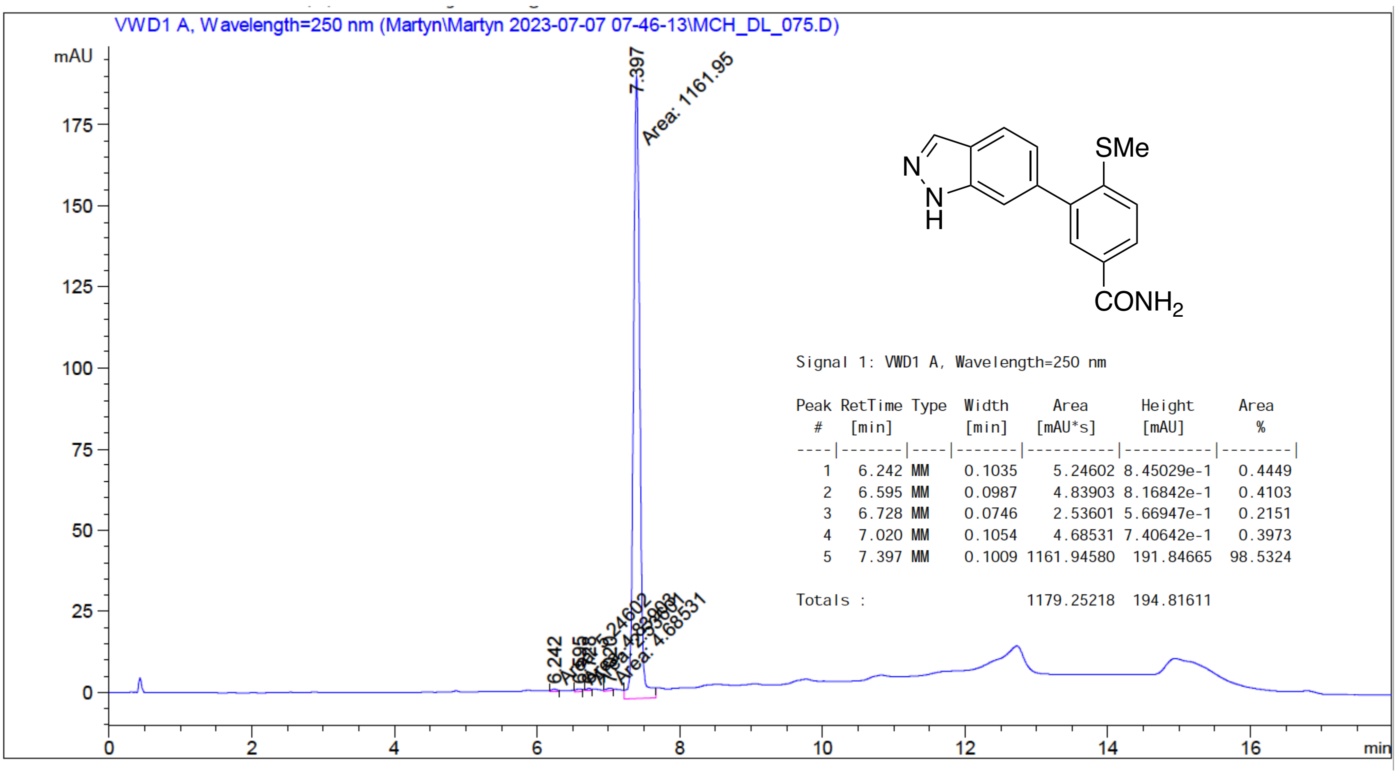

Signal 1: VWD1 A, Wavelength=250 nm

Peak #	RetTime [min]	Type	Width [min]	Area [mAU*s]	Height [mAU]	Area %
1	6.242	MM	0.1035	5.24602	8.45029e-1	0.4449
2	6.595	MM	0.0987	4.83903	8.16842e-1	0.4103
3	6.728	MM	0.0746	2.53601	5.66947e-1	0.2151
4	7.020	MM	0.1054	4.68531	7.40642e-1	0.3973
5	7.397	MM	0.1009	1161.94580	191.84665	98.5324
Totals :				1179.25218	194.81611	

HPLC Trace of 3-(1*H*-benzo[*d*]imidazol-5-yl)-4-(methylthio)benzamide (15)

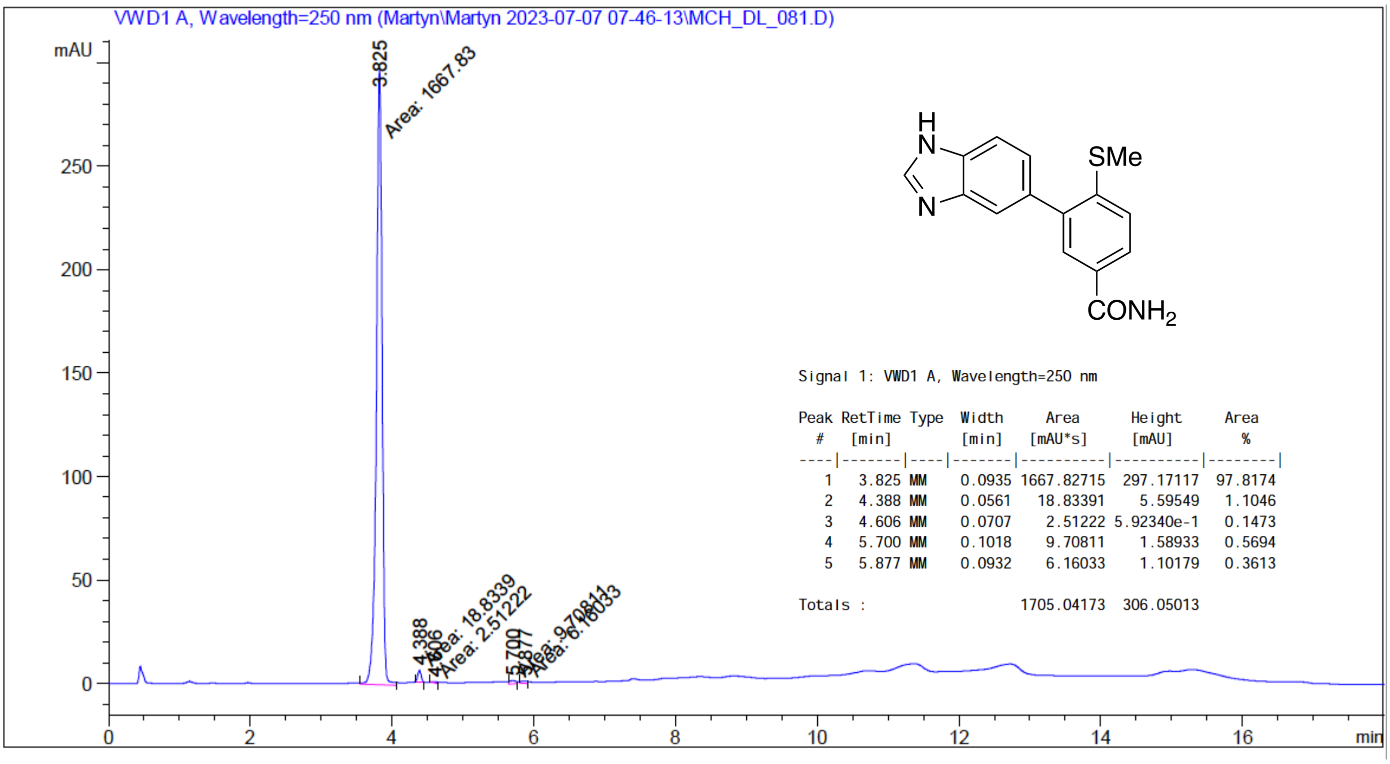

Signal 1: VWD1 A, Wavelength=250 nm

Peak #	RetTime [min]	Type	Width [min]	Area [mAU*s]	Height [mAU]	Area %
1	3.825	MM	0.0935	1667.82715	297.17117	97.8174
2	4.388	MM	0.0561	18.83391	5.59549	1.1046
3	4.606	MM	0.0707	2.51222	5.92340e-1	0.1473
4	5.700	MM	0.1018	9.70811	1.58933	0.5694
5	5.877	MM	0.0932	6.16033	1.10179	0.3613
Totals :				1705.04173	306.05013	

HPLC Trace of 3-(1-methyl-1*H*-indazol-5-yl)-4-(methylthio)benzamide (16)

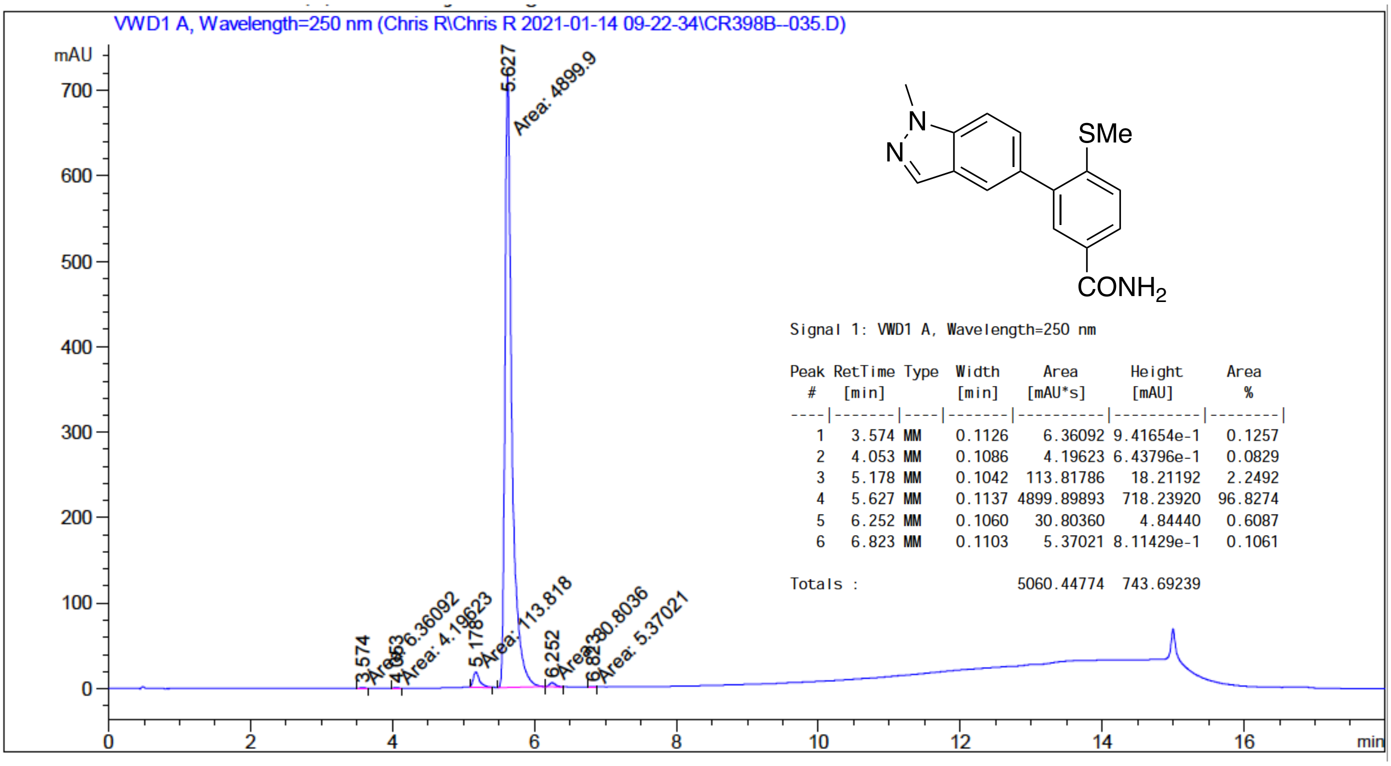

Signal 1: VWD1 A, Wavelength=250 nm

Peak #	RetTime [min]	Type	Width [min]	Area [mAU*s]	Height [mAU]	Area %
1	3.574	MM	0.1126	6.36092	9.41654e-1	0.1257
2	4.053	MM	0.1086	4.19623	6.43796e-1	0.0829
3	5.178	MM	0.1042	113.81786	18.21192	2.2492
4	5.627	MM	0.1137	4899.89893	718.23920	96.8274
5	6.252	MM	0.1060	30.80360	4.84440	0.6087
6	6.823	MM	0.1103	5.37021	8.11429e-1	0.1061

Totals : 5060.44774 743.69239

HPLC Trace of 3-(1-methyl-1*H*-indol-5-yl)-4-(methylthio)benzamide (17)

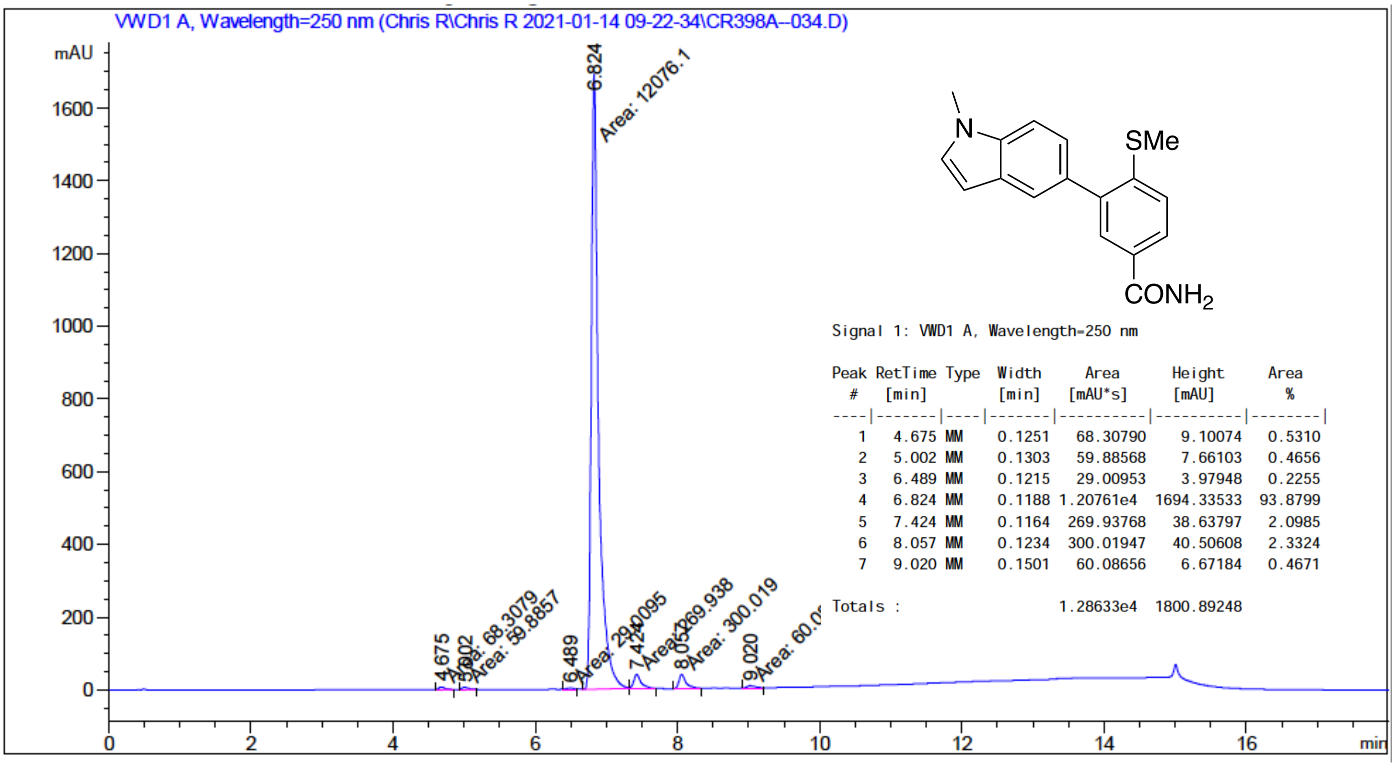

Signal 1: VWD1 A, Wavelength=250 nm

Peak #	RetTime [min]	Type	Width [min]	Area [mAU*s]	Height [mAU]	Area %
1	4.675	MM	0.1251	68.30790	9.10074	0.5310
2	5.002	MM	0.1303	59.88568	7.66103	0.4656
3	6.489	MM	0.1215	29.00953	3.97948	0.2255
4	6.824	MM	0.1188	1.20761e4	1694.33533	93.8799
5	7.424	MM	0.1164	269.93768	38.63797	2.0985
6	8.057	MM	0.1234	300.01947	40.50608	2.3324
7	9.020	MM	0.1501	60.08656	6.67184	0.4671

Totals : 1.28633e4 1800.89248

HPLC Trace of 3-(imidazo[1,2-*a*]pyridin-6-yl)-4-(methylthio)benzamide (18)

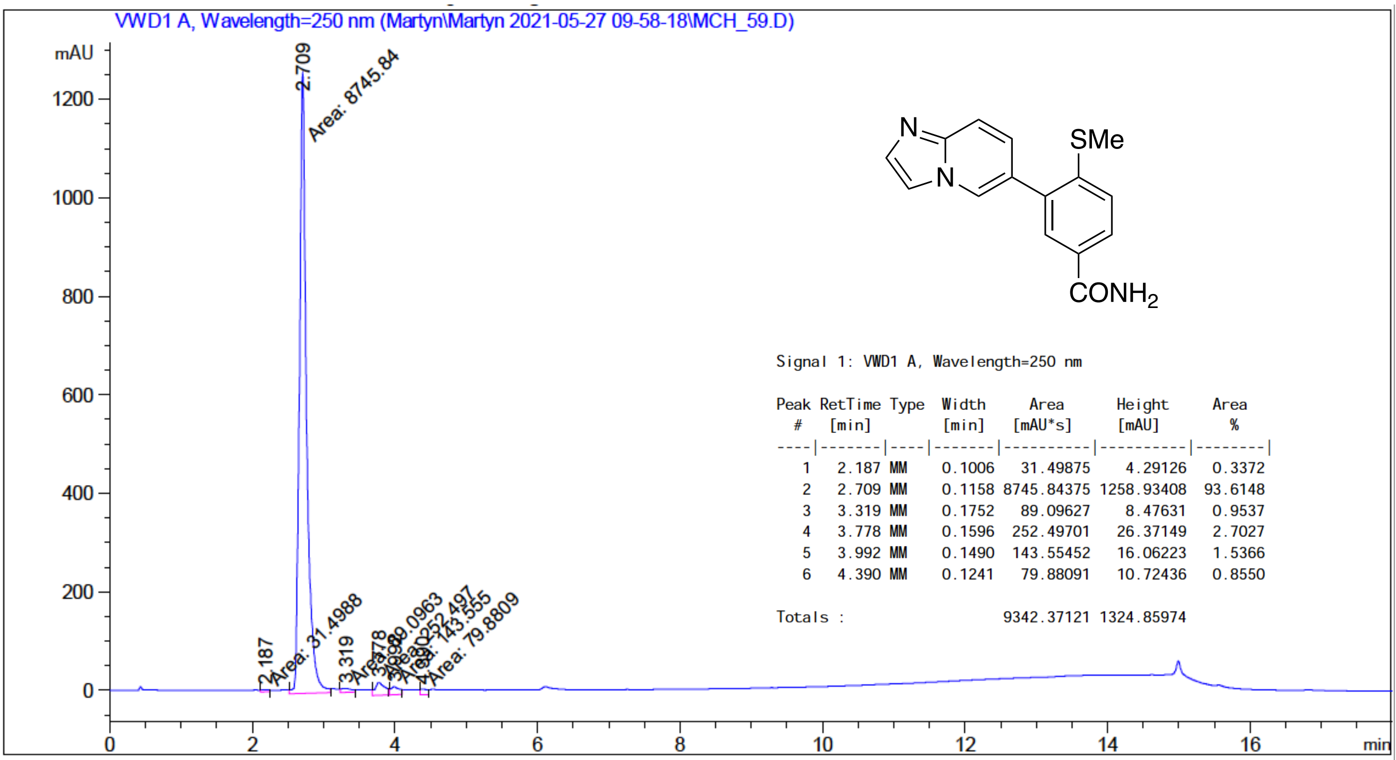

Signal 1: VWD1 A, Wavelength=250 nm

Peak #	RetTime [min]	Type	Width [min]	Area [mAU*s]	Height [mAU]	Area %
1	2.187	MM	0.1006	31.49875	4.29126	0.3372
2	2.709	MM	0.1158	8745.84375	1258.93408	93.6148
3	3.319	MM	0.1752	89.09627	8.47631	0.9537
4	3.778	MM	0.1596	252.49701	26.37149	2.7027
5	3.992	MM	0.1490	143.55452	16.06223	1.5366
6	4.390	MM	0.1241	79.88091	10.72436	0.8550

Totals : 9342.37121 1324.85974

HPLC Trace of 4-(methylthio)-3-(2-oxoindolin-4-yl)benzamide (19)

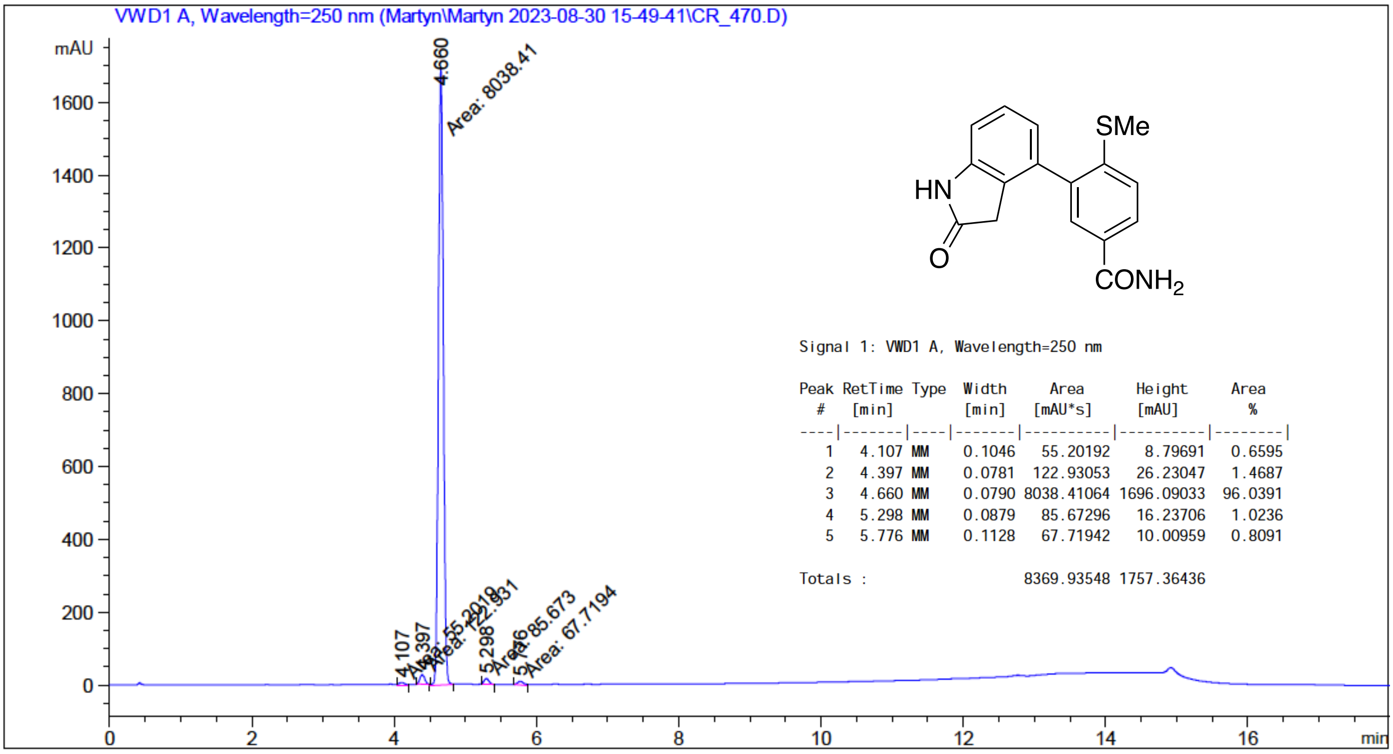

Signal 1: VWD1 A, Wavelength=250 nm

Peak #	RetTime [min]	Type	Width [min]	Area [mAU*s]	Height [mAU]	Area %
1	4.107	MM	0.1046	55.20192	8.79691	0.6595
2	4.397	MM	0.0781	122.93053	26.23047	1.4687
3	4.660	MM	0.0790	8038.41064	1696.09033	96.0391
4	5.298	MM	0.0879	85.67296	16.23706	1.0236
5	5.776	MM	0.1128	67.71942	10.00959	0.8091

Totals : 8369.93548 1757.36436

HPLC Trace of 4-(methylthio)-3-(quinolin-6-yl)benzamide (20)

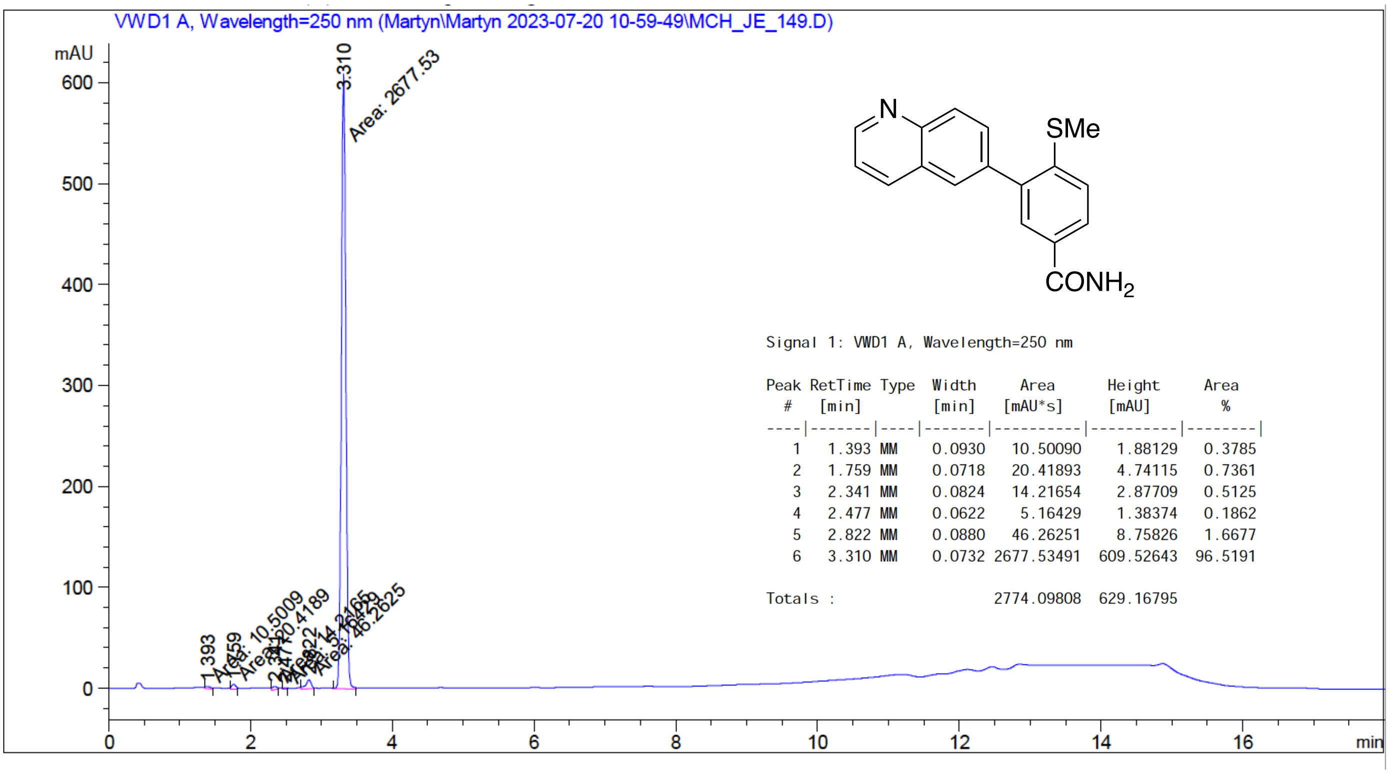

Signal 1: VWD1 A, Wavelength=250 nm

Peak #	RetTime [min]	Type	Width [min]	Area [mAU*s]	Height [mAU]	Area %
1	1.393	MM	0.0930	10.50090	1.88129	0.3785
2	1.759	MM	0.0718	20.41893	4.74115	0.7361
3	2.341	MM	0.0824	14.21654	2.87709	0.5125
4	2.477	MM	0.0622	5.16429	1.38374	0.1862
5	2.822	MM	0.0880	46.26251	8.75826	1.6677
6	3.310	MM	0.0732	2677.53491	609.52643	96.5191

Totals : 2774.09808 629.16795

HPLC Trace of 4-(methylthio)-3-(pyridin-3-yl)benzamide (22)

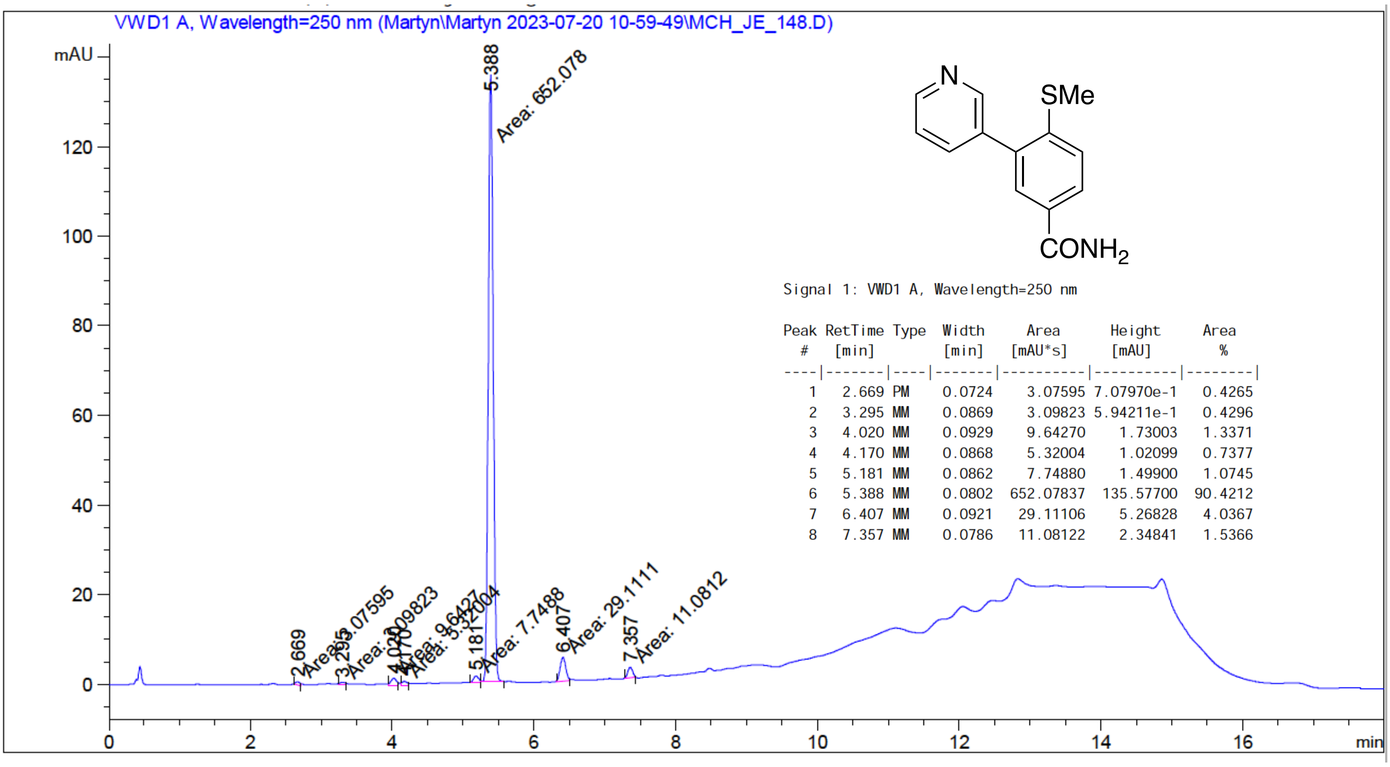

Signal 1: VWD1 A, Wavelength=250 nm

Peak #	RetTime [min]	Type	Width [min]	Area [mAU*s]	Height [mAU]	Area %
1	2.669	PM	0.0724	3.07595	7.07970e-1	0.4265
2	3.295	MM	0.0869	3.09823	5.94211e-1	0.4296
3	4.020	MM	0.0929	9.64270	1.73003	1.3371
4	4.170	MM	0.0868	5.32004	1.02099	0.7377
5	5.181	MM	0.0862	7.74880	1.49900	1.0745
6	5.388	MM	0.0802	652.07837	135.57700	90.4212
7	6.407	MM	0.0921	29.11106	5.26828	4.0367
8	7.357	MM	0.0786	11.08122	2.34841	1.5366

HPLC Trace of 4-(methylthio)-3-(pyrimidin-5-yl)benzamide (24)

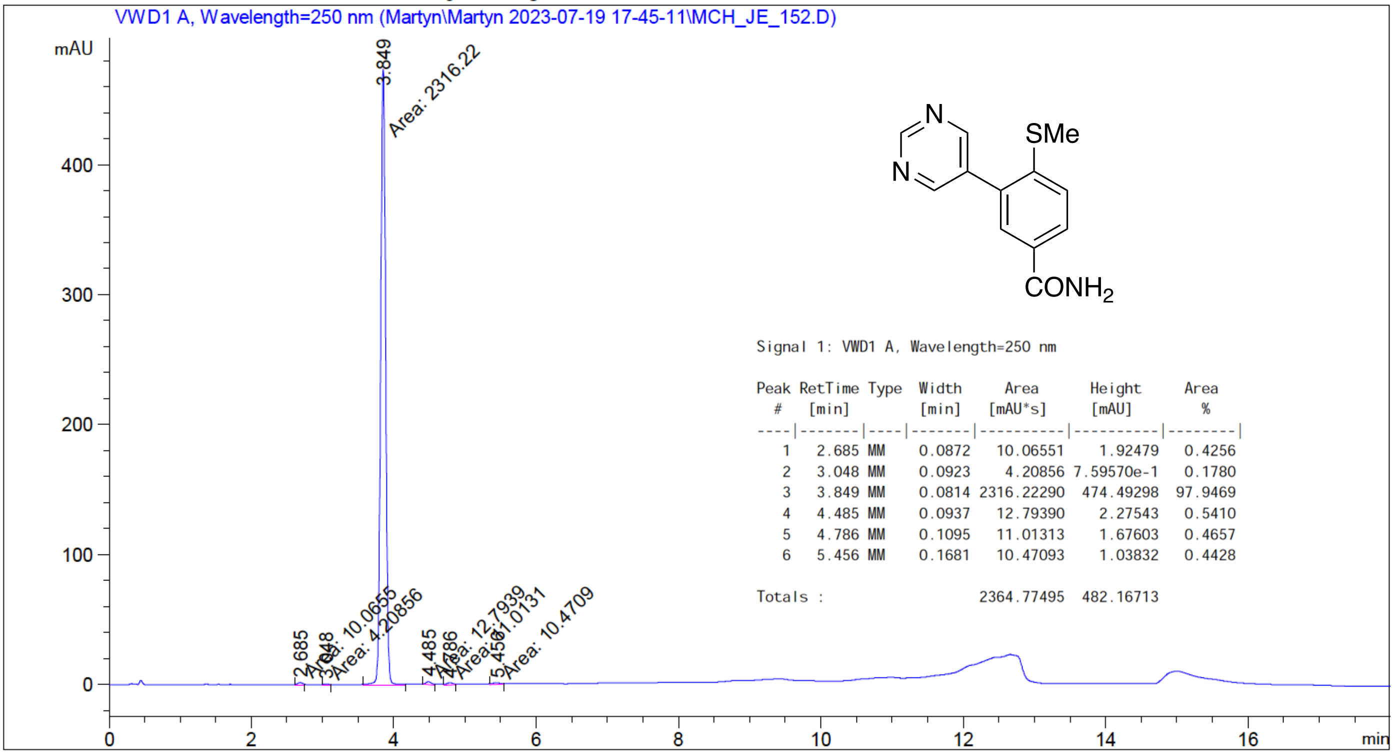

Signal 1: VWD1 A, Wavelength=250 nm

Peak #	RetTime [min]	Type	Width [min]	Area [mAU*s]	Height [mAU]	Area %
1	2.685	MM	0.0872	10.06551	1.92479	0.4256
2	3.048	MM	0.0923	4.20856	7.59570e-1	0.1780
3	3.849	MM	0.0814	2316.22290	474.49298	97.9469
4	4.485	MM	0.0937	12.79390	2.27543	0.5410
5	4.786	MM	0.1095	11.01313	1.67603	0.4657
6	5.456	MM	0.1681	10.47093	1.03832	0.4428

Totals : 2364.77495 482.16713

HPLC Trace of 3-(benzofuran-5′-yl)-4-(methylthio)benzamide (25)

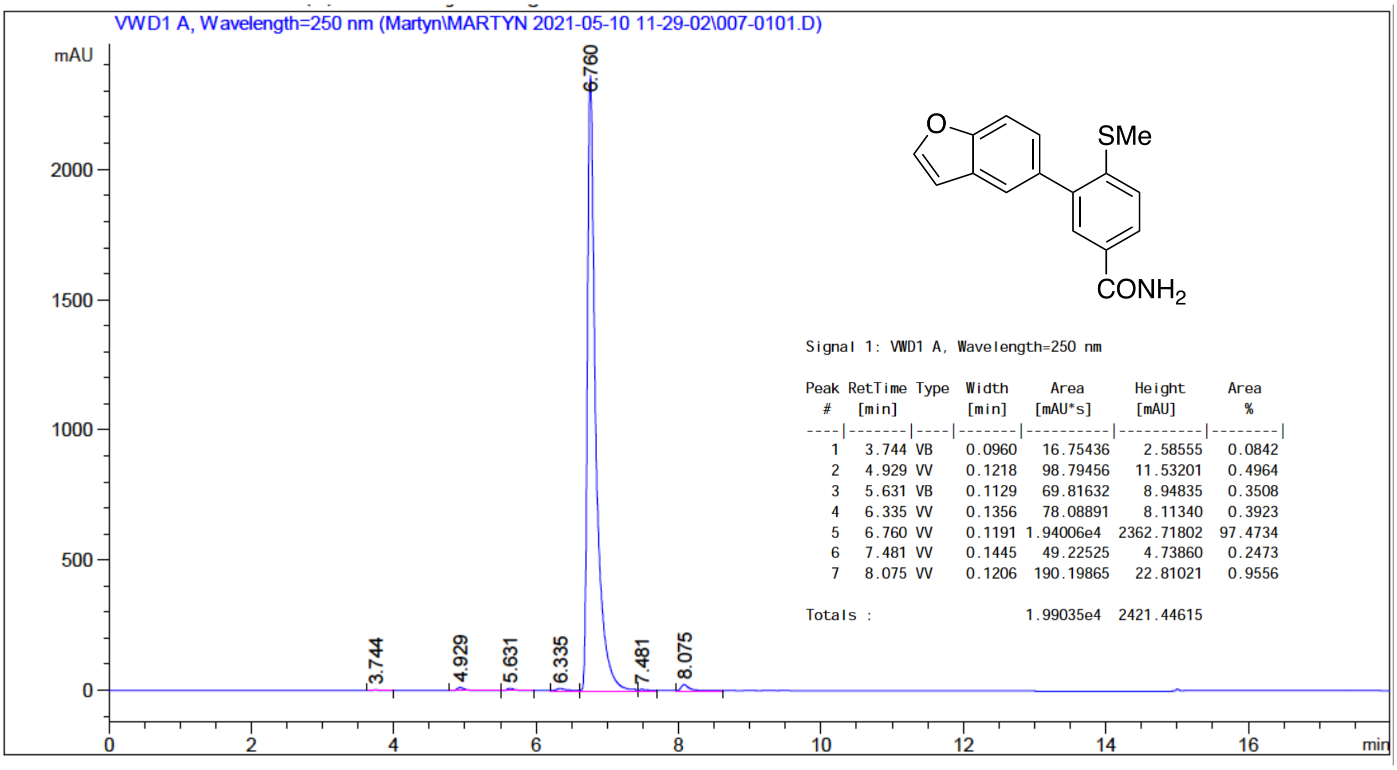

Signal 1: VWD1 A, Wavelength=250 nm

Peak #	RetTime [min]	Type	Width [min]	Area [mAU*s]	Height [mAU]	Area %
1	3.744	VB	0.0960	16.75436	2.58555	0.0842
2	4.929	VV	0.1218	98.79456	11.53201	0.4964
3	5.631	VB	0.1129	69.81632	8.94835	0.3508
4	6.335	VV	0.1356	78.08891	8.11340	0.3923
5	6.760	VV	0.1191	1.94006e4	2362.71802	97.4734
6	7.481	VV	0.1445	49.22525	4.73860	0.2473
7	8.075	VV	0.1206	190.19865	22.81021	0.9556

Totals : 1.99035e4 2421.44615

HPLC Trace of 3-(benzoxazole)-4-(methylthio)benzamide (26)

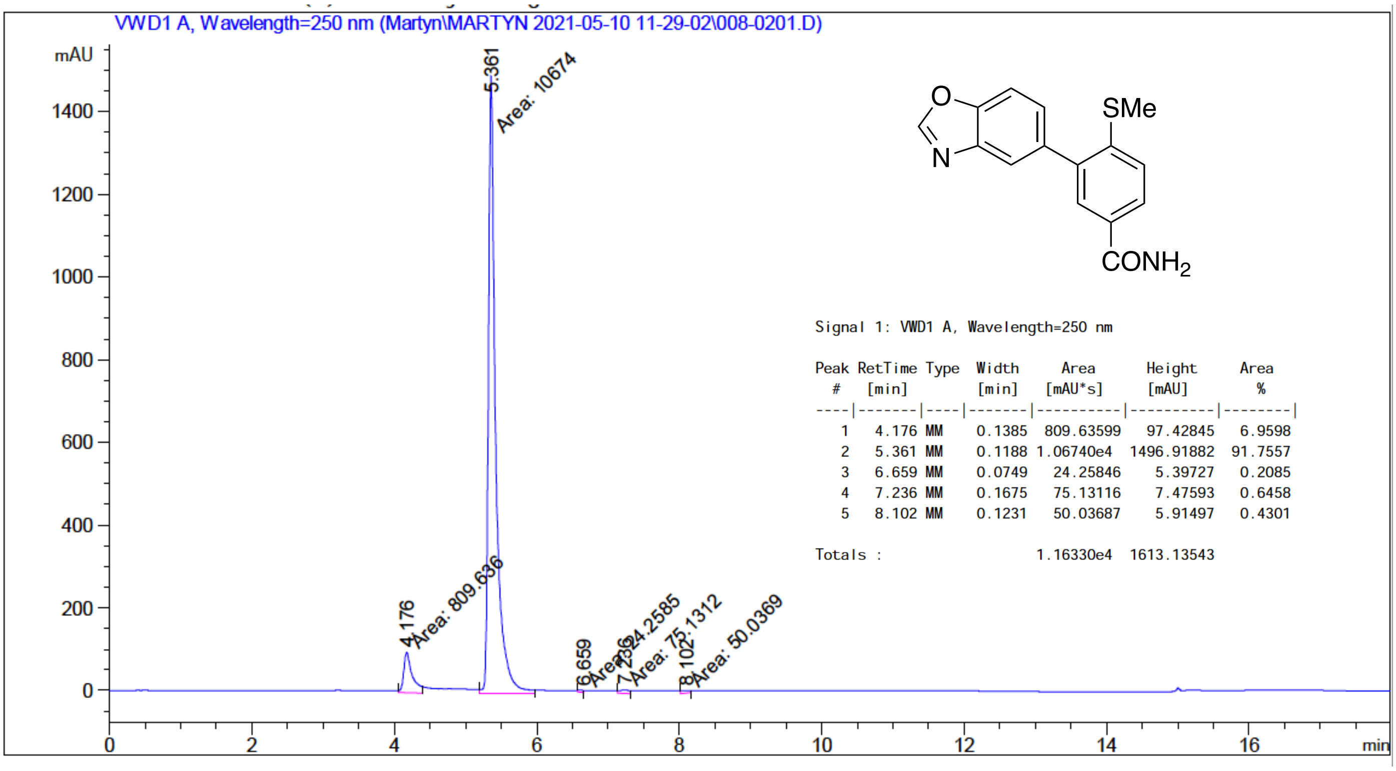

Signal 1: VWD1 A, Wavelength=250 nm

Peak #	RetTime [min]	Type	Width [min]	Area [mAU*s]	Height [mAU]	Area %
1	4.176	MM	0.1385	809.63599	97.42845	6.9598
2	5.361	MM	0.1188	1.06740e4	1496.91882	91.7557
3	6.659	MM	0.0749	24.25846	5.39727	0.2085
4	7.236	MM	0.1675	75.13116	7.47593	0.6458
5	8.102	MM	0.1231	50.03687	5.91497	0.4301

Totals : 1.16330e4 1613.13543

HPLC Trace of 3-(2,3-dihydrobenzofuran-5-yl)-4-(methylthio)benzamide (27)

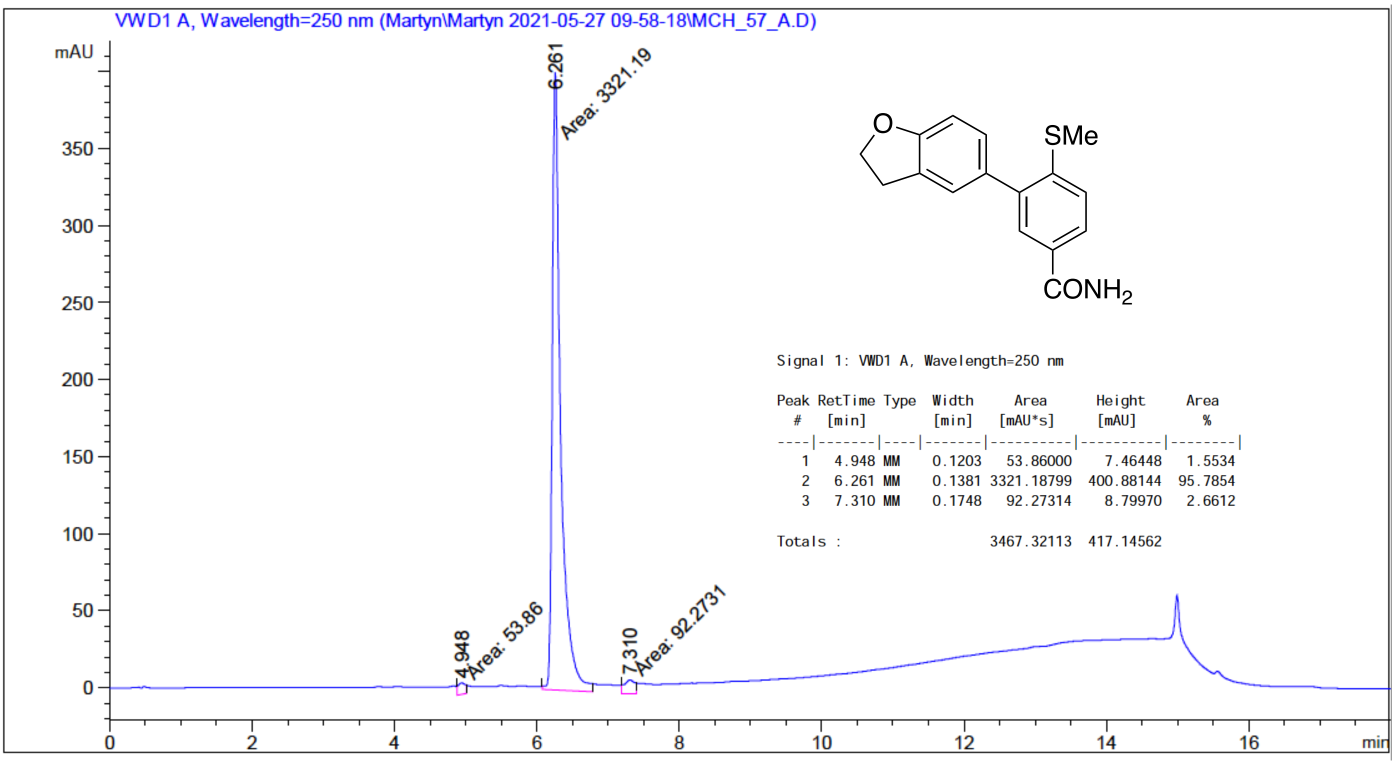

Signal 1: VWD1 A, Wavelength=250 nm

Peak #	RetTime [min]	Type	Width [min]	Area [mAU*s]	Height [mAU]	Area %
1	4.948	MM	0.1203	53.86000	7.46448	1.5534
2	6.261	MM	0.1381	3321.18799	400.88144	95.7854
3	7.310	MM	0.1748	92.27314	8.79970	2.6612

Totals : 3467.32113 417.14562

HPLC Trace of 3-(3′,4′-dimethoxyphenyl)-4-(methylthio)benzamide (28)

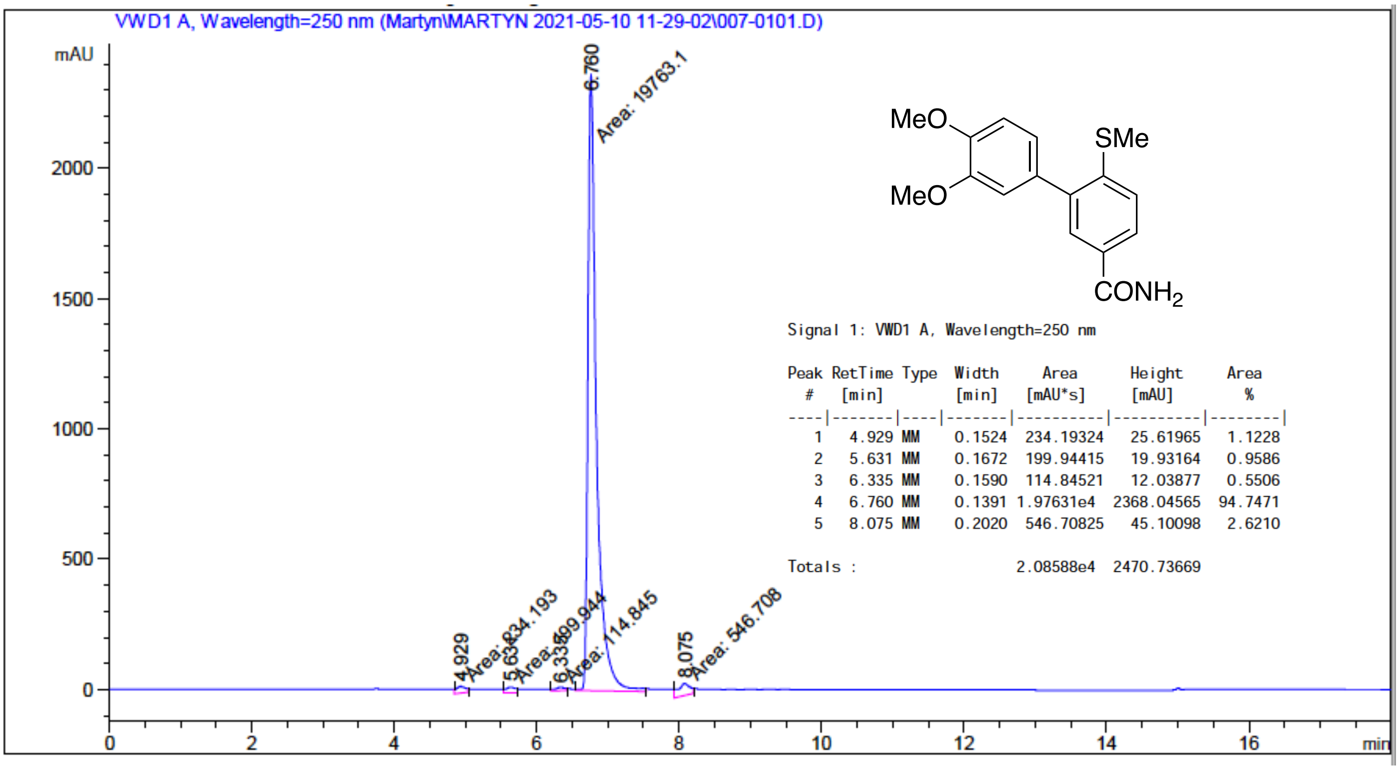

Signal 1: VWD1 A, Wavelength=250 nm

Peak #	RetTime [min]	Type	Width [min]	Area [mAU*s]	Height [mAU]	Area %
1	4.929	MM	0.1524	234.19324	25.61965	1.1228
2	5.631	MM	0.1672	199.94415	19.93164	0.9586
3	6.335	MM	0.1590	114.84521	12.03877	0.5506
4	6.760	MM	0.1391	1.97631e4	2368.04565	94.7471
5	8.075	MM	0.2020	546.70825	45.10098	2.6210

Totals : 2.08588e4 2470.73669

HPLC Trace of 4'-amino-3'-methoxy-6-(methylthio)-[1,1'-biphenyl]-3-carboxamide (30)

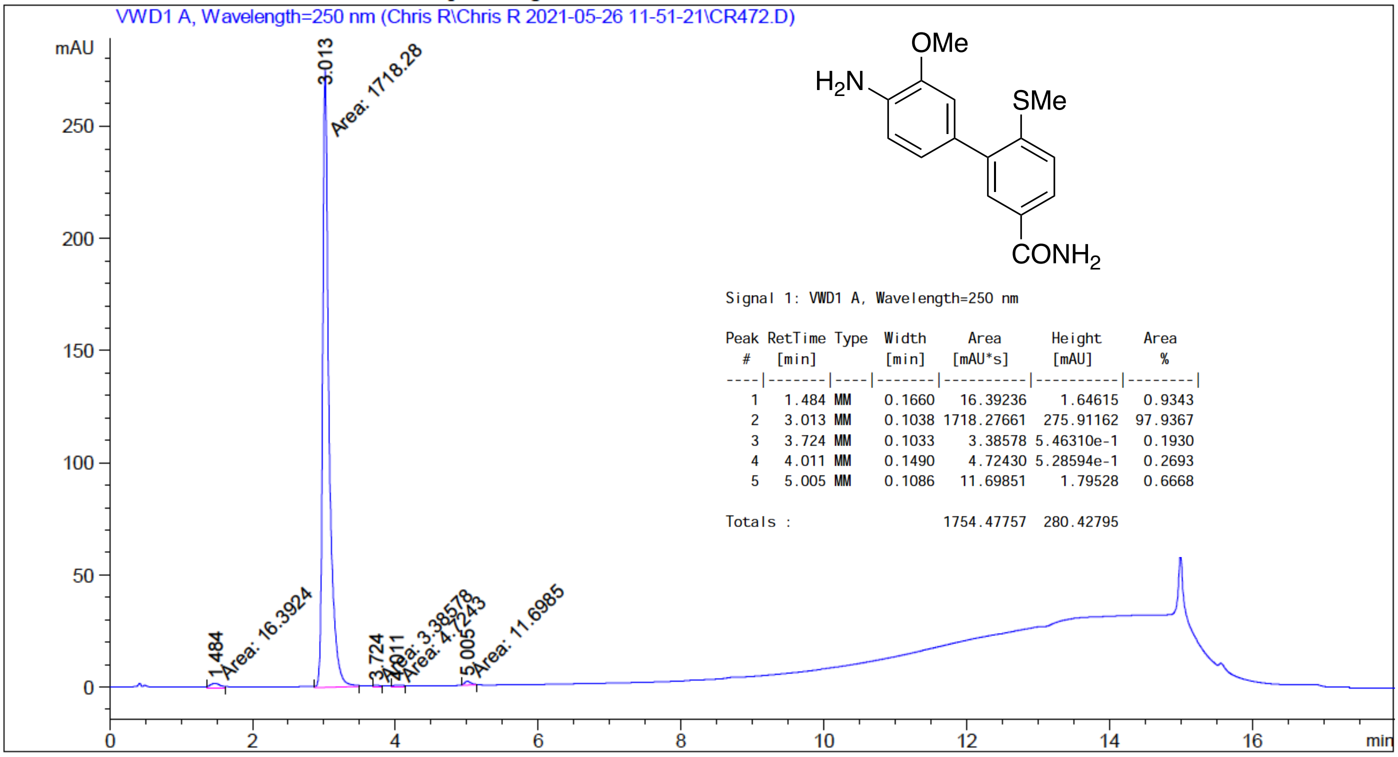

Signal 1: VWD1 A, Wavelength=250 nm

Peak #	RetTime [min]	Type	Width [min]	Area [mAU*s]	Height [mAU]	Area %
1	1.484	MM	0.1660	16.39236	1.64615	0.9343
2	3.013	MM	0.1038	1718.27661	275.91162	97.9367
3	3.724	MM	0.1033	3.38578	5.46310e-1	0.1930
4	4.011	MM	0.1490	4.72430	5.28594e-1	0.2693
5	5.005	MM	0.1086	11.69851	1.79528	0.6668
Totals :				1754.47757	280.42795	

HPLC Trace of 2'-fluoro-3'-hydroxy-6-(methylthio)-[1,1'-biphenyl]-3-carboxamide (31)

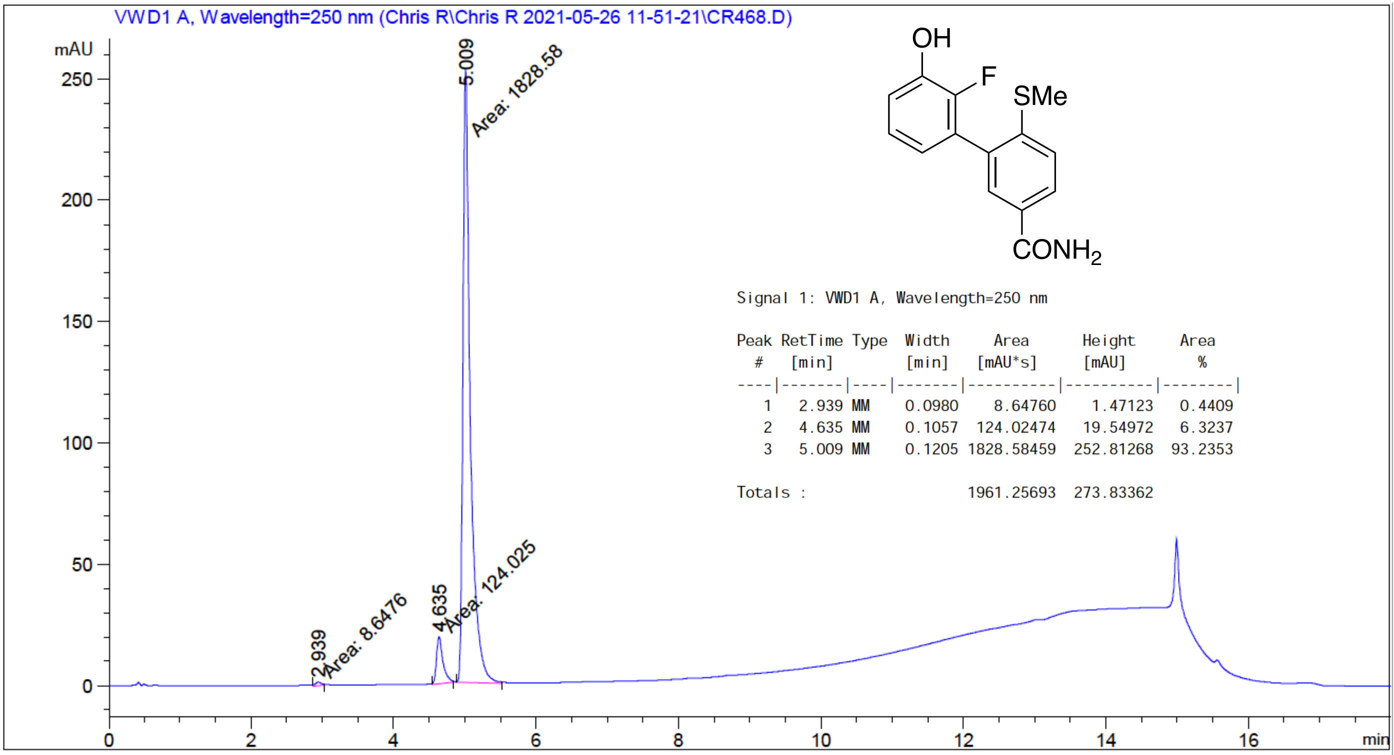

Signal 1: VWD1 A, Wavelength=250 nm

Peak #	RetTime [min]	Type	Width [min]	Area [mAU*s]	Height [mAU]	Area %
1	2.939	MM	0.0980	8.64760	1.47123	0.4409
2	4.635	MM	0.1057	124.02474	19.54972	6.3237
3	5.009	MM	0.1205	1828.58459	252.81268	93.2353
Totals :				1961.25693	273.83362	

HPLC Trace of 3'-fluoro-5'-hydroxy-6-(methylthio)-[1,1'-biphenyl]-3-carboxamide (32)

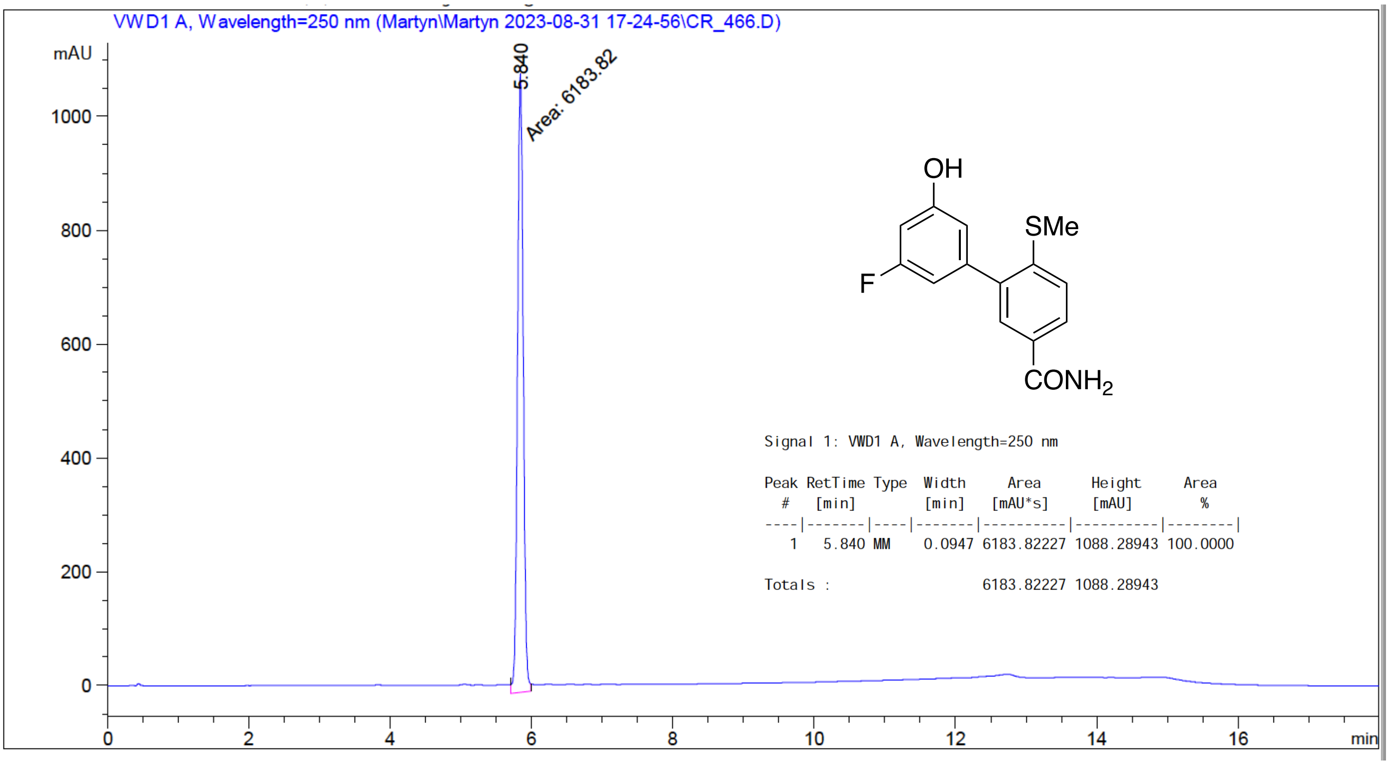

Signal 1: VWD1 A, Wavelength=250 nm

Peak #	RetTime [min]	Type	Width [min]	Area [mAU*s]	Height [mAU]	Area %
1	5.840	MM	0.0947	6183.82227	1088.28943	100.0000

Totals : 6183.82227 1088.28943

HPLC Trace of 4'-fluoro-3'-hydroxy-6-(methylthio)-[1,1'-biphenyl]-3-carboxamide (33)

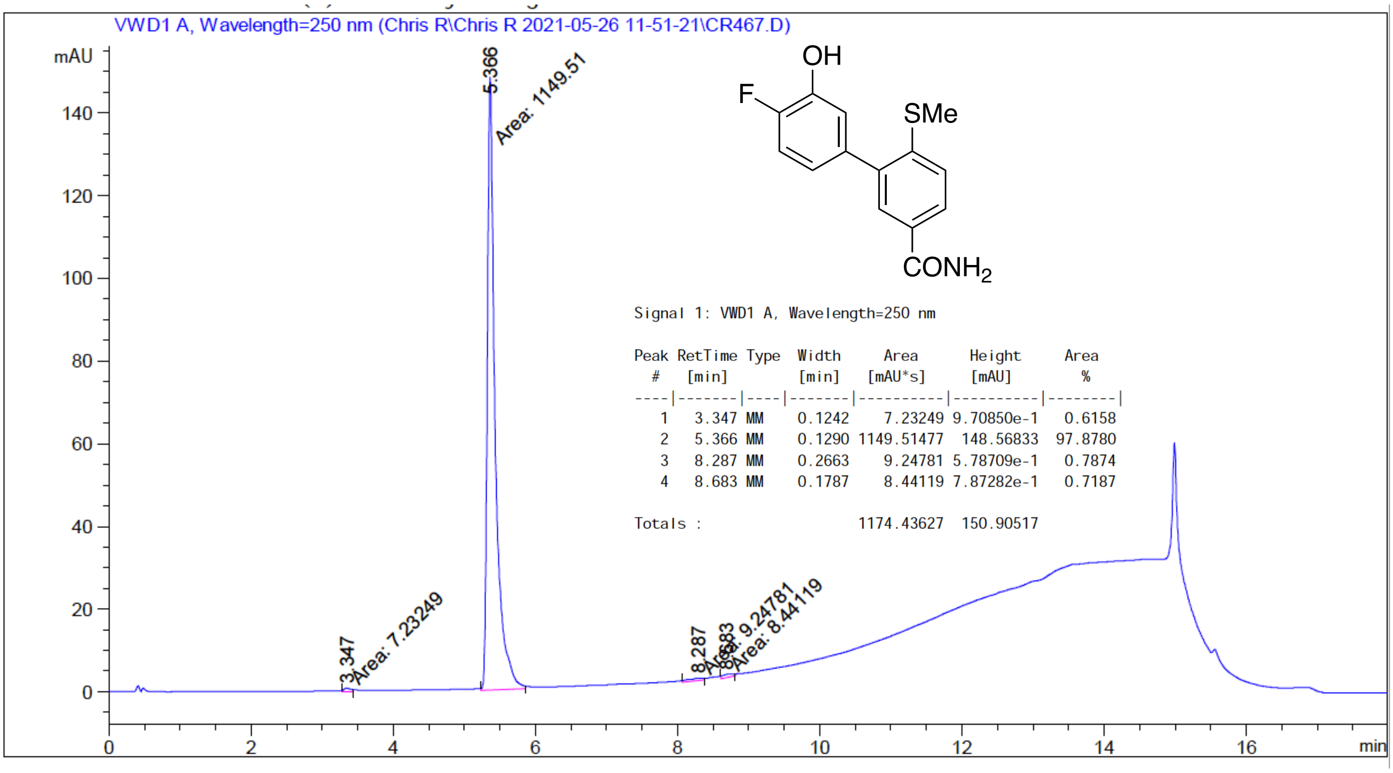

Signal 1: VWD1 A, Wavelength=250 nm

Peak #	RetTime [min]	Type	Width [min]	Area [mAU*s]	Height [mAU]	Area %
1	3.347	MM	0.1242	7.23249	9.70850e-1	0.6158
2	5.366	MM	0.1290	1149.51477	148.56833	97.8780
3	8.287	MM	0.2663	9.24781	5.78709e-1	0.7874
4	8.683	MM	0.1787	8.44119	7.87282e-1	0.7187

Totals : 1174.43627 150.90517

HPLC Trace of 5-(1-methyl-1*H*-indazol-5-yl)-6-(methylthio)nicotinamide (34)

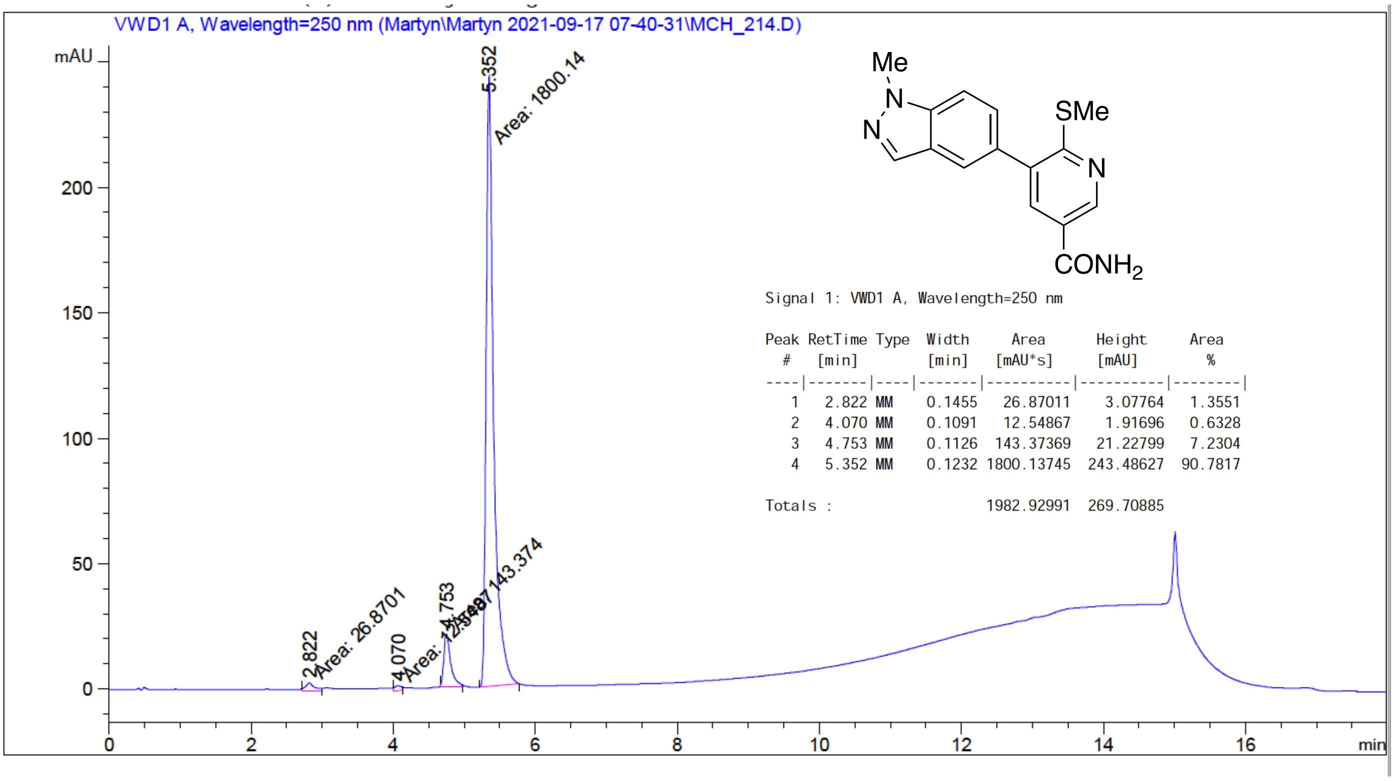

Signal 1: VWD1 A, Wavelength=250 nm

Peak #	RetTime [min]	Type	Width [min]	Area [mAU*s]	Height [mAU]	Area %
1	2.822	MM	0.1455	26.87011	3.07764	1.3551
2	4.070	MM	0.1091	12.54867	1.91696	0.6328
3	4.753	MM	0.1126	143.37369	21.22799	7.2304
4	5.352	MM	0.1232	1800.13745	243.48627	90.7817
Totals :				1982.92991	269.70885	

HPLC Trace of 6-(benzylthio)-5-(1-methyl-1*H*-indazol-5-yl)nicotinamide (35)

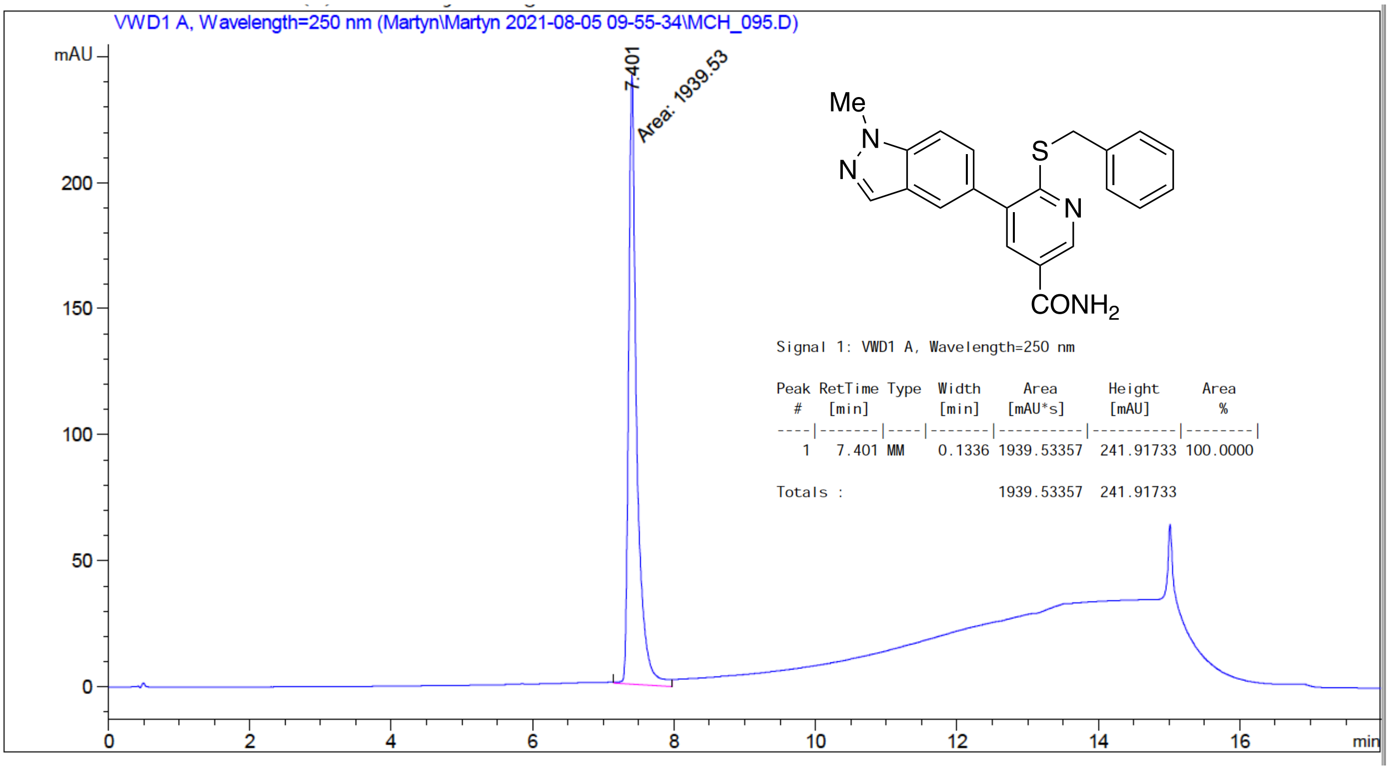

Signal 1: VWD1 A, Wavelength=250 nm

Peak #	RetTime [min]	Type	Width [min]	Area [mAU*s]	Height [mAU]	Area %
1	7.401	MM	0.1336	1939.53357	241.91733	100.0000
Totals :				1939.53357	241.91733	

HPLC Trace of 5-(1-methyl-1*H*-indazol-5-yl)nicotinamide (36)

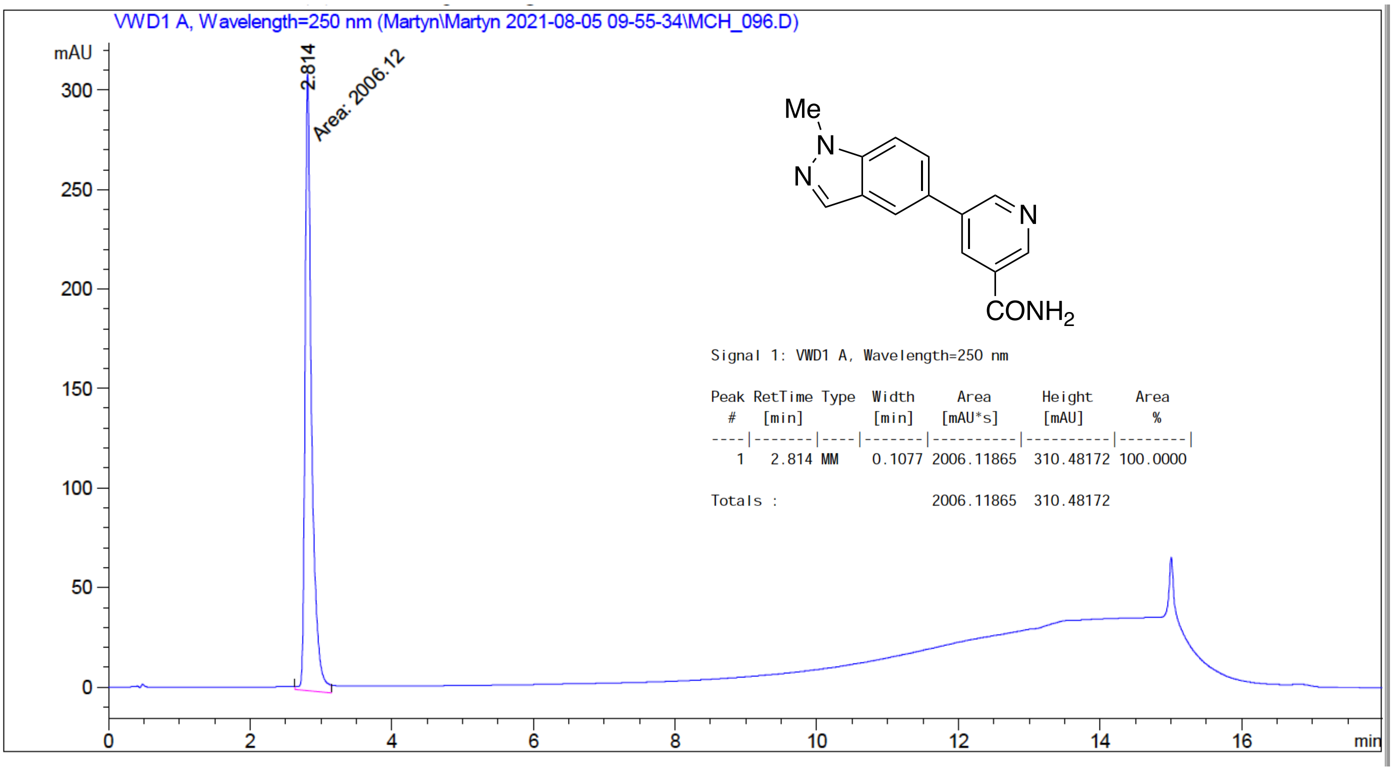

Signal 1: VWD1 A, Wavelength=250 nm

Peak #	RetTime [min]	Type	Width [min]	Area [mAU*s]	Height [mAU]	Area %
1	2.814	MM	0.1077	2006.11865	310.48172	100.0000
Totals :				2006.11865	310.48172	

HPLC Trace of 6-methoxy-5-(1-methyl-1*H*-indazol-5-yl)nicotinamide (37)

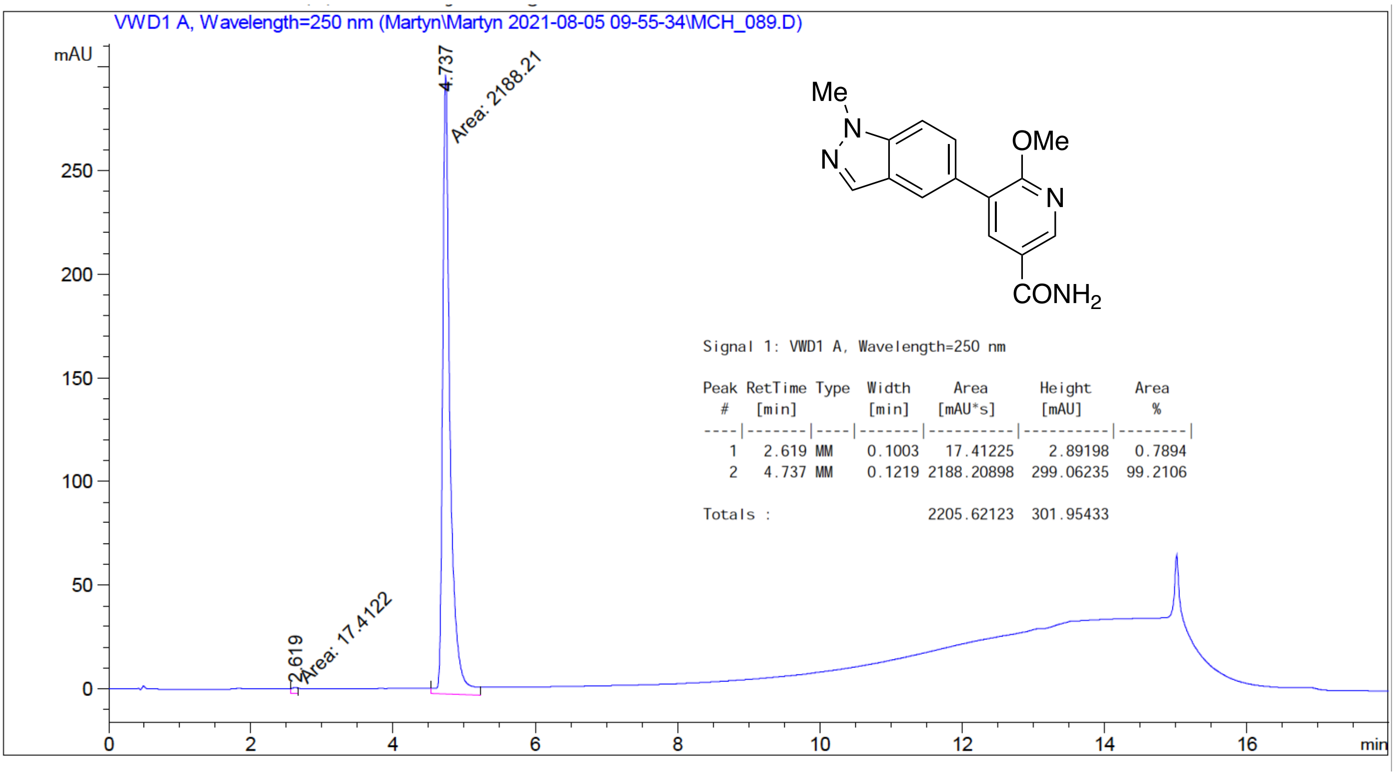

Signal 1: VWD1 A, Wavelength=250 nm

Peak #	RetTime [min]	Type	Width [min]	Area [mAU*s]	Height [mAU]	Area %
1	2.619	MM	0.1003	17.41225	2.89198	0.7894
2	4.737	MM	0.1219	2188.20898	299.06235	99.2106
Totals :				2205.62123	301.95433	

HPLC Trace of 3-fluoro-5-(1-methyl-1*H*-indazol-5-yl)-4-(methylthio)benzamide (38)

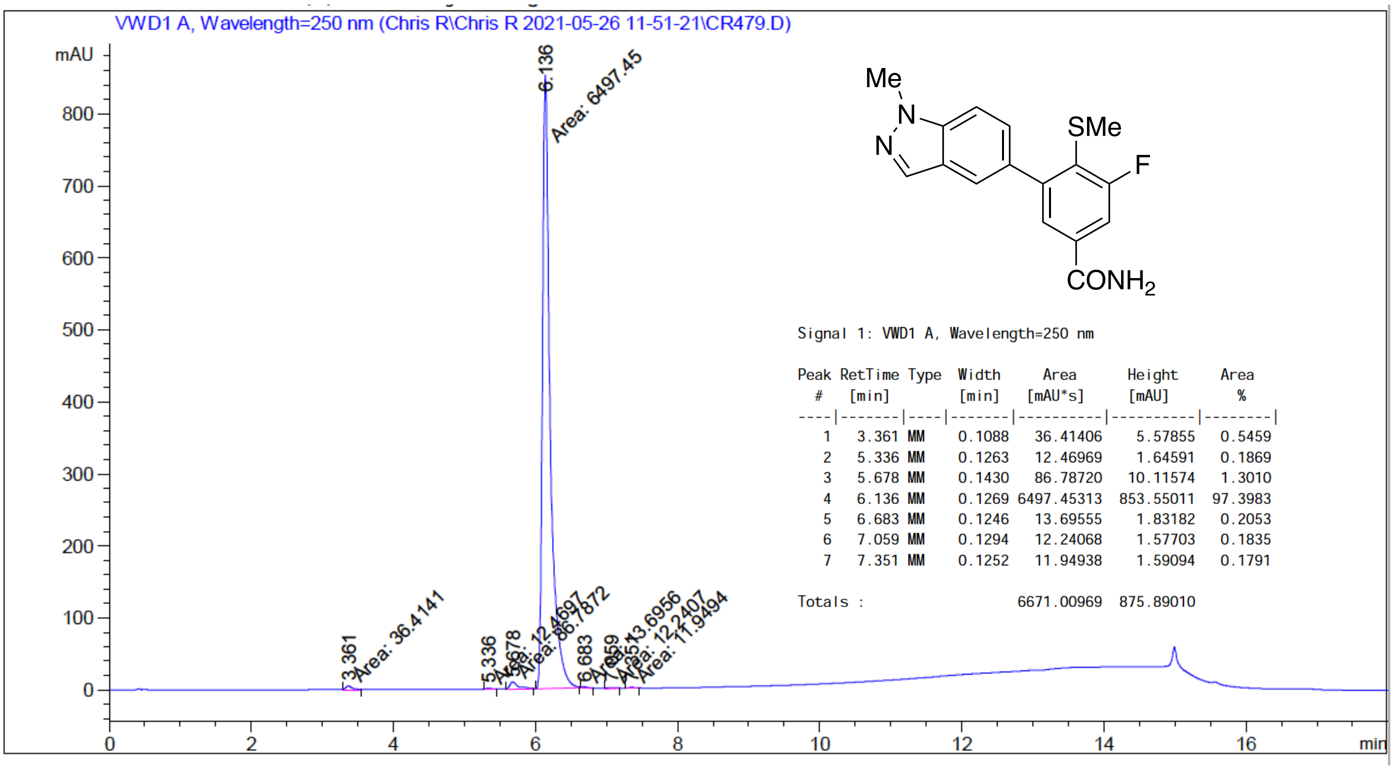

Signal 1: VWD1 A, Wavelength=250 nm

Peak #	RetTime [min]	Type	Width [min]	Area [mAU*s]	Height [mAU]	Area %
1	3.361	MM	0.1088	36.41406	5.57855	0.5459
2	5.336	MM	0.1263	12.46969	1.64591	0.1869
3	5.678	MM	0.1430	86.78720	10.11574	1.3010
4	6.136	MM	0.1269	6497.45313	853.55011	97.3983
5	6.683	MM	0.1246	13.69555	1.83182	0.2053
6	7.059	MM	0.1294	12.24068	1.57703	0.1835
7	7.351	MM	0.1252	11.94938	1.59094	0.1791
Totals :				6671.00969	875.89010	

HPLC Trace of 3-(1-methyl-1*H*-indazol-5-yl)-5-(methylthio)benzamide (39)

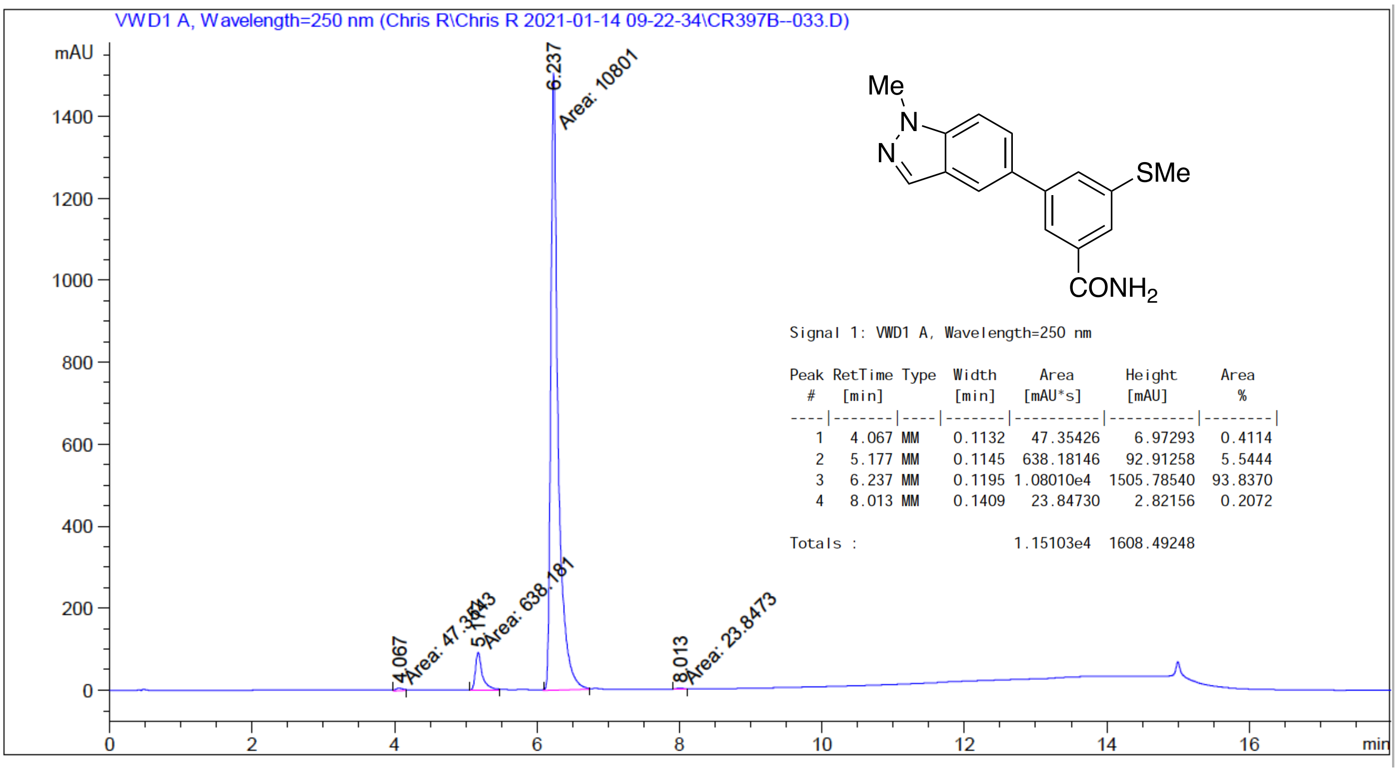

Signal 1: VWD1 A, Wavelength=250 nm

Peak #	RetTime [min]	Type	Width [min]	Area [mAU*s]	Height [mAU]	Area %
1	4.067	MM	0.1132	47.35426	6.97293	0.4114
2	5.177	MM	0.1145	638.18146	92.91258	5.5444
3	6.237	MM	0.1195	1.08010e4	1505.78540	93.8370
4	8.013	MM	0.1409	23.84730	2.82156	0.2072
Totals :				1.15103e4	1608.49248	

HPLC Trace of 4-(benzylthio)-3-(1-methyl-1*H*-indazol-5-yl)benzamide (40)

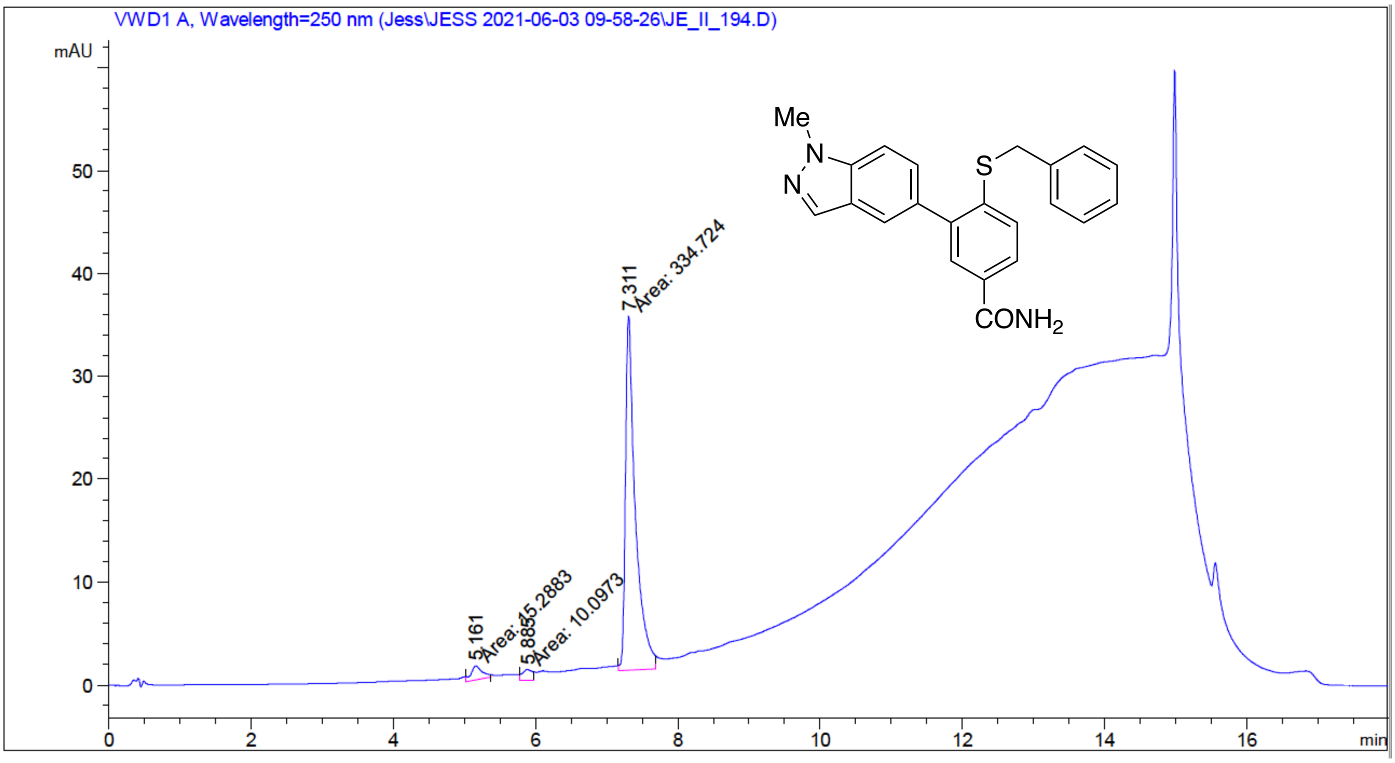

Signal 1: VWD1 A, Wavelength=250 nm

Peak #	RetTime [min]	Type	Width [min]	Area [mAU*s]	Height [mAU]	Area %
1	5.161	MM	0.1850	15.28829	1.37703	4.2455
2	5.885	MM	0.1640	10.09726	1.02640	2.8039
3	7.311	MM	0.1620	334.72382	34.43770	92.9506

Totals : 360.10936 36.84113

HPLC Trace of 4-methyl-3-(1-methyl-1*H*-indazol-5-yl)benzamide (41)

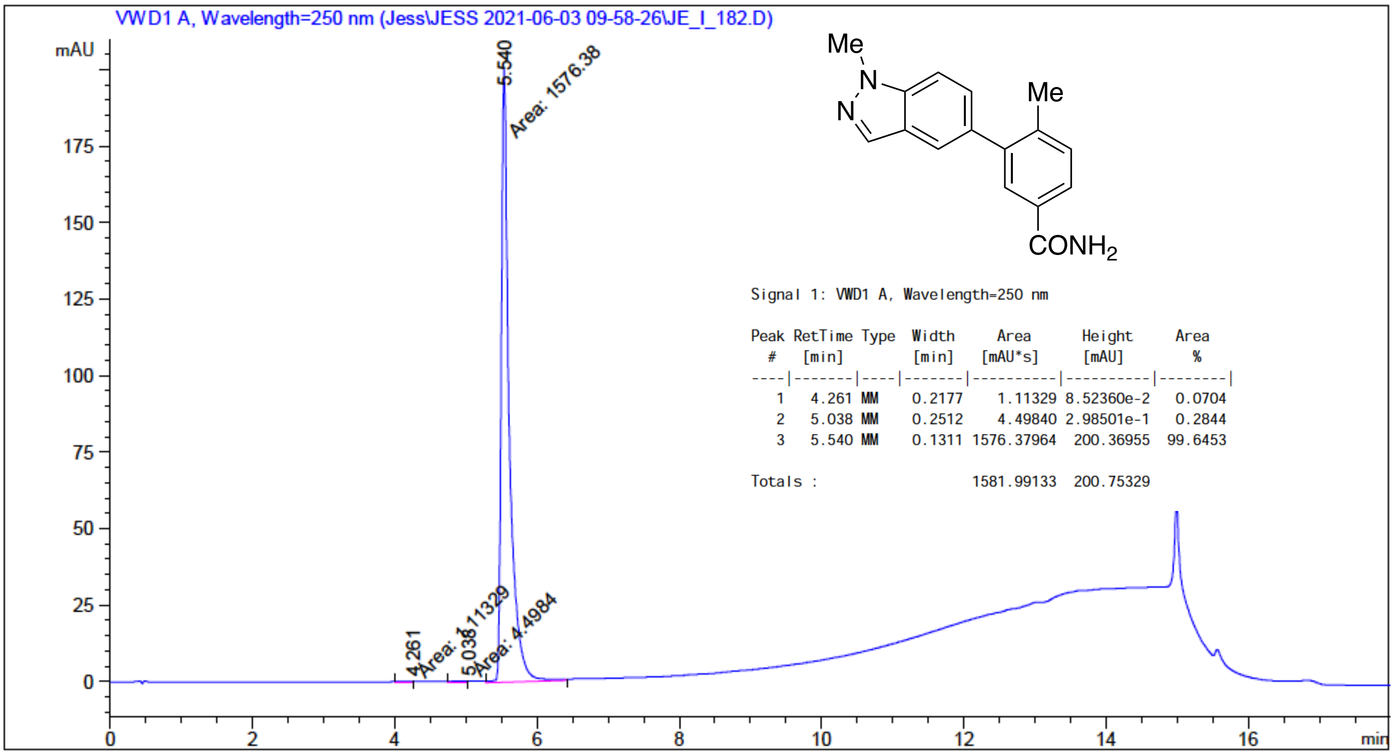

Signal 1: VWD1 A, Wavelength=250 nm

Peak #	RetTime [min]	Type	Width [min]	Area [mAU*s]	Height [mAU]	Area %
1	4.261	MM	0.2177	1.11329	8.52360e-2	0.0704
2	5.038	MM	0.2512	4.49840	2.98501e-1	0.2844
3	5.540	MM	0.1311	1576.37964	200.36955	99.6453

Totals : 1581.99133 200.75329

HPLC Trace of 4-ethyl-3-(1-methyl-1H-indazol-5-yl)benzamide (42)

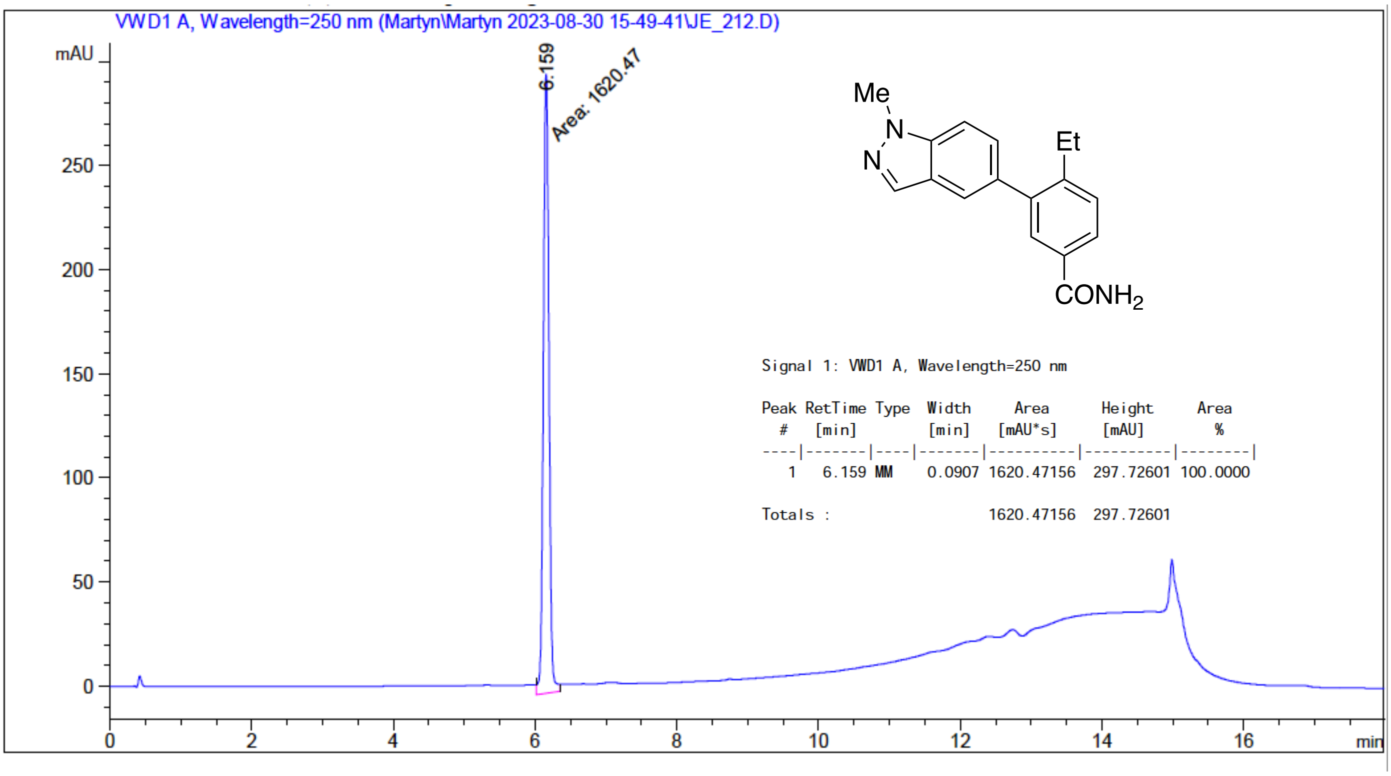

Signal 1: VWD1 A, Wavelength=250 nm

Peak #	RetTime [min]	Type	Width [min]	Area [mAU*s]	Height [mAU]	Area %
1	6.159	MM	0.0907	1620.47156	297.72601	100.0000

Totals : 1620.47156 297.72601

HPLC Trace of 3-(1-methyl-1H-indazol-5-yl)benzamide (43)

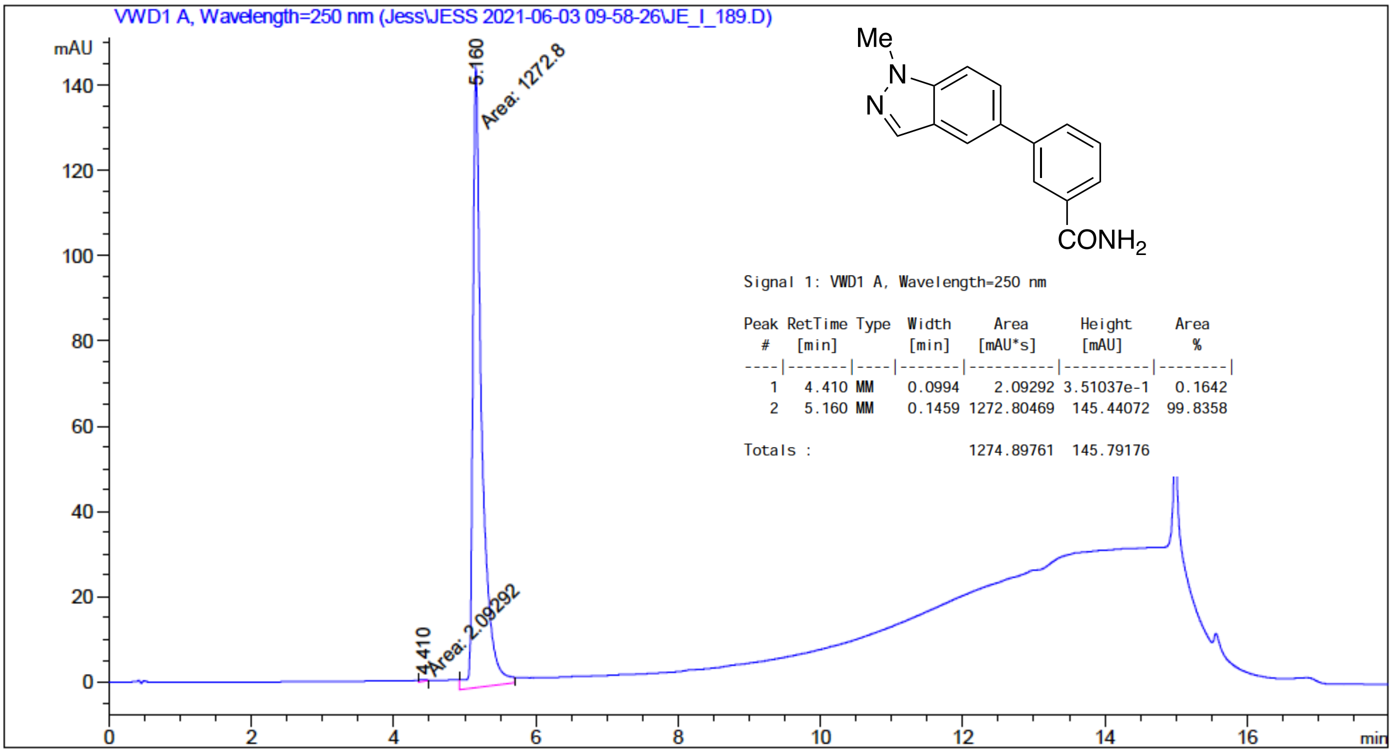

Signal 1: VWD1 A, Wavelength=250 nm

Peak #	RetTime [min]	Type	Width [min]	Area [mAU*s]	Height [mAU]	Area %
1	4.410	MM	0.0994	2.09292	3.51037e-1	0.1642
2	5.160	MM	0.1459	1272.80469	145.44072	99.8358

Totals : 1274.89761 145.79176

HPLC Trace of 4-fluoro-3-(1-methyl-1H-indazol-5-yl)benzamide (44)

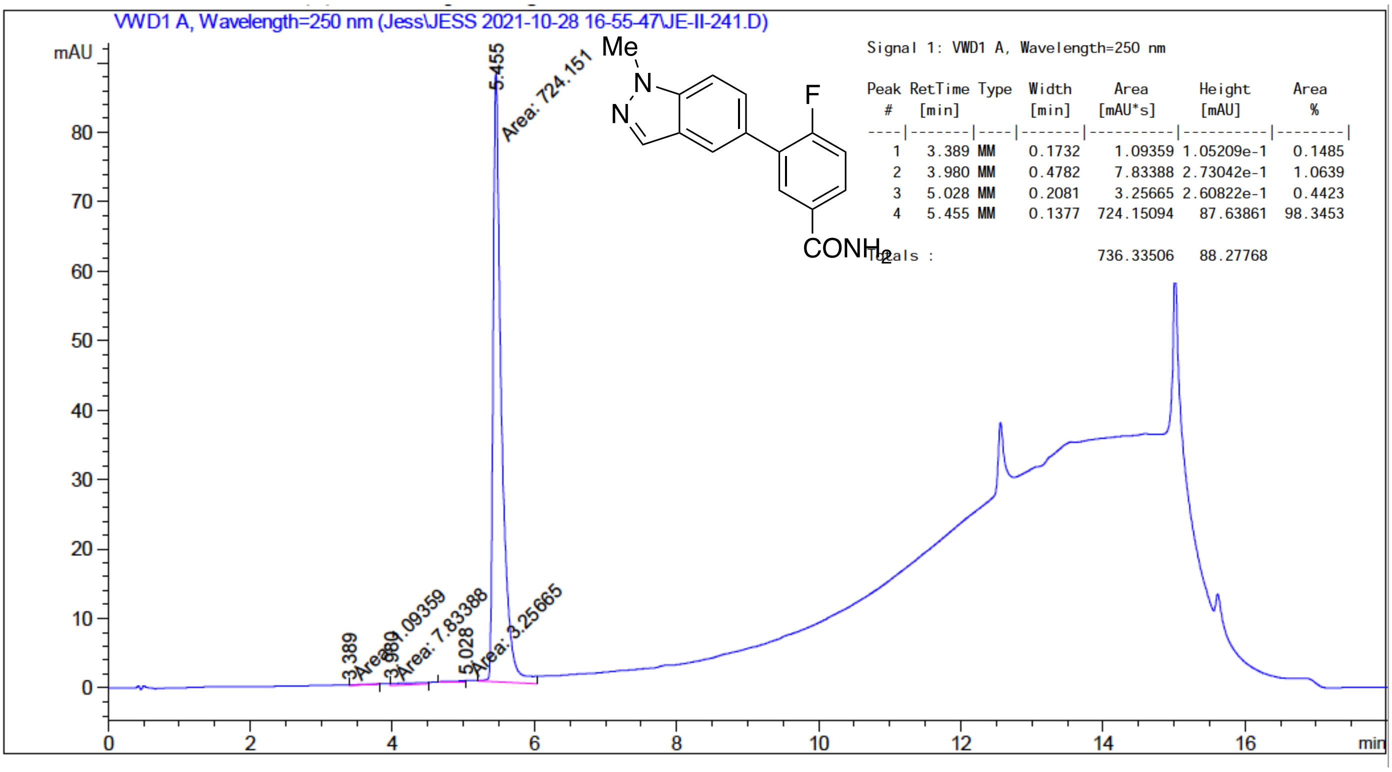

Signal 1: VWD1 A, Wavelength=250 nm

Peak #	RetTime [min]	Type	Width [min]	Area [mAU*s]	Height [mAU]	Area %
1	3.389	MM	0.1732	1.09359	1.05209e-1	0.1485
2	3.980	MM	0.4782	7.83388	2.73042e-1	1.0639
3	5.028	MM	0.2081	3.25665	2.60822e-1	0.4423
4	5.455	MM	0.1377	724.15094	87.63861	98.3453
Totals :				736.33506	88.27768	

HPLC Trace of 3-(1-methyl-1*H*-indazol-5-yl)-4-(trifluoromethyl)benzamide (45)

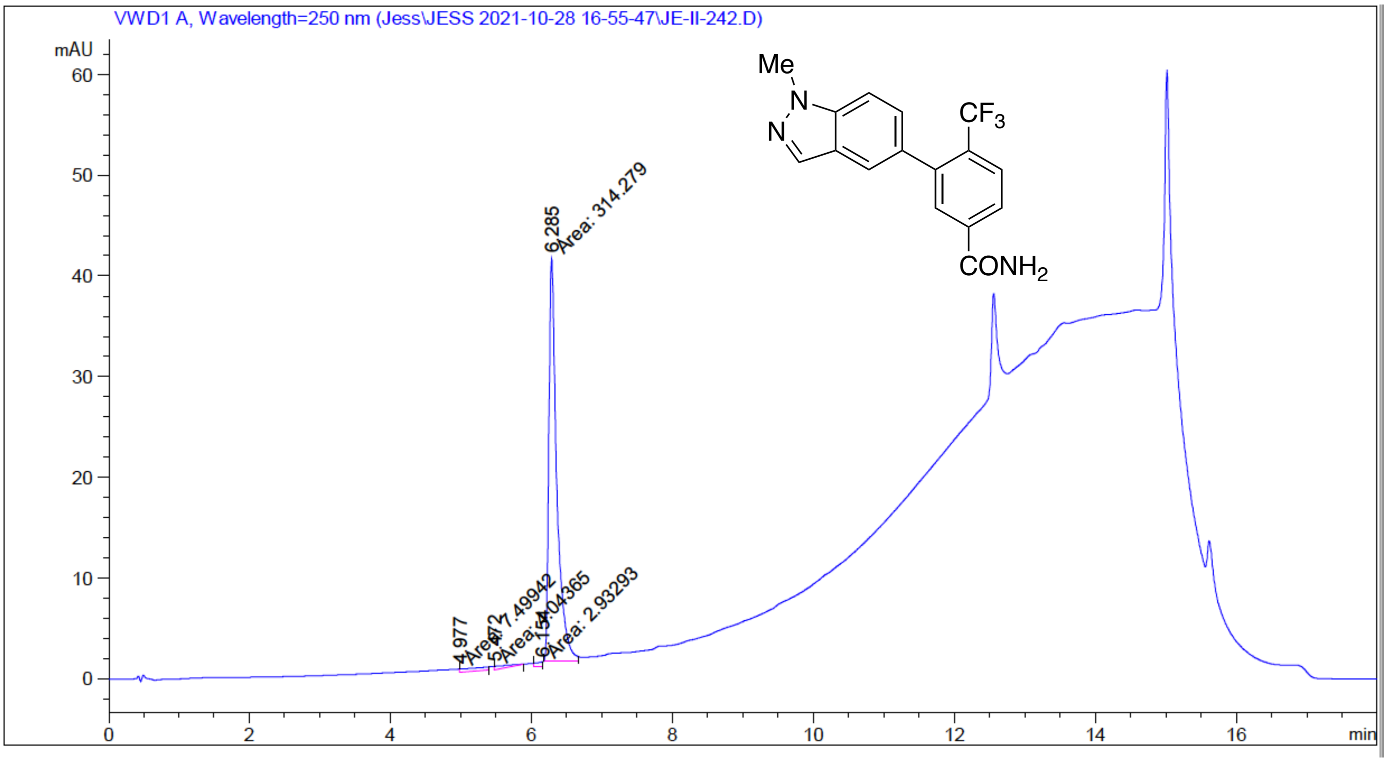

Signal 1: VWD1 A, Wavelength=250 nm

Peak #	RetTime [min]	Type	Width [min]	Area [mAU*s]	Height [mAU]	Area %
1	4.977	MM	0.3626	7.49942	3.44701e-1	2.2812
2	5.472	MM	0.1977	4.04365	3.40948e-1	1.2300
3	6.154	MM	0.0994	2.93293	4.91770e-1	0.8921
4	6.285	PM	0.1308	314.27875	40.04781	95.5967
Totals :				328.75475	41.22523	

HPLC Trace of 4-methoxy-3-(1-methyl-1*H*-indazol-5-yl)benzamide (46)

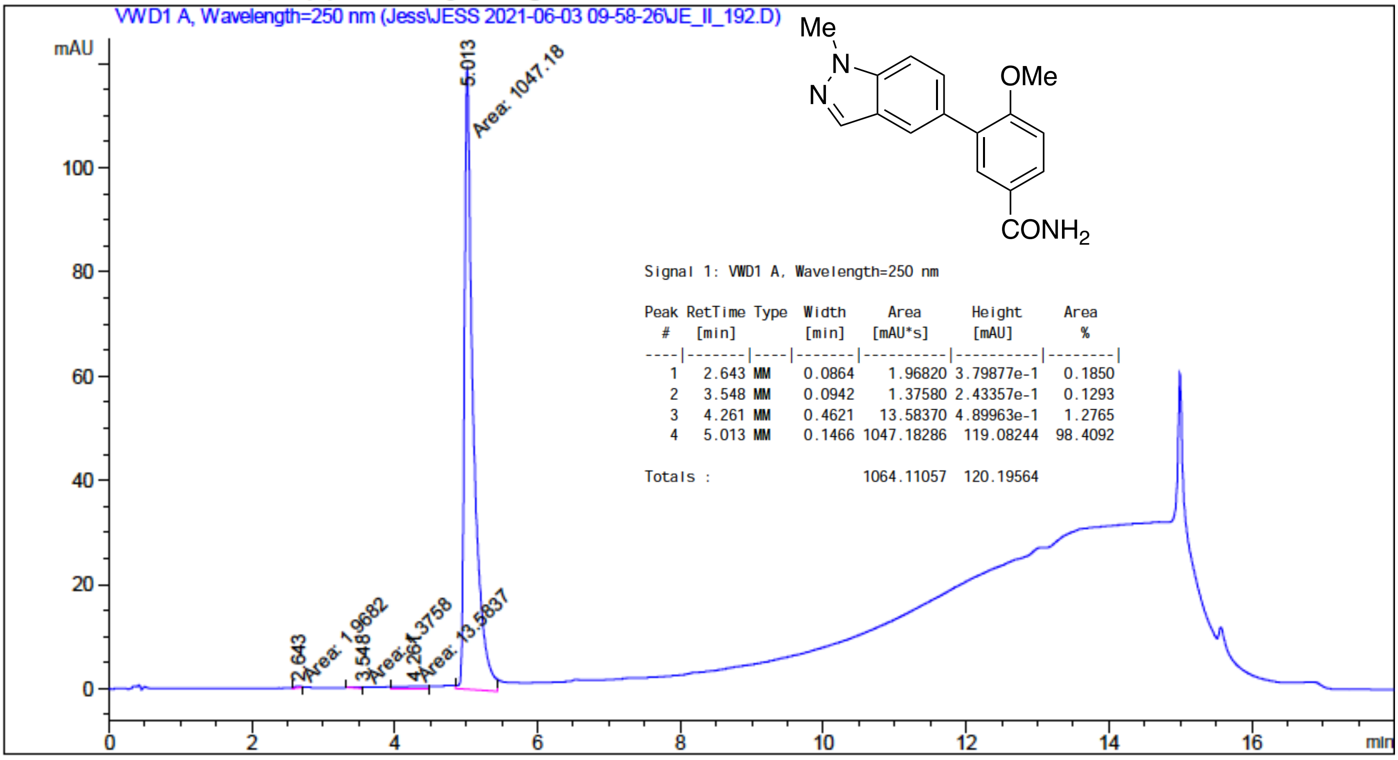

Peak #	RetTime [min]	Type	Width [min]	Area [mAU*s]	Height [mAU]	Area %
1	2.643	MM	0.0864	1.96820	3.79877e-1	0.1850
2	3.548	MM	0.0942	1.37580	2.43357e-1	0.1293
3	4.261	MM	0.4621	13.58370	4.89963e-1	1.2765
4	5.013	MM	0.1466	1047.18286	119.08244	98.4092
Totals :				1064.11057	120.19564	

HPLC Trace of 4-(benzyloxy)-3-(1-methyl-1*H*-indazol-5-yl)benzamide (47)

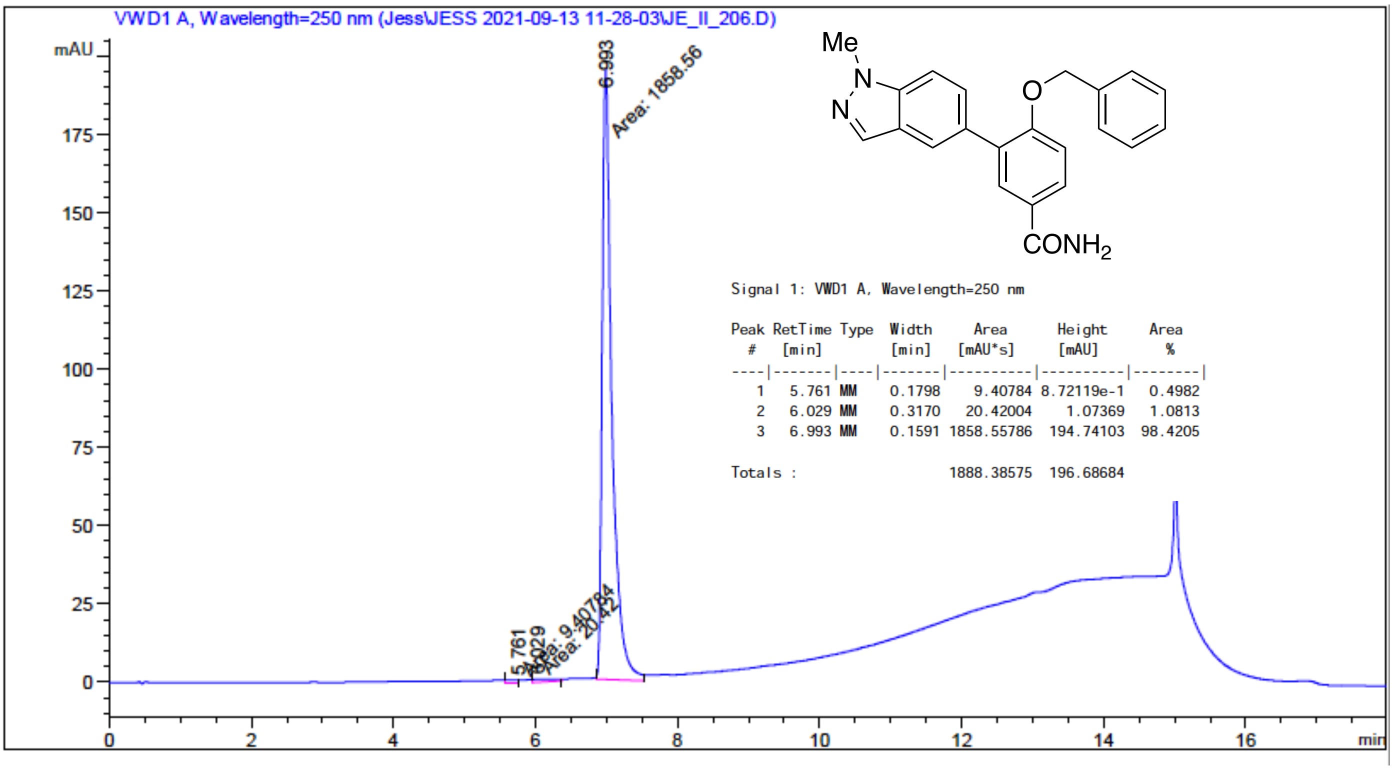

Peak #	RetTime [min]	Type	Width [min]	Area [mAU*s]	Height [mAU]	Area %
1	5.761	MM	0.1798	9.40784	8.72119e-1	0.4982
2	6.029	MM	0.3170	20.42004	1.07369	1.0813
3	6.993	MM	0.1591	1858.55786	194.74103	98.4205
Totals :				1888.38575	196.68684	

HPLC Trace of 3-(1-methyl-1*H*-indazol-5-yl)-4-(trifluoromethoxy)benzamide (48)

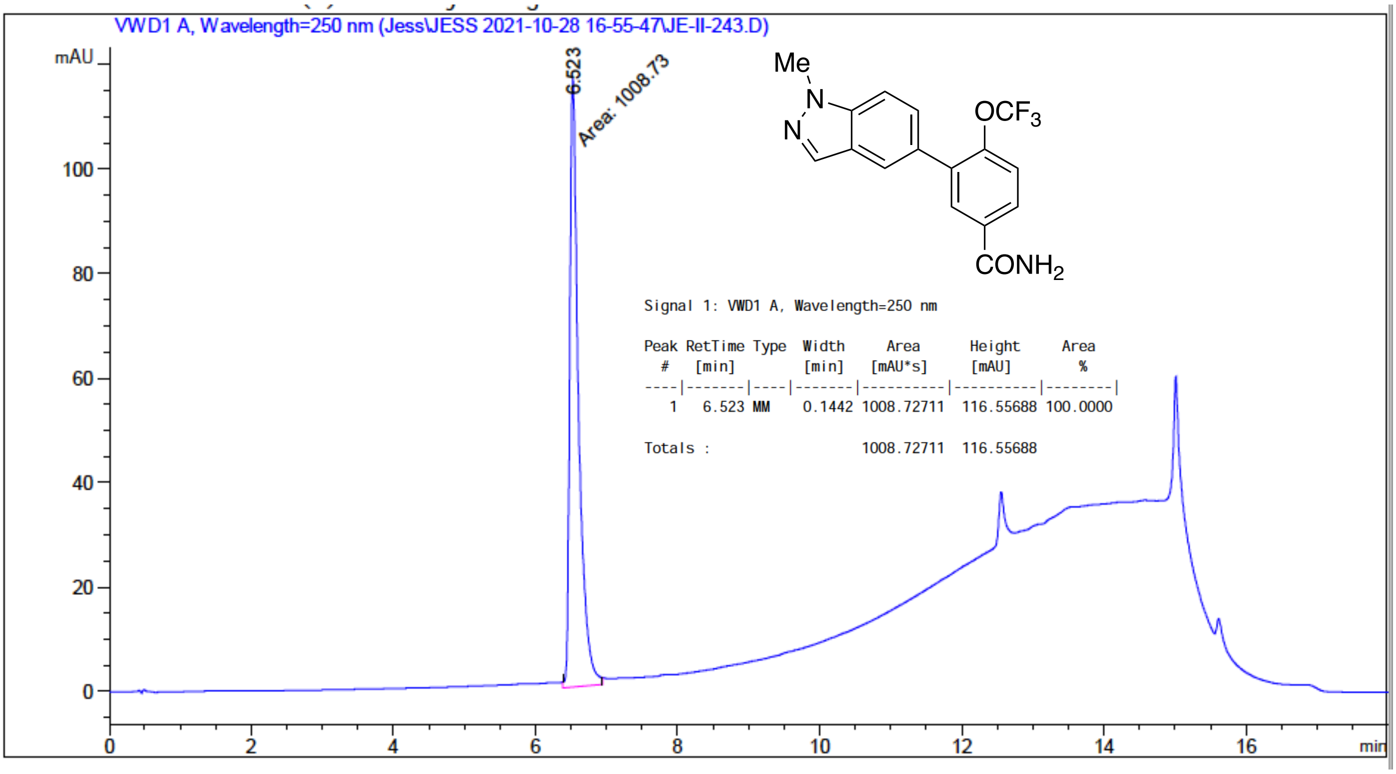

Peak #	RetTime [min]	Type	Width [min]	Area [mAU*s]	Height [mAU]	Area %
1	6.523	MM	0.1442	1008.72711	116.55688	100.0000
Totals :				1008.72711	116.55688	

HPLC Trace of 4-hydroxy-3-(1-methyl-1*H*-indazol-5-yl)benzamide (49)

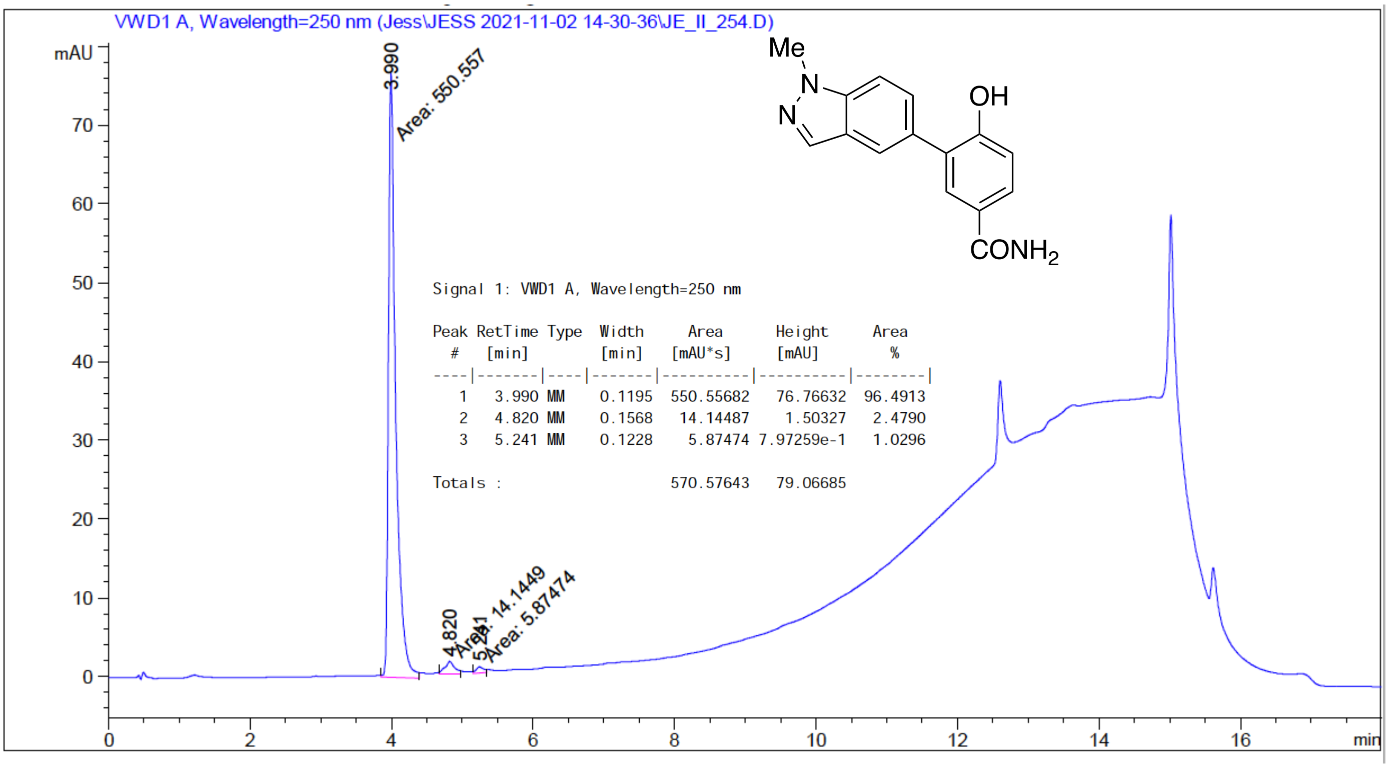

Peak #	RetTime [min]	Type	Width [min]	Area [mAU*s]	Height [mAU]	Area %
1	3.990	MM	0.1195	550.55682	76.76632	96.4913
2	4.820	MM	0.1568	14.14487	1.50327	2.4790
3	5.241	MM	0.1228	5.87474	7.97259e-1	1.0296
Totals :				570.57643	79.06685	

HPLC Trace of 4-(cyclopropylmethoxy)-3-(1-methyl-1*H*-indazol-5-yl)benzamide (50)

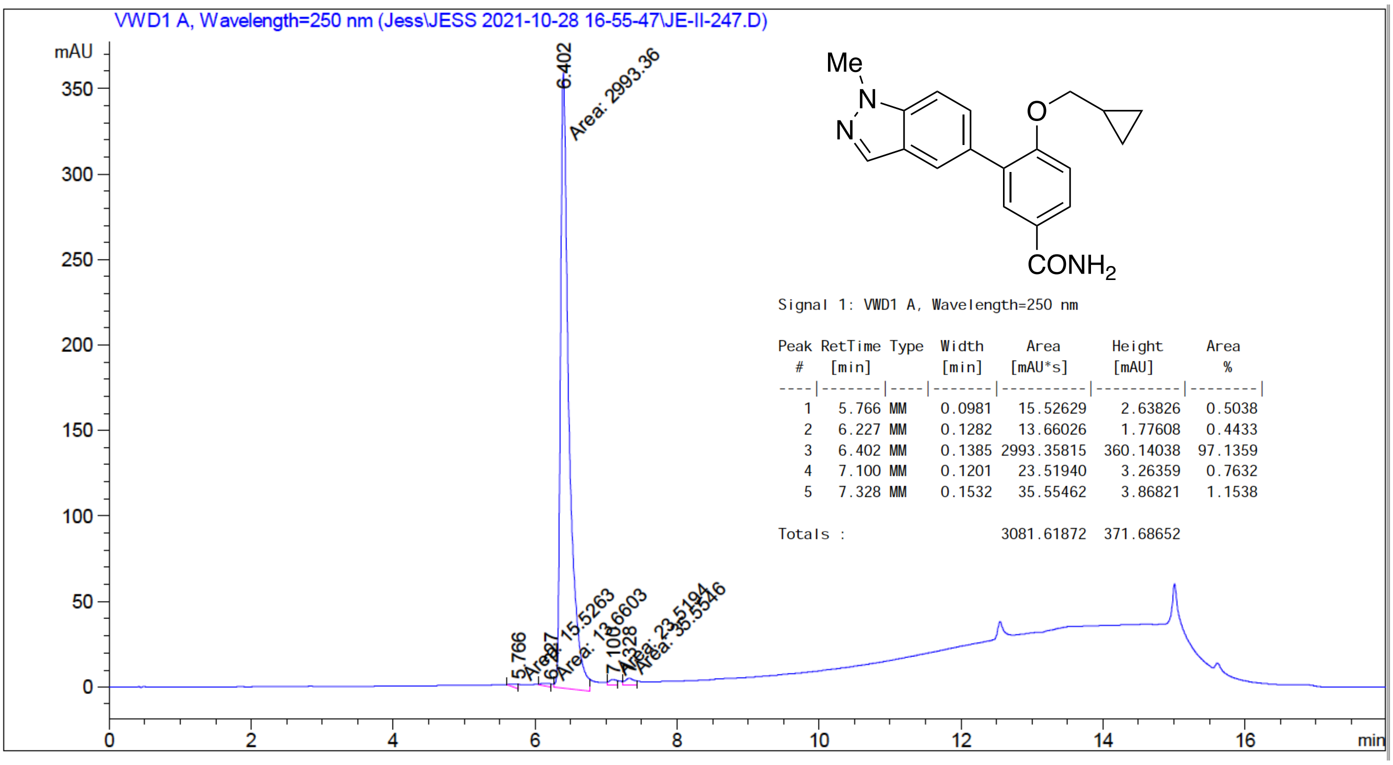

Signal 1: VWD1 A, Wavelength=250 nm

Peak #	RetTime [min]	Type	Width [min]	Area [mAU*s]	Height [mAU]	Area %
1	5.766	MM	0.0981	15.52629	2.63826	0.5038
2	6.227	MM	0.1282	13.66026	1.77608	0.4433
3	6.402	MM	0.1385	2993.35815	360.14038	97.1359
4	7.100	MM	0.1201	23.51940	3.26359	0.7632
5	7.328	MM	0.1532	35.55462	3.86821	1.1538
Totals :				3081.61872	371.68652	

HPLC Trace of 4-isobutoxy-3-(1-methyl-1*H*-indazol-5-yl)benzamide (51)

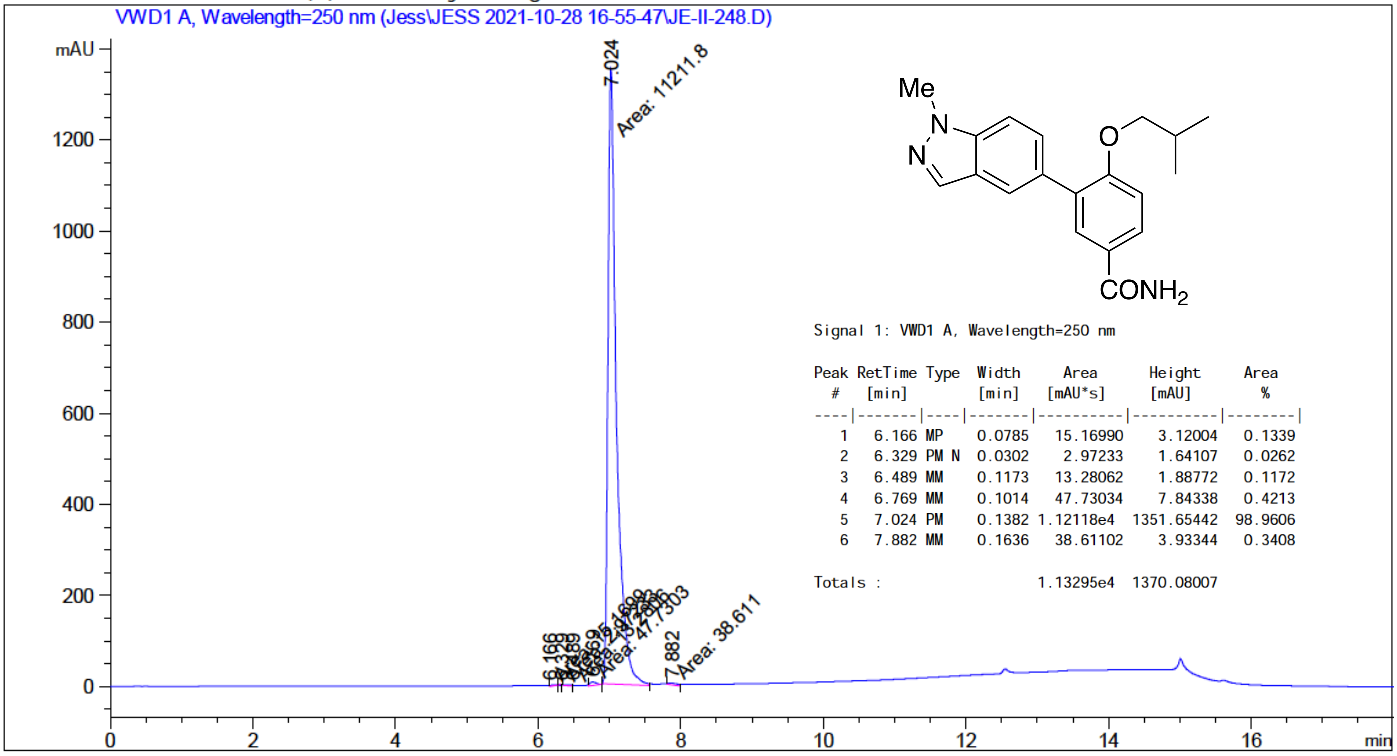

Signal 1: VWD1 A, Wavelength=250 nm

Peak #	RetTime [min]	Type	Width [min]	Area [mAU*s]	Height [mAU]	Area %
1	6.166	MP	0.0785	15.16990	3.12004	0.1339
2	6.329	PM N	0.0302	2.97233	1.64107	0.0262
3	6.489	MM	0.1173	13.28062	1.88772	0.1172
4	6.769	MM	0.1014	47.73034	7.84338	0.4213
5	7.024	PM	0.1382	1.12118e4	1351.65442	98.9606
6	7.882	MM	0.1636	38.61102	3.93344	0.3408
Totals :				1.13295e4	1370.08007	

HPLC Trace of 4-(benzylamino)-3-(1-methyl-1*H*-indazol-5-yl)benzamide (52)

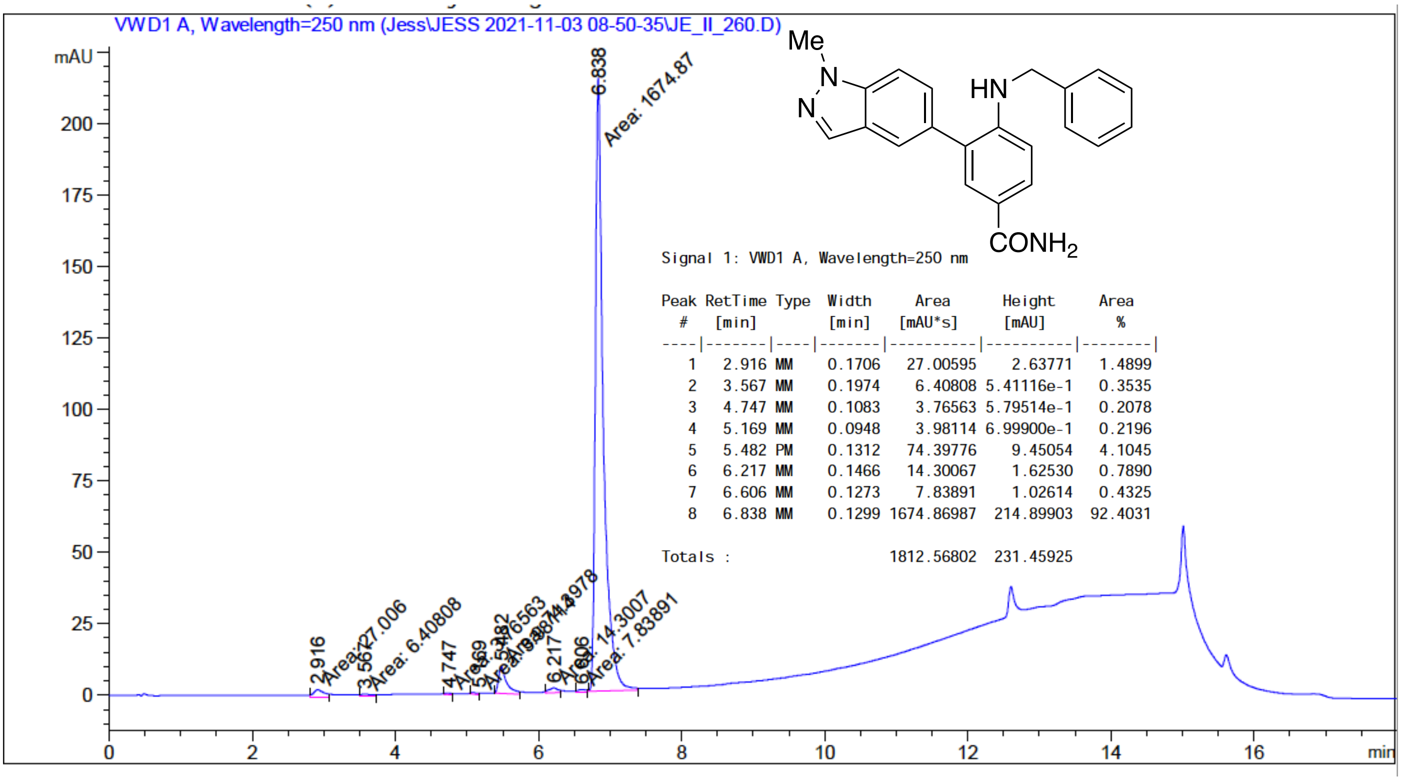

Signal 1: VWD1 A, Wavelength=250 nm

Peak #	RetTime [min]	Type	Width [min]	Area [mAU*s]	Height [mAU]	Area %
1	2.916	MM	0.1706	27.00595	2.63771	1.4899
2	3.567	MM	0.1974	6.40808	5.41116e-1	0.3535
3	4.747	MM	0.1083	3.76563	5.79514e-1	0.2078
4	5.169	MM	0.0948	3.98114	6.99900e-1	0.2196
5	5.482	PM	0.1312	74.39776	9.45054	4.1045
6	6.217	MM	0.1466	14.30067	1.62530	0.7890
7	6.606	MM	0.1273	7.83891	1.02614	0.4325
8	6.838	MM	0.1299	1674.86987	214.89903	92.4031

Totals : 1812.56802 231.45925

HPLC Trace of 3-(1-methyl-1*H*-indazol-5-yl)-4-morpholinobenzamide (53)

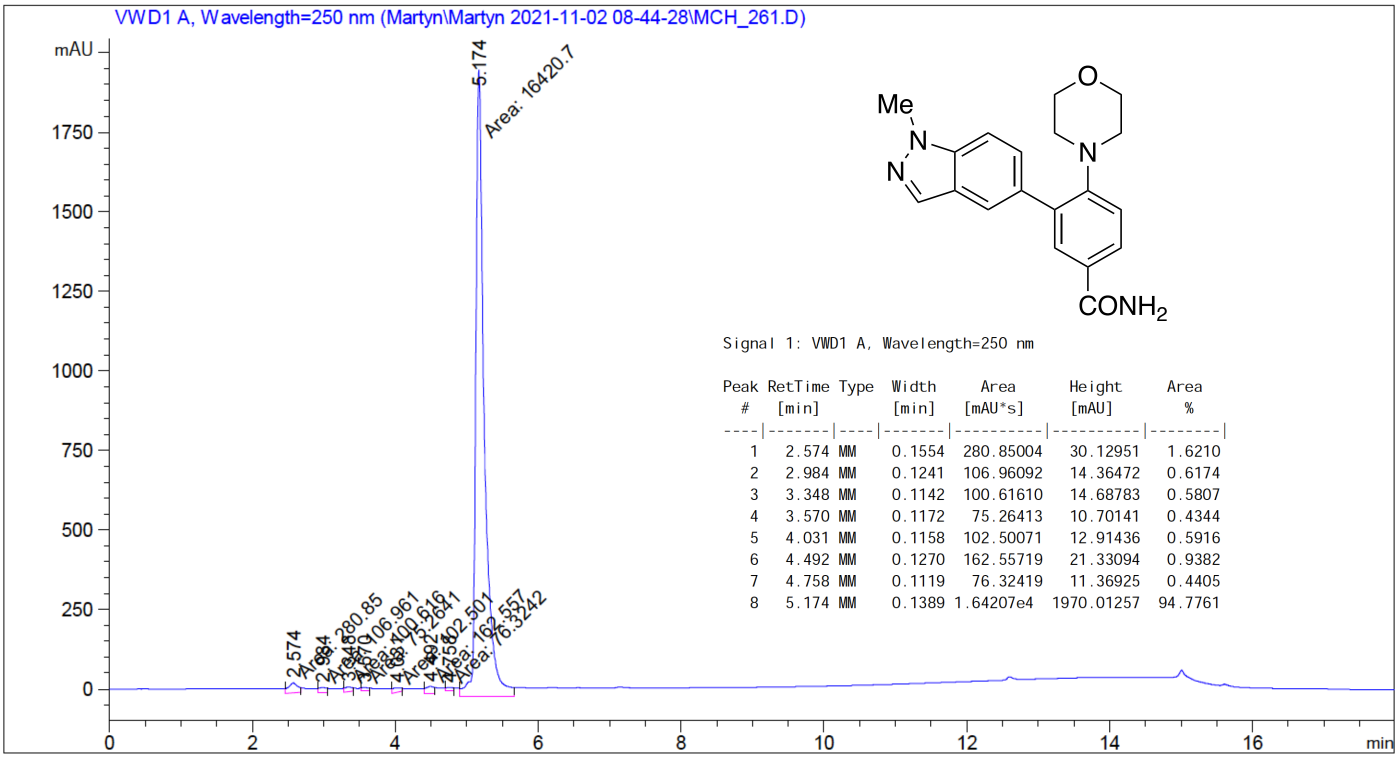

Signal 1: VWD1 A, Wavelength=250 nm

Peak #	RetTime [min]	Type	Width [min]	Area [mAU*s]	Height [mAU]	Area %
1	2.574	MM	0.1554	280.85004	30.12951	1.6210
2	2.984	MM	0.1241	106.96092	14.36472	0.6174
3	3.348	MM	0.1142	100.61610	14.68783	0.5807
4	3.570	MM	0.1172	75.26413	10.70141	0.4344
5	4.031	MM	0.1158	102.50071	12.91436	0.5916
6	4.492	MM	0.1270	162.55719	21.33094	0.9382
7	4.758	MM	0.1119	76.32419	11.36925	0.4405
8	5.174	MM	0.1389	1.64207e4	1970.01257	94.7761

HPLC Trace of 3-(1-methyl-1*H*-indazol-5-yl)-4-((2-morpholinoethyl)amino)benzamide (54)

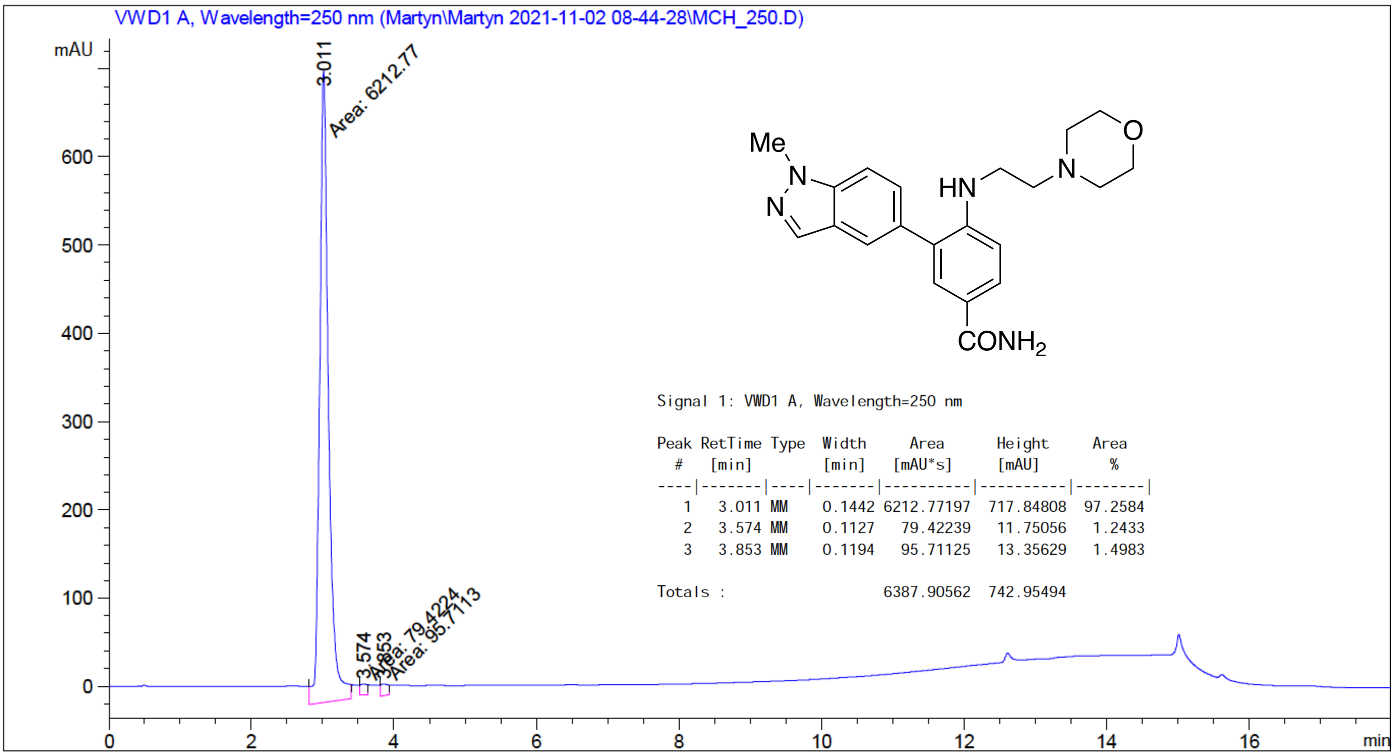

Signal 1: VWD1 A, Wavelength=250 nm

Peak #	RetTime [min]	Type	Width [min]	Area [mAU*s]	Height [mAU]	Area %
1	3.011	MM	0.1442	6212.77197	717.84808	97.2584
2	3.574	MM	0.1127	79.42239	11.75056	1.2433
3	3.853	MM	0.1194	95.71125	13.35629	1.4983
Totals :				6387.90562	742.95494	

HPLC Trace of 3-(1-methyl-1*H*-indazol-5-yl)-4-((2-(piperidin-1-yl)ethyl)amino)benzamide (55)

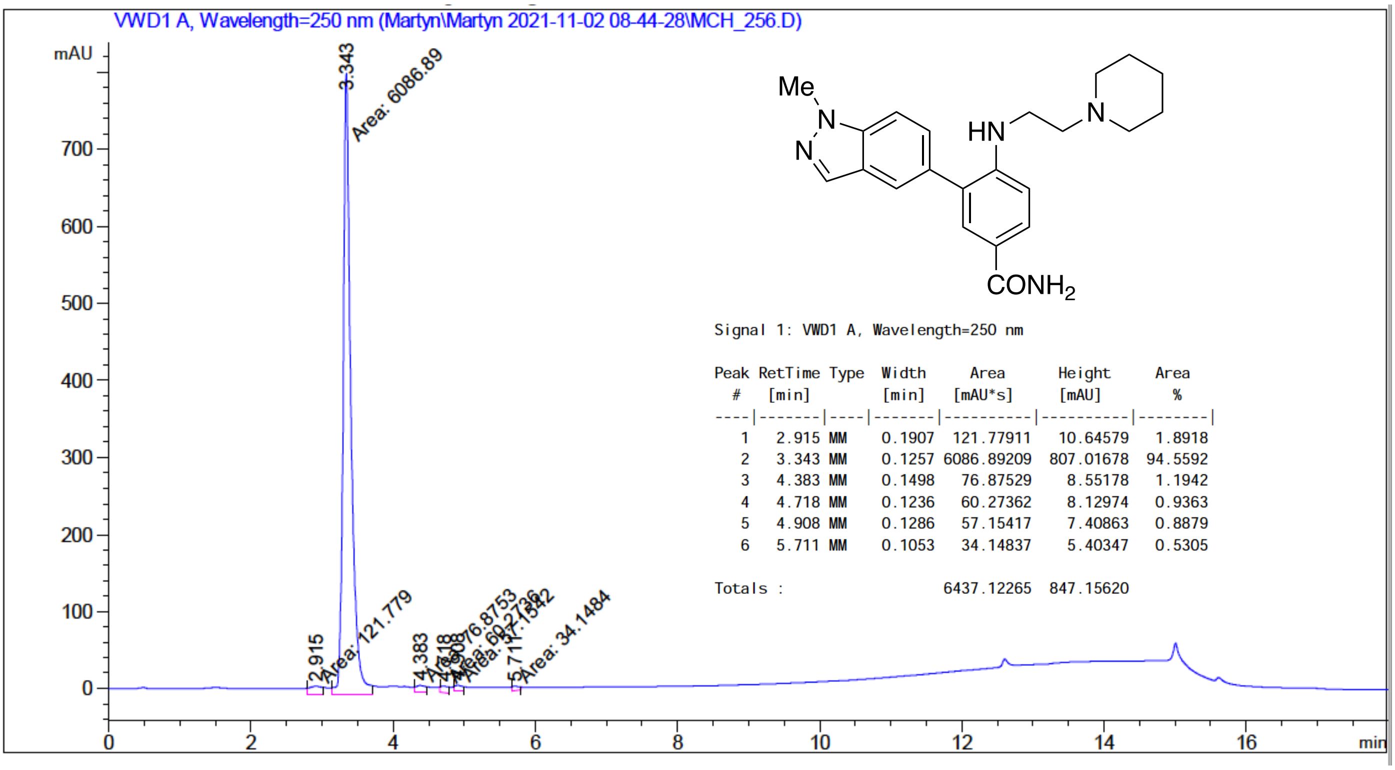

Signal 1: VWD1 A, Wavelength=250 nm

Peak #	RetTime [min]	Type	Width [min]	Area [mAU*s]	Height [mAU]	Area %
1	2.915	MM	0.1907	121.77911	10.64579	1.8918
2	3.343	MM	0.1257	6086.89209	807.01678	94.5592
3	4.383	MM	0.1498	76.87529	8.55178	1.1942
4	4.718	MM	0.1236	60.27362	8.12974	0.9363
5	4.908	MM	0.1286	57.15417	7.40863	0.8879
6	5.711	MM	0.1053	34.14837	5.40347	0.5305
Totals :				6437.12265	847.15620	

HPLC Trace of 4-((2-(methylamino)ethyl)amino)-3-(1-methyl-1*H*-indazol-5-yl)benzamide (56)

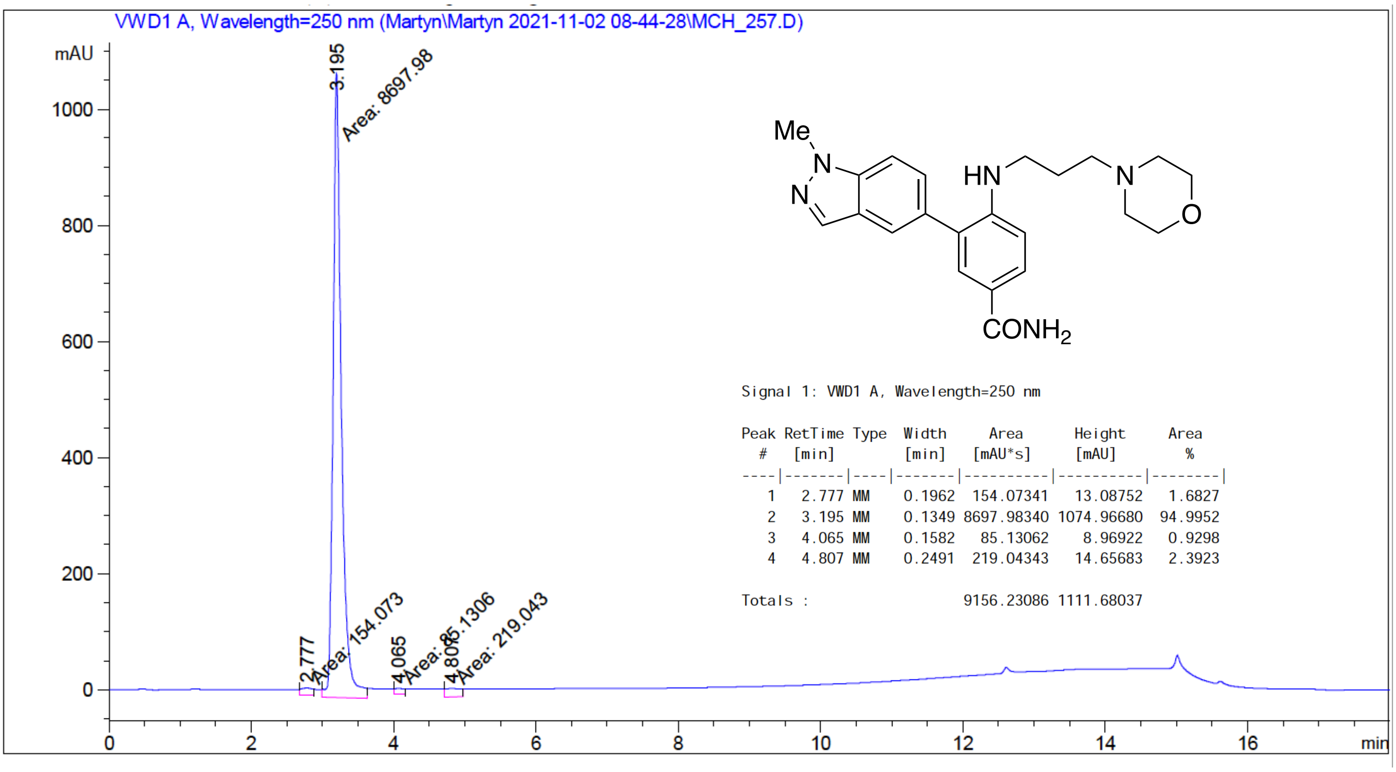

Signal 1: VWD1 A, Wavelength=250 nm

Peak #	RetTime [min]	Type	Width [min]	Area [mAU*s]	Height [mAU]	Area %
1	2.777	MM	0.1962	154.07341	13.08752	1.6827
2	3.195	MM	0.1349	8697.98340	1074.96680	94.9952
3	4.065	MM	0.1582	85.13062	8.96922	0.9298
4	4.807	MM	0.2491	219.04343	14.65683	2.3923

Totals : 9156.23086 1111.68037

HPLC Trace of 4-((2-(methylamino)ethyl)amino)-3-(1-methyl-1*H*-indazol-5-yl)benzamide (57)

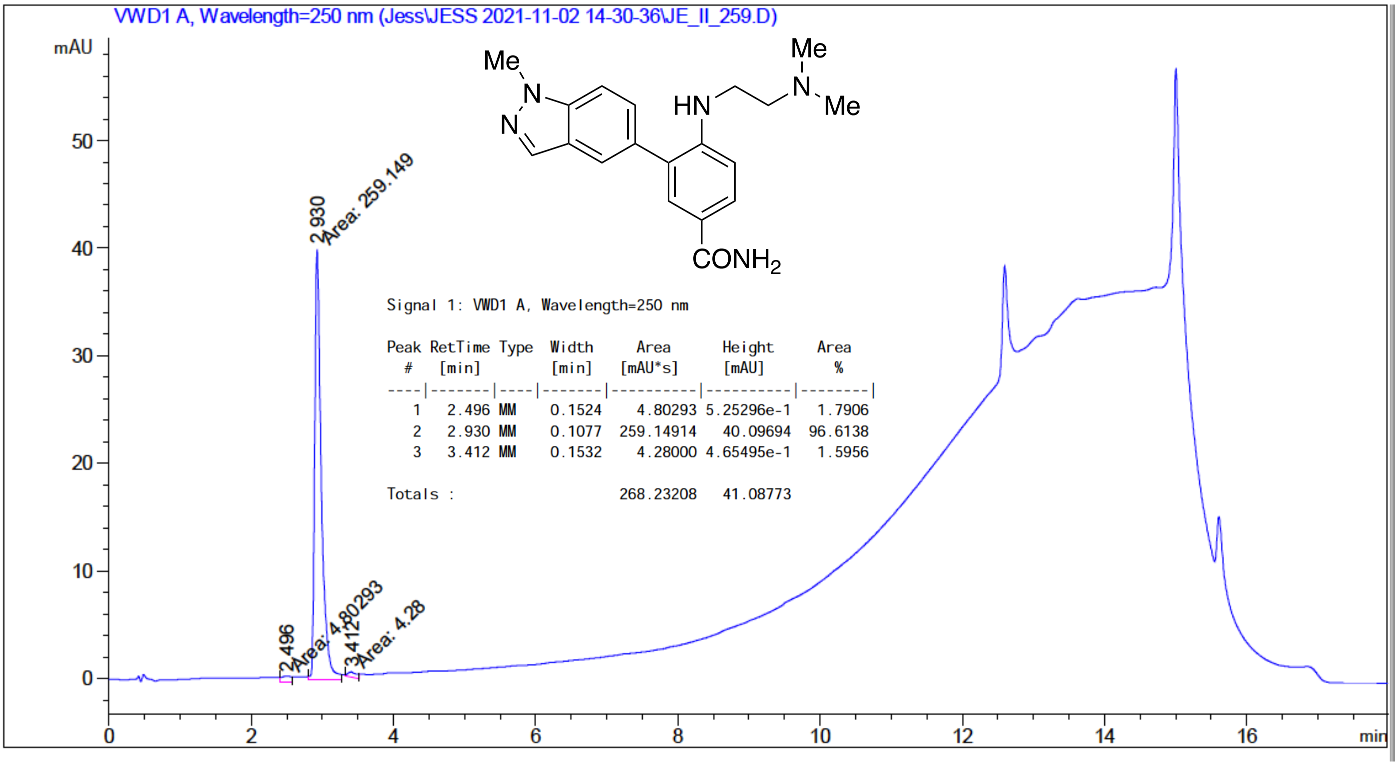

Signal 1: VWD1 A, Wavelength=250 nm

Peak #	RetTime [min]	Type	Width [min]	Area [mAU*s]	Height [mAU]	Area %
1	2.496	MM	0.1524	4.80293	5.25296e-1	1.7906
2	2.930	MM	0.1077	259.14914	40.09694	96.6138
3	3.412	MM	0.1532	4.28000	4.65495e-1	1.5956

Totals : 268.23208 41.08773

HPLC Trace of (4-(methylthio)-3-(pyrimidin-5-yl)phenyl)(piperidin-1-yl)methanone (59)

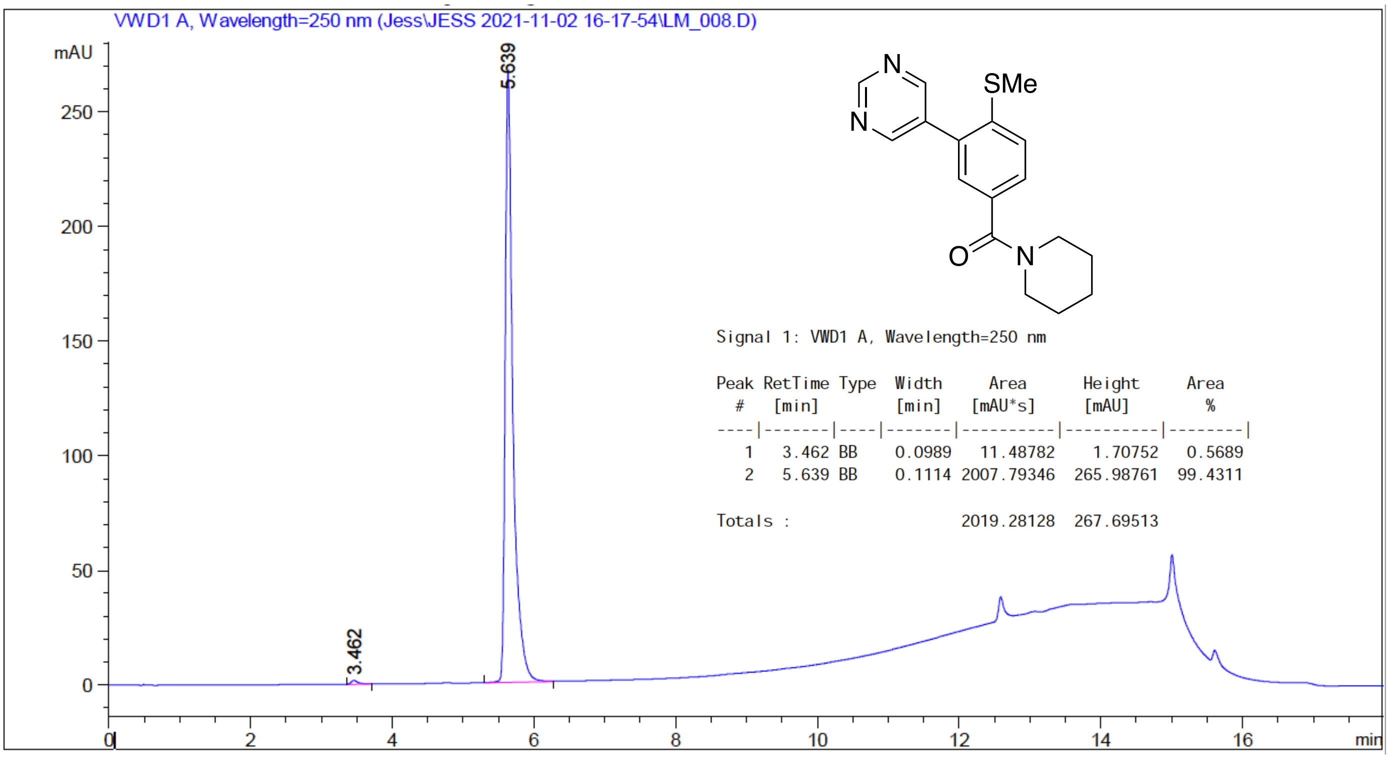

Signal 1: VWD1 A, Wavelength=250 nm

Peak #	RetTime [min]	Type	Width [min]	Area [mAU*s]	Height [mAU]	Area %
1	3.462	BB	0.0989	11.48782	1.70752	0.5689
2	5.639	BB	0.1114	2007.79346	265.98761	99.4311
Totals :				2019.28128	267.69513	

HPLC Trace of (4-(methylthio)-3-(pyrimidin-5-yl)phenyl)(morpholino)methanone (60)

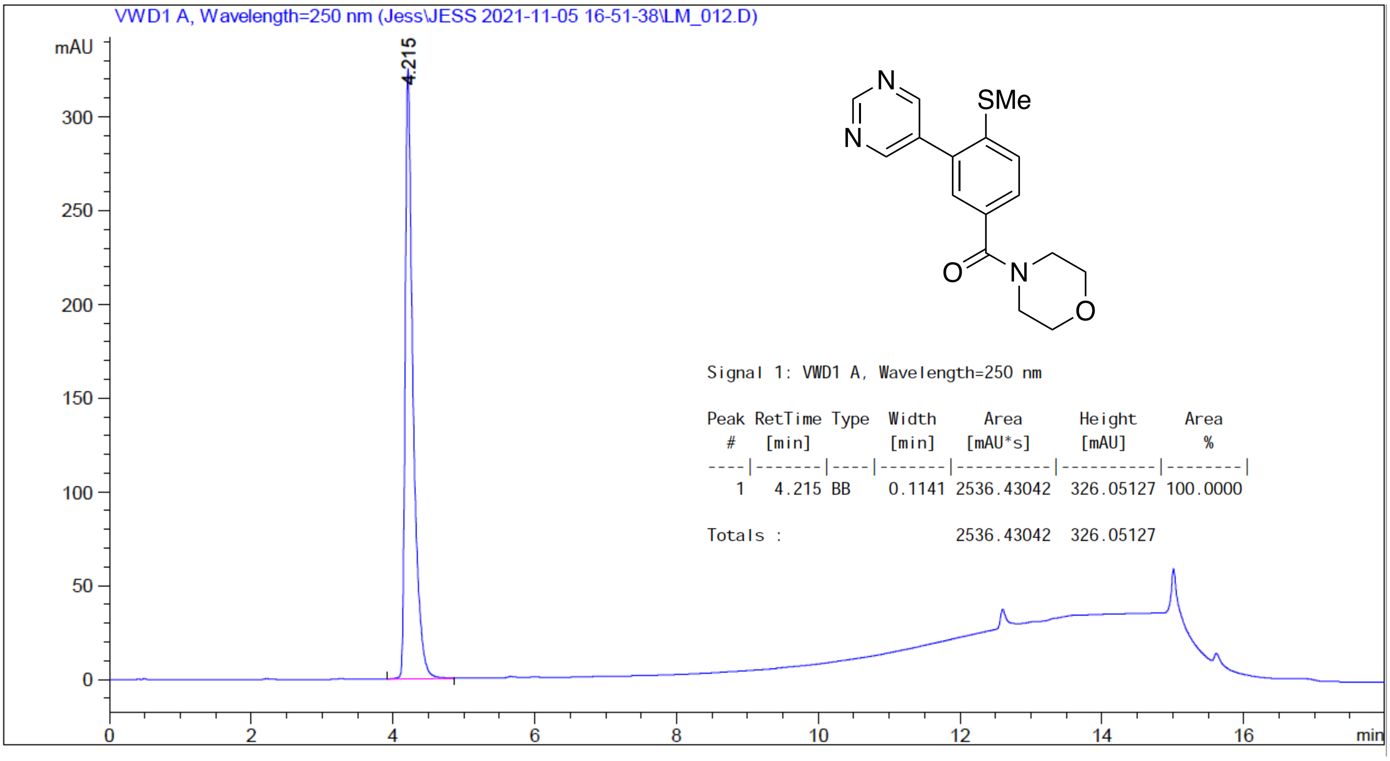

Signal 1: VWD1 A, Wavelength=250 nm

Peak #	RetTime [min]	Type	Width [min]	Area [mAU*s]	Height [mAU]	Area %
1	4.215	BB	0.1141	2536.43042	326.05127	100.0000
Totals :				2536.43042	326.05127	

8. Copies of ^1H and ^{13}C NMR spectra for Final Compounds

^1H NMR: 500 MHz, acetone-d_6

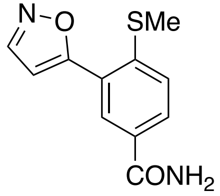

7

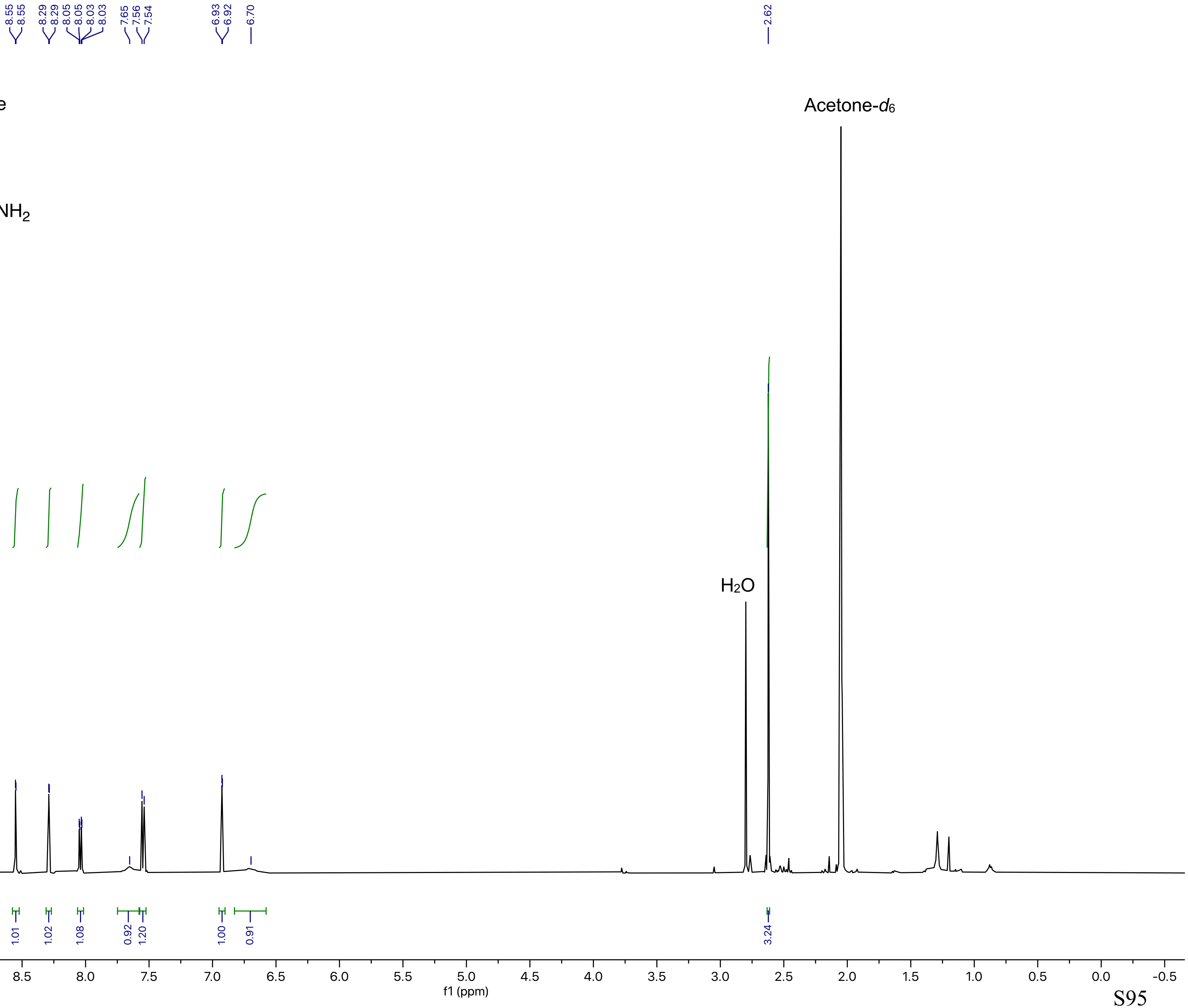

$^{13}C\{^{1}H\}$ NMR: 101 MHz, DMSO-d_6

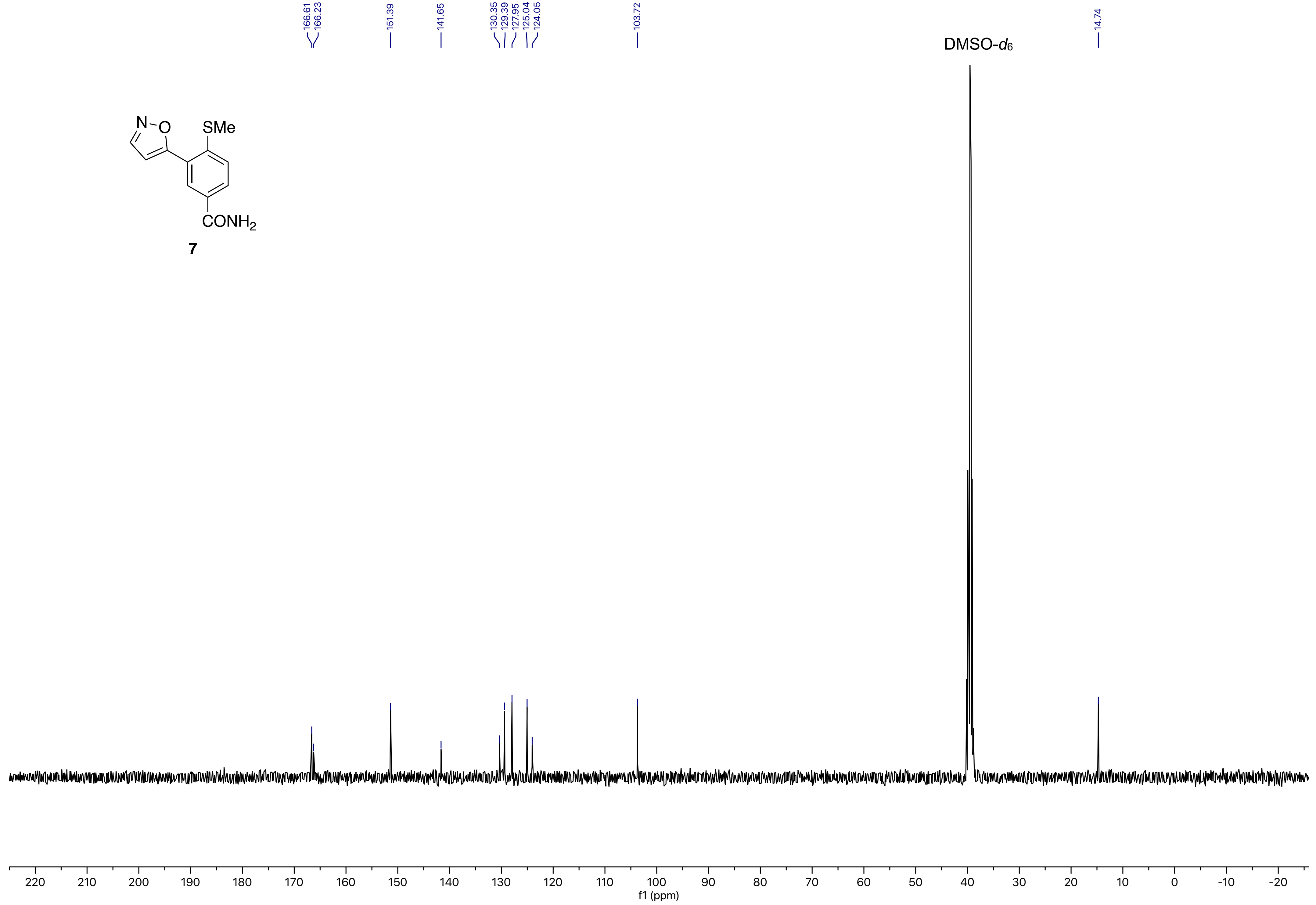

7

^{1}H NMR: 500 MHz, DMSO-d_6

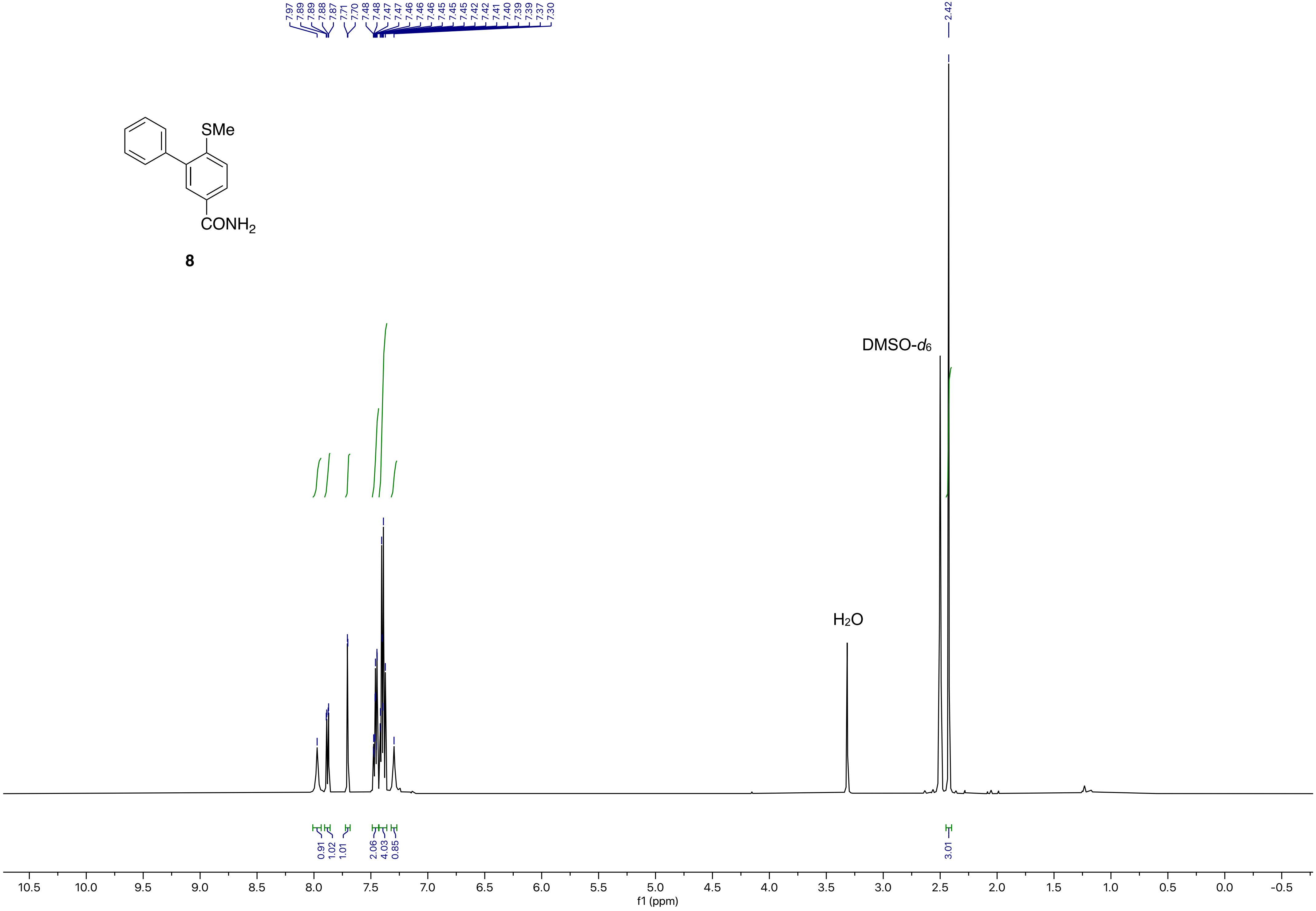

$^{13}C\{^{1}H\}$ NMR: 126 MHz, DMSO-d_6

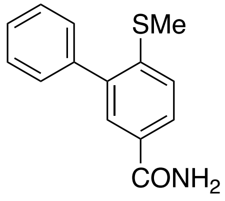

8

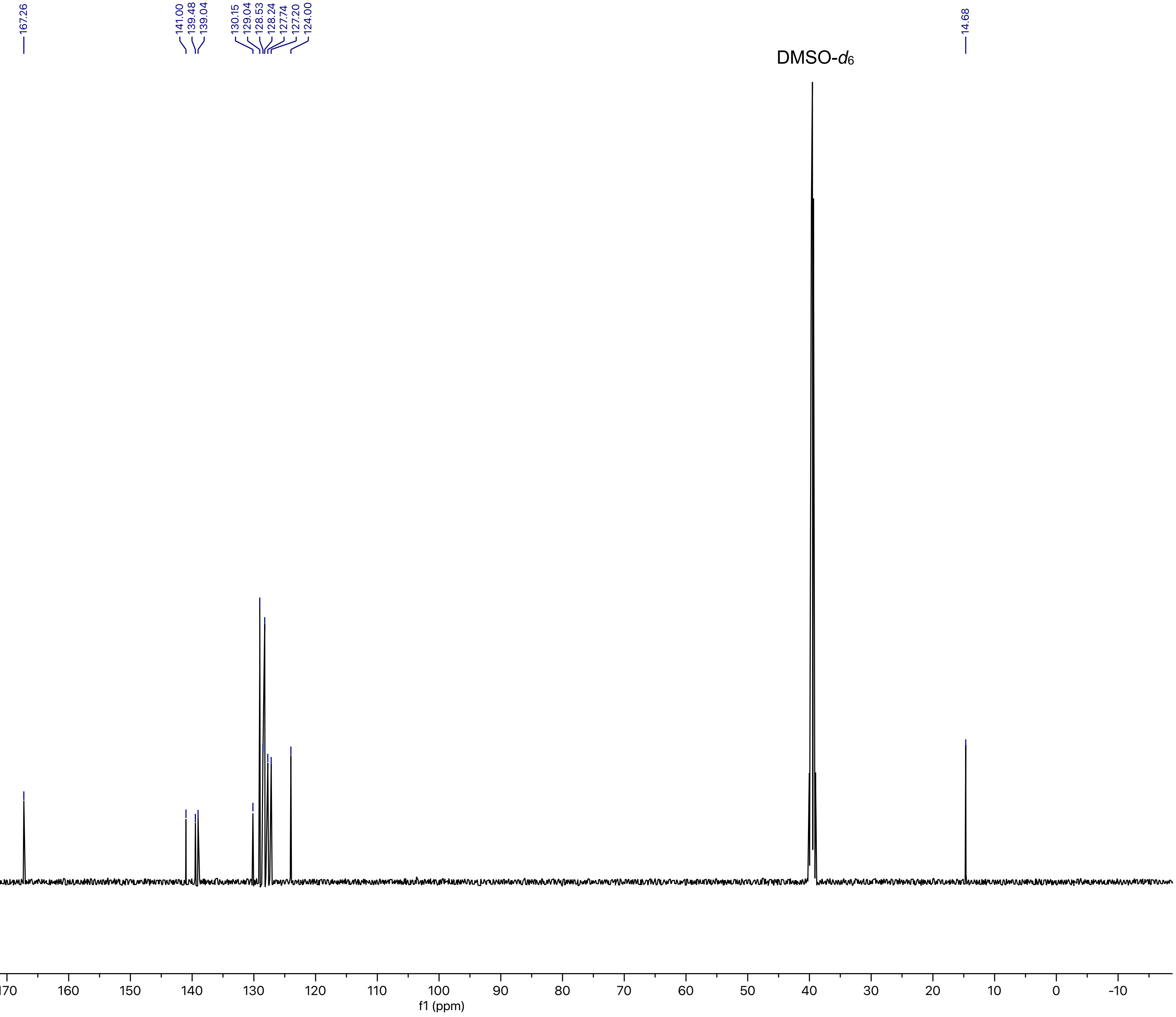

^{1}H NMR: 400 MHz, DMSO-d_6

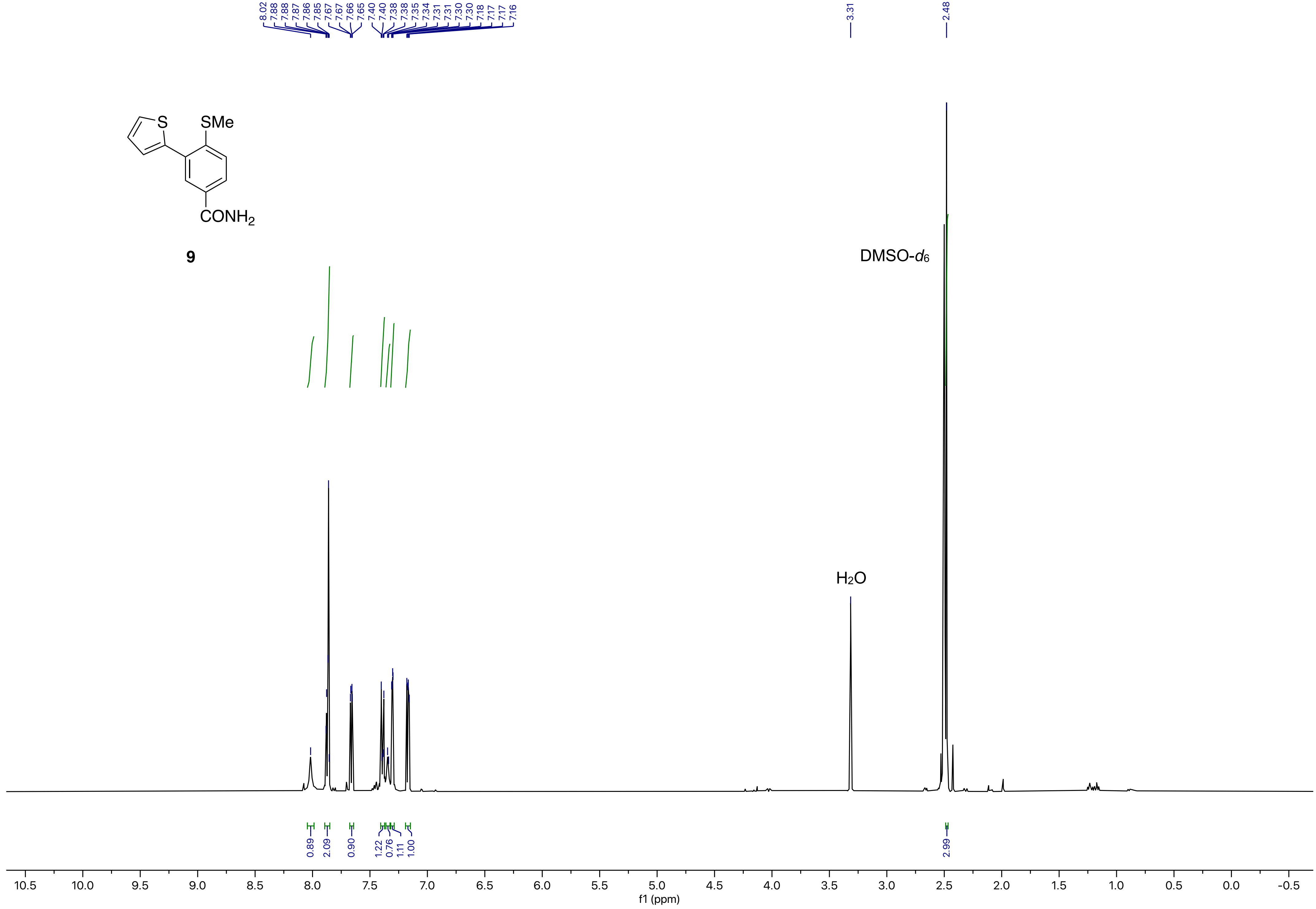

$^{13}C\{^{1}H\}$ NMR: 101 MHz, DMSO-d_6

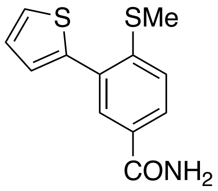

9

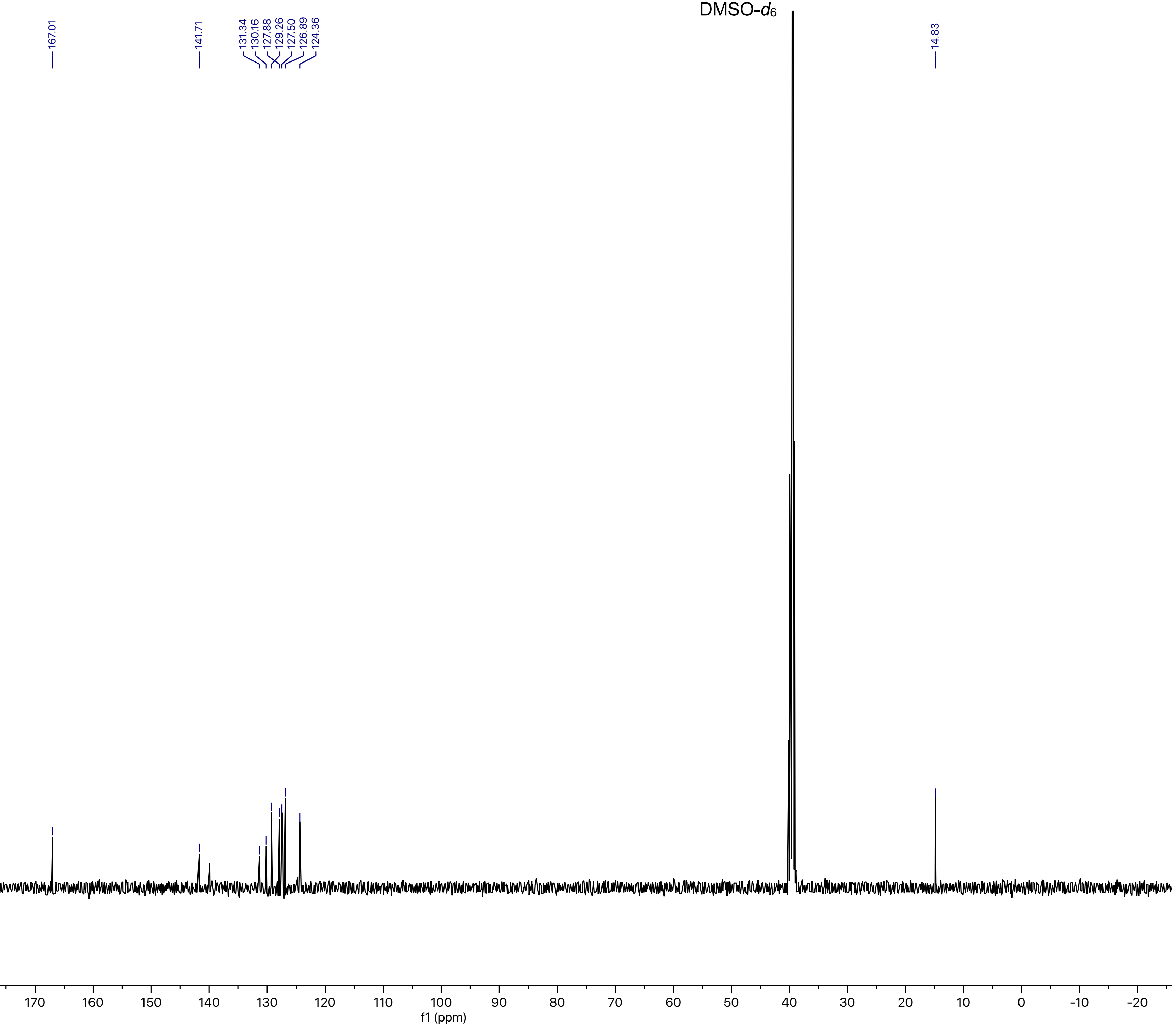

^{1}H NMR: 400 MHz, DMSO-d_6

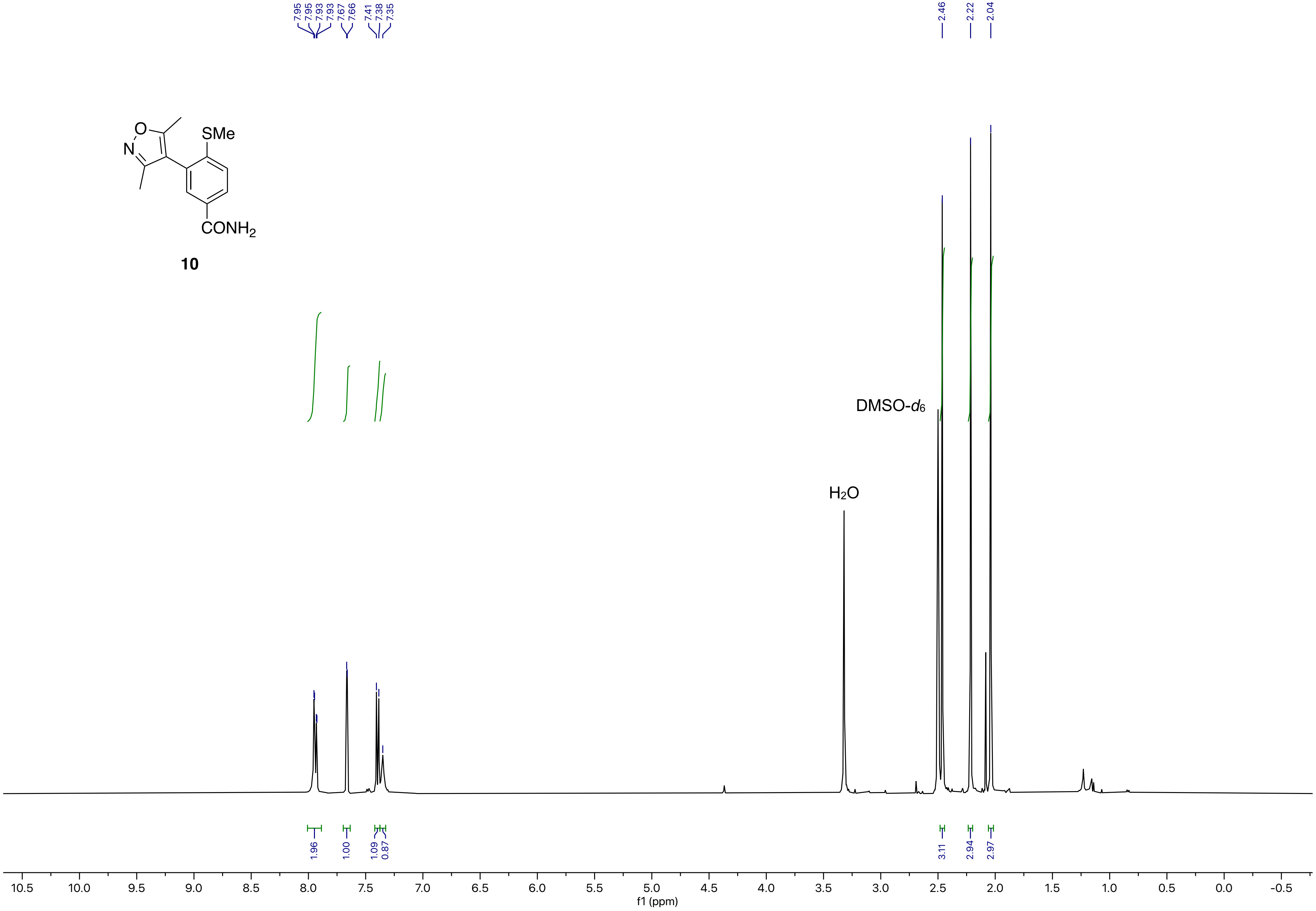

$^{13}C\{^{1}H\}$ NMR: 101 MHz, DMSO-d_6

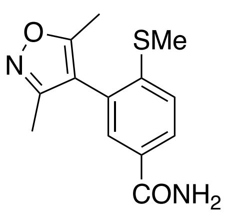

10

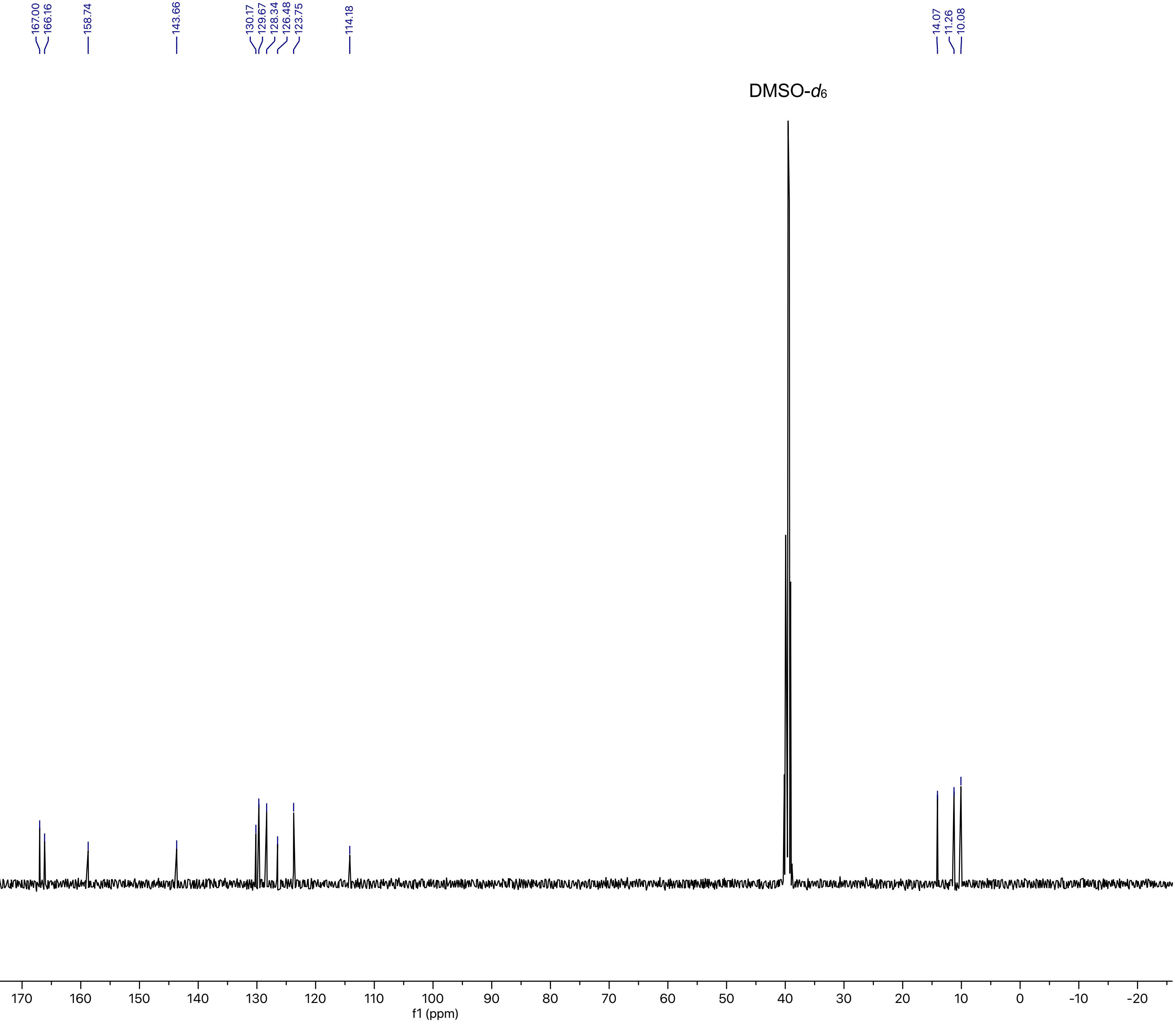

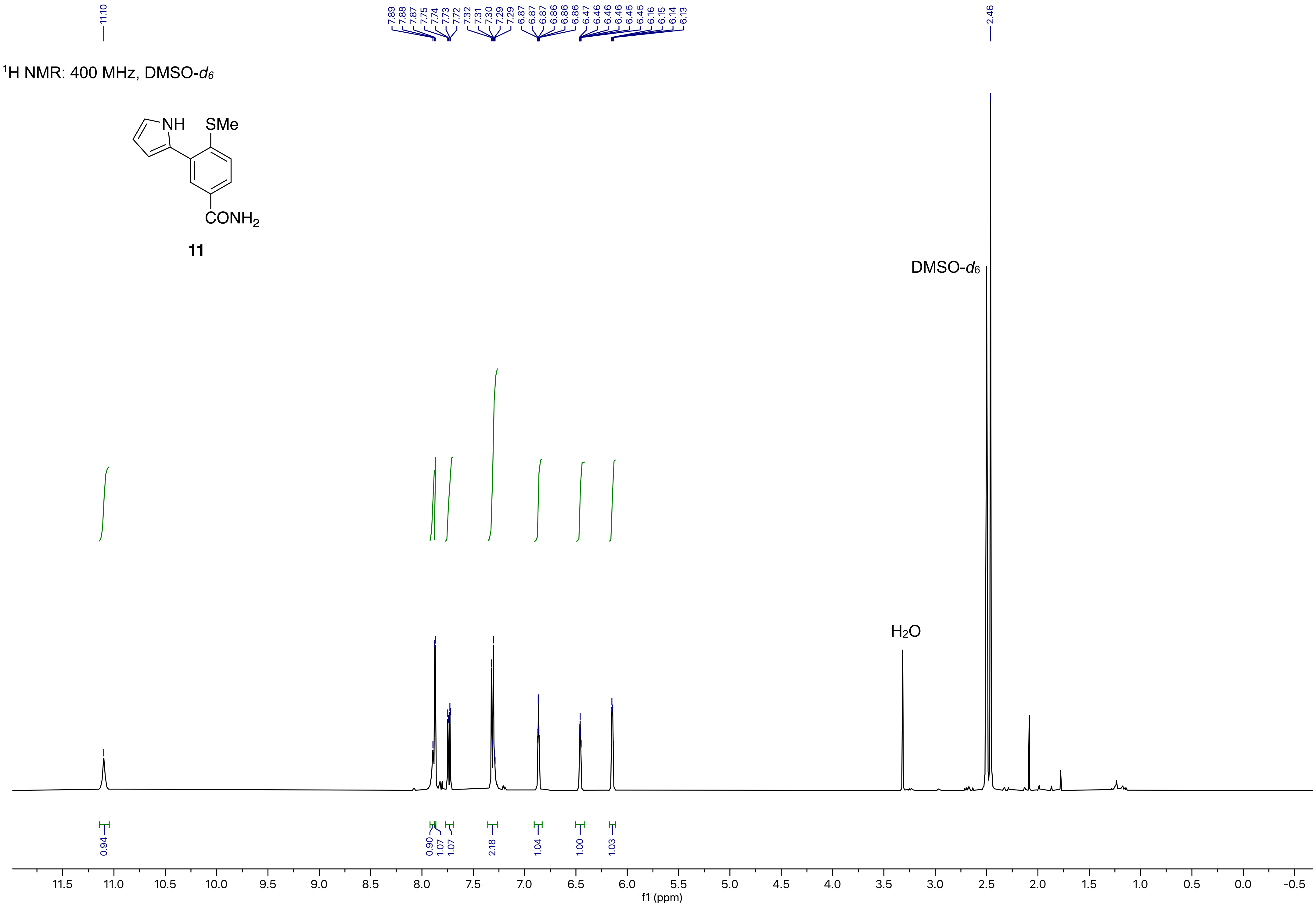

1H NMR: 400 MHz, DMSO-d6
NH
SMe
CONH2
11
11.10
7.89 7.88 7.87 7.75 7.74 7.73 7.72 7.32 7.31 7.30 7.29 7.29 6.87 6.87 6.87 6.86 6.86 6.86 6.47 6.46 6.46 6.46 6.45 6.45 6.16 6.15 6.14 6.13
2.46
DMSO-d6
H2O
0.94
0.90 1.07 1.07
2.18
1.04
1.00
1.03
11.5 11.0 10.5 10.0 9.5 9.0 8.5 8.0 7.5 7.0 6.5 6.0 5.5 5.0 4.5 4.0 3.5 3.0 2.5 2.0 1.5 1.0 0.5 0.0 -0.5
f1 (ppm)

$^{13}C\{^1H\}$ NMR: 101 MHz, DMSO-d_6

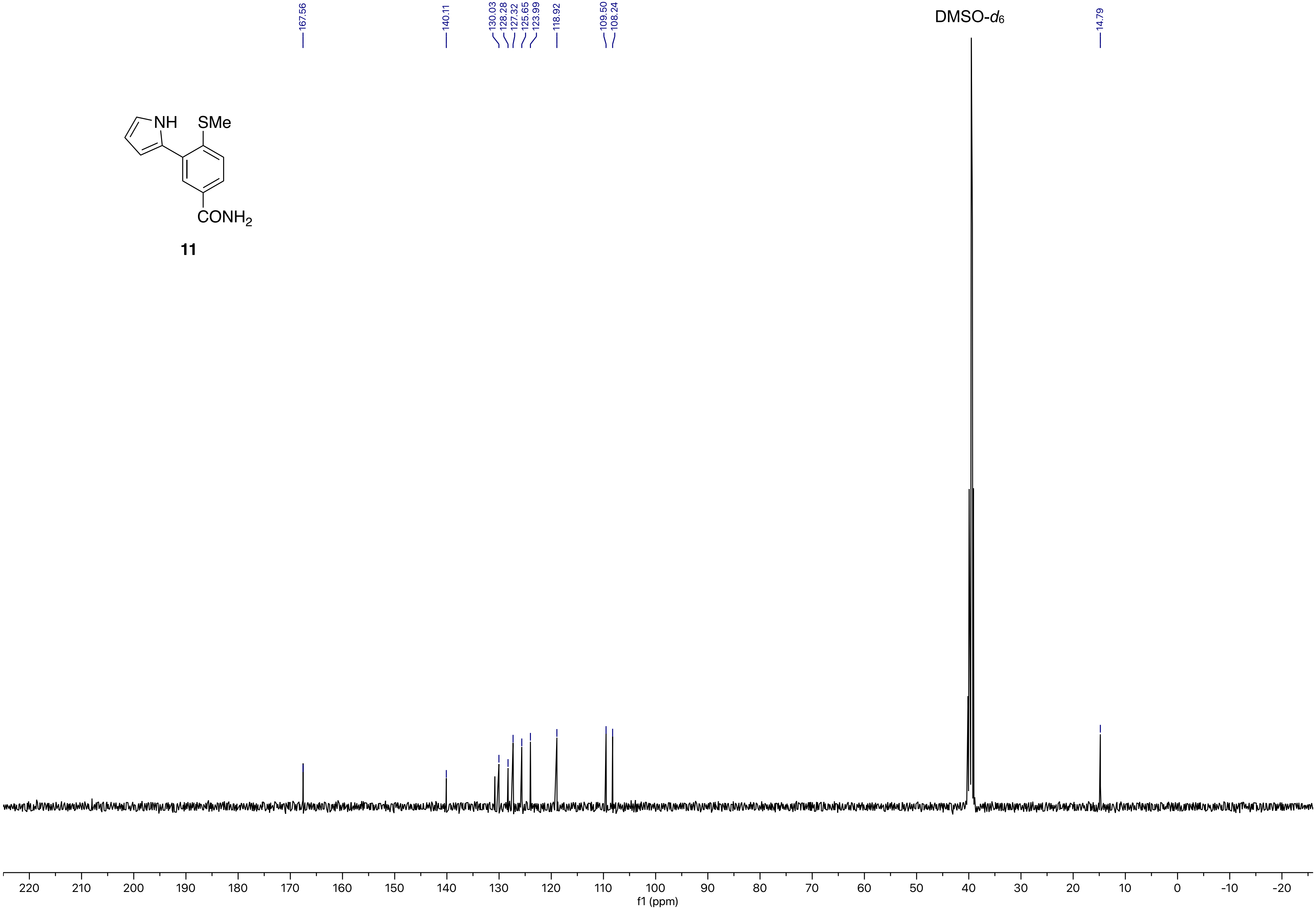

^{1}H NMR: 500 MHz, $CDCl_3$

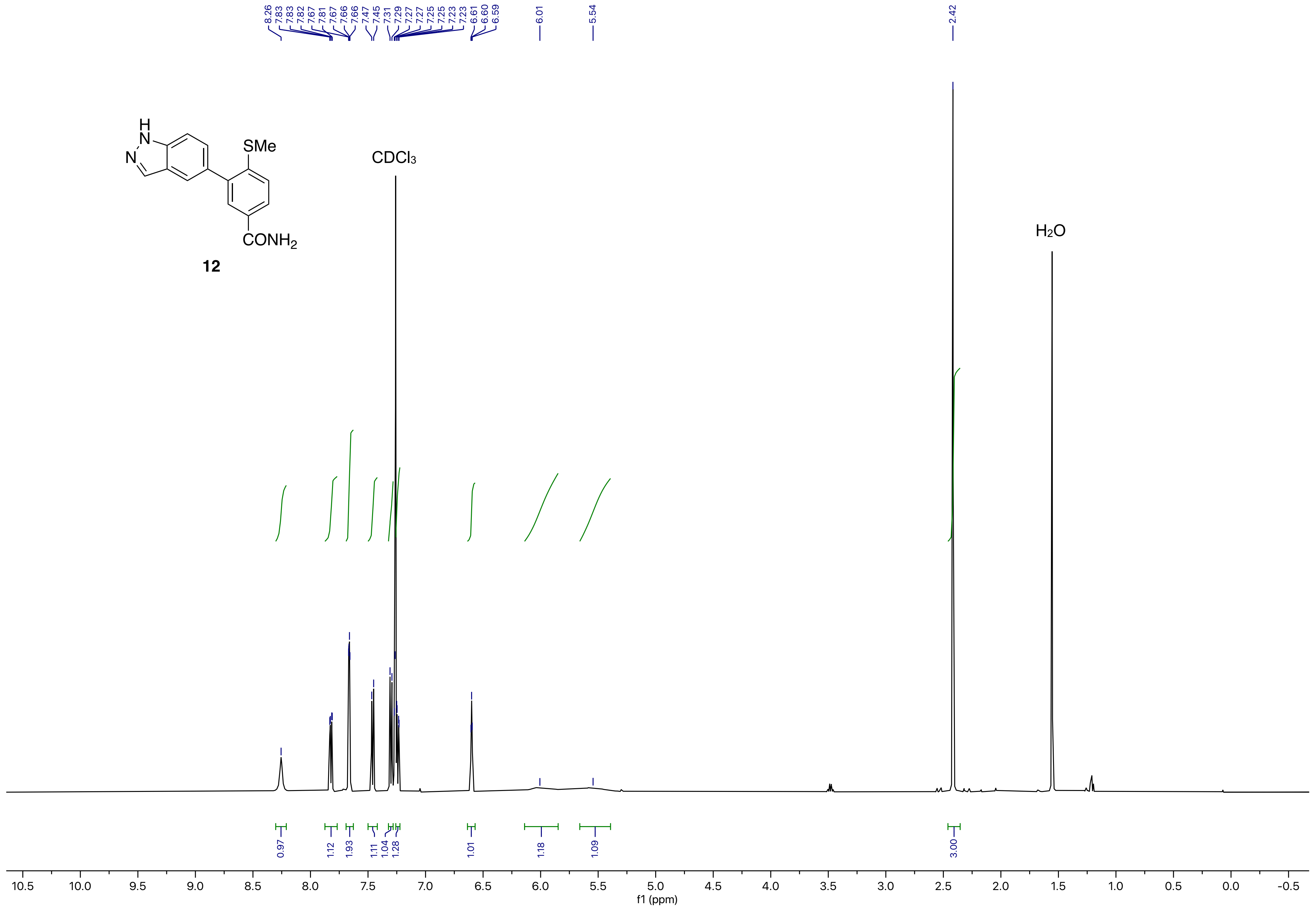

$^{13}C\{^1H\}$ NMR: 126 MHz, $CDCl_3$

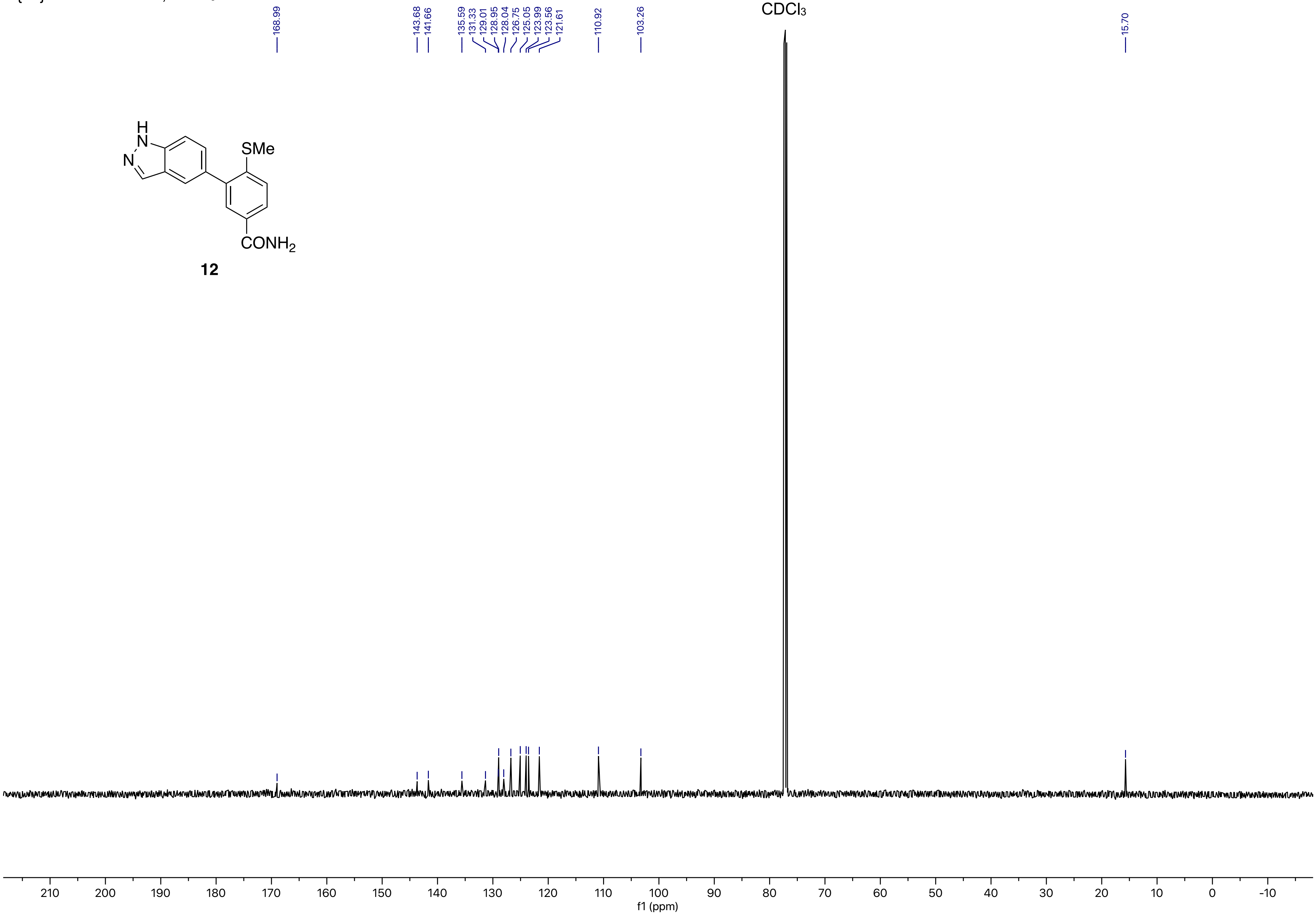

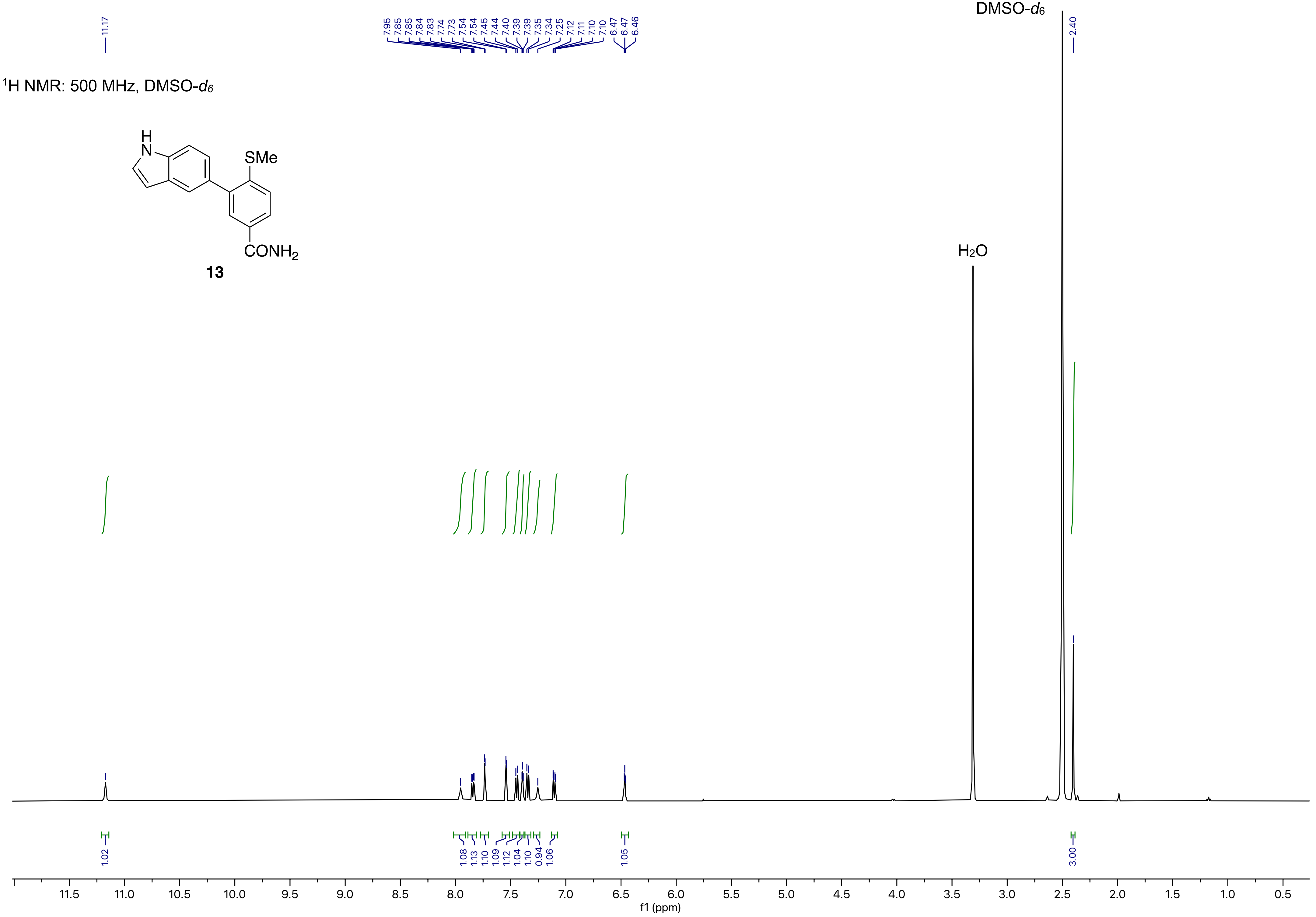

¹H NMR: 500 MHz, DMSO-d6
SMe
CONH2
13
DMSO-d6
H2O
11.17
7.95 7.85 7.85 7.84 7.83 7.74 7.73 7.54 7.54 7.45 7.44 7.40 7.39 7.39 7.35 7.34 7.25 7.12 7.11 7.10 7.10 6.47 6.47 6.46
2.40
1.02
1.08 1.13 1.10 1.09 1.12 1.04 1.10 0.94 1.06
1.05
3.00
11.5 11.0 10.5 10.0 9.5 9.0 8.5 8.0 7.5 7.0 6.5 6.0 5.5 5.0 4.5 4.0 3.5 3.0 2.5 2.0 1.5 1.0 0.5
f1 (ppm)

$^{13}C\{^1H\}$ NMR: 126 MHz, DMSO-d_6

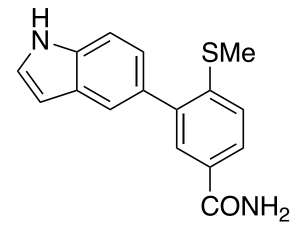

13

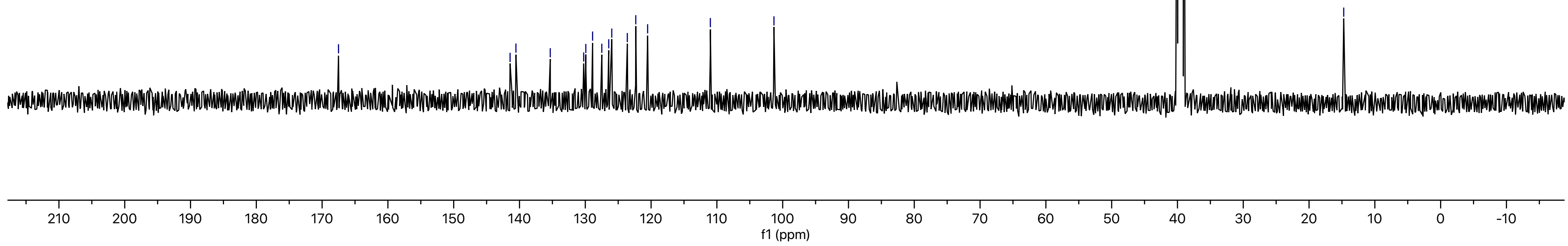

^1H NMR: 500 MHz, DMSO-d_6

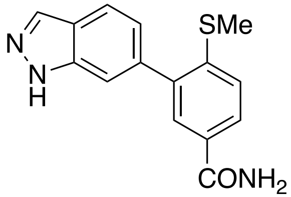

14

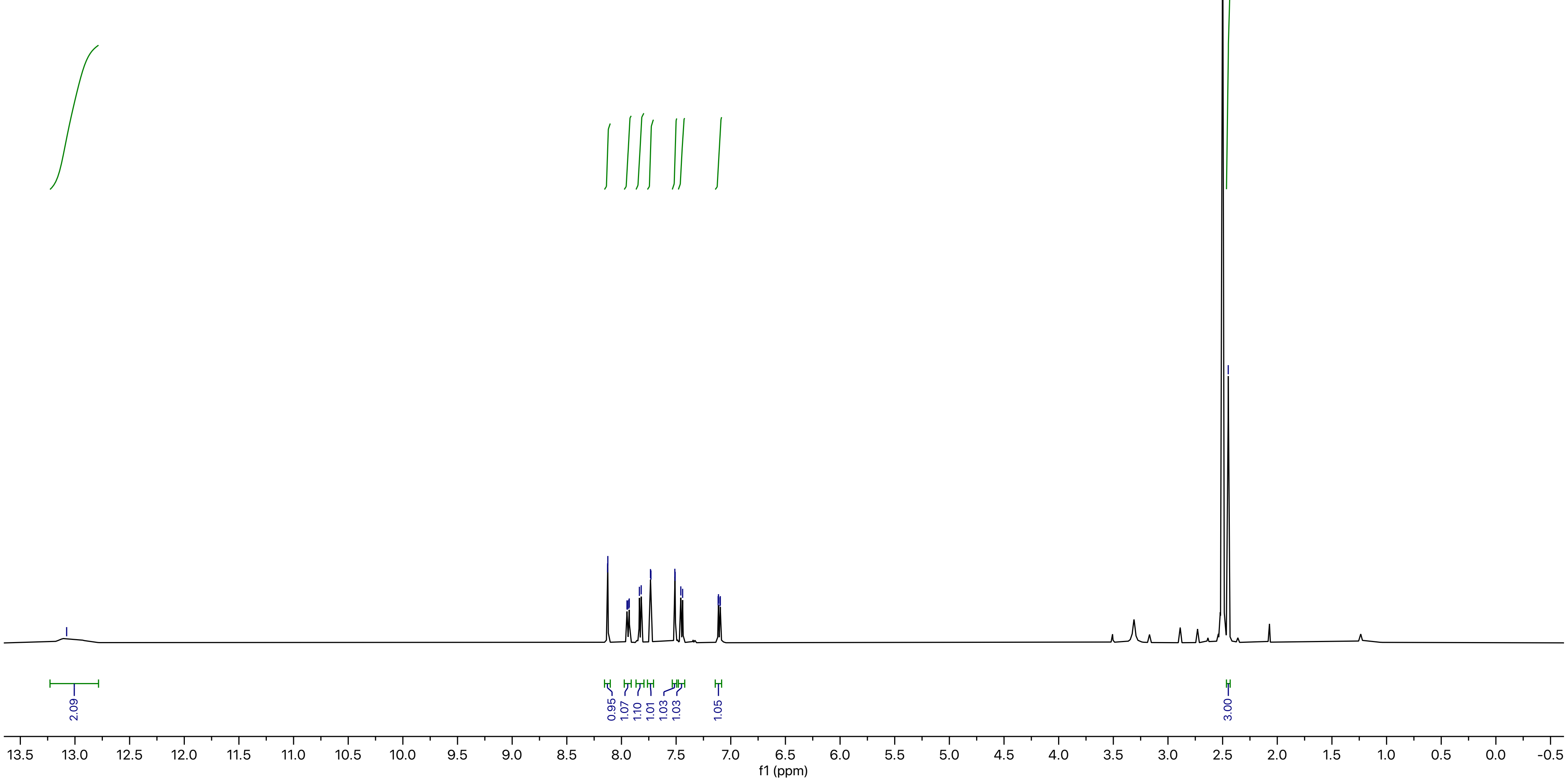

$^{13}C\{^1H\}$ NMR: 101 MHz, DMSO-d_6

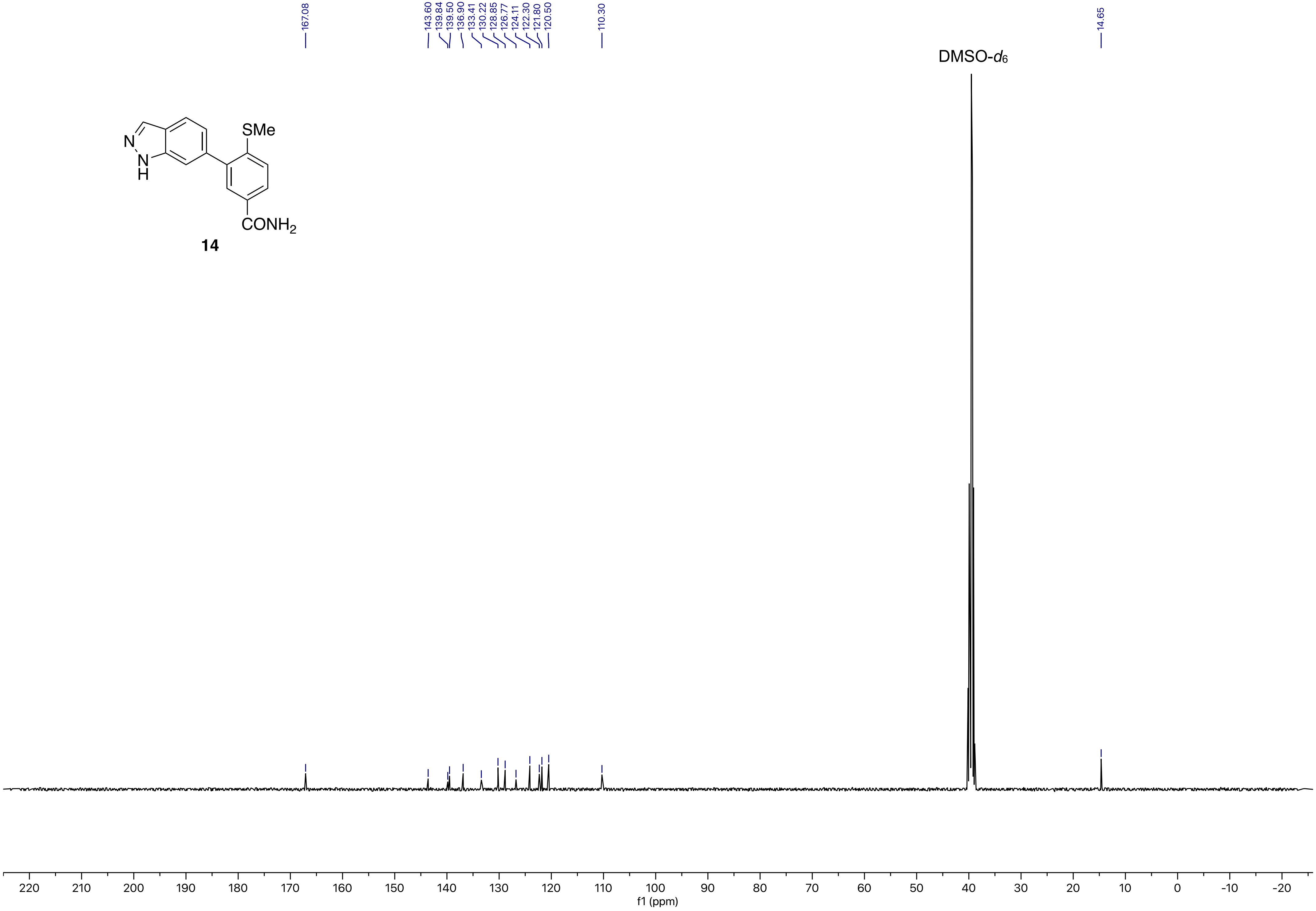

^{1}H NMR: 400 MHz, CD_3OD

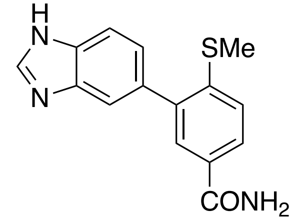

15

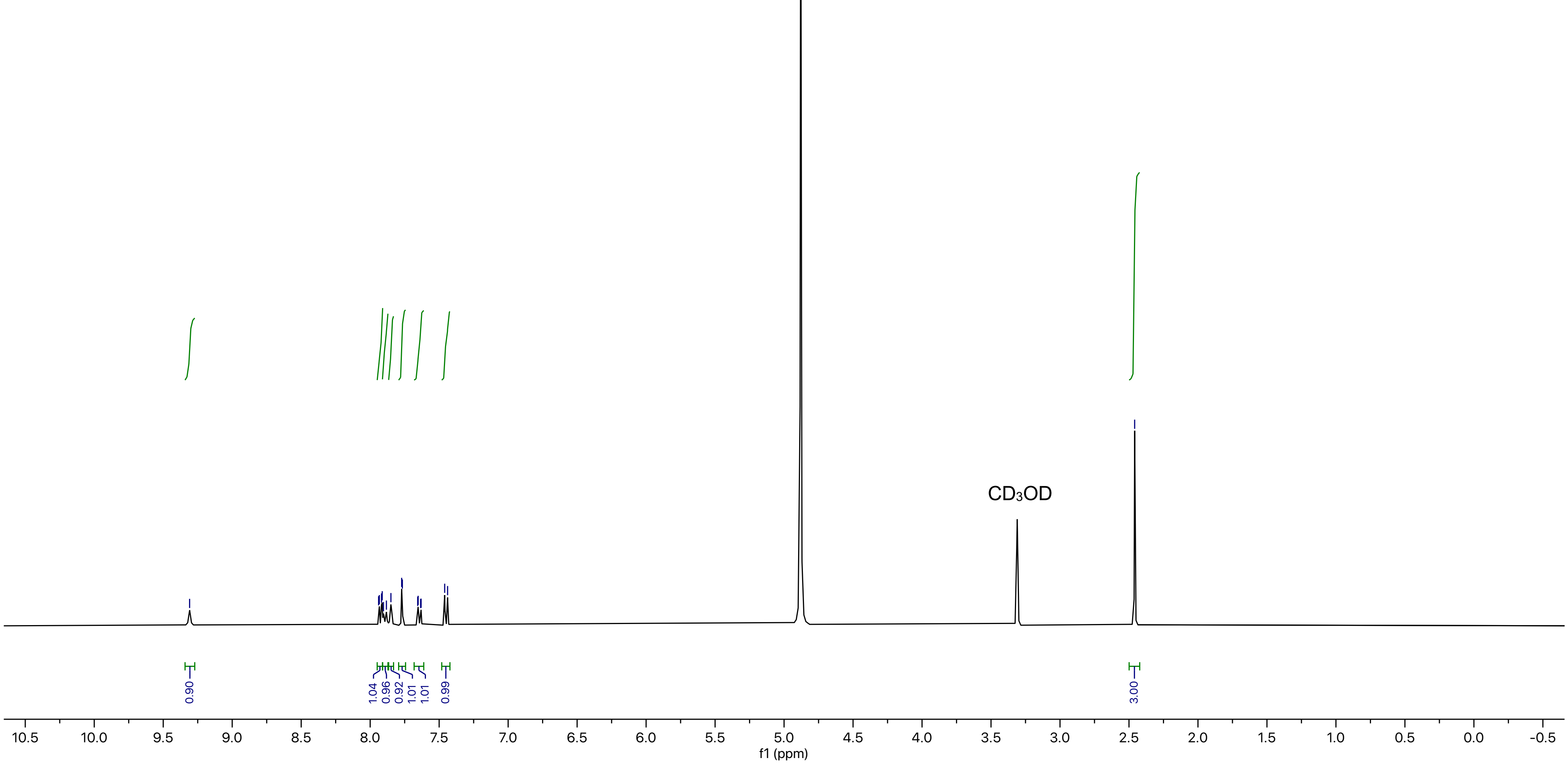

$^{13}C\{^1H\}$ NMR: 101 MHz, CD_3OD

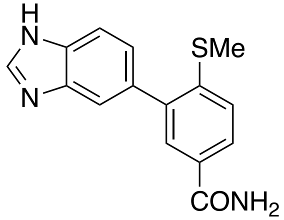

15

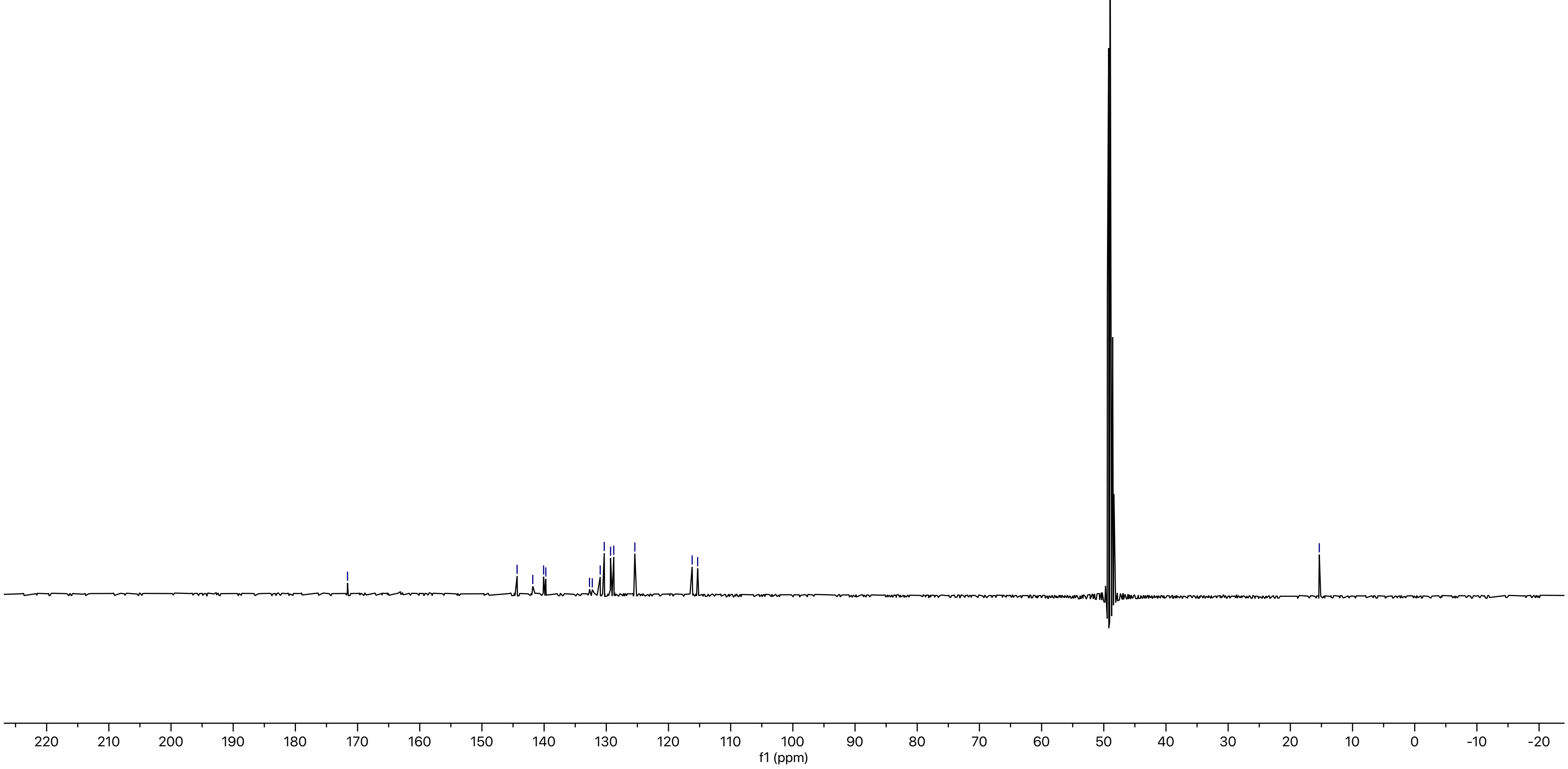

^{1}H NMR: 400 MHz, DMSO-d_6

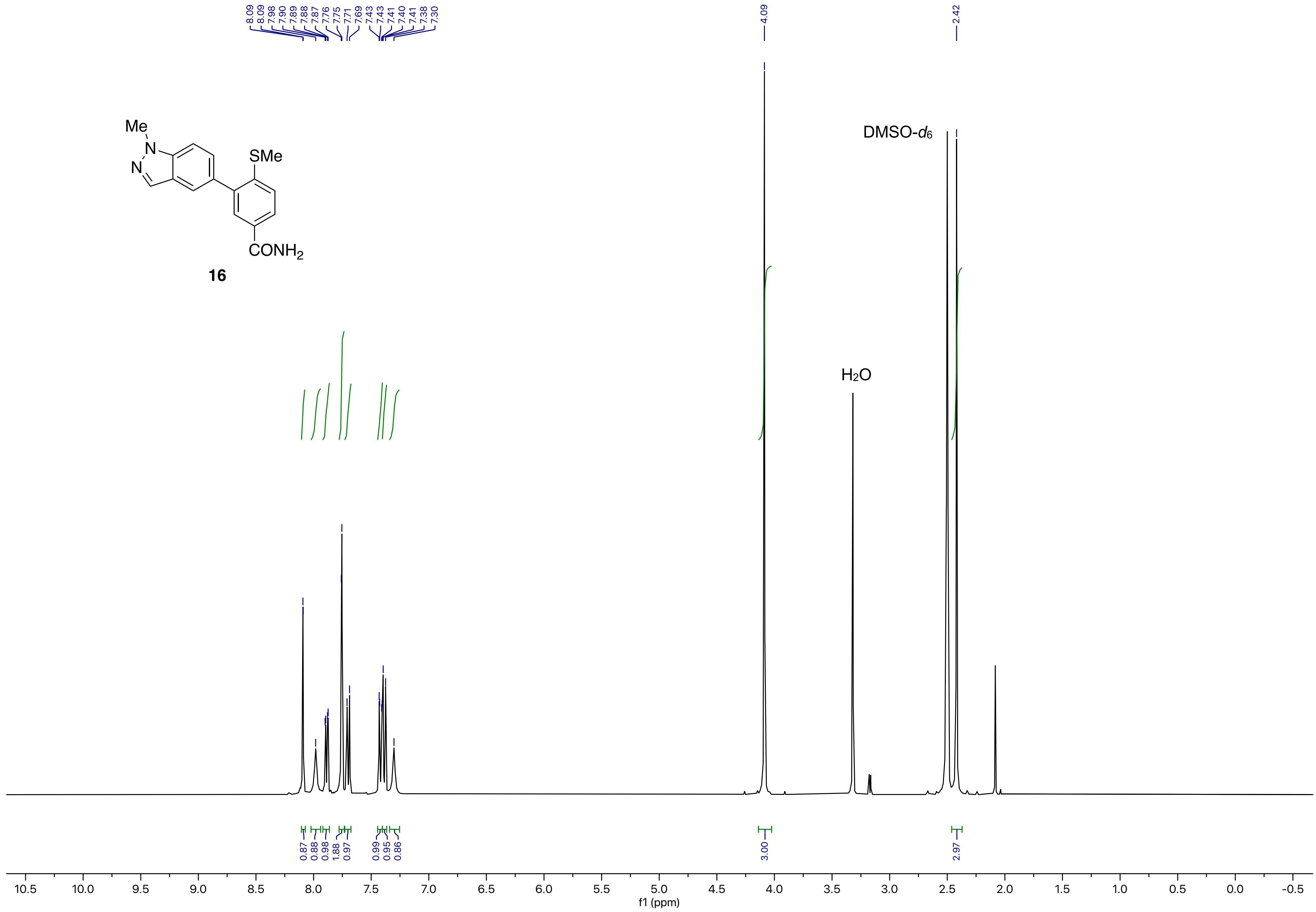

$^{13}C\{^1H\}$ NMR: 101 MHz, DMSO-d_6

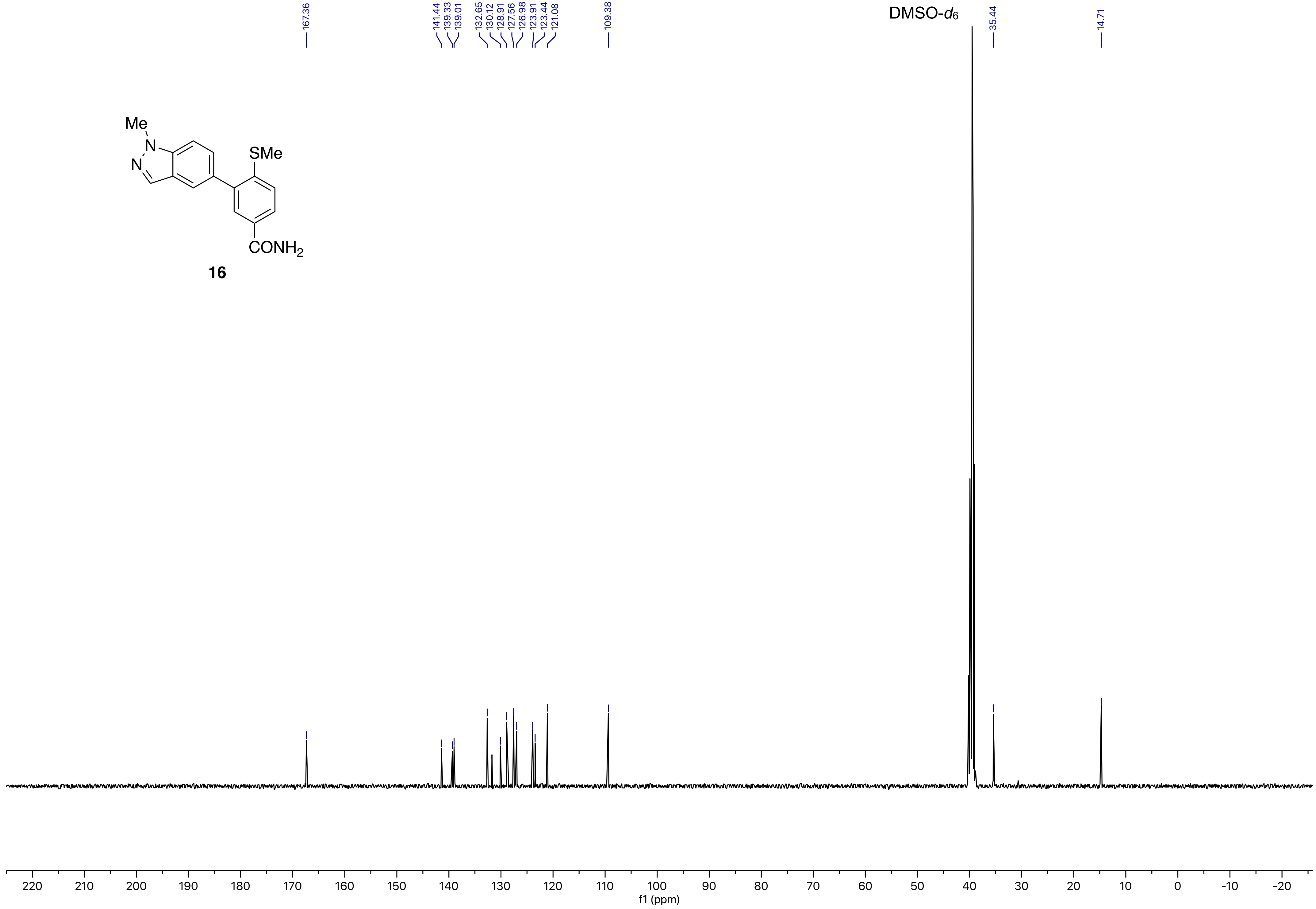

^1H NMR: 400 MHz, DMSO-d_6

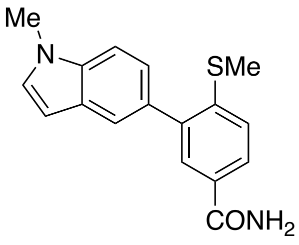

17

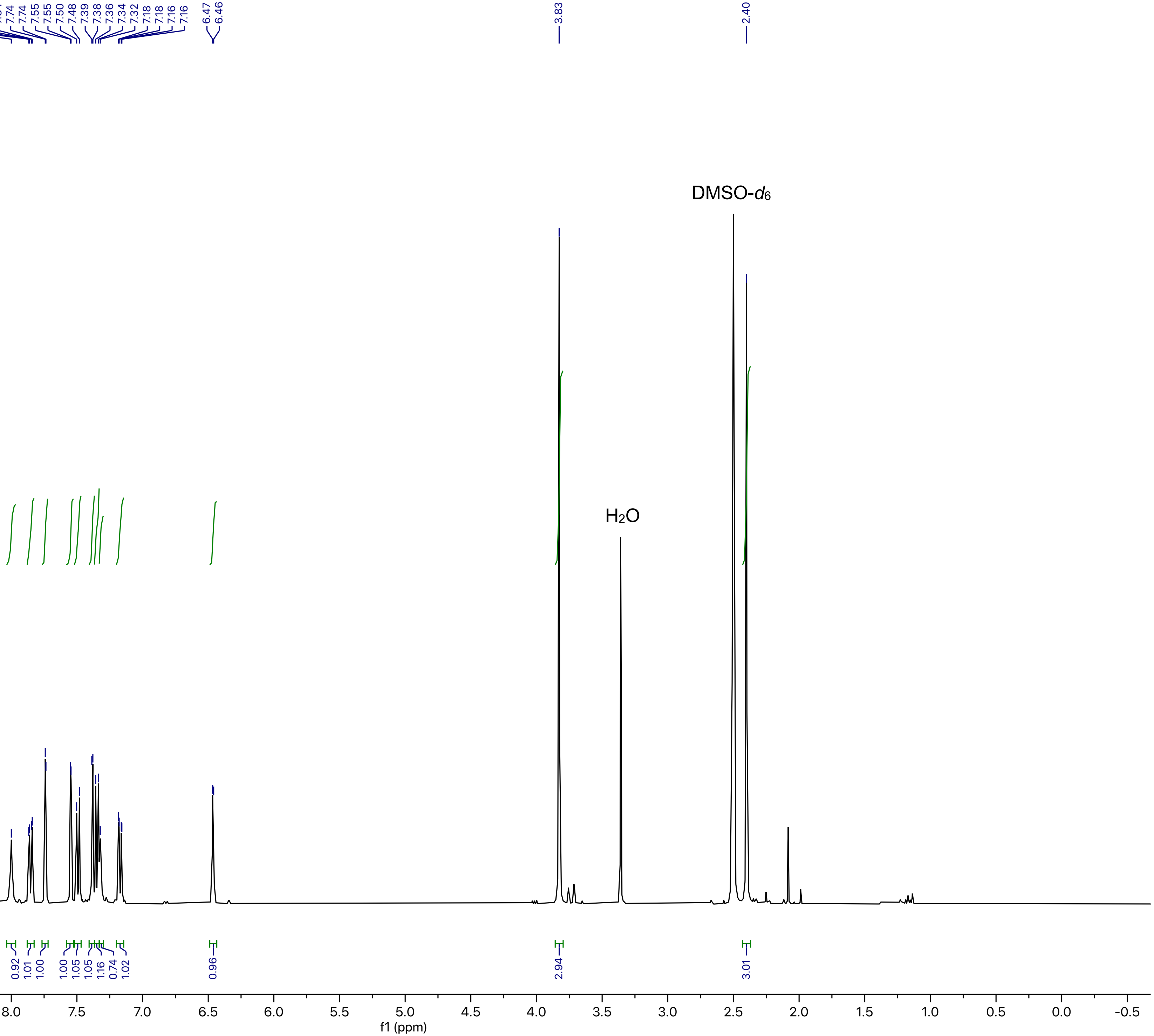

$^{13}C\{^1H\}$ NMR: 101 MHz, DMSO-d_6

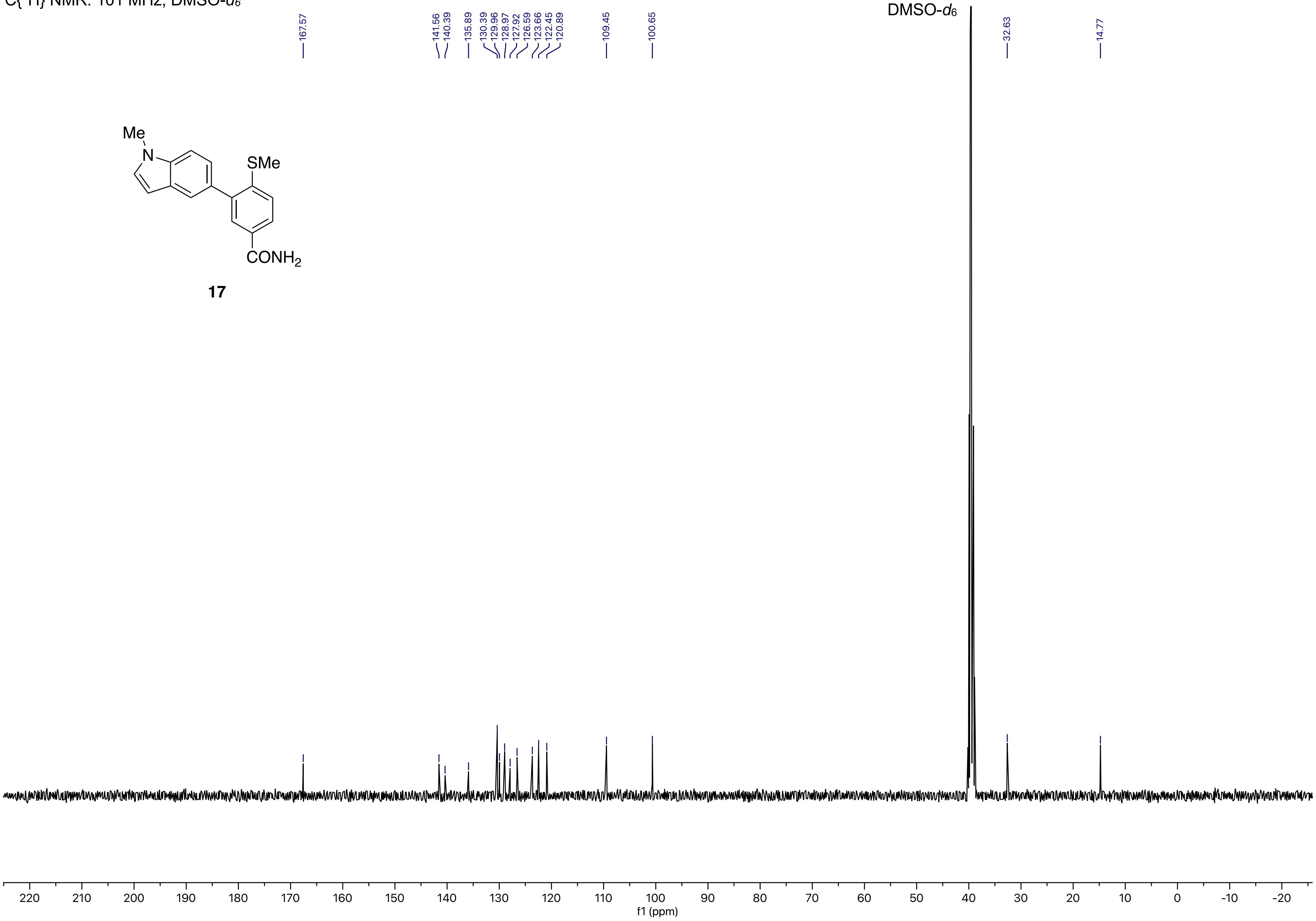

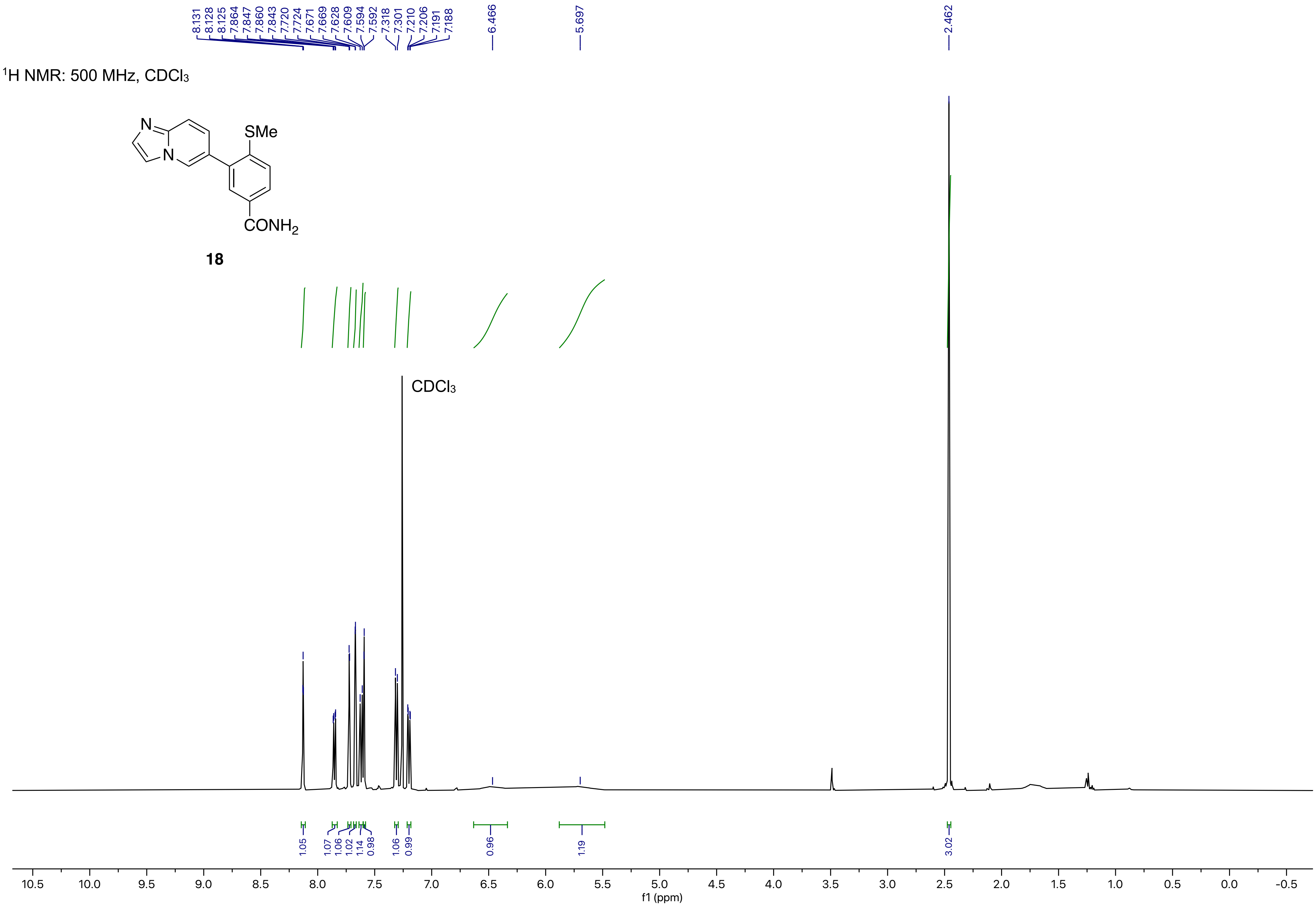

8.131
8.128
8.125
7.864
7.847
7.860
7.843
7.724
7.720
7.671
7.669
7.628
7.609
7.594
7.592
7.318
7.301
7.210
7.206
7.191
7.188
6.466
5.697
2.462
^{1}H NMR: 500 MHz, CDCl3
SMe
N
N
CONH2
18
CDCl3
1.05
1.07
1.06
1.02
1.14
0.98
1.06
0.99
0.96
1.19
3.02
10.5
10.0
9.5
9.0
8.5
8.0
7.5
7.0
6.5
6.0
5.5
5.0
4.5
4.0
3.5
3.0
2.5
2.0
1.5
1.0
0.5
0.0
-0.5
f1 (ppm)

$^{13}C\{^{1}H\}$ NMR: 126 MHz, $CDCl_3$

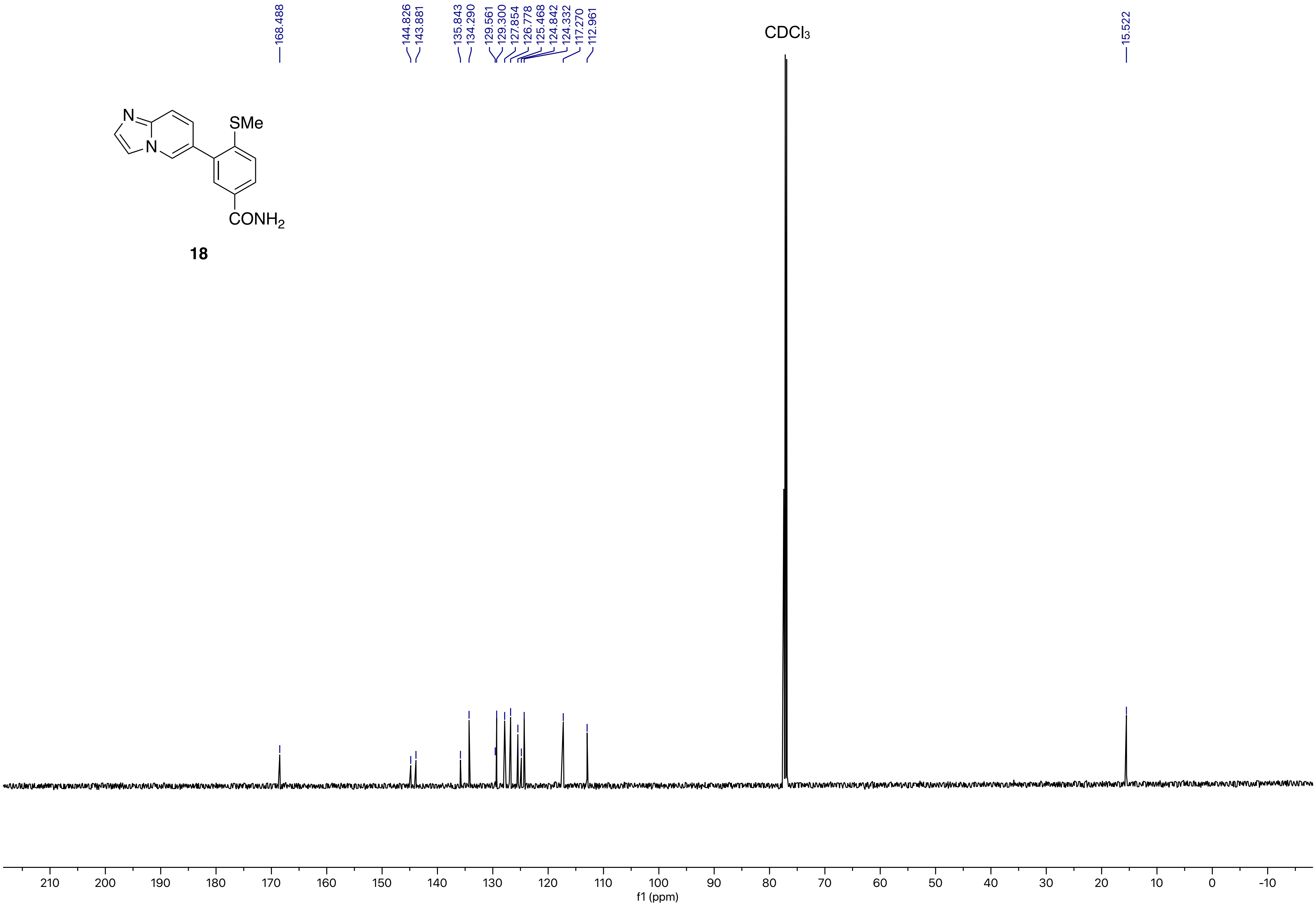

^{1}H NMR: 400 MHz, DMSO-d_6

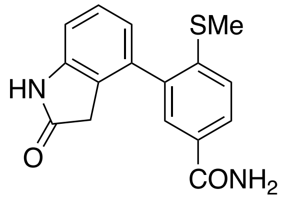

19

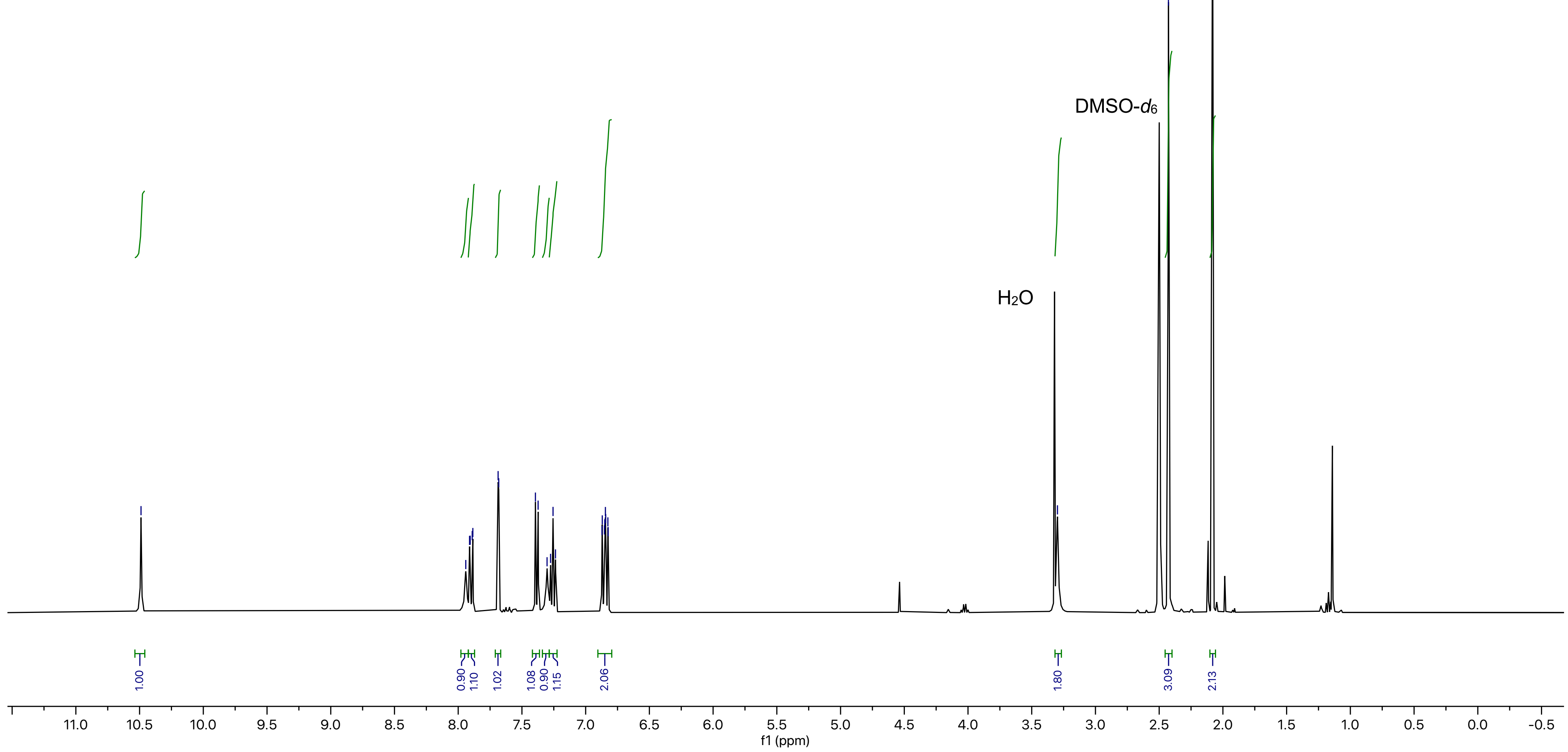

$^{13}C\{^{1}H\}$ NMR: 101 MHz, DMSO-d_6

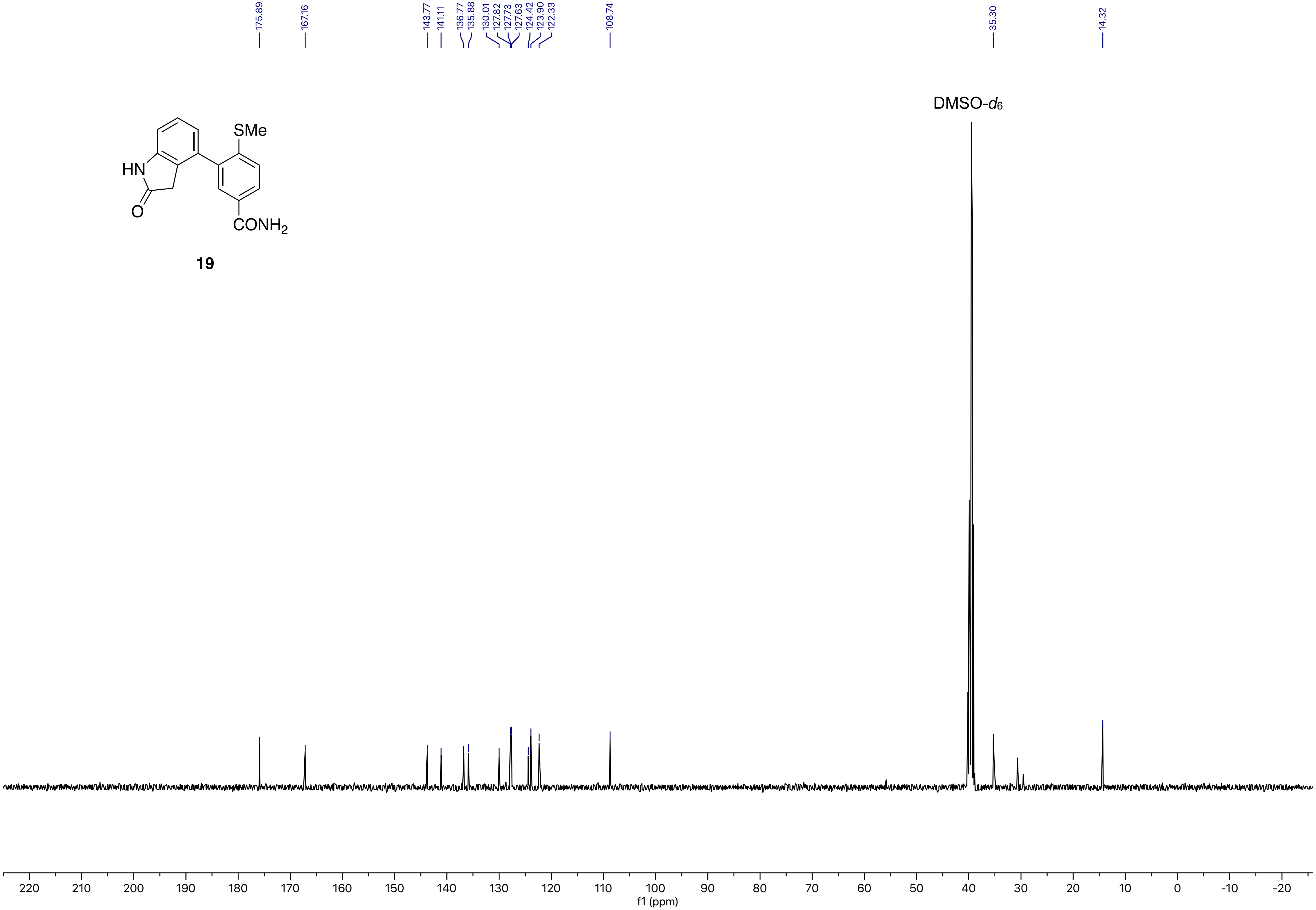

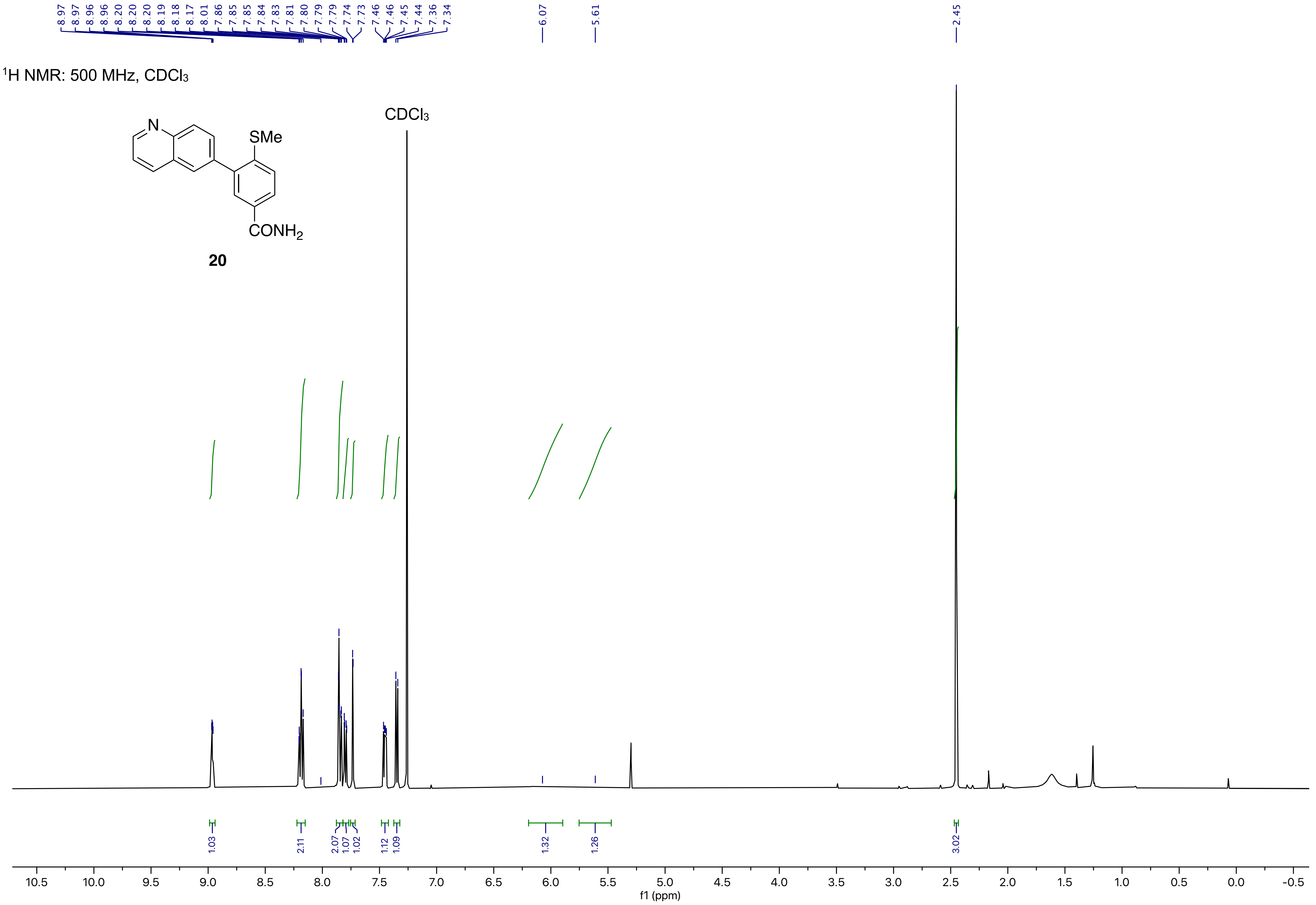
8.97 8.97 8.96 8.96 8.20 8.20 8.20 8.19 8.18 8.17 8.01 7.86 7.85 7.85 7.84 7.83 7.81 7.80 7.79 7.79 7.74 7.73 7.46 7.46 7.45 7.44 7.36 7.34
6.07
5.61
2.45
^{1}H NMR: 500 MHz, CDCl$_3$
N
SMe
CONH$_2$
20
CDCl$_3$
1.03 2.11 2.07 1.07 1.02 1.12 1.09 1.32 1.26 3.02
10.5 10.0 9.5 9.0 8.5 8.0 7.5 7.0 6.5 6.0 5.5 5.0 4.5 4.0 3.5 3.0 2.5 2.0 1.5 1.0 0.5 0.0 -0.5
f1 (ppm)

$^{13}C\{^{1}H\}$ NMR: 126 MHz, $CDCl_3$

20

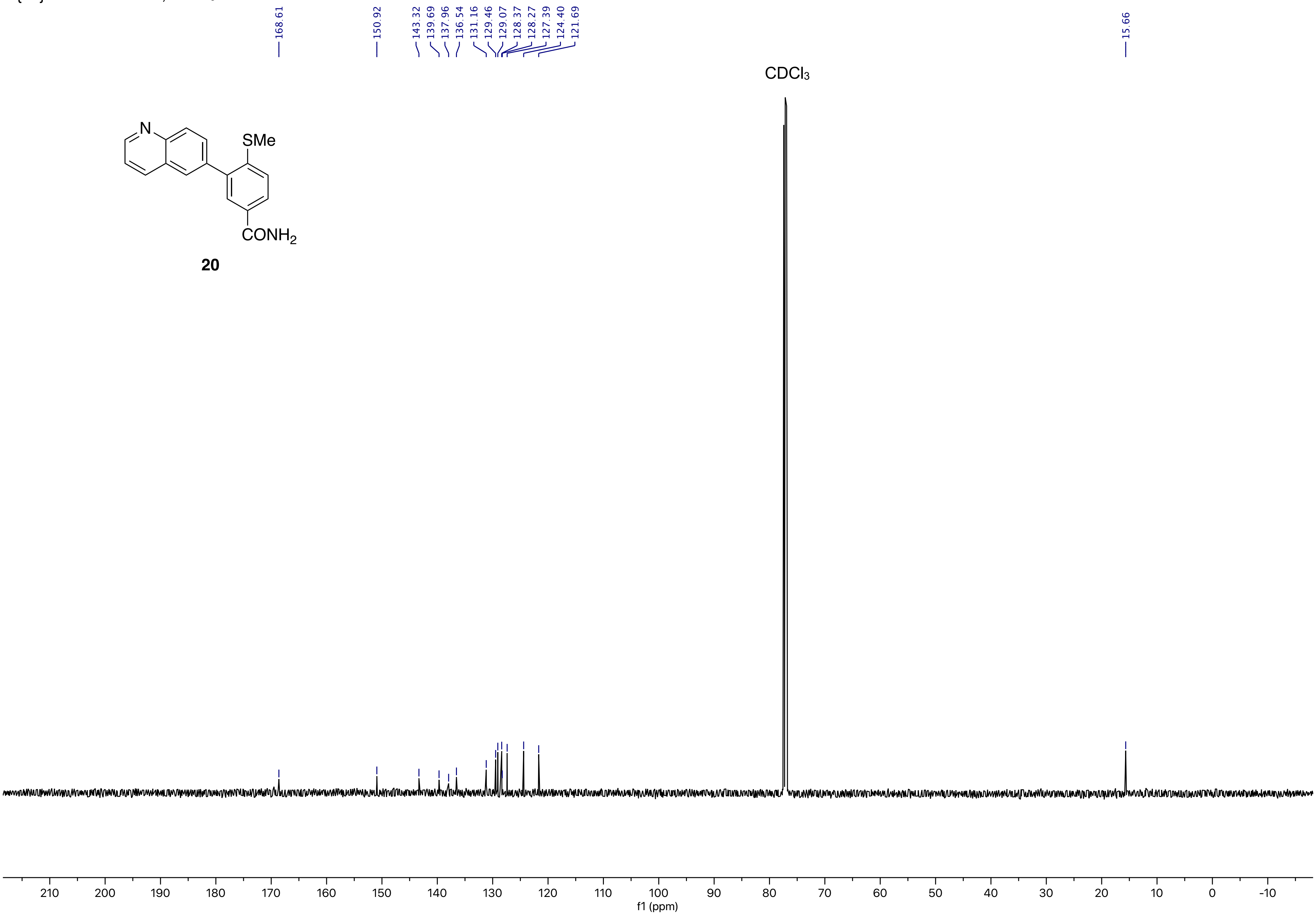

^1H NMR: 400 MHz, DMSO-d_6

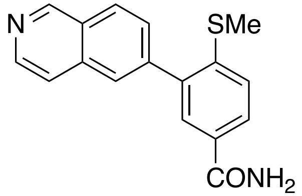

21

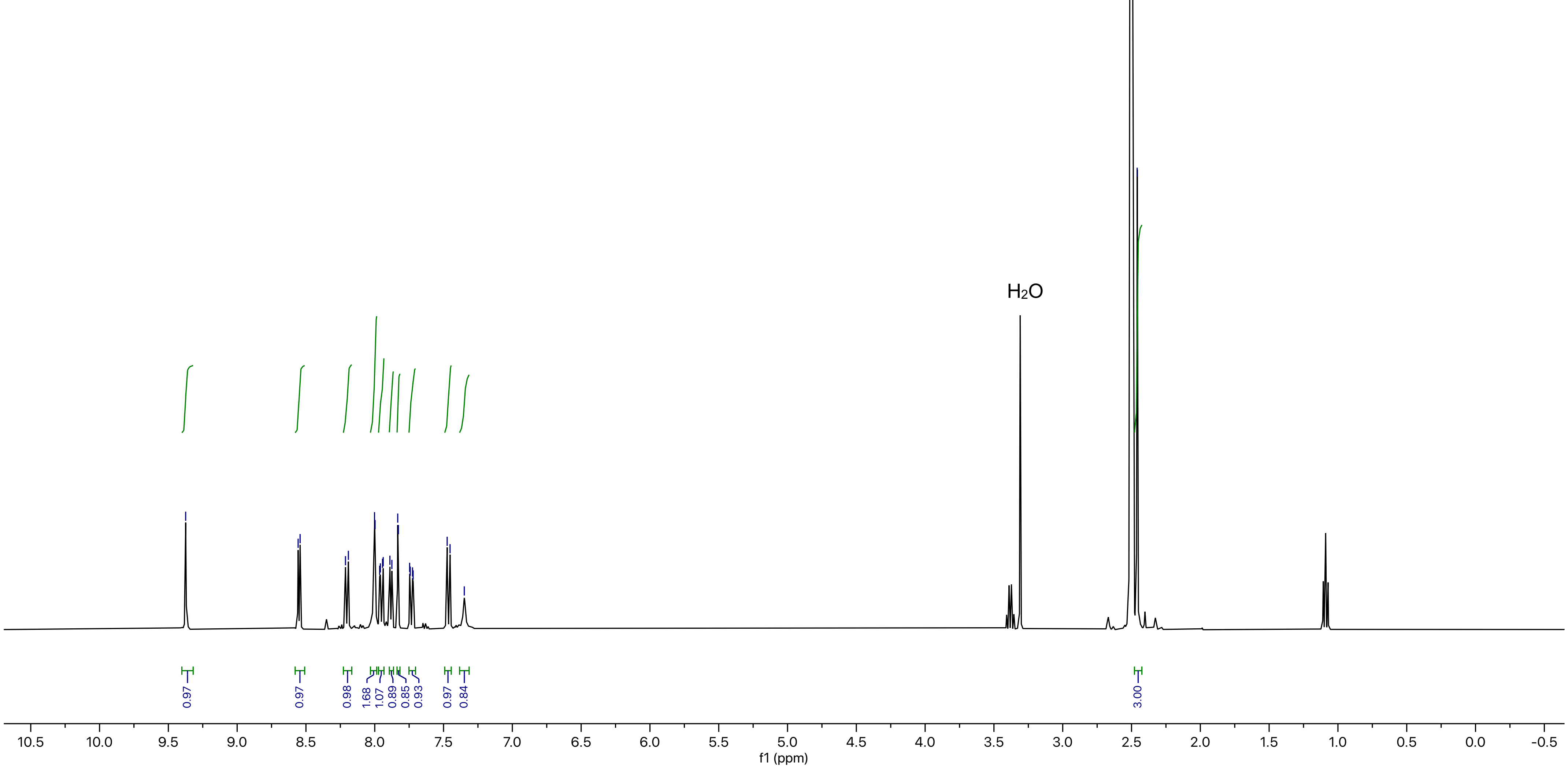

$^{13}C\{^1H\}$ NMR: 101 MHz, DMSO-d_6

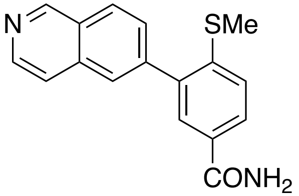

21

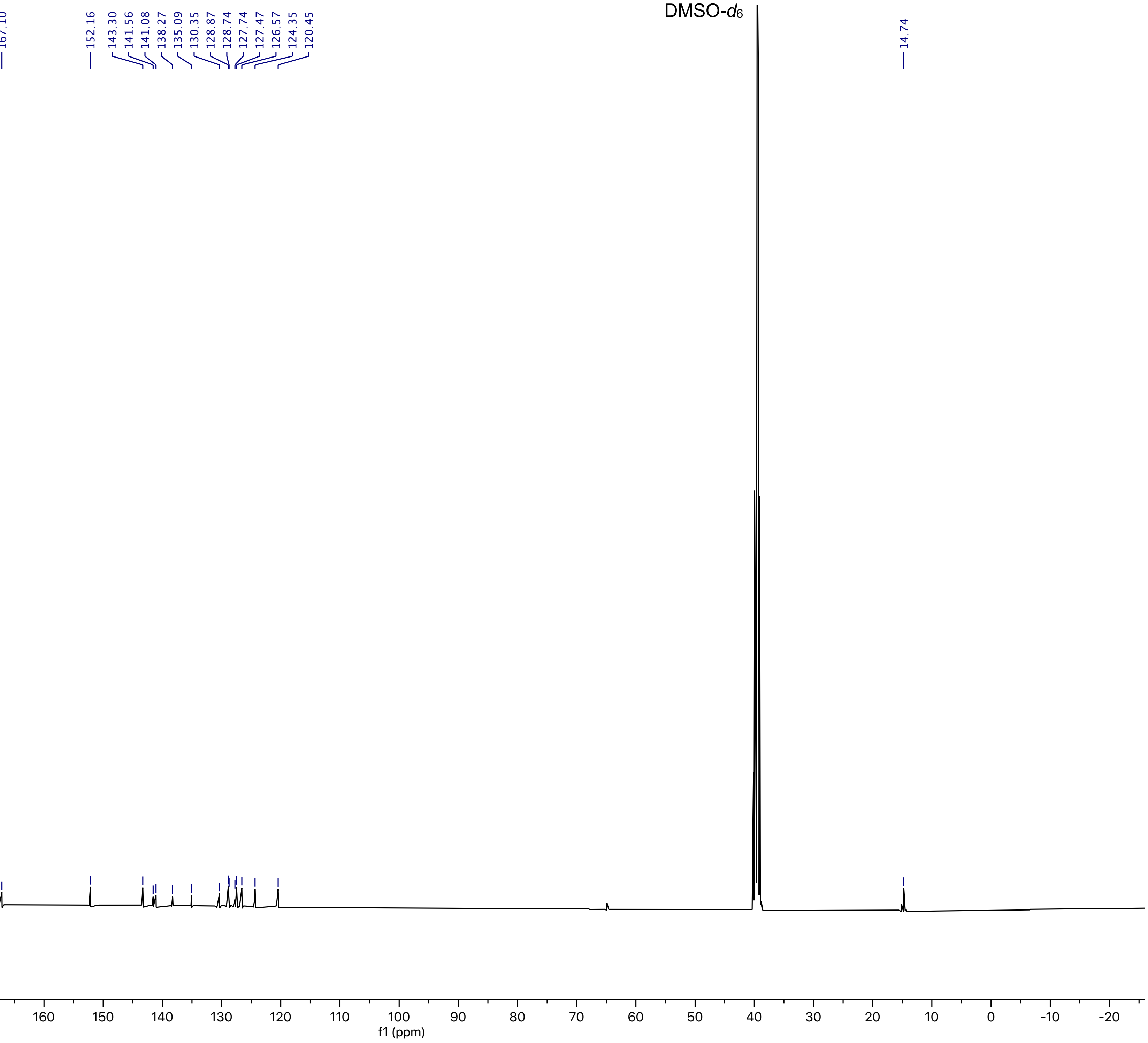

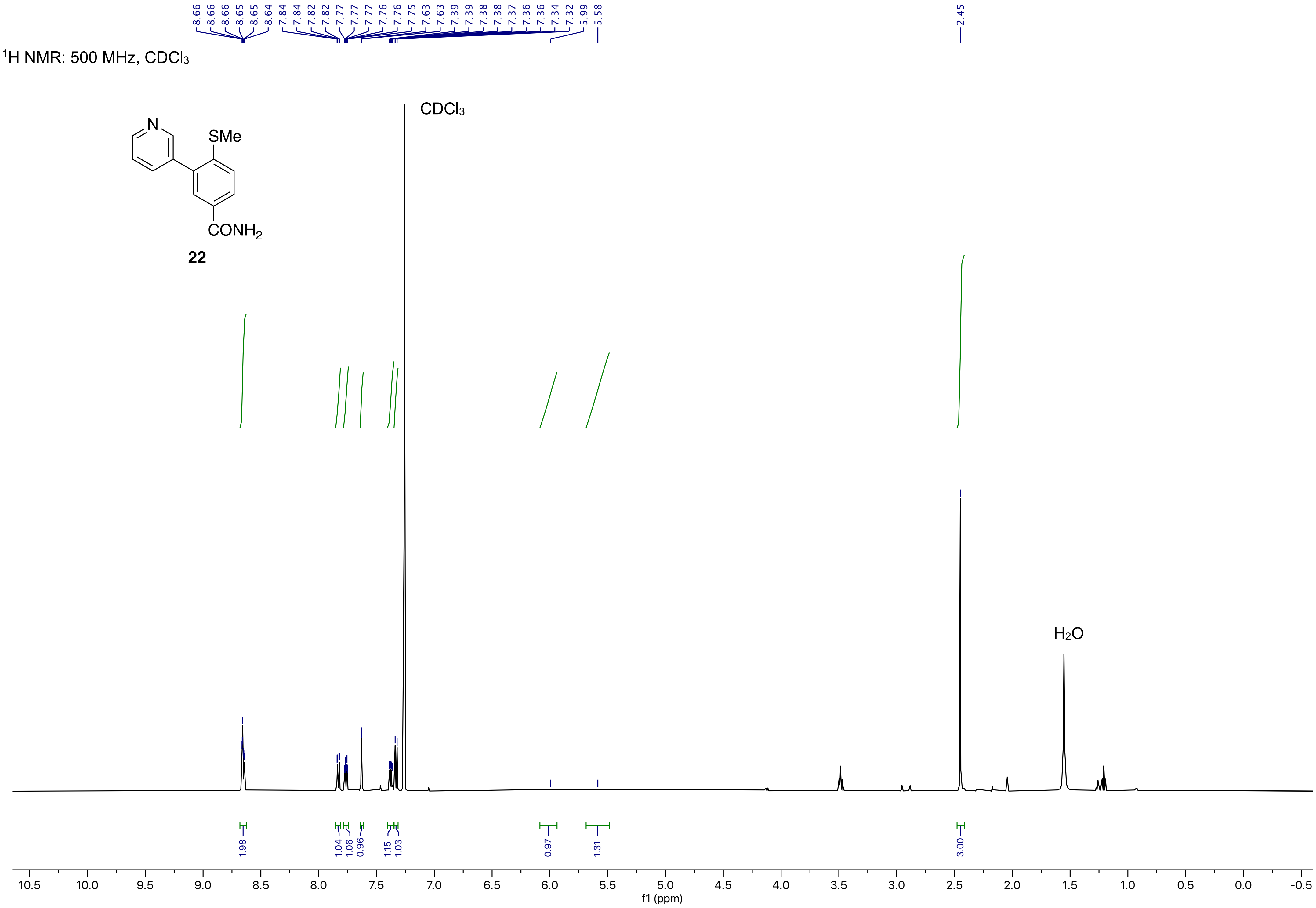

8.66 8.66 8.66 8.65 8.65 8.64 7.84 7.84 7.82 7.82 7.77 7.77 7.77 7.76 7.76 7.75 7.63 7.63 7.39 7.39 7.38 7.38 7.37 7.36 7.36 7.34 7.32 5.99 5.58
2.45
^{1}H NMR: 500 MHz, $CDCl_3$
SMe
N
$CONH_2$
22
$CDCl_3$
H_2O
1.98
1.04 1.06 0.96
1.15 1.03
0.97
1.31
3.00
10.5 10.0 9.5 9.0 8.5 8.0 7.5 7.0 6.5 6.0 5.5 5.0 4.5 4.0 3.5 3.0 2.5 2.0 1.5 1.0 0.5 0.0 -0.5
f1 (ppm)

$^{13}C\{^1H\}$ NMR: 126 MHz, $CDCl_3$

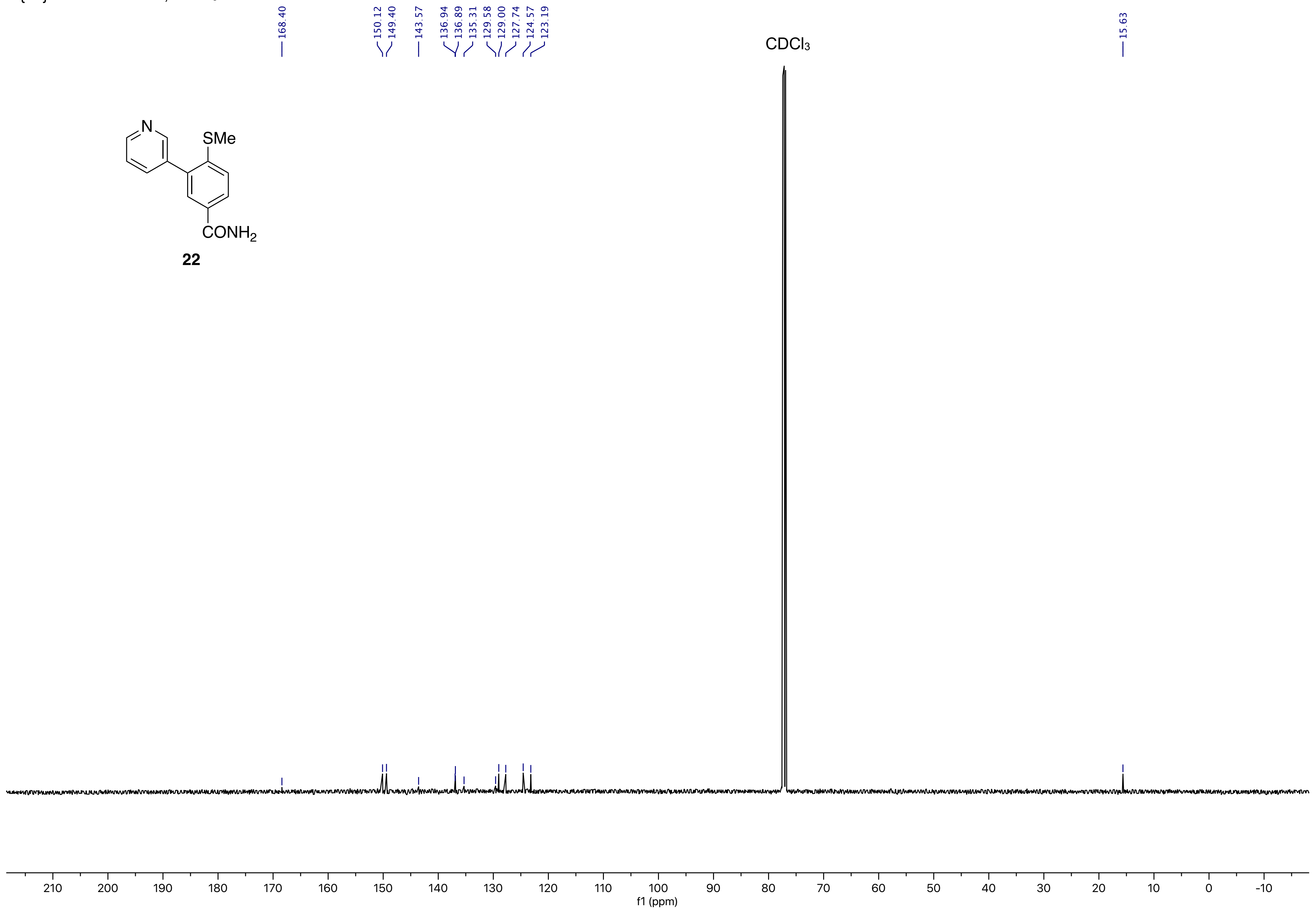

^{1}H NMR: 500 MHz, $CDCl_3$

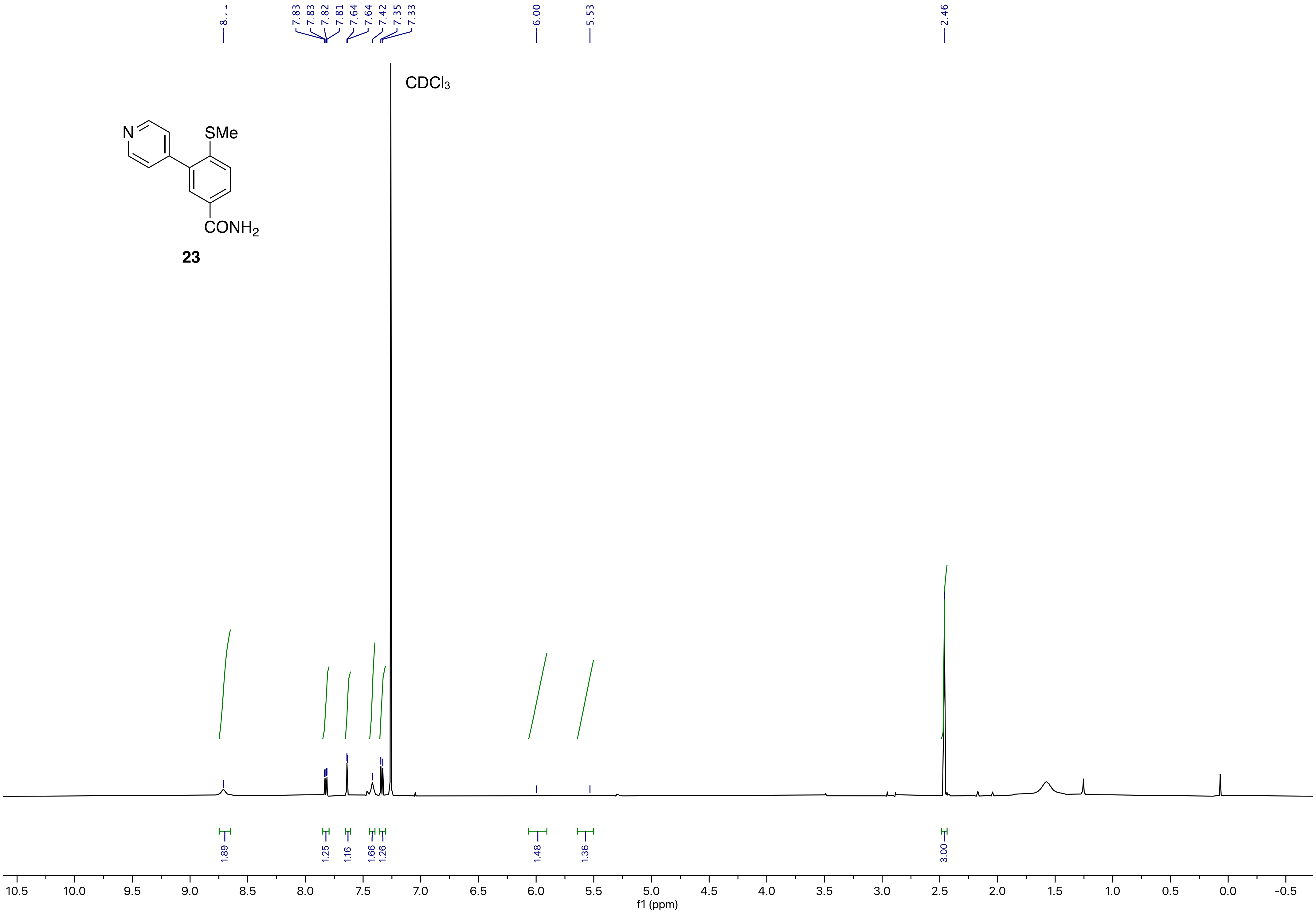

23

$^{13}C\{^1H\}$ NMR: 126 MHz, $CDCl_3$

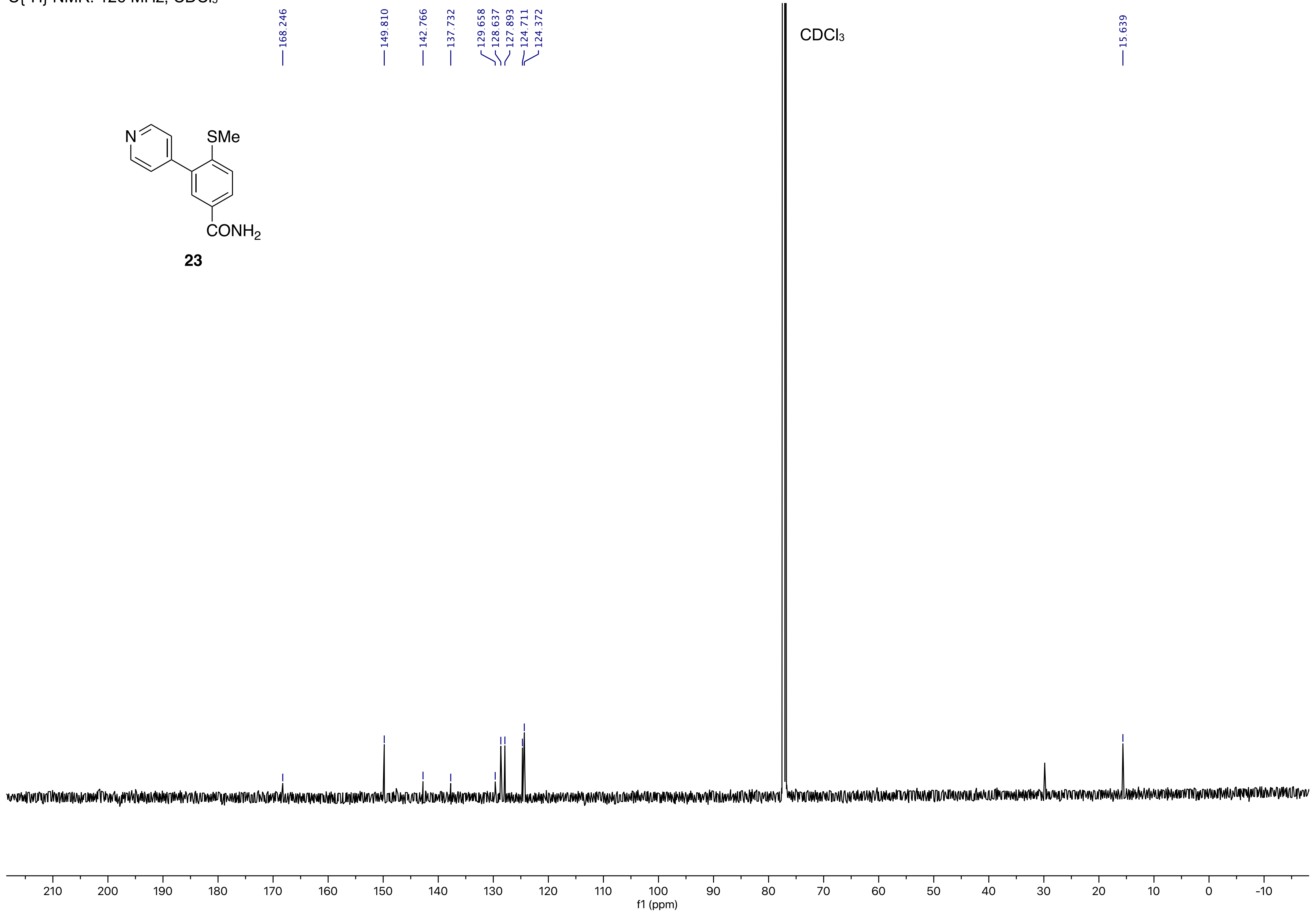

^{1}H NMR: 400 MHz, DMSO-d_6

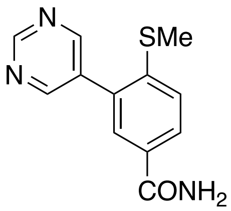

24

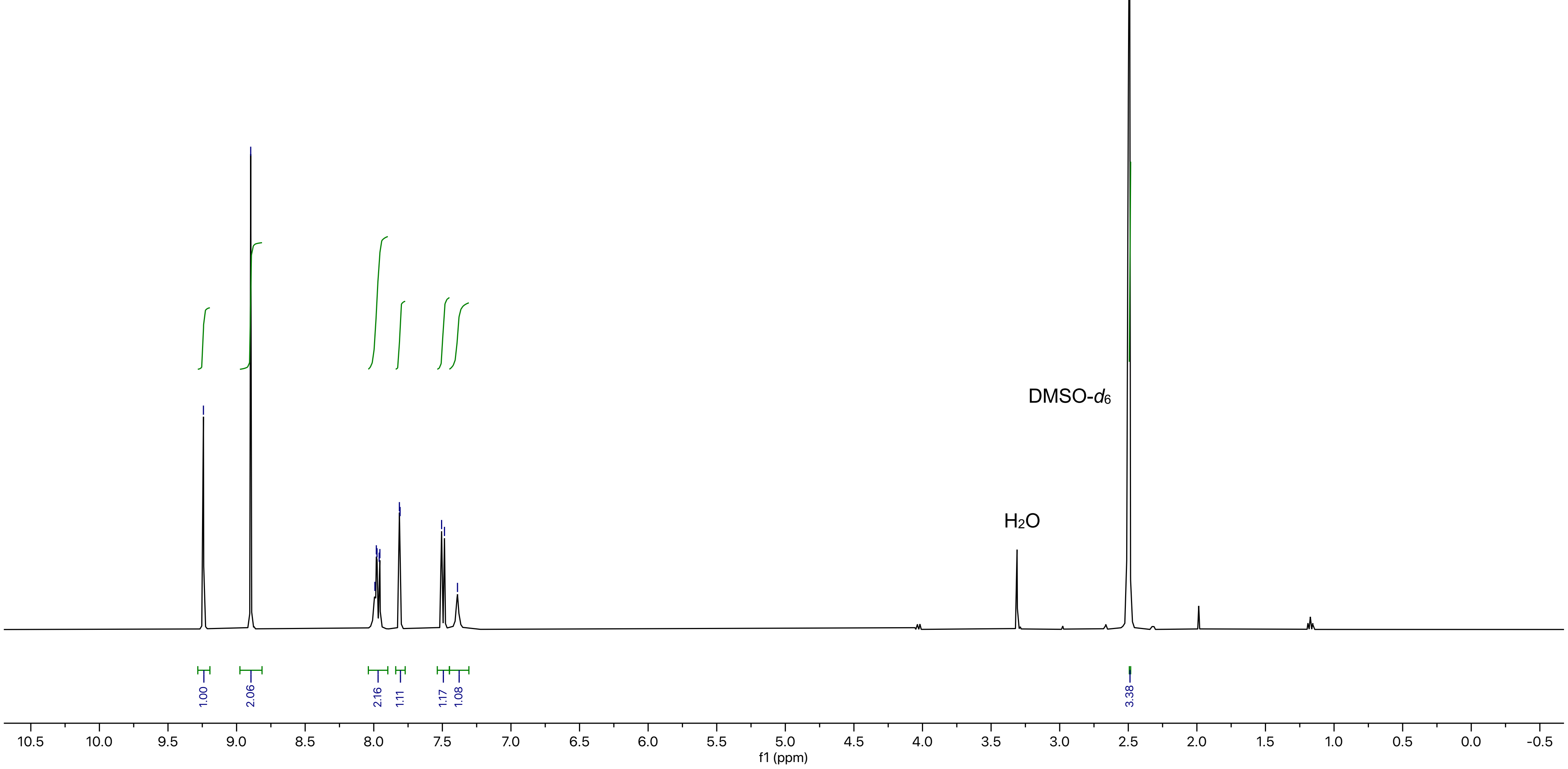

$^{13}C\{^{1}H\}$ NMR: 101 MHz, DMSO-d_6

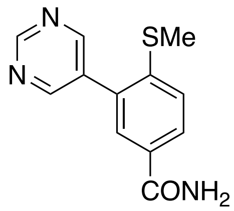

24

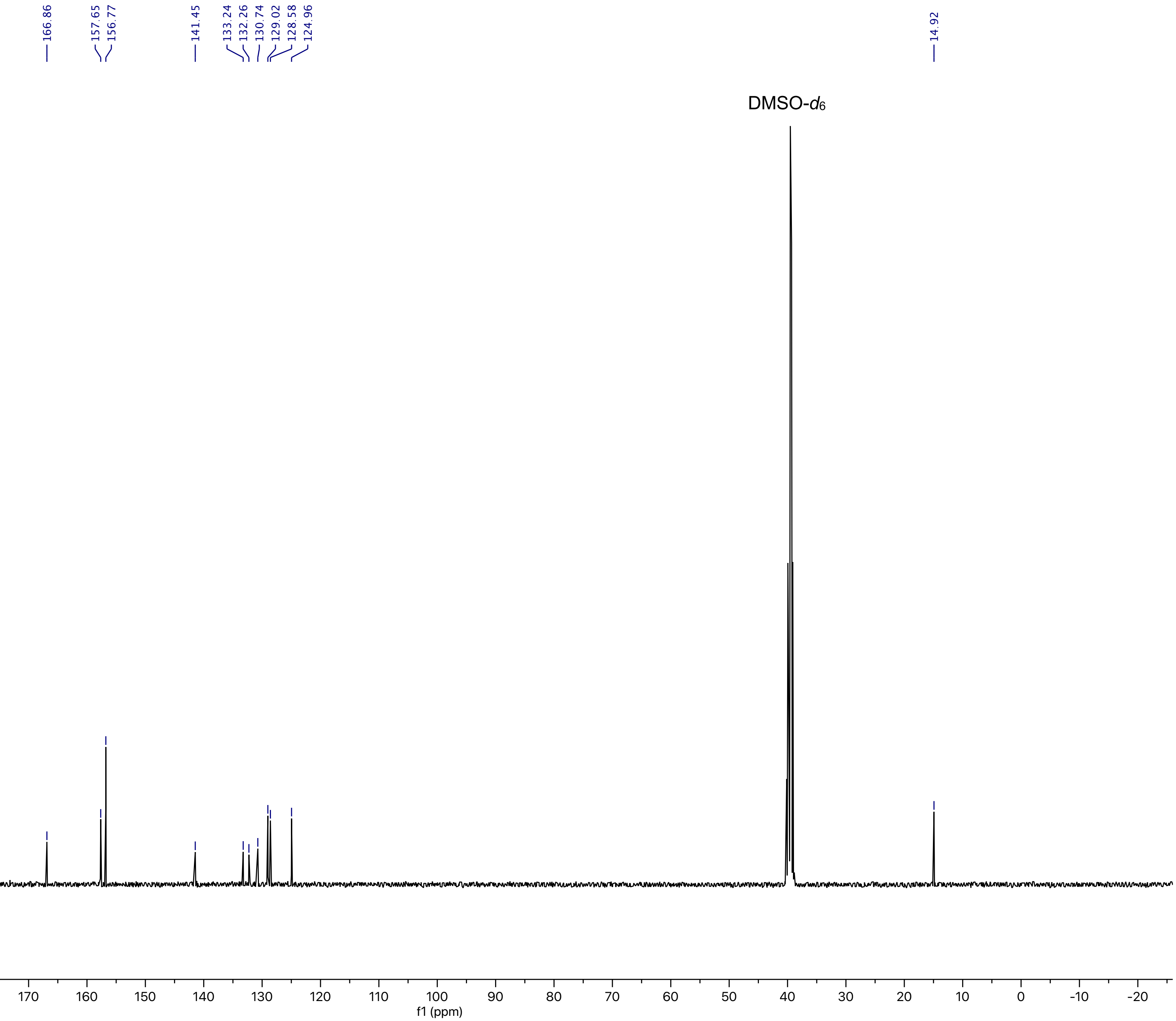

^{1}H NMR: 500 MHz, $CDCl_3$

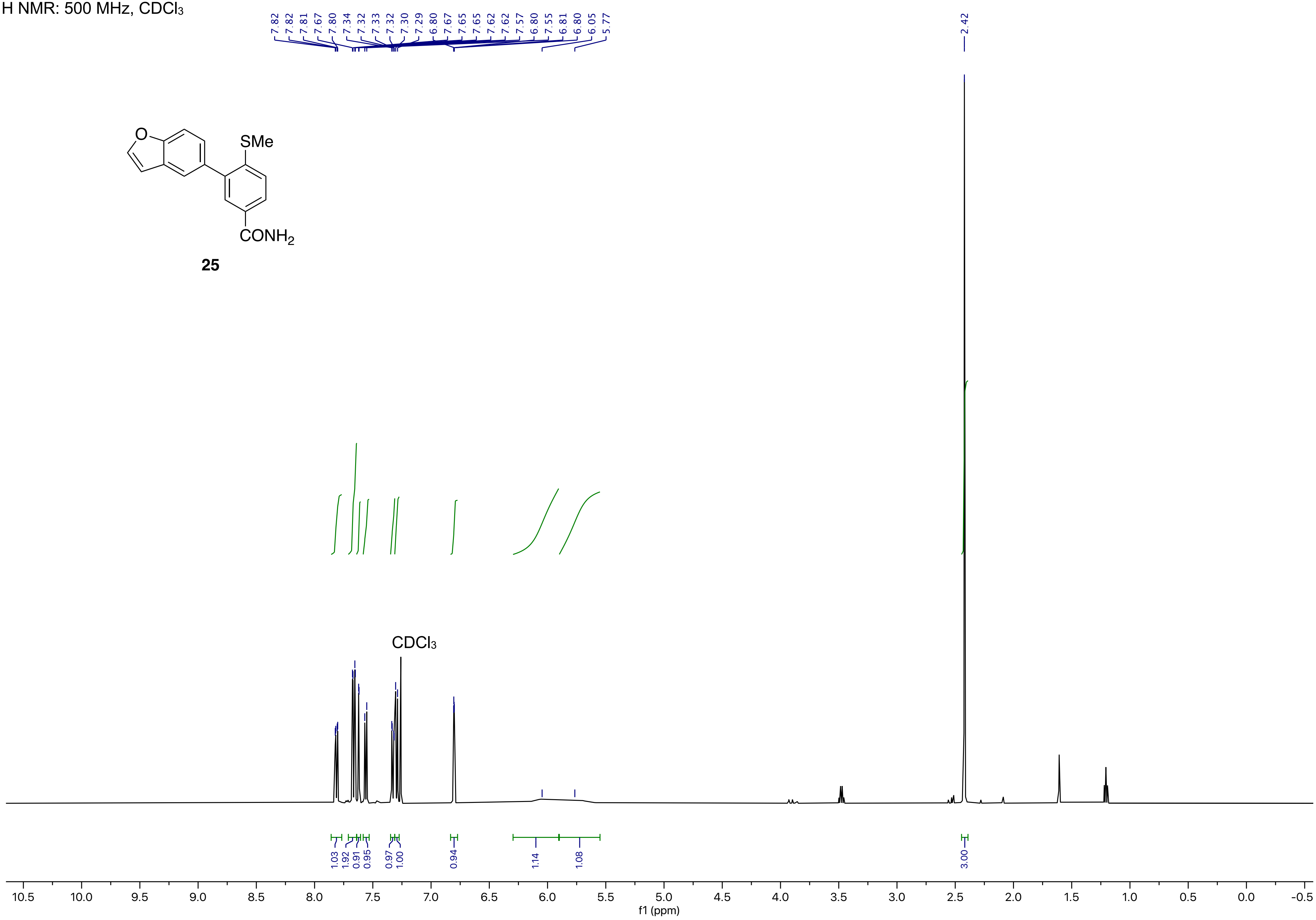

$^{13}C\{^1H\}$ NMR: 126 MHz, $CDCl_3$

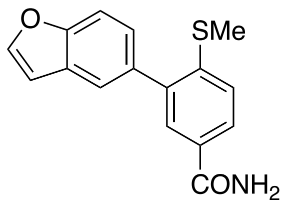

25

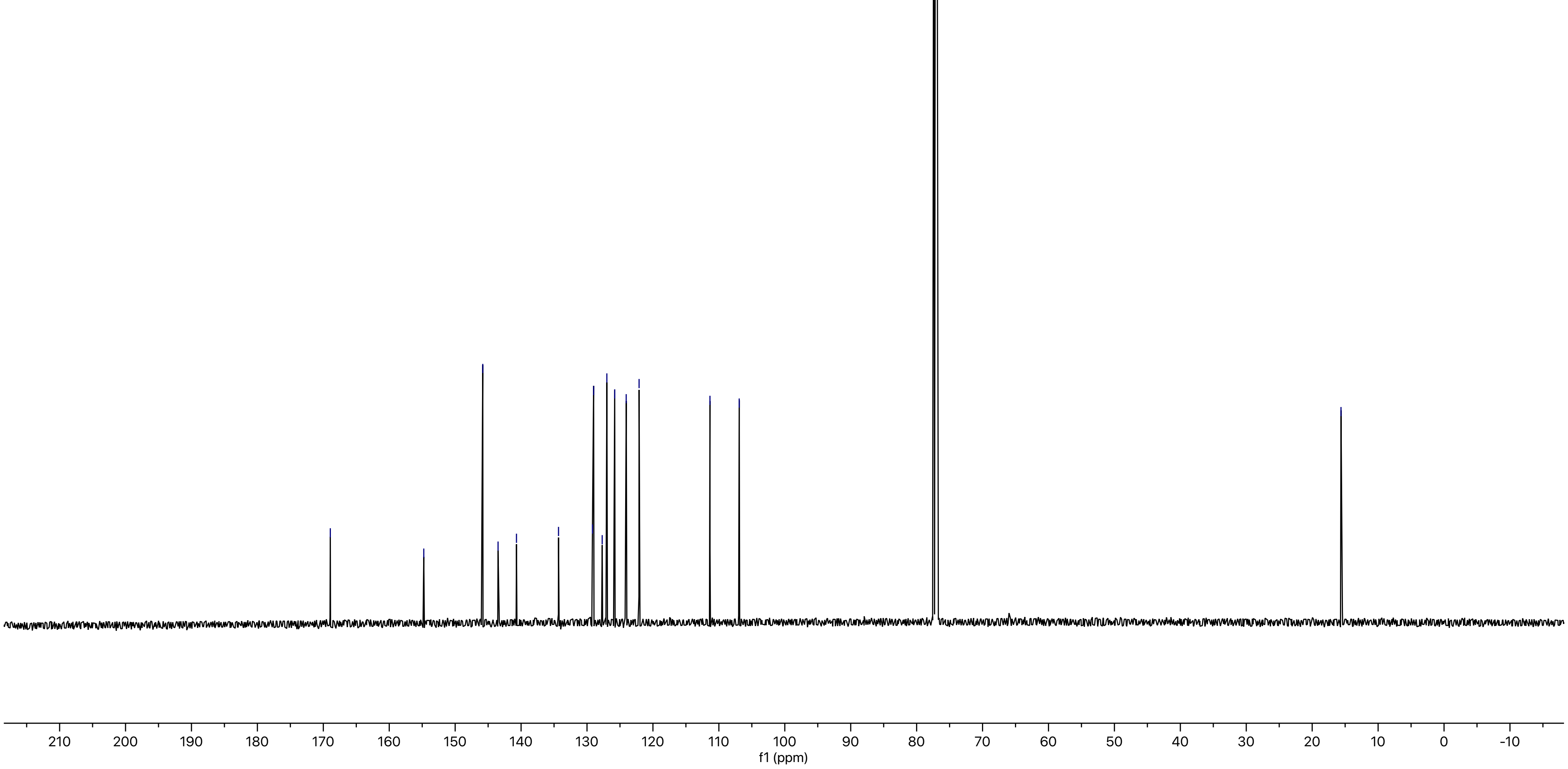

^{1}H NMR: 500 MHz, $CDCl_3$

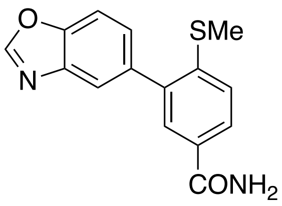

26

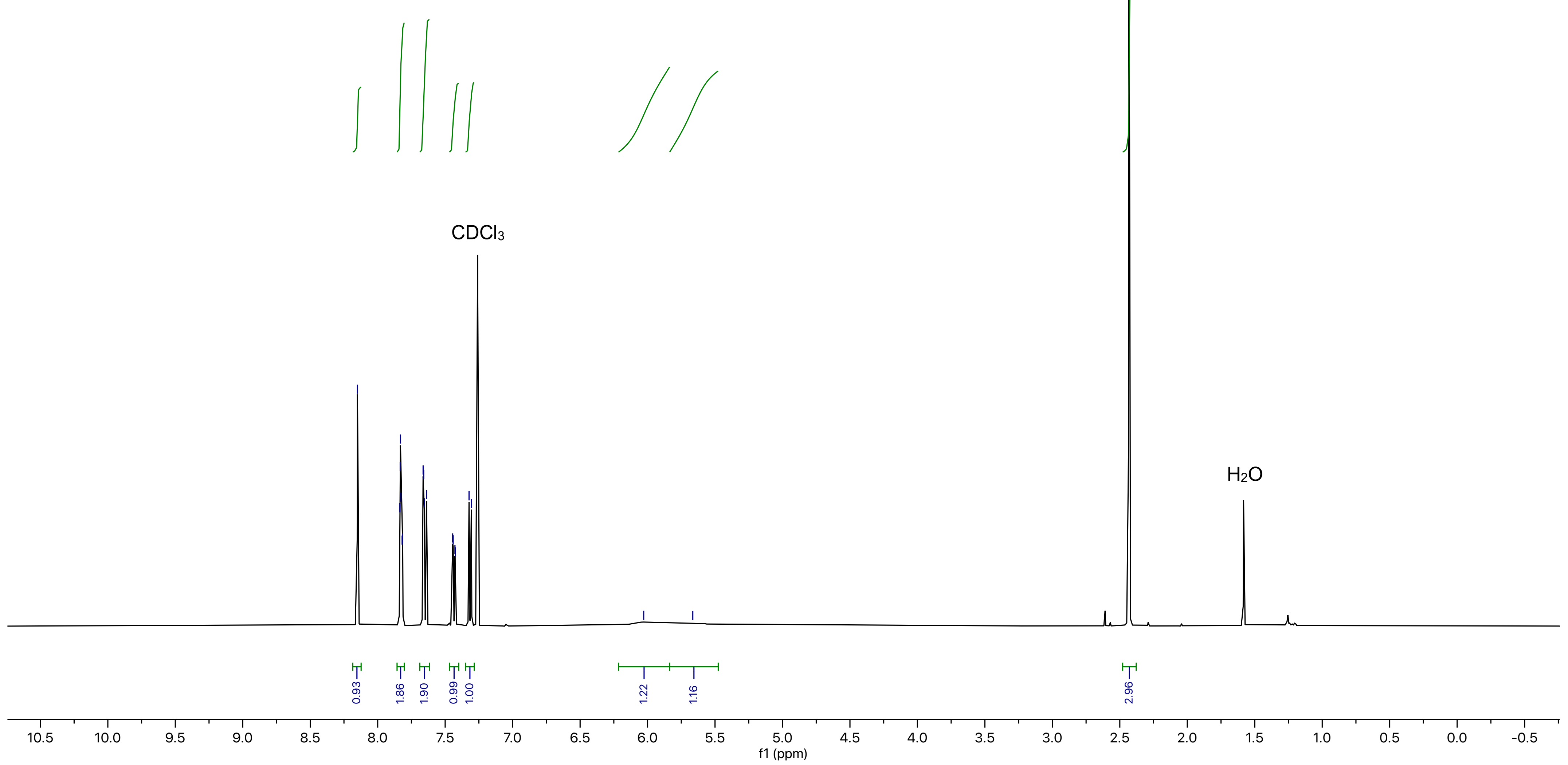

$^{13}C\{^{1}H\}$ NMR: 126 MHz, $CDCl_3$

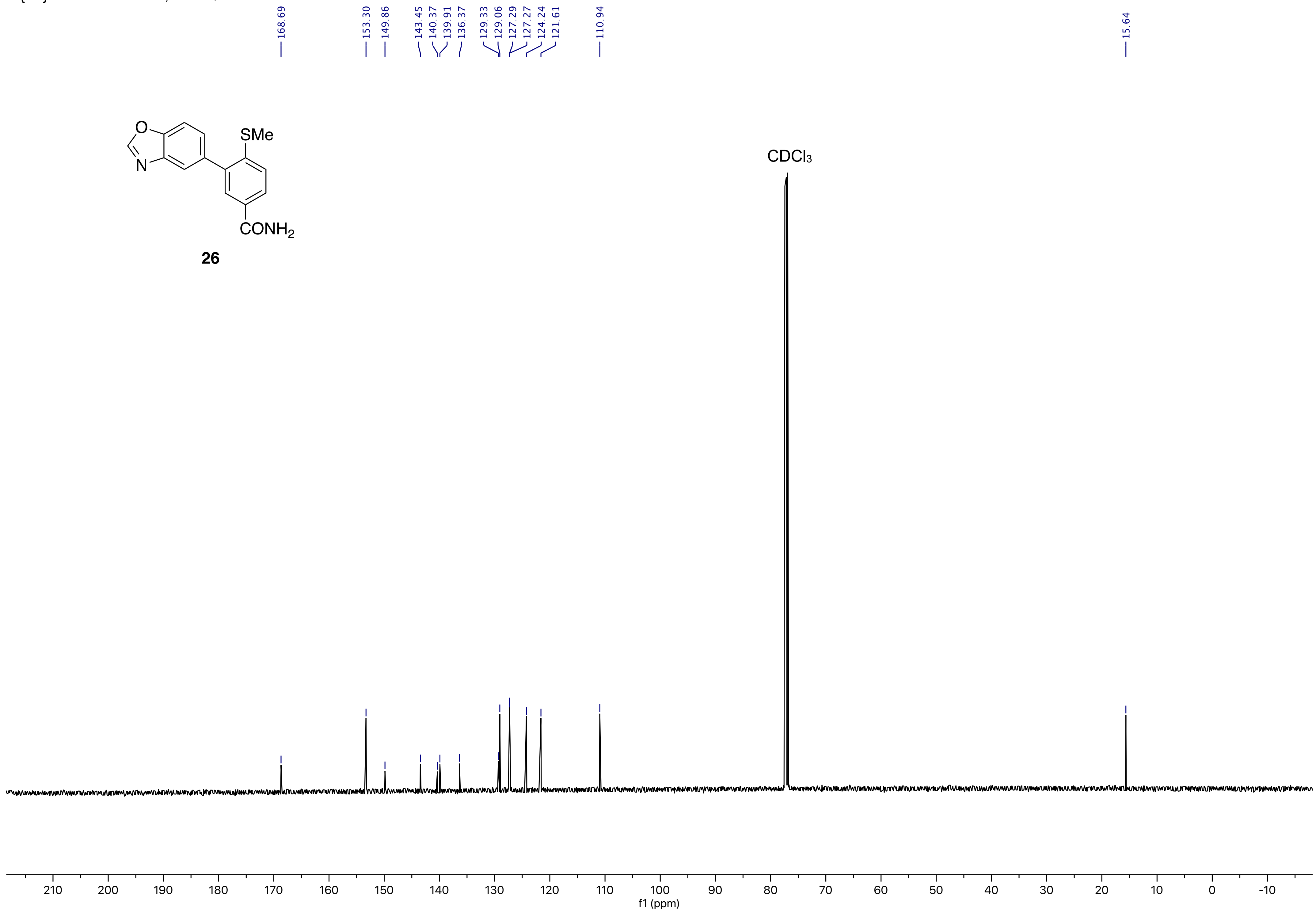

^{1}H NMR: 500 MHz, $CDCl_3$

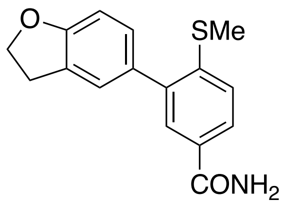

27

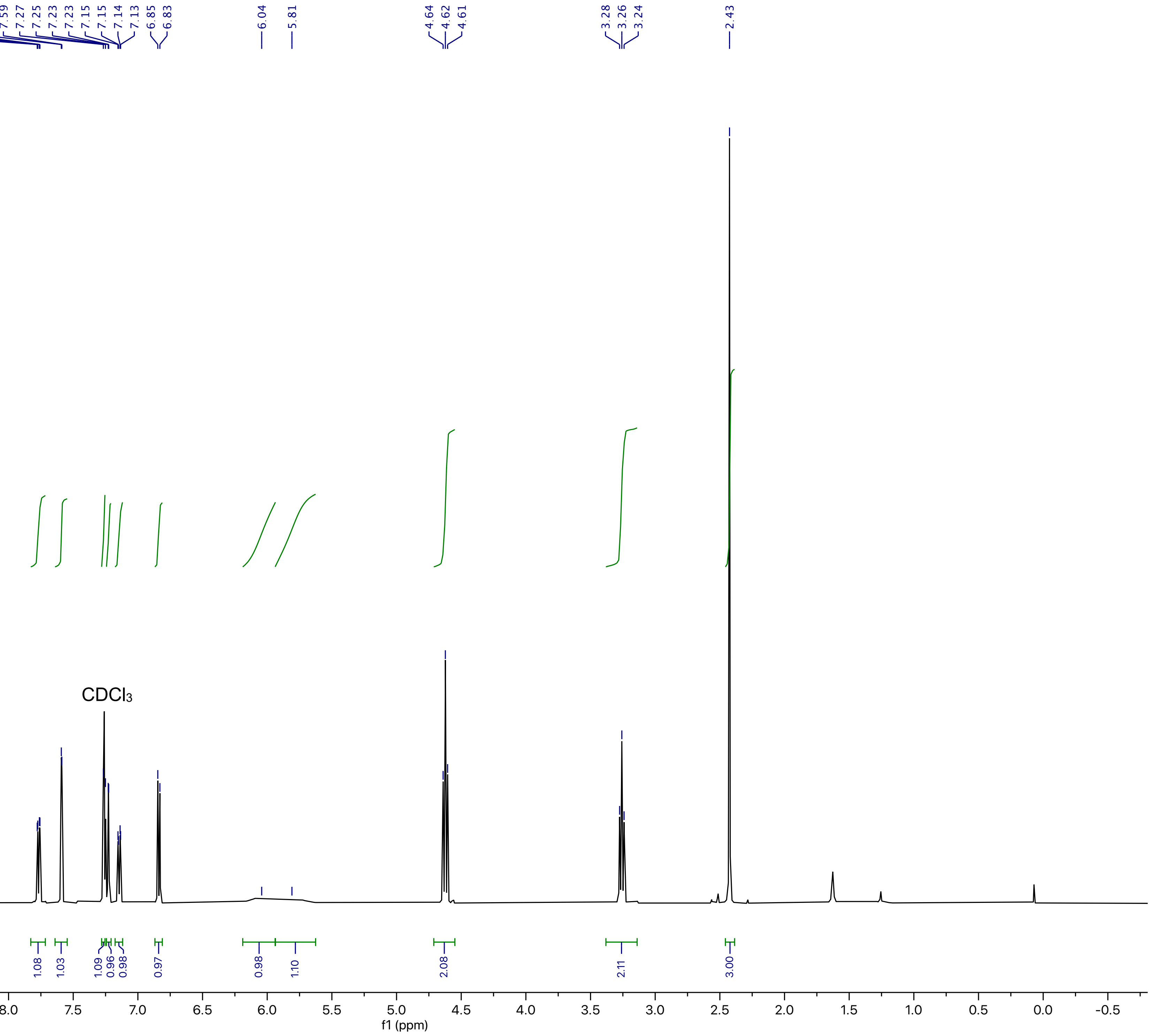

$^{13}C\{^{1}H\}$ NMR: 126 MHz, $CDCl_3$

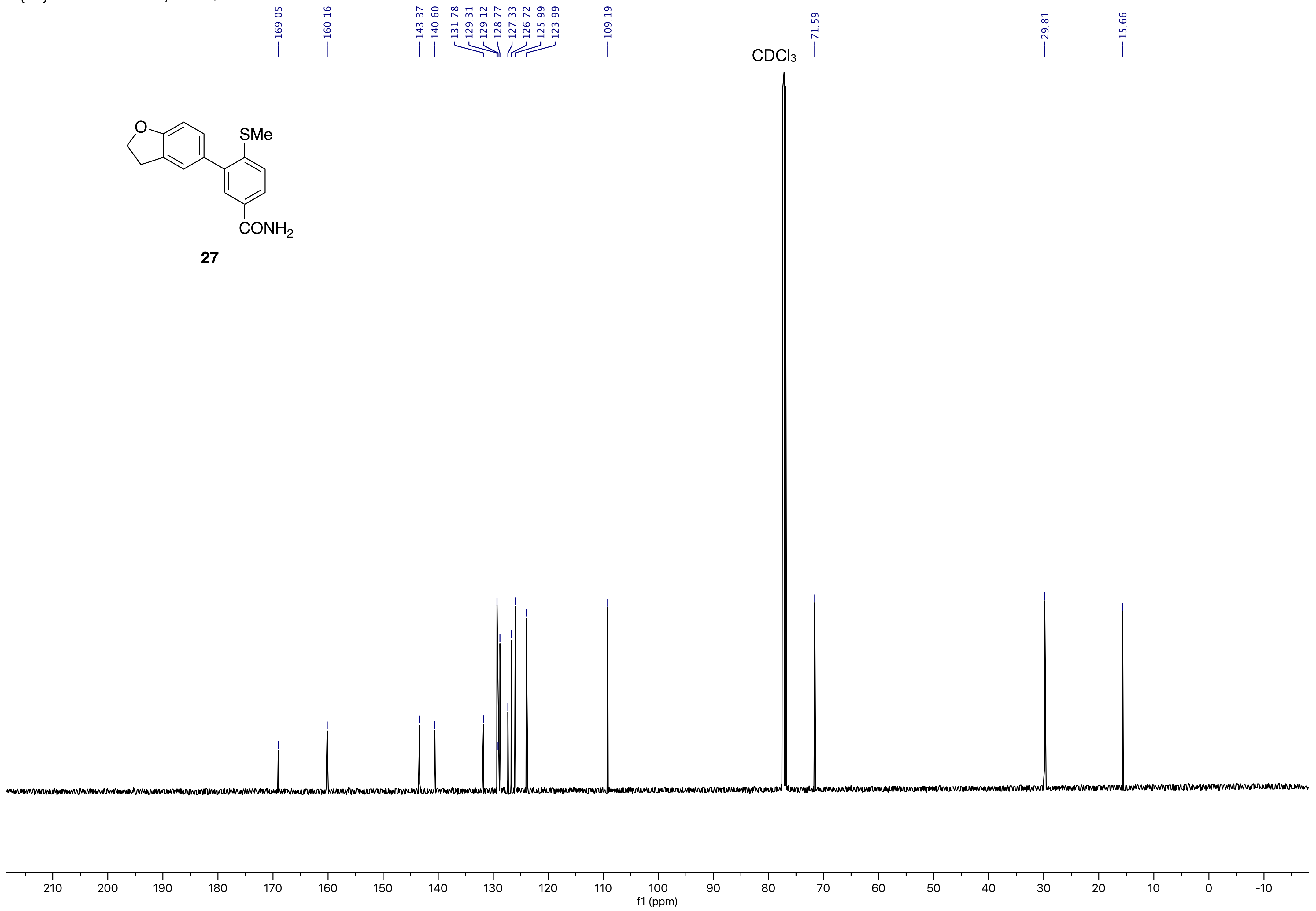

^{1}H NMR: 500 MHz, DMSO-d_6

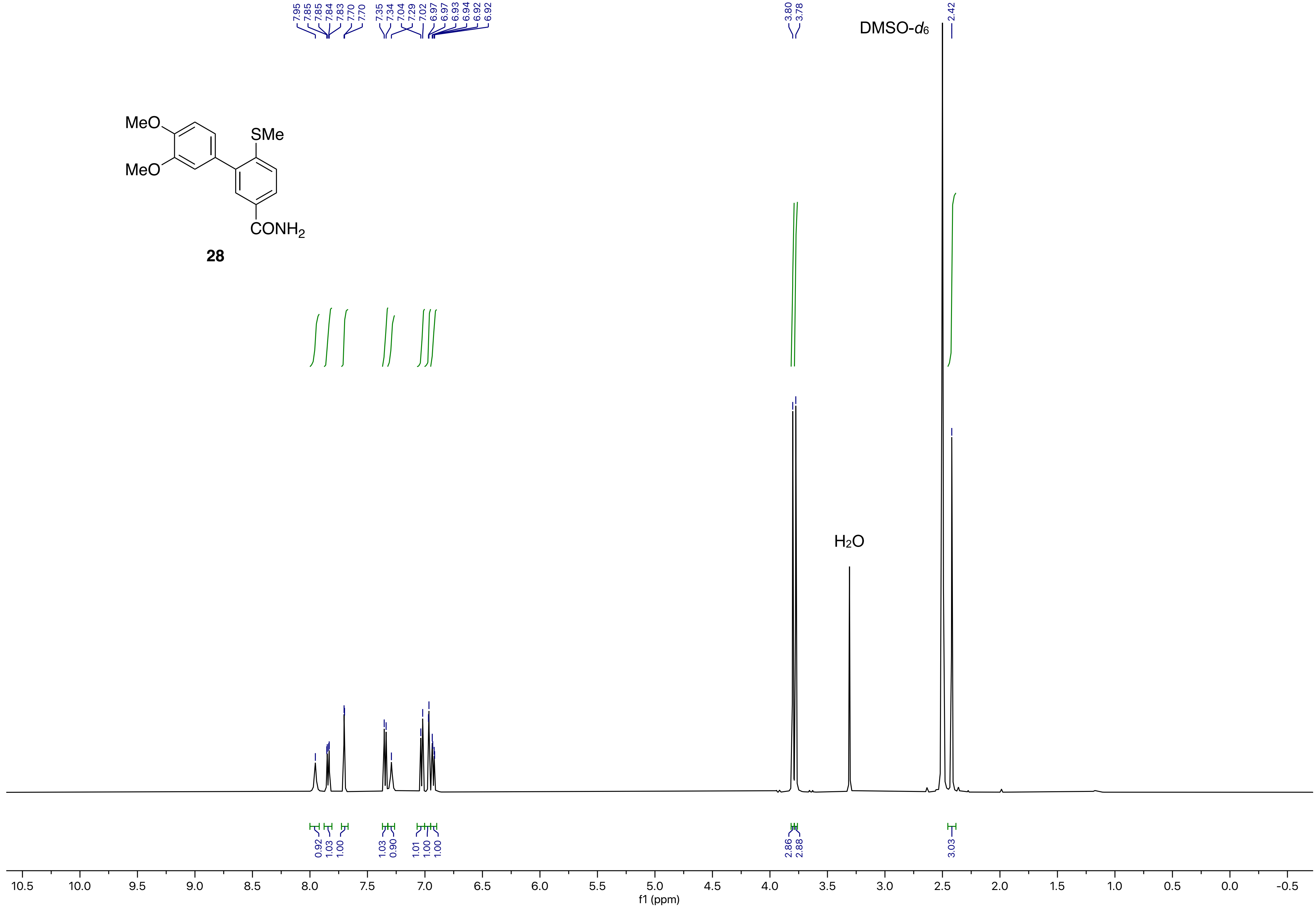

$^{13}C\{^1H\}$ NMR: 126 MHz, DMSO-d_6

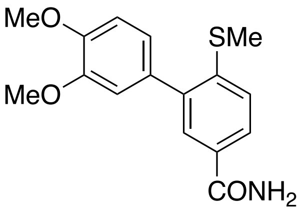

28

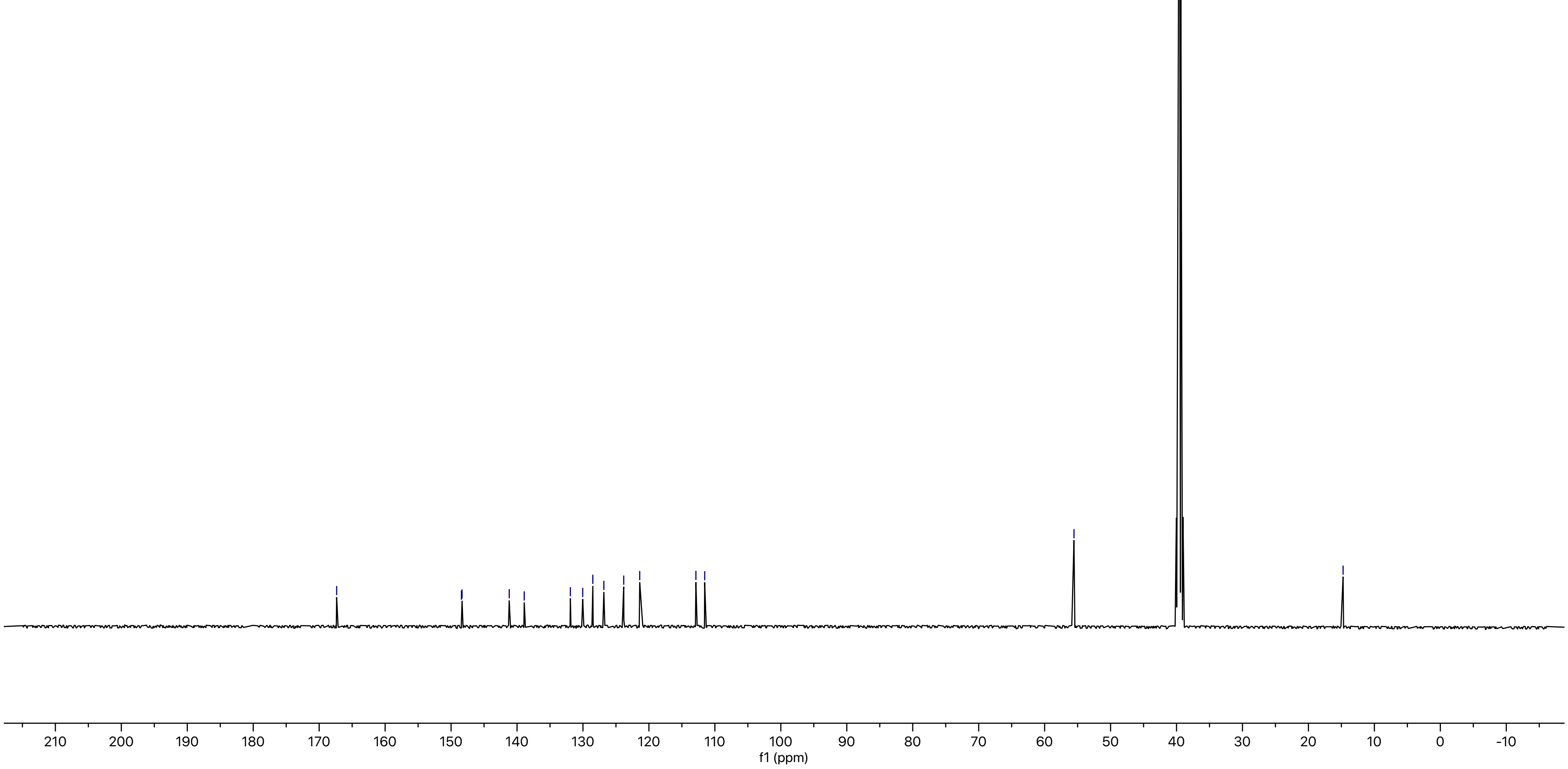

^{1}H NMR: 400 MHz, DMSO-d_6

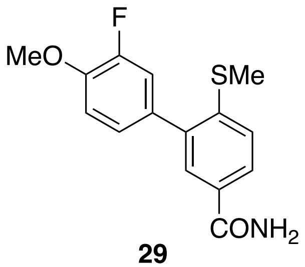

29

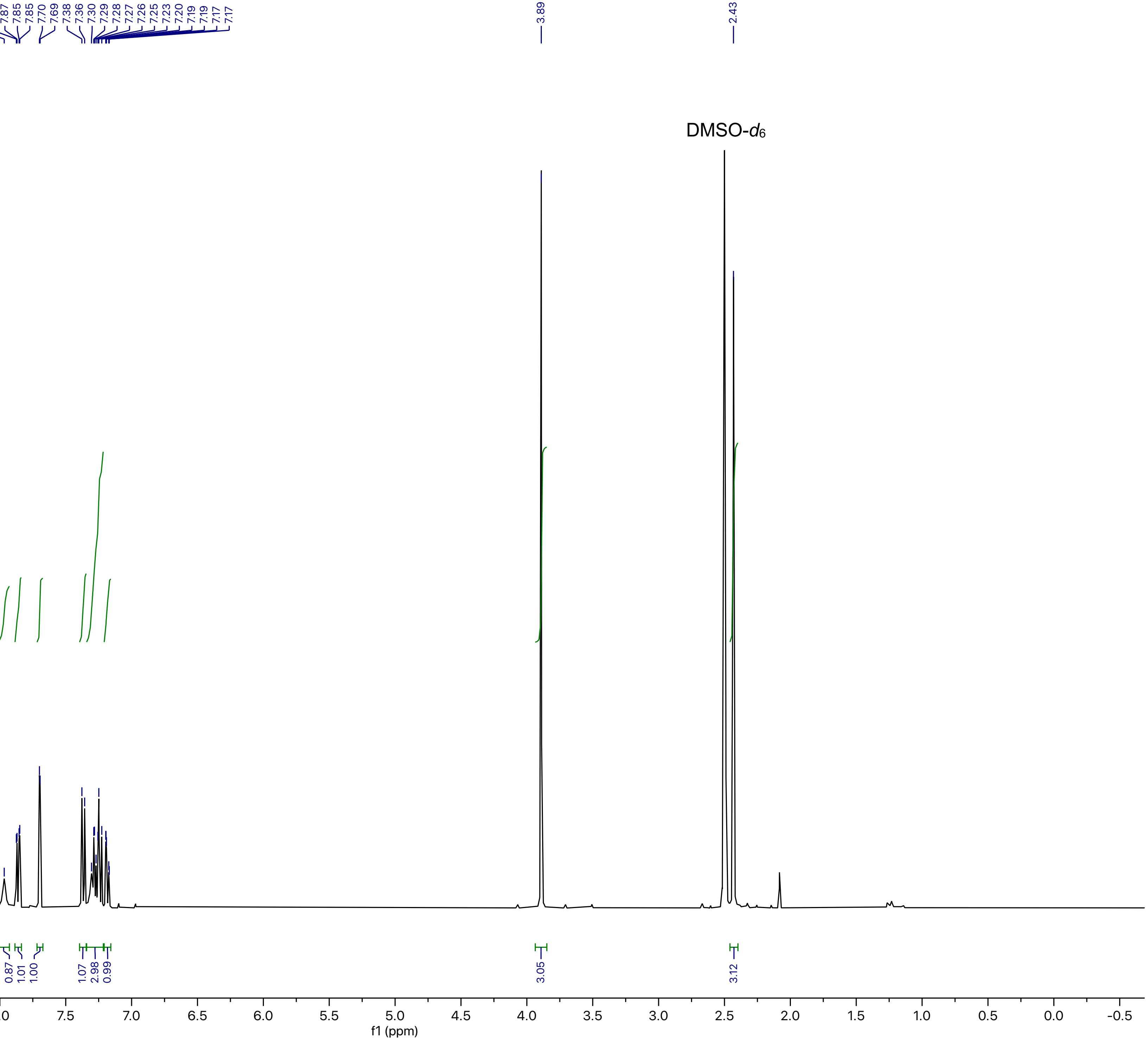

$^{13}C\{^{1}H\}$ NMR: 101 MHz, DMSO-d_6

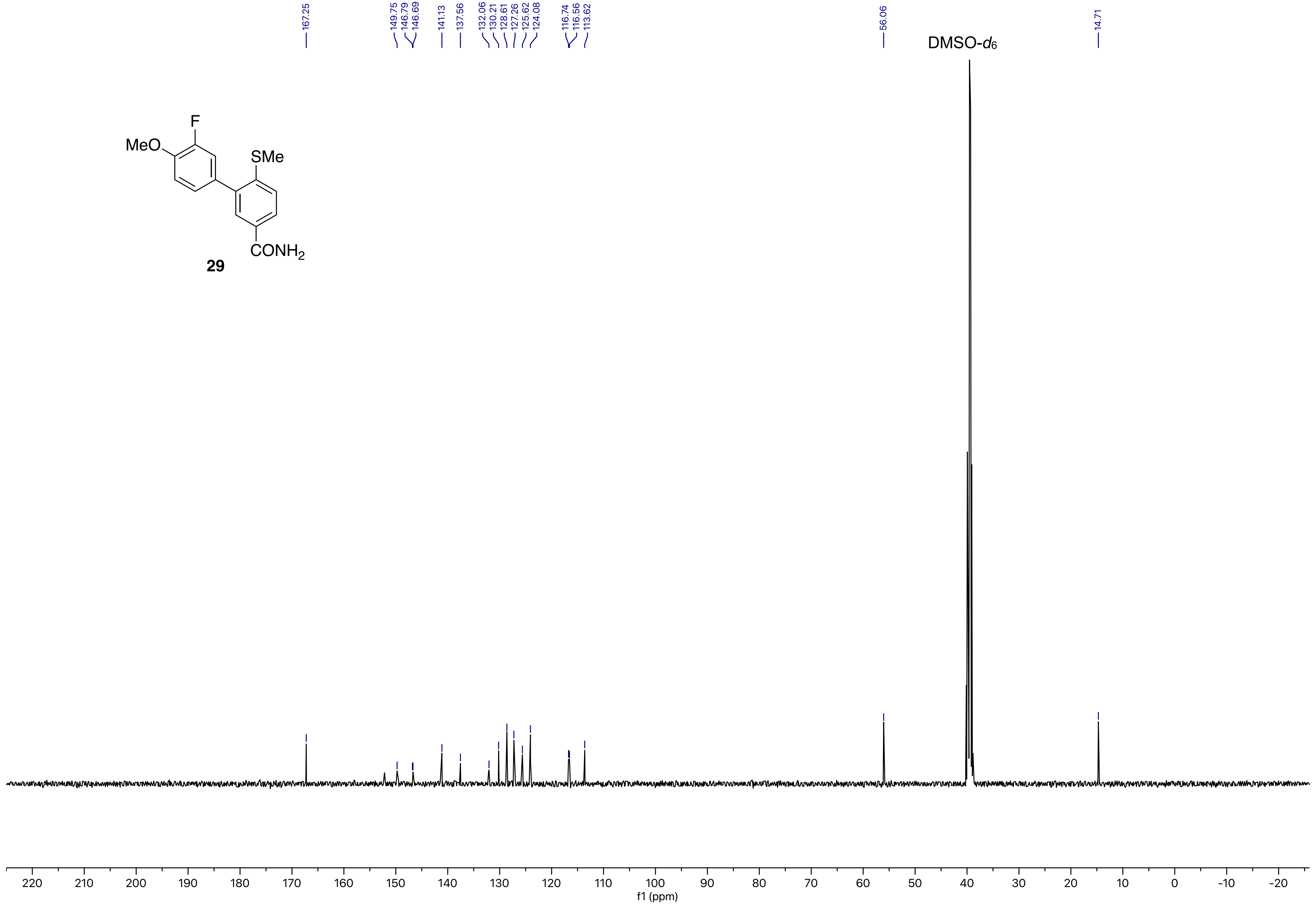

^1H NMR: 400 MHz, DMSO-d_6

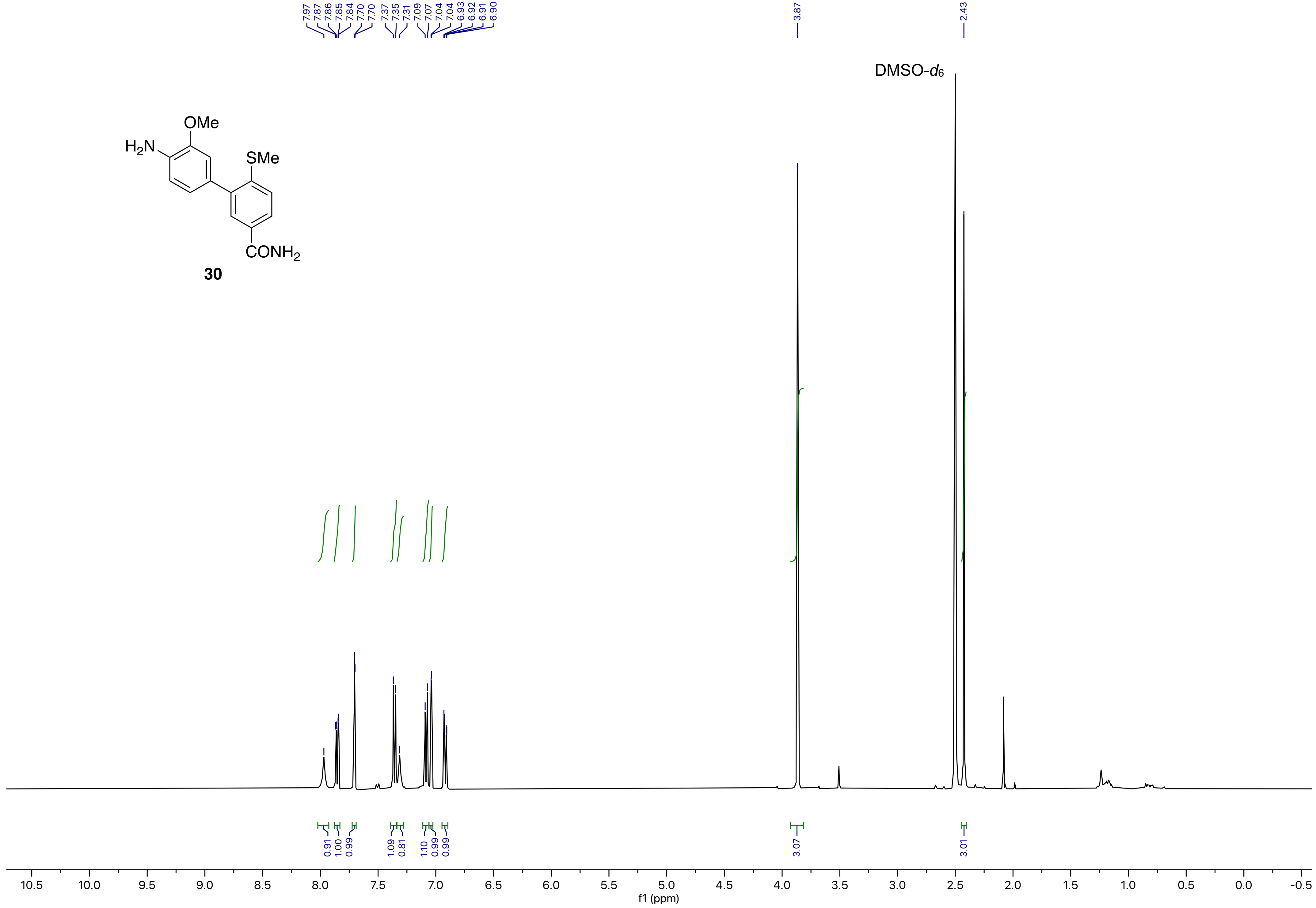

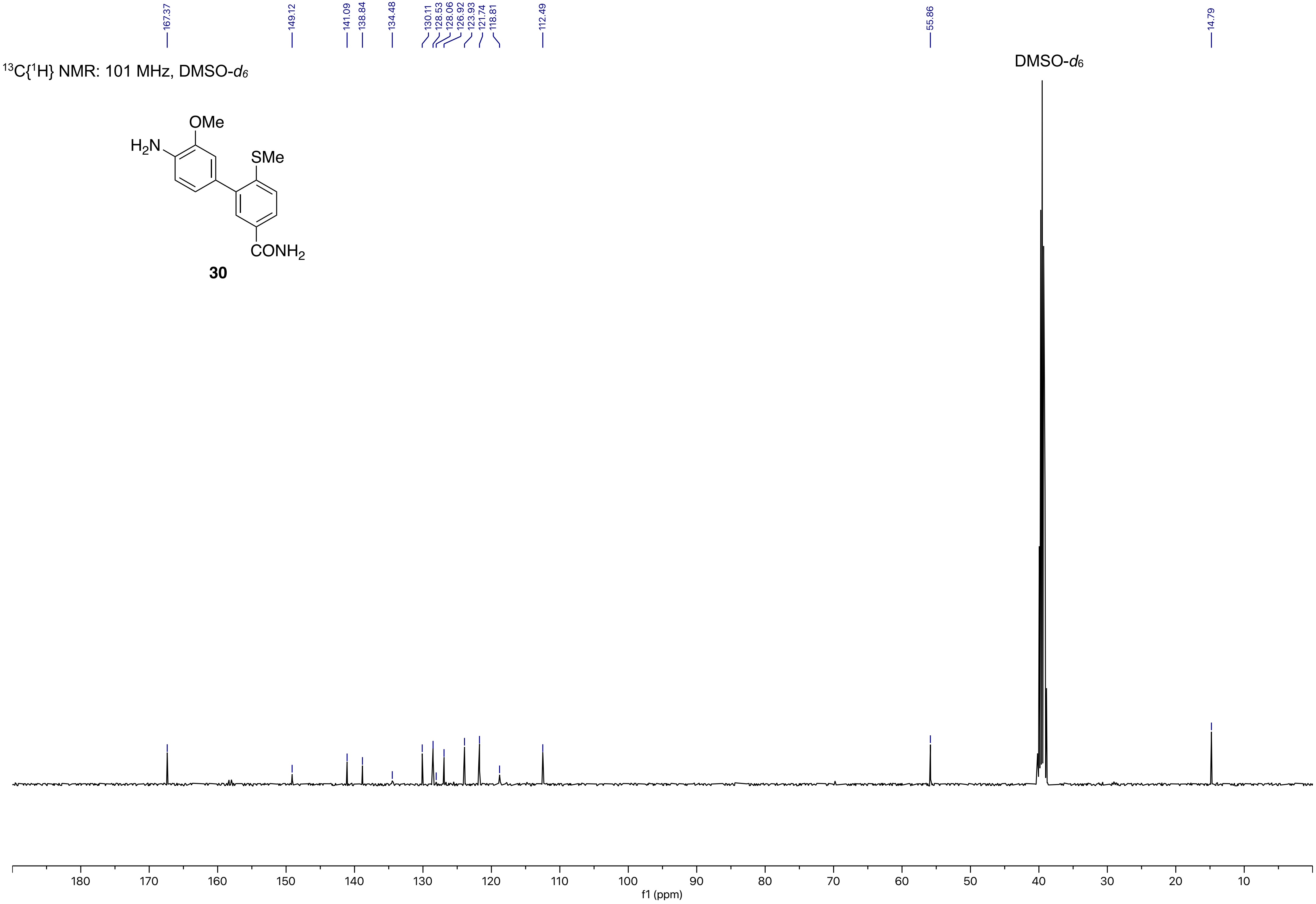

$^{13}C\{^{1}H\}$ NMR: 101 MHz, DMSO-d_6
OMe
H_2N
SMe
$CONH_2$
30
167.37
149.12
141.09
138.84
134.48
130.11
128.53
128.06
126.92
123.93
121.74
118.81
112.49
55.86
14.79
DMSO-d_6
180
170
160
150
140
130
120
110
100
90
80
70
60
50
40
30
20
10
f1 (ppm)

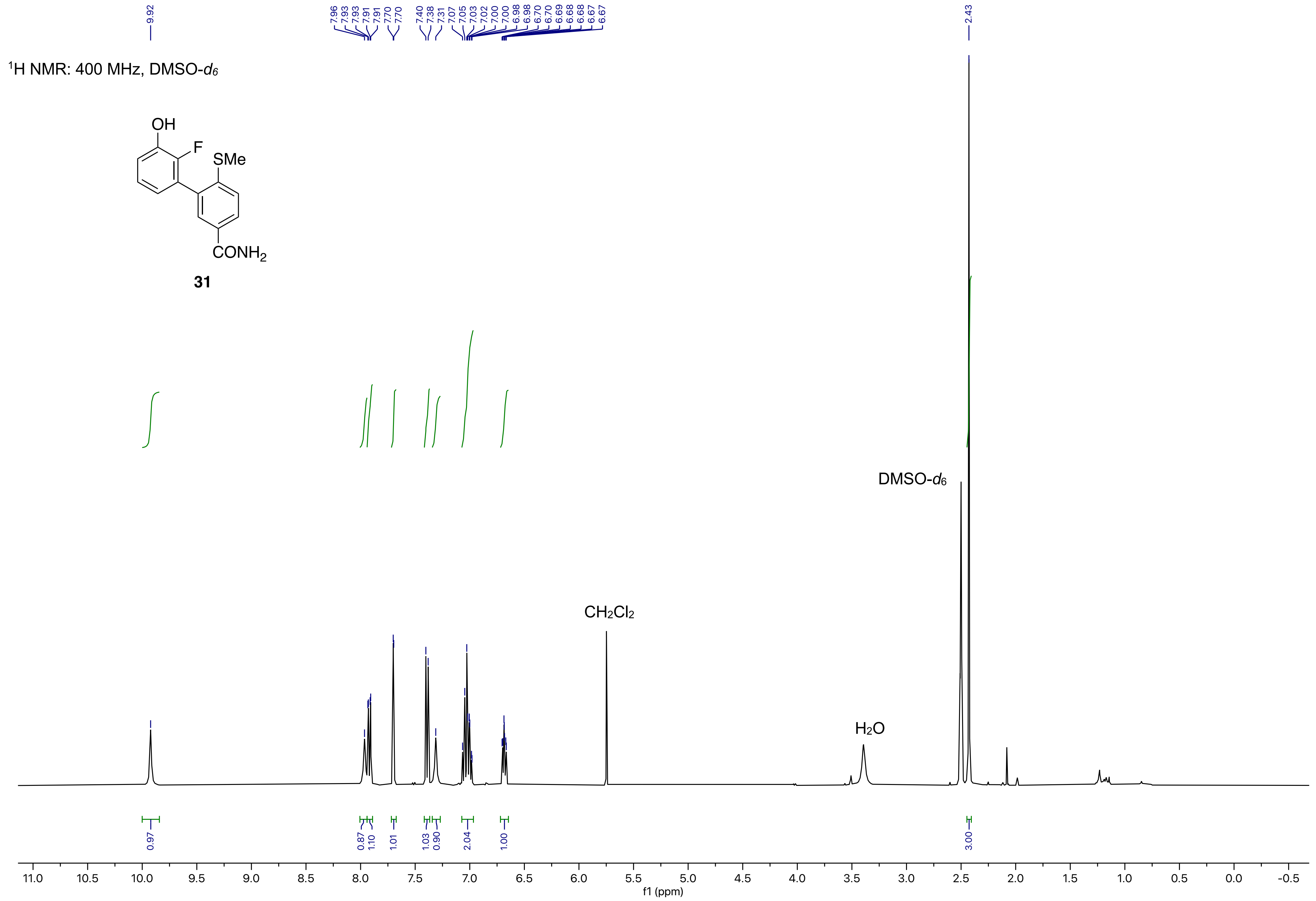

^{1}H NMR: 400 MHz, DMSO-d_6
31
CH$_2$Cl$_2$
H$_2$O
DMSO-d_6
f1 (ppm)

$^{13}C\{^{1}H\}$ NMR: 101 MHz, DMSO-d_6

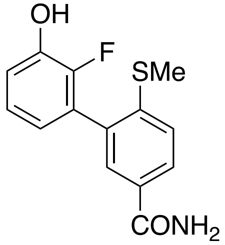

31

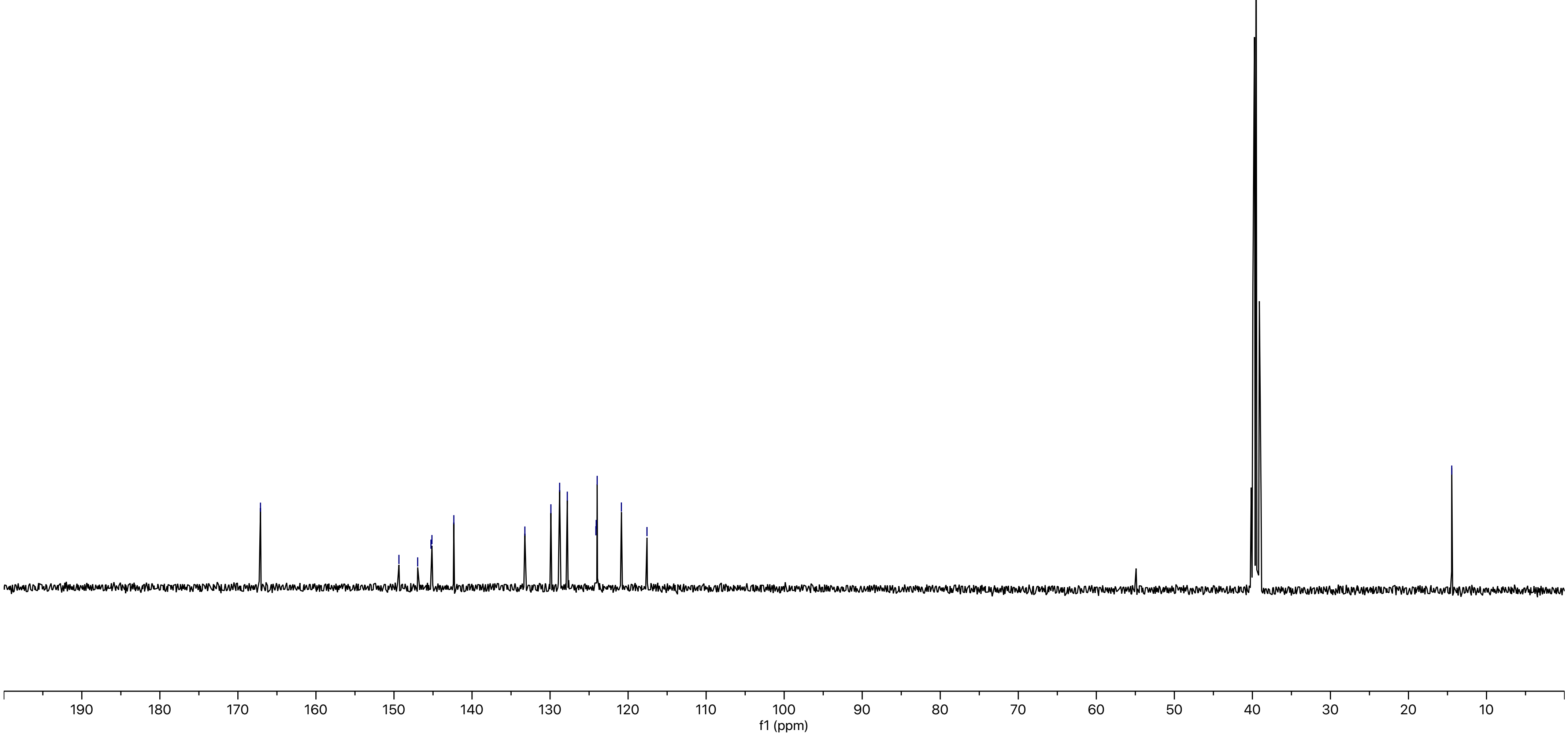

^{19}F NMR: 471 MHz, DMSO-d_6

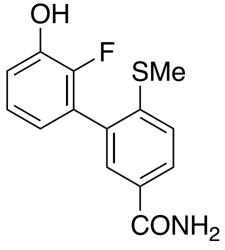

31

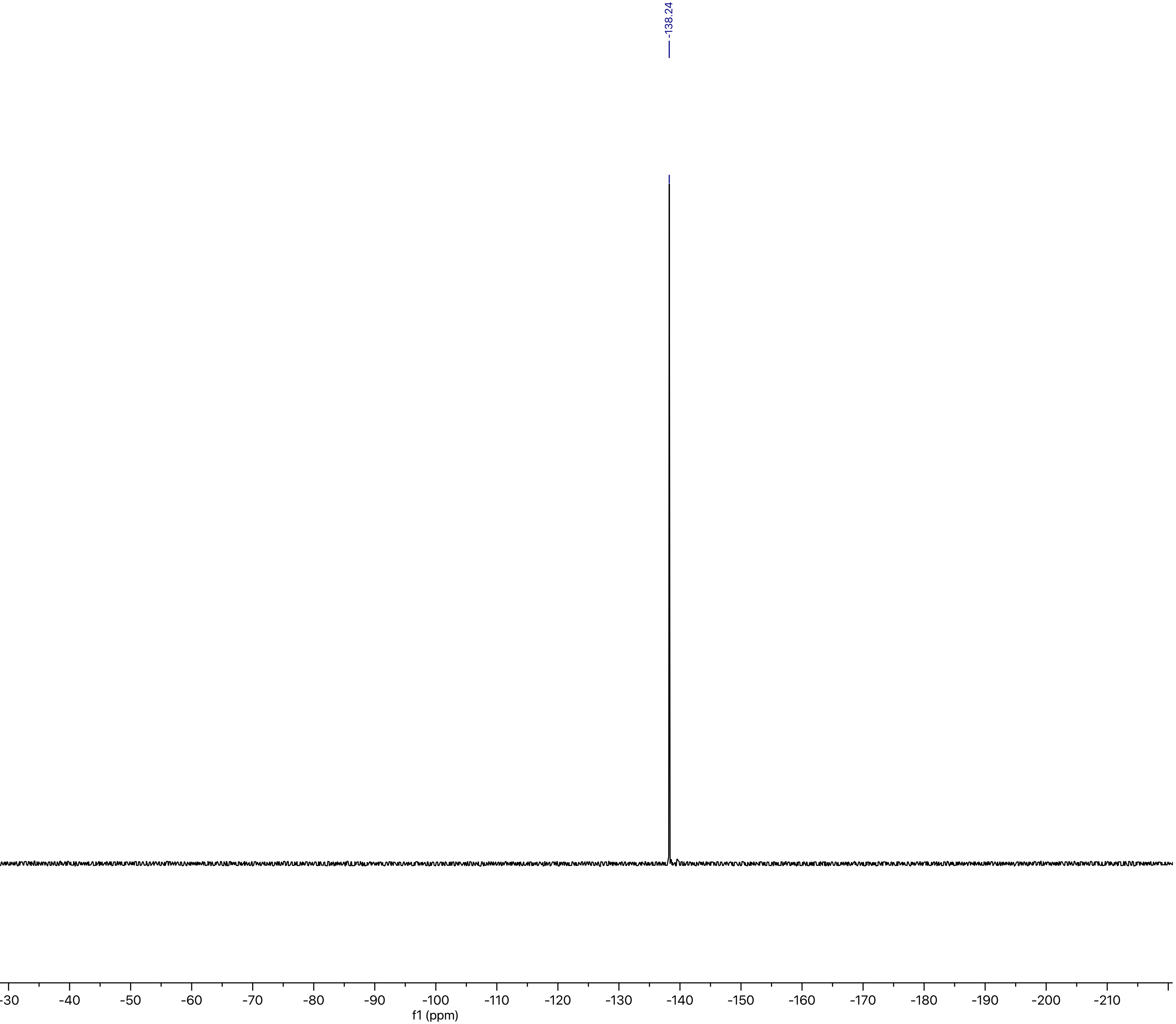

^{1}H NMR: 400 MHz, acetone-d_6

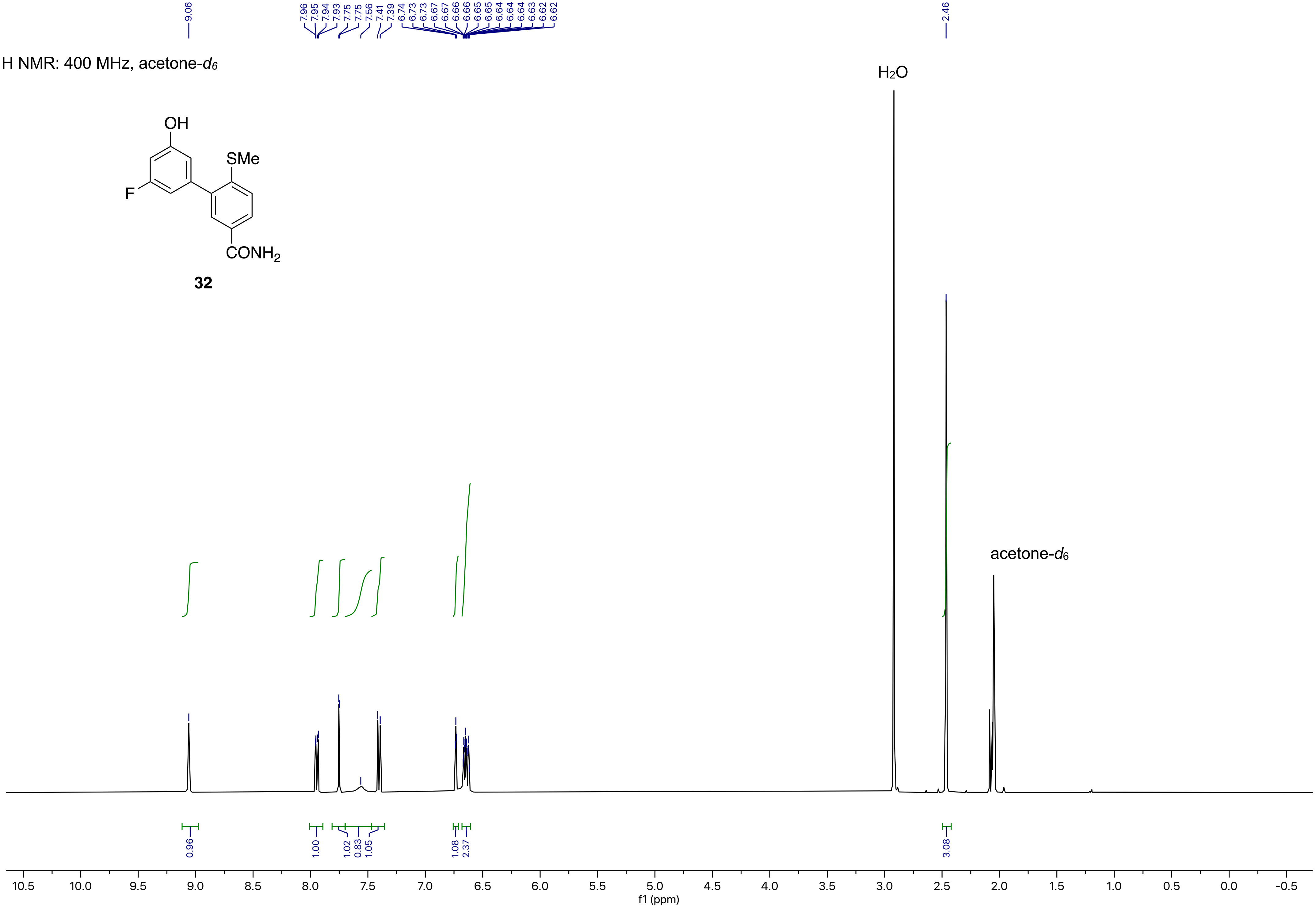

32

$^{13}C\{^1H\}$ NMR: 101 MHz, acetone-d_6

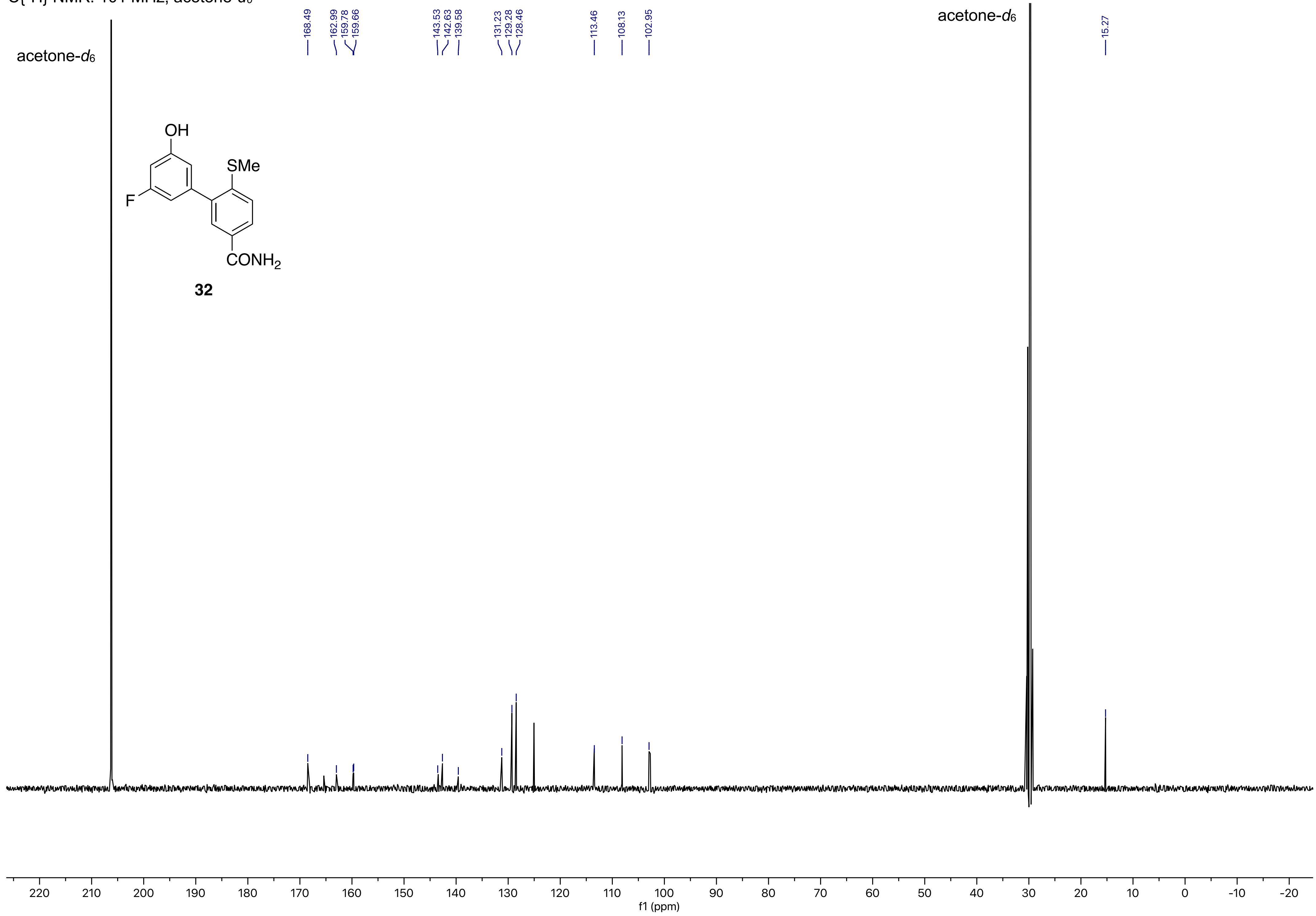

^{19}F NMR: 471 MHz, DMSO-d_6

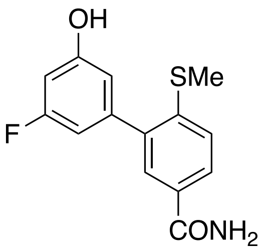

32

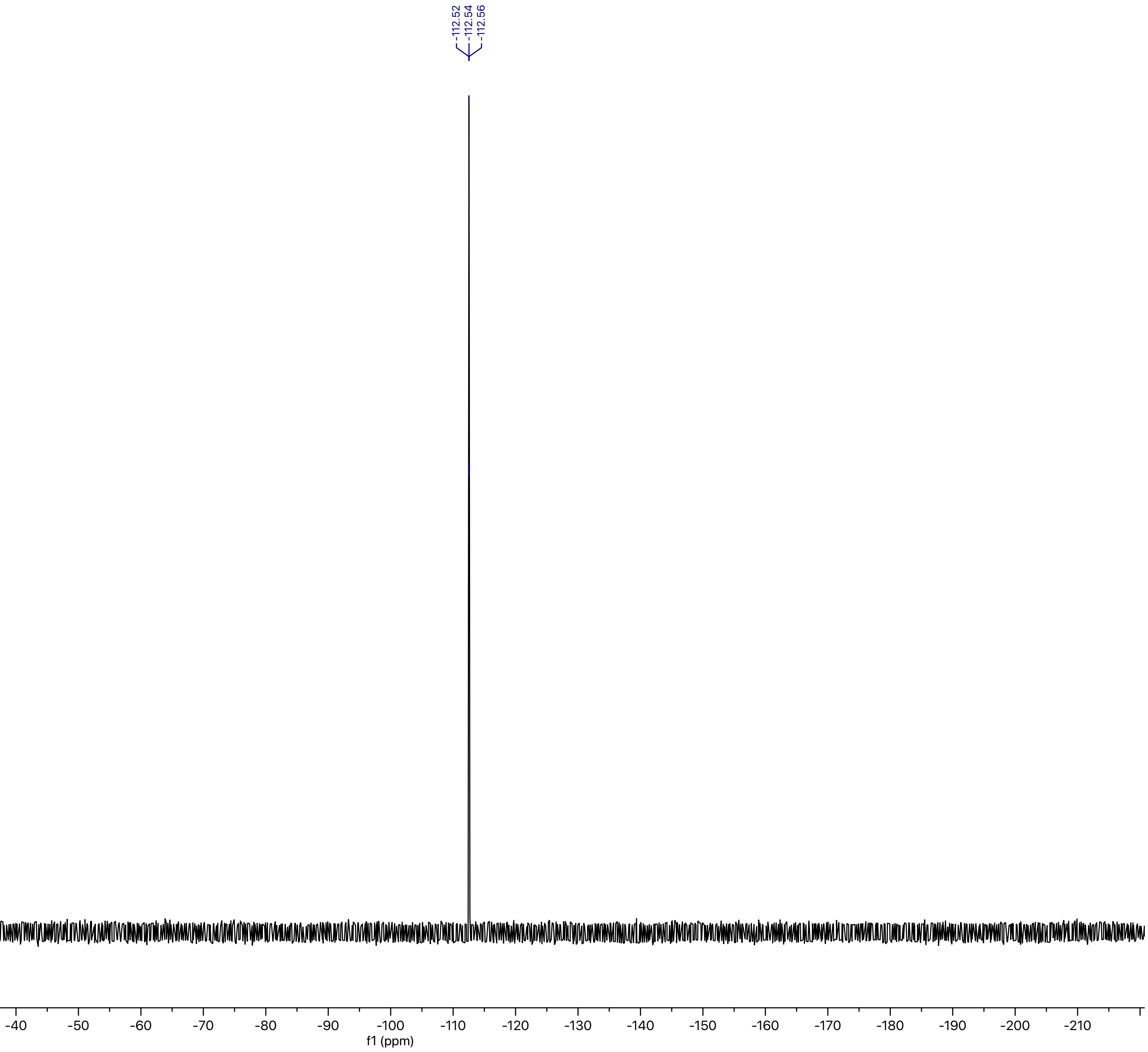

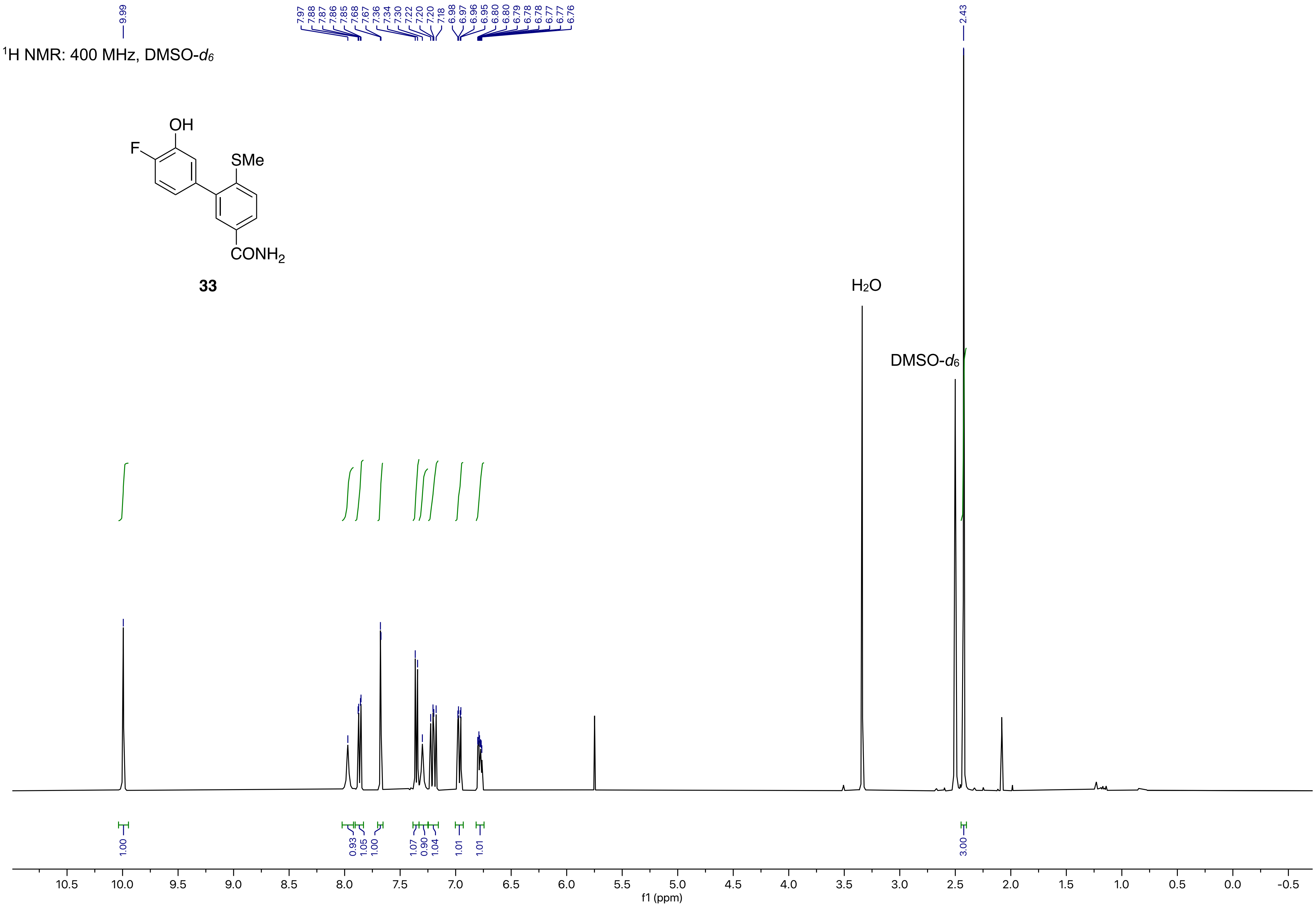

^{1}H NMR: 400 MHz, DMSO-d_6
OH
F
SMe
CONH$_2$
33
9.99
7.97 7.88 7.87 7.86 7.85 7.68 7.67 7.36 7.34 7.30 7.22 7.20 7.20 7.18 6.98 6.97 6.96 6.95 6.80 6.80 6.79 6.78 6.78 6.77 6.77 6.76
2.43
H_2O
DMSO-d_6
1.00
0.93 1.05 1.00
1.07 0.90 1.04
1.01
1.01
3.00
10.5 10.0 9.5 9.0 8.5 8.0 7.5 7.0 6.5 6.0 5.5 5.0 4.5 4.0 3.5 3.0 2.5 2.0 1.5 1.0 0.5 0.0 -0.5
f1 (ppm)

$^{13}C\{^1H\}$ NMR: 101 MHz, DMSO-d_6

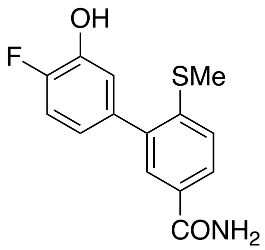

33

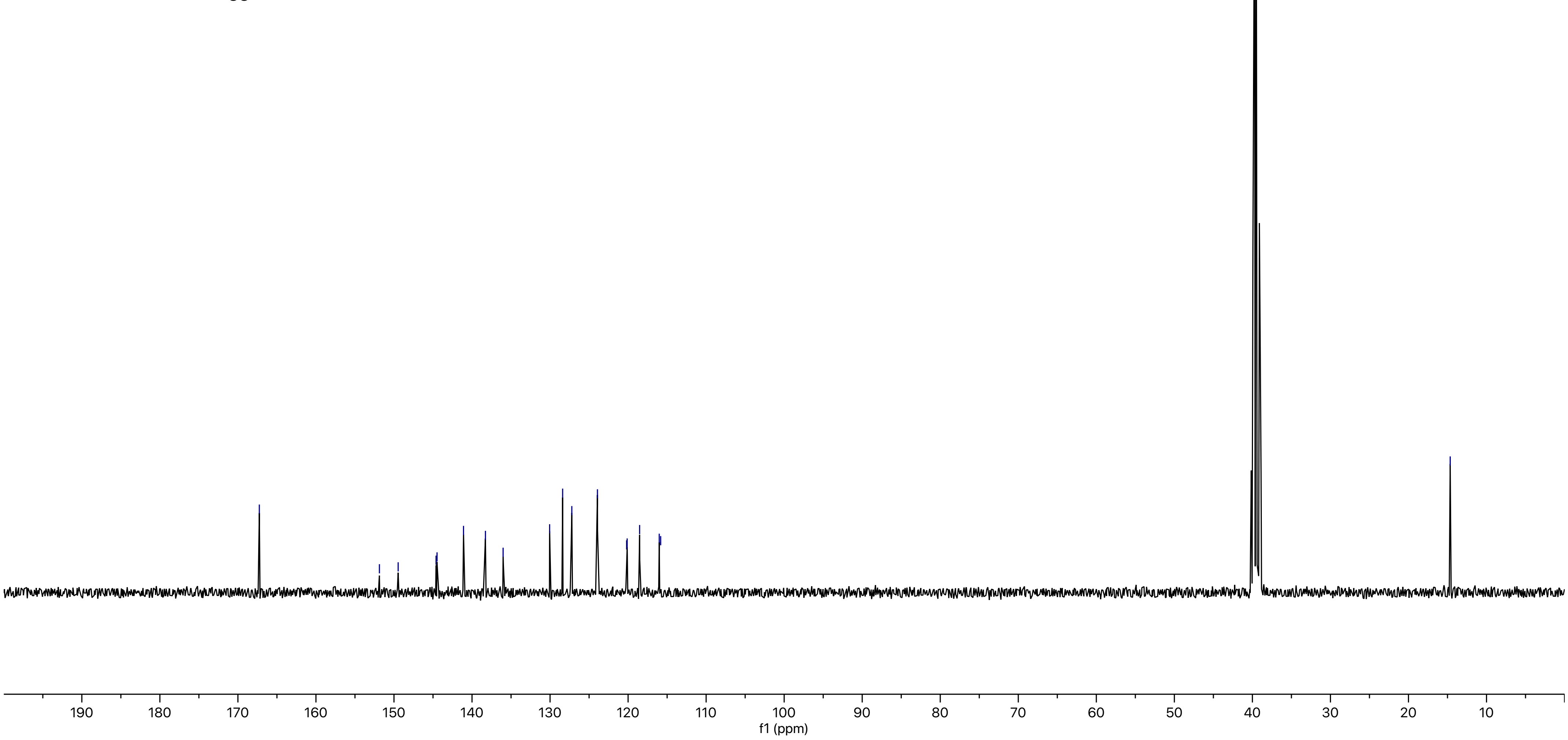

^{19}F NMR: 471 MHz, acetone-d_6

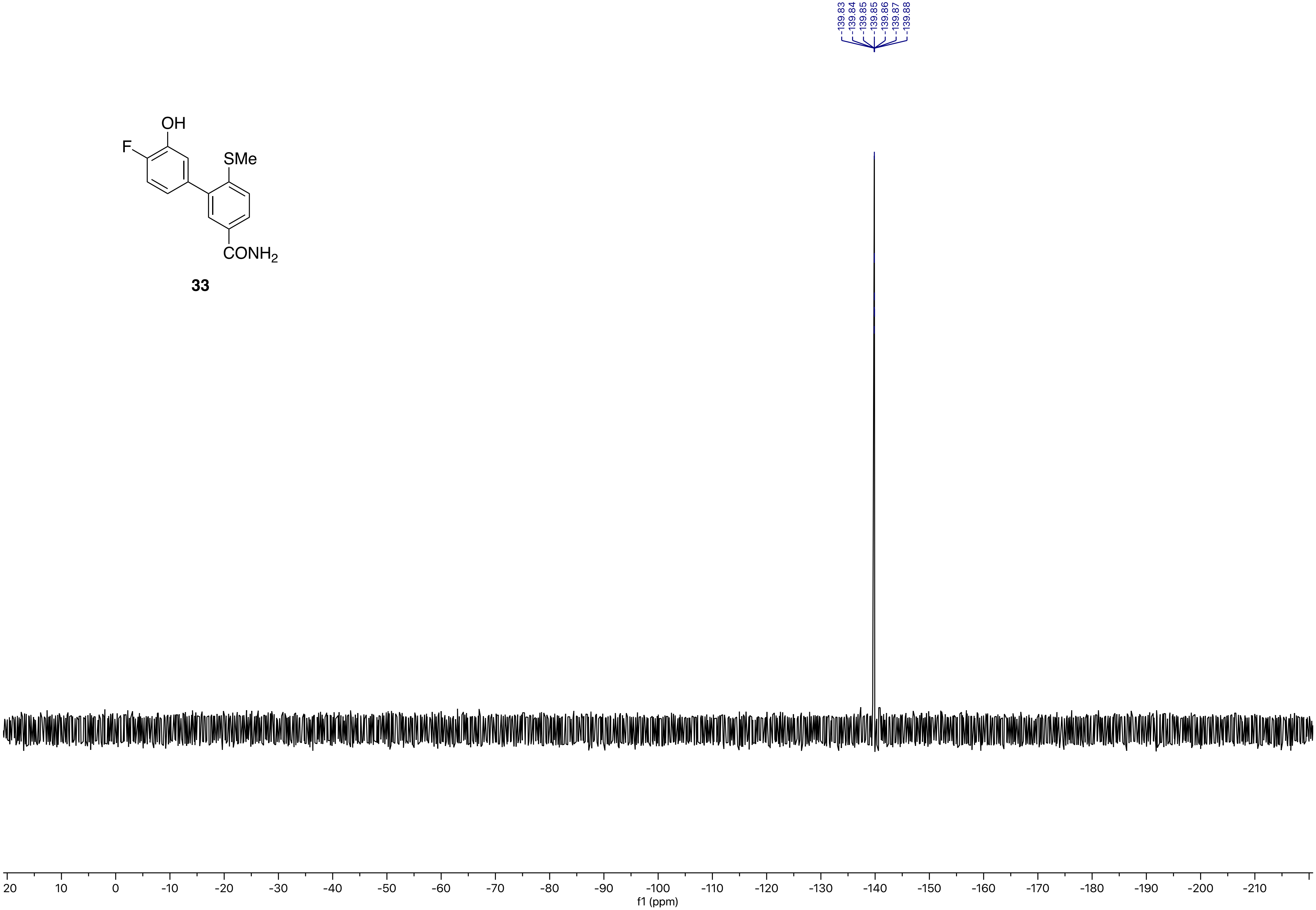

33

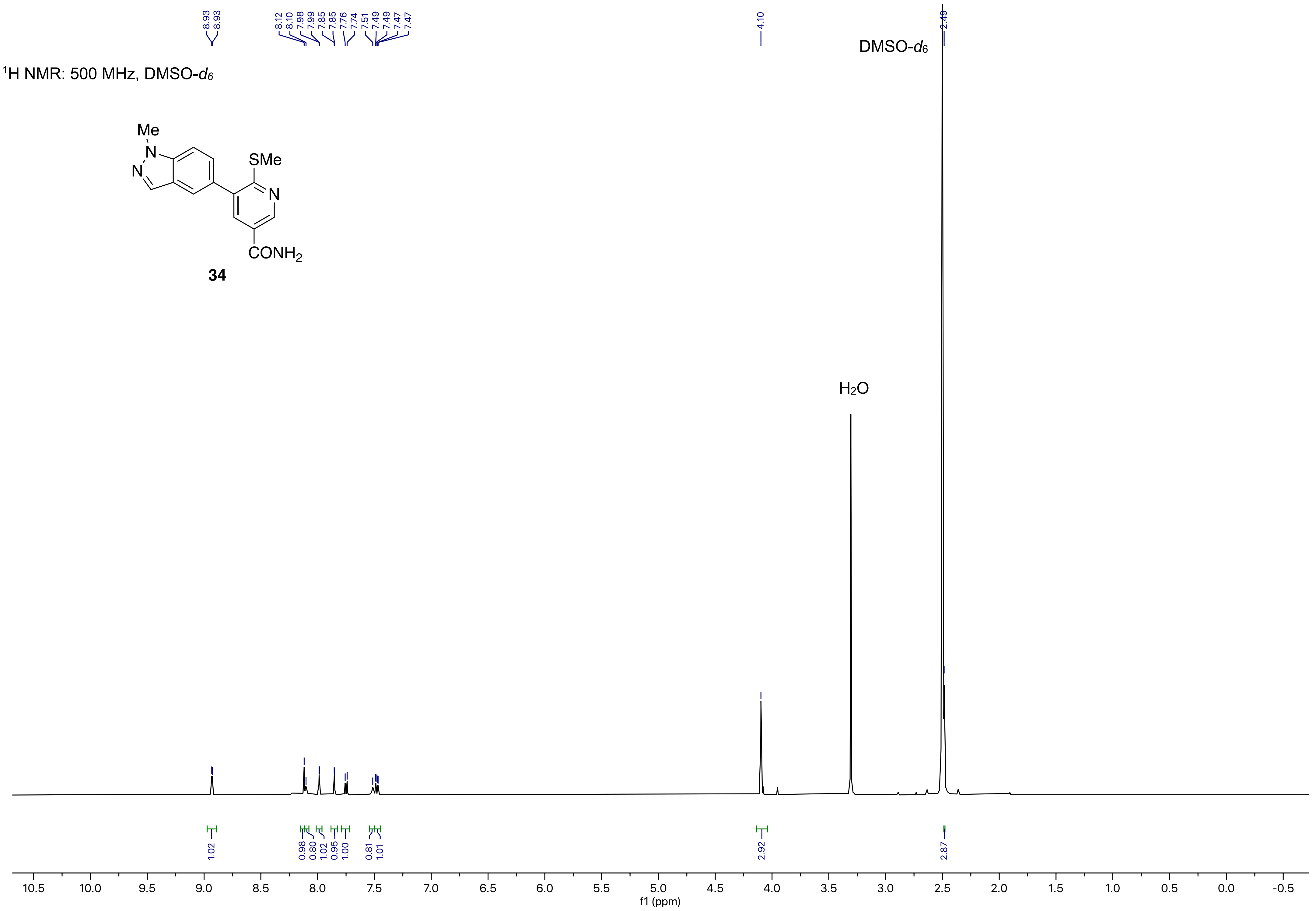

8.93
8.93
8.12
8.10
7.98
7.99
7.85
7.85
7.76
7.74
7.51
7.49
7.49
7.47
7.47
4.10
2.49
DMSO-d6
¹H NMR: 500 MHz, DMSO-d6
Me
N
N
SMe
N
CONH2
34
H2O
1.02
0.98
0.80
1.02
0.95
1.00
0.81
1.01
2.92
2.87
10.5
10.0
9.5
9.0
8.5
8.0
7.5
7.0
6.5
6.0
5.5
5.0
4.5
4.0
3.5
3.0
2.5
2.0
1.5
1.0
0.5
0.0
-0.5
f1 (ppm)

$^{13}C\{^1H\}$ NMR: 126 MHz, DMSO-d_6

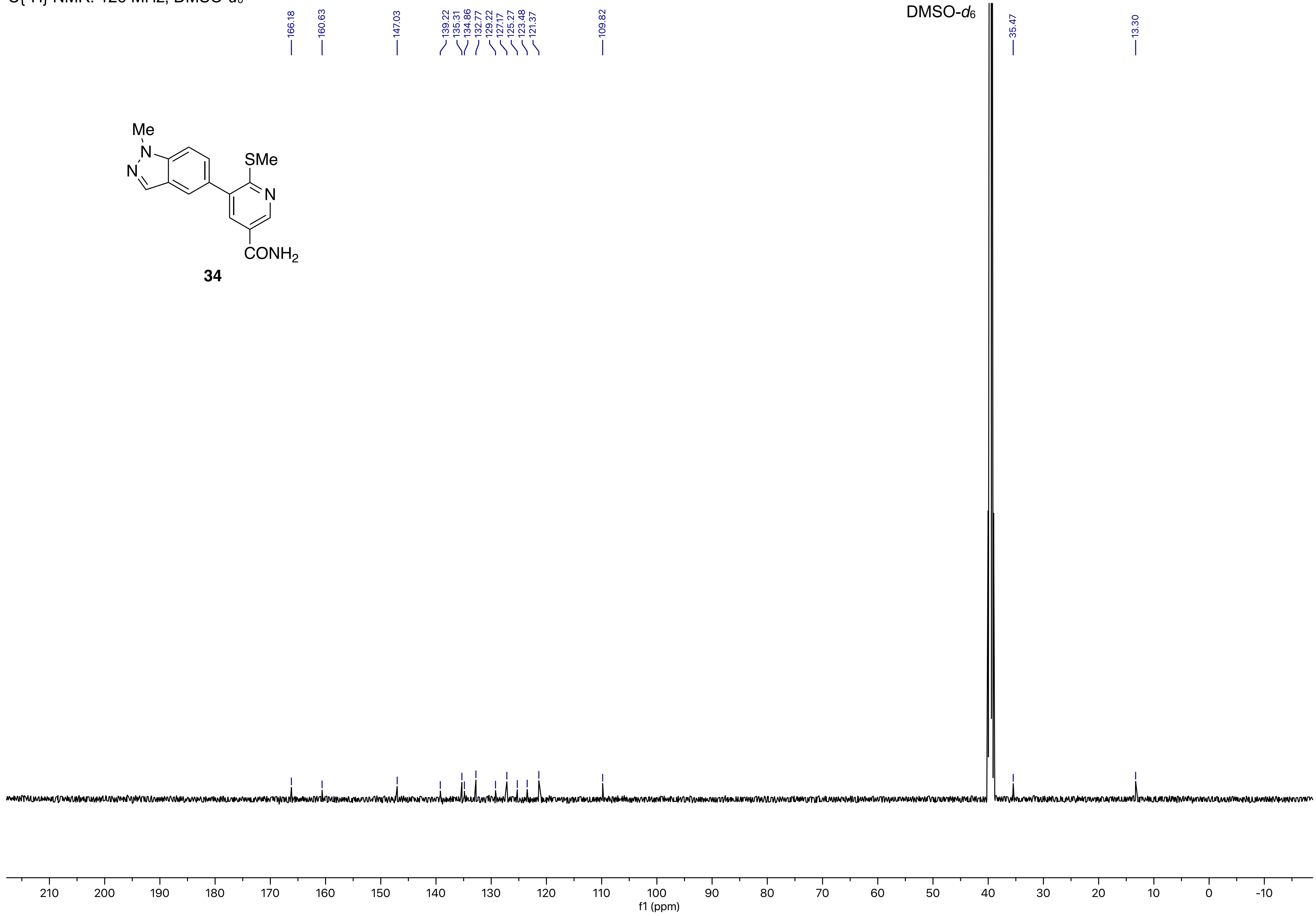

34

^{1}H NMR: 500 MHz, $CDCl_3$

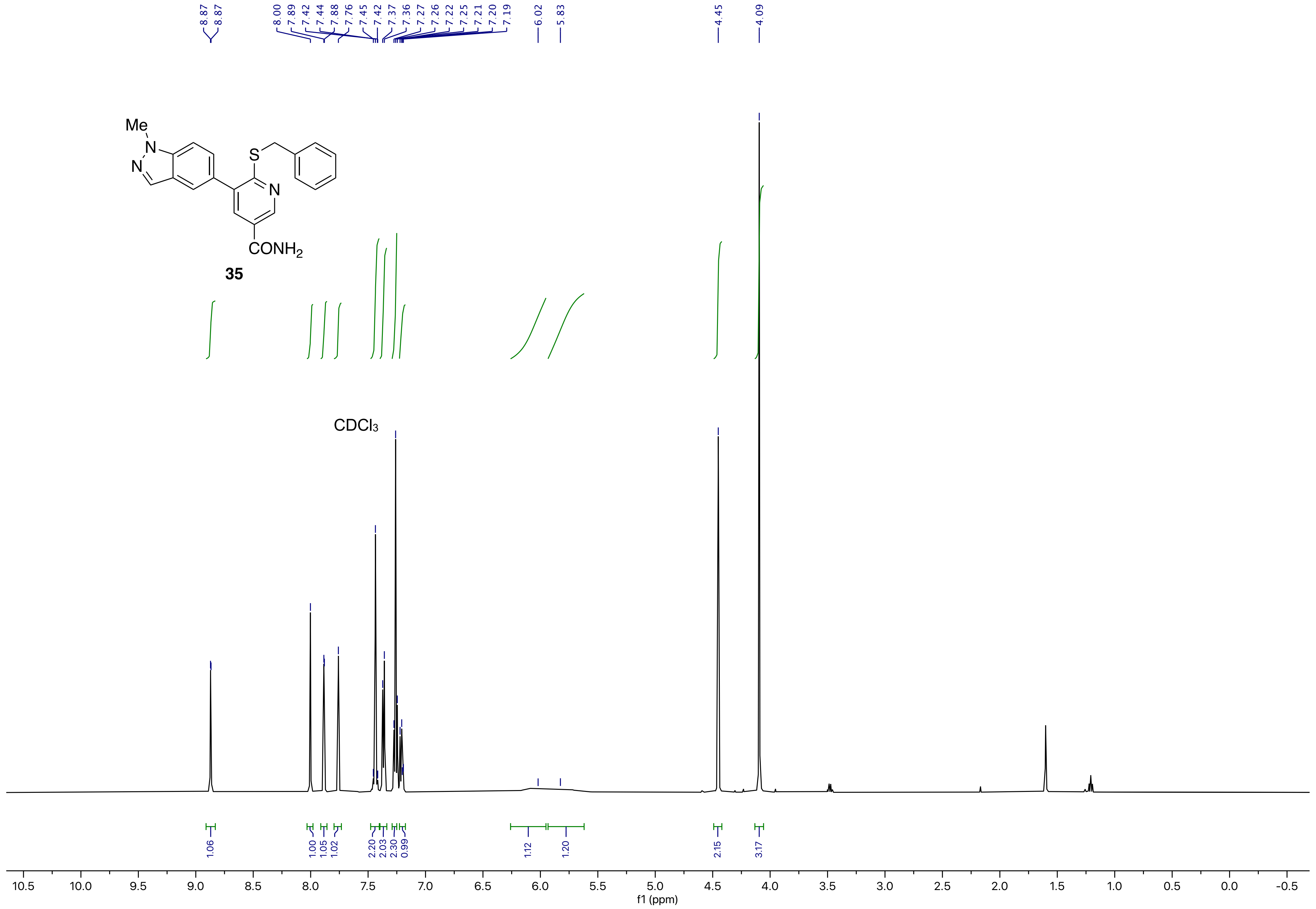

$^{13}C\{^{1}H\}$ NMR: 126 MHz, $CDCl_3$

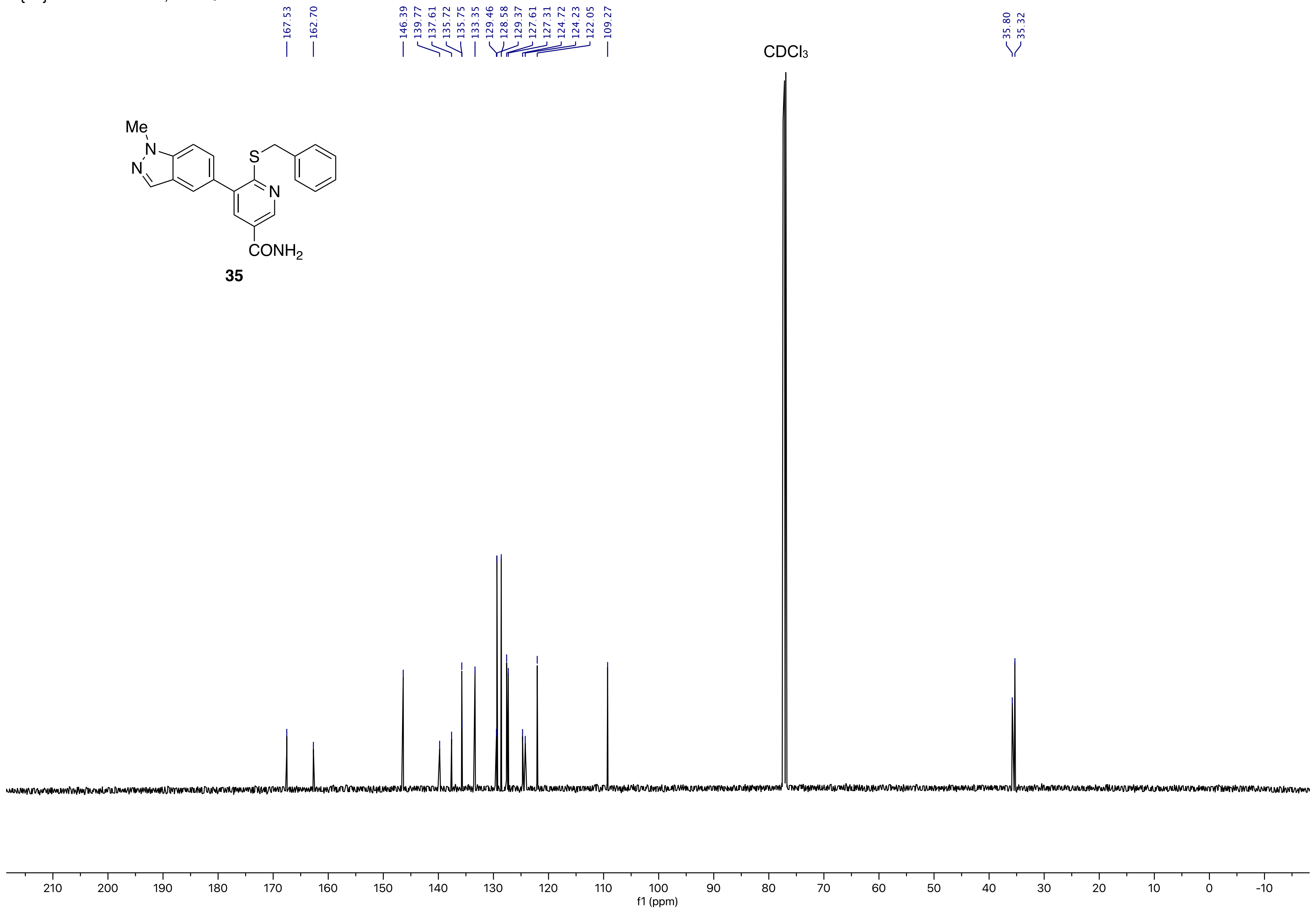

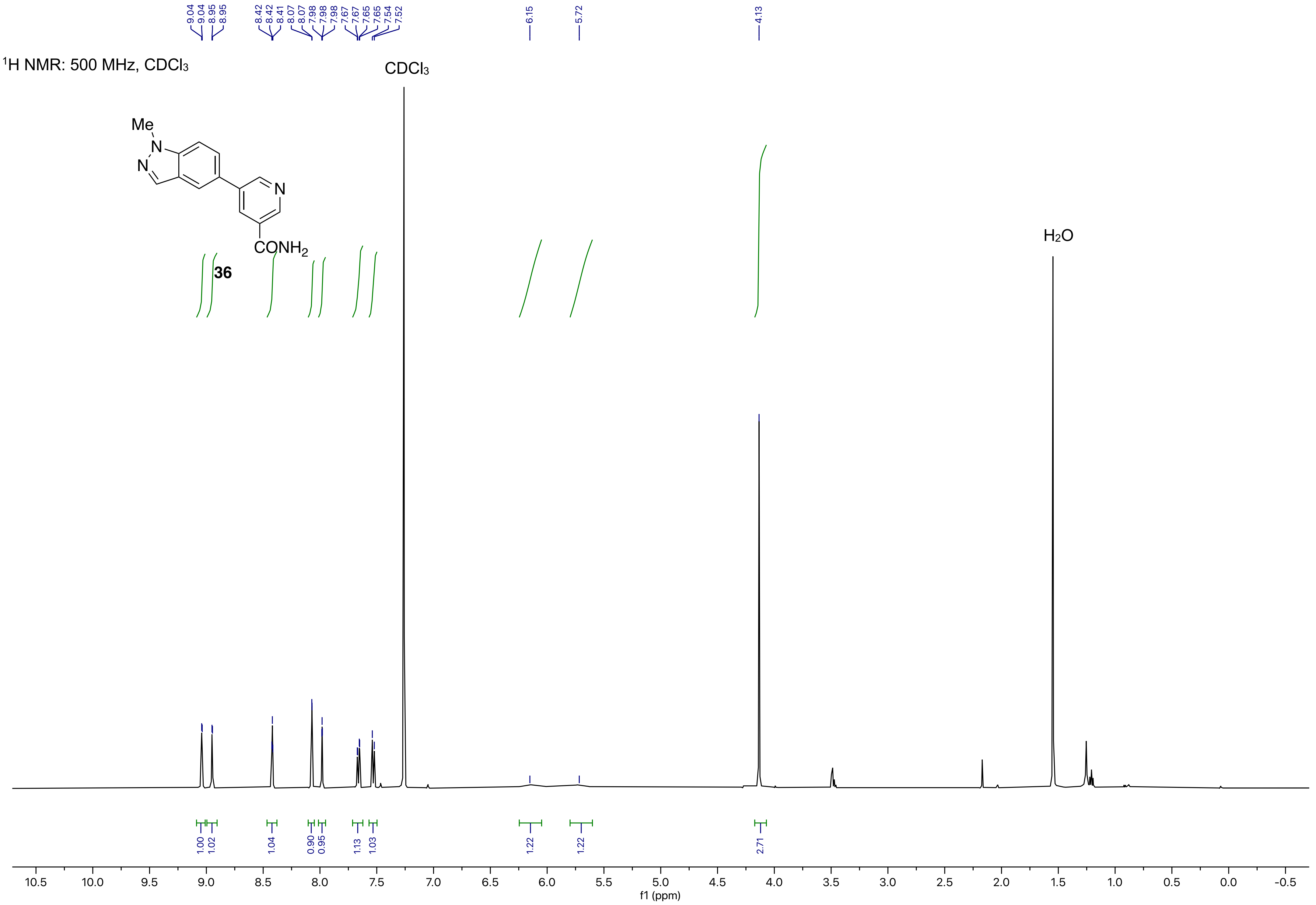

1H NMR: 500 MHz, CDCl3
Me
N
N
N
CONH2
36
CDCl3
H2O
9.04
9.04
8.95
8.95
8.42
8.42
8.41
8.07
8.07
7.98
7.98
7.67
7.67
7.65
7.65
7.54
7.52
6.15
5.72
4.13
1.00
1.02
1.04
0.90
0.95
1.13
1.03
1.22
1.22
2.71
f1 (ppm)

$^{13}C\{^{1}H\}$ NMR: 126 MHz, $CDCl_3$

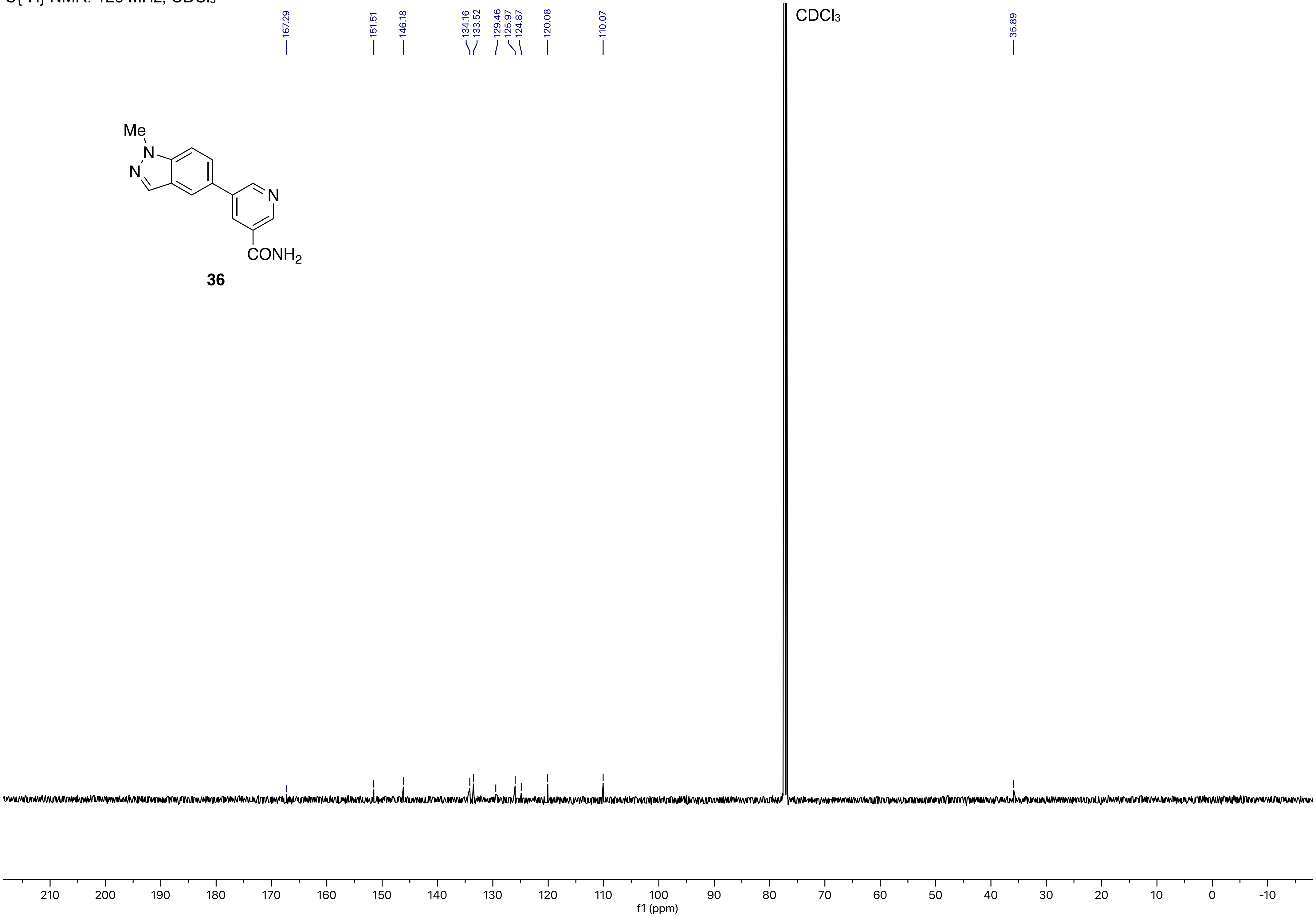

36

^1H NMR: 500 MHz, $CDCl_3$

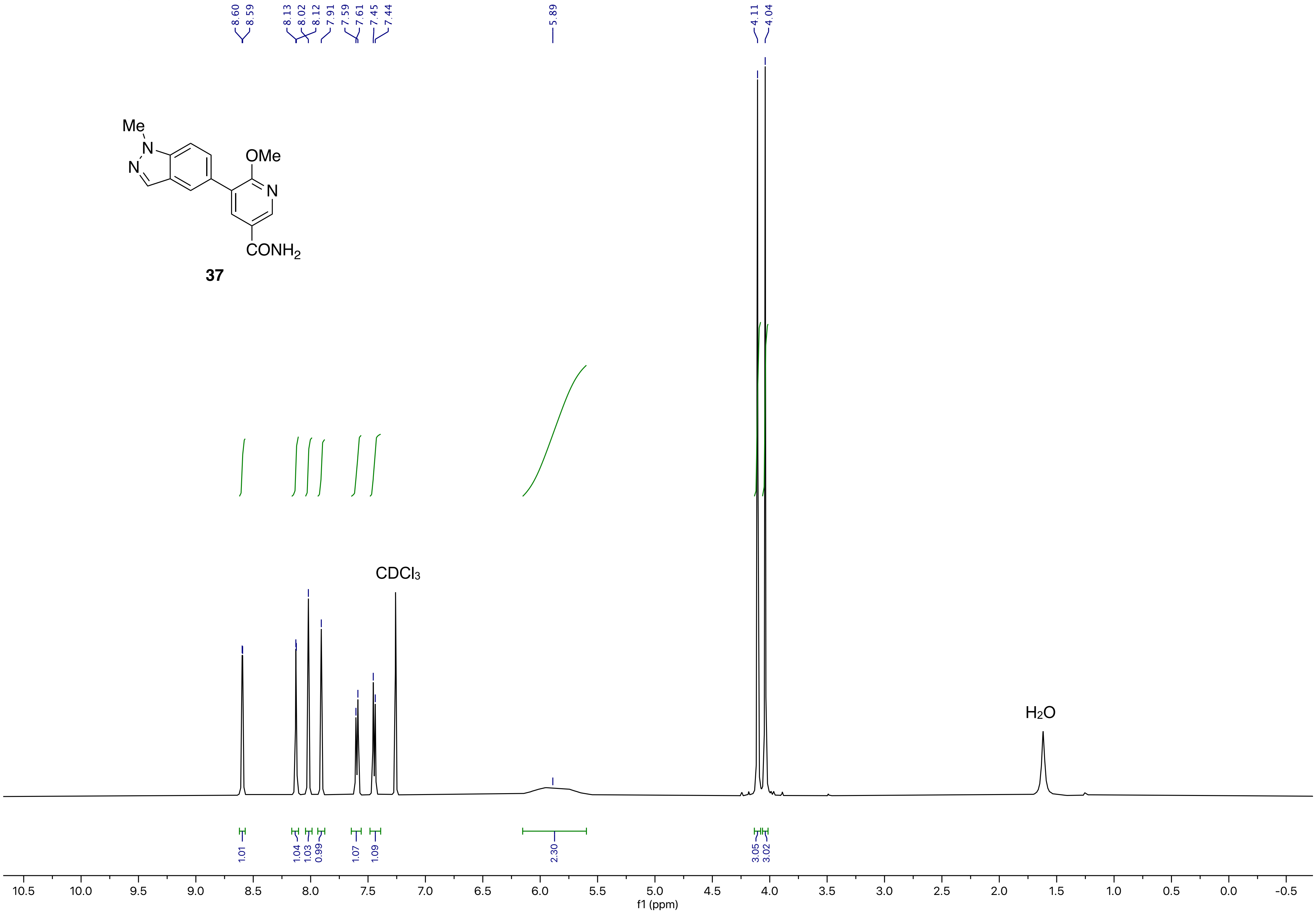

37

$^{13}C\{^1H\}$ NMR: 126 MHz, $CDCl_3$

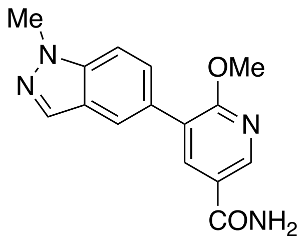

37

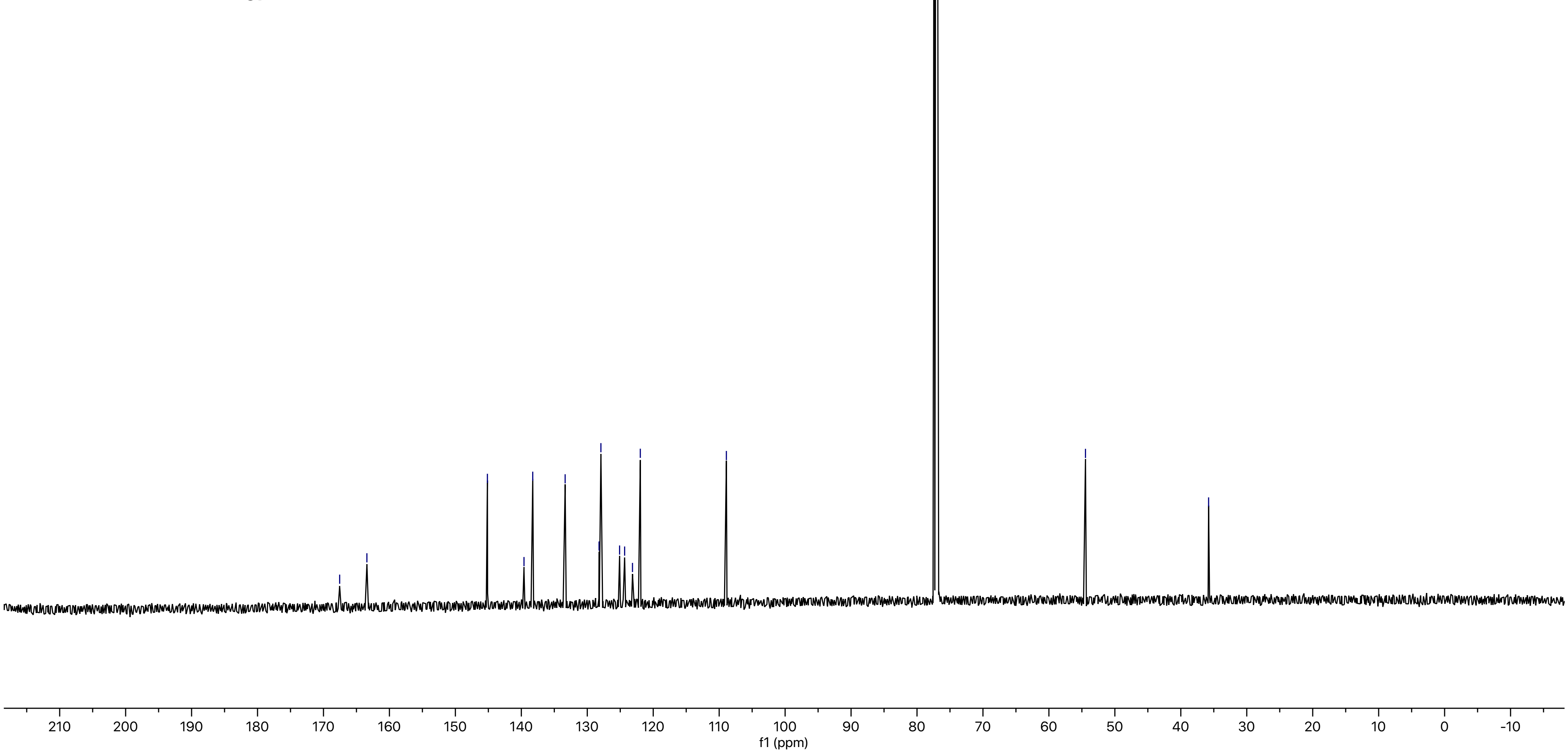

^{1}H NMR: 400 MHz, DMSO-d_6

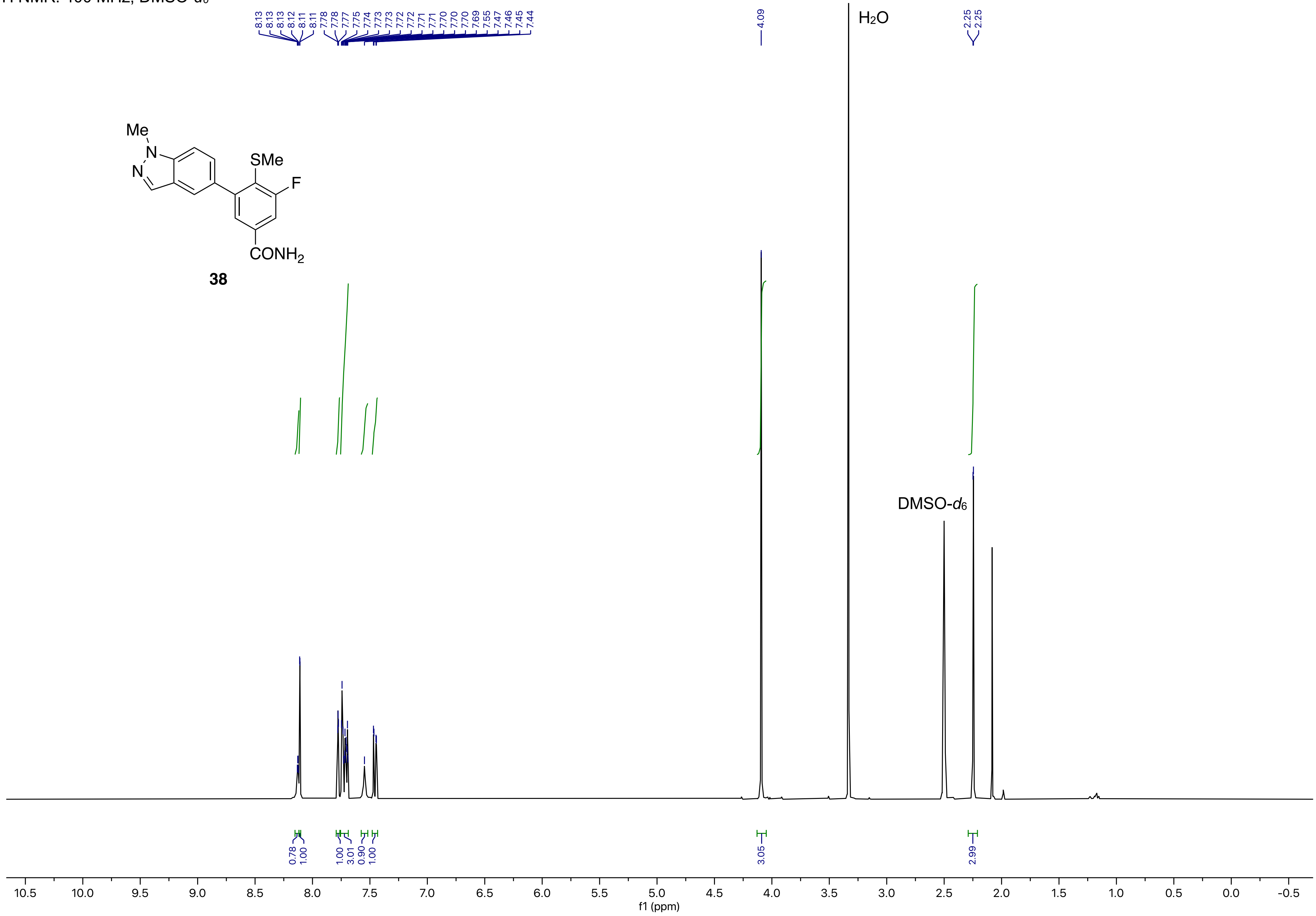

$^{13}C\{^1H\}$ NMR: 101 MHz, DMSO-d_6

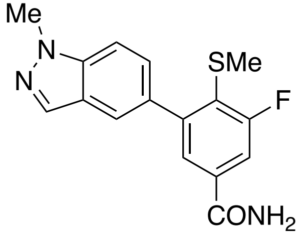

38

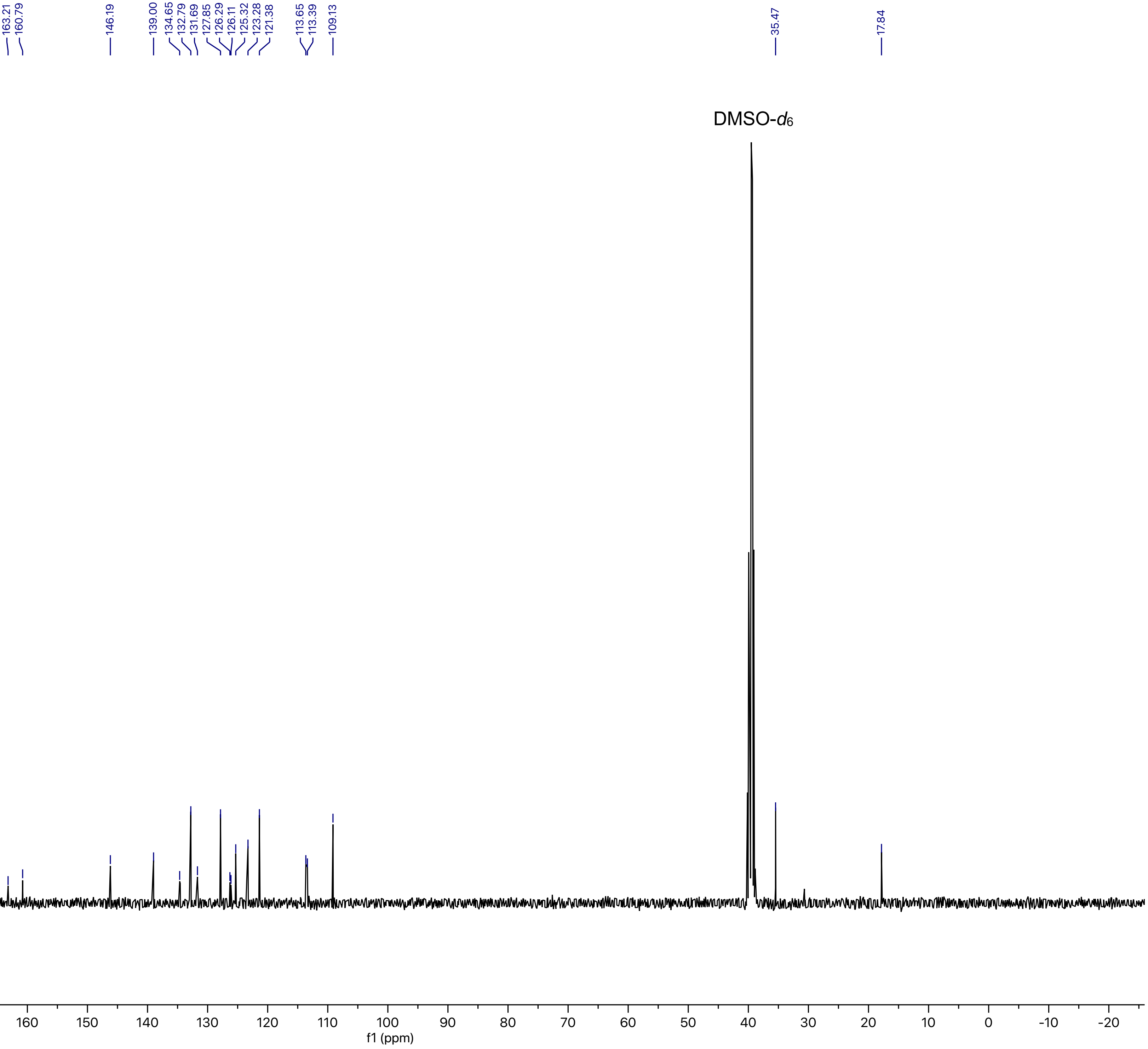

^{19}F NMR: 376 MHz, DMSO-d_6

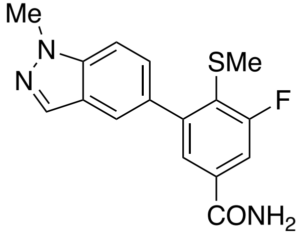

38

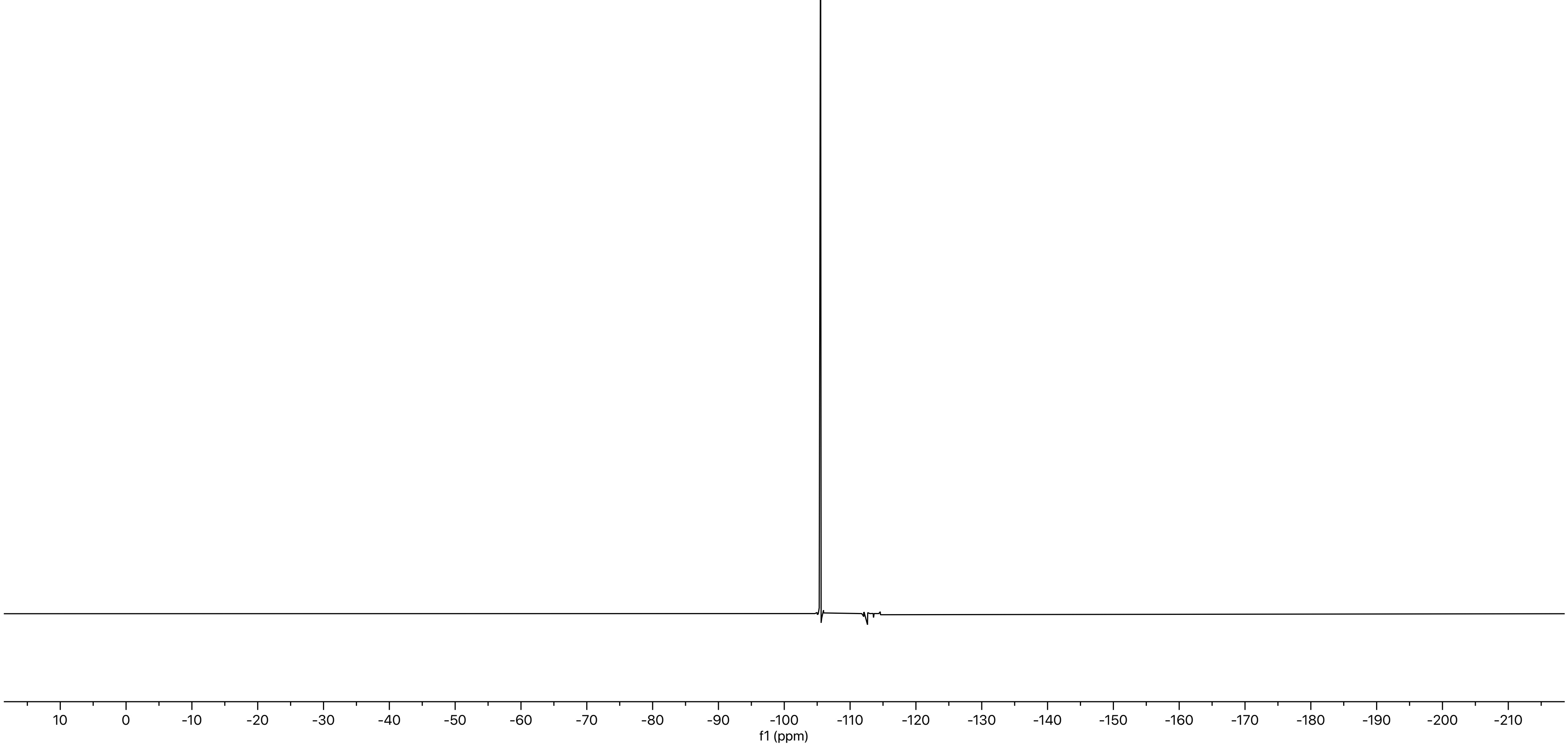

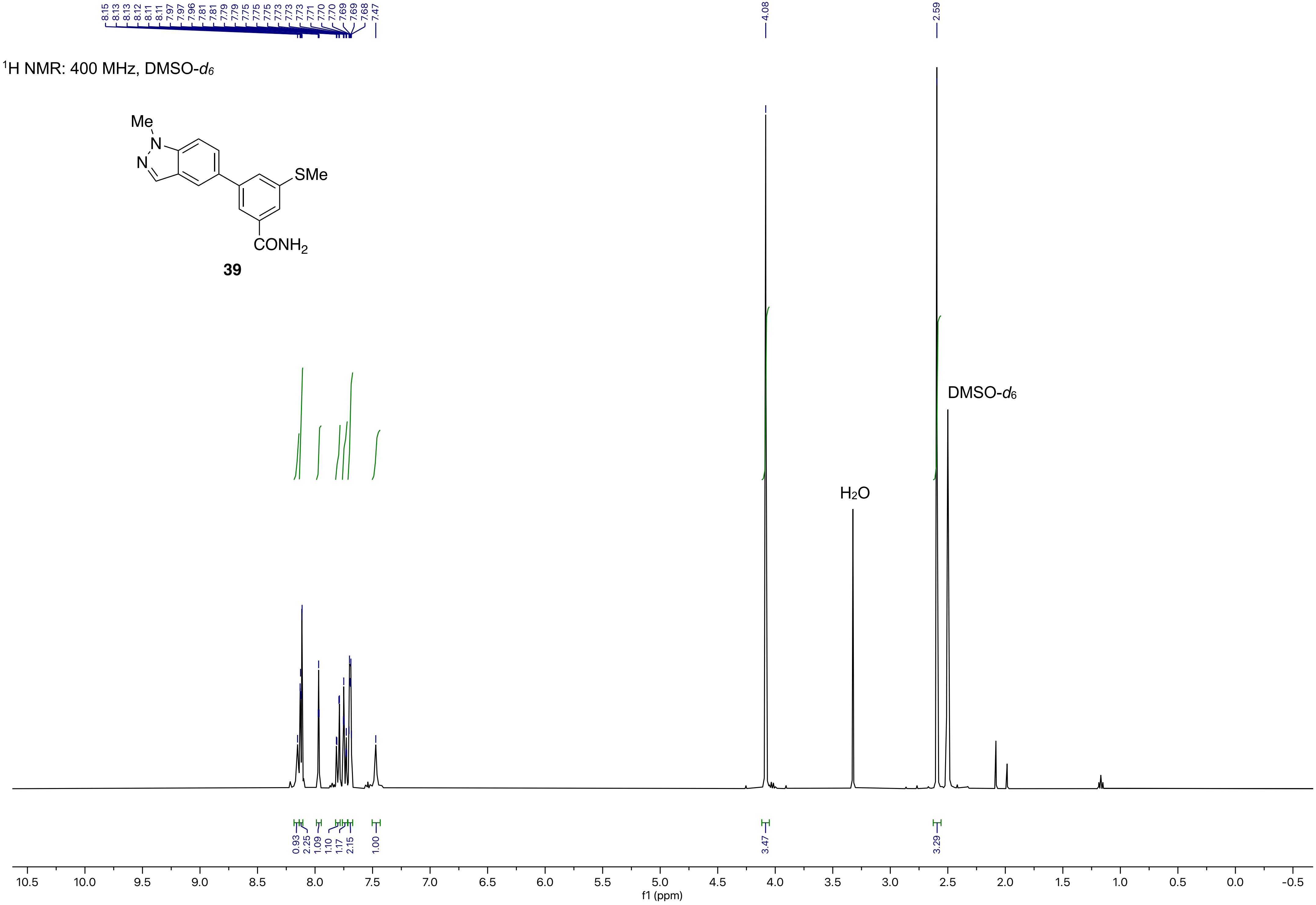

8.15 8.13 8.13 8.12 8.11 8.11 7.97 7.97 7.96 7.81 7.81 7.79 7.79 7.75 7.75 7.73 7.73 7.73 7.71 7.70 7.69 7.69 7.68 7.47
4.08
2.59
^{1}H NMR: 400 MHz, DMSO-d_6
Me
N
N
SMe
CONH$_2$
39
H$_2$O
DMSO-d_6
0.93 2.25 1.09 1.10 1.17 2.15 1.00
3.47
3.29
10.5 10.0 9.5 9.0 8.5 8.0 7.5 7.0 6.5 6.0 5.5 5.0 4.5 4.0 3.5 3.0 2.5 2.0 1.5 1.0 0.5 0.0 -0.5
f1 (ppm)

$^{13}C\{^{1}H\}$ NMR: 101 MHz, DMSO-d_6

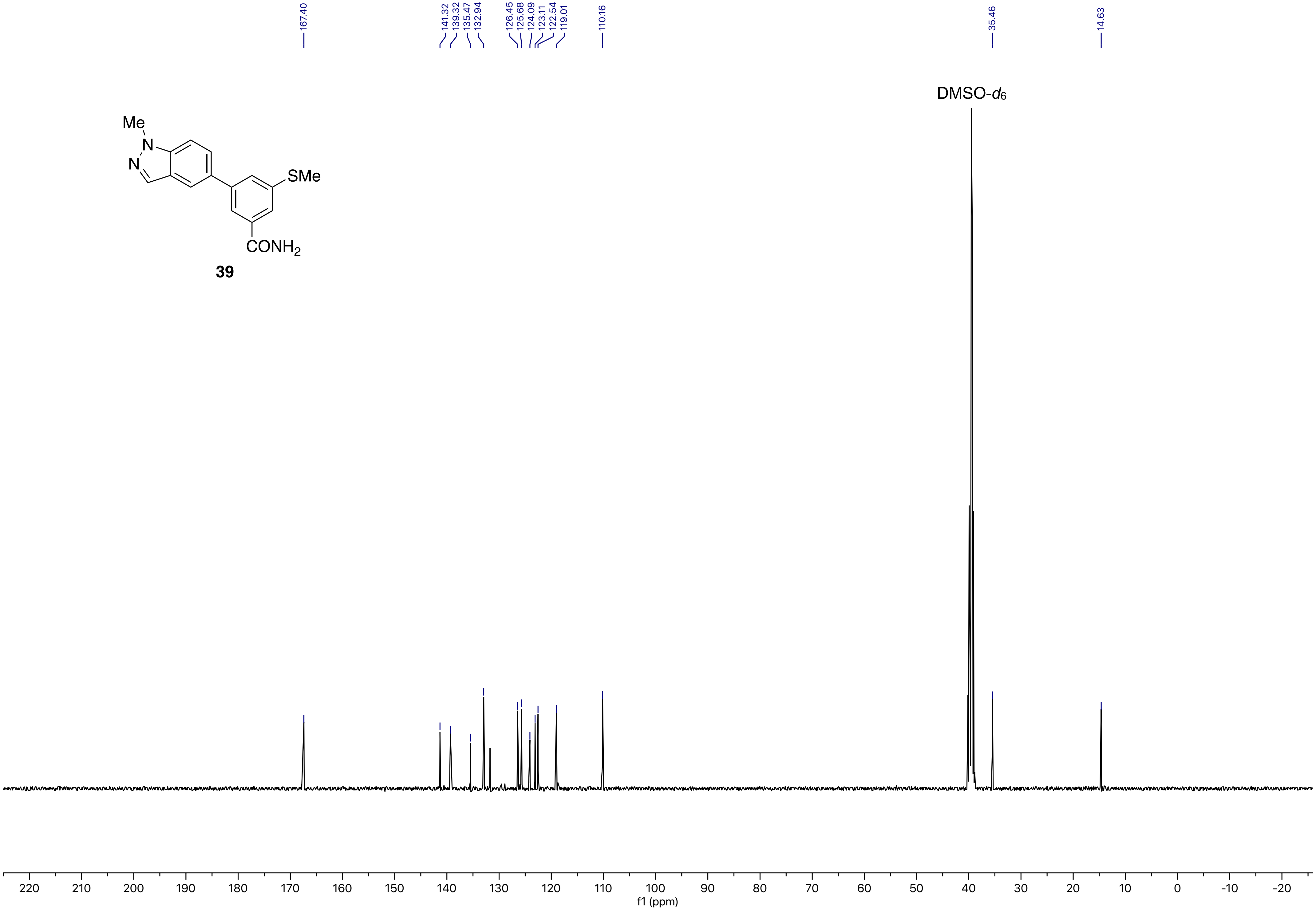

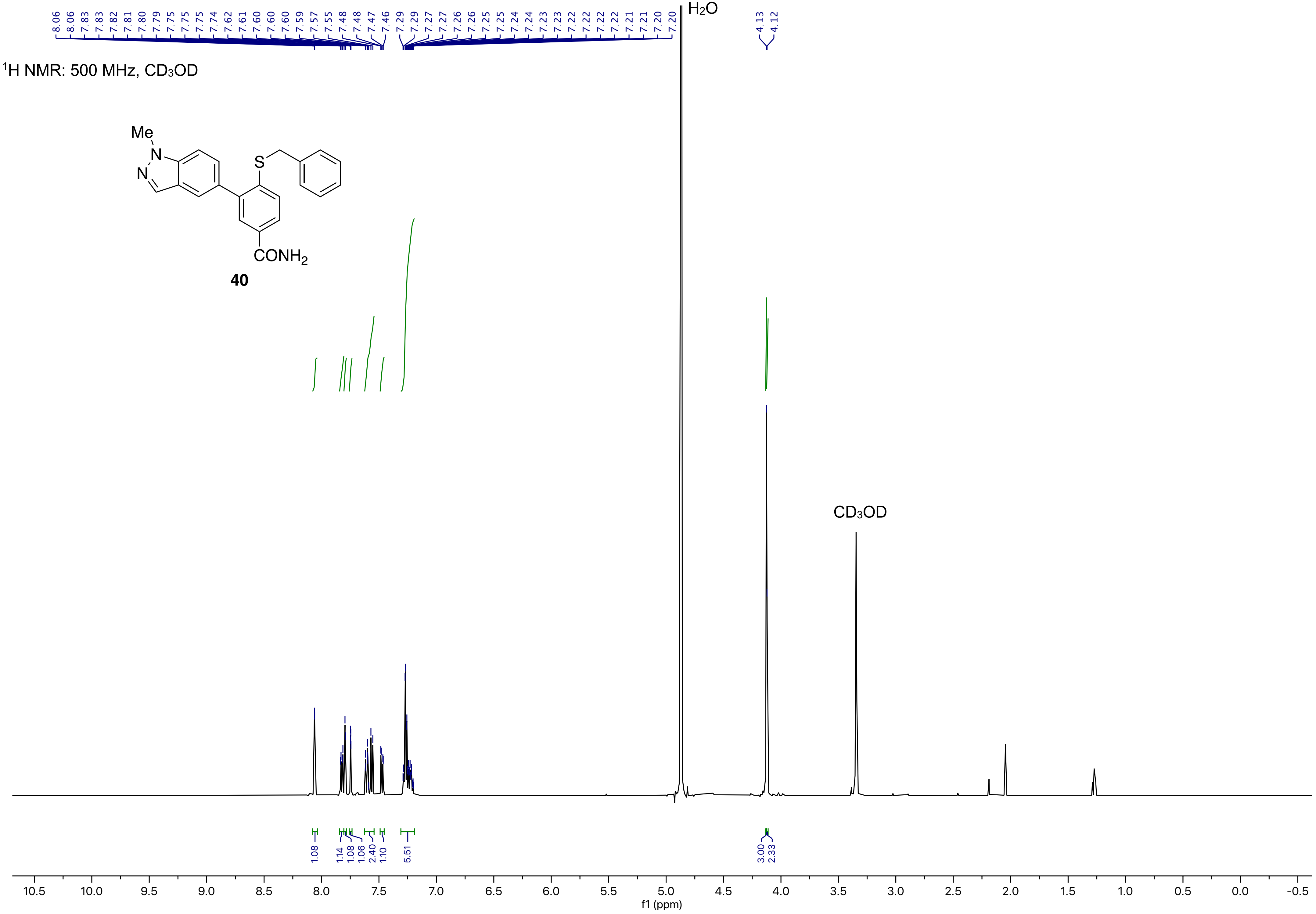

8.06 8.06 7.83 7.83 7.82 7.81 7.80 7.79 7.75 7.75 7.75 7.74 7.62 7.61 7.60 7.60 7.60 7.59 7.57 7.55 7.48 7.48 7.47 7.46 7.29 7.29 7.27 7.27 7.26 7.26 7.25 7.25 7.24 7.24 7.23 7.23 7.22 7.22 7.22 7.22 7.21 7.21 7.20 7.20
H2O
4.13 4.12
1H NMR: 500 MHz, CD3OD
Me
N
N
S
CONH2
40
CD3OD
1.08 1.14 1.08 1.06 2.40 1.10 5.51
3.00 2.33
10.5 10.0 9.5 9.0 8.5 8.0 7.5 7.0 6.5 6.0 5.5 5.0 4.5 4.0 3.5 3.0 2.5 2.0 1.5 1.0 0.5 0.0 -0.5
f1 (ppm)

$^{13}C\{^{1}H\}$ NMR: 126 MHz, CD_3OD

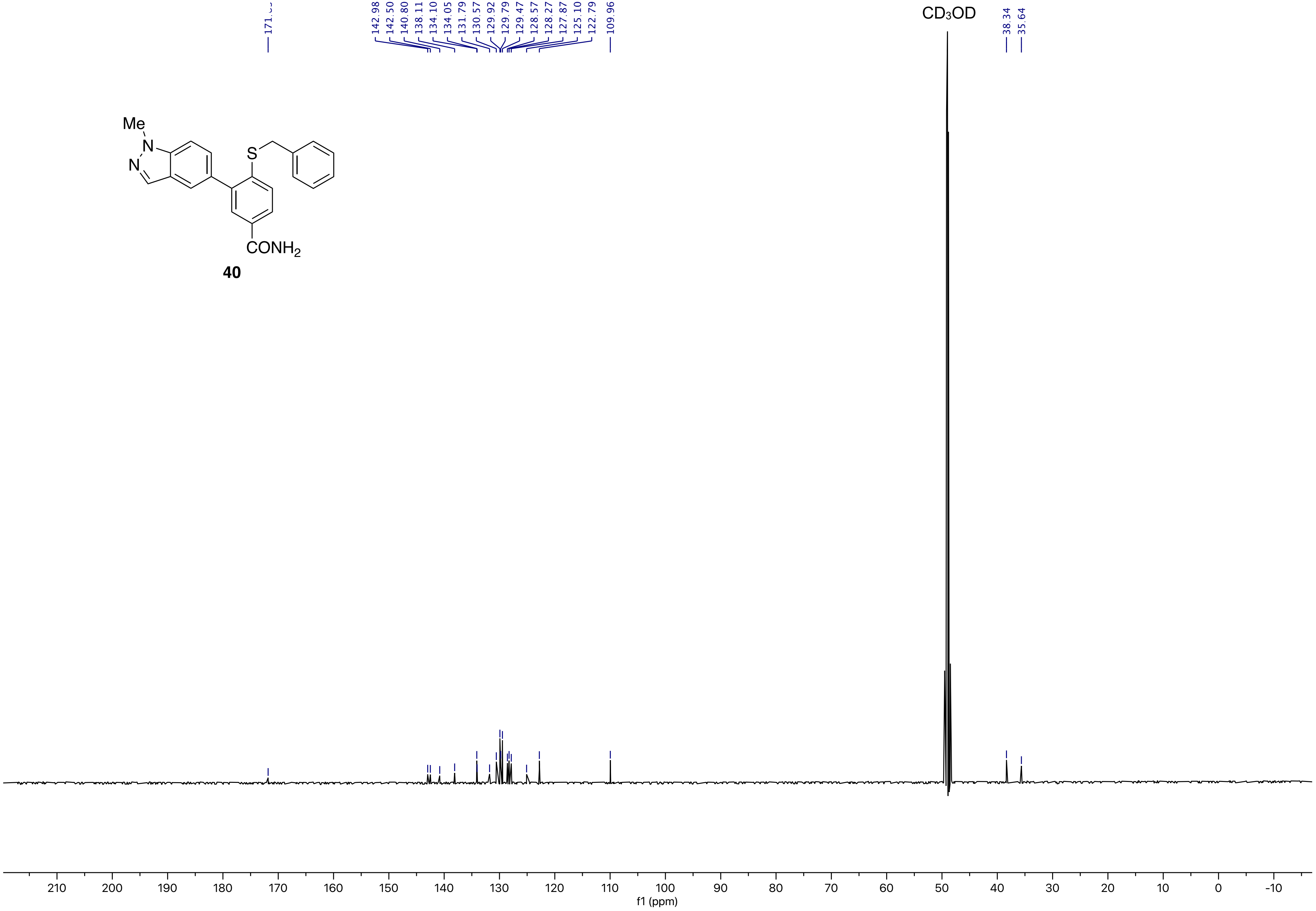

^{1}H NMR: 400 MHz, CD_3OD

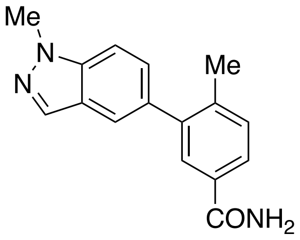

41

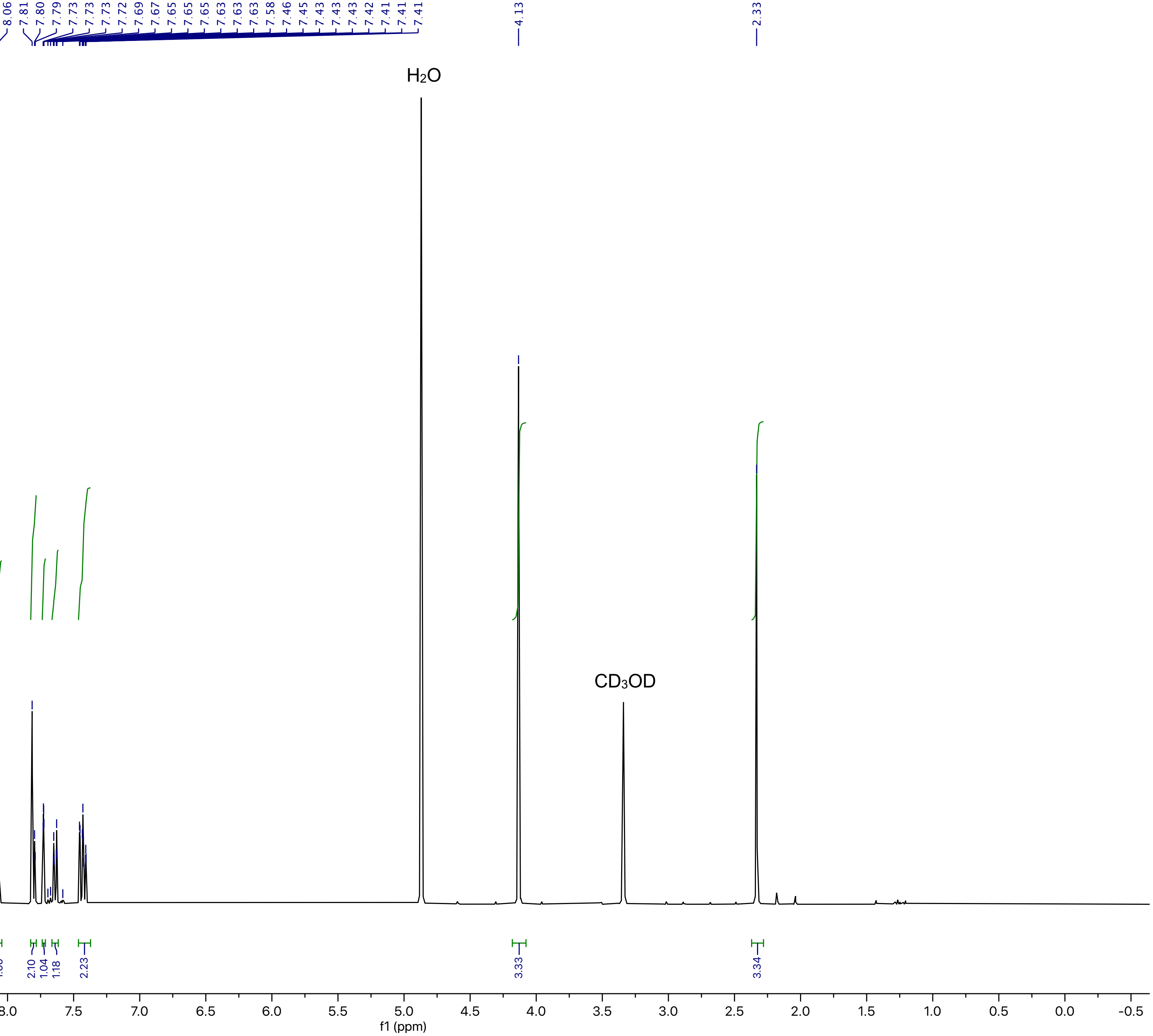

$^{13}C\{^1H\}$ NMR: 101 MHz, CD_3OD

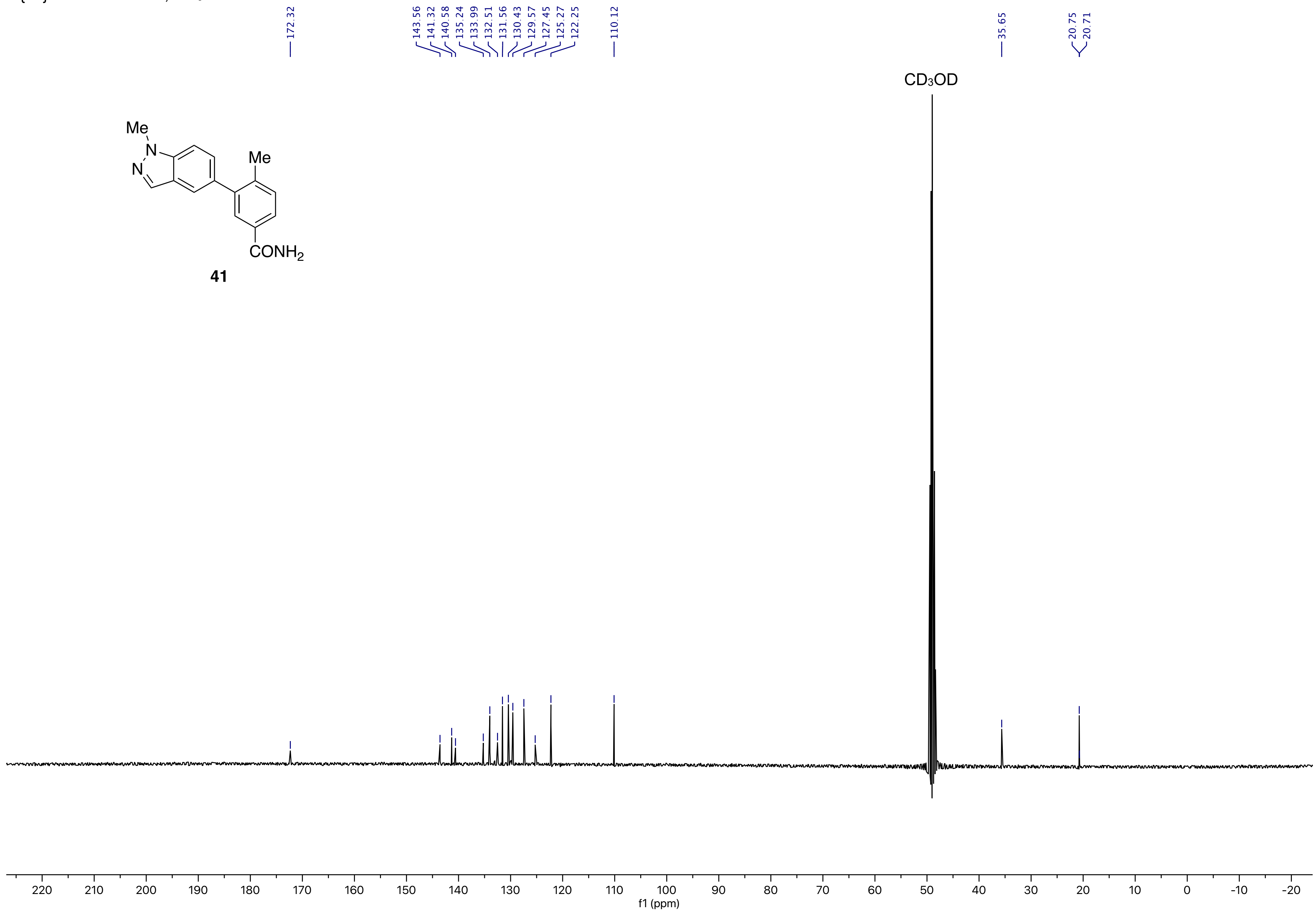

^{1}H NMR: 500 MHz, $CDCl_3$

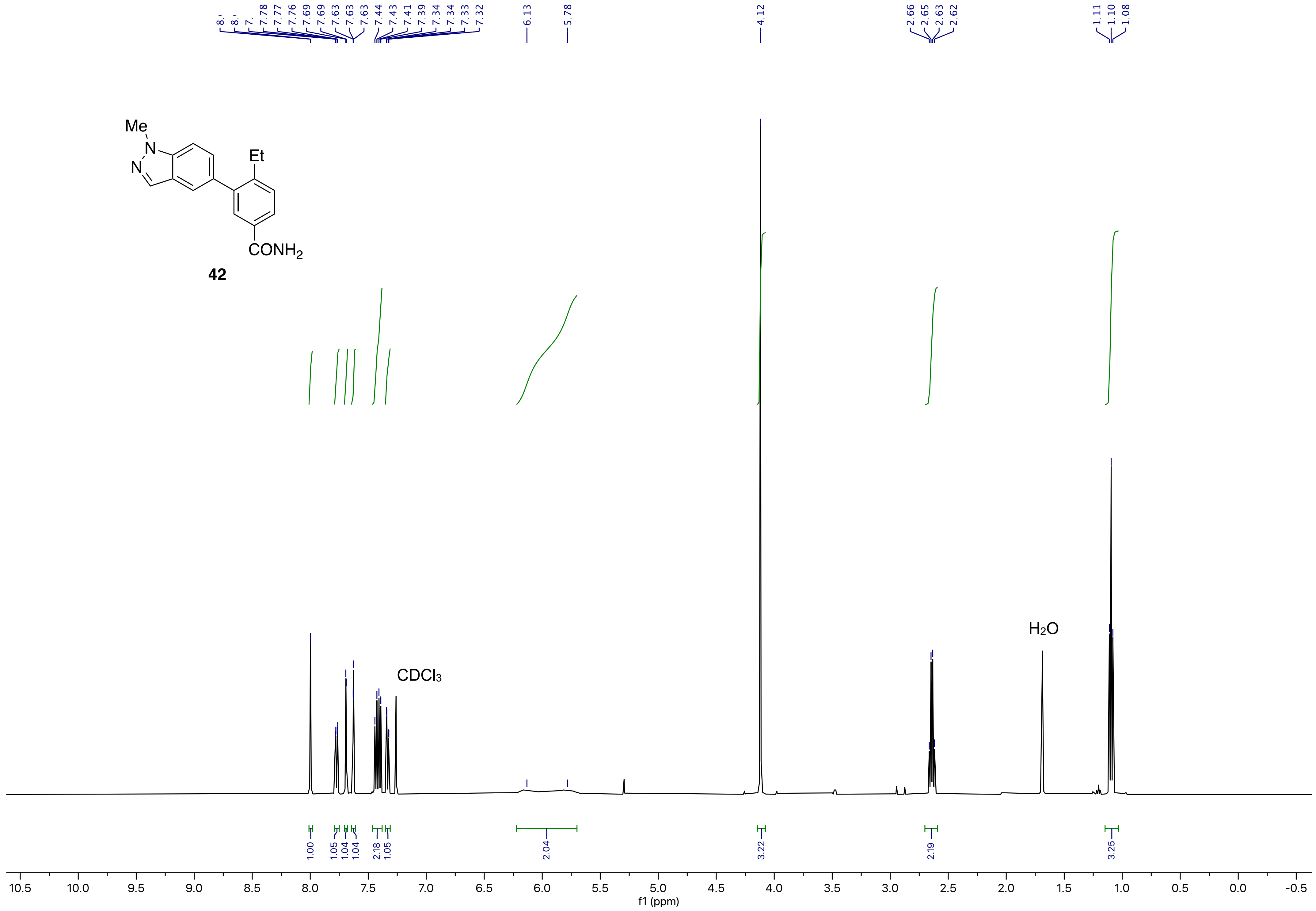

42

$^{13}C\{^{1}H\}$ NMR: 126 MHz, $CDCl_3$

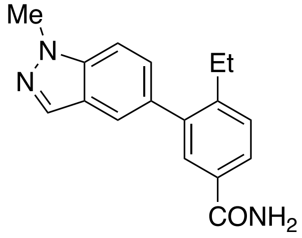

42

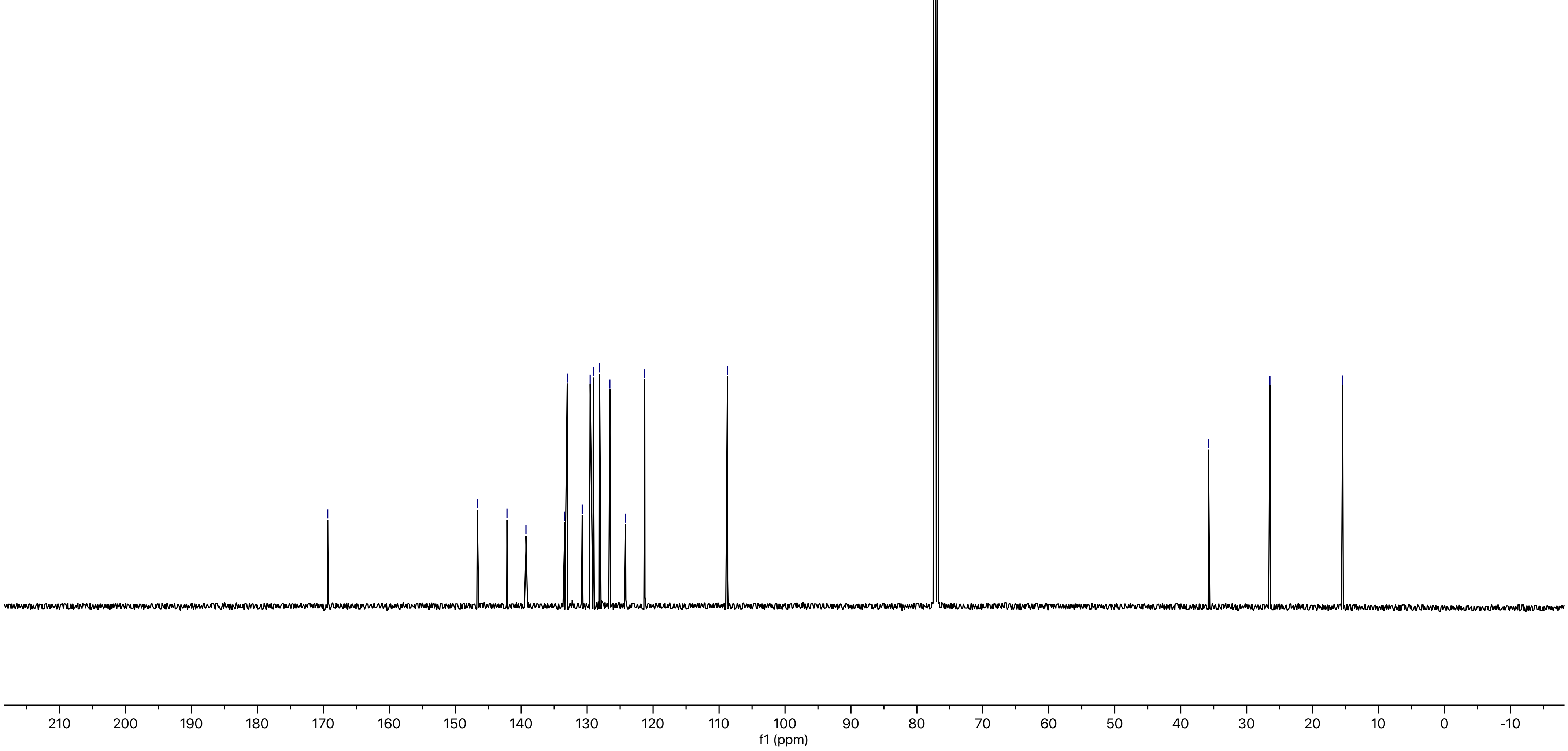

^1H NMR: 500 MHz, DMSO-d_6

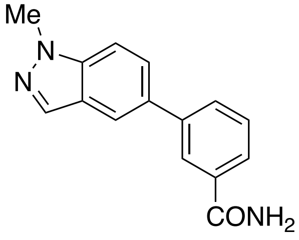

43

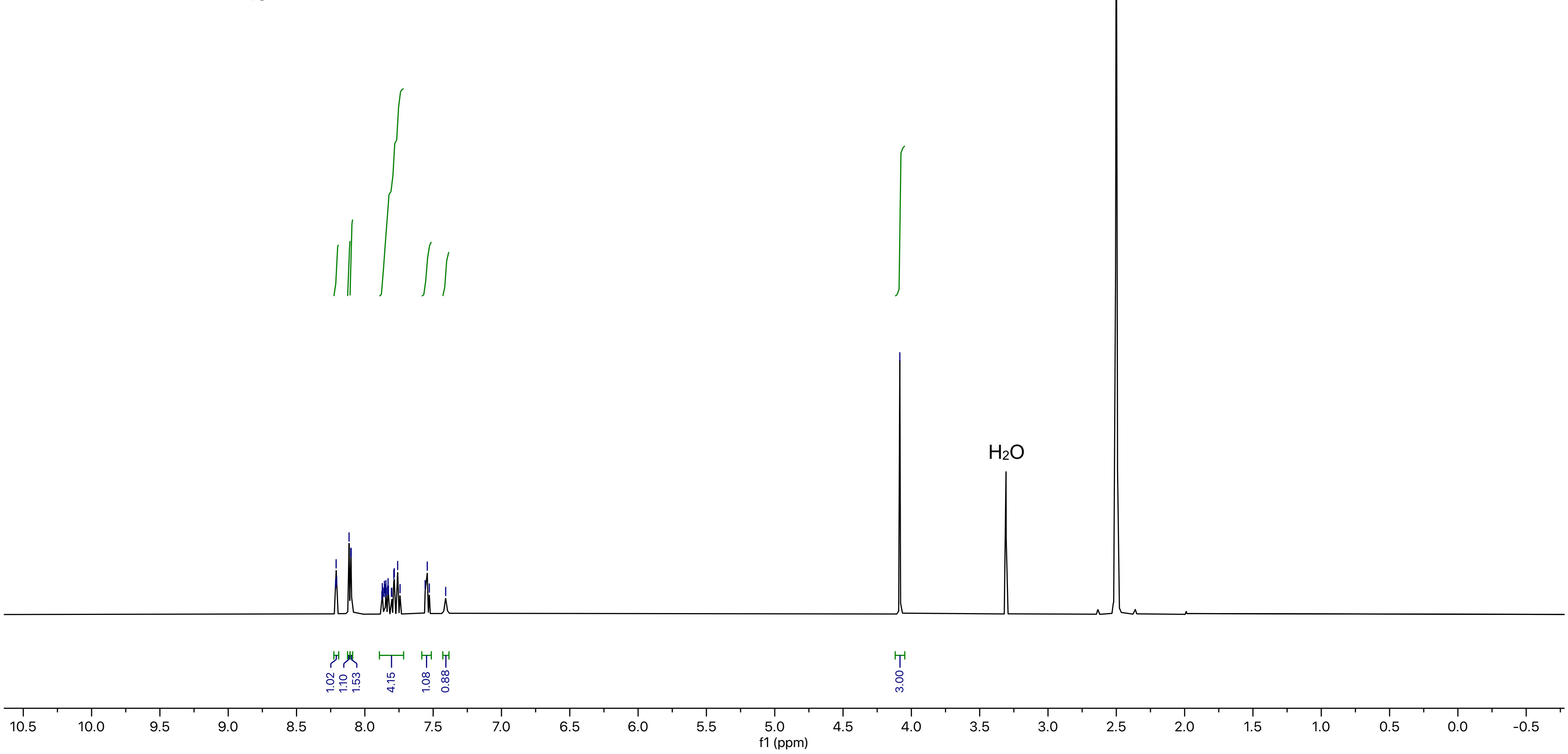

$^{13}C\{^1H\}$ NMR: 126 MHz, DMSO-d_6

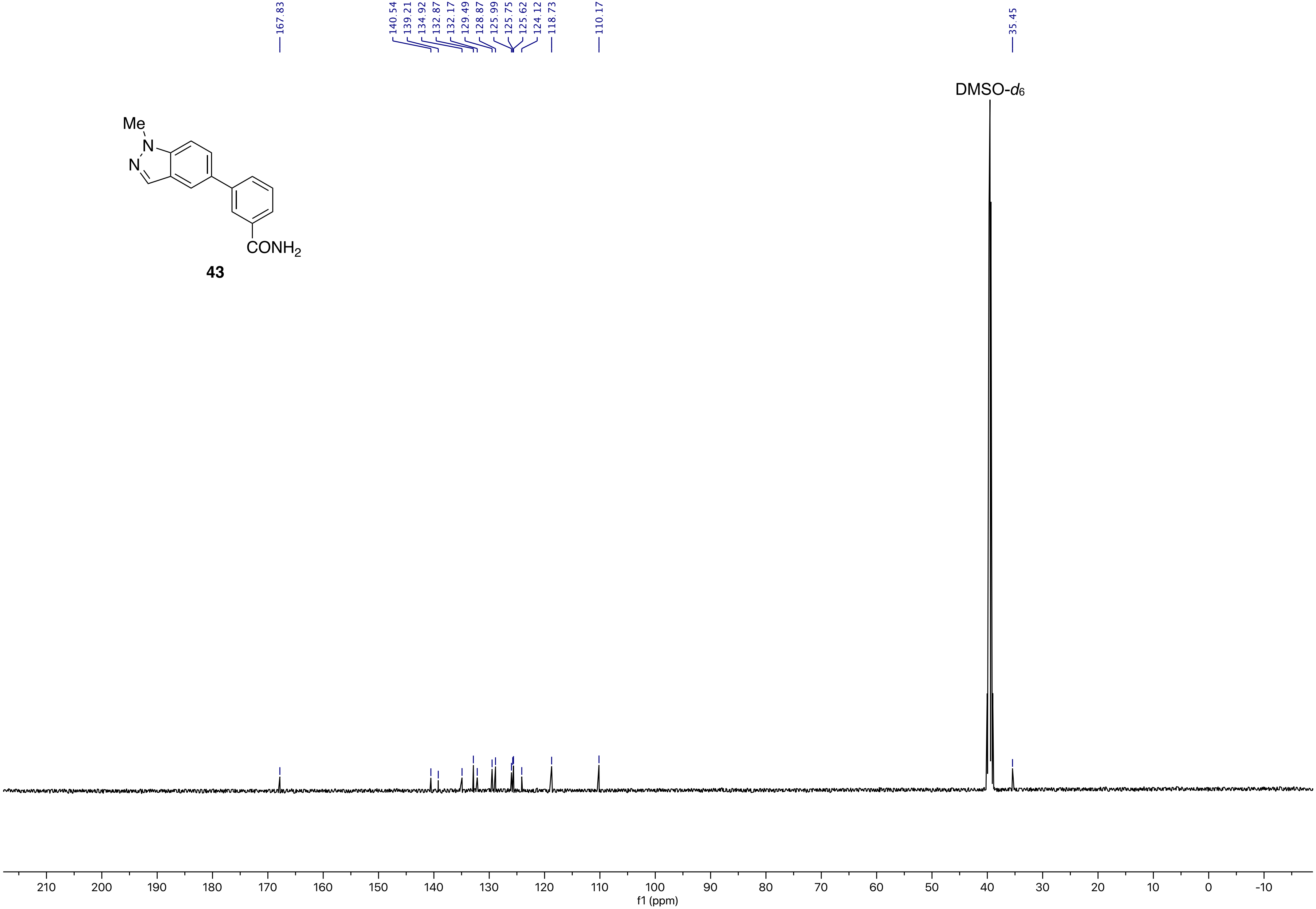

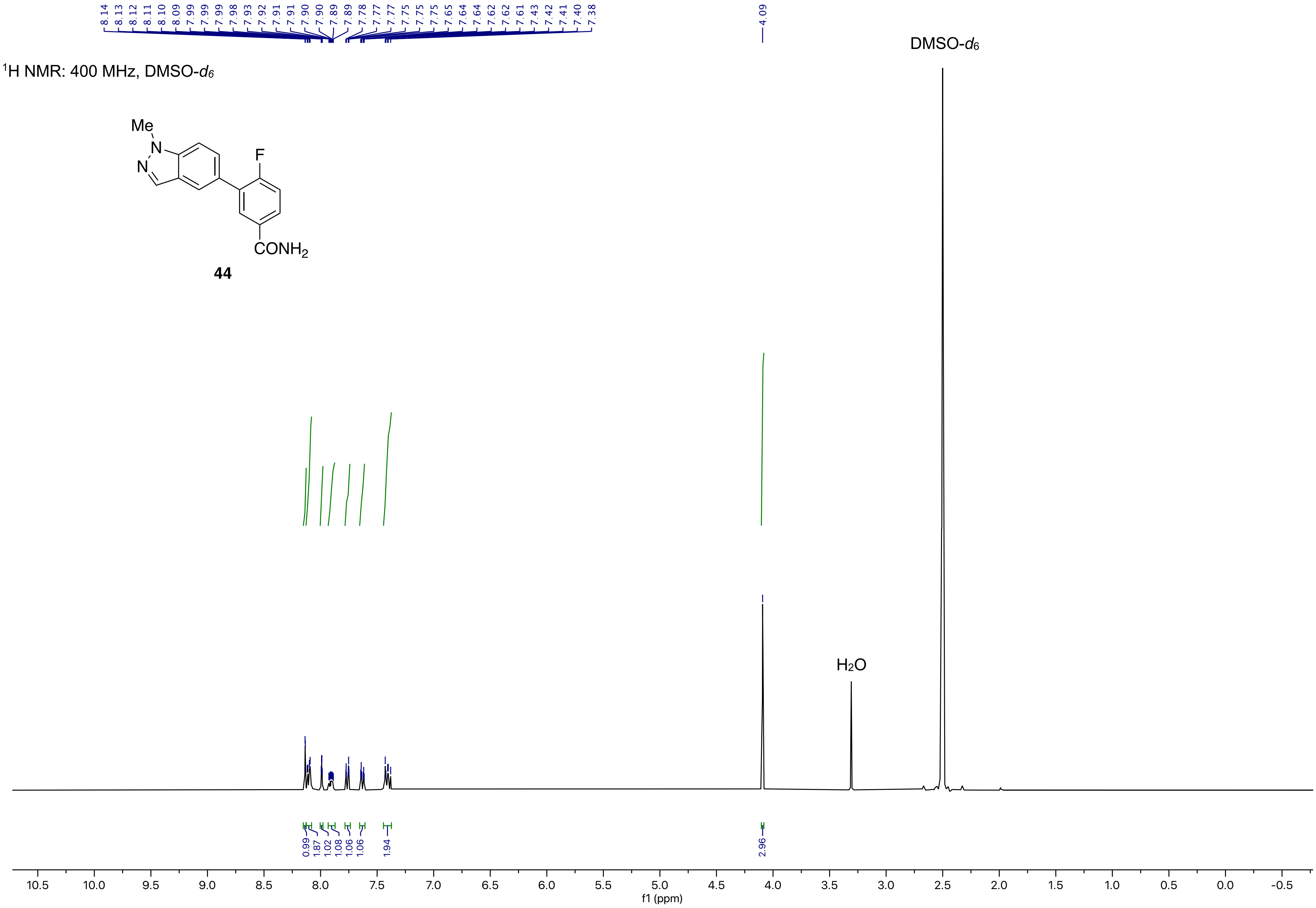
8.14 8.13 8.12 8.11 8.10 8.09 7.99 7.99 7.99 7.98 7.93 7.92 7.91 7.91 7.90 7.90 7.89 7.89 7.78 7.77 7.77 7.75 7.75 7.75 7.65 7.64 7.64 7.62 7.62 7.61 7.43 7.42 7.41 7.40 7.38
4.09
DMSO-d6
1H NMR: 400 MHz, DMSO-d6
Me
N
N
F
CONH2
44
H2O
0.99 1.87 1.02 1.08 1.06 1.06 1.94
2.96
10.5 10.0 9.5 9.0 8.5 8.0 7.5 7.0 6.5 6.0 5.5 5.0 4.5 4.0 3.5 3.0 2.5 2.0 1.5 1.0 0.5 0.0 -0.5
f1 (ppm)

$^{13}C\{^1H\}$ NMR: 101 MHz, DMSO-d_6

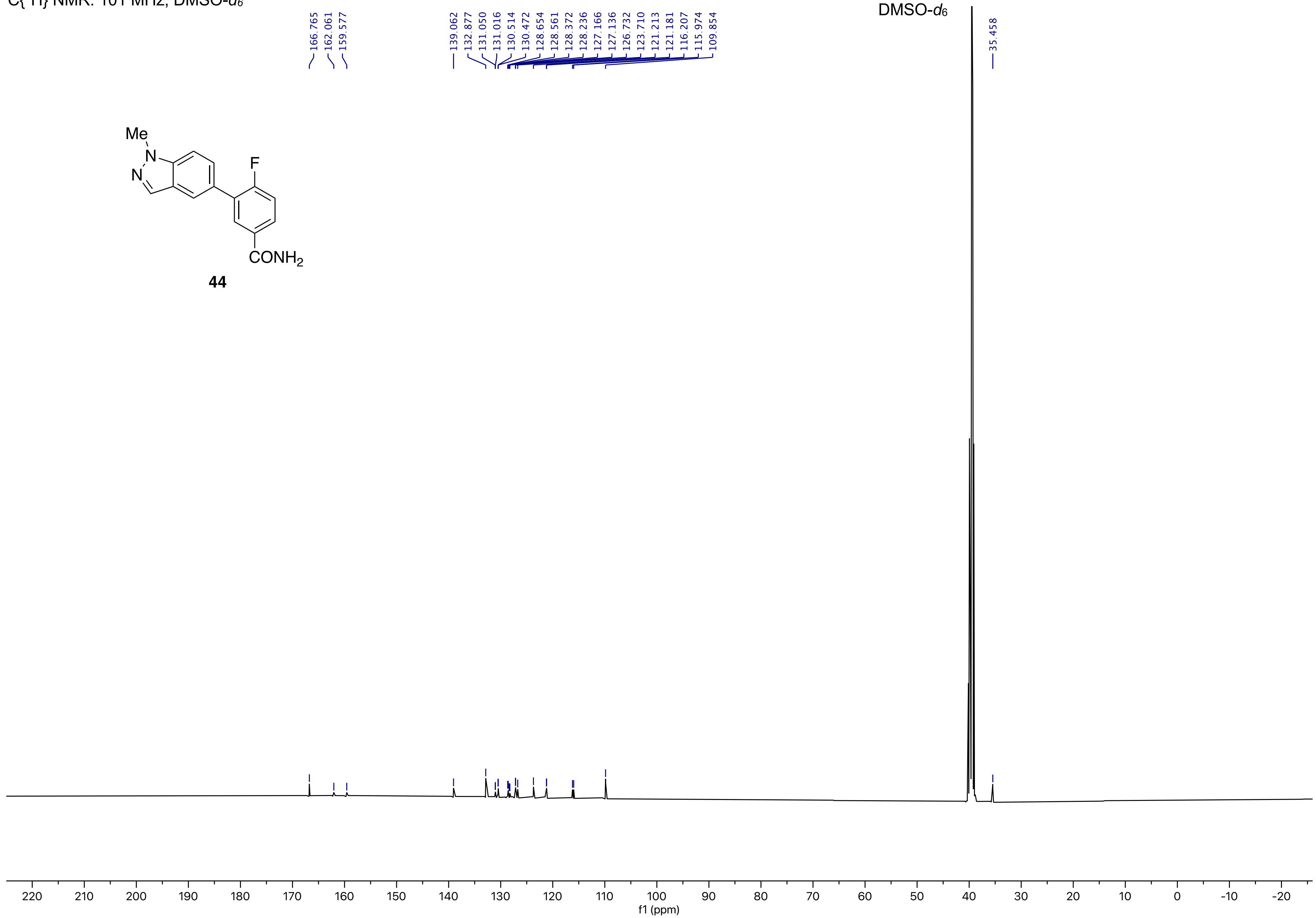

^{19}F NMR: 376 MHz, DMSO-d_6

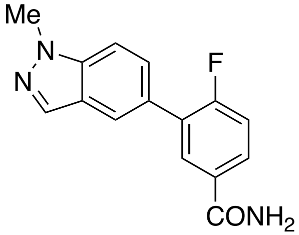

44

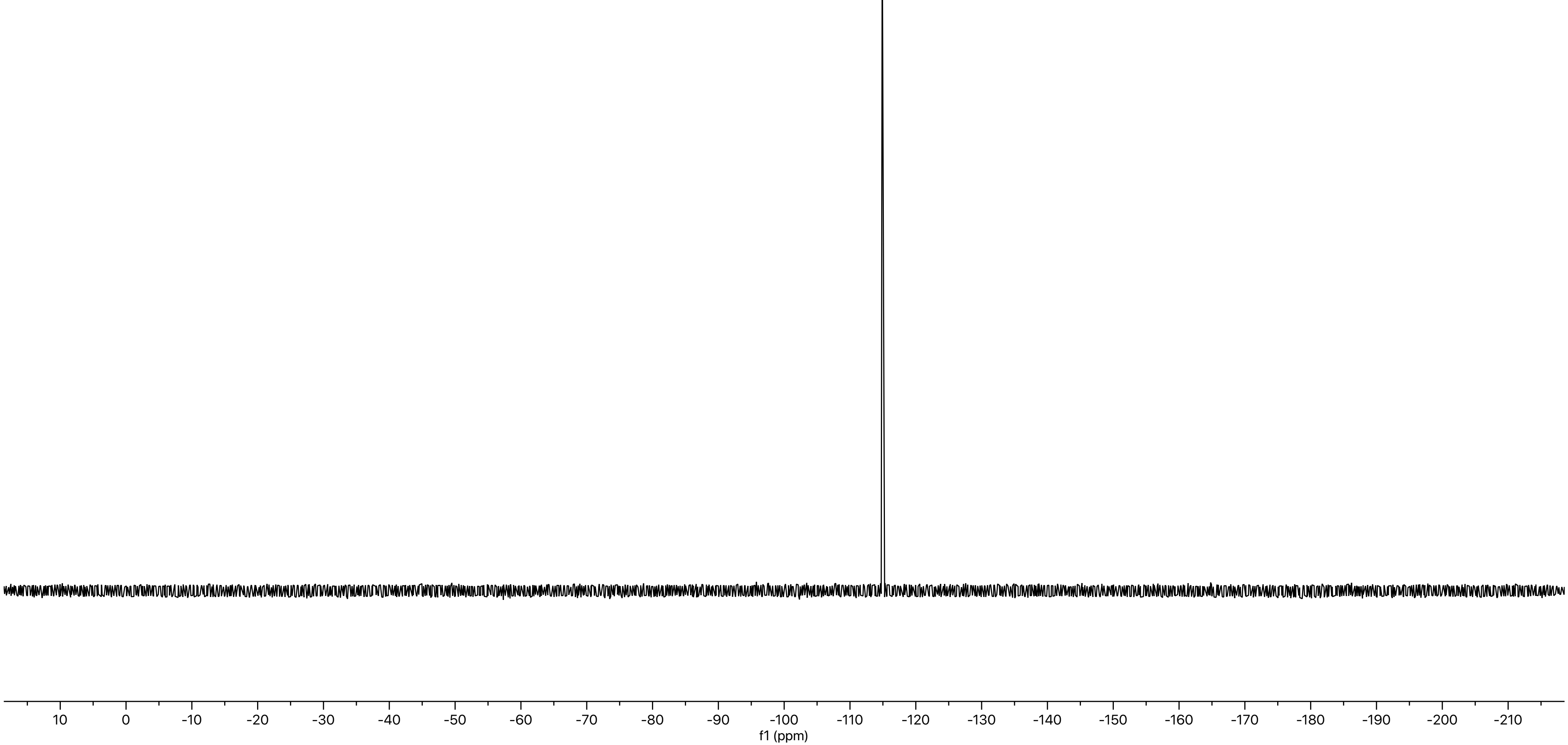

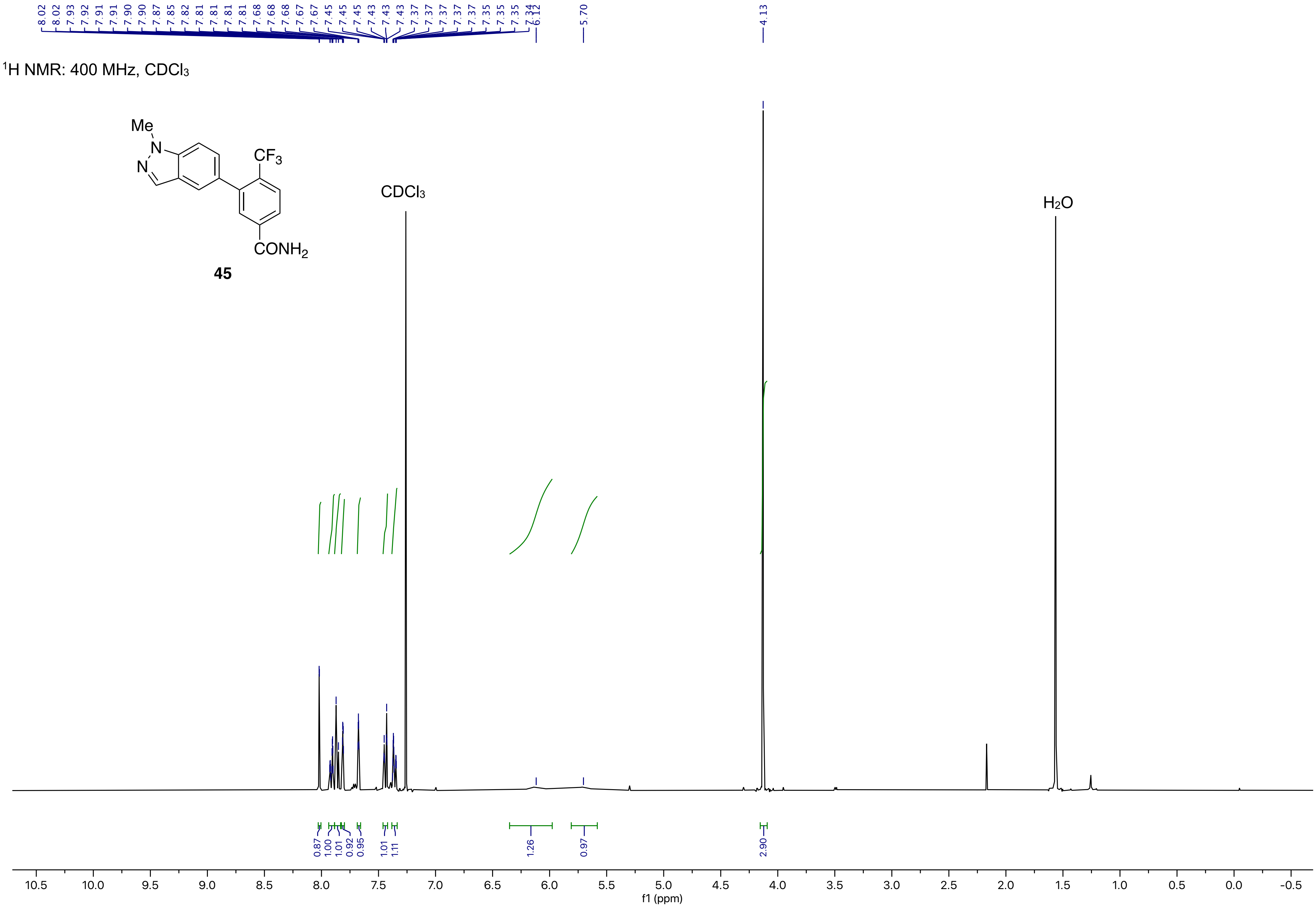

8.02
8.02
7.93
7.92
7.91
7.91
7.90
7.90
7.87
7.85
7.82
7.81
7.81
7.81
7.81
7.68
7.68
7.68
7.67
7.67
7.45
7.45
7.45
7.43
7.43
7.43
7.37
7.37
7.37
7.37
7.37
7.35
7.35
7.35
7.34
6.12
5.70
4.13
¹H NMR: 400 MHz, CDCl3
Me
N
N
CF3
CONH2
45
CDCl3
H2O
0.87
1.00
1.01
0.92
0.95
1.01
1.11
1.26
0.97
2.90
10.5
10.0
9.5
9.0
8.5
8.0
7.5
7.0
6.5
6.0
5.5
5.0
4.5
4.0
3.5
3.0
2.5
2.0
1.5
1.0
0.5
0.0
-0.5
f1 (ppm)

$^{13}C\{^1H\}$ NMR: 101 MHz, $CDCl_3$

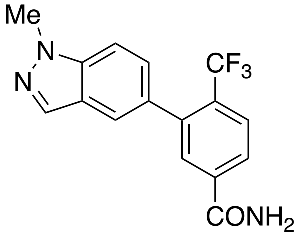

45

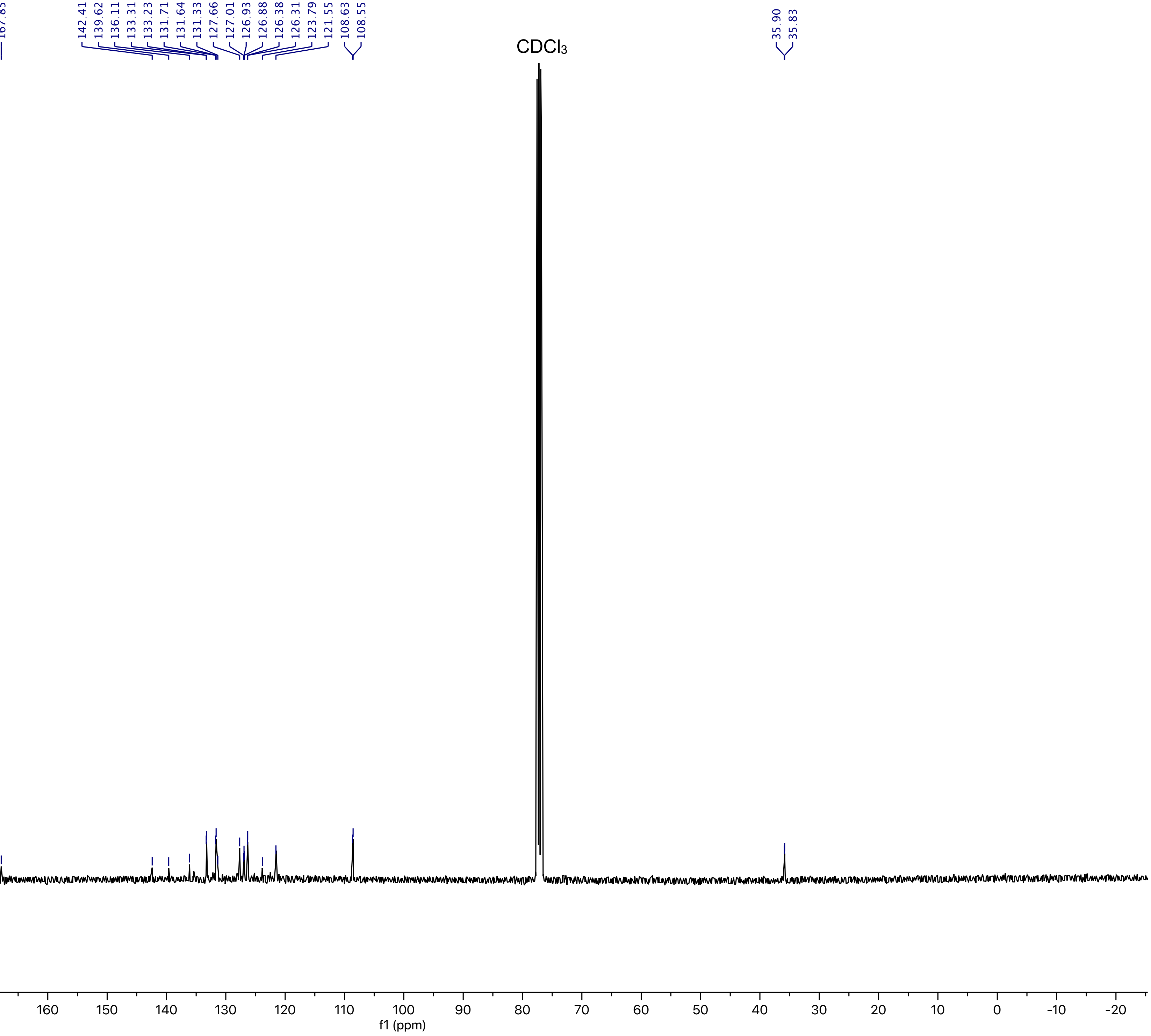

^{19}F NMR: 376 MHz, $CDCl_3$

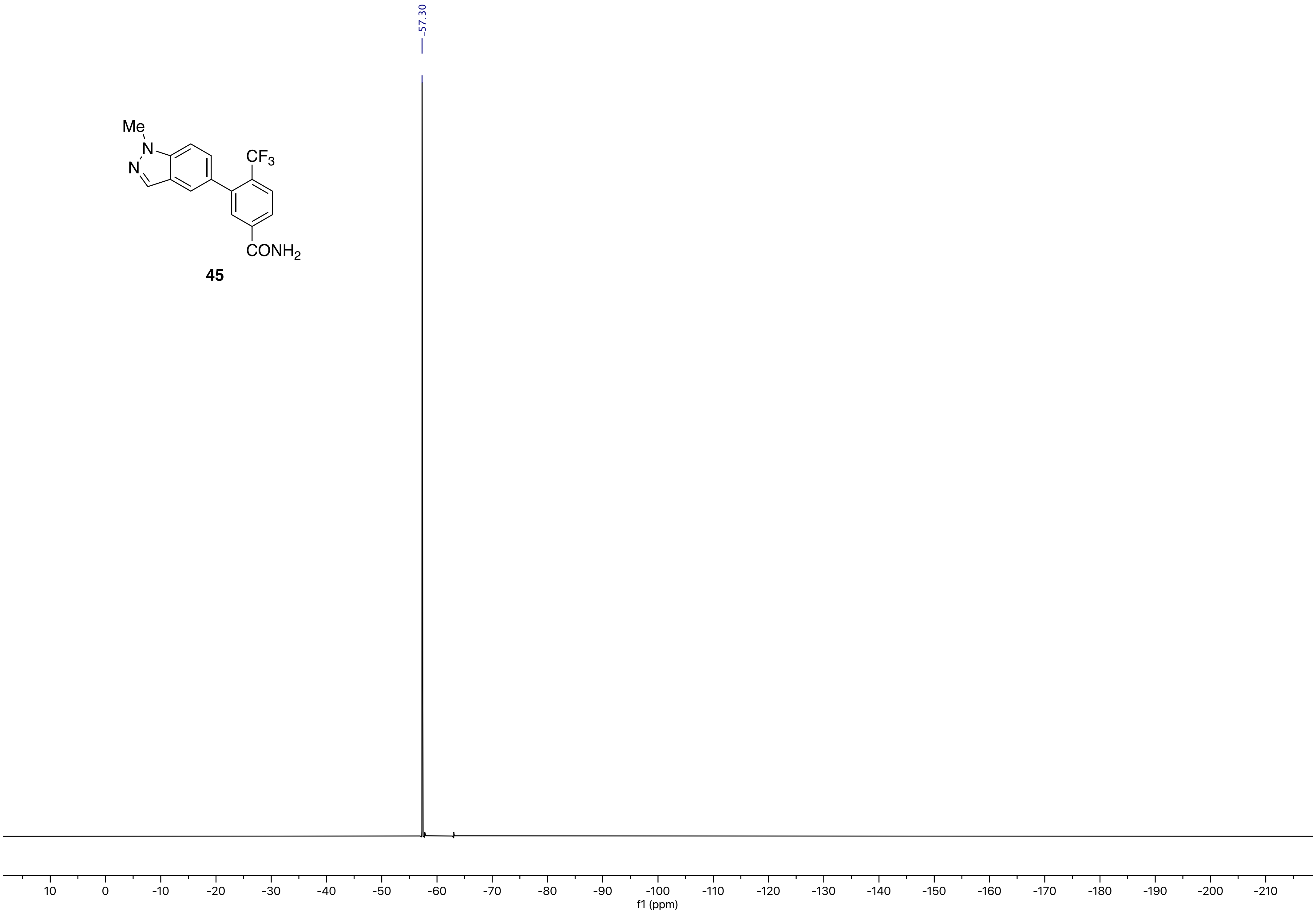

^{1}H NMR: 500 MHz, DMSO-d_6

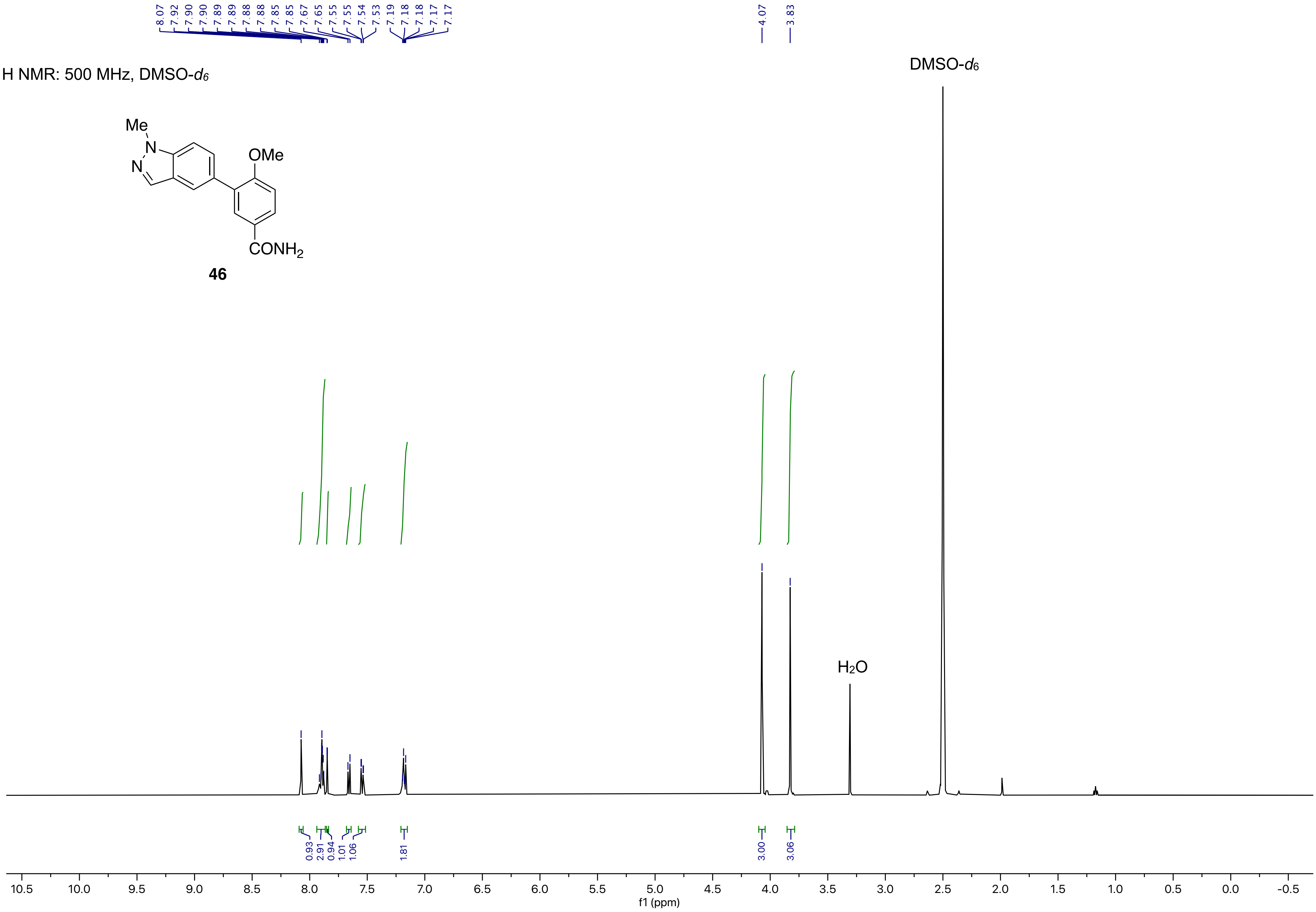

$^{13}C\{^1H\}$ NMR: 101 MHz, DMSO-d_6

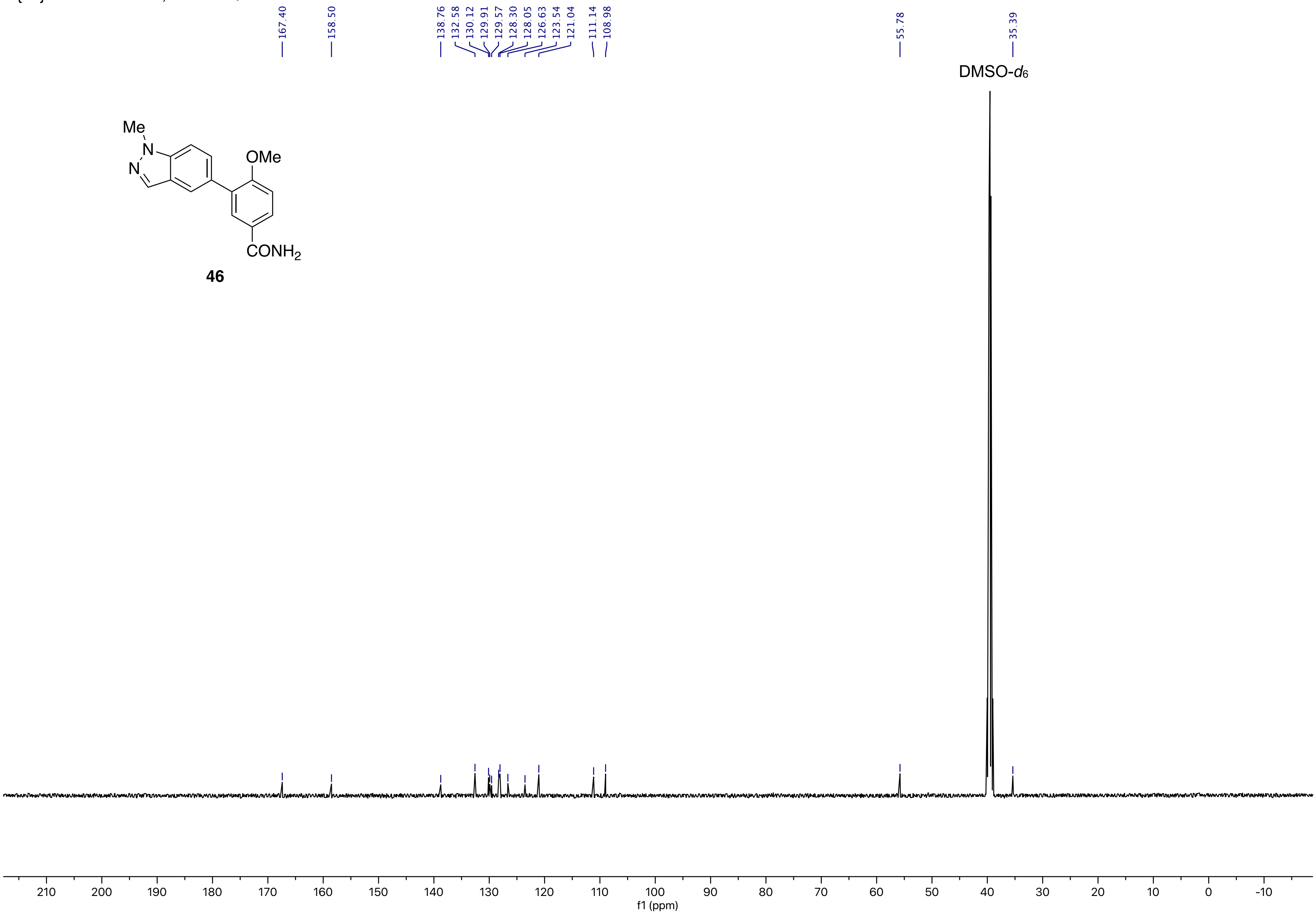

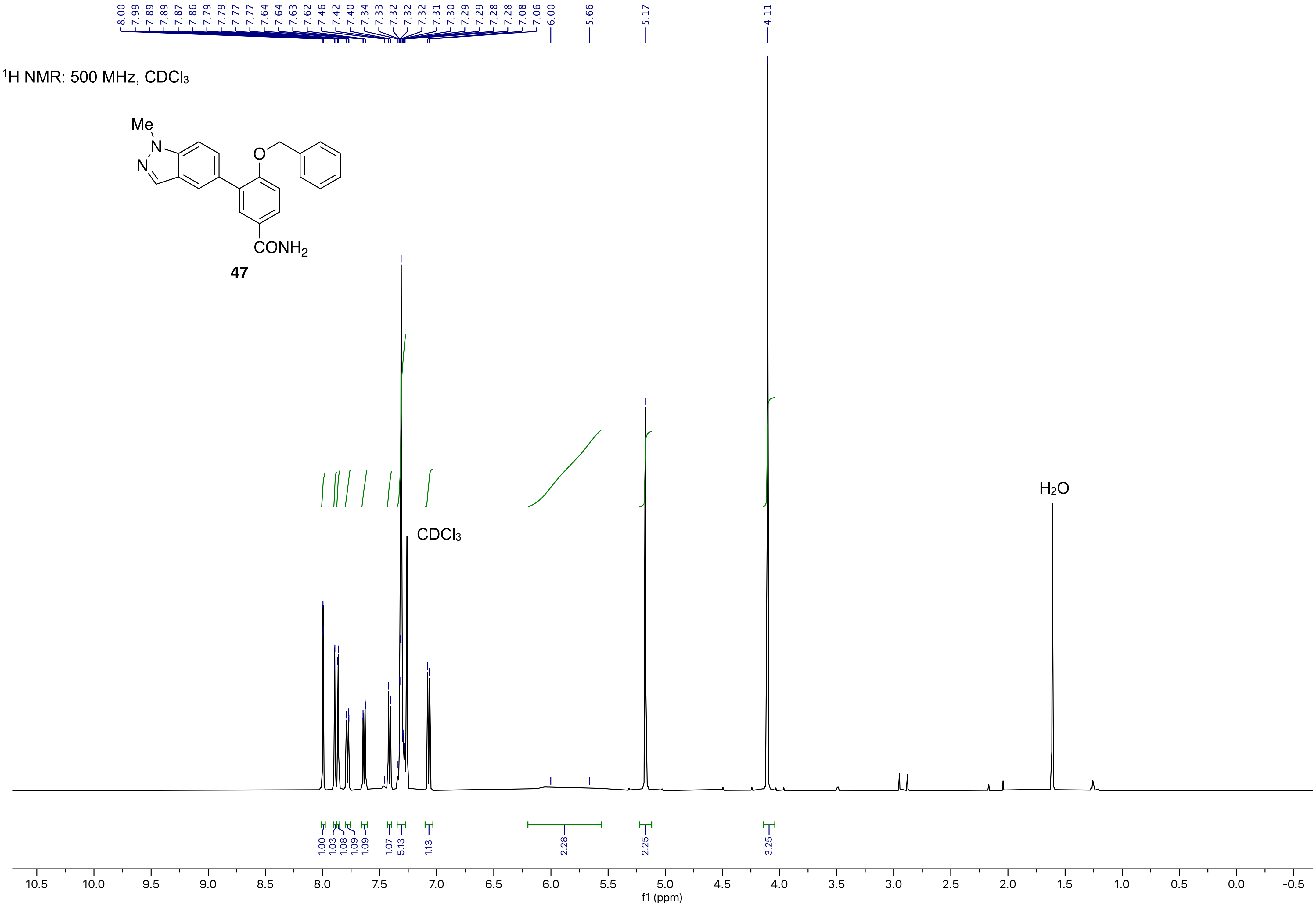

8.00 7.99 7.89 7.89 7.87 7.86 7.79 7.79 7.77 7.77 7.64 7.64 7.63 7.62 7.46 7.42 7.40 7.34 7.33 7.32 7.32 7.32 7.31 7.30 7.29 7.29 7.28 7.28 7.08 7.06 6.00 5.66 5.17 4.11
^{1}H NMR: 500 MHz, $CDCl_3$
Me
N
N
O
$CONH_2$
47
$CDCl_3$
H_2O
1.00 1.03 1.08 1.09 1.09 1.07 5.13 1.13 2.28 2.25 3.25
10.5 10.0 9.5 9.0 8.5 8.0 7.5 7.0 6.5 6.0 5.5 5.0 4.5 4.0 3.5 3.0 2.5 2.0 1.5 1.0 0.5 0.0 -0.5
f1 (ppm)

$^{13}C\{^{1}H\}$ NMR: 101 MHz, DMSO-d_6

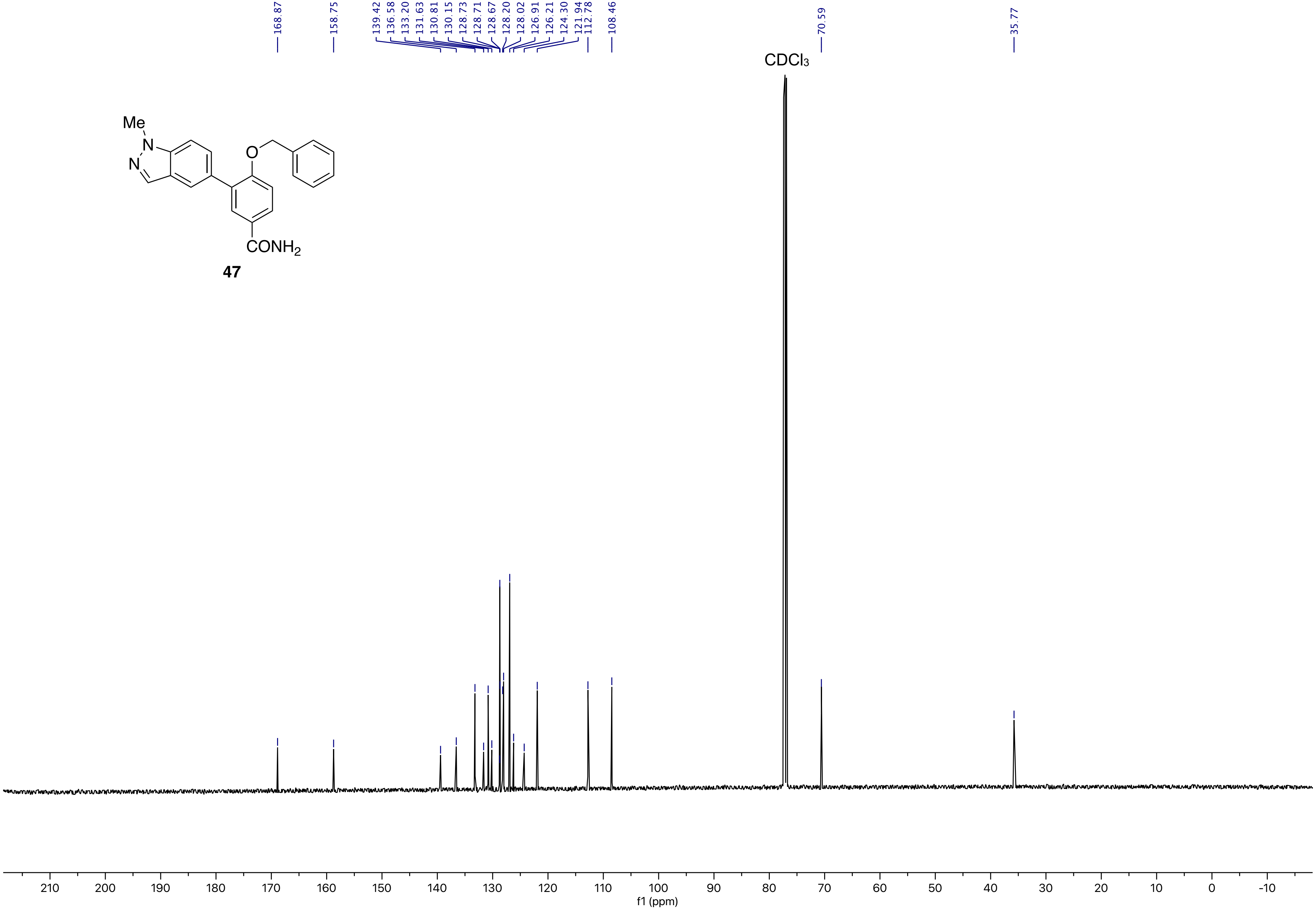

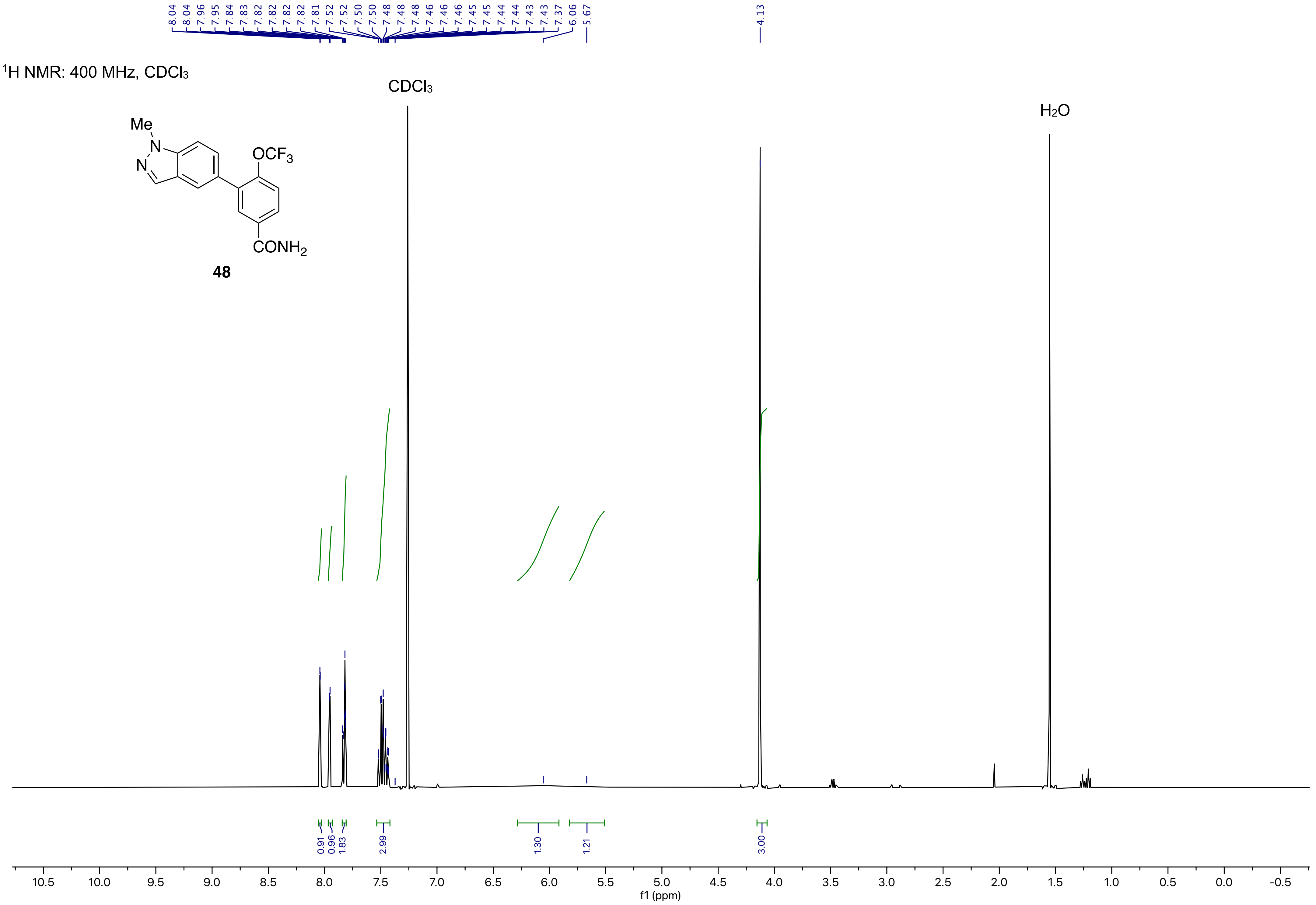
8.04 8.04 7.96 7.95 7.84 7.83 7.82 7.82 7.82 7.82 7.81 7.52 7.52 7.50 7.50 7.48 7.48 7.48 7.46 7.46 7.46 7.45 7.45 7.44 7.44 7.43 7.43 7.37 6.06 5.67
4.13
^{1}H NMR: 400 MHz, $CDCl_3$
$CDCl_3$
H_2O
Me
N
N
OCF_3
$CONH_2$
48
0.91 0.96 1.83 2.99 1.30 1.21 3.00
10.5 10.0 9.5 9.0 8.5 8.0 7.5 7.0 6.5 6.0 5.5 5.0 4.5 4.0 3.5 3.0 2.5 2.0 1.5 1.0 0.5 0.0 -0.5
f1 (ppm)

$^{13}C\{^1H\}$ NMR: 101 MHz, $CDCl_3$

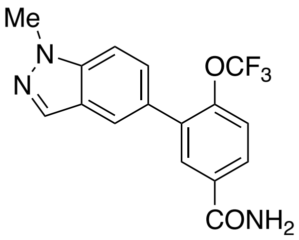

48

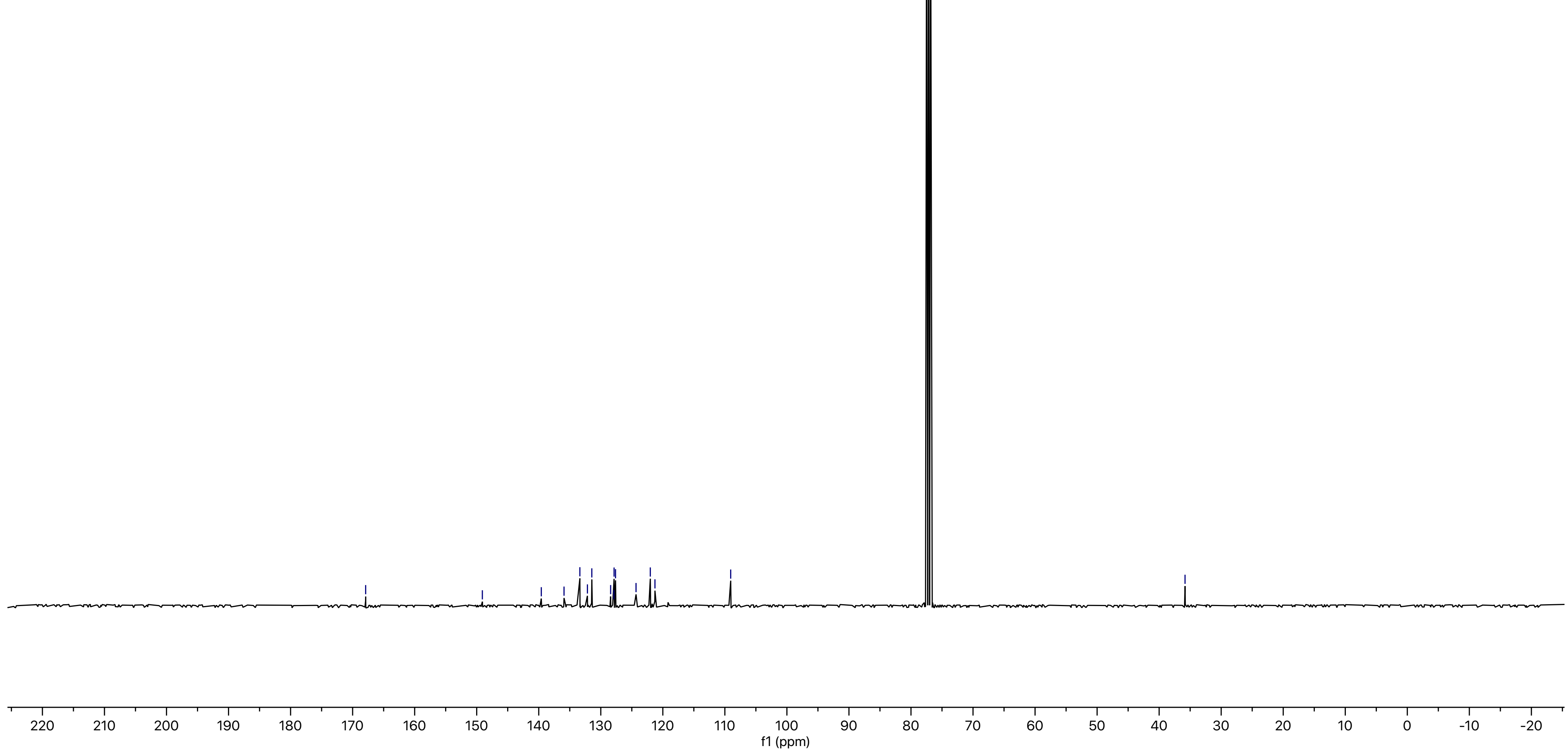

^{19}F NMR: 376 MHz, $CDCl_3$

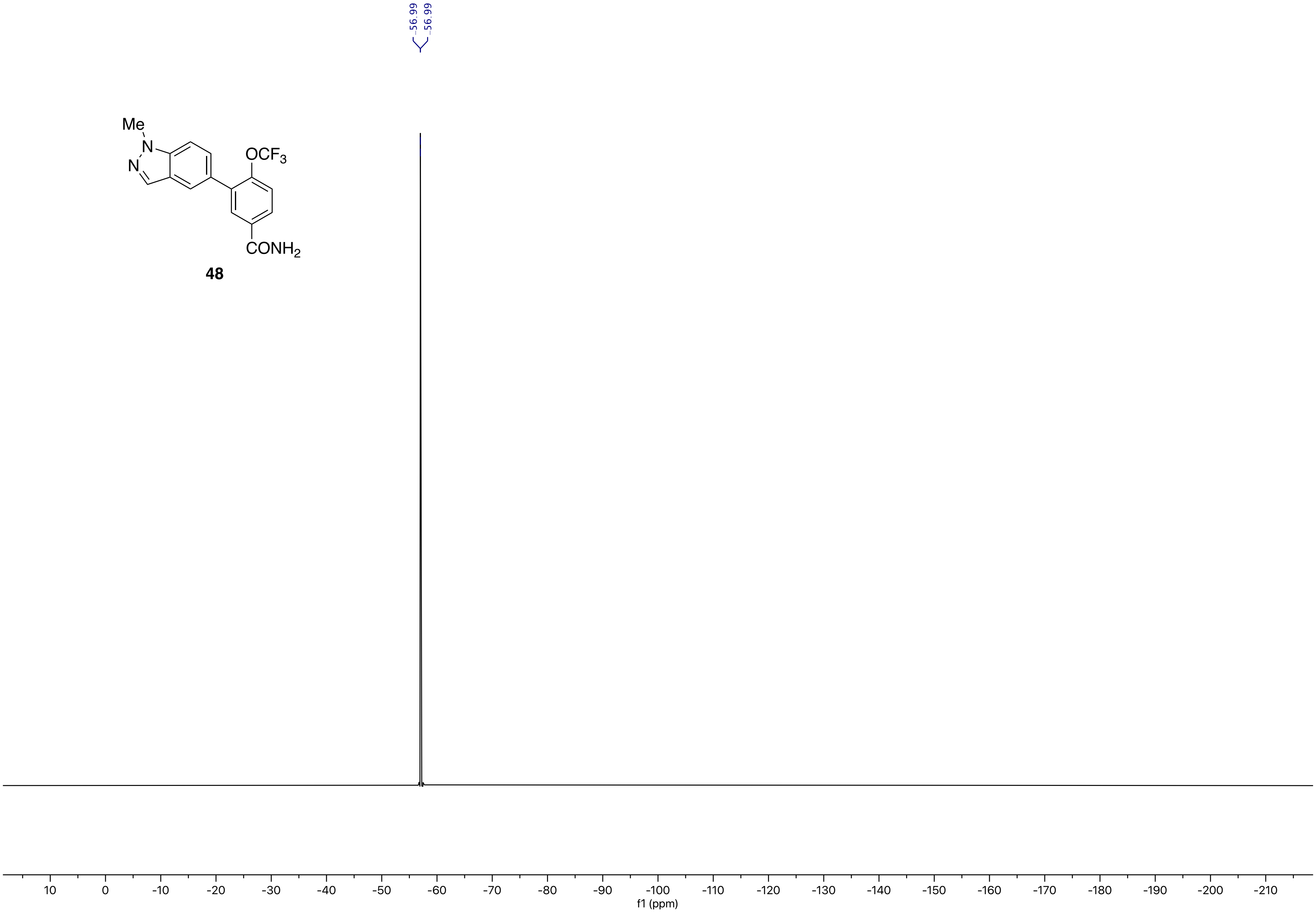

^{1}H NMR: 400 MHz, DMSO-d_6

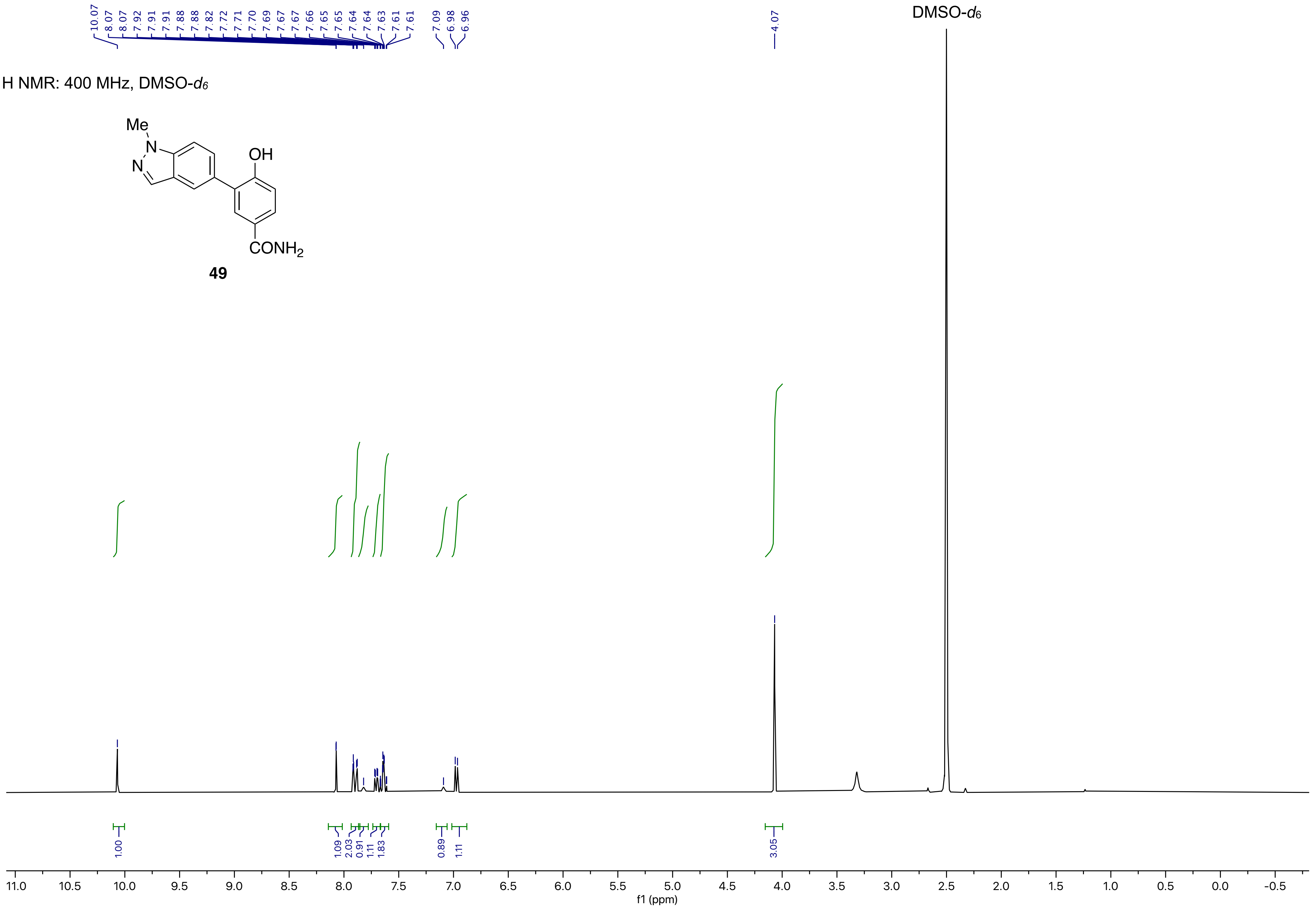

49

$^{13}C\{^{1}H\}$ NMR: 101 MHz, DMSO-d_6

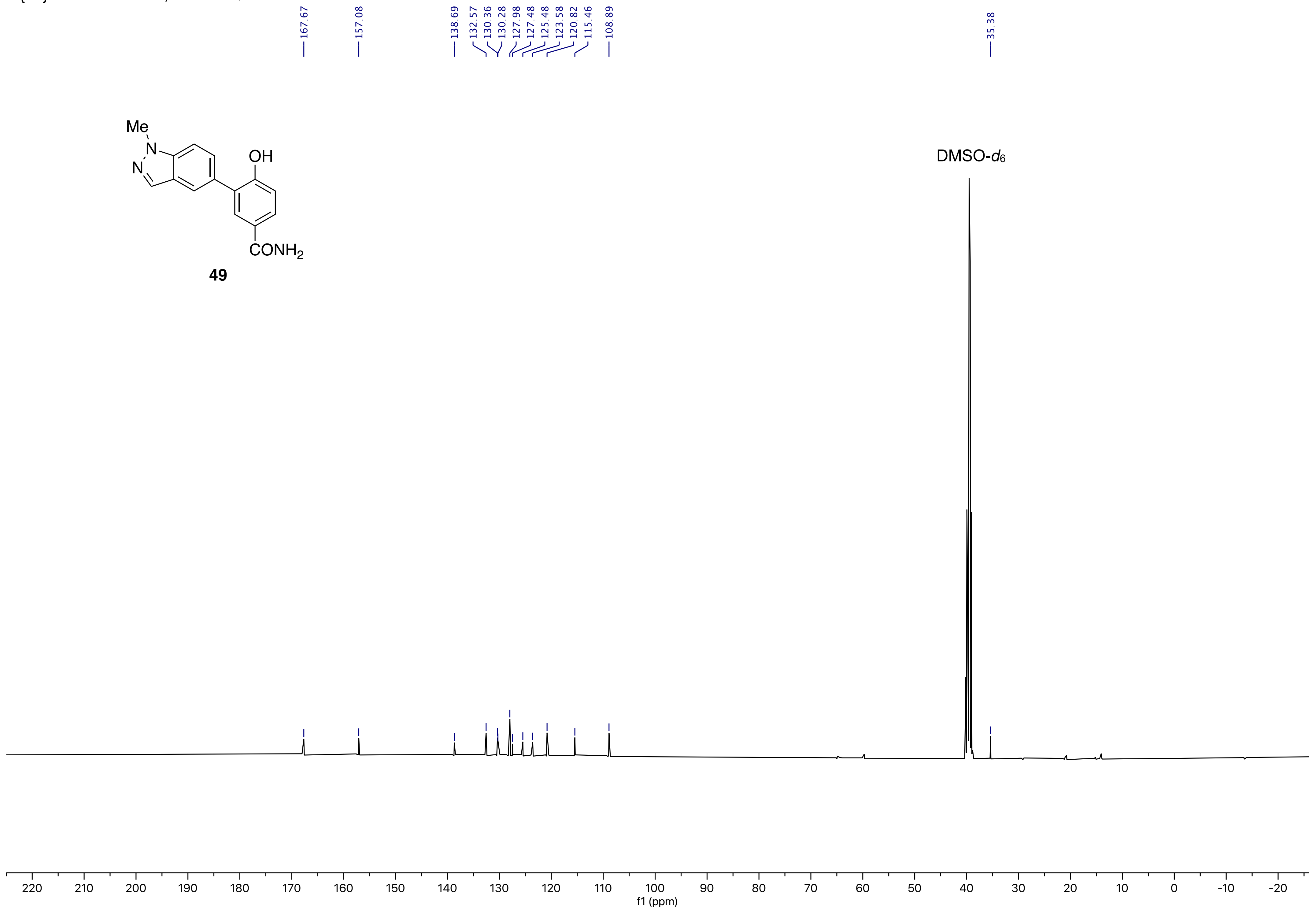

^{1}H NMR: 400 MHz, DMSO-d_6

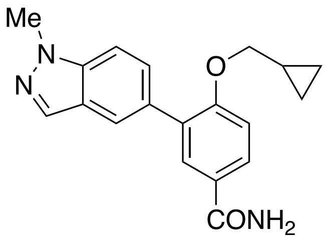

50

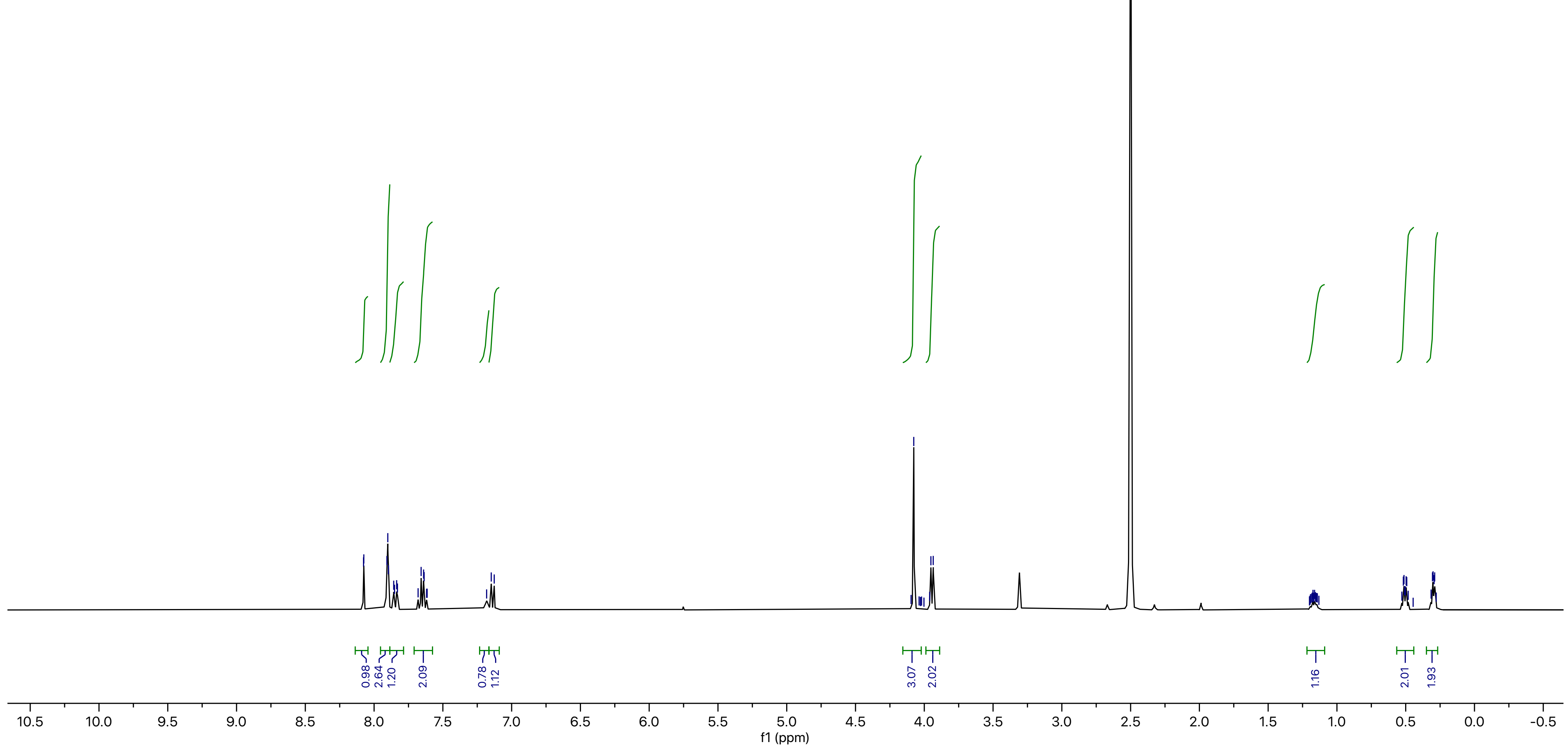

$^{13}C\{^1H\}$ NMR: 101 MHz, DMSO-d_6

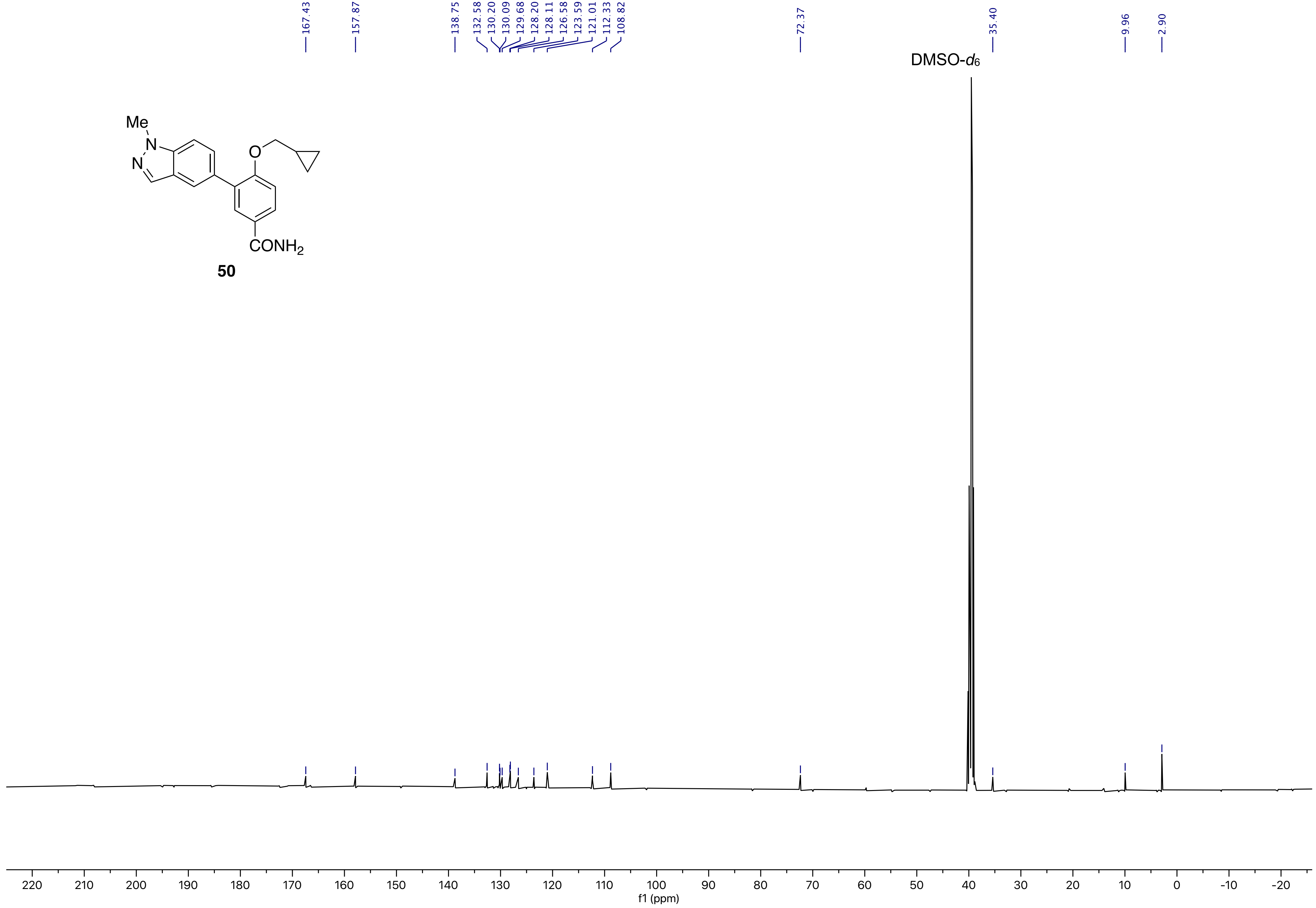

^{1}H NMR: 400 MHz, DMSO-d_6

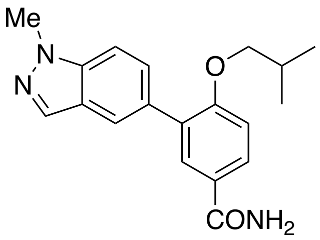

51

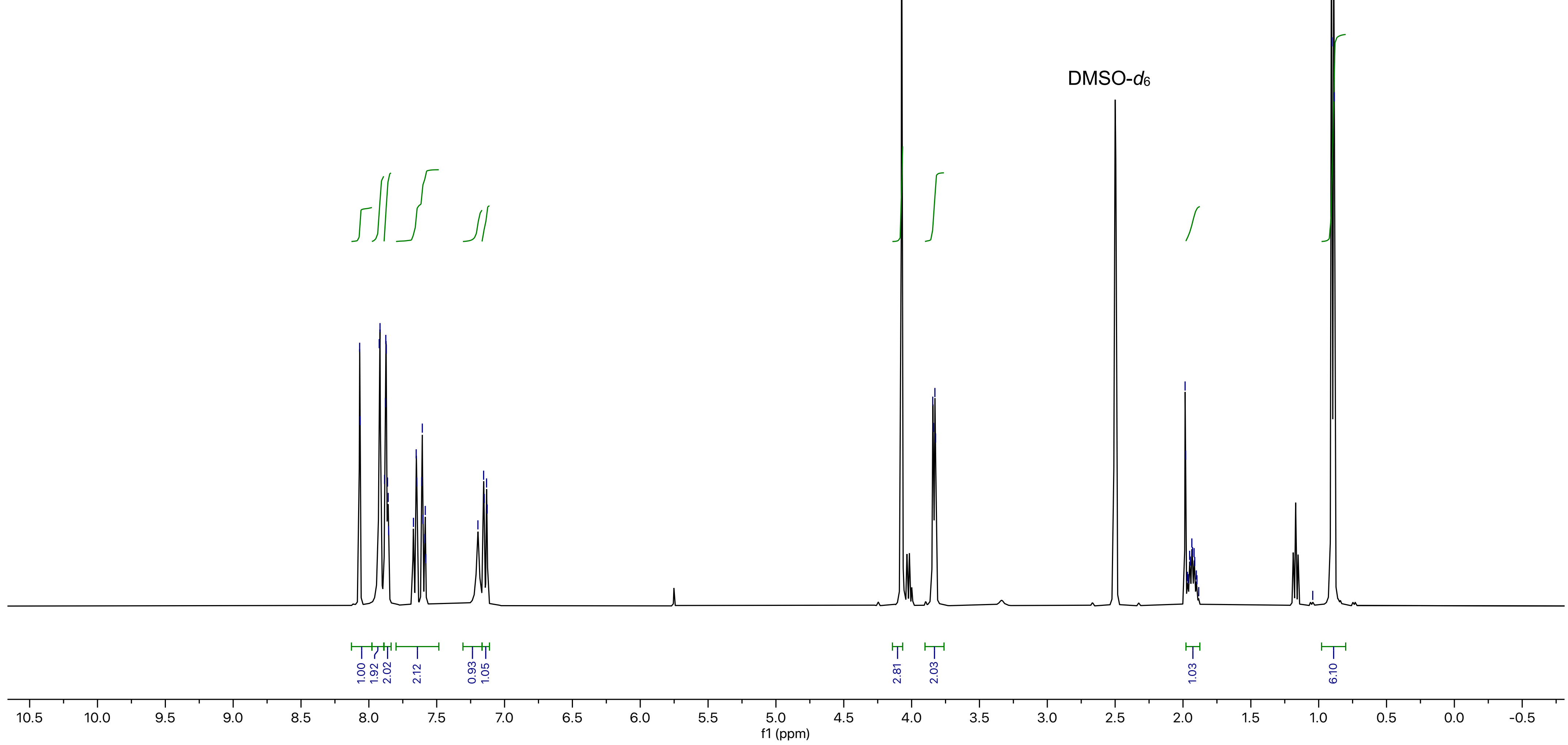

$^{13}C\{^{1}H\}$ NMR: 101 MHz, DMSO-d_6

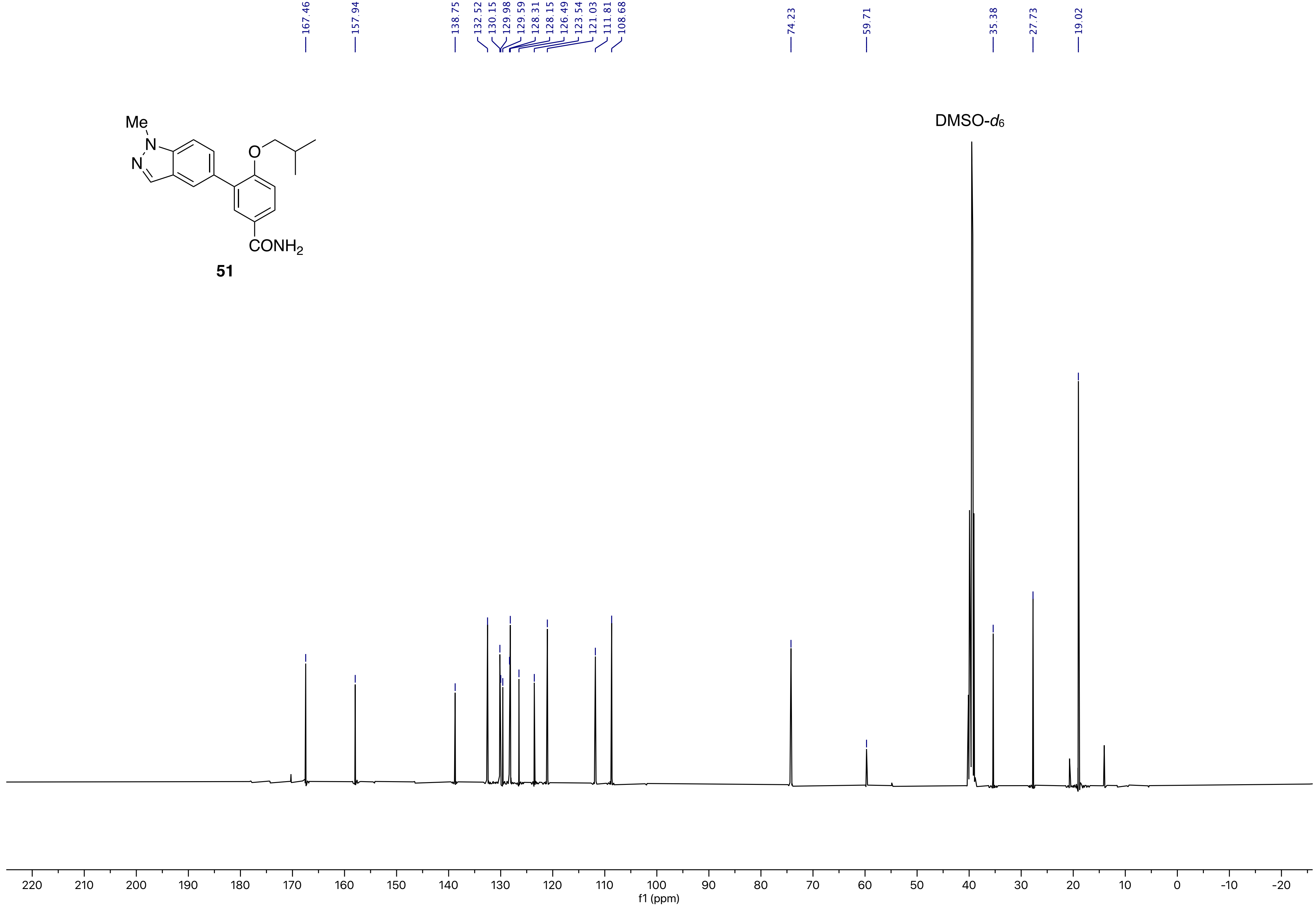

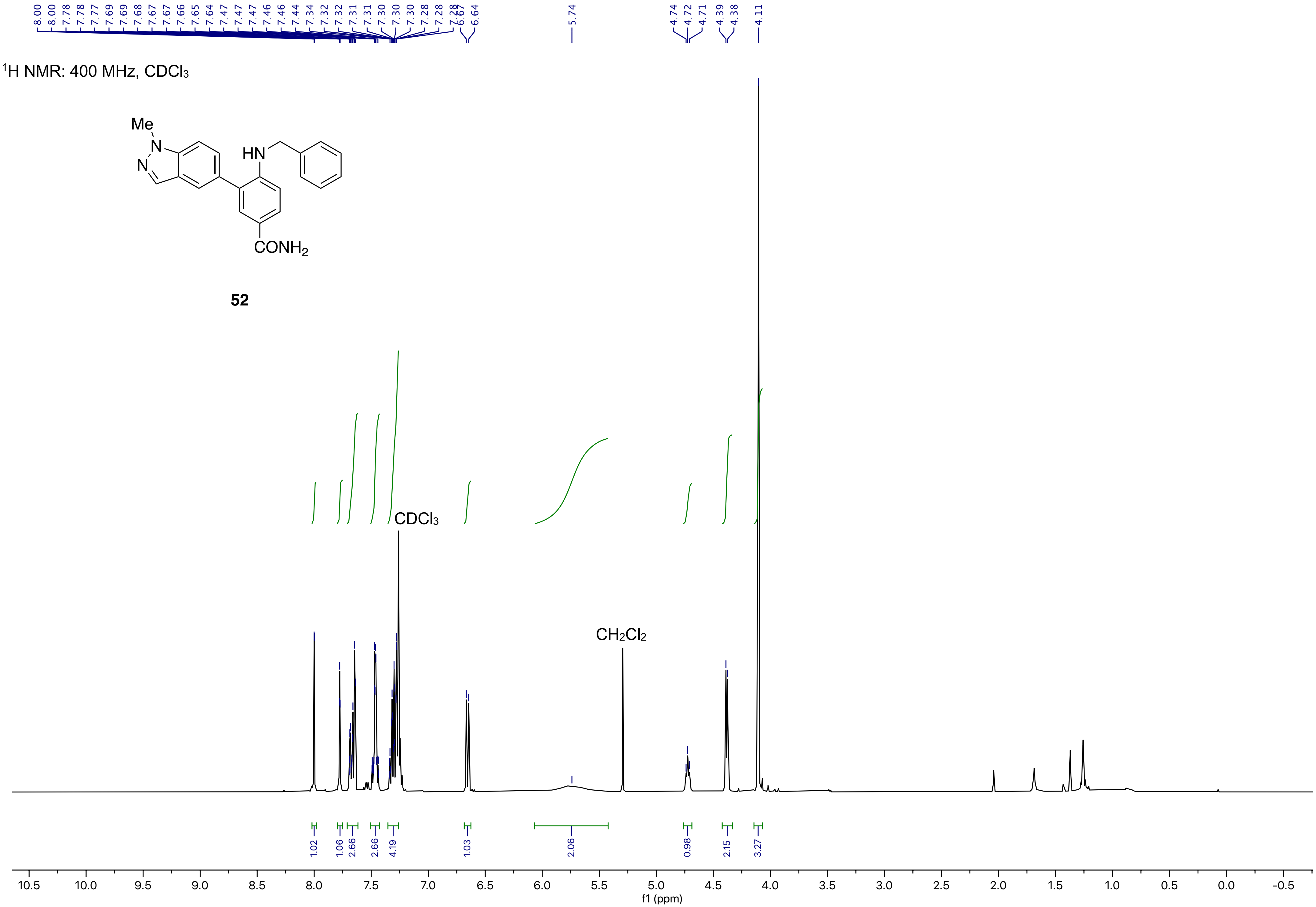

8.00
8.00
7.78
7.78
7.77
7.69
7.69
7.68
7.67
7.67
7.66
7.65
7.64
7.47
7.47
7.47
7.46
7.46
7.44
7.34
7.32
7.32
7.31
7.31
7.30
7.30
7.30
7.28
7.28
7.28
6.67
6.64
5.74
4.74
4.72
4.71
4.39
4.38
4.11
1H NMR: 400 MHz, CDCl3
Me
N
N
HN
CONH2
52
CDCl3
CH2Cl2
1.02
1.06
2.66
2.66
4.19
1.03
2.06
0.98
2.15
3.27
10.5
10.0
9.5
9.0
8.5
8.0
7.5
7.0
6.5
6.0
5.5
5.0
4.5
4.0
3.5
3.0
2.5
2.0
1.5
1.0
0.5
0.0
-0.5
f1 (ppm)

$^{13}C\{^1H\}$ NMR: 101 MHz, $CDCl_3$

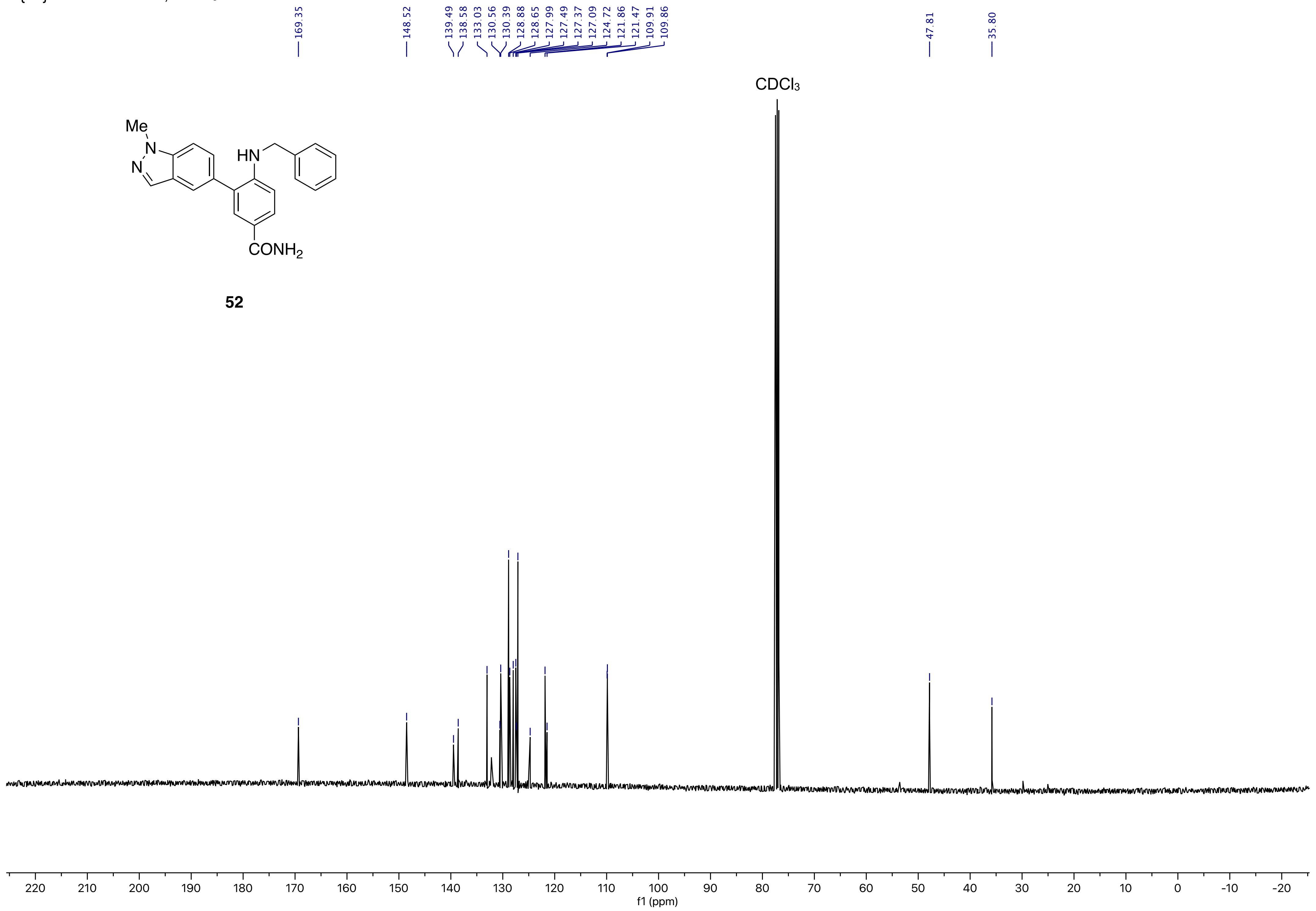

^{1}H NMR: 500 MHz, $CDCl_3$

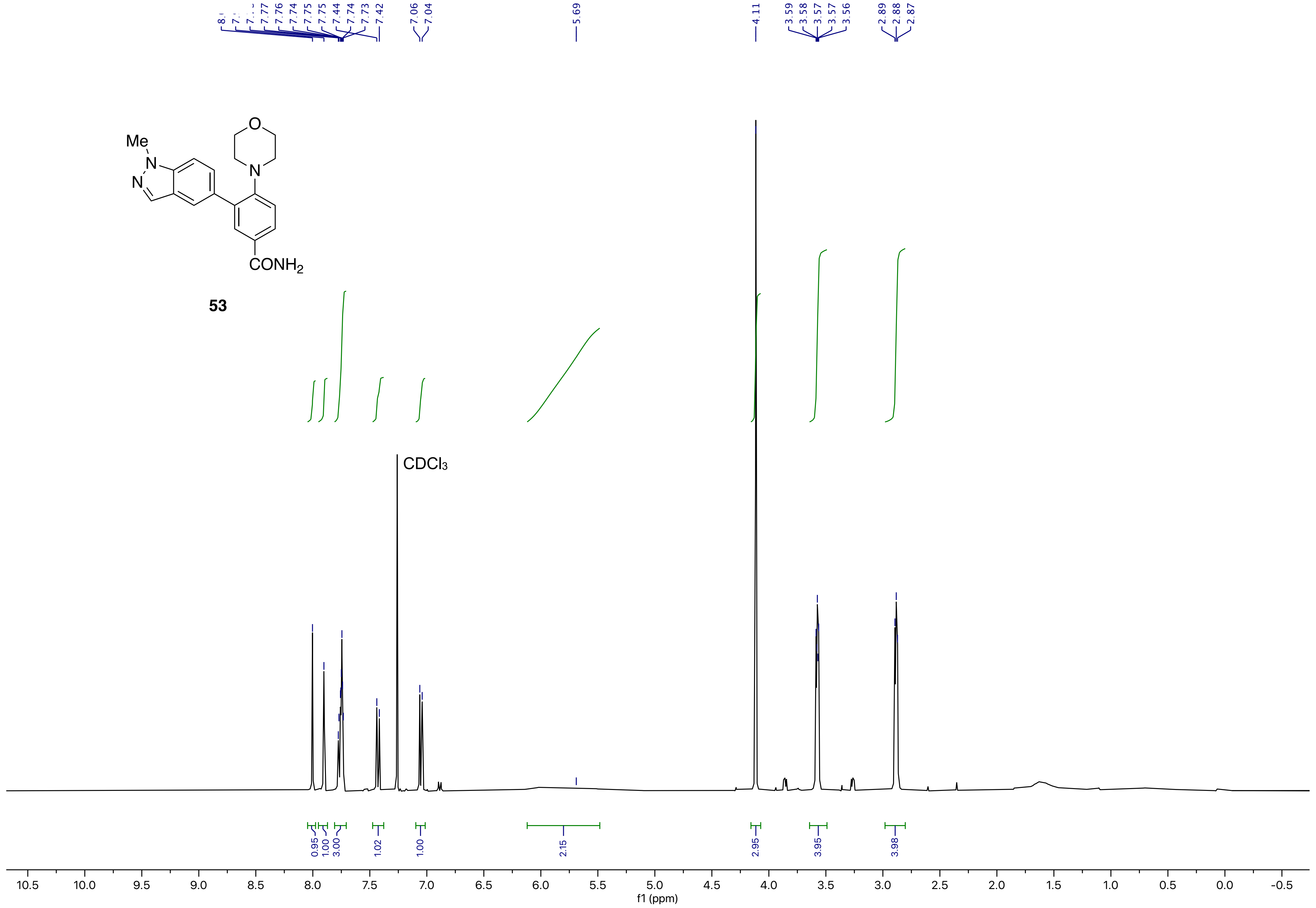

$^{13}C\{^1H\}$ NMR: 126 MHz, $CDCl_3$

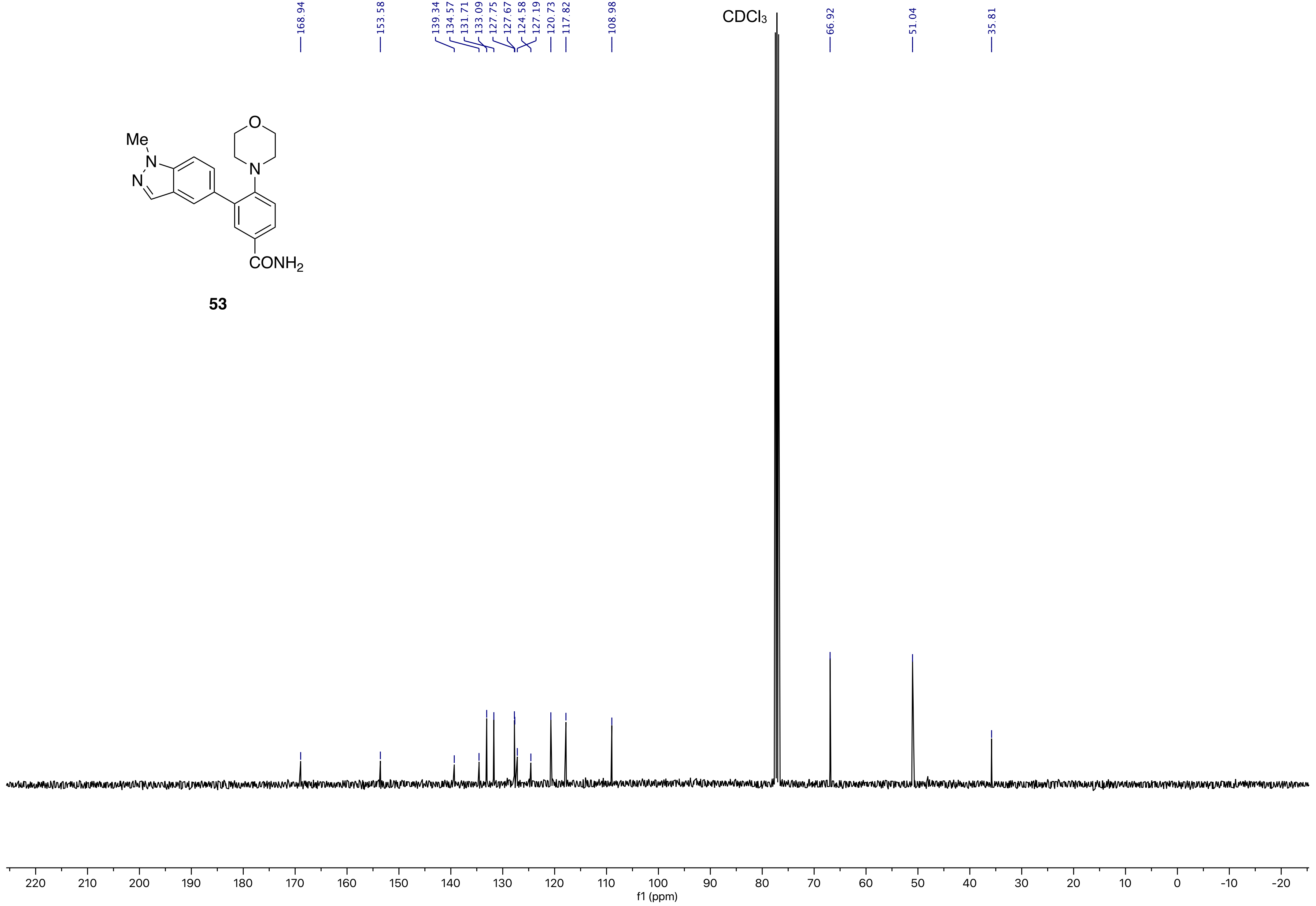

^{1}H NMR: 500 MHz, DMSO-d_6

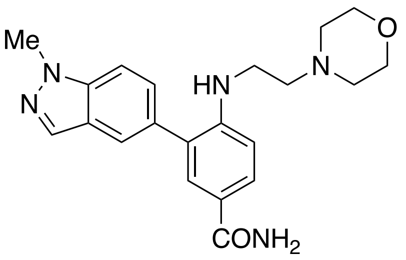

54

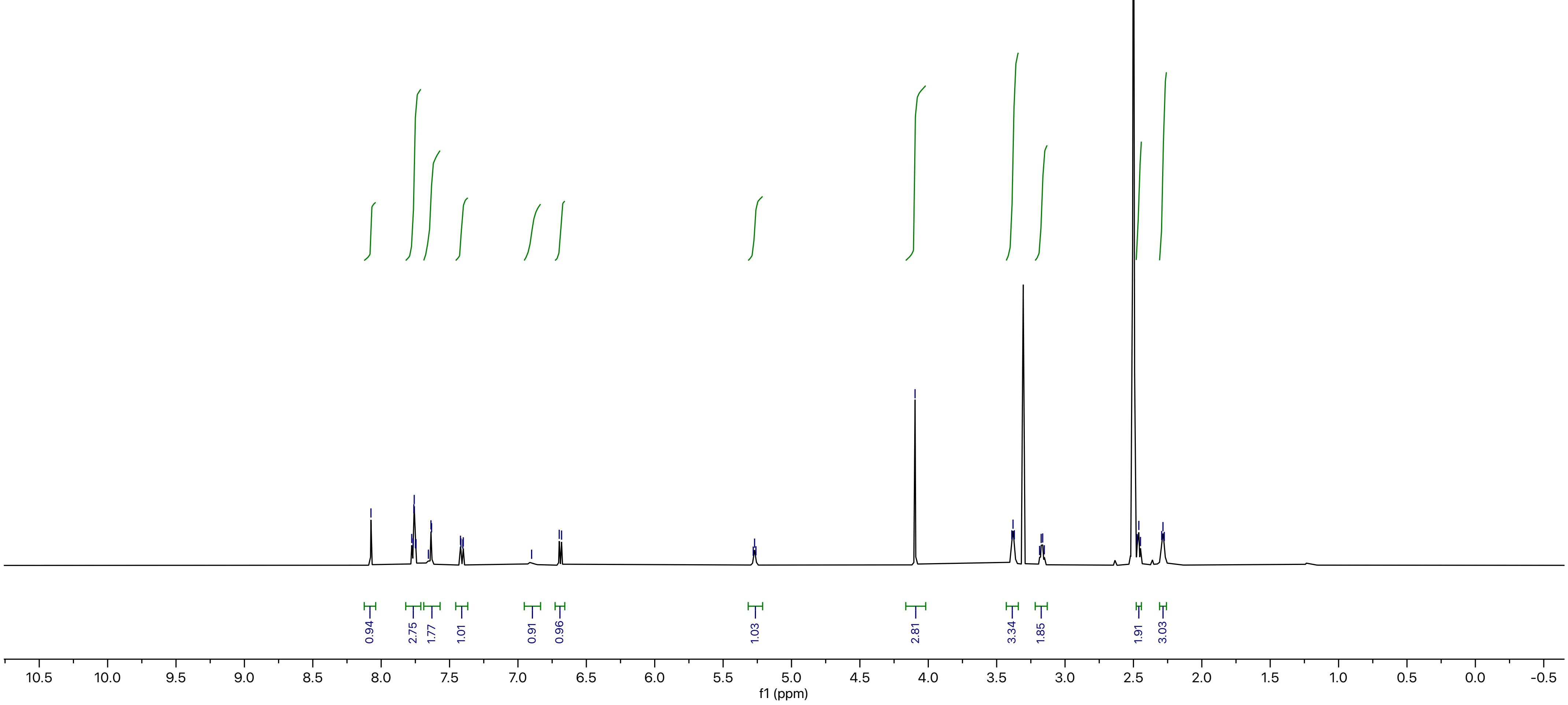

$^{13}C\{^1H\}$ NMR: 126 MHz, DMSO-d_6

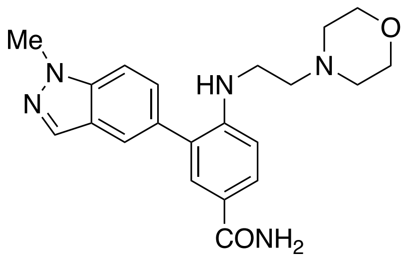

54

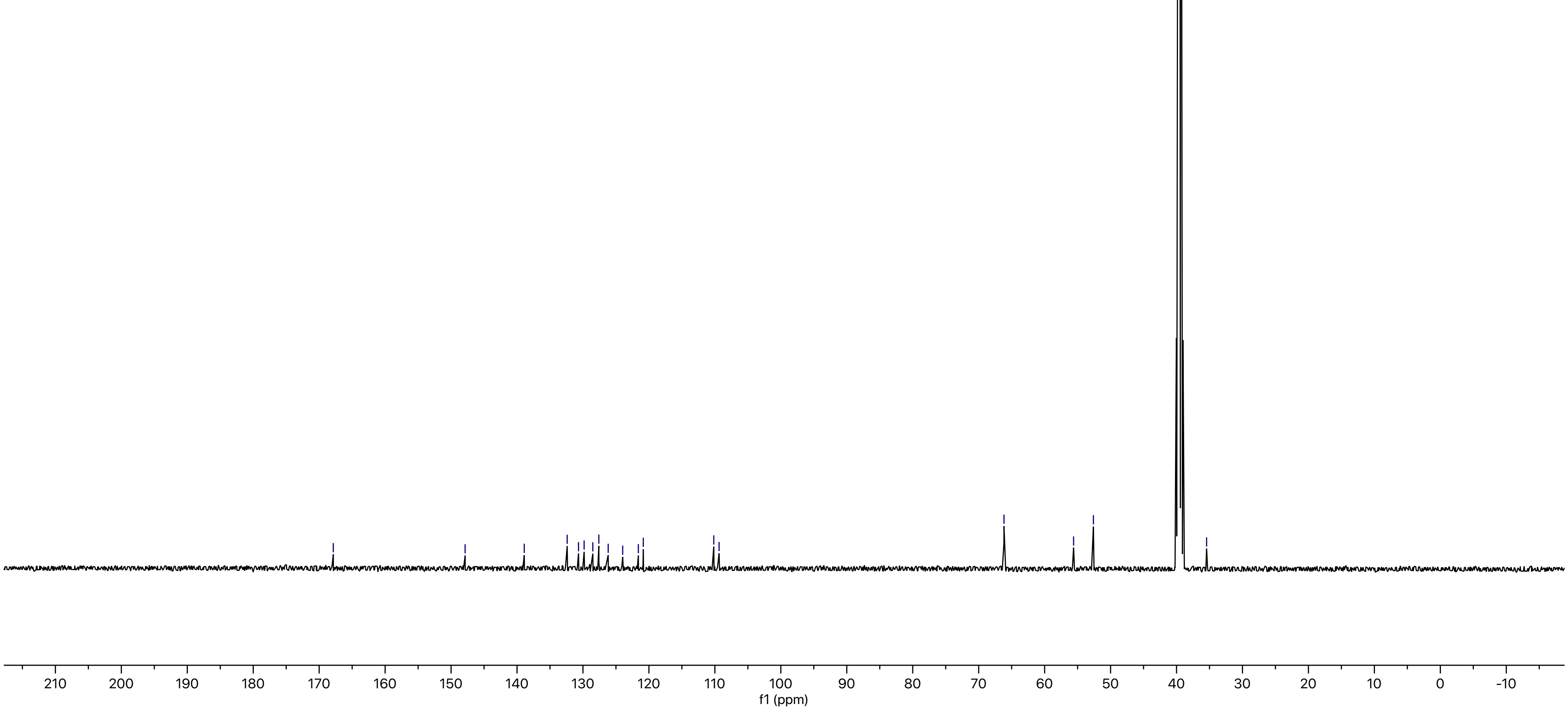

^{1}H NMR: 500 MHz, DMSO-d_6

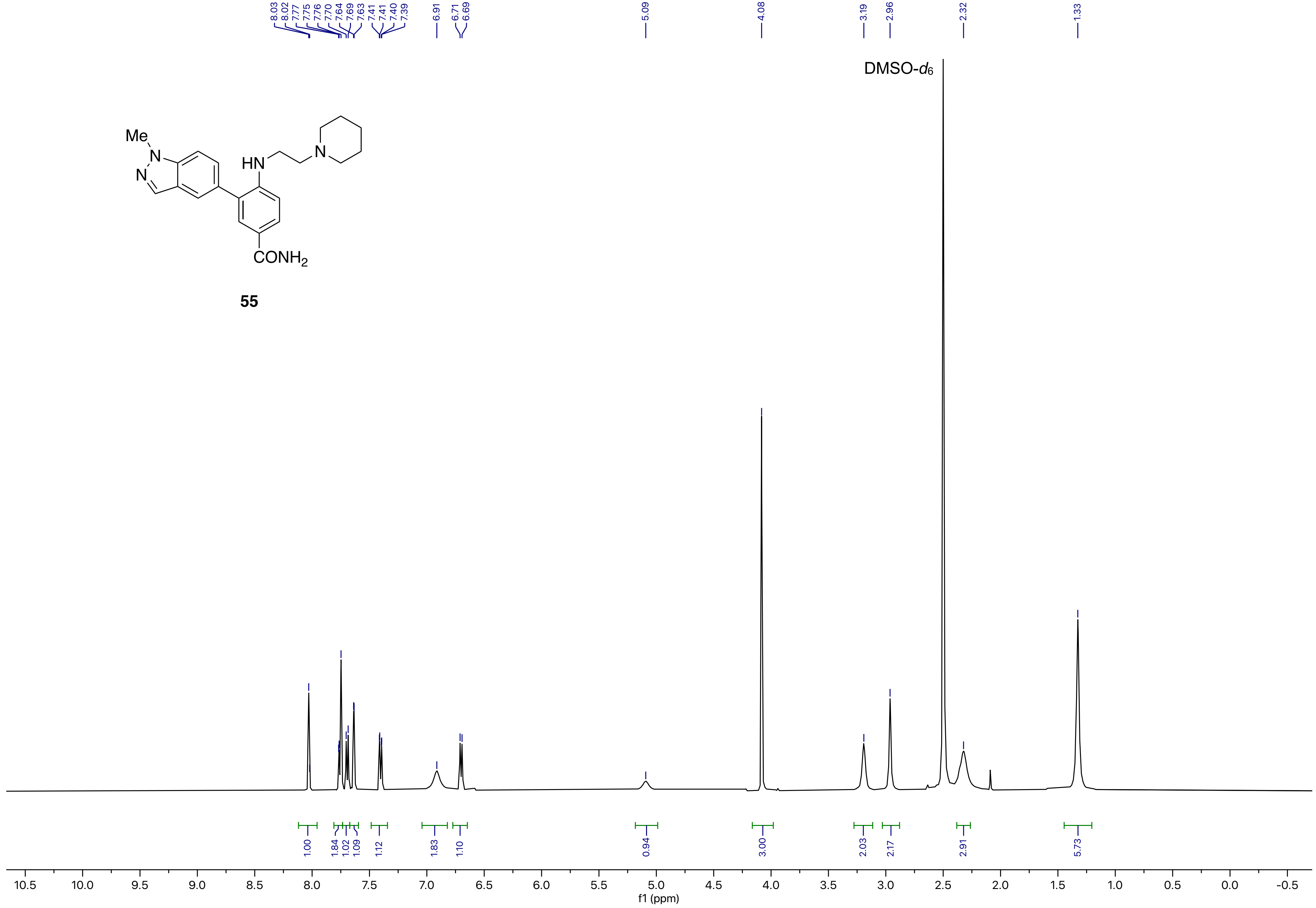

$^{13}C\{^1H\}$ NMR: 126 MHz, DMSO-d_6

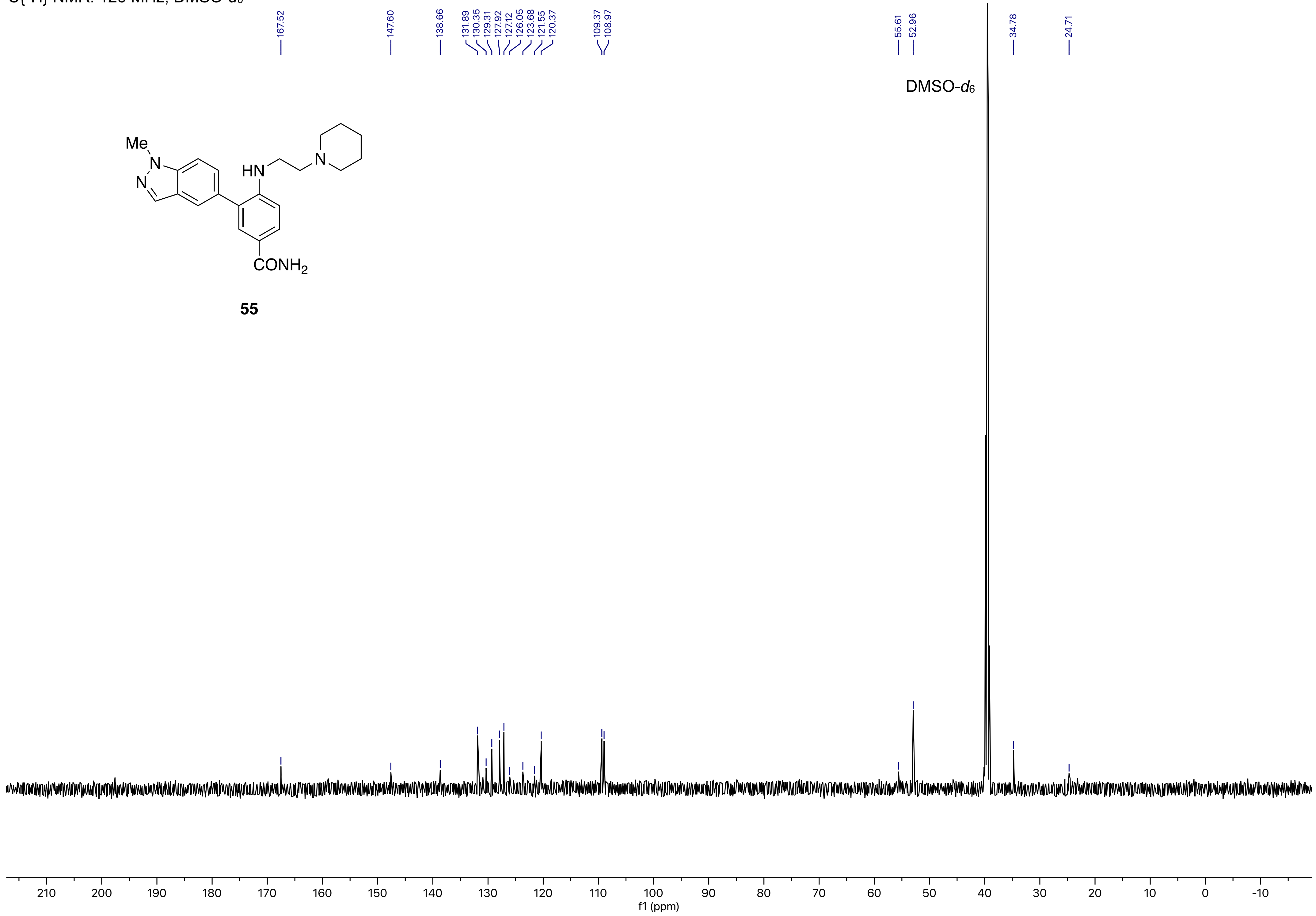

^1H NMR: 500 MHz, $CDCl_3$

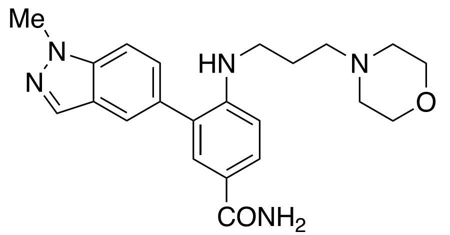

56

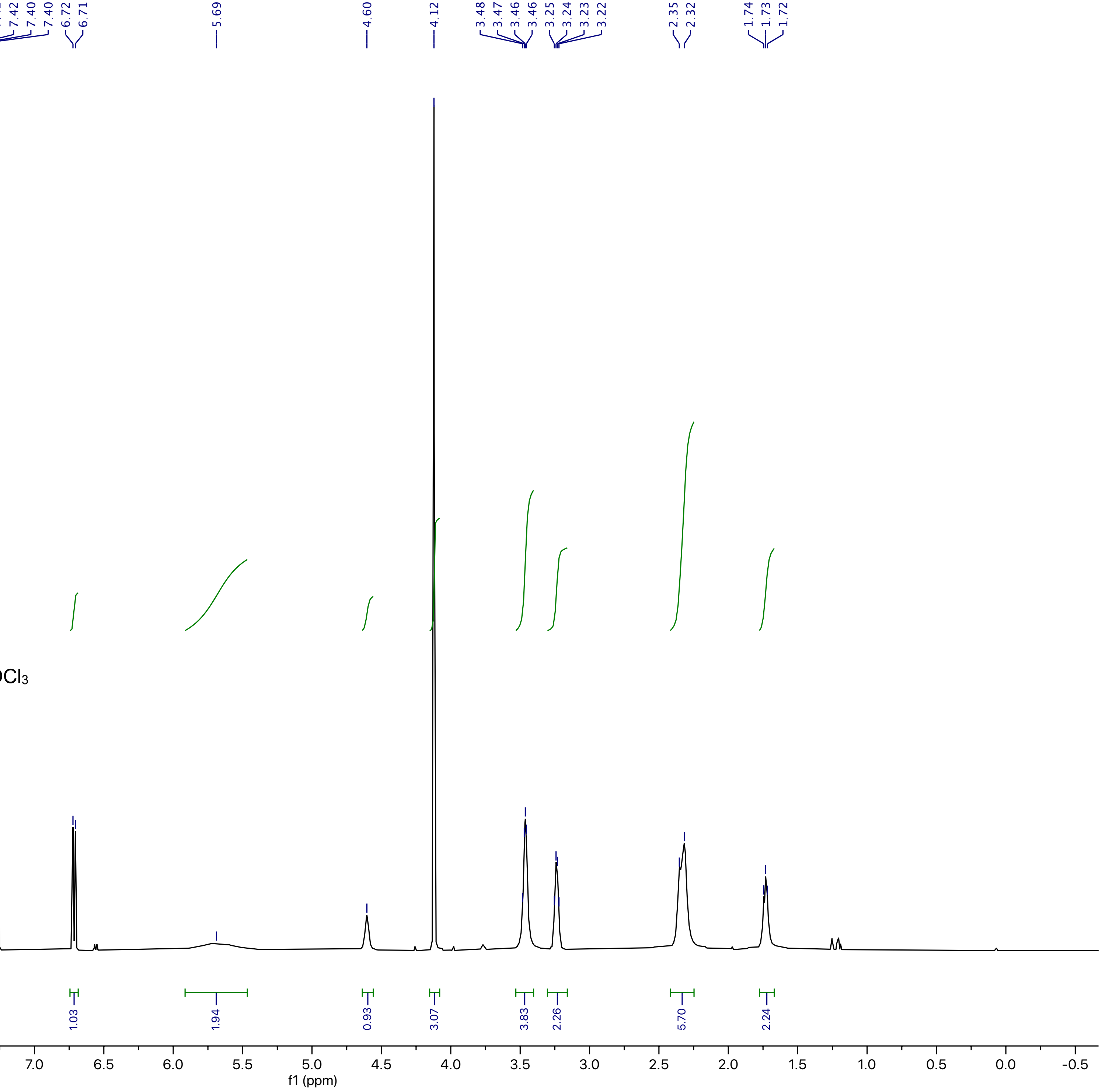

$^{13}C\{^1H\}$ NMR: 126 MHz, $CDCl_3$

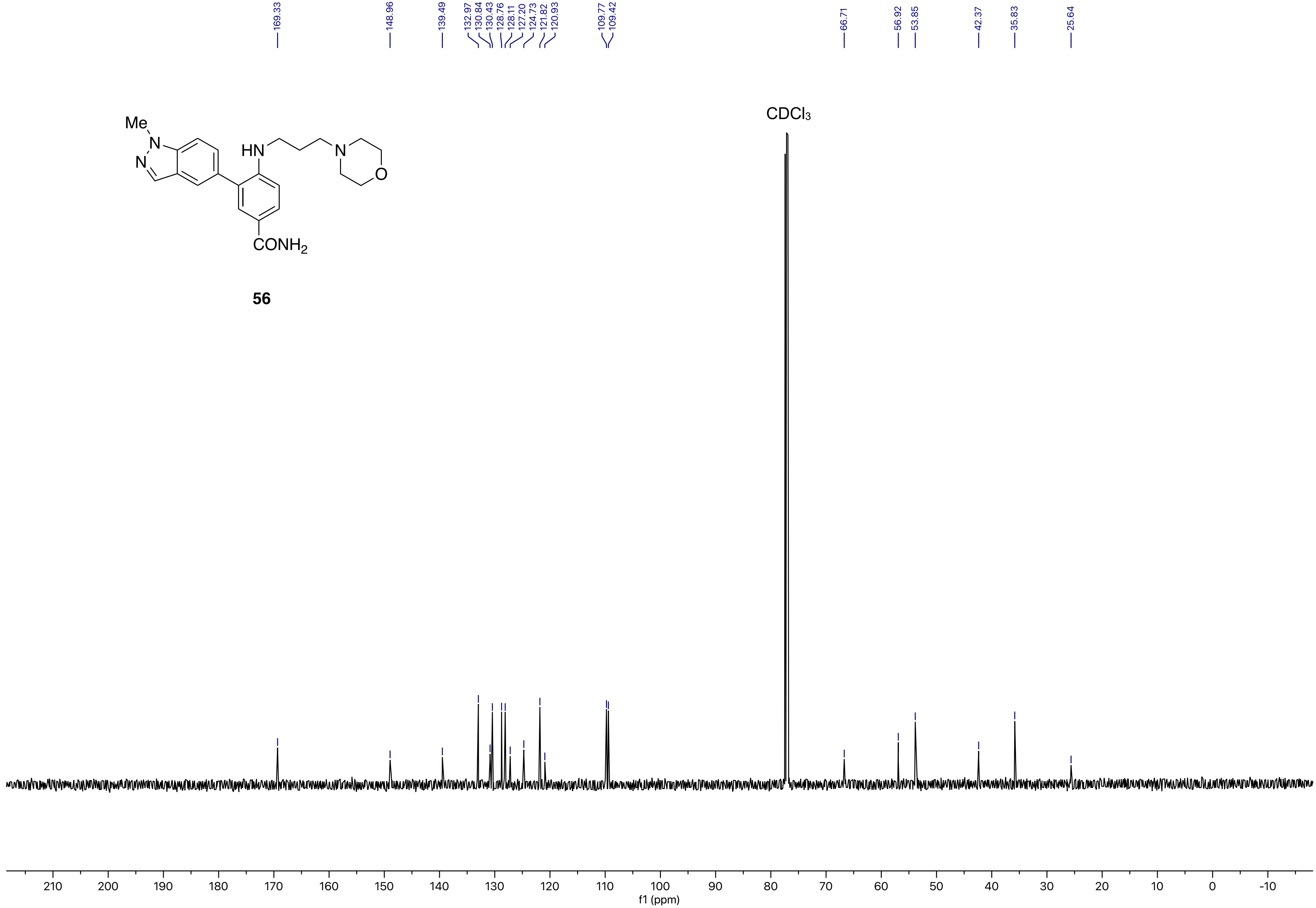

^{1}H NMR: 400 MHz, $CDCl_3$

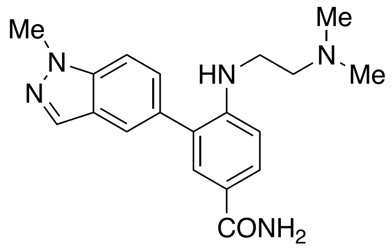

57

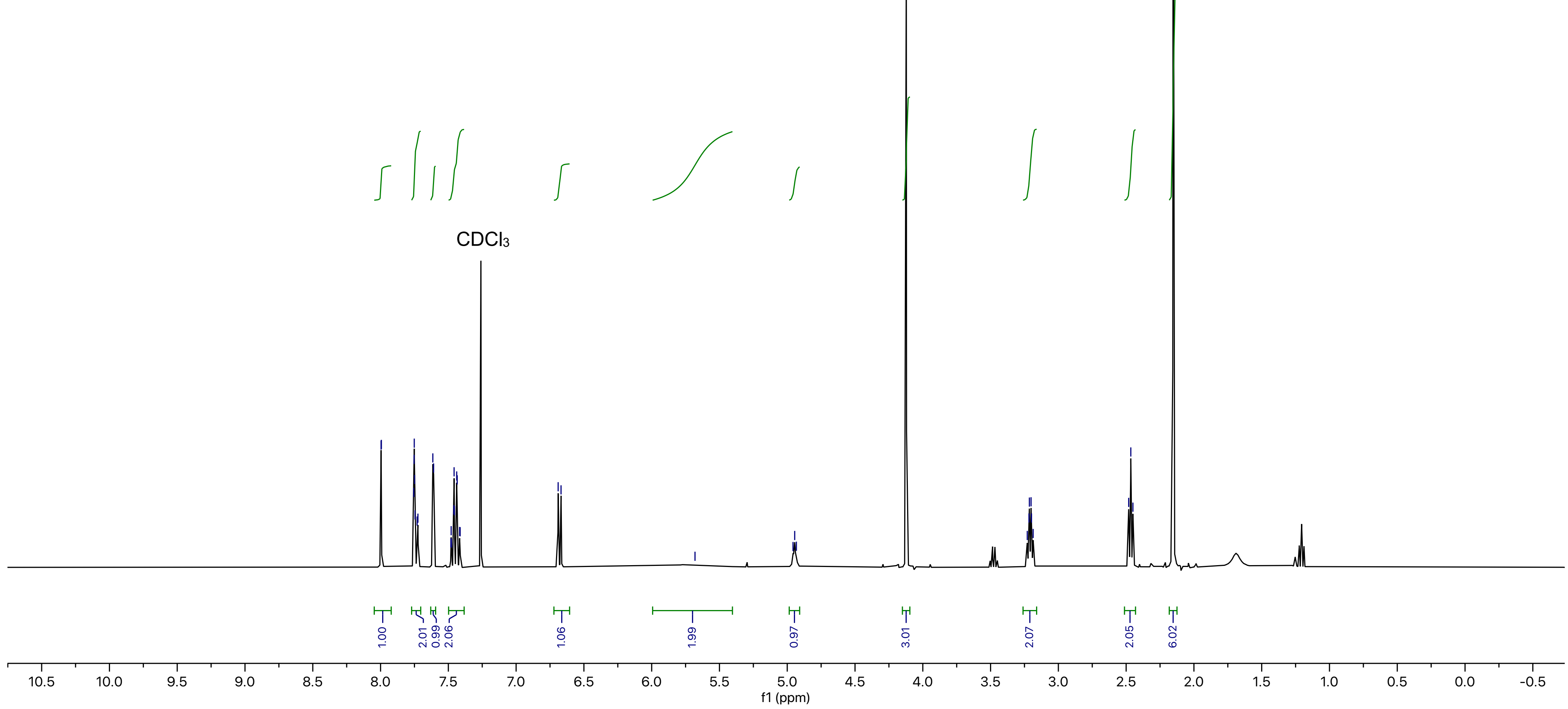

$^{13}C\{^1H\}$ NMR: 101 MHz, $CDCl_3$

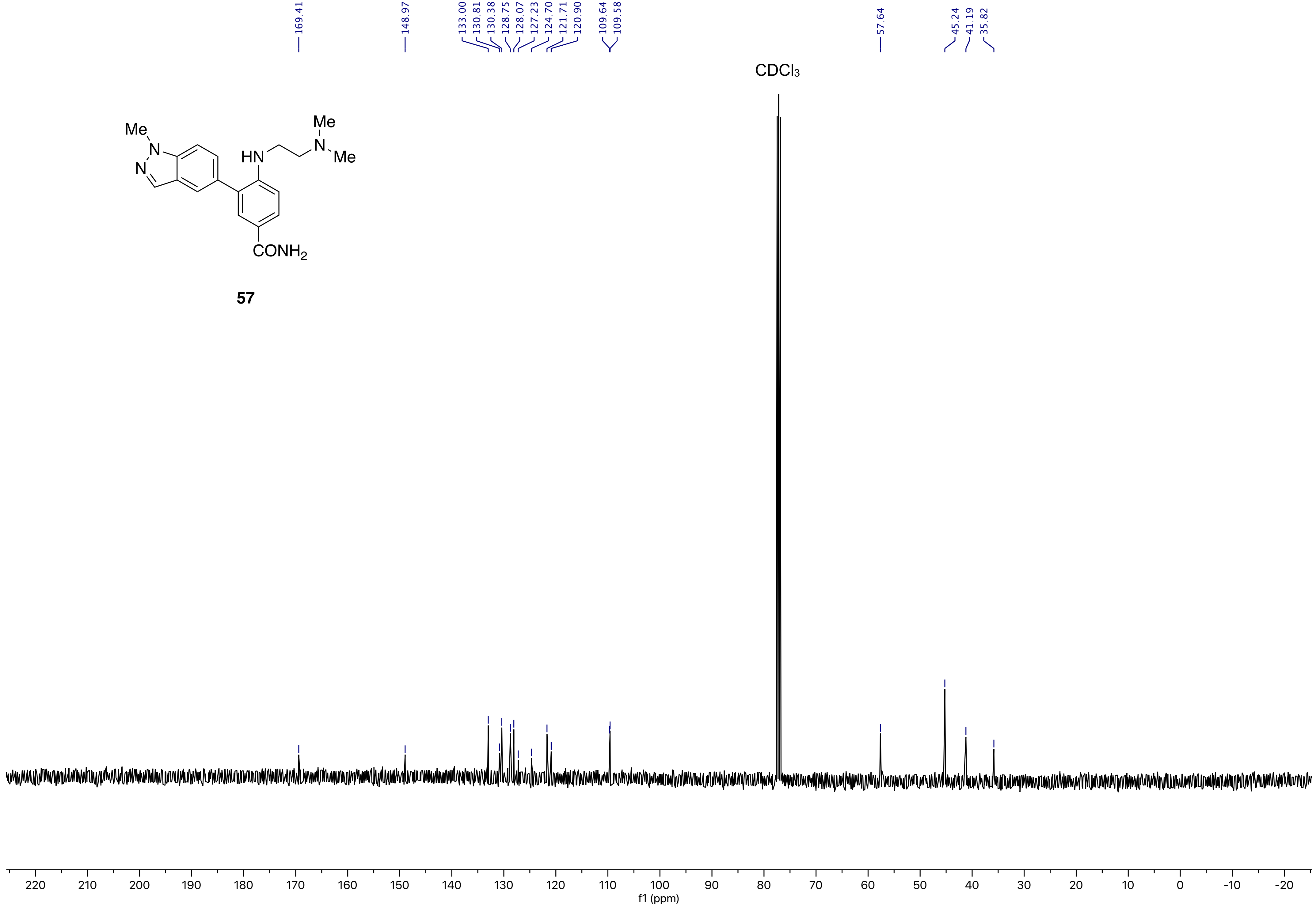

57

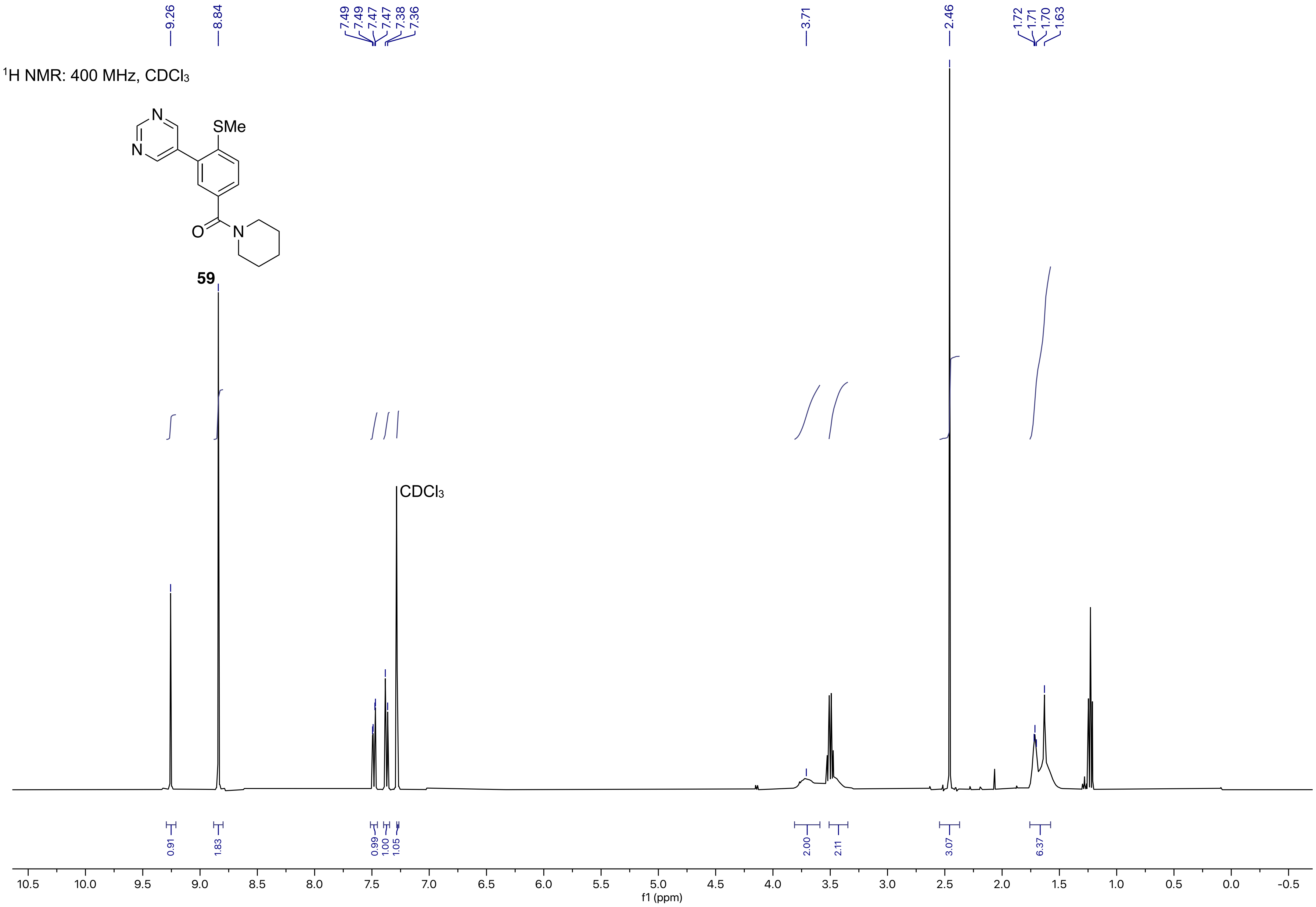

^{1}H NMR: 400 MHz, CDCl3
SMe
N
N
O
N
59
CDCl3
9.26
8.84
7.49
7.49
7.47
7.47
7.38
7.36
3.71
2.46
1.72
1.71
1.70
1.63
0.91
1.83
0.99
1.00
1.05
2.00
2.11
3.07
6.37
10.5 10.0 9.5 9.0 8.5 8.0 7.5 7.0 6.5 6.0 5.5 5.0 4.5 4.0 3.5 3.0 2.5 2.0 1.5 1.0 0.5 0.0 -0.5
f1 (ppm)

$^{13}C\{^1H\}$ NMR: 101 MHz, $CDCl_3$

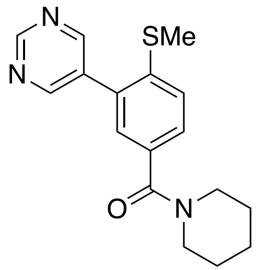

59

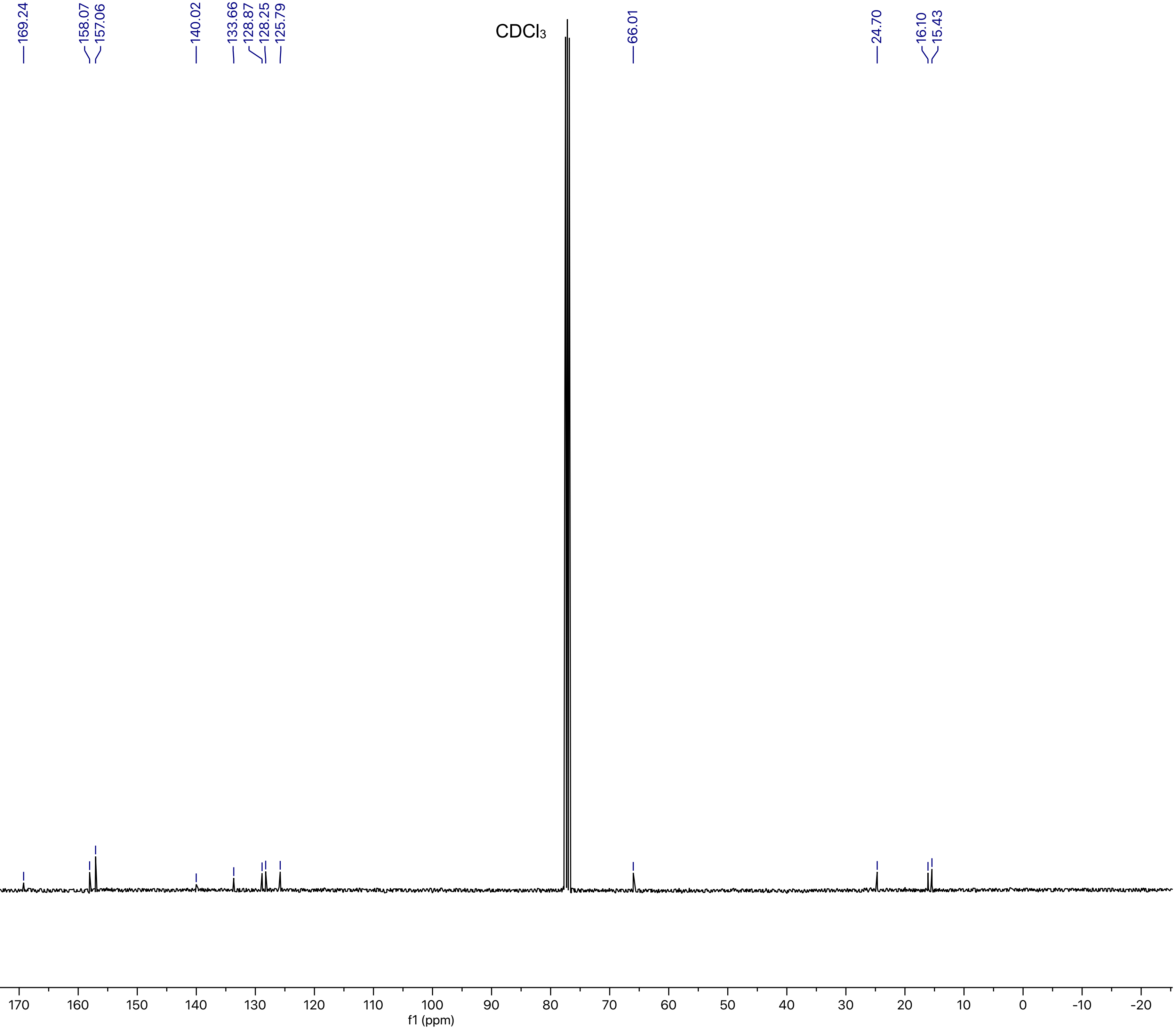

¹H NMR: 400 MHz, $CDCl_3$

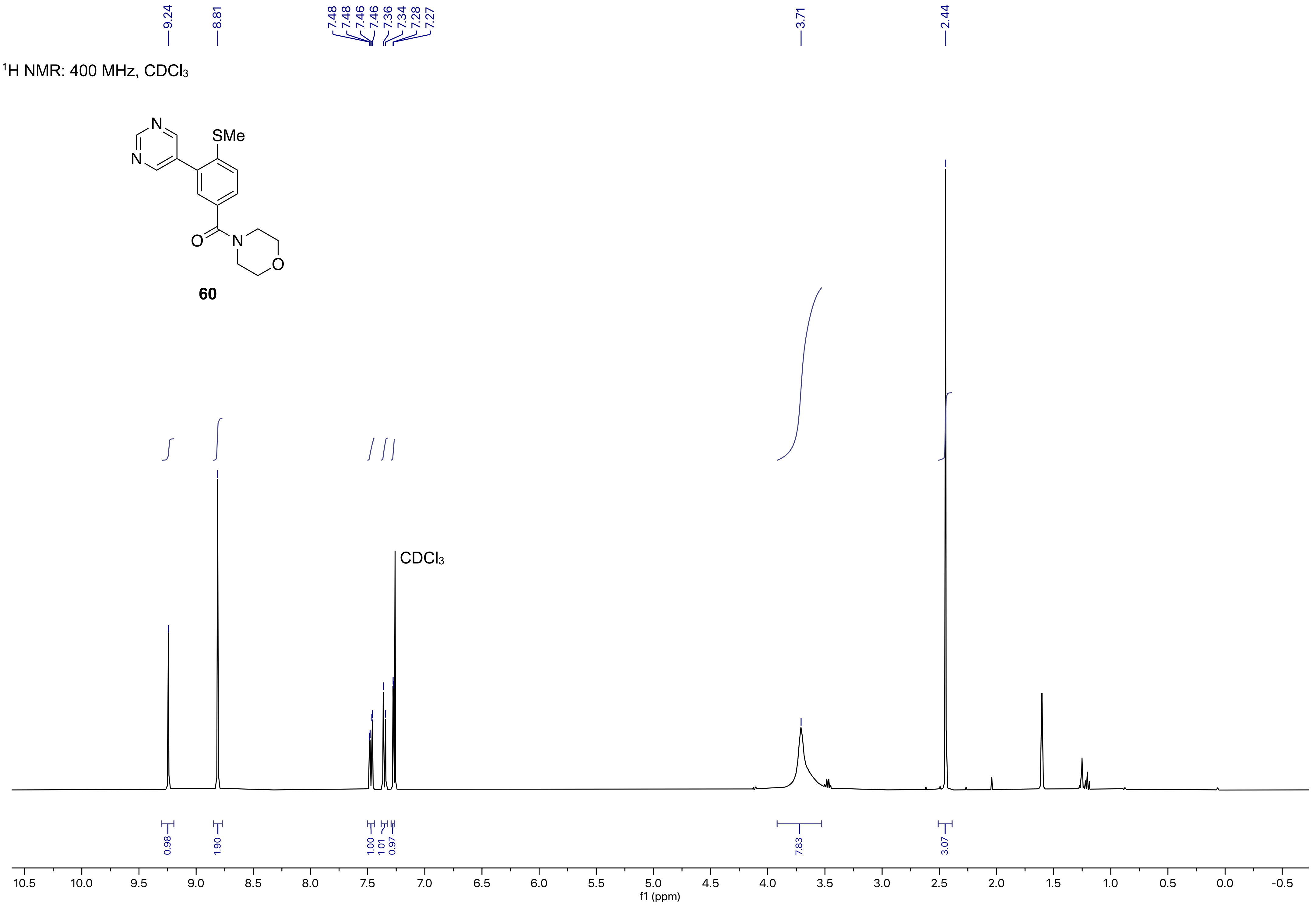

$^{13}C\{^{1}H\}$ NMR: 101 MHz, $CDCl_3$

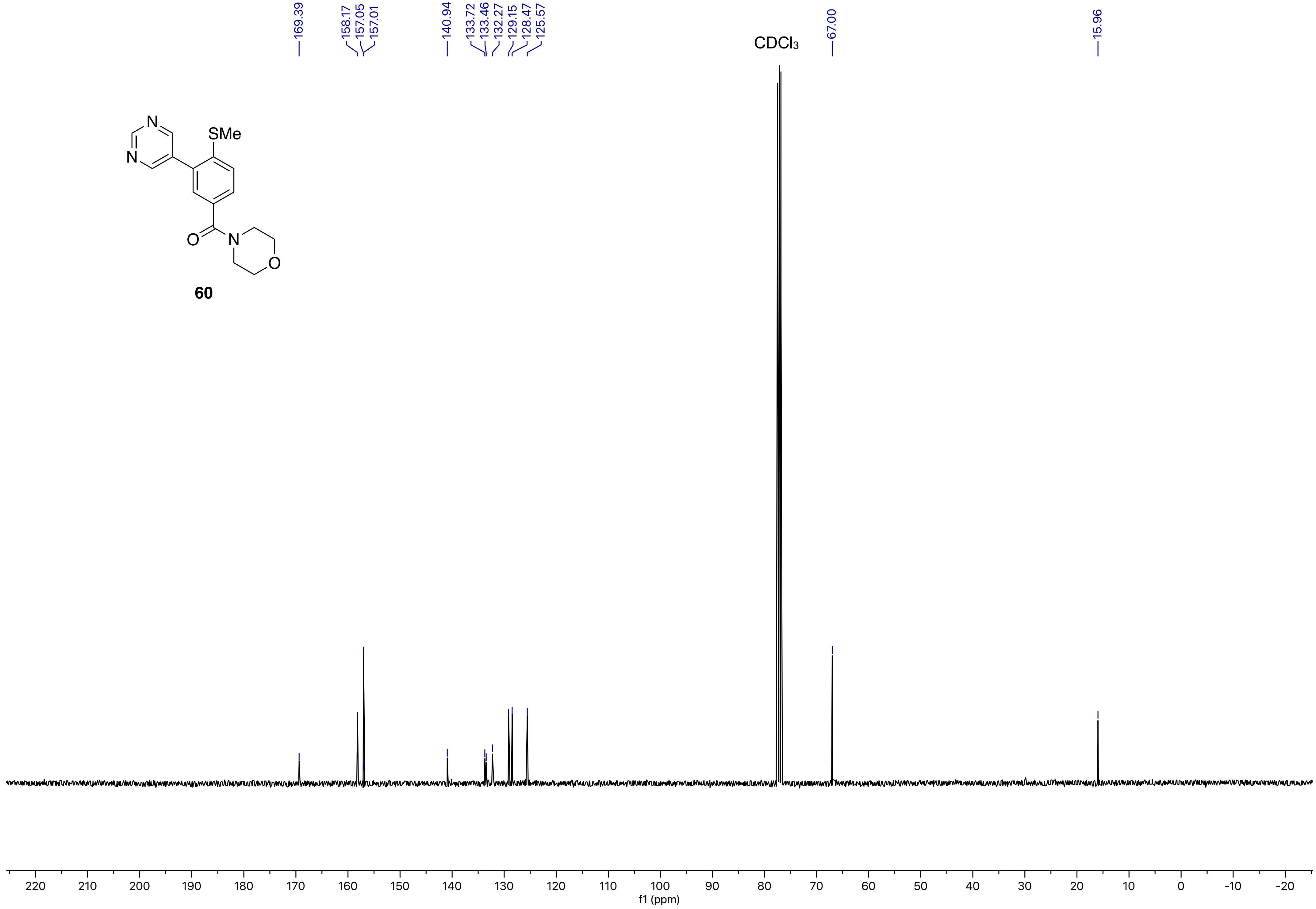

Supplement: Supplementary file 1 — ml3c00426_si_001.pdf [file ml3c00426_si_001.pdf]
